# Nickel-catalyzed hydro- and deuteroacylation of acrylates using water as the hydrogen source

**DOI:** 10.1039/d6sc04272d

**Published:** 2026-08-06

**Authors:** Ivo H. Lindenmaier, Fabian Bölzle, Ivana Fleischer

**Affiliations:** a Institute of Organic Chemistry, Faculty of Science, Eberhard Karls Universität Tübingen Auf der Morgenstelle 18 72076 Tübingen Germany ivana.fleischer@uni-tuebingen.de

## Abstract

Acylation of activated alkenes provides useful and quick access to synthetically useful 1,4-difunctionalized compounds. In this work, an operationally simple mechanochemical protocol for the hydroacylation of acrylates and related activated alkenes is reported using thioesters as acyl donors and water as the hydrogen source. This approach selectively furnished linear coupling products, complementing previous nickel-catalyzed methodologies that predominantly yielded branched 1,3-adducts. Substitution of water with D_2_O enables straightforward synthesis of deuterium-labeled products with high levels of selective isotopic incorporation. Detailed mechanistic investigations, including primary and secondary KIE studies, Hammett analysis, DFT computations and NMR spectroscopy, support a pathway involving migratory insertion at the nickel center, while providing no evidence for the involvement of radical intermediates.

## Abstract 2

We report a mechanochemical approach for hydroacylation of acrylates using thioesters and water under nickel catalysis. Mechanistic studies indicate that an ionic pathway involving a Ni(i)/Ni(iii) catalytic cycle is likely operative.

## Graphical abstract

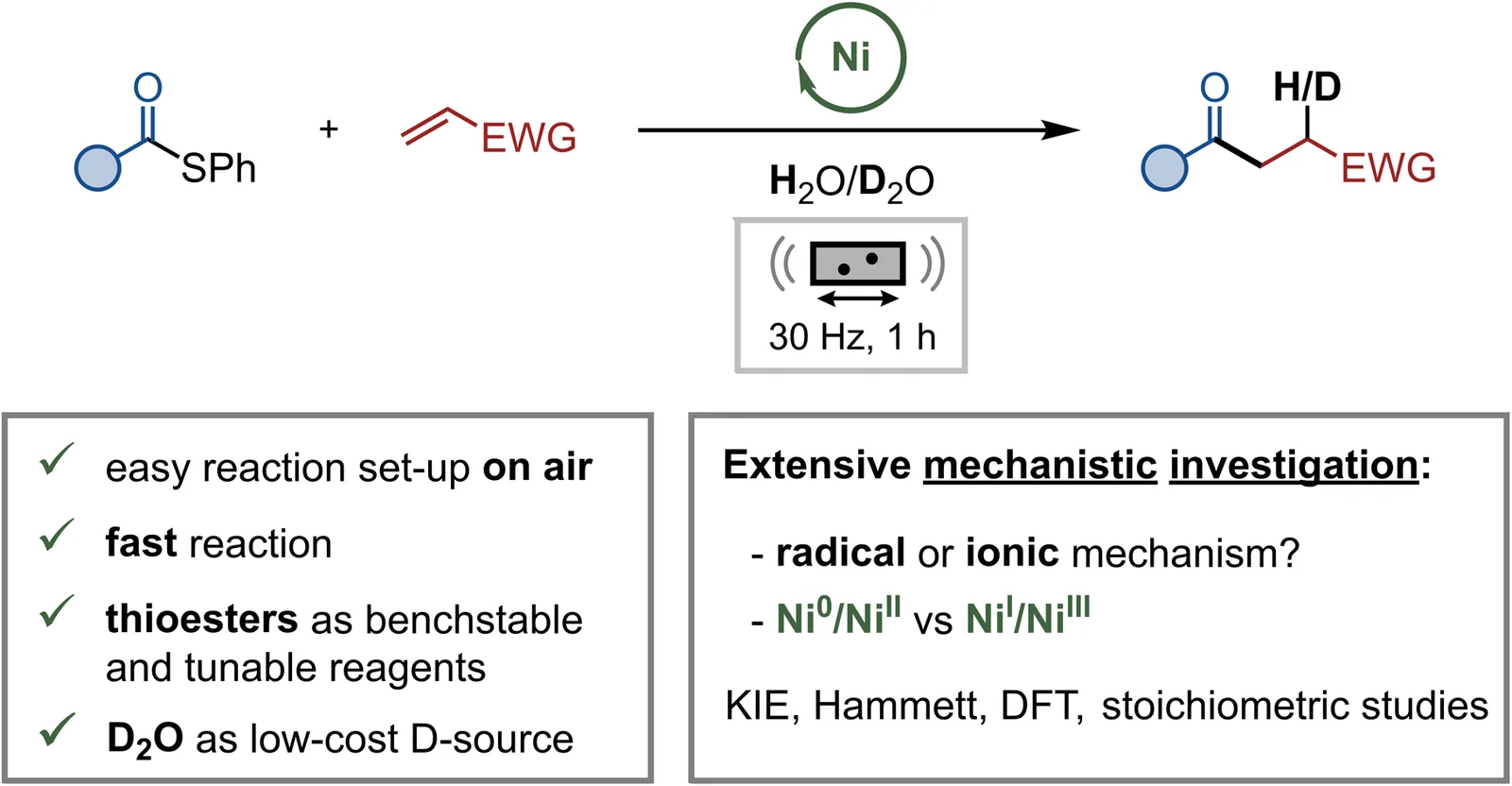

## Introduction

1,4-Dicarbonyl compounds are valuable intermediates for the synthesis of heterocycles and cyclopentenones,^1–3^ but their preparation is inherently challenging due to polarity mismatch, necessitating umpolung of either the alkene (*e.g.*, *via* homoenolate equivalents)^4,5^ or the acyl component (Scheme 1A). Traditional protocols for acyl umpolung are well known,^6,7^ but additional synthetic steps and strong bases are often needed, although organocatalysis offers an interesting alternative.^8–10^ Extensive efforts have been devoted to rhodium-catalyzed hydroacylation of alkenes using aldehydes (Scheme 1B),^11–13^ resulting in highly efficient protocols. However, the high cost of rhodium limits the practicality of these methods. More recently, attention shifted to the photochemical generation of acyl radicals that undergo Giese-type addition to activated alkenes, providing the linear 1,4-dicarbonyl coupling product after radical quenching.^14^ Such acyl radicals are typically generated *in situ* from aldehydes, α-ketoacids or, more rarely, carboxylic acid derivatives.^15–19^ Generally, these reactions provide an elegant approach towards 1,4-dicarbonyl compounds, but suffer from limited substrate availability, long reaction times and poor scalability. In addition, the nickel-catalyzed intermolecular hydroacylation of alkenes has been reported,^20,21^ including an enantioselective version.^22^ These examples rely on the use of silanes to forge a nickel-hydride catalyst, resulting in the branched 1,3-dicarbonyl coupling product. To the best of our knowledge, no nickel-catalyzed hydroacylation of acrylates has been reported that proceeds without photochemical activation, although related transformations have been described for 1,3-dienes, styrenes, and alkynes.^23–25^ In our continuous efforts to utilize thioesters as acyl donors,^26–29^ we noticed a side reaction that occurred in the mechanochemical cross-electrophile coupling (XEC) of thioesters with alkyl halides under nickel catalysis: In the presence of ethyl acrylate, the acylated Michael-adduct was formed next to the desired coupling product.^29^ Intrigued by this rather unusual reactivity that exploited the natural polarity of both coupling partners under reductive conditions, we decided to study this reaction in more detail. The previously established mechanochemical conditions represented an ideal starting point for our investigations, as they enable reaction setup at ambient conditions, without the need for inert atmosphere techniques. Instead, all reagents can be subsequently weighed into the milling jar and subjected to the ball-mill. Moreover, mechanochemistry can enhance reaction kinetics,^30^ selectivity^31^ and is usually seen as more sustainable compared to solution-based methods.^32^

**Scheme 1:**
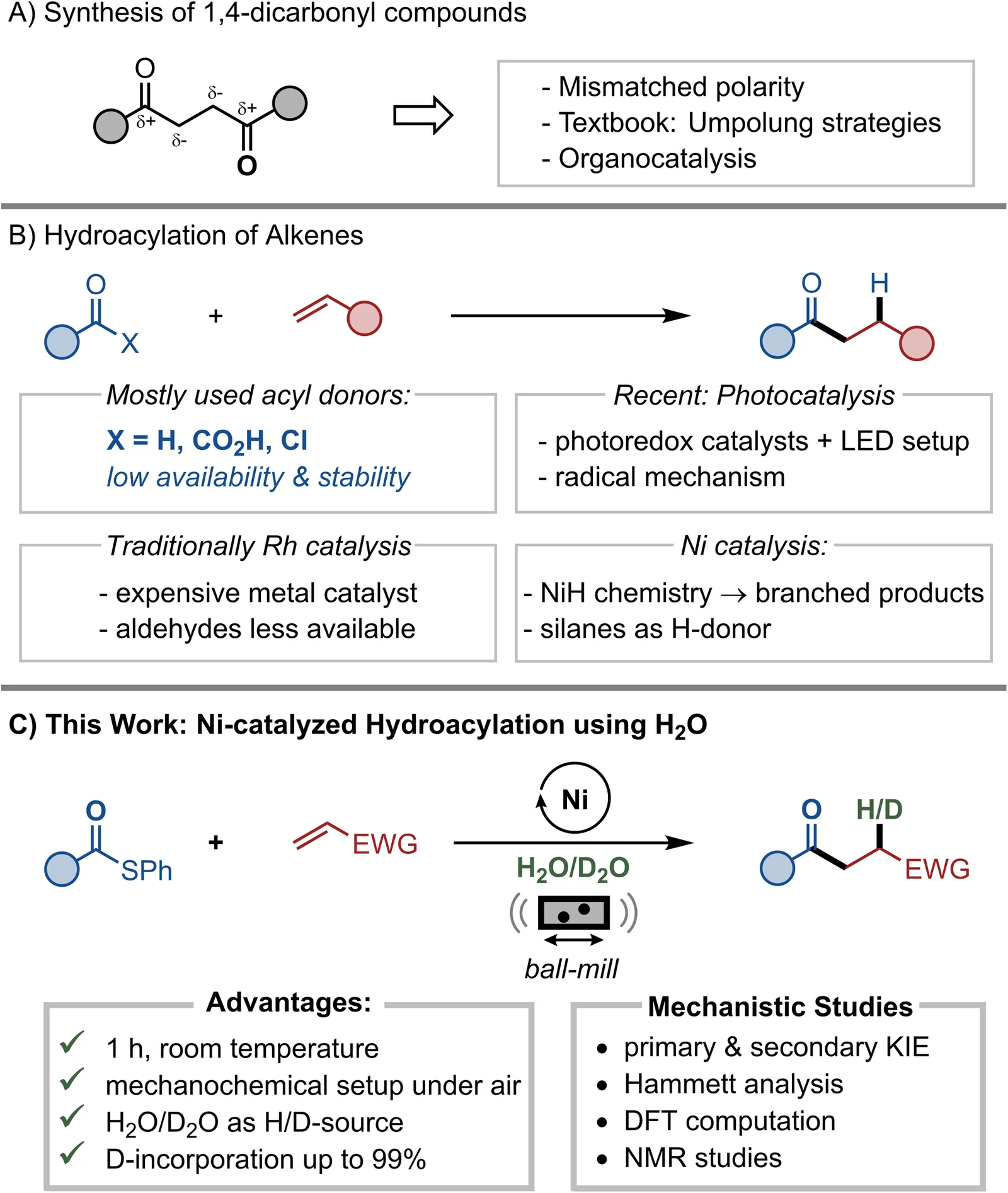
Overview of various methods for the hydroacylation of alkenes.

## Development & scope

Initial screenings utilizing a similar catalytic system as in XEC delivered the linear addition product in 18% GC yield (Table 1). Ligand screening revealed superior performance of electron-deficient bipyridines (entries 1–7), identifying 4,4′-bis(trifluoromethyl)-2,2′-bipyridine (L5) as the best ligand. L5 is commercially available but can also be synthesized in a one-step reaction on decagram scale (see SI Section 5.2). In contrast, alternative ligand classes, including phosphines and bis(oxazoline) derivatives, proved less effective (see SI). At this point we speculated that the added hydrogen atom would originate from the nickel precatalyst NiCl_2_(H_2_O)_6_. Thus, the substoichiometric amount of coordinated water might require an external source to achieve higher yields. Systematic solvent screening with L1 (entries 8–12) revealed that polar amide solvents, but also water afforded the highest yields of the coupling product, while other solvents resulted in diminished reactivity. Fine tuning of the solvent with L5 revealed a 5 : 1 mixture of DMF/H_2_O as the optimal medium (entry 14). It should be noted that solvents could be used straight from the bottle (HPLC grade) without the need for extensive deoxygenation or drying procedures. Upon performing the reaction under anhydrous conditions, no productive coupling was observed, and the starting materials were recovered. Control experiments confirmed the necessity of both nickel and ligand for productive reactivity, while the system showed low dependence on the specific nickel source, so we continued with NiCl_2_(H_2_O)_6_ as the most cost-effective precatalyst. In contrast, the influence of the mechanochemical setup is less transparent: replacing ball milling with magnetic stirring still afforded the coupling product, albeit in lower yield and at a substantially reduced reaction rate (see SI). Increasing the solvent volume to achieve solution-phase conditions further diminished the reaction efficiency. We propose that ball milling facilitates activation of the zinc metal,^33,34^ while also promoting efficient mixing under highly concentrated conditions.^35^

**Table 1:** Optimization screening^a^

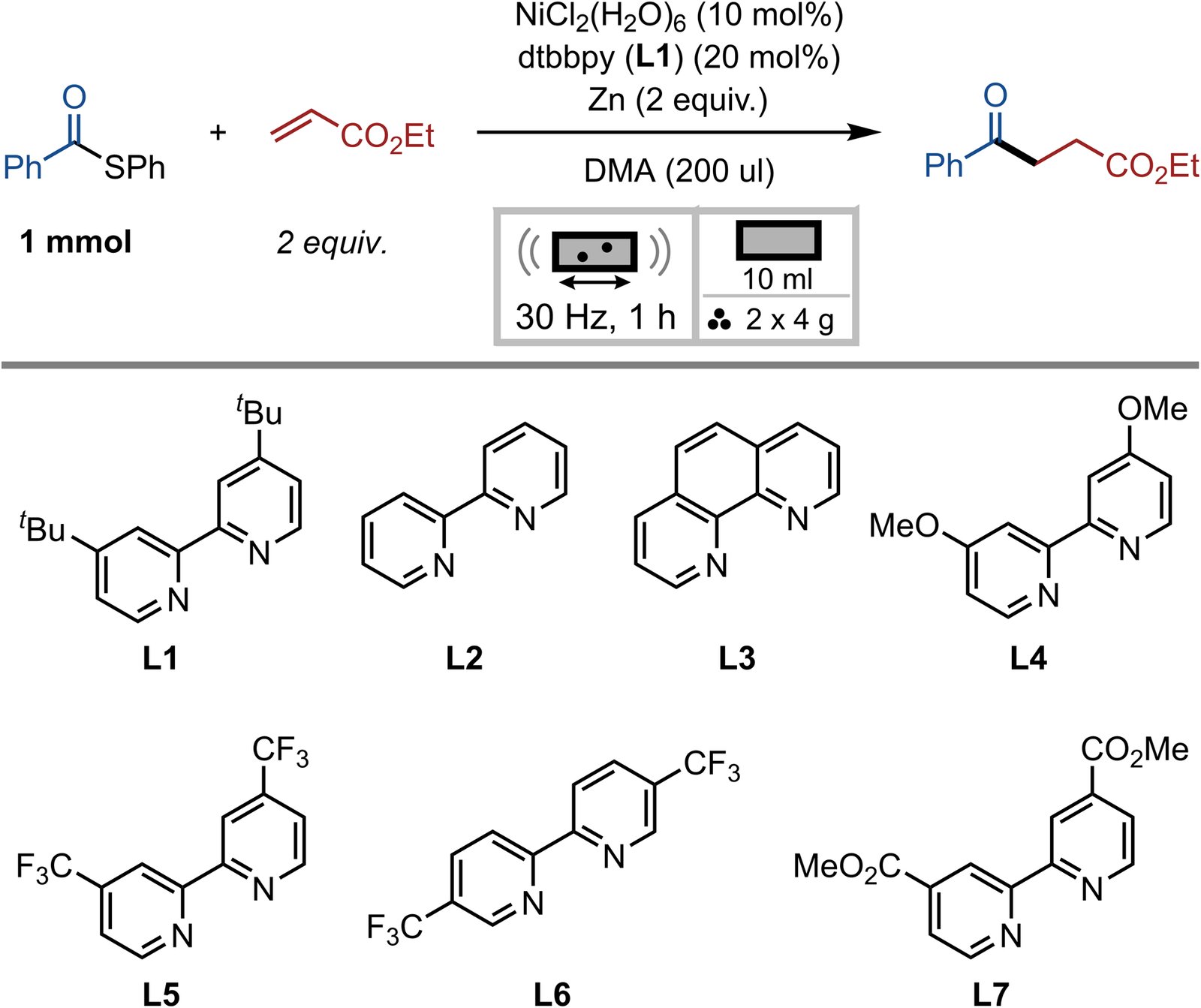			
Entry	Deviation	Conv. [%]	Yield [%]
1	None	39	18
2	L2	9	4
3	L3	2	1
4	L4	6	3
**5**	**L5**	**88**	**58**
6	L6	93	49
7	L7	76	43
8	DMF	28	15
9	H_2_O	30	16
10	MeOH	19	4
11	DCM	27	3
12	THF	8	3

**Lines below contain L5 (10 mol%) as ligand**			
13	DMF	94	59
**14**	**DMF/H**_**2**_ **O (5 : 1)**	**100**	**82 (77)^b^**

a Conversion and yields were determined by GC-FID using pentadecane as internal standard. Reaction conditions: thioester (1 mmol, 1 equiv.), ethyl acrylate (2 mmol, 2 equiv.), Ni(H_2_O)_6_Cl_2_ (10 mol%), ligand (20 mol%), Zn (2 equiv.), solvent (200 µl), SS milling jar (10 ml), SS milling balls (2 × 4 g), milling at 30 Hz for 1 h.

b Isolated yield.

Furthermore, addition of catalytic amounts of Cp_2_TiCl_2_, likely acting as a Lewis-acid,^28,36^ did not greatly vary the yield of 1, but was advantageous for functionalized, especially electron-poor thioesters and was therefore employed for subsequent investigation of the substrate scope (see SI for more detailed screening results). Other acyl sources, including aroyl halides, anhydrides, and twisted amides, were also evaluated under the developed reaction conditions (see SI). However, these substrates afforded inferior yields, highlighting the optimal reactivity of thioesters in this transformation. An advantage of thioesters compared to commonly employed aldehydes is the easy access from widely available carboxylic acids, their increased stability under benchtop conditions and tunability through the *S*-substituent.

With optimized conditions at hand, we isolated 1 in 77% yield and turned our attention towards the generality and limitations of this system (Scheme 2). Various aryl thioesters posed feasible acyl sources, but a strong influence of the electronic properties of the aroyl moiety was observed. Electron-neutral substrates delivered the product in high yields (2–4), whereas more electron-withdrawing substituents led to diminished yields (5–7). Substituents exhibiting –M-effects (*e.g.*, esters, nitriles) suppressed reactivity entirely. On the other hand, electron-rich substrates generally gave high yields (8, 9). A heteroaromatic substrate afforded the coupling product in moderate yield (10), consistent with the electron-deficient nature of the pyridine moiety. Meanwhile, alkyl thioesters were also compatible, enabling access to aliphatic 1,4-ketoesters. Primary alkyl thioesters afforded the coupling product in high yields (11, 12), while secondary thioesters were feasible but required longer reaction times (13–16). Formation of a tertiary ketone 17 proceeded in low yield, showcasing the impact of steric hindrance. Thioesters derived from pharmaceutically relevant and natural compounds were synthesized from the corresponding carboxylic acids and used as substrates in the developed system, affording the corresponding 1,4-dicarbonyl compounds in good yields (18–20).

**Scheme 2:**
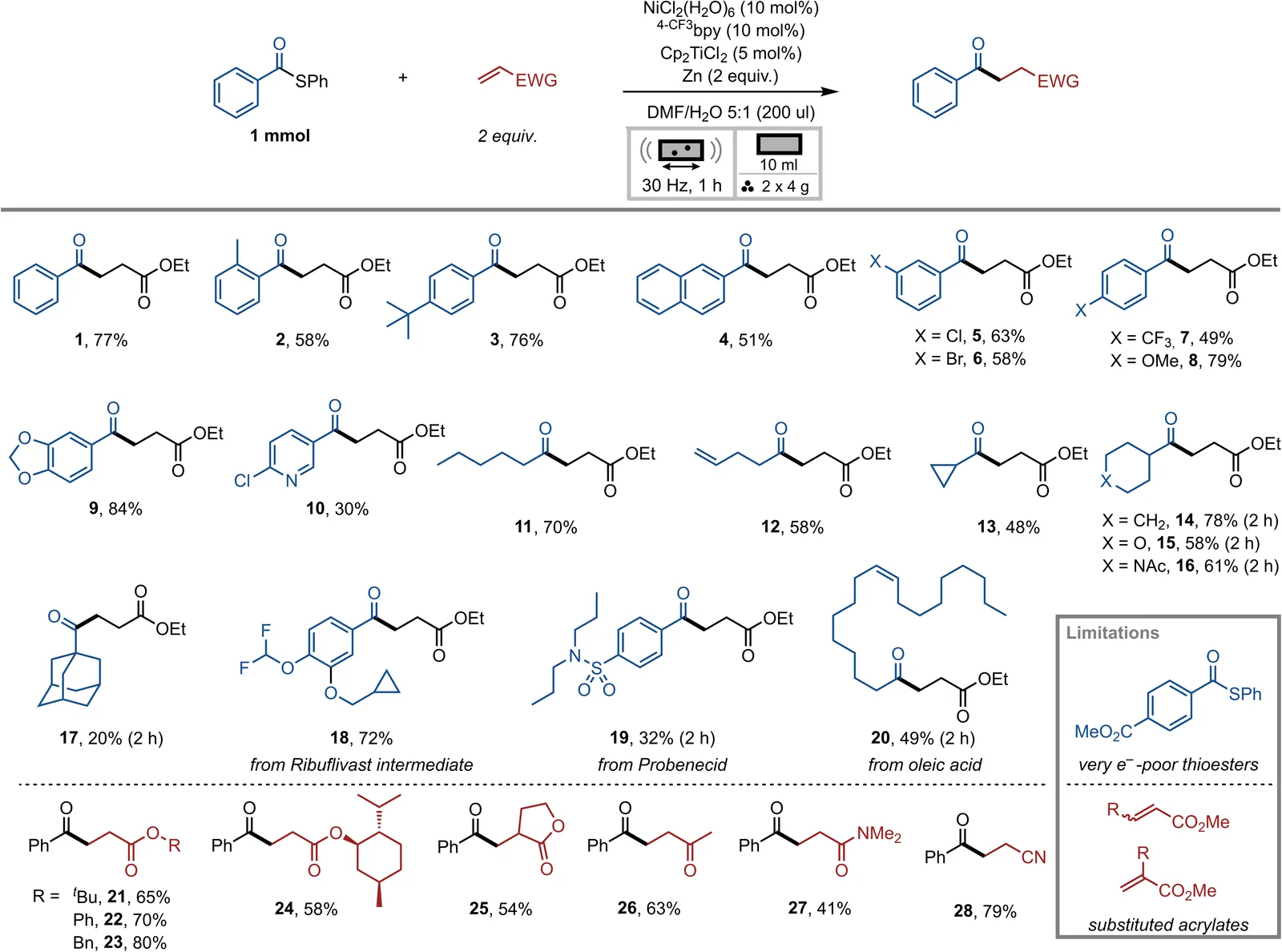
Scope of the nickel-catalyzed hydroacylation of activated alkenes. Isolated yields. Reaction conditions: thioester (1 mmol, 1 equiv.), acrylate (2 mmol, 2 equiv.), Ni(H_2_O)_6_Cl_2_ (10 mol%), L5 (10 mol%), Cp_2_TiCl_2_ (5 mol%), Zn (2 equiv.), premixed DMF/H_2_O (5 : 1, 200 µl), SS milling jar (10 ml), SS milling balls (2 × 4 g), milling at 30 Hz for 1 h.

In contrast, the scope of activated alkenes proved more sensitive towards steric hindrance: While a range of acrylates afforded high yields (21–24), substitution at the α- or β-position prevented product formation. In addition, alternative activating groups, including ketones (25), amides (26), and nitriles (27) were also compatible. In all examples, linear coupling products were obtained exclusively. Non-activated alkene derivatives, such as styrenes, were unreactive, regardless of the electronic properties on the aromatic moiety (see SI for examples). One possible reason could be that the steric constraint of the aromatic ring prevents effective coupling, as the reaction appeared to be highly sensitive to steric hindrance. Alternatively, polarization of the alkene or a coordinating functionality to stabilize a putative nickel–alkyl complex could be necessary (*vide infra*), as it was previously observed for the nickel-mediated migratory insertion of an acrylate into a Ni–C(sp^2^)-bond.^37^

## Deuteroacylation studies

Given that water appeared to be the hydrogen source in this reaction, we questioned if deuterium oxide, one of the widest commercially available sources for deuterium, would offer a straightforward strategy for selective deuteroacylation (Scheme 3). Indeed, after minor reoptimization of the nickel source and solvent, the deuterated 1,4-ketoester 1-D was received in 80% isolated yield with excellent and selective deuterium incorporation (99% D), as confirmed by HR-MS and ^1^H NMR spectroscopy. To the best of our knowledge, this constitutes the only thermal method for the synthesis of 3-deutero-1,4-dicarbonyl compounds. Comparable photochemical protocols suffer from lower deuterium incorporation and challenging scale-up.^18,38^ The studied scope demonstrated the generality of this approach, providing deuteroacylated products (1-D, 6-D, 9-D, 28-D) in yields comparable to the standard protocol and consistently high levels of deuterium incorporation. Notably, aryl bromide functionalities remained intact under the reaction conditions (6-D), providing a convenient handle for subsequent cross-coupling transformations, enabling synthesis of larger structures containing the C3-deuterated 1,4-ketoester motif. The formation of 29-D further demonstrated that more complex acrylates, such as estrone-derived acrylate, were suitable for deuteroacylation and furnished the product in good yield.

**Scheme 3:**
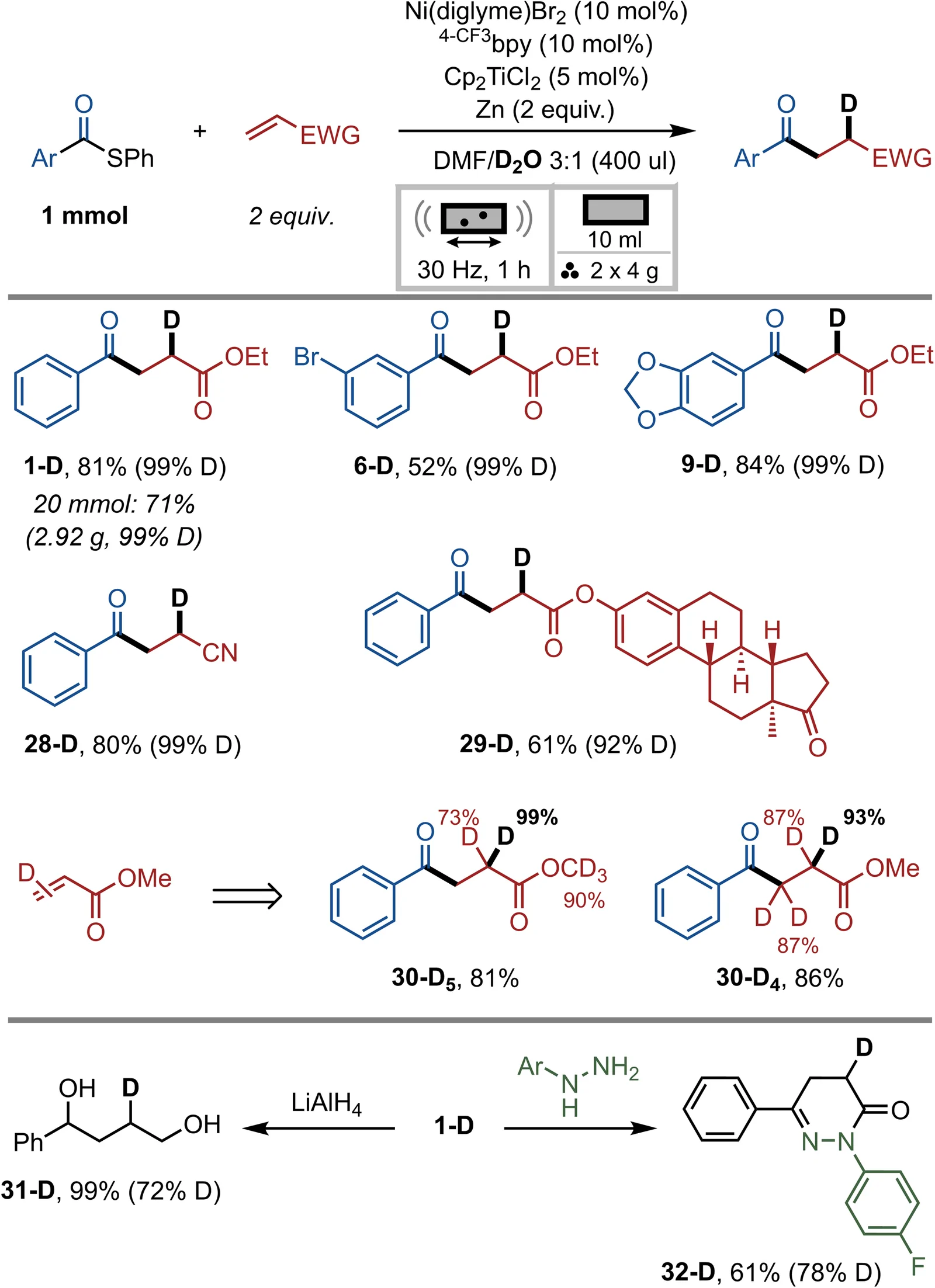
Scope of deuterated 1,4-ketoesters and derivatization of products. Isolated yields. Deuterium content was determined by ^1^H NMR. Reaction conditions: thioester (1 mmol, 1 equiv.), acrylate (2 mmol, 2 equiv.), Ni(diglyme)Br_2_ (10 mol%), L5 (10 mol%), Cp_2_TiCl_2_ (5 mol%), Zn (2 equiv.), premixed DMF/D_2_O (3 : 1, 400 µl), SS milling jar (10 ml), SS milling balls (2 × 4 g), milling at 30 Hz for 1 h.

Use of deuterated acrylates afforded coupling products containing CD_2_-groups (30-D_4_, 30-D_5_), which represent valuable intermediates for the synthesis of deuterium-labelled pyrroles and furans. The degree of deuteration on non-participating positions reflected the starting material, indicating that no H/D exchange or enolization occurred during the reaction.

Further derivatization of deuterated 1,4-ketoester 1-D demonstrated the utility of this methodology for the preparation of labelled compounds (31-D, 32-D), although partial H/D-exchange was observed under basic conditions.

## Mechanistic investigations

Next, we decided to investigate the reaction mechanism. Specifically, it remained unclear if a radical mechanism involving a Giese-type addition of an acyl radical to the alkene, or an ionic mechanism mediated by nickel was operative (Scheme 4A). A mechanism involving nickel hydride intermediates, as proposed in related systems, seemed unlikely as they typically afford branched rather than linear products.^39^ Initially, a primary kinetic isotope effect (KIE) was studied, to provide information about the possible coupling mechanism (Scheme 4B). Due to the limitations of mechanochemical setups for precise rate measurements, competition experiments at low conversion were utilized. From competition between H_2_O/D_2_O a primary KIE = *k*_H_/*k*_D_ = 3.1 was determined. Given that hydrogen atom abstraction by organic radicals from protic sources typically exhibits KIE values ≥ 6,^40^ these results argue against a radical pathway. Instead, the observed KIE suggests a more complex quenching step, although the precise mechanism remains unresolved. To obtain more definitive mechanistic insight into the coupling step, we conducted secondary KIE (sKIE) studies. Such experiments are particularly informative in reactions involving changes in carbon hybridization, such as at alkene carbons.^41^ In the present case, sKIE analysis can differentiate between the two proposed mechanisms by probing carbon rehybridization: In a radical mechanism, initial rehybridization would be expected to occur only at the β-carbon, as the unpaired electron resides in a p-orbital at the α-position.

**Scheme 4:**
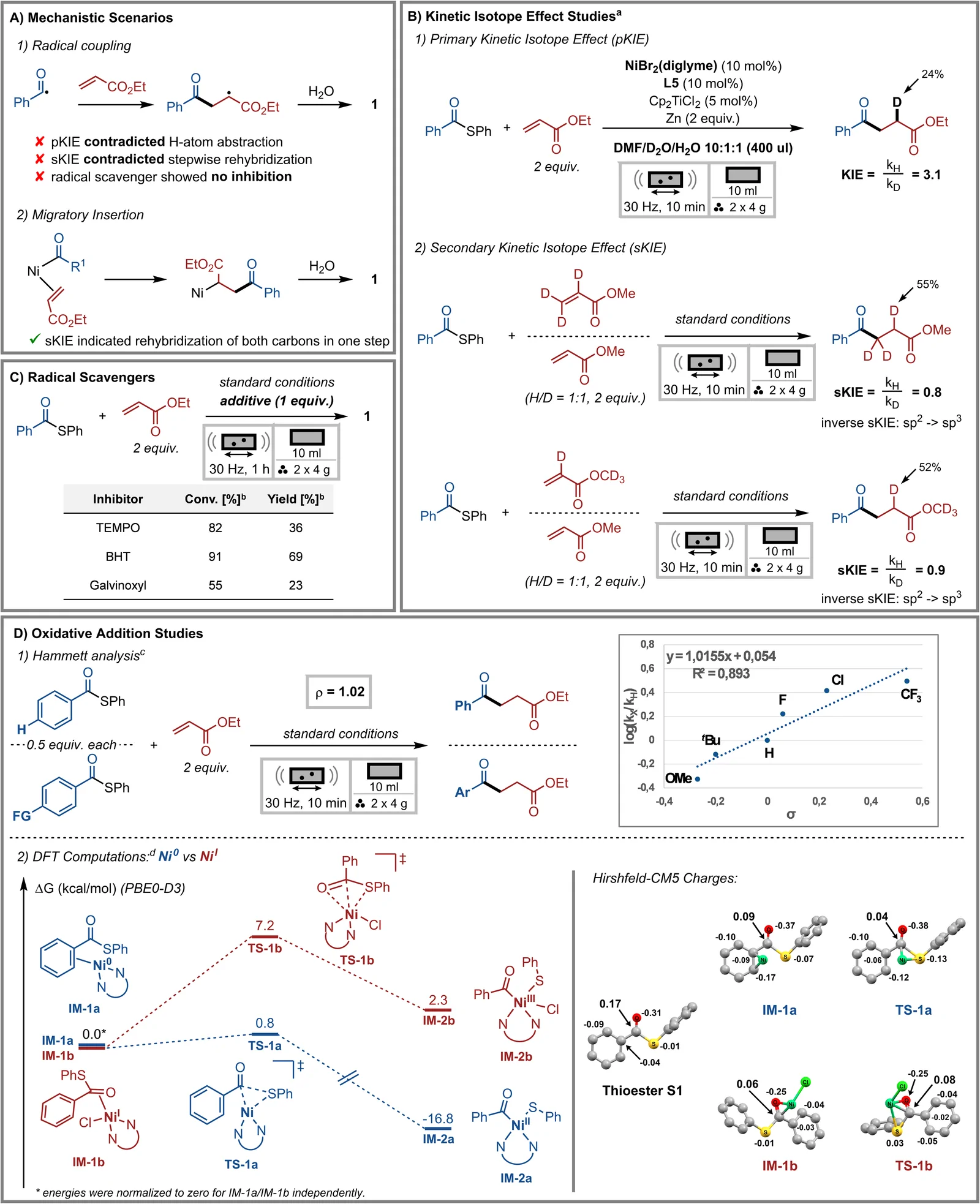
Mechanistic studies of coupling steps. ^*a*^KIE was determined from product ratios (^1^H NMR) after isolation. ^*b*^Yields determined by GC-FID using pentadecane an internal standard. ^*c*^Ratio of products determined by ^1^H NMR from an isolated sample. ^*d*^Calculations were performed at the PBE0-D3(BJ)/def2-TZVP(Ni), def2-SVP (other atoms), SMD(DMF) level of theory. Each reaction pathway was normalized independently by setting its respective starting structure to Δ*G* = 0. Consequently, the absolute energies of the two pathways cannot be compared directly.

Competition between methyl acrylate and its α,β-deuterated analogue resulted in an inverse secondary KIE of *k*_H_/*k*_D_ = 0.8, consistent with a rehybridization of the carbon centers from sp^2^ to sp^3^ in the product-determining step.^41^ A complementary experiment, employing an α-deuterated acrylate gave an sKIE of 0.9. Together, these findings indicate that both carbon atoms of the alkene are rehybridized in the same product-determining step. This is conclusive with a migratory insertion of the alkene into a Ni–acyl bond, but inconsistent with a radical mechanism, where only the β-carbon is rehybridized initially.

Additional evidence against radical intermediates was obtained from inhibition experiments (Scheme 4C). The presence of radical scavengers such as (2,2,6,6-tetramethylpiperidin-1-yl)oxyl (TEMPO), butylhydroxytoluene (BHT), or galvinoxyl reduced the yield but did not fully suppress product formation, further supporting a non-radical pathway, albeit the competitive radical acceptor ability of acrylate may question the reliability of these experiments.^42^ Our empirical observations on substrate scope and limitations are also consistent with a migratory insertion mechanism, which should proceed faster with electronically polarized alkenes, where the electrophilic β-carbon is susceptible to nucleophilic attack. In a mechanistic simplification, the Ni–acyl intermediate can be considered as a “Ni^+−^C(O)R” equivalent, and the acyl fragment would undergo nucleophilic addition to the β-carbon,^43^ thereby accounting for the observed perfect regioselectivity. In contrast, a radical pathway would be expected to also enable productive coupling with styrene substrates, as known from related photochemical methods.^18,44,45^ Moreover, we decided to study the oxidative addition in more detail (Scheme 4D). Once again utilizing low-conversion competition experiments, a Hammett study was performed using *p*-substituted benzoate thioesters. It gave rise to a *ρ*-value of 1.02, indicating the accumulation of a negative charge in the product-determining step at the carbonyl moiety. Although comparable studies for acyl electrophiles are scarce, we see this relatively small positive value in best agreement with a concerted oxidative addition, considering that the oxidative addition of aryl halides to Ni(0) gives rise to *ρ*-values around ∼2.^46,47^ Yu and co-workers reported in a computational study that in the oxidative addition of thioesters to palladium(0), the C–S-bond activation barrier is lowered by electron-withdrawing substituents,^48^ which is in line with our results.

To gain more insight in the nickel-catalyzed C–S-bond activation step, the oxidative addition of *S*-phenyl benzothioate to (L5)Ni(0) was studied by density functional theory (DFT, Scheme 4D). The concerted oxidative addition of the C–S bond appeared to be nearly barrierless (0.8 kcal mol^−1^) at the PBE0-D3(BJ)/def2-TZVP(Ni), def2-SVP (other atoms), SMD(DMF) level of theory, consistent with reported oxidative addition of *S*-alkyl thioesters to a comparable nickel(0) complex.^49^ Comparison of atomic charges at the carbonyl atom in the thioester, intermediate IM-1 and the transition-state TS-1 revealed depletion of positive charge (*i.e.* accumulation of negative charge), which is in agreement with the experimental Hammett results. As we found later evidence for a Ni(i)/Ni(iii) catalytic cycle (*vide infra*), the oxidative addition of a thioester to the corresponding (L5)Ni(i)–Cl complex was equally studied. Comparable to previous studies,^49^ our computational analysis revealed a competitively low barrier of oxidative addition (7.2 kcal mol^−1^). Meanwhile, depletion of positive charge was only observed for substrate coordination but was surprisingly absent for the oxidative addition. As expected, the lower stability of Ni(iii) compared to Ni(ii) render the oxidative addition to Ni(i) slightly endergonic. These results make both mechanisms possible, and the viability of oxidative addition to either Ni(0) or Ni(i) was also validated in stoichiometric experiments (see below).

Further mechanistic insight was obtained from NMR studies of organometallic intermediates (Scheme 5). Mixing Ni(cod)_2_ and L5 with methyl acrylate in DMF-d_7_ led to upfield-shifted and broadened alkene signals, indicative of coordination to nickel(0). Interestingly, the chemical shift of the alkene protons was dependent on the amount of acrylate added, showcasing that a dynamic equilibrium could be present. Indeed, low-temperature NMR of the reaction of (L5)Ni(cod) with 4 equiv. acrylate revealed that this equilibrium could be frozen at −40 °C, showing distinct signals for coordinated and unbound acrylate. On preparative scale, a Ni(0)-acrylate complex (Ni0) was isolated, although solution-phase NMR indicated the presence of multiple species, likely isomers. Notably, Ni0 showed no reactivity toward water over 24 h at room temperature.

**Scheme 5:**
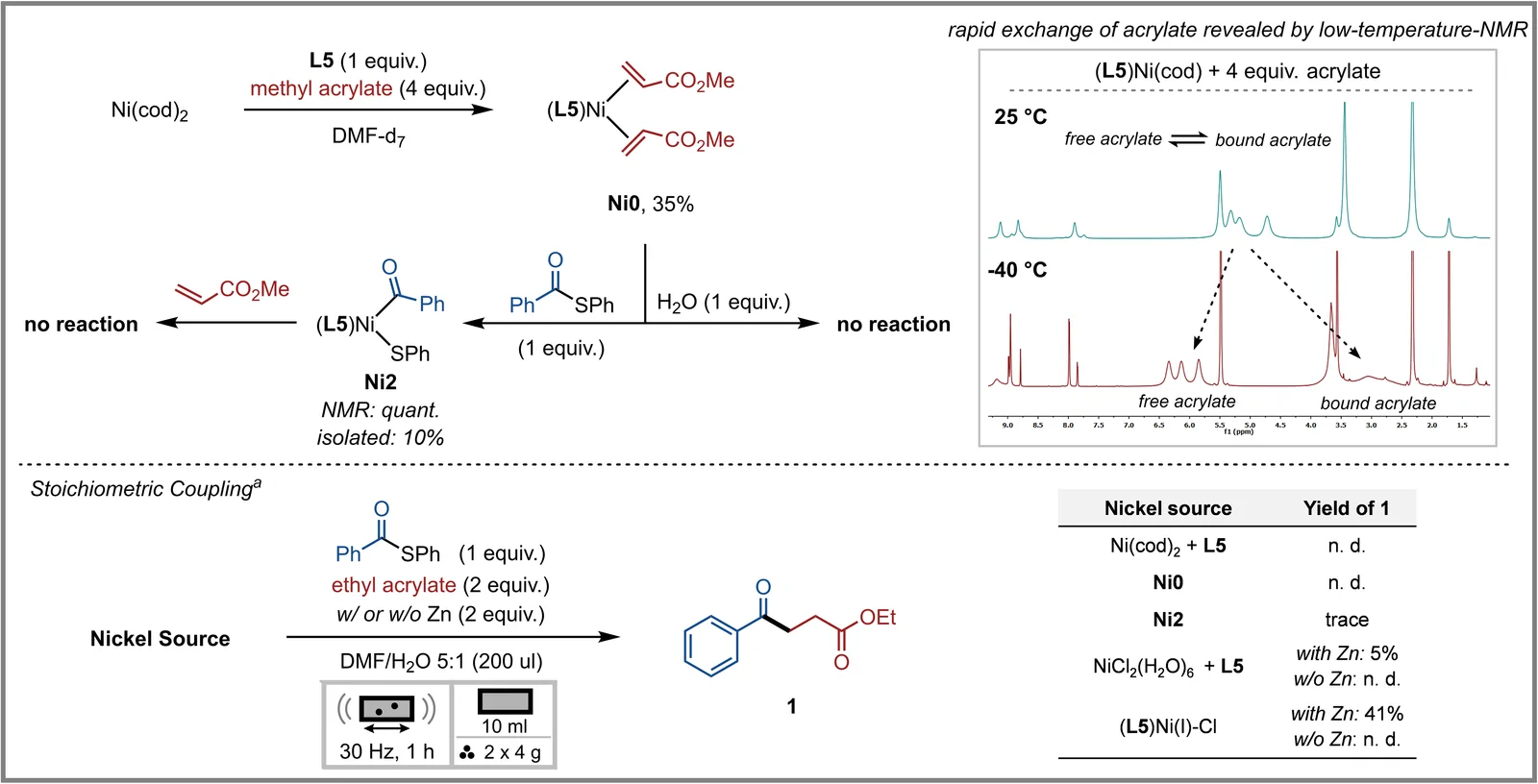
Synthesis and reactivity of potential nickel intermediates. ^*a*^GC-FID yields using pentadecane as internal standard.

On the other hand, adding 1 equiv. of thioester to Ni0 resulted in the formation of the oxidative addition complex Ni2, releasing the acrylate, as observed from the alkene proton chemical shift. To our surprise, no reaction of Ni2 with the acrylate occurred, neither in the presence nor in the absence of water and/or zinc, indicating that this complex represented an off-cycle species and is not an intermediate of the catalytic cycle. In following stoichiometric coupling experiments, various nickel sources were subjected to stoichiometric amounts of thioester and acrylate, either in the presence or absence of zinc. Surprisingly, no reaction occurred starting from nickel(0) sources, such as Ni(cod)_2_ or Ni0, indicating that this oxidation state may not be part of the catalytic cycle, conclusive with the lack of reactivity of oxidative addition complex Ni2. Another unexpected result was the inability of NiCl_2_(H_2_O)_6_ to promote the coupling in stoichiometric manner, even though it performed well when used in catalytic amounts. In contrast, independently synthesized nickel(i) complex (L5)Ni(Cl) (Ni1) furnished the coupling product in moderate yield, indicating that productive coupling relied on a Ni(i) complex. In agreement with our computational findings, addition of 1 equiv. of thioester to Ni1 in THF-d_8_ at room temperature led to the disappearance of its signals in the paramagnetic ^1^H NMR spectrum (see SI). Opposing to common proposals in XEC where zinc serves to close the catalytic cycle by reducing inactive species after product release,^50^ the observation that zinc is required even under stoichiometric conditions implied that a reduction step occurs prior to product formation. Thus, we propose the following catalytic cycle (Scheme 6): initial reduction of the precatalyst NiCl_2_(H_2_O)_6_ yields nickel(i) complex I.

**Scheme 6:**
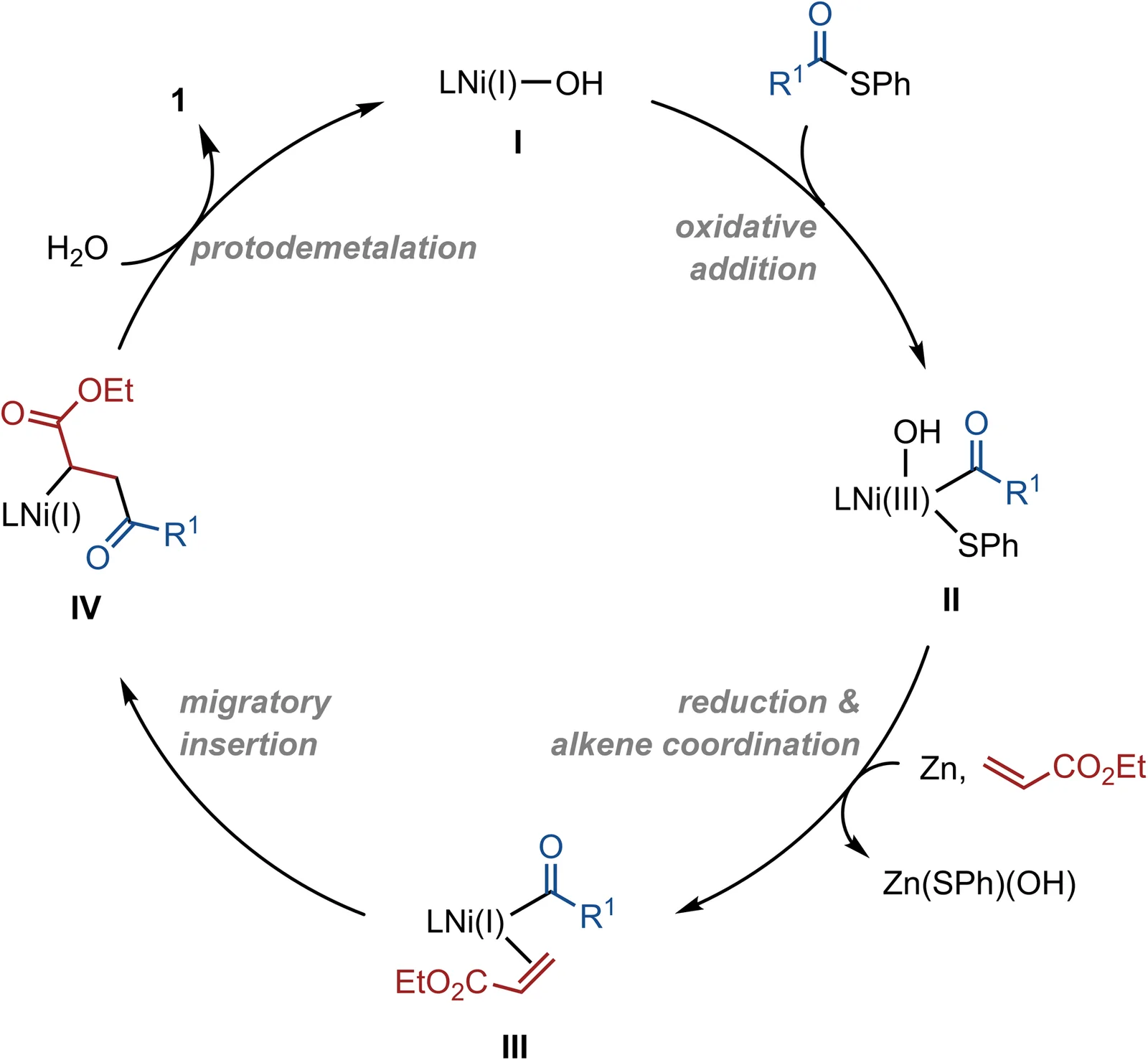
Proposed mechanism.

Oxidative addition of the thioester to Ni(i) affords high-valent Ni(iii) intermediate II. Subsequent reduction by zinc and alkene coordination leads to intermediate III, which transforms to IV after migratory insertion of the alkene into the Ni–acyl bond. According to our experimental data, the alkene does not coordinate to a Ni(ii) oxidative addition complex (Ni2), which therefore represents a catalytically inactive species. Protodemetalation of Ni–alkyl intermediate IV yields the coupling product and regenerates the active catalyst I. The regioselectivity of the insertion is controlled by the coordination of the acrylate carbonyl moiety, as proposed in the literature.^21^

## Conclusions

A mechanochemical procedure for the hydro- and deuteroacylation of activated alkenes using thioesters as acyl source is reported. Key features are the tolerance towards ambient conditions, short reaction times and the general use of water or deuterium oxide as H- or D-source, respectively. The applicability was demonstrated on the synthesis of a range of 3-deutero-1,4-ketoesters, which could also be obtained in gram scale with excellent and selective deuterium incorporation, offering a broad entry for synthesis of deuterated heterocycles. Mechanistic investigations suggested an ionic Ni(i)/(iii) mechanism without the involvement of radicals.

## Author contributions

I. H. L. conceived the project with help from I. F., conducted the experimental work and data analysis, and wrote the SI. F. B. carried out computational analysis. I. F. provided funding and supervised the project. The manuscript was written through contributions of all authors.

## Conflicts of interest

There are no conflicts to declare.

## Supplementary Material

SC-017-D6SC04272D-s001

## Data Availability

All data supporting this article have been included as part of the supplementary information (SI). Supplementary information: general procedures, further conditions screening and compound characterization. See DOI: https://doi.org/10.1039/d6sc04272d.
