# Supplementary material for: Nickel-catalyzed hydro- and deuteroacylation of acrylates using water as the hydrogen source
Source: Chem Sci. 2026 Aug 6;17(38):18396–404. doi: 10.1039/d6sc04272d (PMC13464617; doi:10.1039/d6sc04272d)
Supplement: Supplementary file 1 [file SC-017-D6SC04272D-s001.pdf]

Supplementary Information (SI) for Chemical Science.
This journal is © The Royal Society of Chemistry 2026

Nickel-Catalyzed Hydro- and Deuteroacylation of Acrylates using Water as Hydrogen Source

SUPPORTING INFORMATION

Ivo H. Lindenmaier,[a] Fabian Bölzle,[a] and Ivana Fleischer[a*]

[a] Institute of Organic Chemistry, Faculty of Science,
Eberhard Karls Universität Tübingen, Auf der Morgenstelle 18, 72076 Tübingen, Germany
ivana.fleischer@uni-tuebingen.de

Table of Contents

1. General Information 3

1.1 Chemicals and General Techniques 3

1.2 Analytical Techniques 3

2. General Procedures 5

3. Screening Information 8

3.1 Ligand Screening 8

3.2 Liquid Assisted Grinding Additive Screening 10

3.3 Nickel-Source Screening 11

3.4 Influence of Cp_2TiCl_2 11

3.5 Reductant Screening 12

3.6 Additive Screening 12

3.7 Solvent Screening for Deuteroacylation 13

3.8 Reaction under anhydrous conditions 13

4. Mechanistic Studies 14

4.1 Kinetic Isotope Studies 14

4.2 Radical Scavengers 19

4.3 Hammett Studies 20

4.4 Organometallic Intermediates 22

4.5 Stoichiometric Coupling Experiments 25

5. Analytical Data 26

5.1 Synthesis of Nickel Complexes 26

5.2 Synthesis of Thioester 34

5.3 Synthesis of Acrylates 76

5.4 Synthesis of 1,4-Dicarbonyl Compounds 86

5.5 Synthesis of Deuterated Compounds 149

6. Computational Details 177

7. Unsuccessful Substrates 194

8. References 195

1. General Information

1.1 Chemicals and General Techniques

Mechanochemical XEC reactions and syntheses of starting materials were conducted without taking special precautions towards the reaction atmosphere unless denoted otherwise. Mechanistic experiments were carried out under an argon atmosphere using standard Schlenk techniques or an argon atmosphere Glovebox (GS MEGA E-Line, Glovebox Systemtechnik) and pre-dried glassware. Dry solvents (THF, *n*-hexane, DCM) were prepared using a solvent-purification-system (MB-SPS5, MBraun) and stored over 3 Å molsieves. Column chromatography was carried out on a Puriflash system (Interchim XS420) using pre-packed columns (30 or 50 µm) from Büchi. Thin Layer Chromatography was performed on silica gel coated glass plates (0.25 mm) with fluorescence indicator UV254 (Macherey-Nagel, TLC plates SIL G-25 UV254). For detection of spots, irradiation of UV light at 254 nm or a stain of $KMnO_4$ was used. Chemicals were purchased from abcr, Acros, Alfa Aesar, BLDChem, Carbolution Chemicals, Carl Roth, Fluorochem, Sigma-Aldrich or TCI and used as received.

Mechanochemical reactions were carried out in a MM400 mixer mill from Retsch in stainless steel milling jars (10 mL) using two stainless steel balls (4 g). The temperature of the jars typically rose to approximately 35 °C during the reaction due to kinetic energy. No precautions to heat or cool the device were taken.

1.2 Analytical Techniques

NMR spectra were recorded using Bruker Avance III HD 400 or Bruker Avance III HDX 700 at room temperature (400 or 700 MHz for ^{1}H experiments; 101 or 176 MHz for ^{13}C experiments; 376 MHz for ^{19}F experiments) in commercially available deuterated solvents. For mechanistic experiments, low-temperature NMR spectra were obtained with a TCI-cryo-probe. ^{13}C NMR experiments were performed with broadband proton-decoupling. Chemical shifts (*δ)* are reported in ppm relative to the residual NMR solvent signals (chloroform: ^{1}H 7.26 ppm and ^{13}C 77.16 ppm).[1] Hetero-NMR chemical shifts are given relative to external standards (^{19}F: $CFCl_3$). The coupling constants (*J* values) are given in Hz and spin multiplicity with the usual designations for splitting patterns (s = singlet, d = doublet, t = triplet, q = quartet, m = multiplet).

HR-MS (ESI, APCI) measurements were carried out by the mass spectrometry department of the Institute of Organic Chemistry, University of Tübingen. Measurements were carried out using maXis 4G from Bruker (ESI, APCI) or Q-exactive HF from Thermo Scientific (ESI). The molecular ions $[M+H]^+$ and $[M+Na]^+$ respectively are given in m/z units.

GC-FID (flame ionization detection) analysis was carried out on an Agilent 7820A system using dry hydrogen as carrier gas. An Agilent 19091J-431 column (30 m × 320 µm × 0.25 µm) was used. Program: heating from 35 °C to 280 °C within 53 minutes (5 °C/min, 4 min hold time). Conversion and yield were determined *via* calibration against the internal standard *n*-pentadecane.

Melting point determination was achieved by using a Büchi B-540 machine with a visual detection (heating rate 5 °C/min).

FT-IR spectra were recorded using a Cary 630 FTIR by applying the sample neat on a diamond ATR sampler.

2. General Procedures

General Procedure A1 (GP-A1): Synthesis of *S*-phenyl thioesters from acid chlorides.

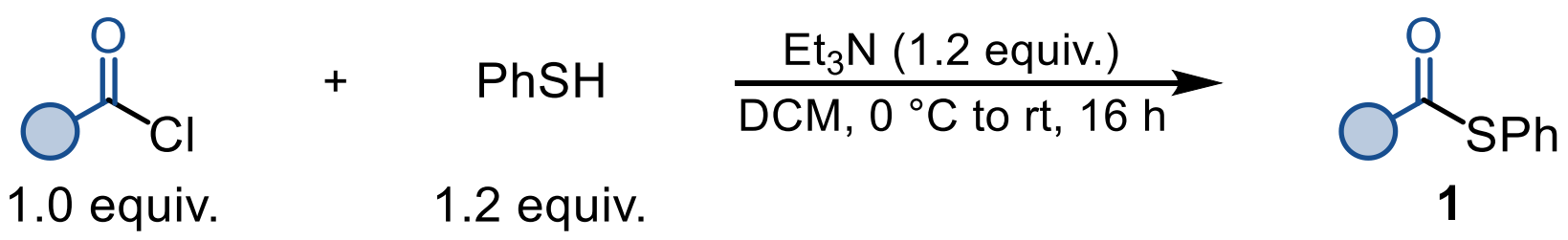

S-Phenyl thioesters were synthesized according to literature.[2] In a RBF, Et_3N (1.2 equiv.) and the respective acid chloride (1.0 equiv.) were dissolved in DCM (0.4 M) and cooled to 0 °C. Thiophenol (1.2 equiv.) was added under stirring and after 30 min, the mixture was allowed to warm to rt and stirred overnight. The reaction was quenched with water (20 mL) and the aqueous phase was extracted with DCM (2 × 30 mL). The combined organic phases were washed with aq. HCl (1 M, 20 mL), sat. aq. $NaHCO_3$ (20 mL), and brine (20 mL) and dried over $MgSO_4$. After filtration and solvent removal *in vacuo,* the crude product was purified by recrystallization or flash column chromatography.

Note: Schlenk conditions were applied for alkyl acid chlorides.

General Procedure A2 (GP-A2): Synthesis of *S*-phenyl thioesters by Steglich esterification.

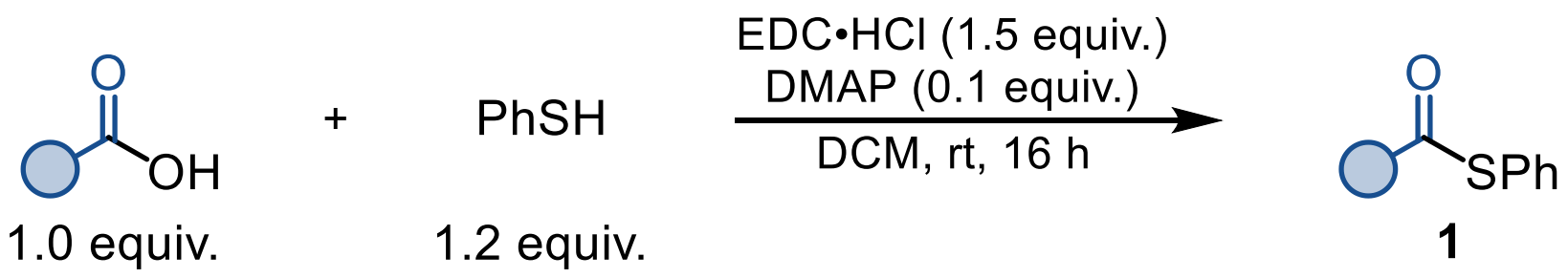

S-Phenyl thioesters were synthesized according to literature with slight modifications.[3] In a RBF under inert atmosphere, the respective carboxylic acid (10 mmol, 1.0 equiv.), EDC•HCl (1.5 equiv.), and DMAP (0.1 equiv.) were stirred in dry DCM (50 mL) for 10 min. Subsequently, thiophenol (1.2 equiv.) was added and the mixture was stirred overnight. The reaction was quenched with water (20 mL), and the aqueous phase was extracted with DCM (3 × 30 mL). The combined organic phases were dried over $MgSO_4$, concentrated *in vacuo* and purified by flash column chromatography.

General Procedure B (GP-B): Nickel-catalyzed hydroacylation of acrylates with *S*-phenyl thioesters under mechanochemical conditions (scope).

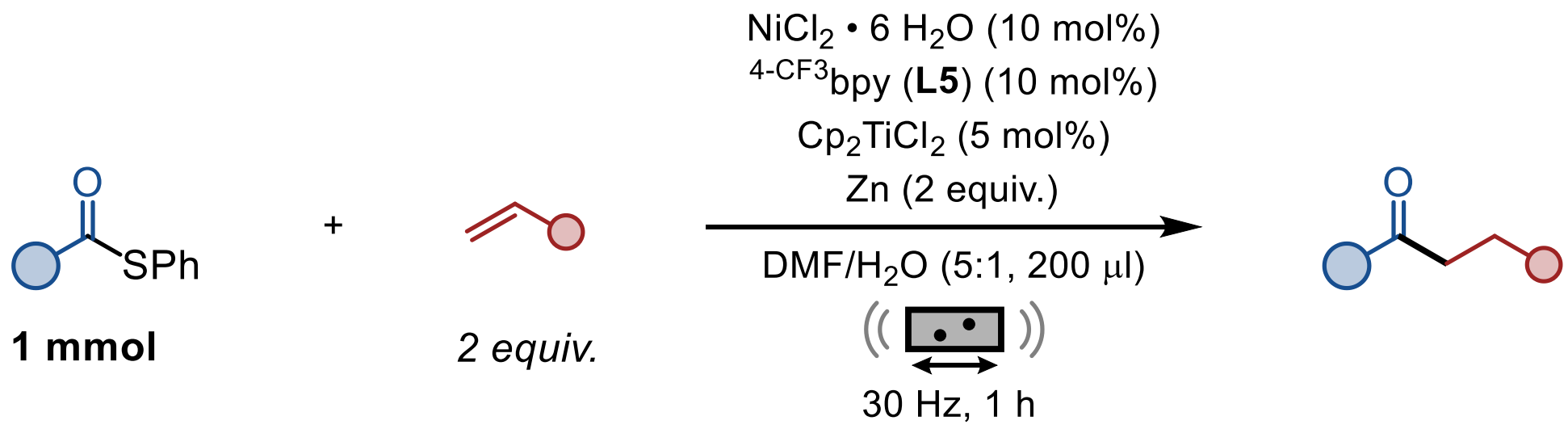

No efforts to exclude the ambient atmosphere from the reaction were made. DMF and water were HPLC grade.

A 10 mL stainless steel milling jar was charged with two stainless steel balls (⌀ = 10 mm, m = 4 g), $NiCl_2 \cdot 6H_2O$ (23.8 mg, 0.1 mmol, 10 mol%), 4,4'-bis(trifluoromethyl)-2,2'-bipyridine (**L5**, 29.2 mg, 0.1 mmol, 10 mol%), titanocene dichloride (12.5 mg, 0.05 mmol, 5 mol%), zinc (131 mg, 2.0 mmol, 2.0 equiv.), the respective thioester (1.0 mmol, 1.0 equiv.), the respective alkene (2.0 mmol, 2.0 equiv.), and a premixed solvent mixture of DMF/H_2O (5:1 v/v, 200 µL). The milling jar was closed, placed in a MM 400 and shaken at 30 Hz for 1 h. Subsequently, the jar was opened and rinsed with EtOAc (10 mL). Volatiles were removed *in vacuo* and the residue was subjected to flash column chromatography (*n*-hexane/EtOAc) for purification.

General Procedure C (GP-C): Nickel-catalyzed deuteroacylation of acrylates with *S*-phenyl thioesters under mechanochemical conditions.

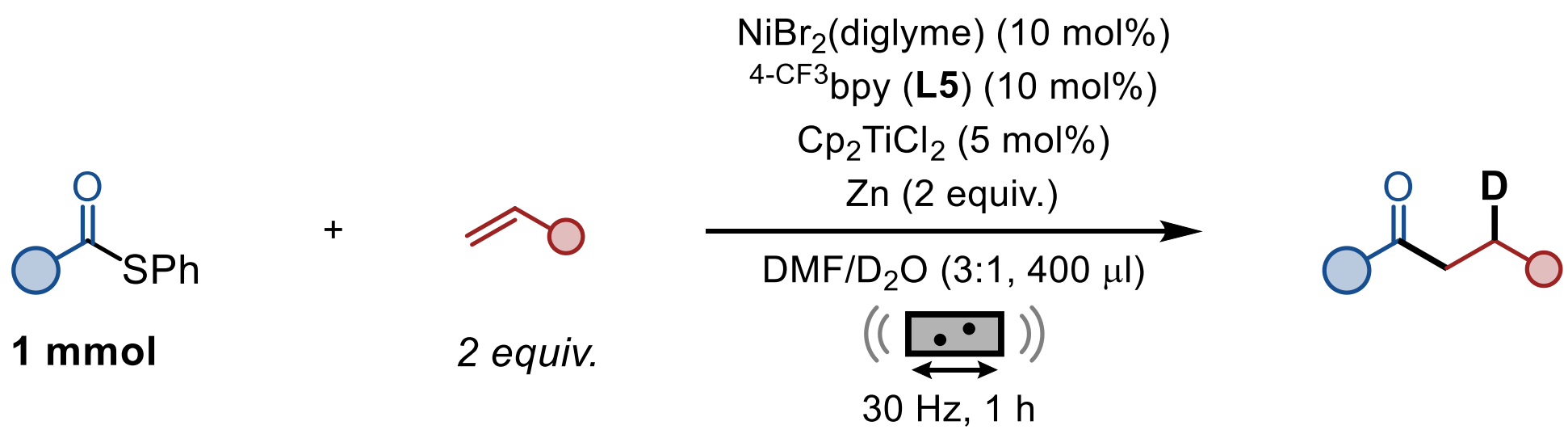

No efforts to exclude the ambient atmosphere from the reaction were made.

A 10 mL stainless steel milling jar was charged with two stainless steel balls (⌀ = 10 mm, m = 4 g), $NiBr_2$(diglyme) (35.2 mg, 0.1 mmol, 10 mol%), 4,4'-bis(trifluoromethyl)-2,2'-bipyridine (**L5**, 29.2 mg, 0.1 mmol, 10 mol%), titanocene dichloride (12.5 mg, 0.05 mmol, 5 mol%), zinc (131 mg, 2.0 mmol, 2.0 equiv.), the respective thioester (1.0 mmol, 1.0 equiv.), the respective alkene (2.0 mmol, 2.0 equiv.), and a premixed solvent mixture of DMF/D_2O (3:1

v/v, 400 μL). The milling jar was closed, placed in a MM 400 and shaken at 30 Hz for 1 h. Subsequently, the jar was opened and rinsed with EtOAc (10 mL). Volatiles were removed *in vacuo* and the residue was subjected to flash column chromatography (*n*-hexane/EtOAc) for purification.

3. Screening Information

3.1 Ligand Screening

Table S1. Ligand screening for the nickel catalyzed hydroacylation of *S*-phenyl thioesters with acrylates mediated by mechanochemistry.

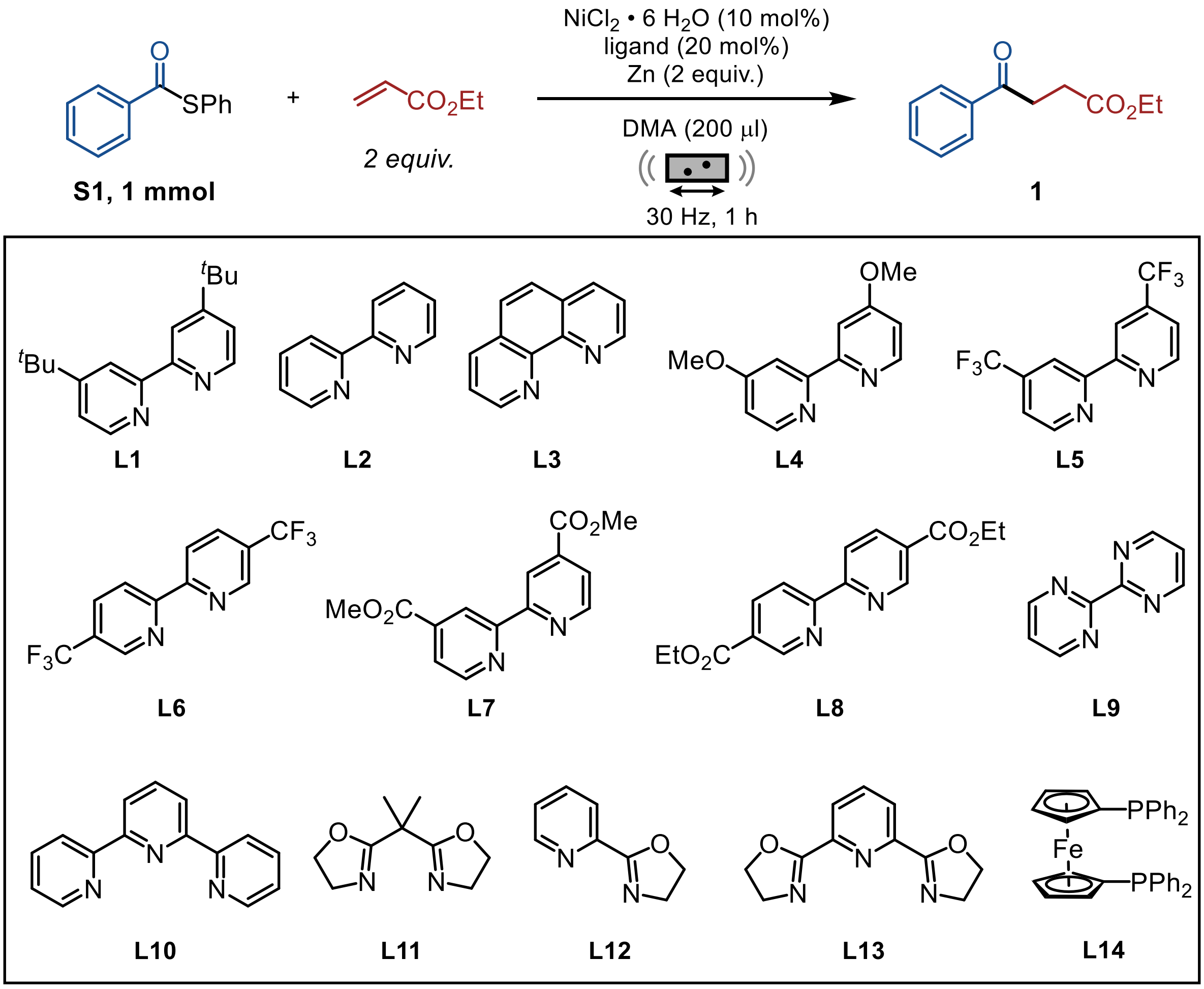

Entry	Ligand	Yield (%)[a]	Conversion S1 (%)[a]
1	dtbbpy (**L1**)	18	39
2	Bpy (**L2**)	4	9
3	phen (**L3**)	1	2
4	$^{4,4'\text{-OMe}}$bpy (**L4**)	3	6
5	$^{4,4'\text{-CF3}}$bpy (**L5**)	52	89
6	$^{5,5'\text{-CF3}}$bpy (**L6**)	49	93
7	$^{4,4'\text{-CO2Me}}$bpy (**L7**)	43	76
8	$^{5,5'\text{-CO2Et}}$bpy (**L8**)	24	62
9	2,2'-bipyrimidine (**L9**)	5	7
10	terpy (**L10**)	7	15
11	BOX (**L11**)	3	10
12	PyOX (**L12**)	22	48

13	PyBOX (**L13**)	9	38
14	PPh_3	5	14
15	dppf (**L14**)	0	11
16	**L5** (10 mol%)	57	85

Reaction conditions: **S1** (1 mmol, 1.0 equiv.), ethyl acrylate (2.0 equiv.), $NiCl_2(H_2O)_6$ (10 mol%), ligand (20 mol%), Zn (2 equiv.), DMA (200 µL), SS milling jar (10 mL), SS milling balls (2 × 4 g), MM (30 Hz, 1 h). [a]Determined by GC-FID using *n*-pentadecane as an internal standard.

3.2 Liquid Assisted Grinding Additive Screening

Table S2. LAG additive screening for the nickel catalyzed hydroacylation of *S*-phenyl thioesters with acrylates mediated by mechanochemistry.

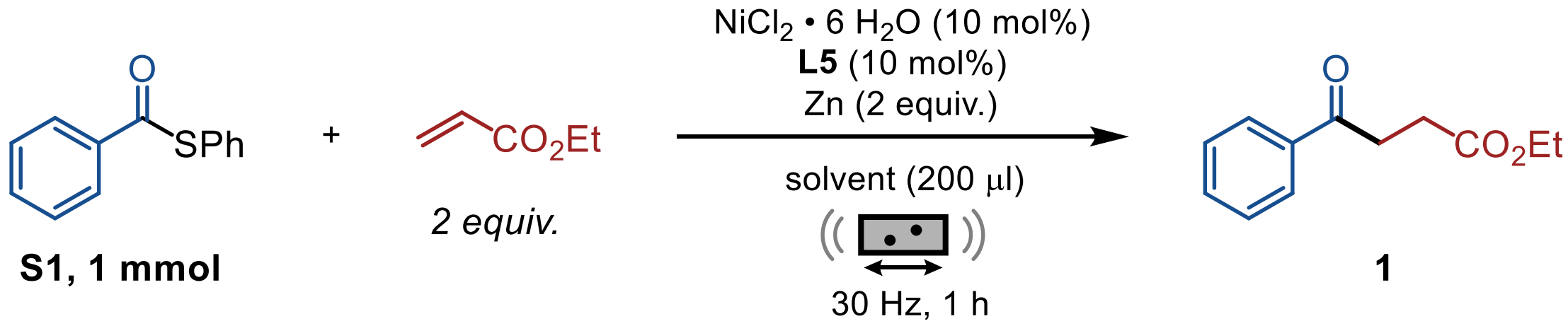

Entry	LAG	Yield (%)[a]	Conversion S1 (%)[a]
1	DMA	52	89
2	DMF	59	94
3	MeCN	7	35
4	H_2O	17	35
5	Toluene	6	18
6	DCM	3	31
7	Acetone	3	18
8	MeOH	4	24
9	Hexane	6	14
10	EtOAc	3	14
11	THF	3	14
12	–	9	24
13	DMF/H_2O (1:1)	67	94
14	DMF/H_2O (3:1)	77	98
15	DMF/H_2O (5:1)	82	100
16	DMF/H_2O (6:1)	77	100
17	DMF/H_2O (5:1, 100 µL)	78	100
18	DMF/H_2O (5:1, 400 µL)	83	100
19	DMF/H_2O (5:1) magnetic stirring instead MM	33	64
20	DMF/H_2O (5:1), 16 h magnetic stirring instead MM	81	97
21	DMF/H_2O (5:1, 1 ml), 16 h magnetic stirring instead MM	53	94
22	DMF/H_2O (5:1, 2 ml), 16 h magnetic stirring instead MM	30	45

Reaction conditions: **S1** (1 mmol, 1.0 equiv.), ethyl acrylate (2.0 equiv.), $NiCl_2(H_2O)_6$ (10 mol%), **L5** (10 mol%), Zn (2 equiv.), solvent (200 µL), SS milling jar (10 mL), SS milling balls (2 × 4 g), MM (30 Hz, 1 h). [a]Determined by GC-FID using *n*-pentadecane as an internal standard.

3.3 Nickel-Source Screening

Table S3. Nickel-Source screening for the nickel catalyzed hydroacylation of *S*-phenyl thioesters with acrylates mediated by mechanochemistry.

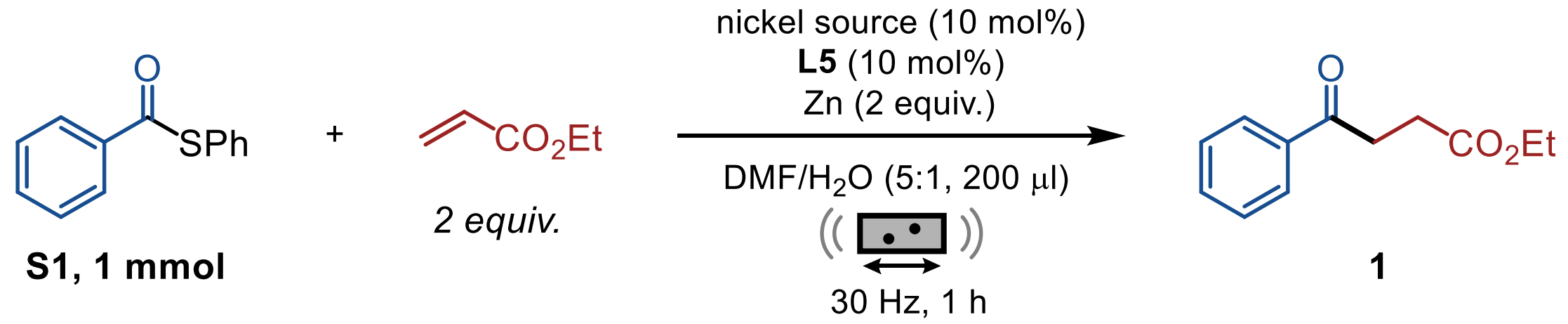

Entry	Nickel source	Yield (%)[a]	Conversion S1 (%)[a]
1	$NiCl_2(H_2O)_6$	82	100
2	$NiCl_2$(dme)	83	100
3	$NiBr_2$(dme)	81	100
4	$Ni(acac)_2$	73	99
5	$Ni(OAc)_2(H_2O)_4$	65	96
6	–	n. d.	8

Reaction conditions: **S1** (1 mmol, 1.0 equiv.), ethyl acrylate (2.0 equiv.), nickel source (10 mol%), **L5** (10 mol%), Zn (2 equiv.), DMF/H_2O (5:1, 200 µL), SS milling jar (10 mL), SS milling balls (2 × 4 g), MM (30 Hz, 1 h). [a]Determined by GC-FID using *n*-pentadecane as an internal standard.

3.4 Influence of Cp_2TiCl_2

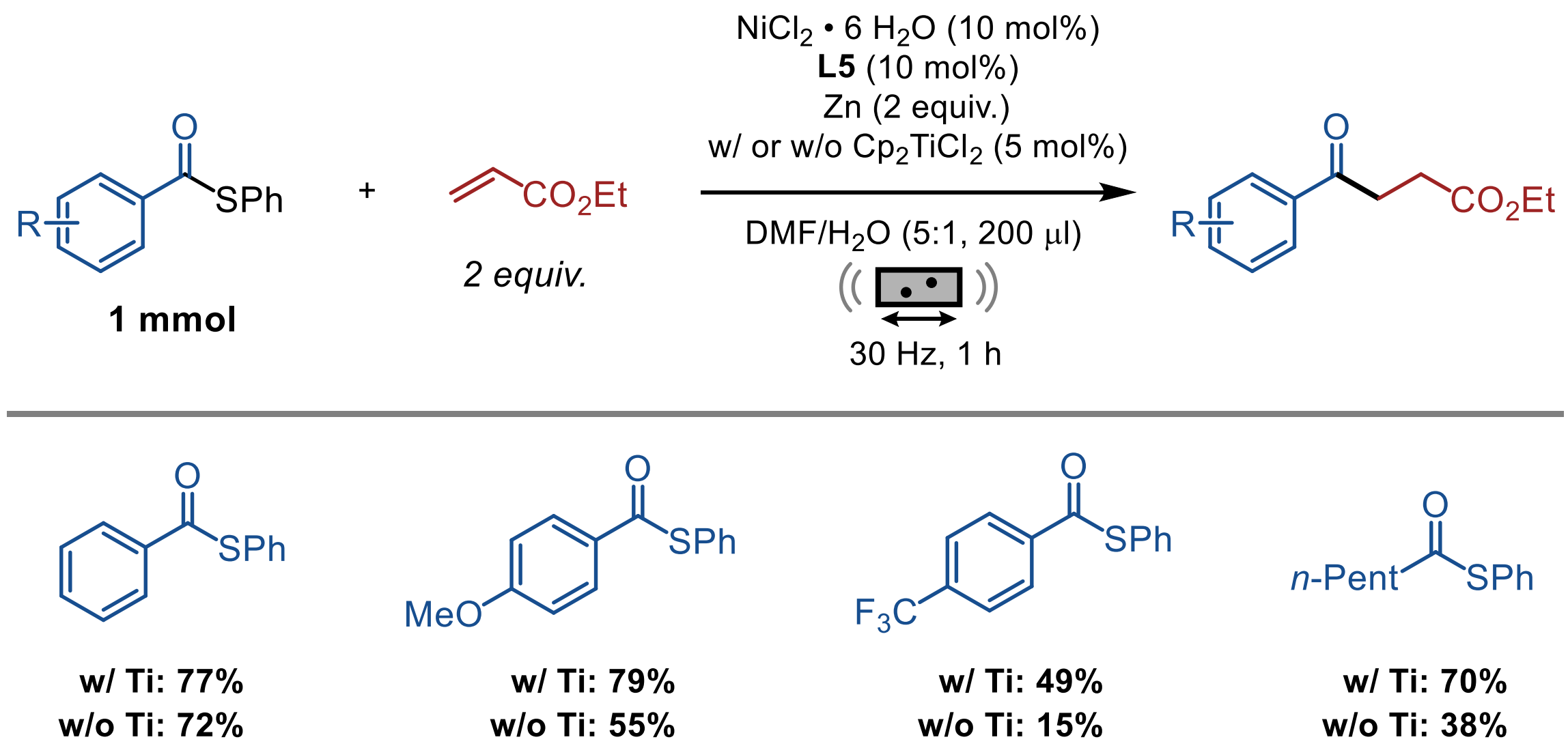

Reaction conditions: Thioester (1 mmol, 1.0 equiv.), ethyl acrylate (2.0 equiv.), $NiCl_2(H_2O)_6$ (10 mol%), **L5** (10 mol%), zinc (2 equiv.), Cp_2TiCl_2 (0 or 5 mol%), DMF/H_2O (5:1, 200 µL), SS milling jar (10 mL), SS milling balls (2 × 4 g), MM (30 Hz, 1 h). Isolated yields.

3.5 Reductant Screening

Table S4. Reductant screening for the nickel catalyzed hydroacylation of *S*-phenyl thioesters with acrylates mediated by mechanochemistry.

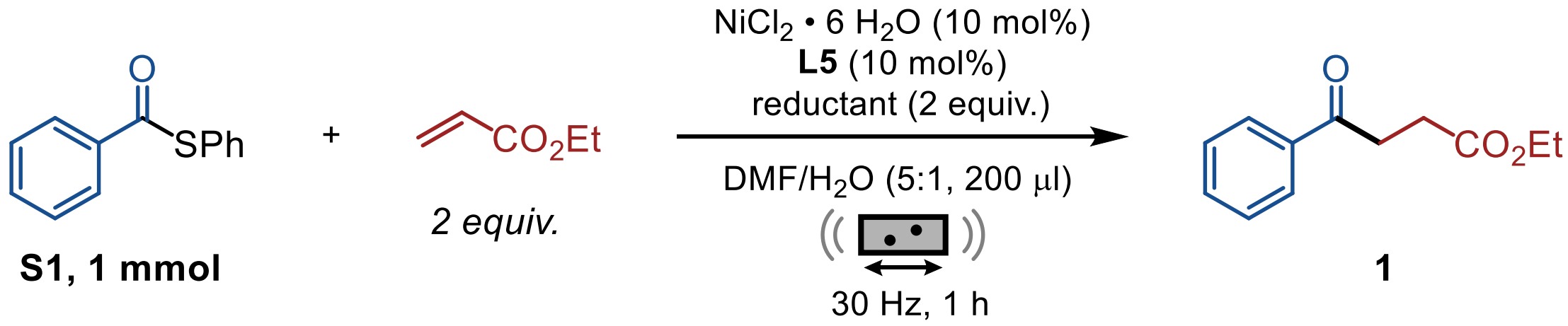

Entry	Reductant	Yield (%)[a]	Conversion S1 (%)[a]
1	Zn	82	100
2	Mn	3	79
3	Tetra(dimethylamino)ethylene	n. d.	15

Reaction conditions: **S1** (1 mmol, 1.0 equiv.), ethyl acrylate (2.0 equiv.), $NiCl_2(H_2O)_6$ (10 mol%), **L5** (10 mol%), reductant (2 equiv.), DMF/H_2O (5:1, 200 µL), SS milling jar (10 mL), SS milling balls (2 × 4 g), MM (30 Hz, 1 h). [a]Determined by GC-FID using *n*-pentadecane as an internal standard.

3.6 Additive Screening

Table S5. Additive screening for the nickel catalyzed hydroacylation of *S*-phenyl thioesters with acrylates mediated by mechanochemistry.

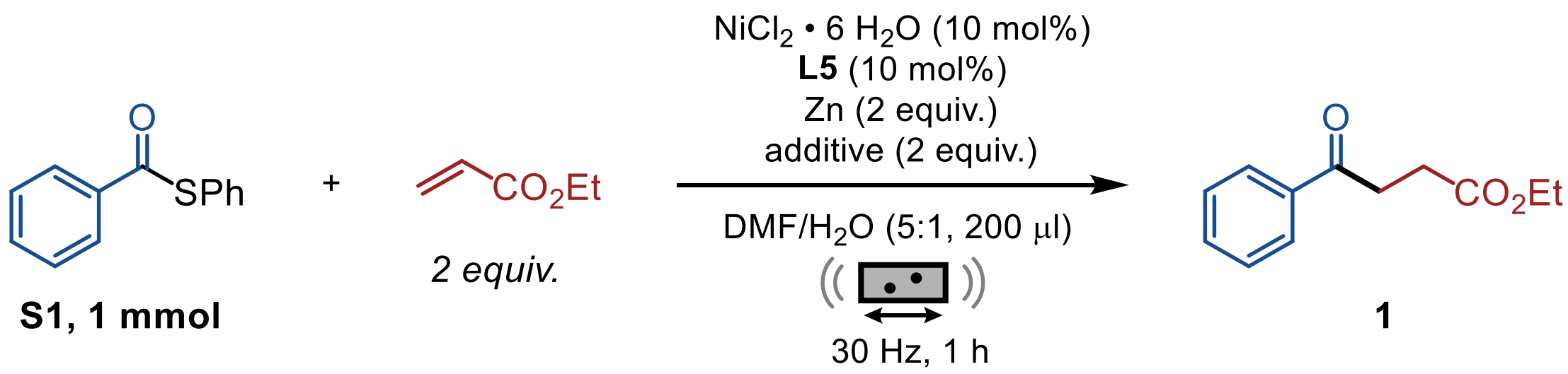

Entry	Additive	Yield (%)[a]	Conversion S1 (%)[a]
1	Pyridine	67	99
2	$Ph_3P=O$	67	100
3	NaCl	56	99
4	γ-terpinene	35	76
5	Et_2O-BF_3	70	92

Reaction conditions: **S1** (1 mmol, 1.0 equiv.), ethyl acrylate (2.0 equiv.), $NiCl_2(H_2O)_6$ (10 mol%), **L5** (10 mol%), Zn (2 equiv.), DMF/H_2O (5:1, 200 µL), additive (2 equiv.), SS milling jar (10 mL), SS milling balls (2 × 4 g), MM (30 Hz, 1 h). [a]Determined by GC-FID using *n*-pentadecane as an internal standard.

3.7 Solvent Screening for Deuteroacylation

Table S3. LAG additive screening for the nickel catalyzed deuteroacylation of *S*-phenyl thioesters with acrylates mediated by mechanochemistry.

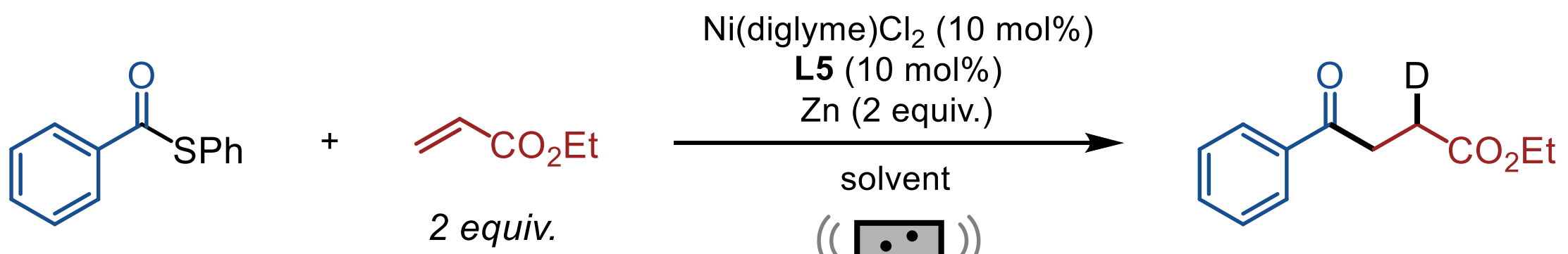

S1, 1 mmol **1**

Entry	LAG	Yield (%)[a]	Deuteration (%)[b]
1	DMF-d$_7$/H_2O (5:1, 200 µL)	78	< 1
2	DMF/D_2O (5:1, 200 µL)	70	81
3	DMF/D_2O (3:1, 200 µL)	72	85
4	DMF/D_2O (3:1, 400 µL)	80	99

Reaction conditions: **S1** (1 mmol, 1.0 equiv.), ethyl acrylate (2.0 equiv.), Ni(diglyme)Cl_2 (10 mol%), **L5** (10 mol%), Zn (2 equiv.), solvent, SS milling jar (10 mL), SS milling balls (2 × 4 g), MM (30 Hz, 1 h). [a] Isolated yield. [b] Determined by ^{1}H NMR spectroscopy.

3.8 Reaction under anhydrous conditions

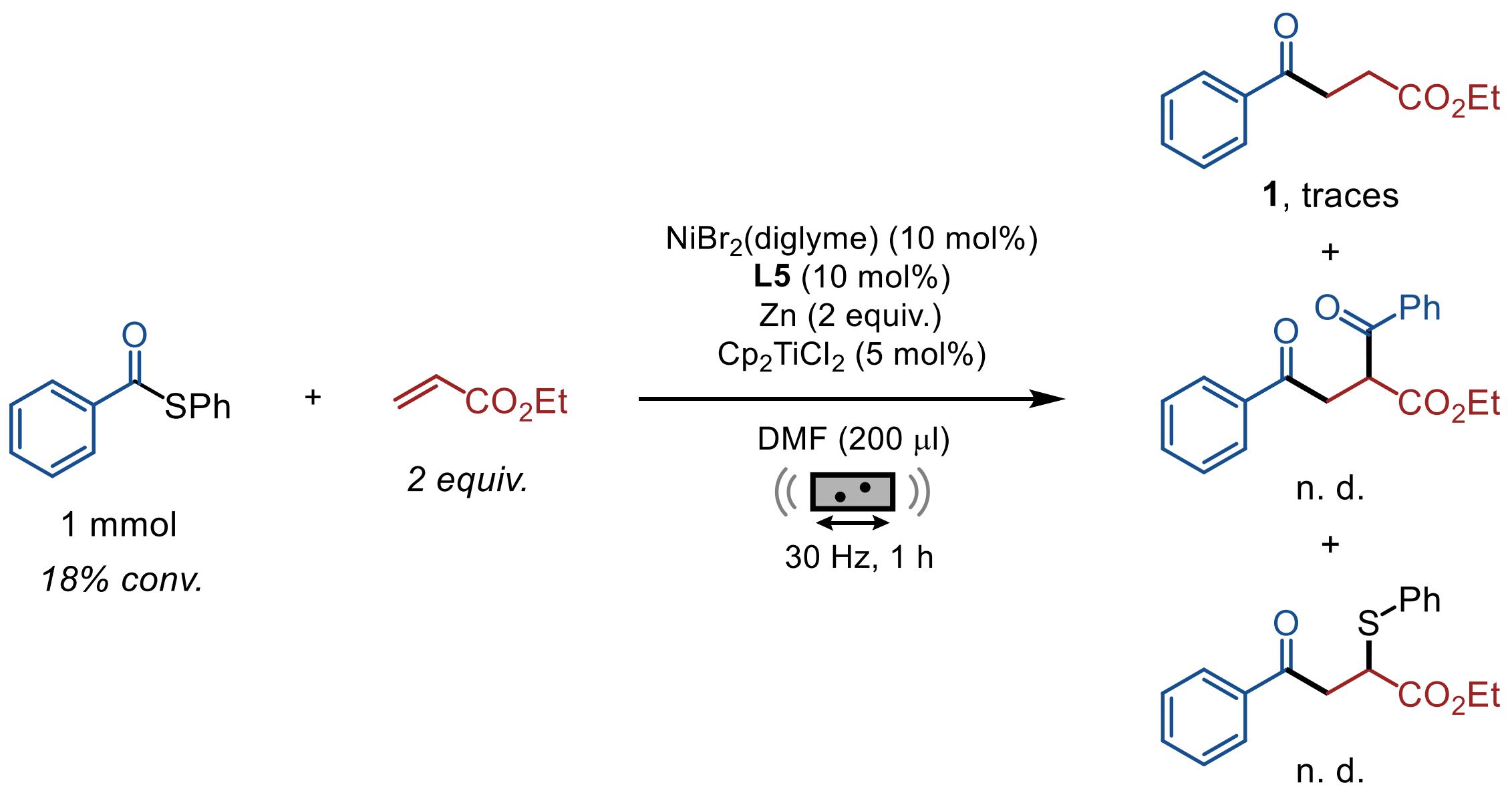

Results were analyzed by GC-MS.

4. Mechanistic Studies

4.1 Kinetic Isotope Studies

Note: To prevent falsification of the data by varying concentrations, competition experiments have to be conducted at low conversions. Thus, the reaction time was reduced to 10 min, ensuring conversion of 10–15%.

Primary kinetic isotope effect (pKIE)

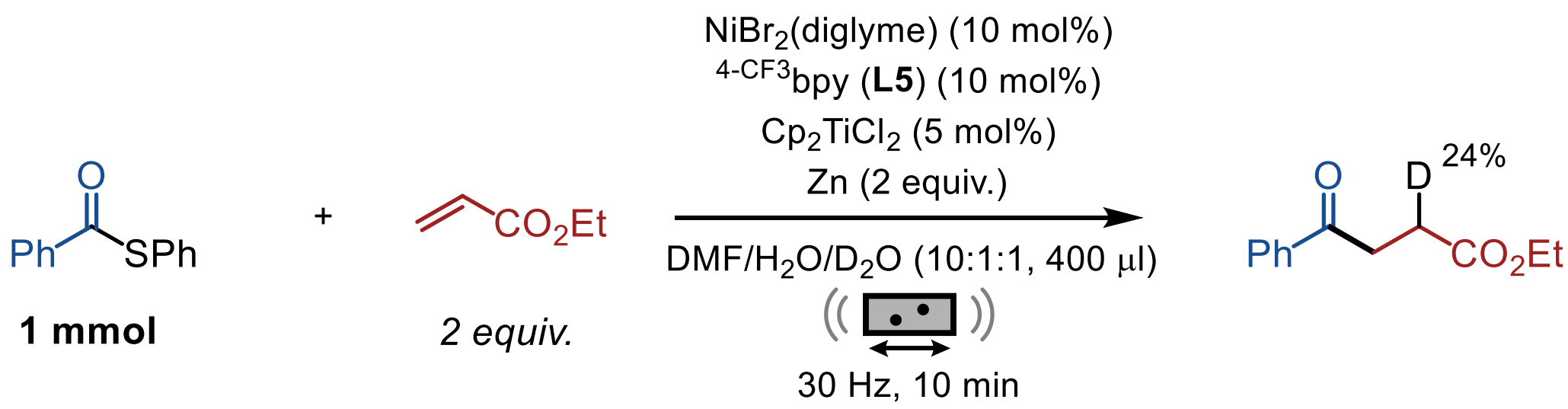

Scheme S1. Primary kinetic isotope effect experiment.

A 10 mL stainless steel milling jar was charged with two stainless steel balls (⌀ = 10 mm, m = 4 g), $NiBr_2$(diglyme) (35.2 mg, 0.1 mmol, 10 mol%), 4,4'-bis(trifluoromethyl)-2,2'-bipyridine (**L5**, 29.2 mg, 0.1 mmol, 10 mol%), titanocene dichloride (12.5 mg, 0.05 mmol, 5 mol%), zinc (131 mg, 2.0 mmol, 2.0 equiv.), **S1** (1.0 mmol, 1.0 equiv.), ethyl acrylate (2.0 mmol, 2.0 equiv.), and a premixed solvent mixture of DMF/H_2O/D_2O (10:1:1 v/v/v, 400 µL). The milling jar was closed, placed in a MM 400 and shaken at 30 Hz for 10 min. Subsequently, the jar was opened and rinsed with EtOAc (10 mL). Volatiles were removed *in vacuo* and the residue was subjected to flash column chromatography (*n*-hexane/EtOAc) for purification. **1/1-D** (28 mg, 135 µmol, 14%) was received as colorless oil.

The deuterium incorporation was determined: 24% D (^{1}H NMR).

Calculation of KIE using Taylor approximation for small x (low conversion) ln(1–x) = –x – …

$$KIE = \frac{k_H}{k_D} = \frac{\ln(1-n(P_H))}{\ln(1-n(P_D))} \approx \frac{-n(P_H)}{-n(P_D)} = \frac{0.7568\, n(P_D)}{0.2432\, n(P_D)} = 3.1$$

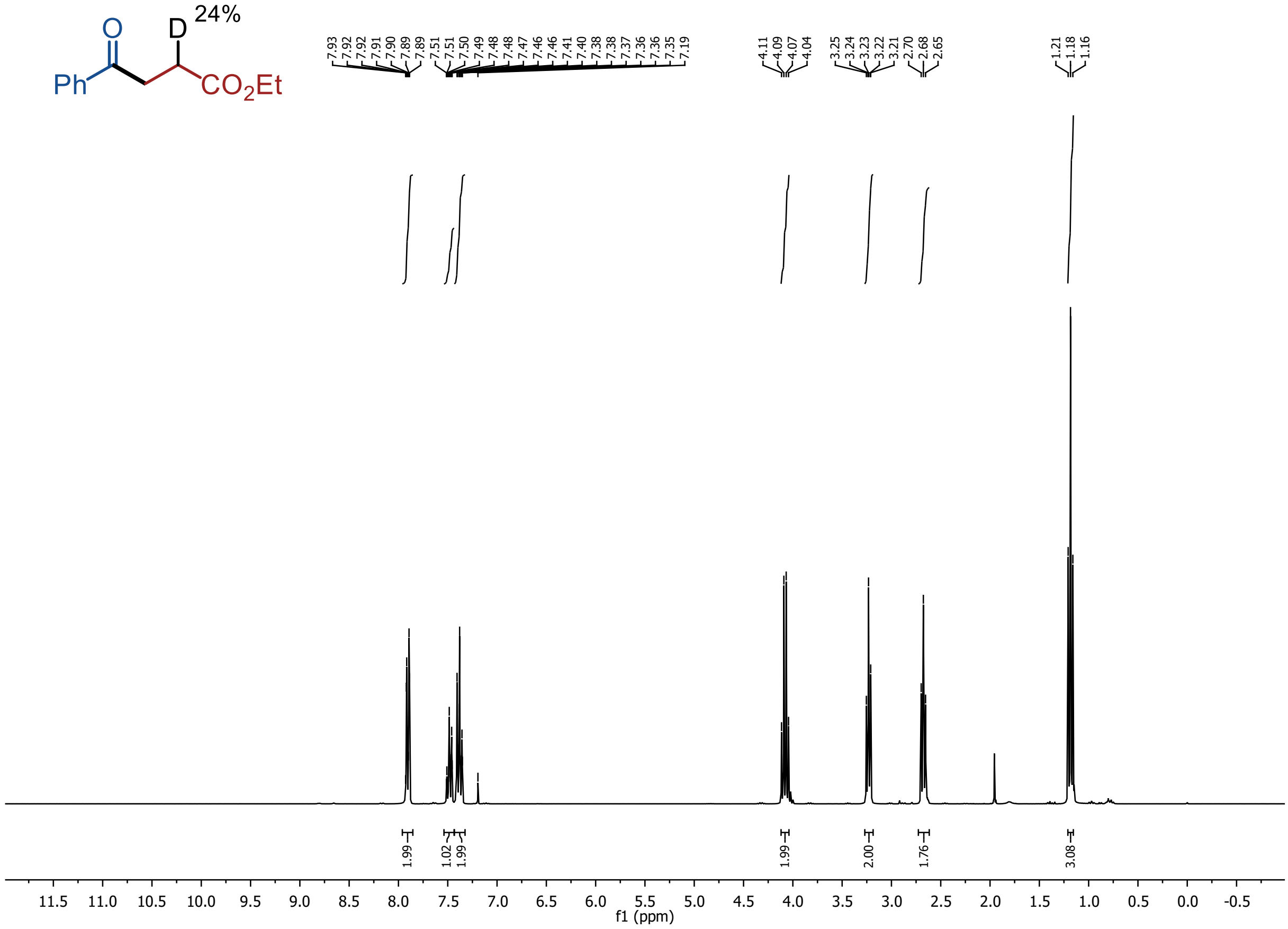

Figure S1. ^{1}H NMR spectrum (300 MHz, $CDCl_3$) of the pKIE experiment.

Secondary kinetic isotope effect (sKIE)

sKIE at α- and β-position

Note: Since methyl acrylate-d3 (**S28b**) was not received with 100% D, the amount of **S28b** was adjusted volumetrically, so the ratio of protonated/deuterated acrylate was 1:1.

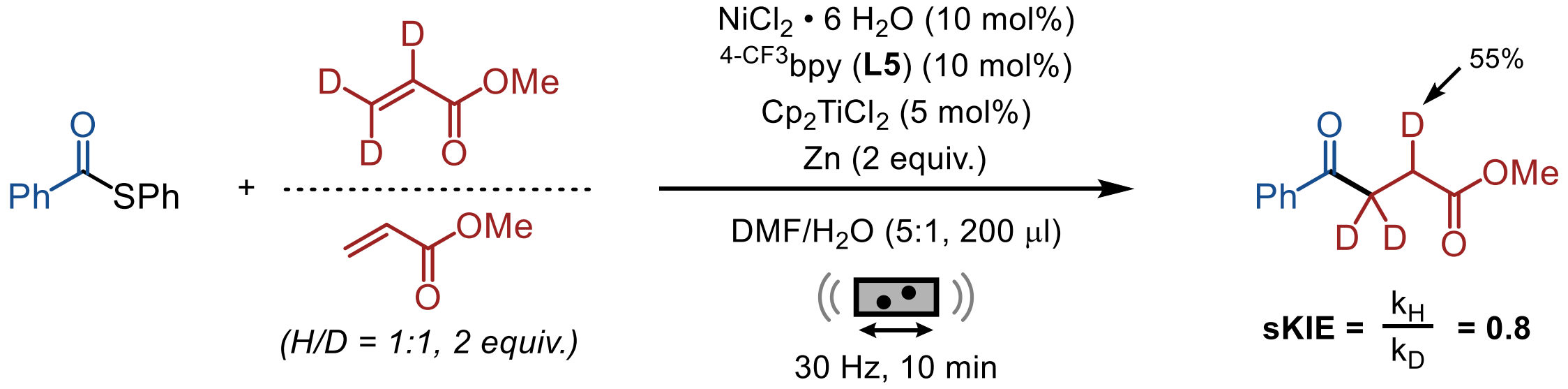

Scheme S2. Secondary kinetic isotope effect experiment at *α*- and *β*-position.

A 10 mL stainless steel milling jar was charged with two stainless steel balls (⌀ = 10 mm, m = 4 g), $NiCl_2 \cdot 6H_2O$ (23.8 mg, 0.1 mmol, 10 mol%), 4,4'-bis(trifluoromethyl)-2,2'-bipyridine (**L5**, 29.2 mg, 0.1 mmol, 10 mol%), titanocene dichloride (12.5 mg, 0.05 mmol, 5 mol%), zinc (131 mg, 2.0 mmol, 2.0 equiv.), **S1** (1.0 mmol, 1.0 equiv.), methyl acrylate-d3 (**S28b**) (1.0 mmol, 1.0 equiv.), methyl acrylate (1.0 mmol, 1.0 equiv.), and a premixed solvent mixture of DMF/H_2O (5:1 v/v, 200 µL). The milling jar was closed, placed in a MM 400 and shaken at 30 Hz for 10 min. Subsequently, the jar was opened and rinsed with EtOAc (10 mL). Volatiles were removed *in vacuo* and the residue was subjected to flash column chromatography (*n*-hexane/EtOAc) for purification. **28/28-D3** (30 mg, 154 µmol, 15%) was received as colorless oil.

The deuterium incorporation was determined: 55% D (^{1}H NMR).

This equals an sKIE = 0.8.

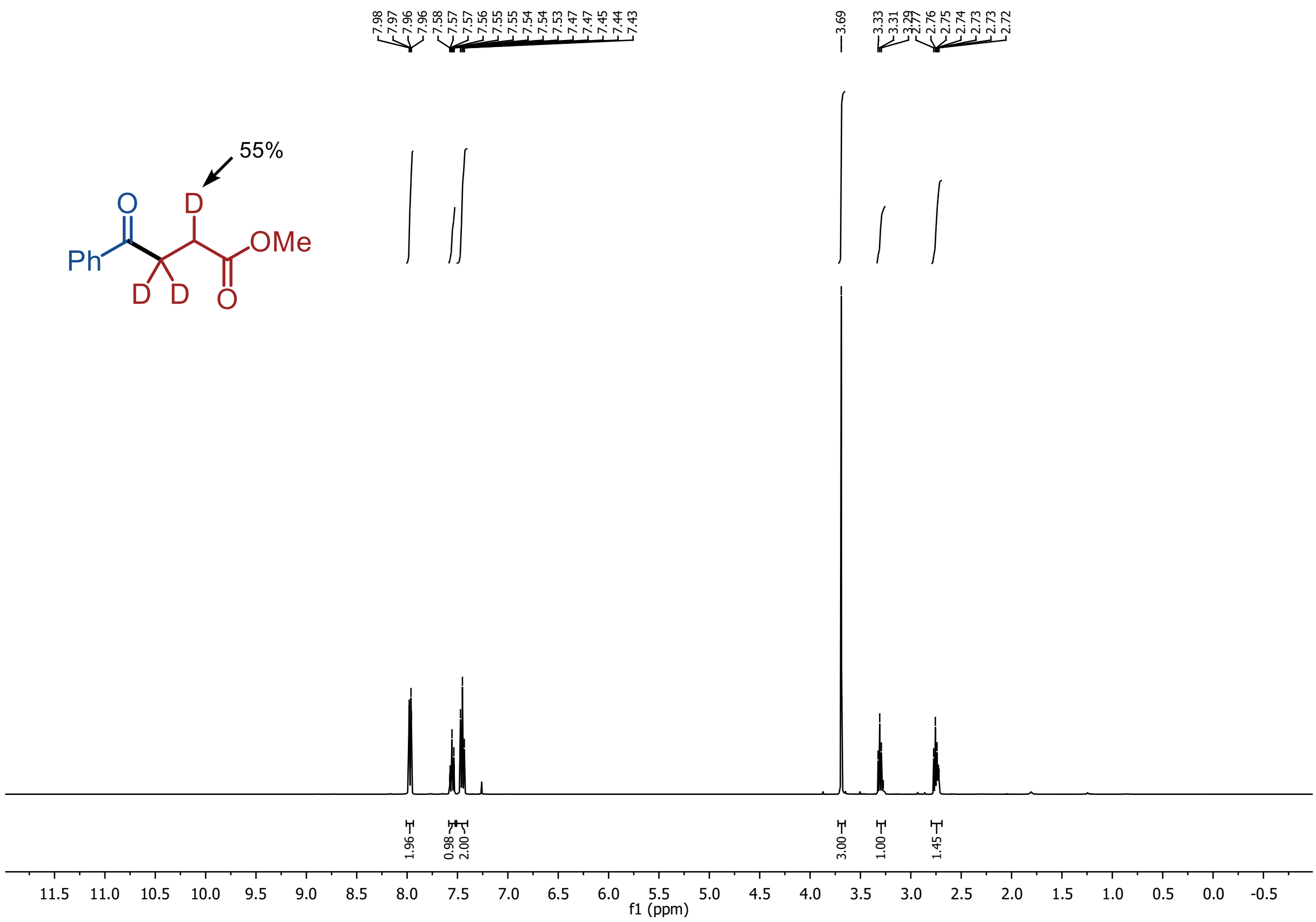

Figure S2. ^{1}H NMR spectrum (400 MHz, $CDCl_3$) of the sKIE experiment at *α*- and *β*-position.

sKIE at α *-position*

Note: Since methyl acrylate-d$_4$ (**S28a**) was not received with 100% D, the amount of **S28a** was adjusted volumetrically, so the ratio of protonated/deuterated acrylate was 1:1.

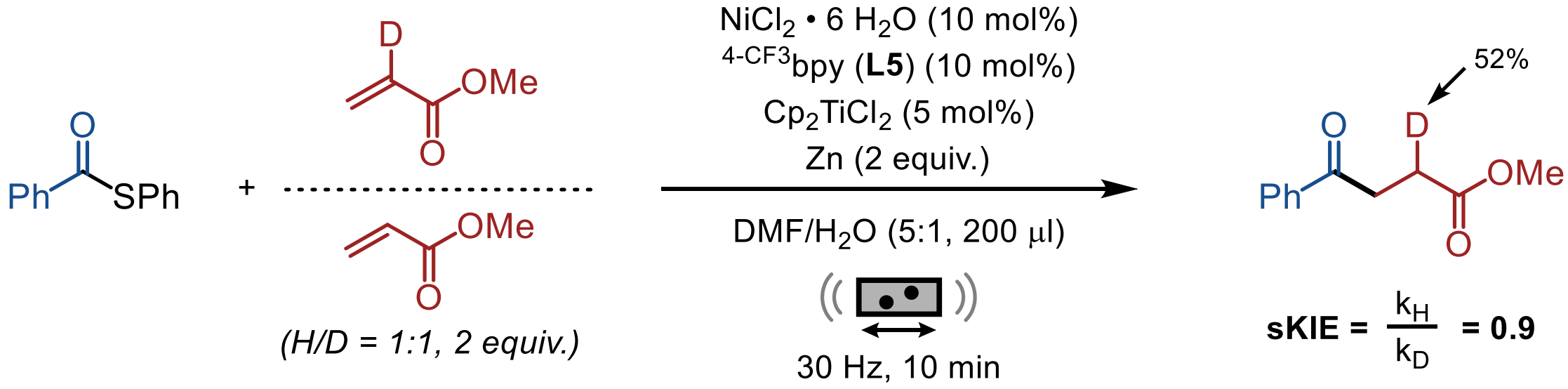

Scheme S3. Secondary kinetic isotope effect experiment at *α*-position.

A 10 mL stainless steel milling jar was charged with two stainless steel balls (⌀ = 10 mm, m = 4 g), $NiCl_2 \cdot 6H_2O$ (23.8 mg, 0.1 mmol, 10 mol%), 4,4'-bis(trifluoromethyl)-2,2'-bipyridine (**L5**, 29.2 mg, 0.1 mmol, 10 mol%), titanocene dichloride (12.5 mg, 0.05 mmol, 5 mol%), zinc (131 mg, 2.0 mmol, 2.0 equiv.), **S1** (1.0 mmol, 1.0 equiv.), methyl acrylate-d$_4$ (**S28a**)

(1.0 mmol, 1.0 equiv.), methyl acrylate (1.0 mmol, 1.0 equiv.), and a premixed solvent mixture of DMF/H_2O (5:1 v/v, 200 µL). The milling jar was closed, placed in a MM 400 and shaken at 30 Hz for 10 min. Subsequently, the jar was opened and rinsed with EtOAc (10 mL). Volatiles were removed *in vacuo* and the residue was subjected to flash column chromatography (*n*-hexane/EtOAc) for purification. **28**/**28-D$_4$** (27 mg, 139 µmol, 14%) was received as colorless oil.

The deuterium incorporation was determined: 52% D at *α*-position (^{1}H NMR).

This equals an sKIE = 0.9.

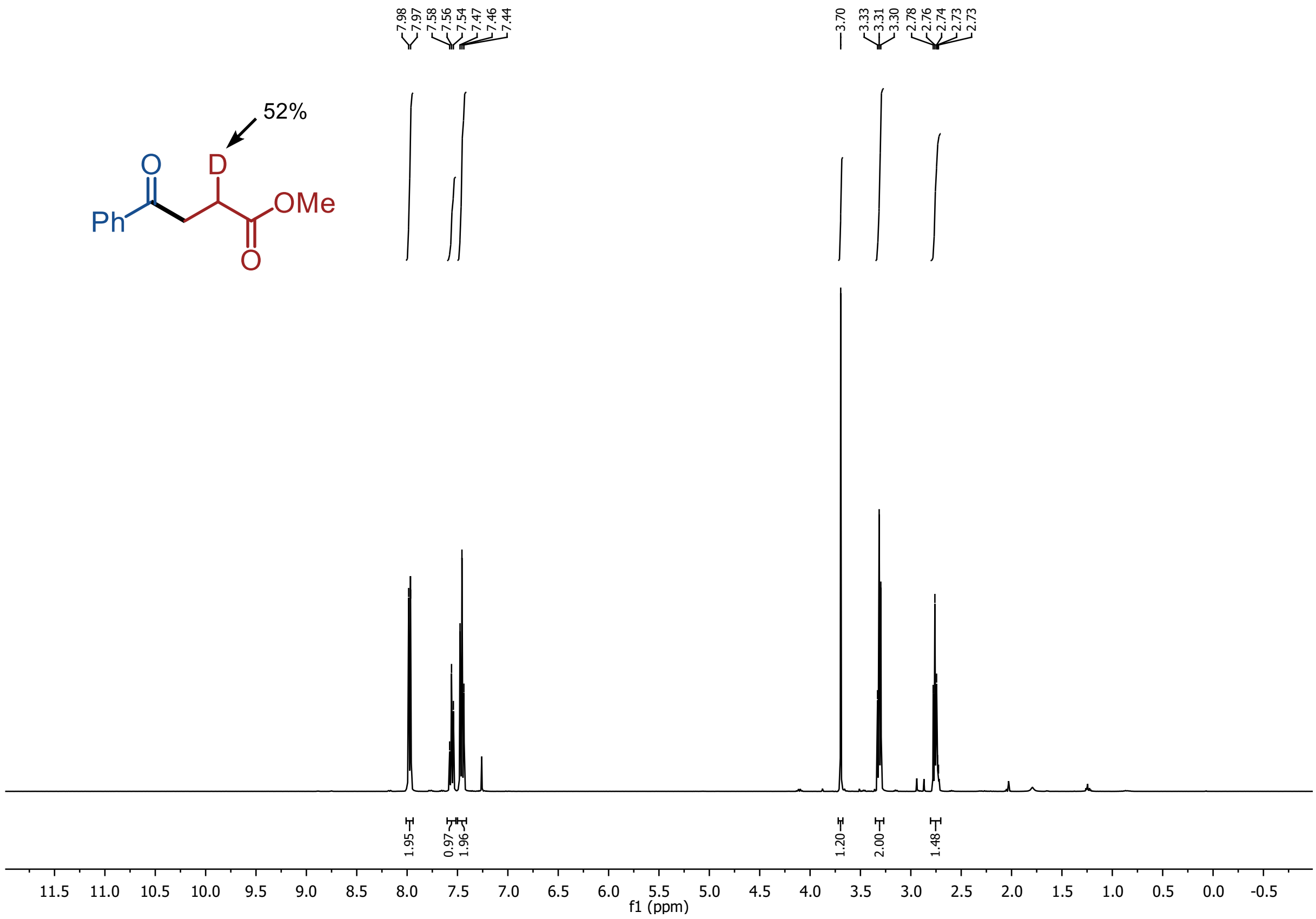

Figure S3. ^{1}H NMR spectrum (400 MHz, $CDCl_3$) of the sKIE experiment at *α*-position.

4.2 Radical Scavengers

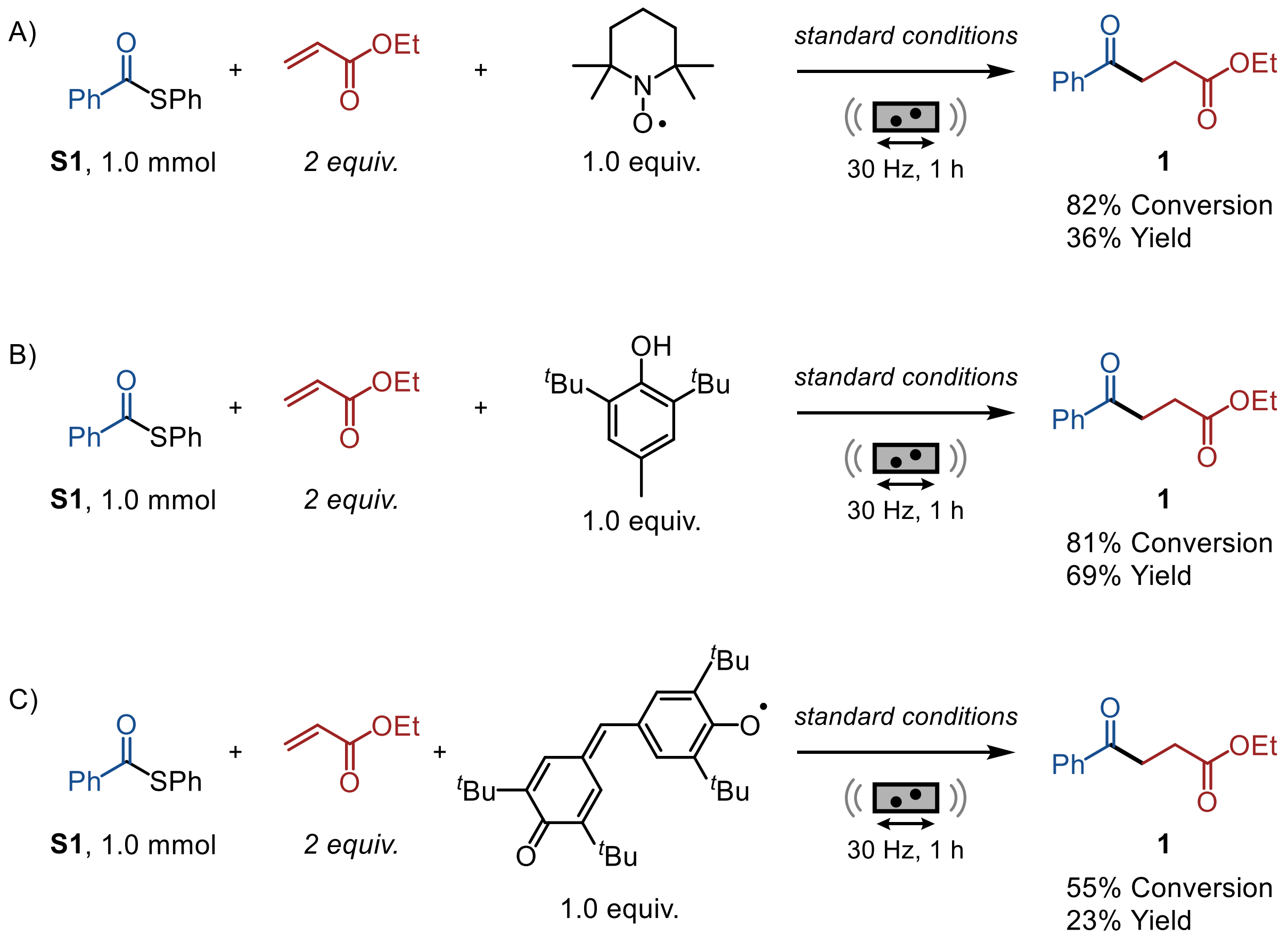

Scheme S4. Radical scavenger experiments. Yields and conversions determined by GC-FID using pentadecane as internal standard.

A 10 mL stainless steel milling jar was charged with two stainless steel balls (⌀ = 10 mm, m = 4 g), $NiCl_2{\cdot}6H_2O$ (23.8 mg, 0.1 mmol, 10 mol%), 4,4'-bis(trifluoromethyl)-2,2'-bipyridine (**L5**, 29.2 mg, 0.1 mmol, 10 mol%), titanocene dichloride (12.5 mg, 0.05 mmol, 5 mol%), zinc (131 mg, 2.0 mmol, 2.0 equiv.), **S1** (1.0 mmol, 1.0 equiv.), ethyl acrylate (2.0 mmol, 2.0 equiv.), the respective radical scavenger (1.0 mmol, 1.0 equiv.) and a premixed solvent mixture of DMF/H_2O (5:1 v/v, 200 µL). The milling jar was closed, placed in a MM 400 and shaken at 30 Hz for 1 h. Subsequently, the jar was opened, internal standard and EtOAc (3 mL) were added. A sample was filtered through celite and analyzed by GC-FID.

4.3 Hammett Studies

Note: To prevent falsification of the data by varying concentrations, competition experiments have to be conducted at low conversions. Thus, the reaction time was reduced to 10 min, ensuring conversion of 10–20%.

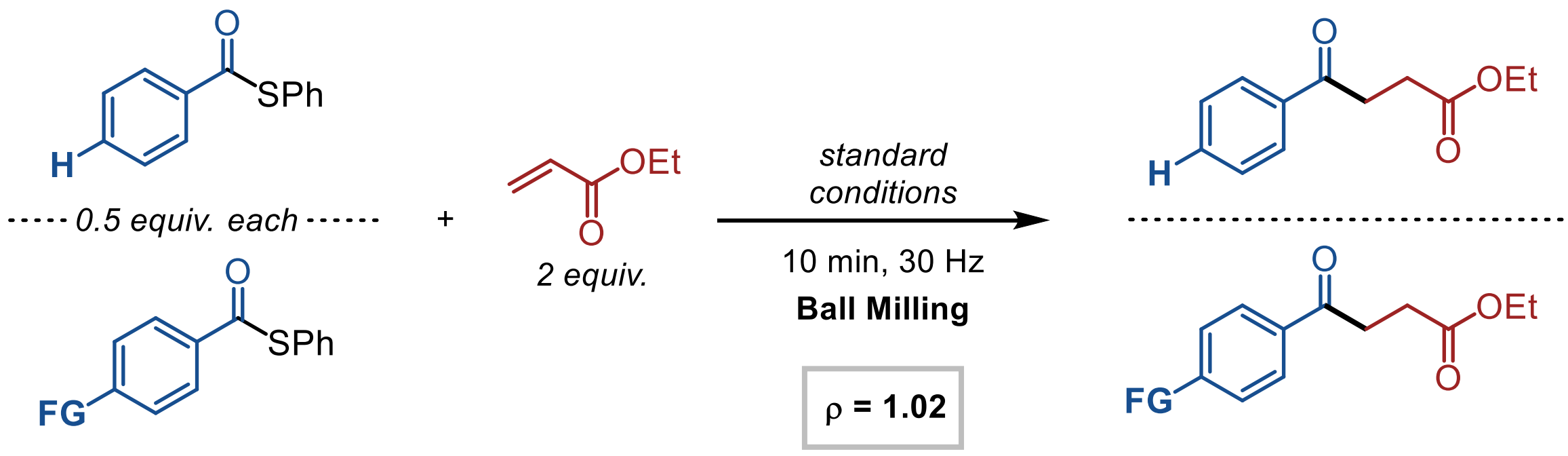

Scheme S5. Hammett analysis.

A 10 mL stainless steel milling jar was charged with two stainless steel balls (⌀ = 10 mm, m = 4 g), $NiCl_2 \cdot 6H_2O$ (23.8 mg, 0.1 mmol, 10 mol%), 4,4'-bis(trifluoromethyl)-2,2'-bipyridine (**L5**, 29.2 mg, 0.1 mmol, 10 mol%), titanocene dichloride (12.5 mg, 0.05 mmol, 5 mol%), zinc (131 mg, 2.0 mmol, 2.0 equiv.), **S1** (0.5 mmol, 0.5 equiv.), the respective thioester (0.5 mmol, 0.5 equiv.), ethyl acrylate (2.0 mmol, 2.0 equiv.), and a premixed solvent mixture of DMF/H_2O (5:1 v/v, 200 µL). The milling jar was closed, placed in a MM 400 and shaken at 30 Hz for 10 min. Subsequently, the jar was opened and rinsed with EtOAc (10 mL). Volatiles were removed *in vacuo* and the residue was subjected to flash column chromatography (*n*-hexane/EtOAc) for purification. The ratio of products was determined by ^{1}H NMR spectroscopy.

Table S1. Results of the Hammett analysis.

Entry	Functional Group FG	σ [a]	Yield (%)[b]	n(FG)/n(H)[c]	$\log(k_X/k_H)$
1	OMe	-0.27	17	0.47	-0.33
2	*tert*-butyl	-0.20	17	0.76	-0.12
3	H	0	–	1	0
4	F	0.06	16	1.67	0.22
5	Cl	0.23	19	2.60	0.42
6	CF_3	0.54	21	3.13	0.50

[a]σ-values obtained from literature.[4] [b]Isolated yield of the product mixture. [c]Ratio determined by ^{1}H NMR spectroscopy.

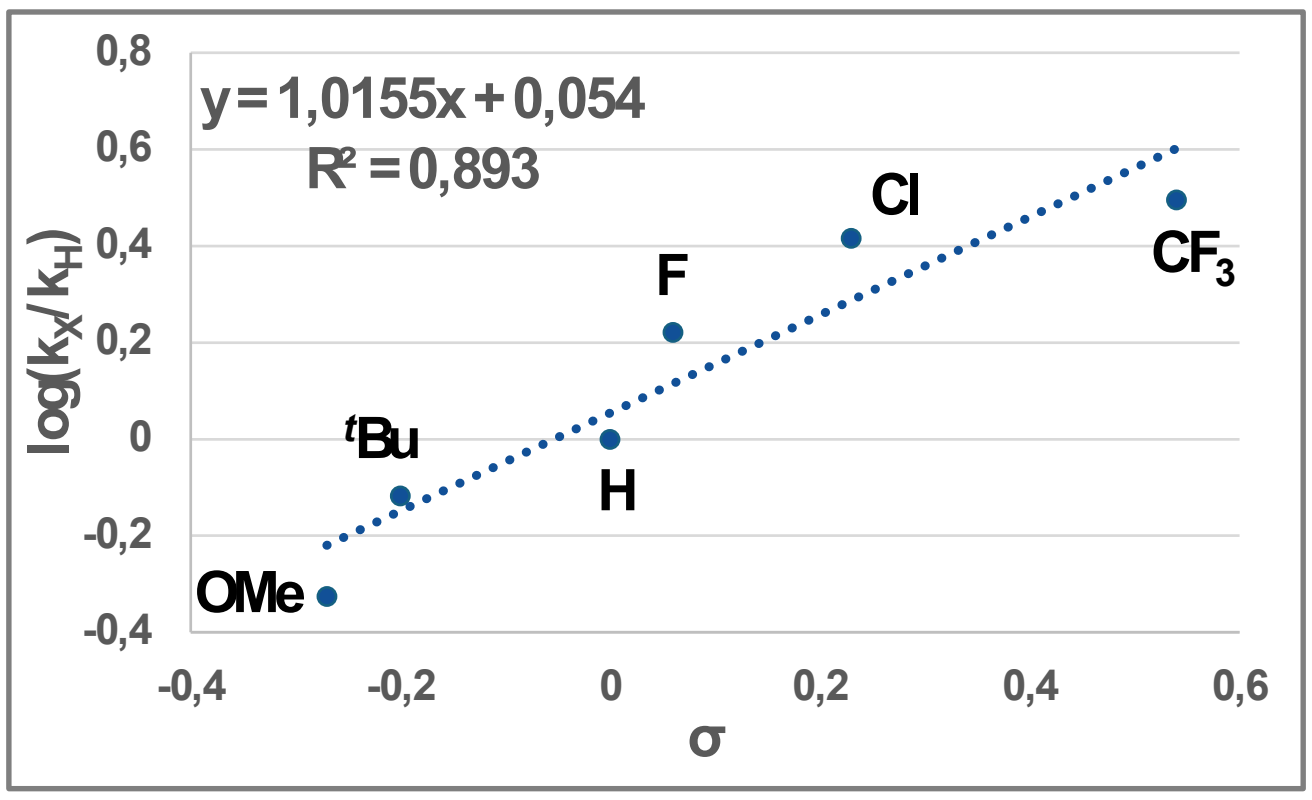

Figure S4. Results of the Hammett analysis.

4.4 Organometallic Intermediates

*Interaction of acrylate with (**L5**)Ni(cod)*

In an NMR tube, Ni(cod)$_2$ (10.0 mg, 38.6 µmol, 1 equiv.), and **L5** (11.3 mg, 38.6 µmol, 1 equiv.) were dissolved in THF-d$_5$ or DMF-d$_7$ and a reference spectrum was obtained. Ethyl acrylate was added in portions of 2 equiv. and after each portion, an NMR spectrum was obtained.

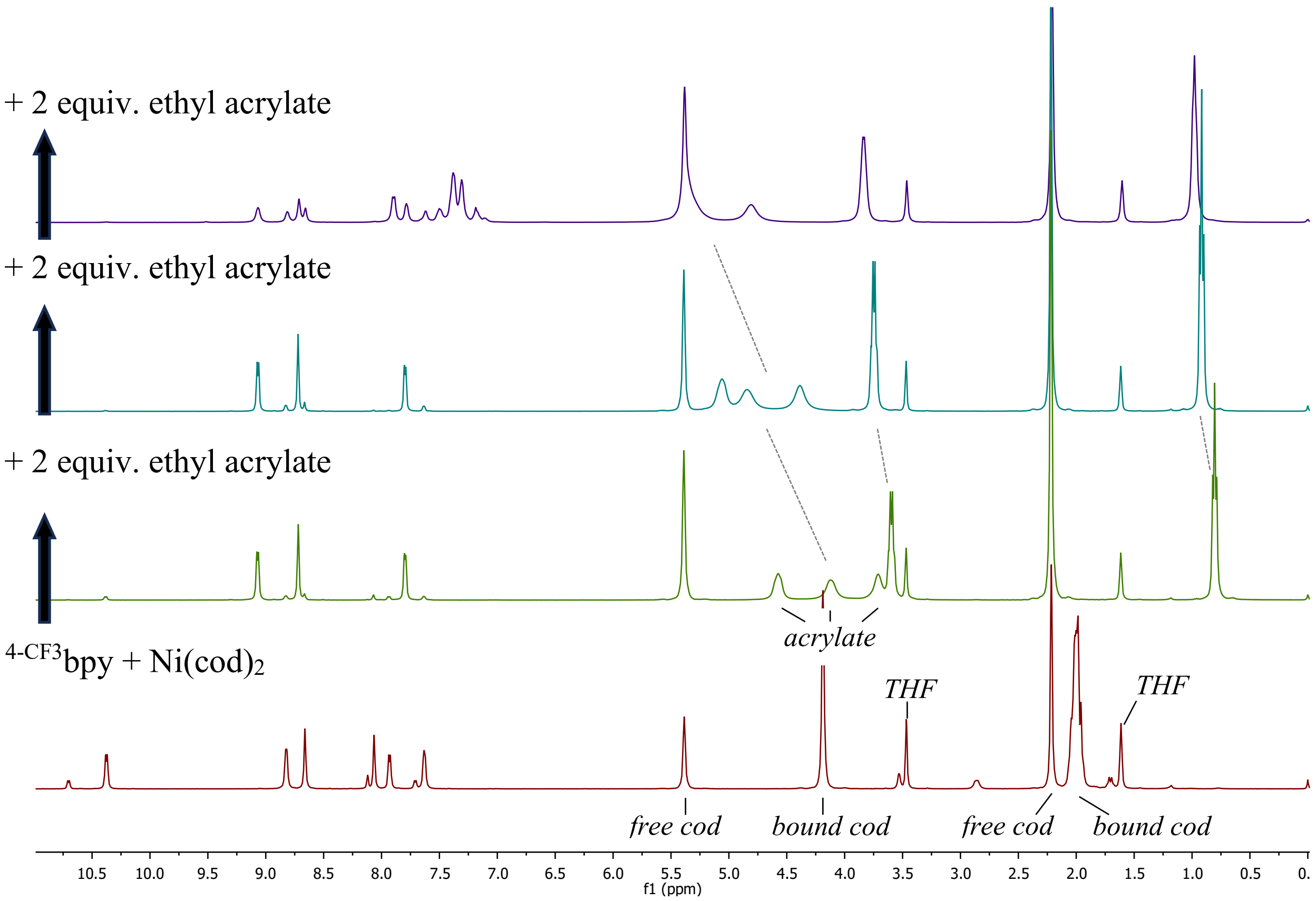

Figure S5. Addition of ethyl acrylate to (**L5**)Ni(cod).

After each addition step, the proton resonances of the acrylate moiety are shifted more downfield. This indicates a dynamic equilibrium between bound and free acrylate. The more acrylate is added, the more free acrylate is present, shifting the average signal. Similar behavior was observed in DMF-d$_7$, but the low solubility of Ni(cod)$_2$ in DMF-d$_7$ rendered the use of THF more convenient.

This equilibrium could be further examined by low-temperature NMR spectroscopy (Figure S6):

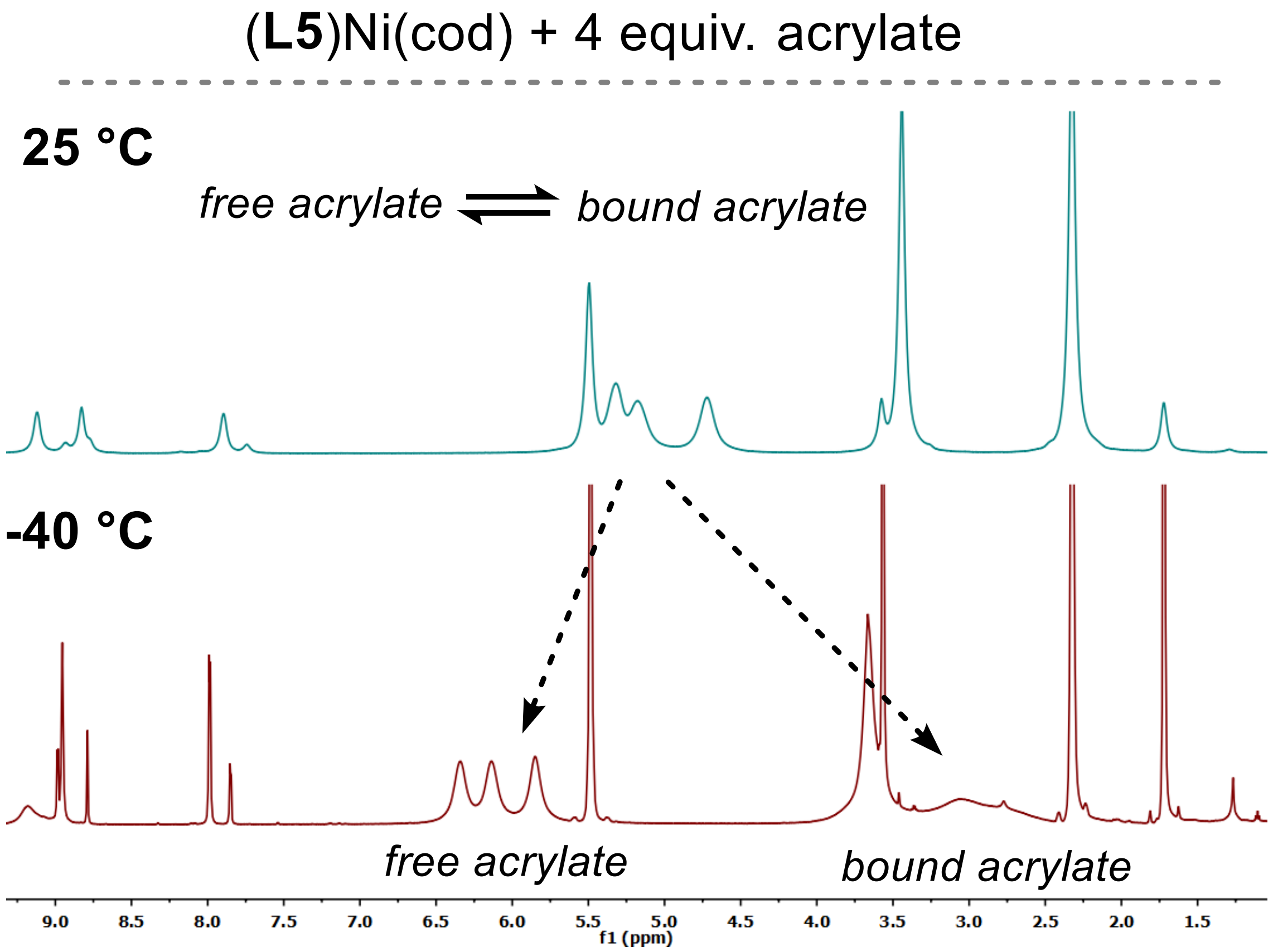

Figure S6. ^{1}H NMR spectra (700 MHz, THF-d$_8$) of a mixture of (**L5**)Ni(cod) with 4 equiv. methyl acrylate at 25 °C (blue line) and -40 °C (red line).

Oxidative addition of thioester to ***Ni0*** *and reactivity with water and zinc.*

Ni0 was synthesized independently (see chapter 5.1).

In an NMR tube, **Ni0** (5.0 mg, 1 equiv.) and *S*-phenyl benzothioate (1 equiv.) were dissolved in THF-d$_8$ (0.5 mL) and the reaction was monitored by NMR spectroscopy (Figure S7). From the spectrum after the addition was a strong downfield shift of the acrylate protons visible, indicating that no coordination of the alkene to nickel was present.

In an NMR tube, Ni(cod)$_2$ (10 mg, 38.6 µmol, 1 equiv.), **L5** (11.3 mg, 38.6 µmol, 1 equiv.) and *S*-phenyl benzothioate (1 equiv.) were dissolved in DMF-d$_7$ (0.5 mL) by ultrasonication for 30 min. Methyl acrylate (2 equiv.) was added and the reaction was monitored by NMR spectroscopy (Figure S8). Subsequently, water (2 equiv.) was added.

No reaction upon addition of water was observed, even after 1 d reaction time.

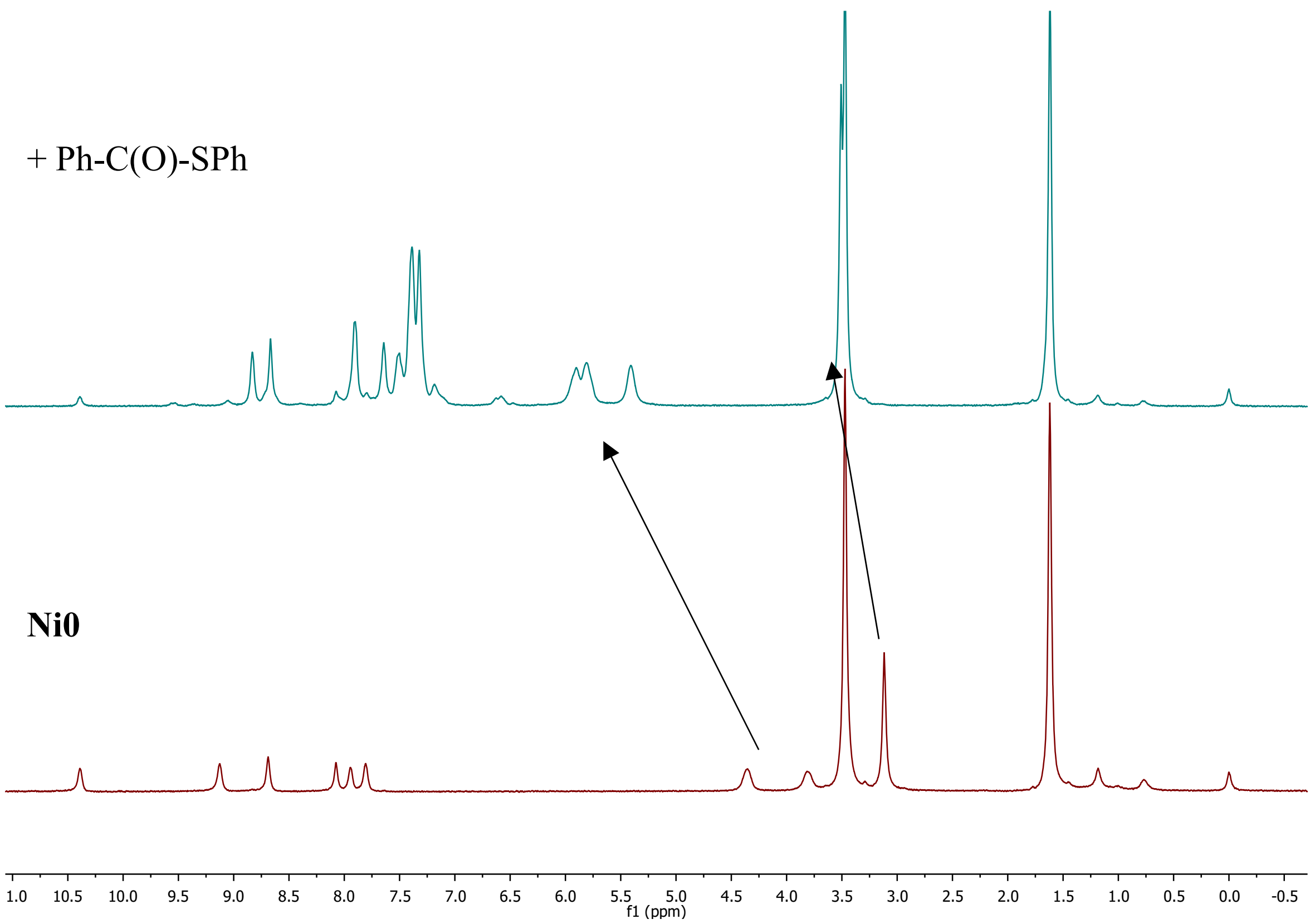

Figure S7. ^{1}H NMR spectra (400 MHz, THF-d_8) of the reaction of **Ni0** with *S*-phenyl benzothioate.

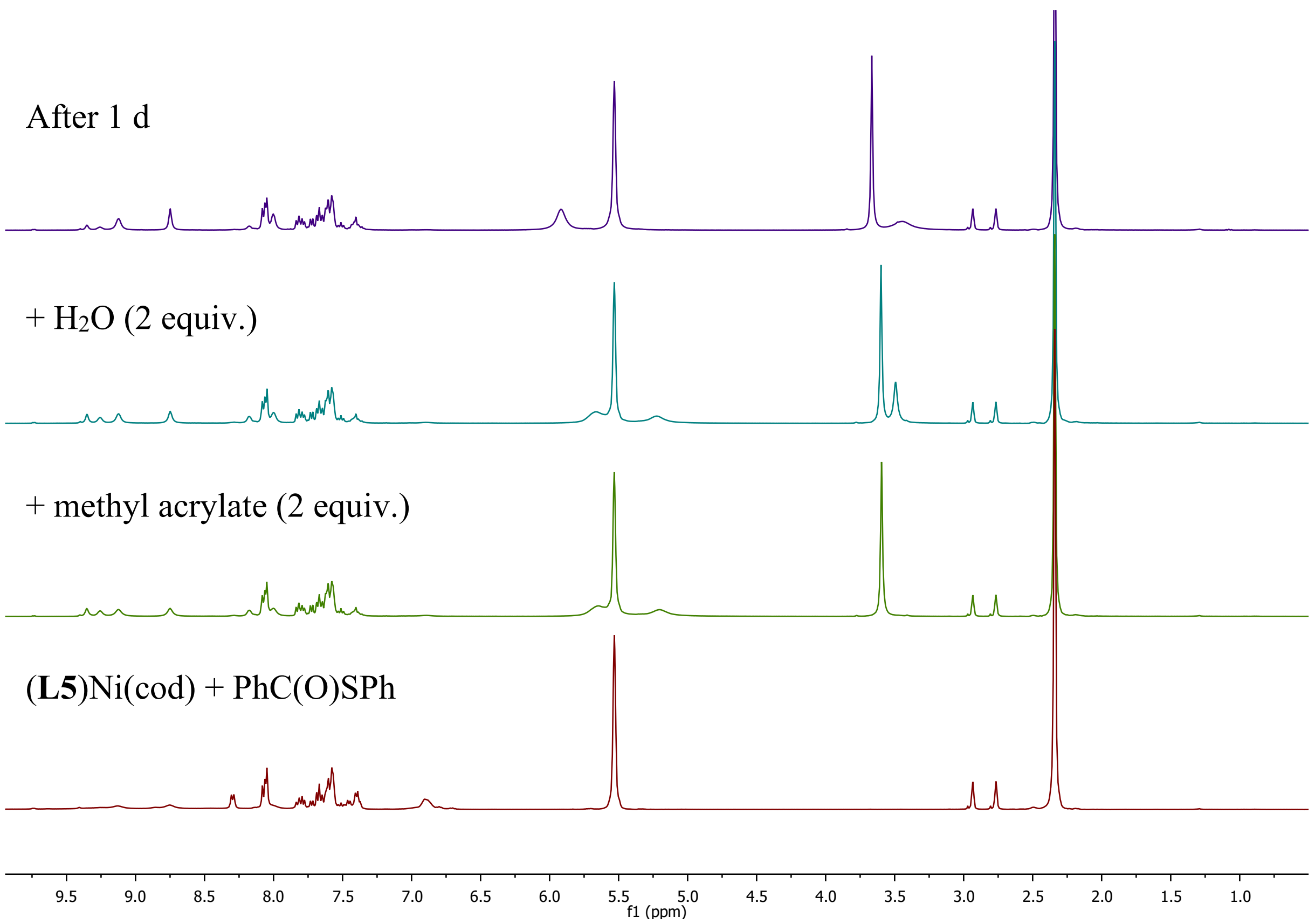

Figure S8. ^{1}H NMR spectra (400 MHz, DMF-d_7) of the reaction of the oxidative addition complex with acrylate and water.

4.5 Stoichiometric Coupling Experiments

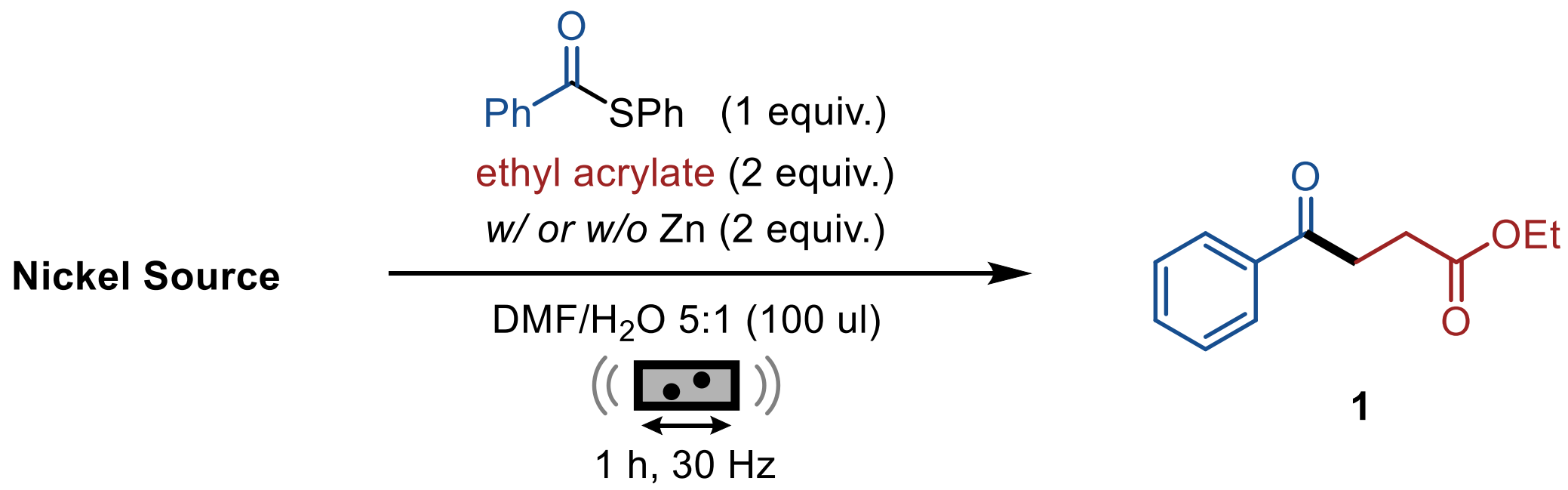

In a glovebox, a milling jar (SS, 10 mL) was subsequently filled with milling balls (SS, 2 × 4 g), the corresponding nickel source (50 µmol, 1 equiv.), ligand **L5** (if not preligated, 1 equiv.), thioester (1 equiv.), zinc (2 equiv.), ethyl acrylate (2 equiv.) and DMF/H_2O (premixed, 5:1, 100 µl). The jar was closed and subjected to ball-milling at 30 Hz for 1 h. The jar was opened and a sample was analyzed by GC-FID using pentadecane as internal standard. The following results were obtained:

Nickel source	Yield of 1
$Ni(cod)_2$ + **L5**	n. d.
**Ni0**	n. d.
**Ni2**	trace
$NiCl_2(H_2O)_6$ + **L5**	*with Zn:* 5%<br>*w/o Zn*: n. d.
(**L5**)Ni(I)-Cl	*with Zn:* 41%<br>*w/o Zn*: n. d.

5. Analytical Data

5.1 Synthesis of Nickel Complexes

Ni0

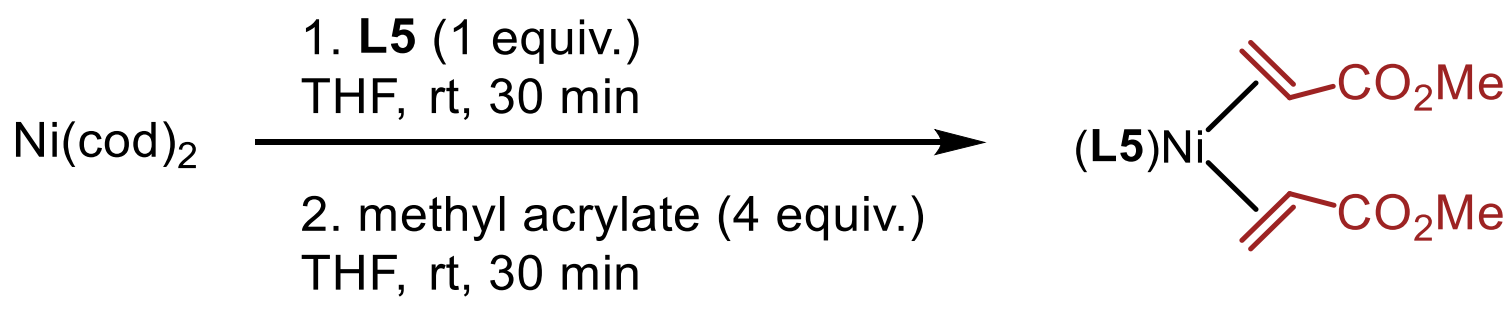

Ni0, 35%

In a Schlenk tube, $Ni(cod)_2$ (100 mg, 386 µmol, 1 equiv.) and 4,4'-bis(trifluoromethyl)-2,2'-bipyridine (**L5**, 113 mg, 386 µmol, 1 equiv.) were dissolved in dry THF (5 mL) and stirred for 30 min, resulting in a deep blue solution. Methyl acrylate (133 mg, 1.54 mmol, 4 equiv.) was added and the mixture was stirred at rt for another 30 min until a brown precipitate formed. The mixture was filtered and the residue washed with *n*-hexane (3 × 5 mL) and dried under vacuum. **Ni0** (74 mg, 134 µmol, 35%) was received as a brown solid.

NMR analysis revealed that two species with differing amounts of acrylate ligands were present.

Broadening of signals prevented analysis of *J*-coupling.

^{1}H NMR (600 MHz, THF-d_8, δ): 10.50 (s, 1.6H), 9.23 (s, 2H), 8.80 (s, 2H), 8.19 (s, 1.6H), 8.05 (s, 1.6H), 7.92 (s, 2H), 4.49 (s, 3.5H), 3.95 (s, 3.5H), 3.49 (s, 3.5H, *under residual solvent peak*) 3.23 (s, 9H).

^{13}C{^{1}H} NMR (151 MHz, THF-d_8, δ): 169.3 (br), 152.7, 150.9, 141.3, 137.0 (q, *J* = 34.5 Hz), 127.6 (q, *J* = 271.1 Hz), 124.0 (q, *J* = 271.7 Hz), 123.6 (q, *J* = 4.6 Hz), 122.9 (q, *J* = 33.2 Hz), 121.9, 118.9, 118.1, 50.1.

^{19}F NMR (565 MHz, THF-d_8, δ): -65.2, -66.3.

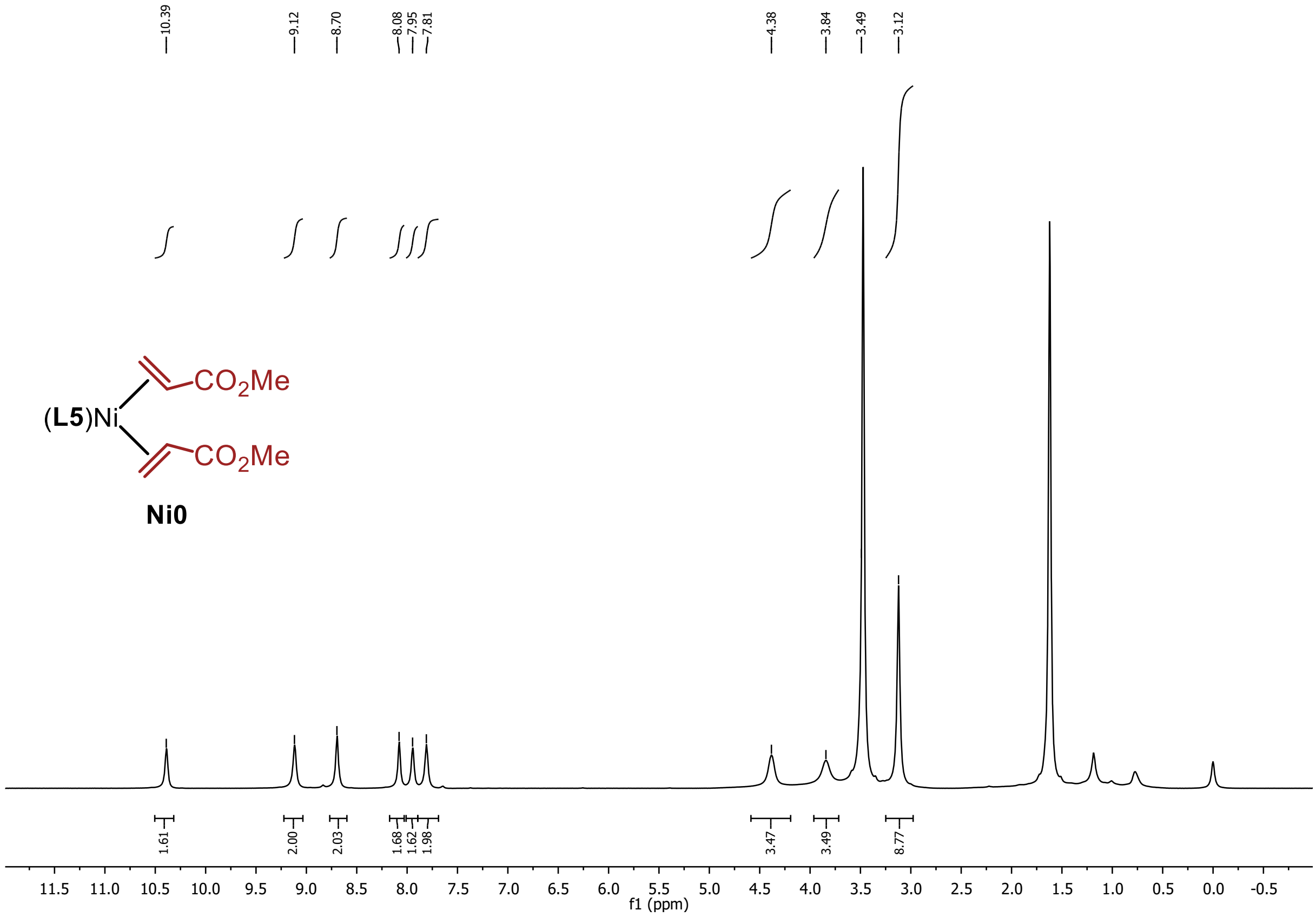

Figure S9. ^{1}H NMR spectrum (600 MHz, THF-d_8) of **Ni0**.

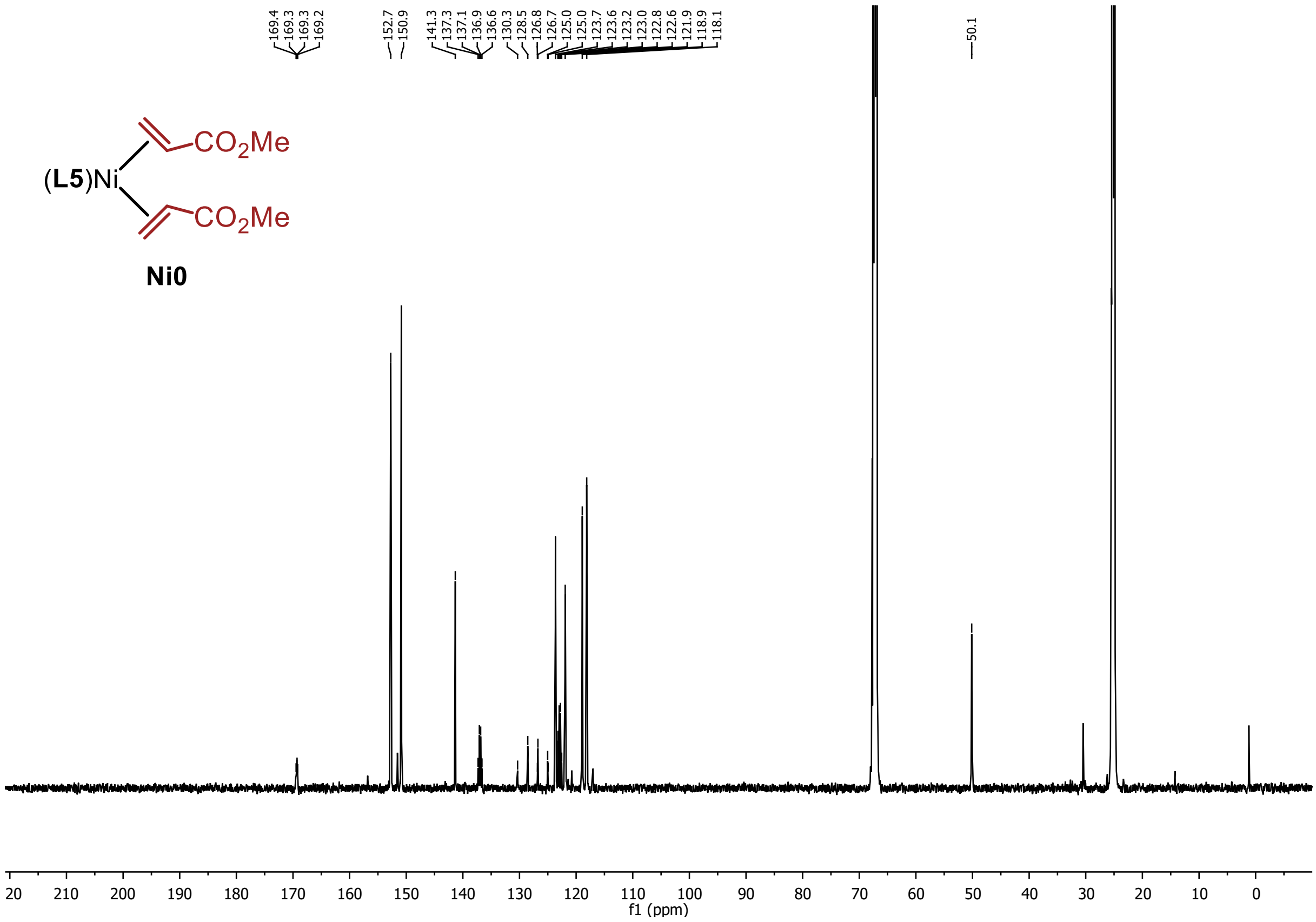

Figure S10. ^{13}C NMR spectrum (151 MHz, THF-d_8) of **Ni0**.

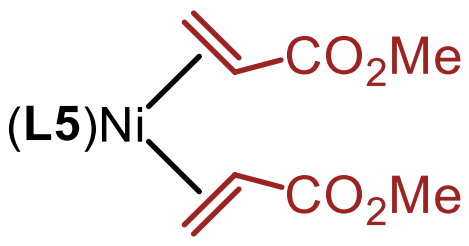

Ni0

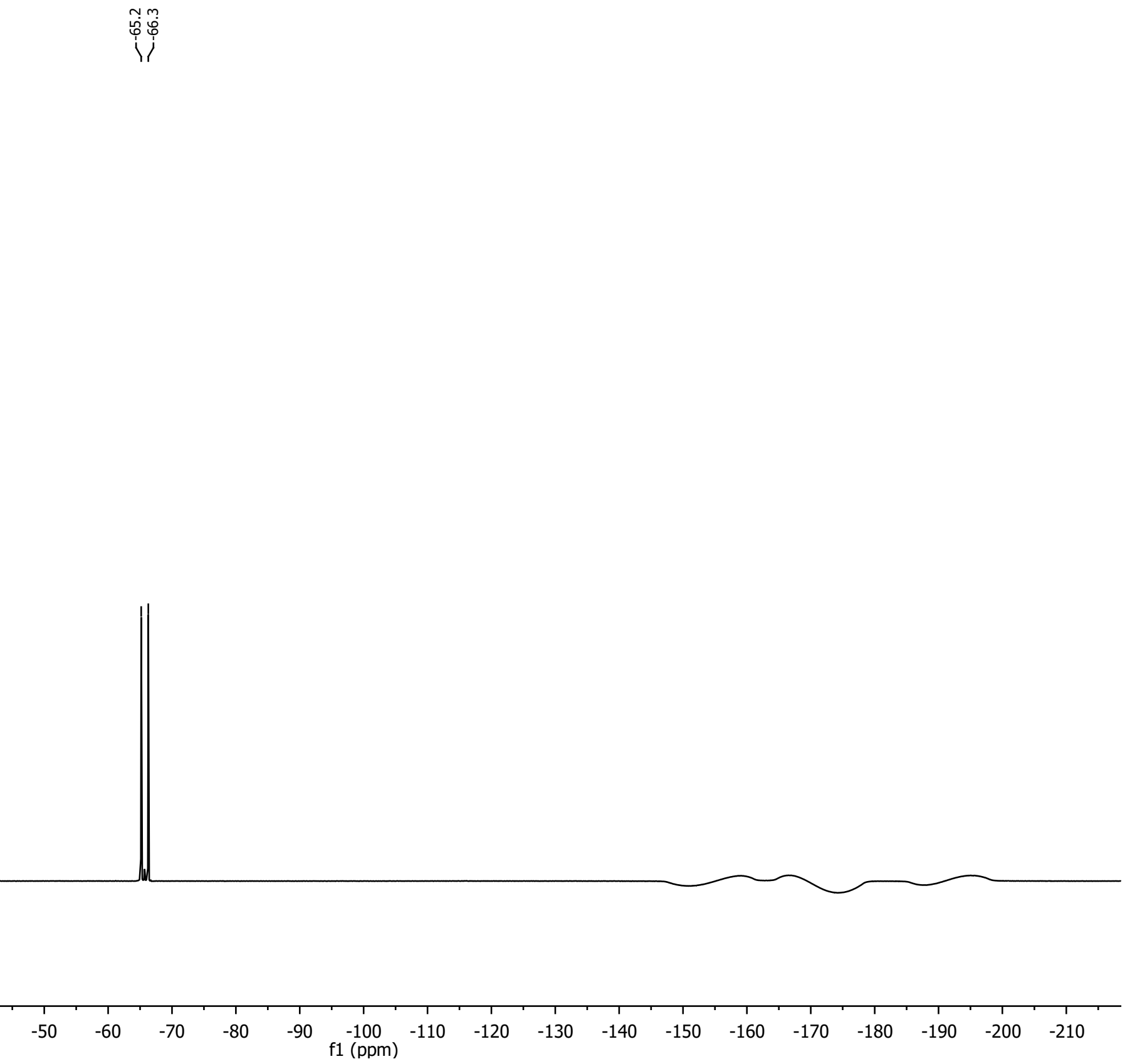

Figure S11. ^{19}F NMR spectrum (565 MHz, THF-d_8) of **Ni0**.

Ni1

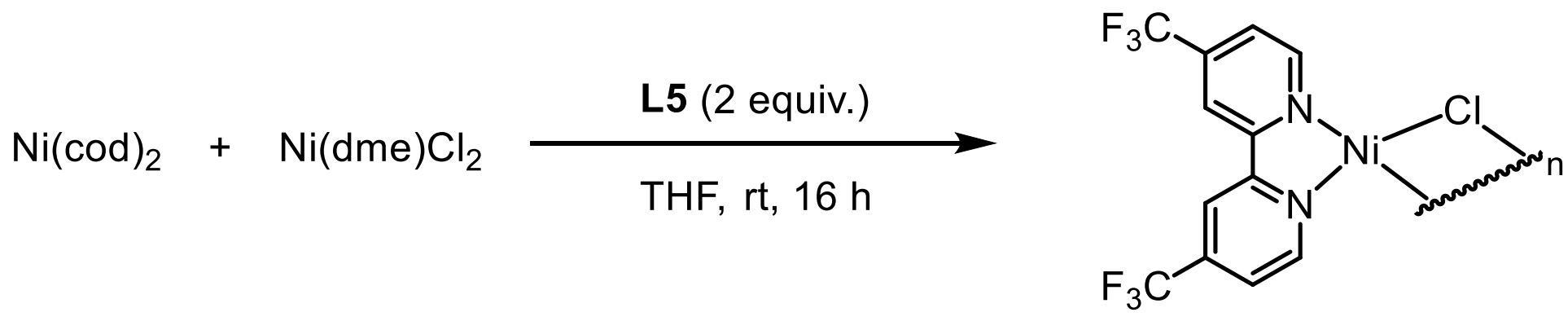

In a screw-cap vial in a glovebox, Ni(cod)$_2$ (88.4 mg, 341 µmol, 0.5 equiv.), nickel(dimethoxyethane)dichloride (75.0 mg, 341 341 µmol, 0.5 equiv.), and 4,4'-bis(trifluoromethyl)-2,2'-bipyridine (**L5**, 199.5 mg, 683 µmol, 1 equiv.) were dissolved in dry THF (3 mL) and stirred for 16 h. *n*-Hexane (5 mL) was added and the mixture was filtered with a frit (P3) and the residue washed with *n*-pentane (3 × 5 mL). The residue was dried under vacuum, affording **Ni1** (164 mg, 425 µmol, 62%) as a grey solid. Paramagnetic NMR data conforms to analytical data of related compounds.[5]

^{1}H NMR (400 MHz, THF-d$_8$/acetone-d$_6$ (1:1), δ): 168.6, 150.5, 60.3, 58.1, 55.9, 45.7, 41.5.

^{19}F NMR (376 MHz, THF-d$_8$/acetone-d$_6$ (1:1), δ): -72.8.

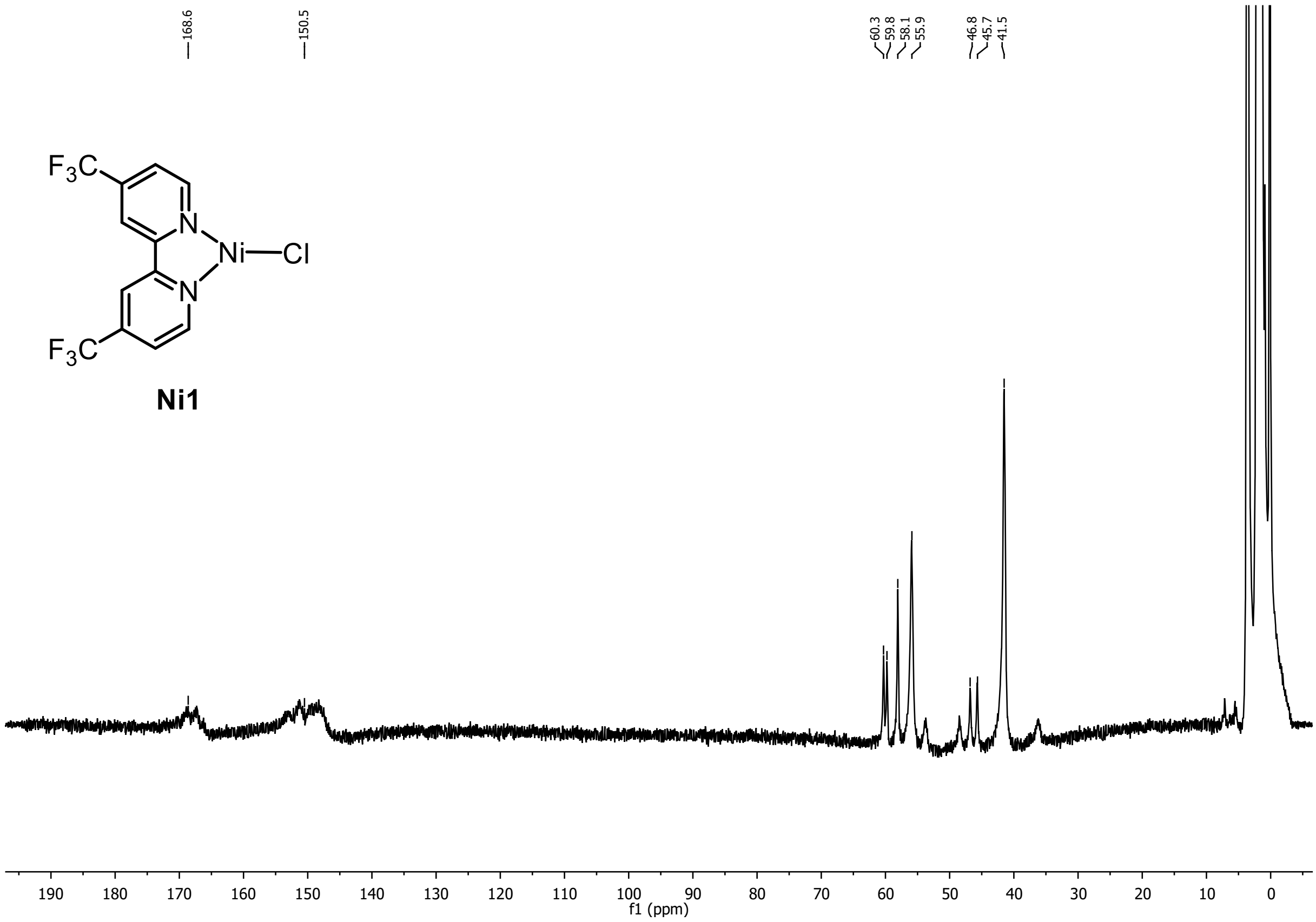

Figure S12. Paramagnetic ^{1}H NMR spectrum (400 MHz, THF-d_8/acetone-d_6 (1:1)) of **Ni1**.

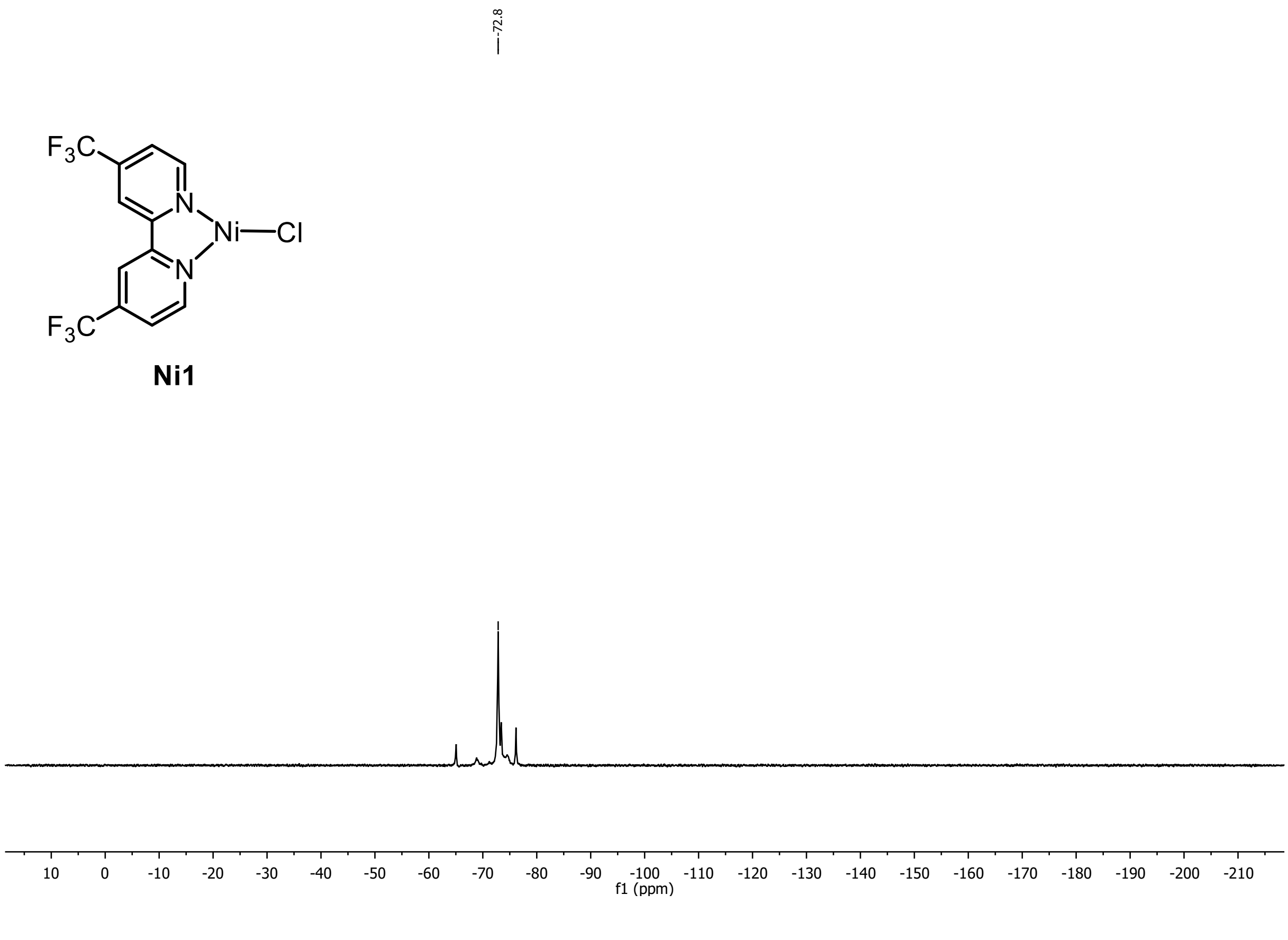

Figure S13. Paramagnetic ^{19}F NMR spectrum (376 MHz, THF-d_8/acetone-d_6 (1:1)) of **Ni1**.

Ni2

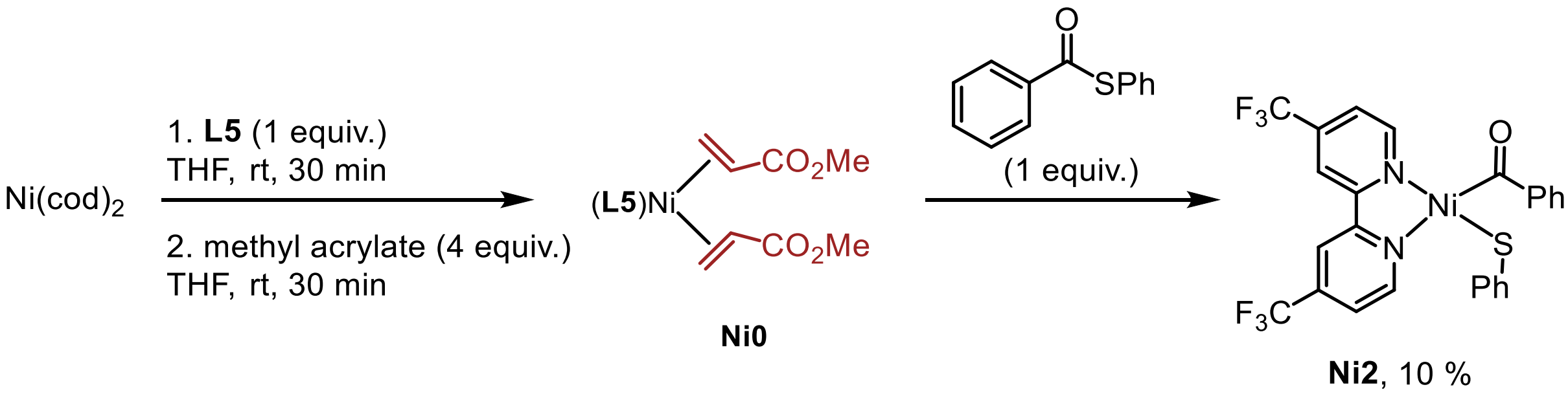

In a Schlenk tube, Ni(cod)$_2$ (100 mg, 386 µmol, 1 equiv.) and 4,4'-bis(trifluoromethyl)-2,2'-bipyridine (**L5**, 113 mg, 386 µmol, 1 equiv.) were dissolved in dry THF (5 mL) and stirred for 30 min, resulting in a deep blue solution. Methyl acrylate (133 mg, 1.54 mmol, 4 equiv.) was added and the mixture was stirred at rt for another 30 min until a brown precipitate formed. The mixture was filtered and the residue washed with *n*-hexane (3 × 5 mL), then suspended in THF (5 mL). Next, *S*-phenyl benzothiate (**S1**, 82.8 mg, 386 µmol, 1 equiv.) was added in one portion and the mixture was stirred for 2 h at rt. Subsequently, volatiles were removed *in vacuo*. The residue washed with *n*-hexane (3 × 5 mL) and dried under vacuum (*note: **Ni2** was partly lost in this purification step, as remaining polar compounds appeared to increase the solubility of **Ni2** in the washing solution*). **Ni2** (21 mg, 37.2 µmol, 10%) was received as a black solid.

^{1}H NMR (400 MHz, THF-d$_8$, δ): 8.99 – 8.90 (m, 2H), 8.78 (s, 1H), 8.04 – 8.00 (m, 1H), 7.79 – 7.72 (m, 3H), 7.69 – 7.54 (m, 2H), 7.54 – 7.35 (m, 7H).

^{13}C{^{1}H} NMR (151 MHz, THF-d$_8$, δ): 188.9, 156.8, 151.6, 139.8 (q, J = 34.2 Hz), 135.7, 134.3, 130.4, 129.9, 129.7, 129.5, 129.3, 127.9, 127.5, 124.0 (q, J = 272.9 Hz), 120.8, 117.0.

^{19}F NMR (376 MHz, THF-d$_8$, δ): -64.9.

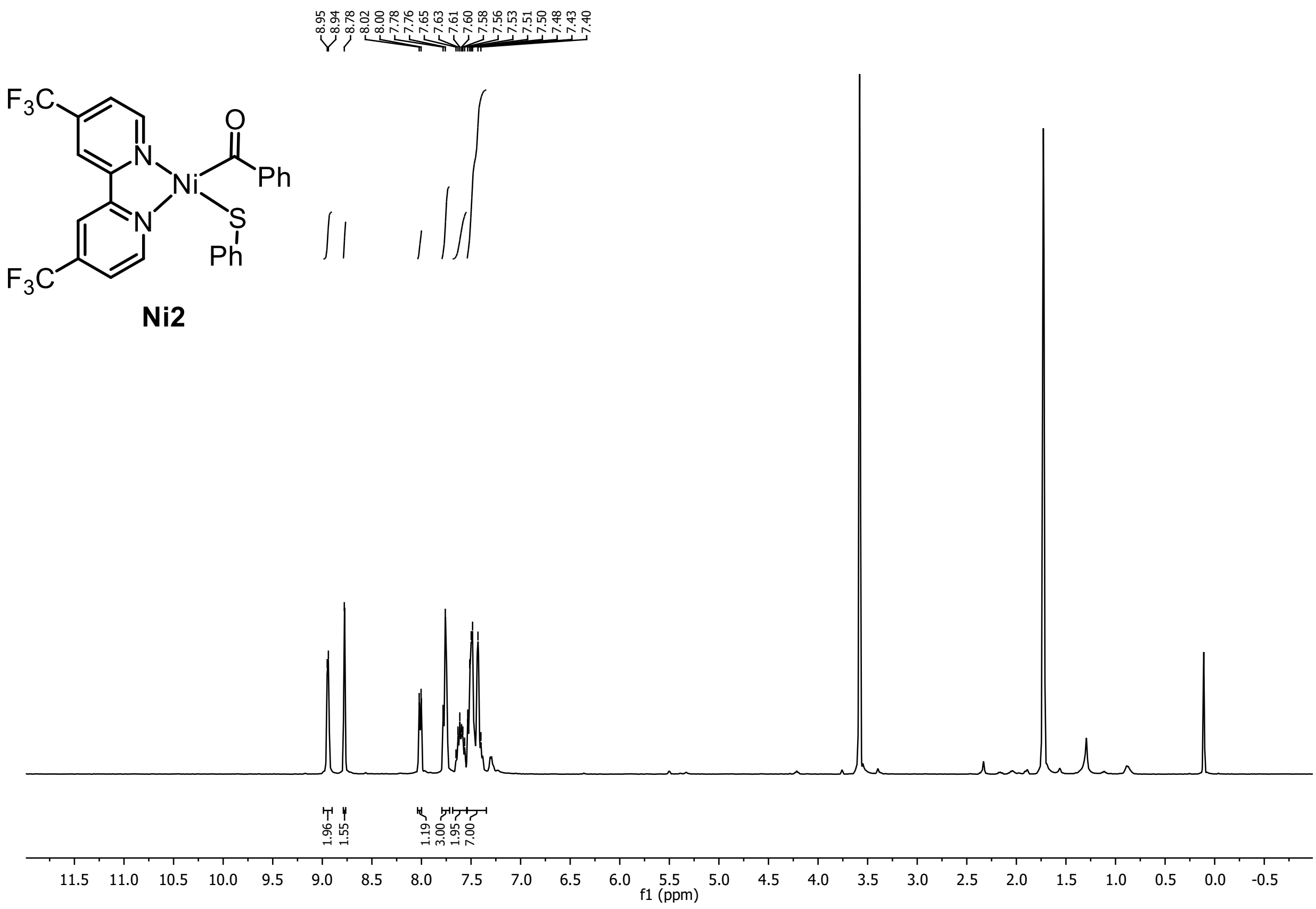

Figure S14. ^{1}H NMR spectrum (400 MHz, THF-d_8) of **Ni2**.

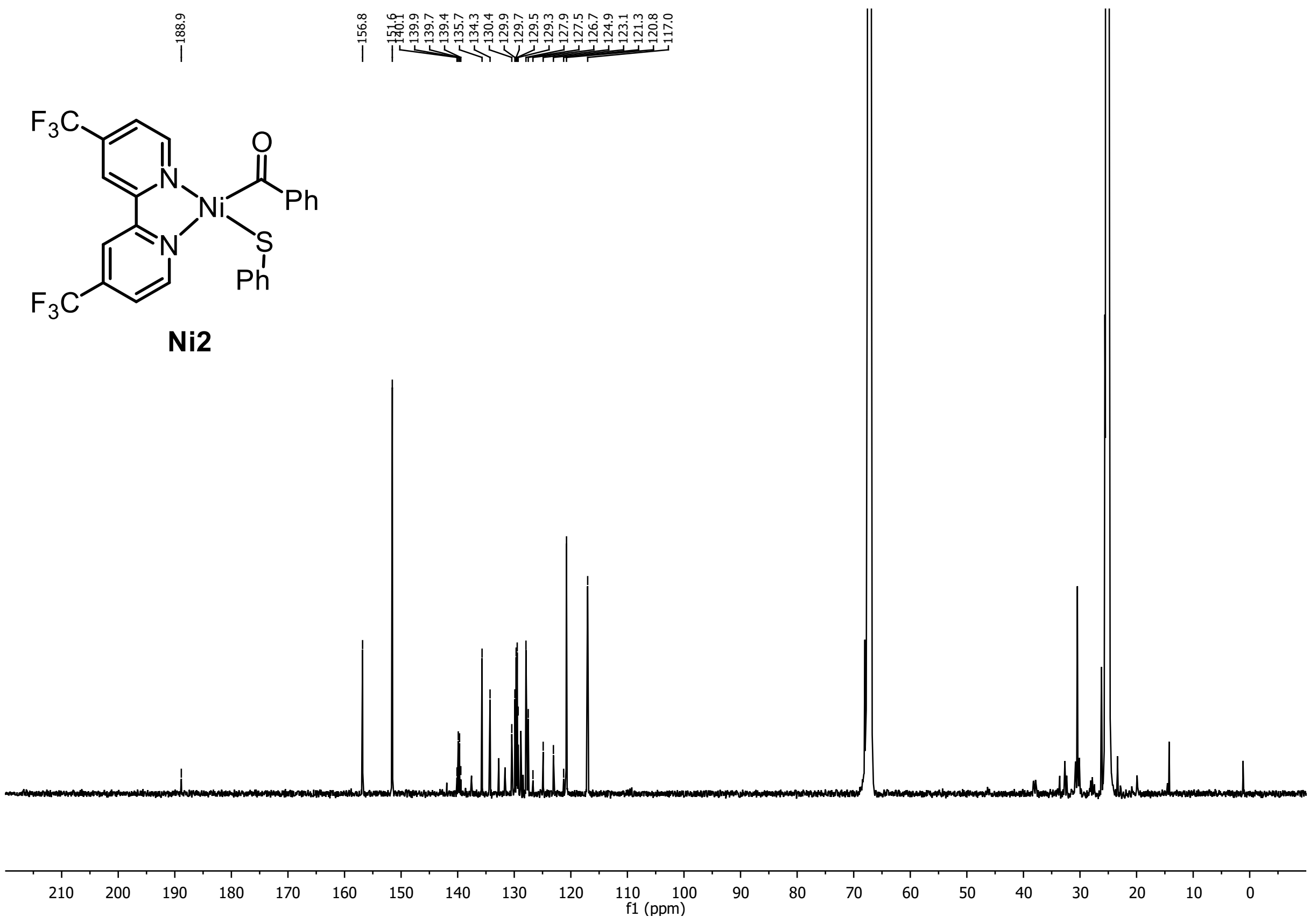

Figure S15. ^{13}C NMR spectrum (151 MHz, THF-d_8) of **Ni2**.

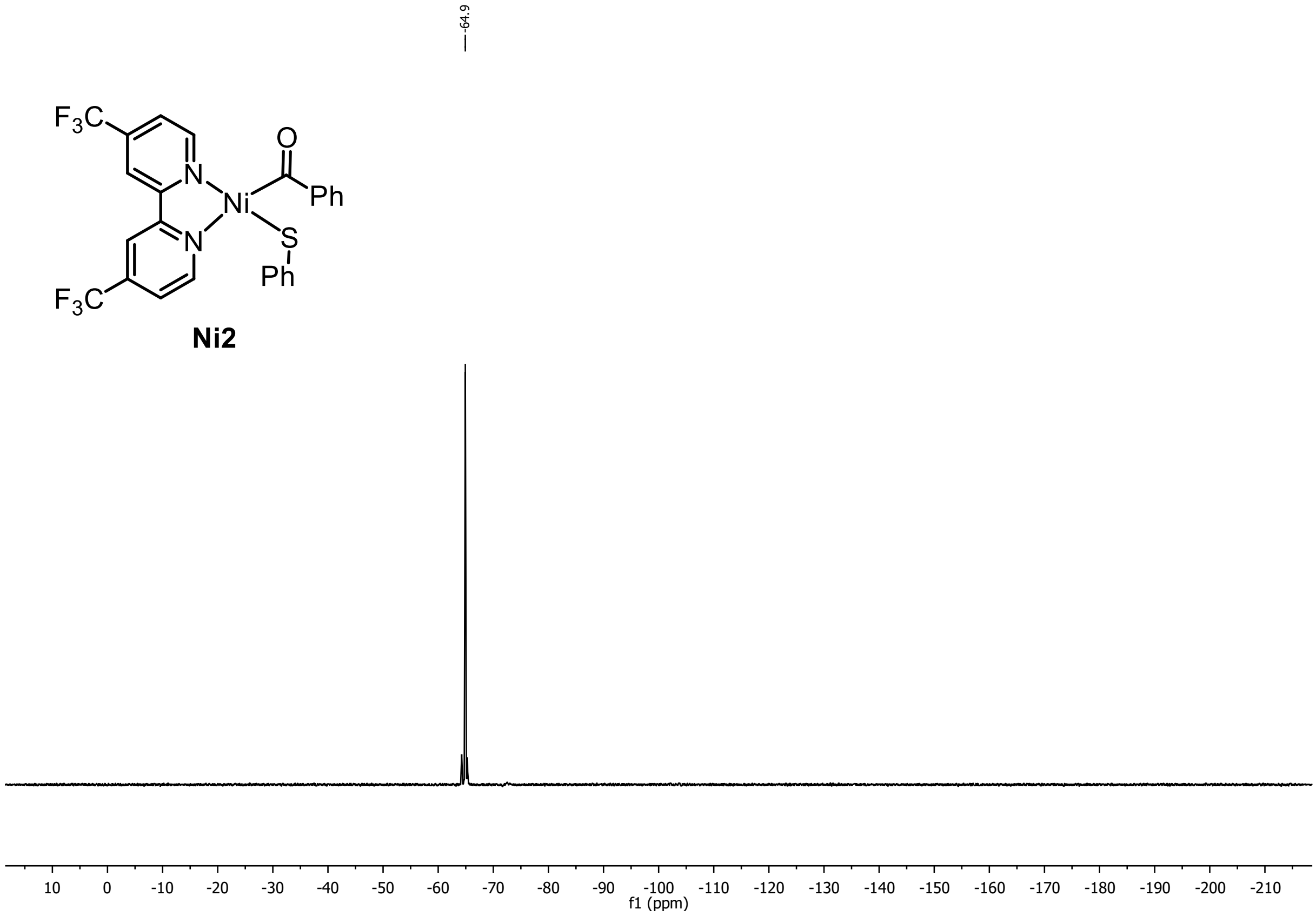

Figure S16. ^{19}F NMR spectrum (376 MHz, THF-d_8) of **Ni2**.

5.2 Synthesis of Thioester

***S*-phenyl benzothioate (S1):**

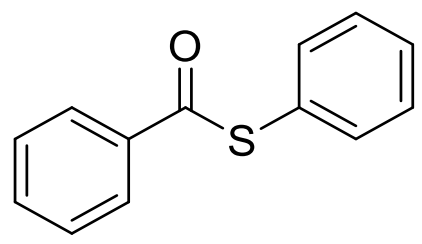

S1

$C_{13}H_{10}OS$ (214.28 g/mol)

Following **GP-A1**, **S1** was synthesized using benzoyl chloride (20.9 mL, 180 mmol, 1.0 equiv.), thiophenol (22.1 mL, 216 mmol, 1.2 equiv.) and triethylamine (25 mL, 180 mmol, 1.0 equiv.). Purification by crystallization (DCM/*n*-hexane) afforded **S1** (24.1 g, 113 mmol, 63%) as colorless solid. Conforms to reported analytical data.[6]

^{1}H NMR (400 MHz, $CDCl_3$, δ): 8.10 – 8.03 (m, 2H), 7.66 – 7.45 (m, 8H).

^{13}C{^{1}H} NMR (101 MHz, $CDCl_3$, δ): 190.2, 136.7, 135.2, 133.7, 129.6, 129.3, 128.8, 127.6, 127.5.

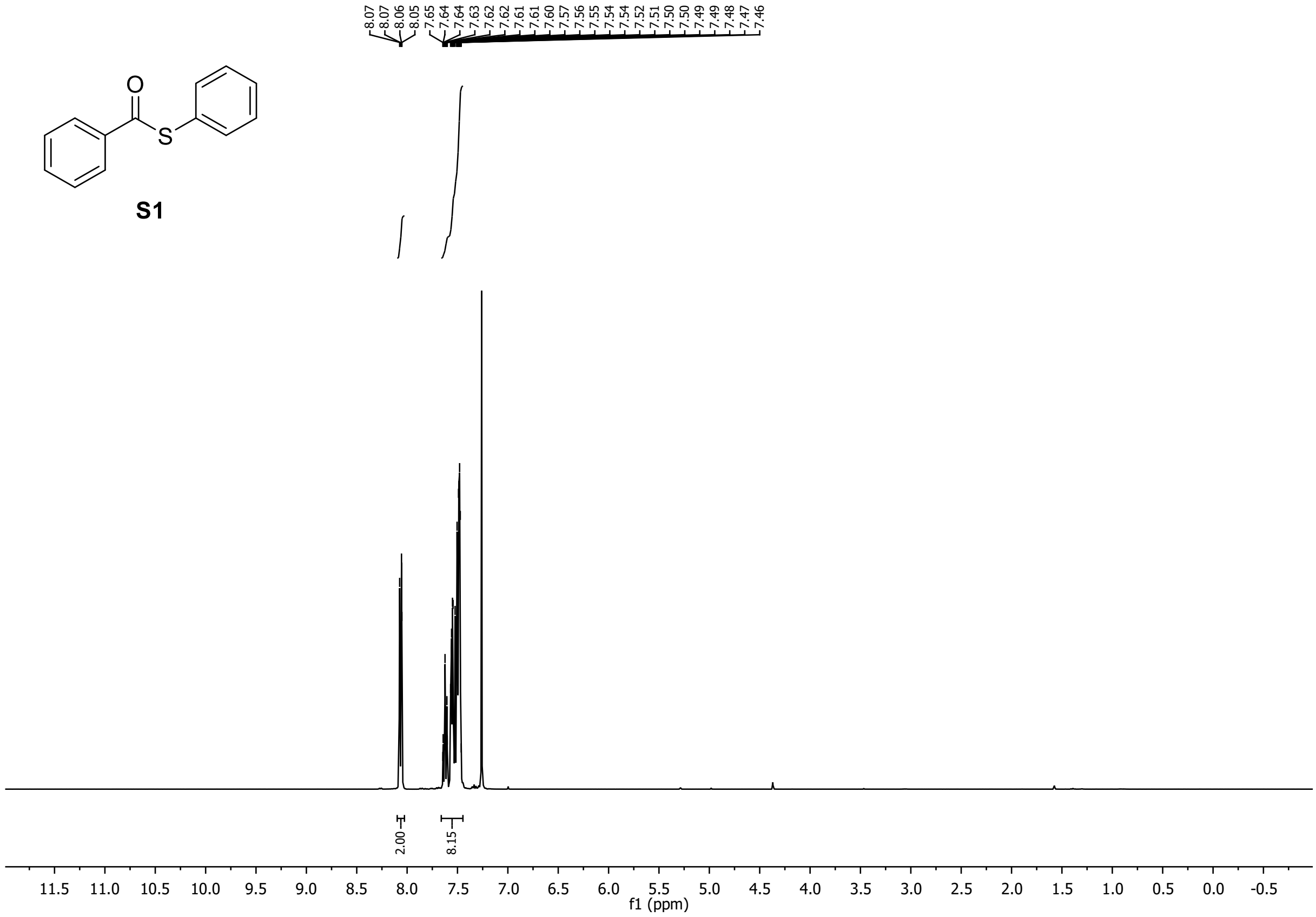

Figure S17. ^{1}H NMR spectrum (400 MHz, $CDCl_3$) of **S1**.

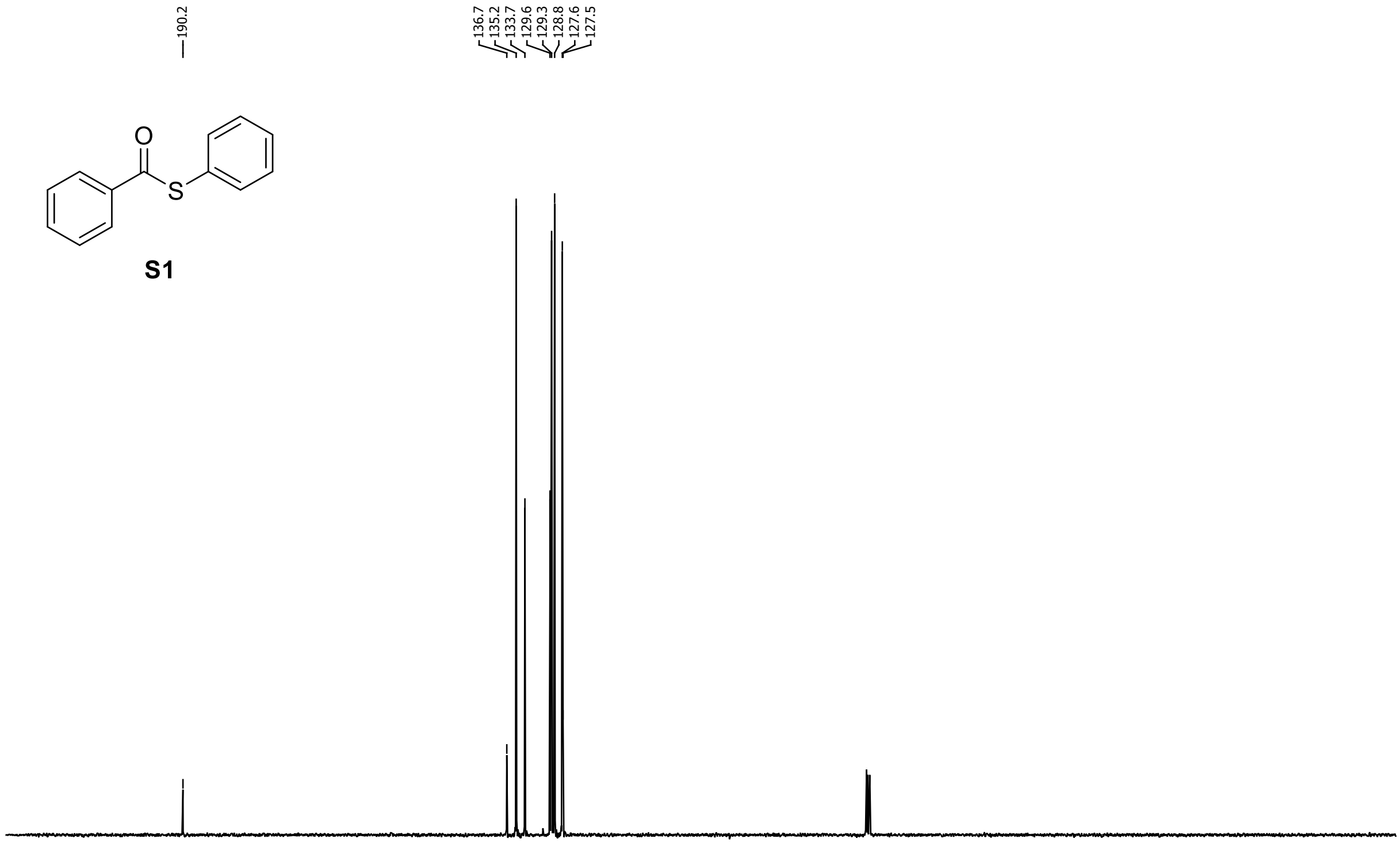

Figure S18. ^{13}C NMR spectrum (101 MHz, $CDCl_3$) of **S1**.

S-phenyl 2-methylbenzothioate (S2):

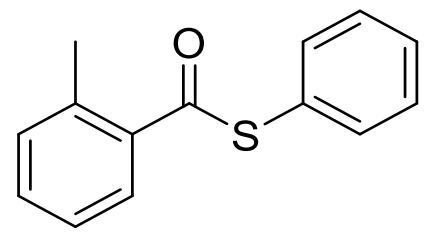

S2

$C_{14}H_{12}OS$ (228.31 g/mol)

Following **GP-A1**, **S2** was synthesized using 2-methylbenzoyl chloride (2.29 mL, 20.0 mmol, 1.0 equiv.), thiophenol (2.45 mL, 24.0 mmol, 1.2 equiv.) and triethylamine (3.34 mL, 24.0 mmol, 1.2 equiv.). Purification by crystallization (DCM/*n*-hexane) afforded **S2** (0.55 g, 2.40 mmol, 12%) as colorless solid. Conforms to reported analytical data.[6]

^{1}H NMR (400 MHz, $CDCl_3$, δ): 7.96 (dd, *J* = 7.8, 1.3 Hz, 1H), 7.56 – 7.40 (m, 6H), 7.35 – 7.26 (m, 2H), 2.51 (s, 3H).

^{13}C{^{1}H} NMR (101 MHz, $CDCl_3$, δ): 192.3, 137.6, 136.9, 135.0, 132.1, 131.9, 129.6, 129.4, 128.8, 128.4, 126.0, 20.9.

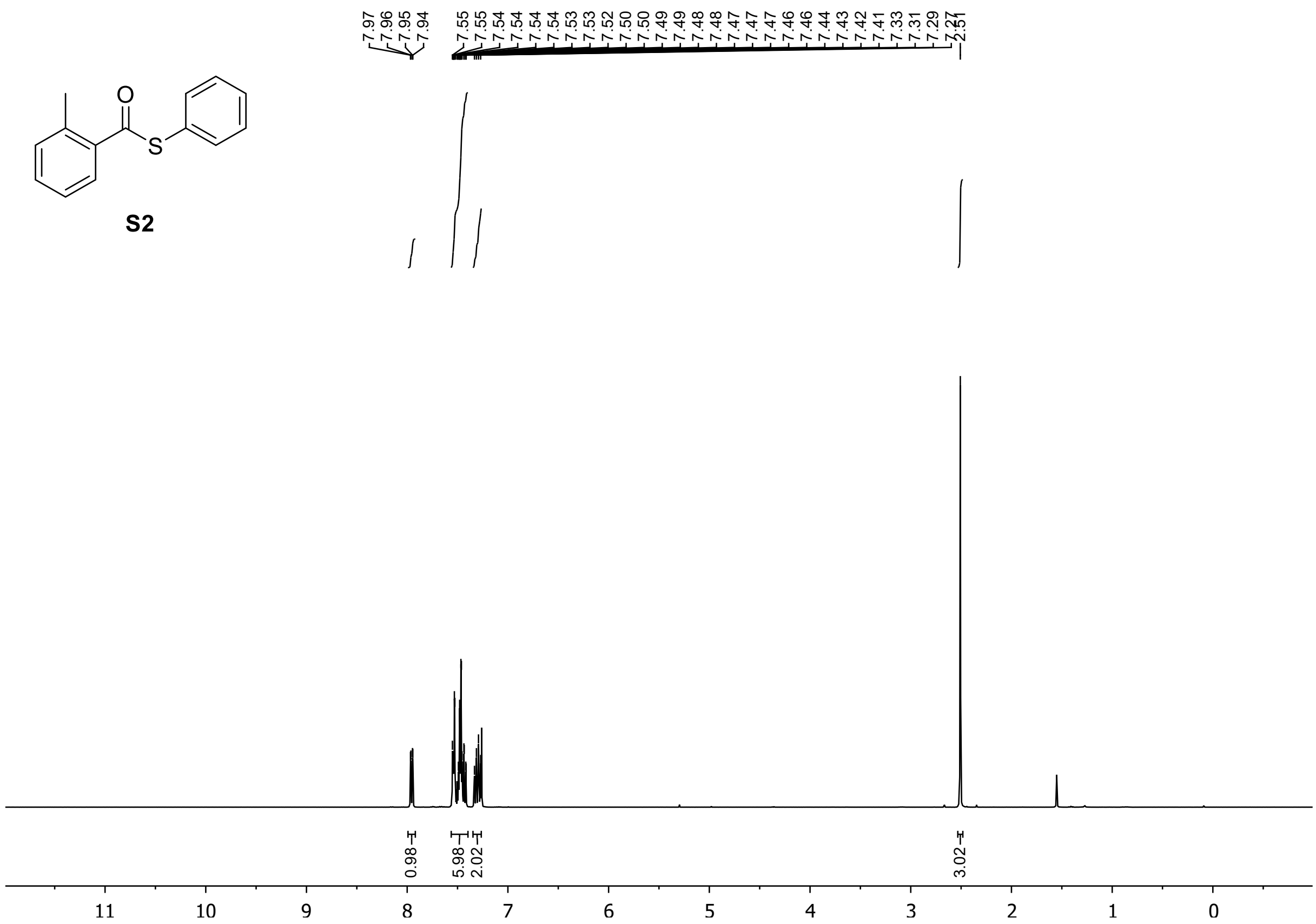

Figure S19. ^{1}H NMR spectrum (400 MHz, $CDCl_3$) of **S2**.

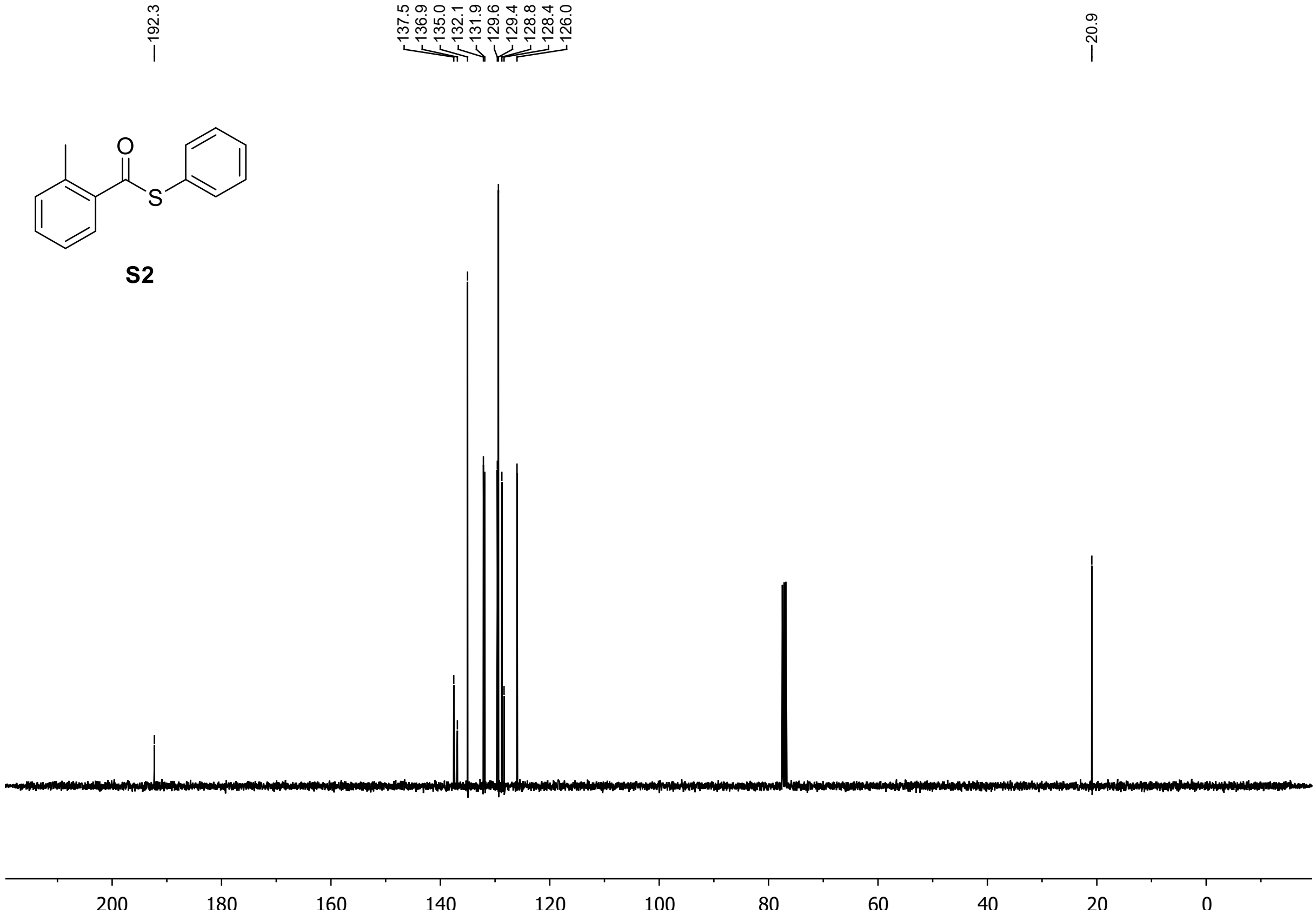

Figure S20. ^{13}C NMR spectrum (101 MHz, $CDCl_3$) of **S2**.

***S*-Phenyl 4-(*tert*-butyl)benzothioate (S3):**

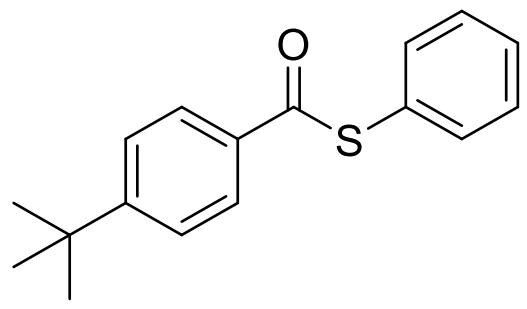

S3

$C_{17}H_{18}OS$ (270.39 g/mol)

Following **GP-1**, **S3** was synthesized using 4-(*tert*-butyl)benzoyl chloride (3.93 g, 20.0 mmol, 1.0 equiv.), thiophenol (2.20 mL, 21.0 mmol, 1.05 equiv.) and triethylamine (3.35 mL, 24.0 mmol, 1.05 equiv.). Purification by crystallization (DCM/*n*-hexane) afforded **S3** (2.01 g, 7.43 mmol, 37%) as colorless solid. Conforms to reported analytical data.[7]

^{1}H NMR (400 MHz, $CDCl_3$, δ): 8.02 – 7.92 (m, 2H), 7.57 – 7.43 (m, 7H), 1.36 (s, 9H).

^{13}C{^{1}H} NMR (101 MHz, $CDCl_3$, δ): 189.8, 157.7, 135.3, 134.2, 129.5, 129.3, 127.7, 127.6, 125.9, 35.4, 31.2.

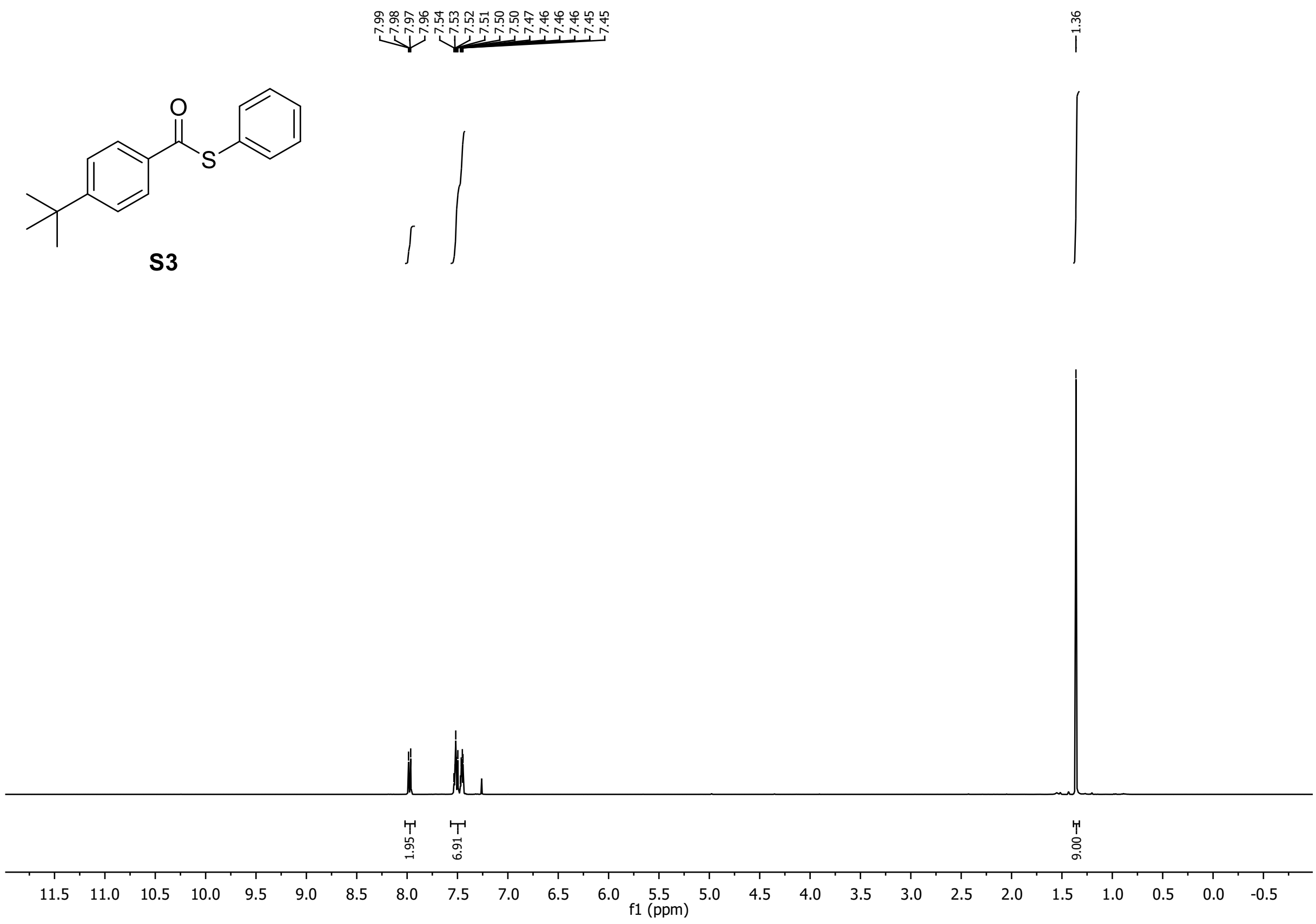

Figure S21. ^{1}H NMR spectrum (400 MHz, $CDCl_3$) of **S3**.

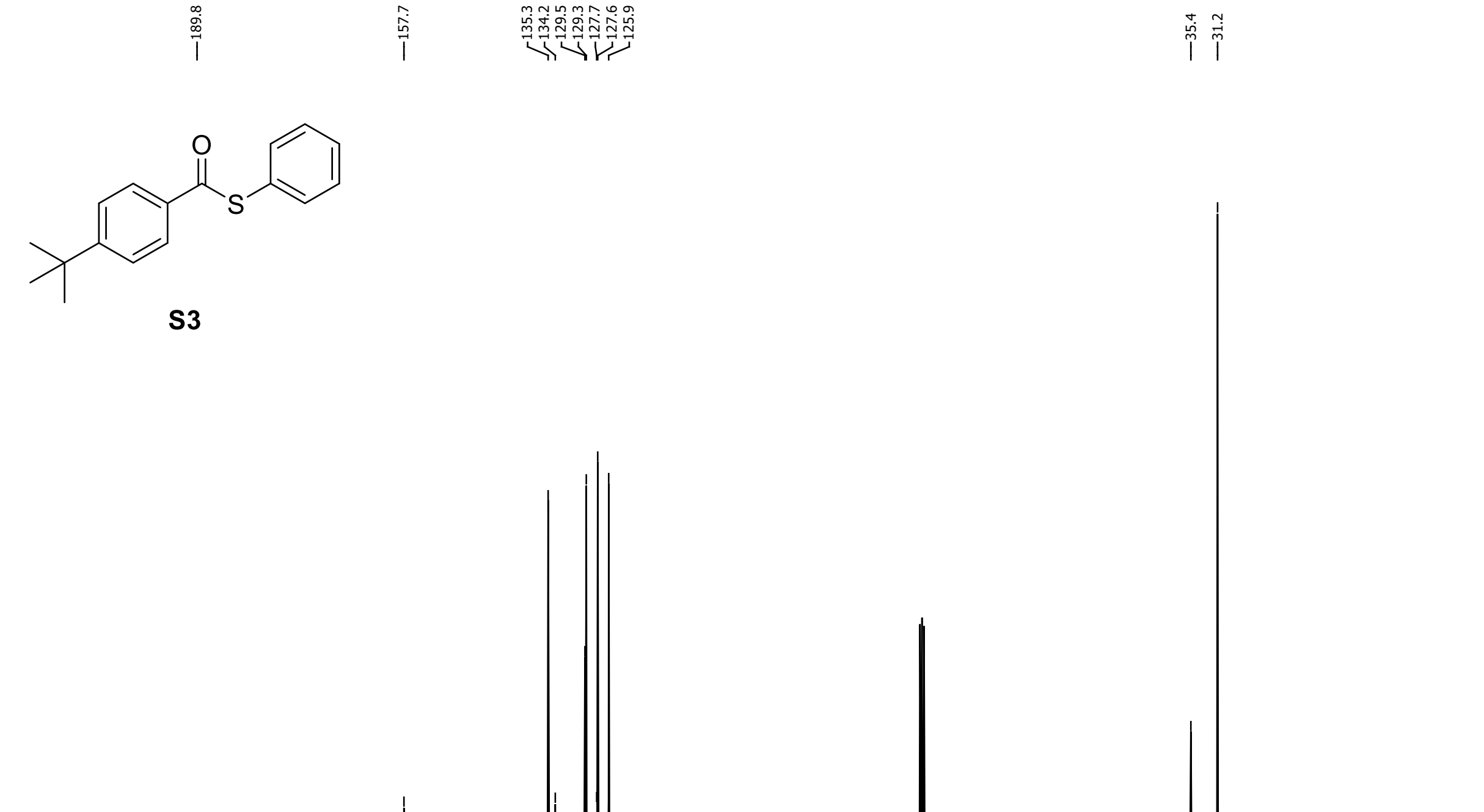

Figure S22. ^{13}C NMR spectrum (101 MHz, $CDCl_3$) of **S3.**

***S*-Phenyl naphthalene-2-carbothioate (S4):**

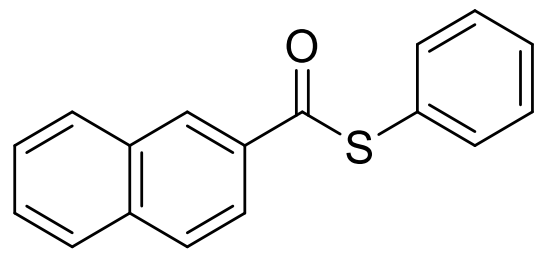

S4

$C_{17}H_{12}OS$ (264.34 g/mol)

Following **GP-1**, **S4** was synthesized using 2-naphthoyl chloride (3.81 g, 20.0 mmol, 1.0 equiv.), thiophenol (2.20 mL, 21.0 mmol, 1.05 equiv.) and triethylamine (3.35 mL, 24.0 mmol, 1.05 equiv.). Purification by crystallization (DCM/*n*-hexane) afforded **S4** (2.65 g, 10.0 mmol, 50%) as colorless solid. Conforms to reported analytical data.[7]

^{1}H NMR (400 MHz, $CDCl_3$, δ): 8.63 (s, 1H), 8.07 – 7.98 (m, 2H), 7.95 – 7.88 (m, 2H), 7.67 – 7.54 (m, 4H), 7.53 – 7.45 (m, 3H).

^{13}C{^{1}H} NMR (101 MHz, $CDCl_3$, δ): 190.2, 136.0, 135.3, 134.1, 132.6, 129.8, 129.7, 129.4, 129.2, 128.8, 128.0, 127.6, 127.2, 123.4.

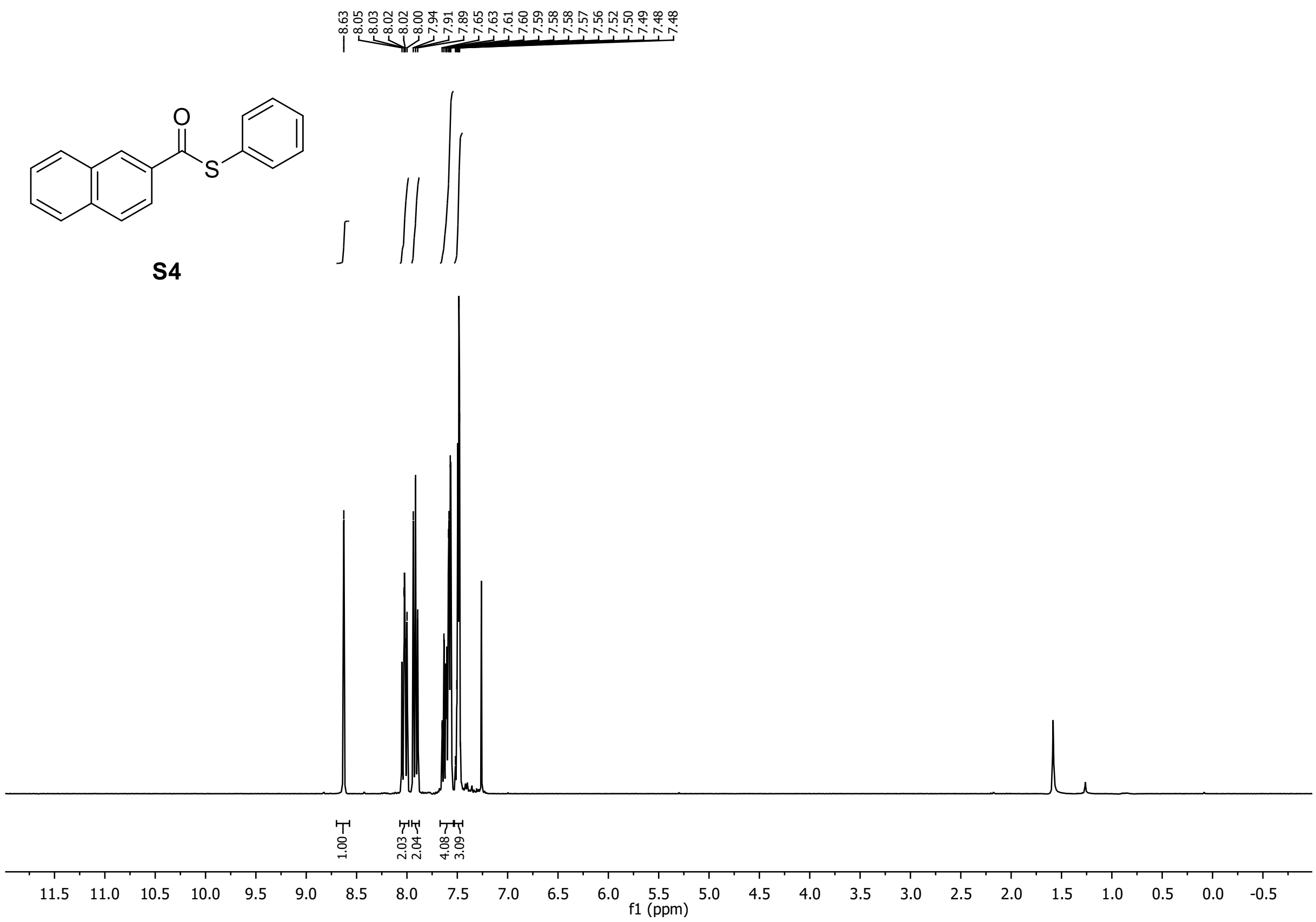

Figure S23. ^{1}H NMR spectrum (400 MHz, $CDCl_3$) of **S4**.

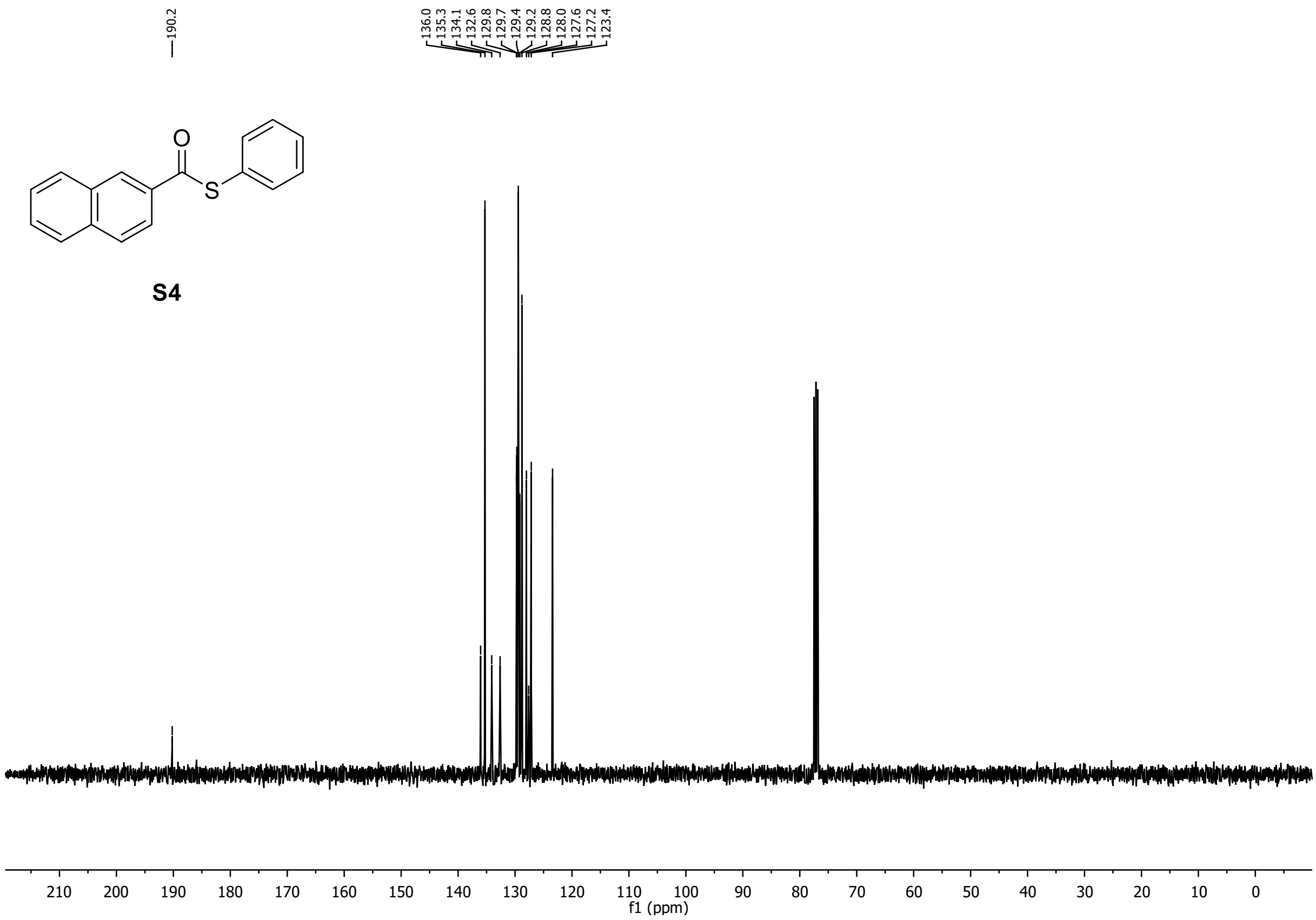

Figure S24. ^{13}C NMR spectrum (101 MHz, $CDCl_3$) of **S4**.

***S*-phenyl 3-chlorobenzothioate (S5):**

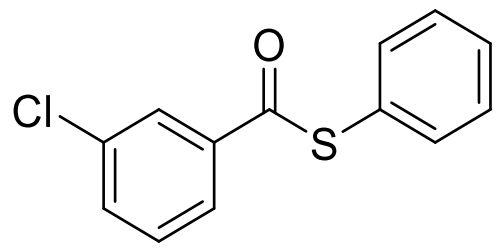

S5

$C_{13}H_9ClOS$ (248.72 g/mol)

Following **GP-A1**, **S5** was synthesized using 3-chlorobenzoyl chloride (2.57 mL, 20.0 mmol, 1.0 equiv.), thiophenol (2.45 mL, 24.0 mmol, 1.2 equiv.) and triethylamine (3.34 mL, 24.0 mmol, 1.2 equiv.). Purification by crystallization (DCM/*n*-hexane) afforded **S5** (2.53 g, 10.2 mmol, 51%) as colorless solid. Conforms to reported analytical data.[2]

^{1}H NMR (400 MHz, $CDCl_3$, δ): 8.00 (m, 1H), 7.92 (m, 1H), 7.58 (m, 1H), 7.55 – 7.40 (m, 6H).

^{13}C{^{1}H} NMR (101 MHz, $CDCl_3$, δ): 189.1, 138.3, 135.1, 133.7, 130.2, 129.9, 129.5, 127.6, 126.9, 125.7.

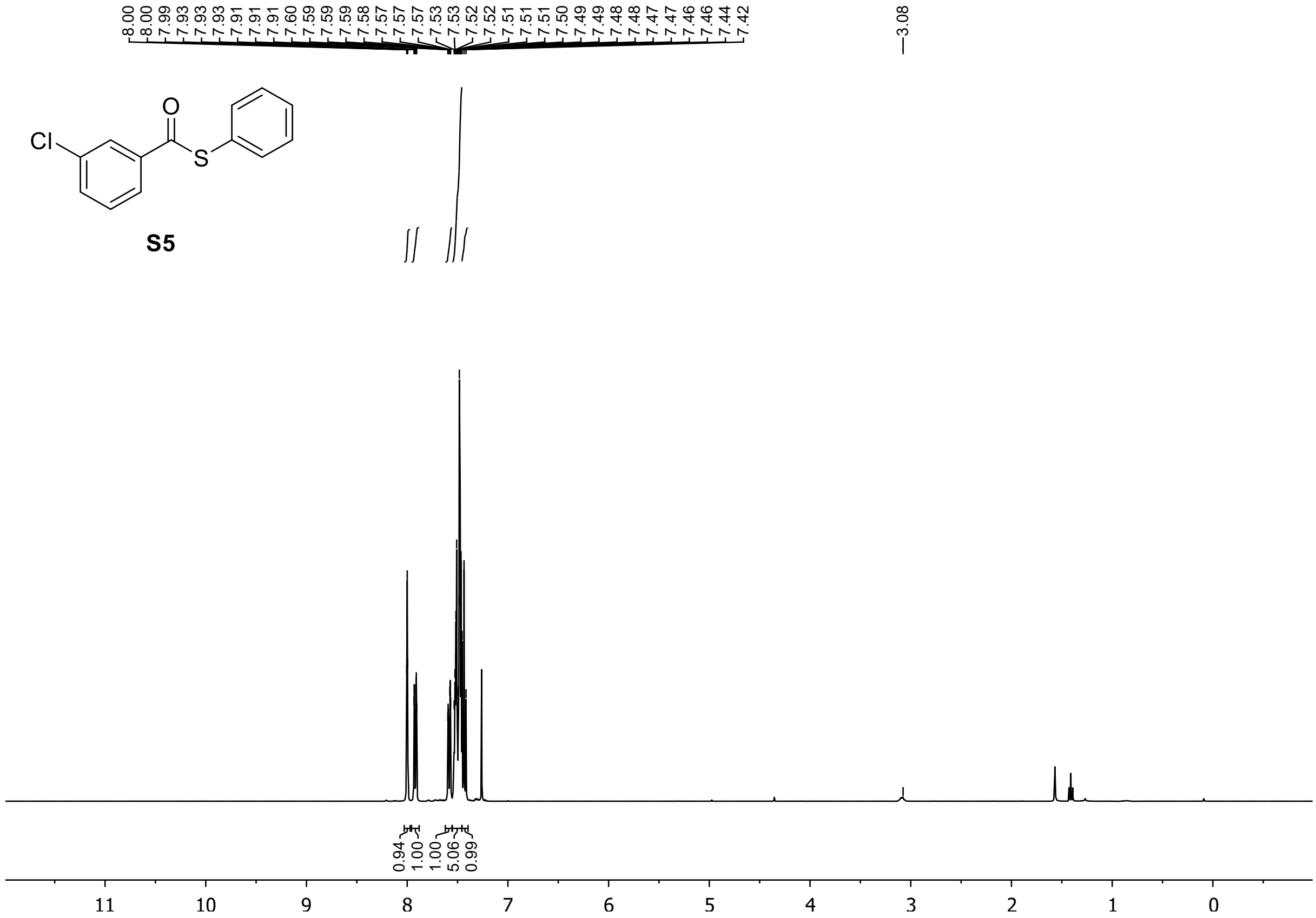

Figure S25. ^{1}H NMR spectrum (400 MHz, $CDCl_3$) of **S5**.

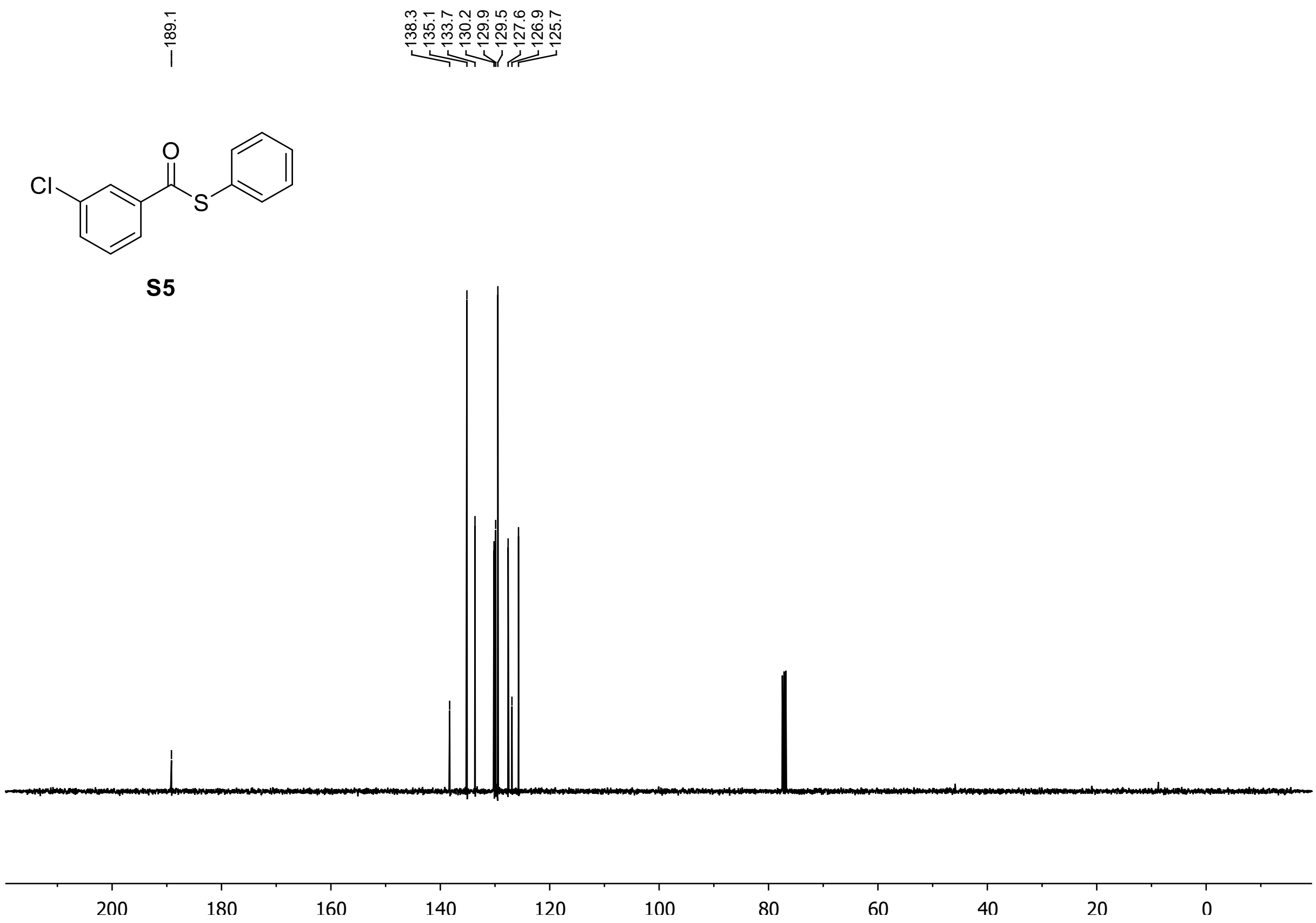

Figure S26. ^{13}C NMR spectrum (101 MHz, $CDCl_3$) of **S5**.

***S*-Phenyl 3-bromobenzothioate (S6):**

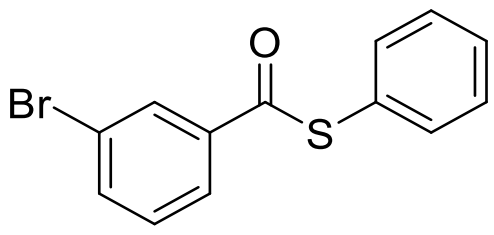

S6

$C_{13}H_9BrOS$ (293.18 g/mol)

Following **GP-1**, **S6** was synthesized using 3-bromobenzoyl chloride (2.20 g, 10.0 mmol, 1.0 equiv.), thiophenol (1.03 mL, 10.5 mmol, 1.05 equiv.) and triethylamine (1.53 mL, 11.0 mmol, 1.1 equiv.). Purification by crystallization (DCM/*n*-hexane) afforded **S6** (2.77 g, 9.45 mmol, 95%) as yellow oil. Conforms to reported analytical data.[8]

^{1}H NMR (400 MHz, $CDCl_3$, δ): 8.15 (dd, *J* = 1.8, 1.8 Hz, 1H), 8.00 – 7.92 (m, 1H), 7.77 – 7.70 (m, 1H), 7.54 – 7.46 (m, 5H), 7.37 (dd, *J* = 7.9, 7.9 Hz, 1H).

^{13}C{^{1}H} NMR (101 MHz, $CDCl_3$, δ): 189.0, 138.5, 136.6, 135.1, 130.5, 130.4, 129.9, 129.5, 126.9, 126.1, 123.1.

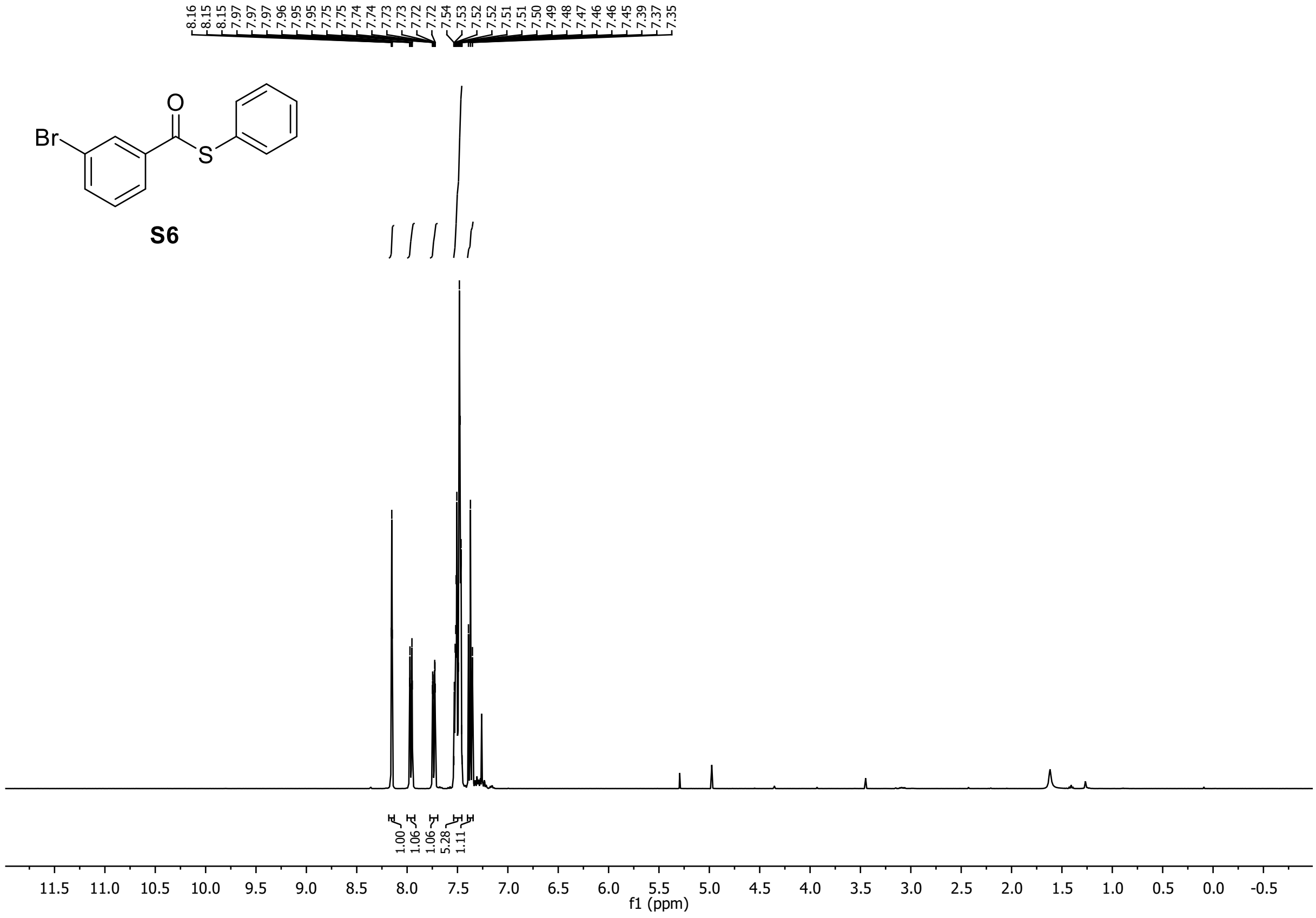

Figure S27. ^{1}H NMR spectrum (400 MHz, $CDCl_3$) of **S6**.

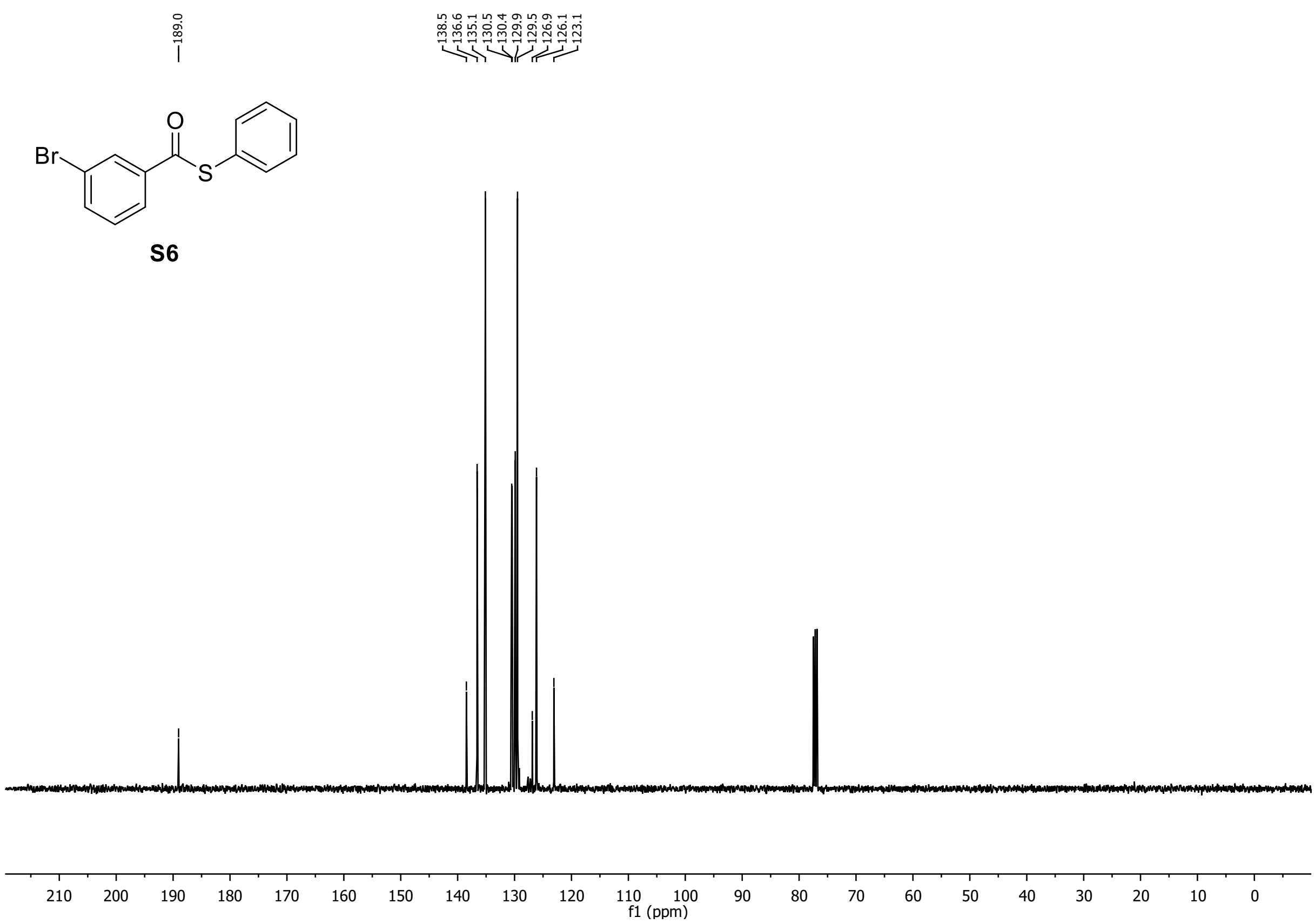

Figure S28. ^{13}C NMR spectrum (101 MHz, $CDCl_3$) of **S6**.

***S*-Phenyl 4-(trifluoromethyl)benzothioate (S7):**

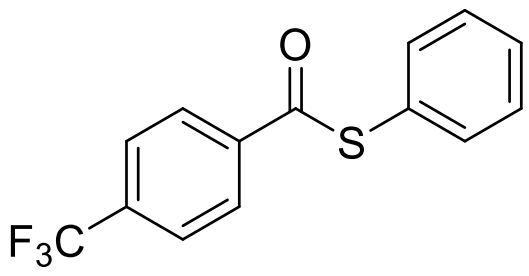

S7

$C_{14}H_9F_3OS$ (282.28 g/mol)

Following **GP-A1**, **S7** was synthesized using 4-(trifluoromethyl)benzoyl chloride (3.07 mL, 20.0 mmol, 1.0 equiv.), thiophenol (2.45 mL, 24.0 mmol, 1.2 equiv.) and triethylamine (3.34 mL, 24.0 mmol, 1.2 equiv.). Purification by crystallization (DCM/*n*-hexane) afforded **S7** (4.39 g, 15.6 mmol, 78%) as colorless solid. Conforms to reported analytical data.[9]

^{1}H NMR (400 MHz, $CDCl_3$, δ): 8.14 (d, *J* = 8.0 Hz, 2H), 7.76 (d, *J* = 8.2 Hz, 2H), 7.58 – 7.44 (m, 5H).

^{13}C{^{1}H} NMR (101 MHz, $CDCl_3$, δ): 189.5, 139.6, 135.1, 130.0, 129.6, 128.0, 126.7, 126.0 (q, *J* = 3.7 Hz), 123.6 (q, *J* = 272.7 Hz).

^{19}F NMR (376 MHz, $CDCl_3$, δ): -63.1.

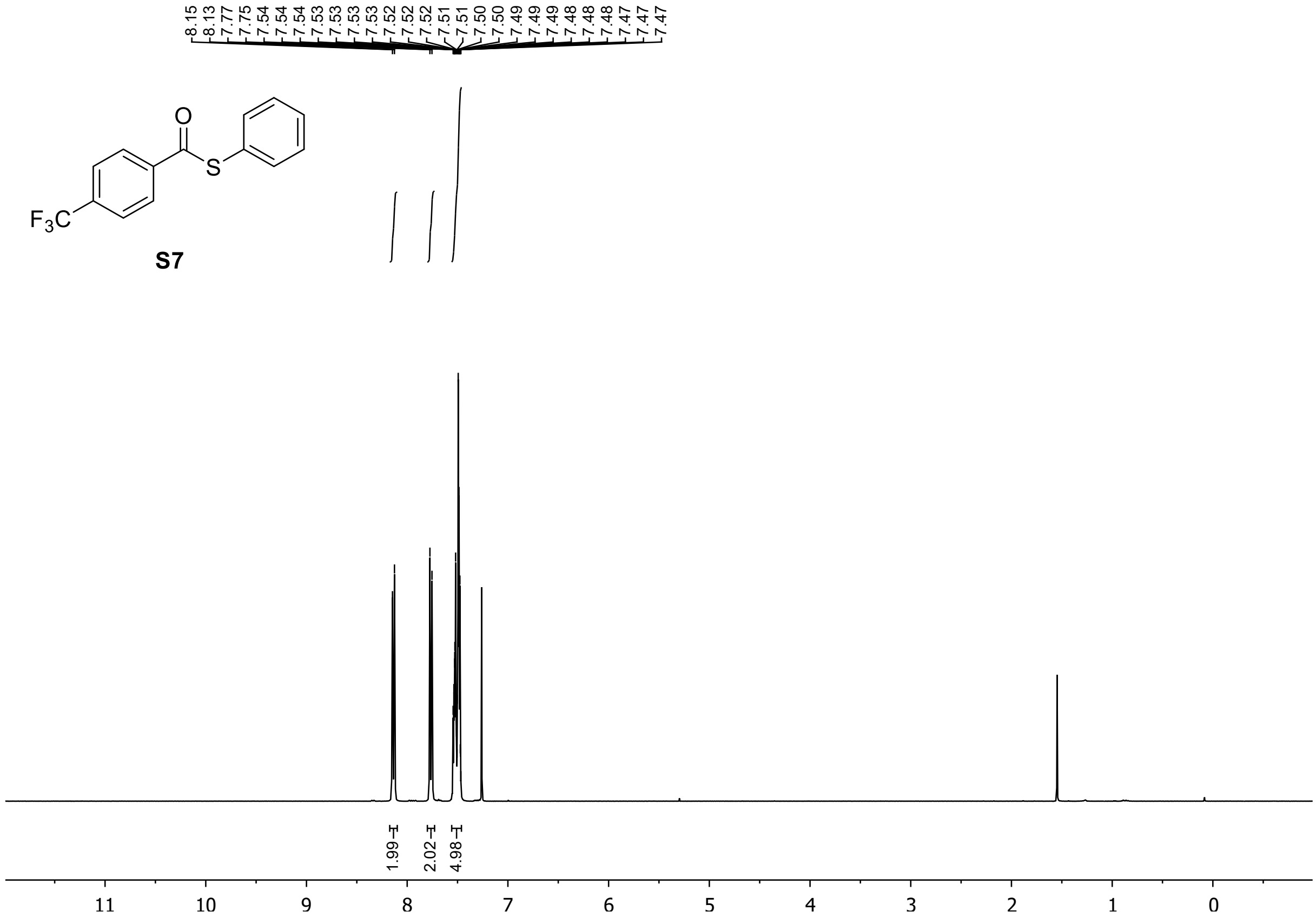

Figure S29. ^{1}H NMR spectrum (400 MHz, $CDCl_3$) of **S7**.

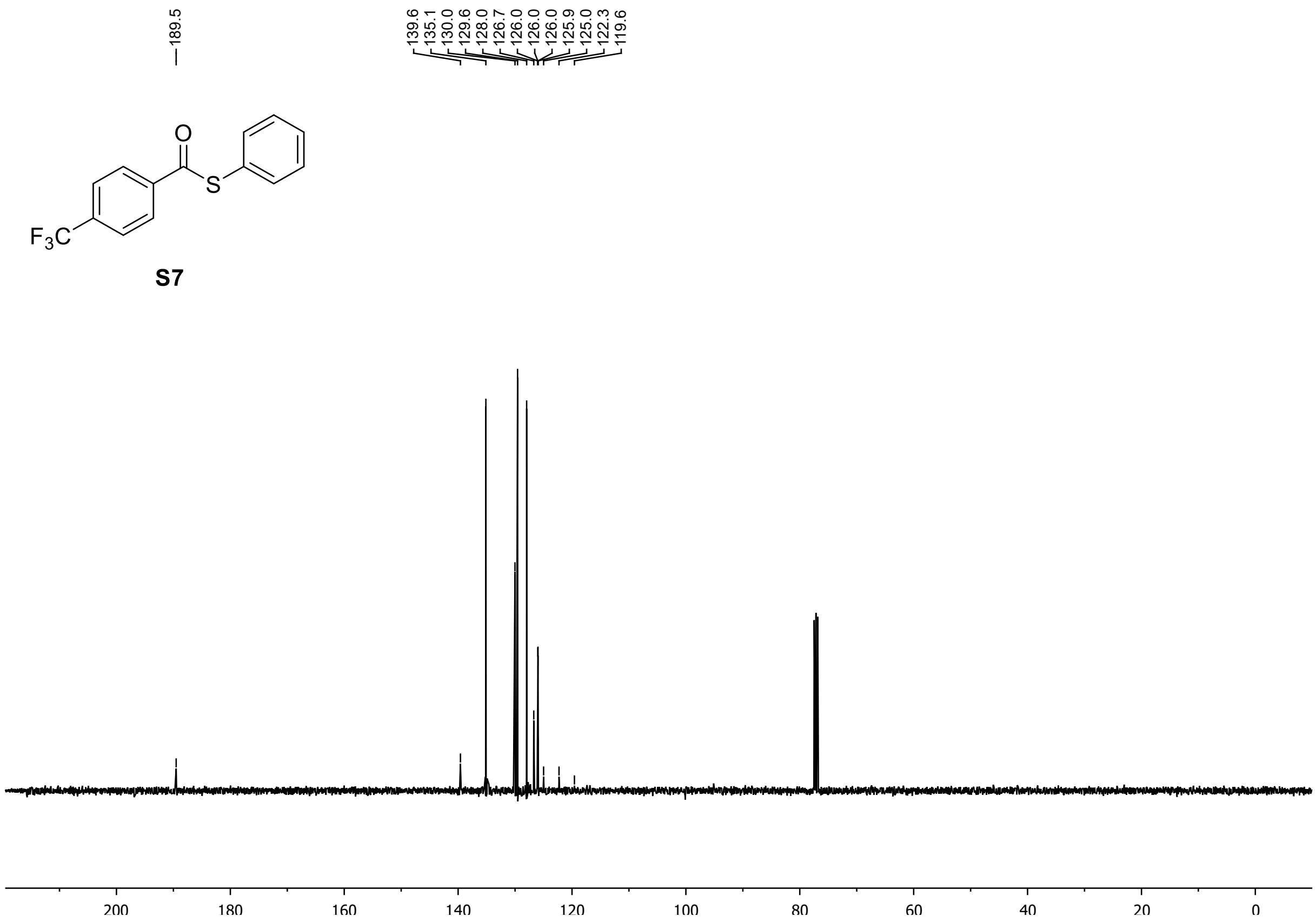

Figure S30. ^{13}C NMR spectrum (101 MHz, $CDCl_3$) of **S7**.

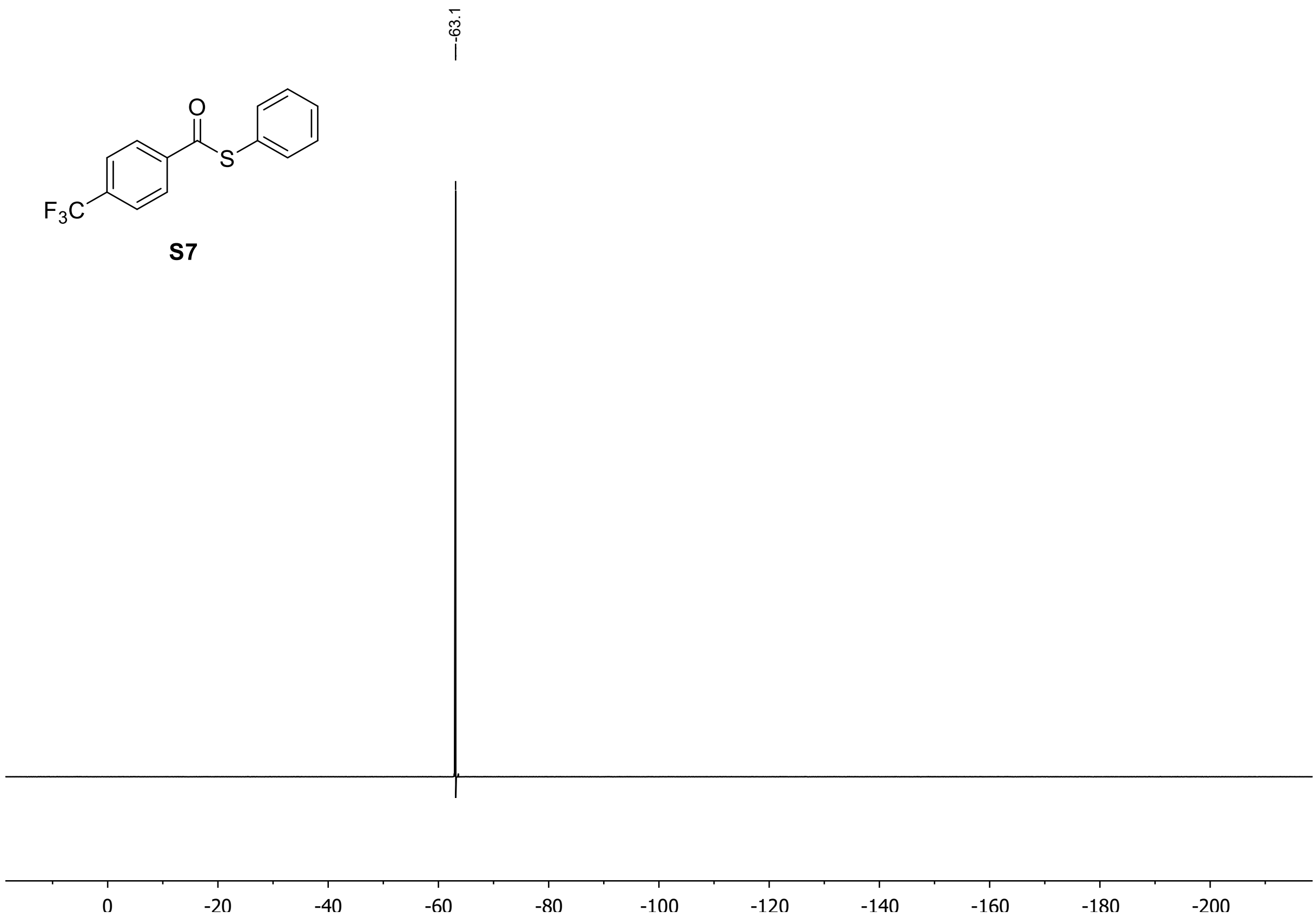

Figure S31. ^{19}F NMR spectrum (376 MHz, $CDCl_3$) of **S7**.

***S*-phenyl 4-methoxybenzothioate (S8):**

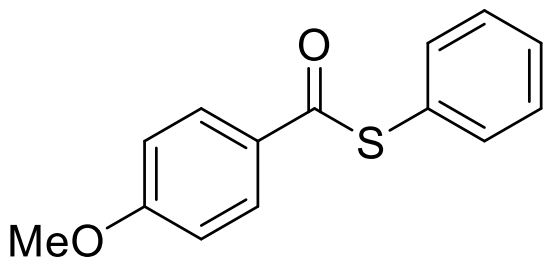

S8

$C_{14}H_{12}O_2S$ (244.31 g/mol)

Following **GP-A1**, **S8** was synthesized using methyl 4-(chlorocarbonyl)benzoate (2.74 mL, 20.0 mmol, 1.0 equiv.), thiophenol (2.45 mL, 24.0 mmol, 1.2 equiv.) and triethylamine (3.34 mL, 24.0 mmol, 1.2 equiv.). Purification by crystallization (DCM/*n*-hexane) afforded **S8** (4.41 g, 18.0 mmol, 90%) as colorless solid. Conforms to reported analytical data.[6]

^{1}H NMR (400 MHz, $CDCl_3$, δ): 8.07 – 7.97 (m, 2H), 7.56 – 7.49 (m, 2H), 7.49 – 7.39 (m, 3H), 7.01 – 6.92 (m, 2H), 3.88 (s, 3H).

^{13}C{^{1}H} NMR (101 MHz, $CDCl_3$, δ): 188.7, 164.1, 135.3, 129.8, 129.5, 129.5, 129.3, 127.8, 114.0, 55.7.

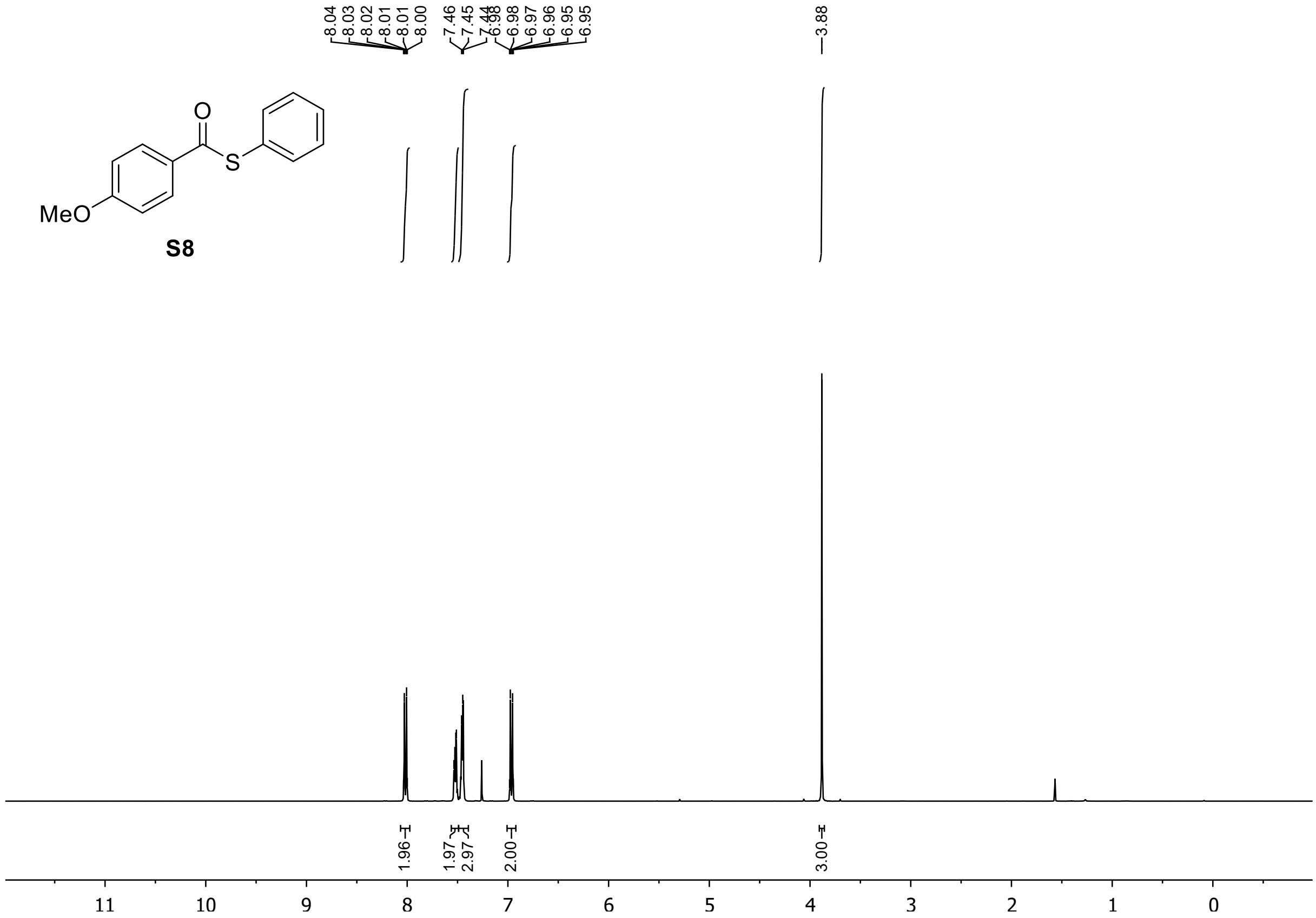

Figure S32. ^{1}H NMR spectrum (400 MHz, $CDCl_3$) of **S8**.

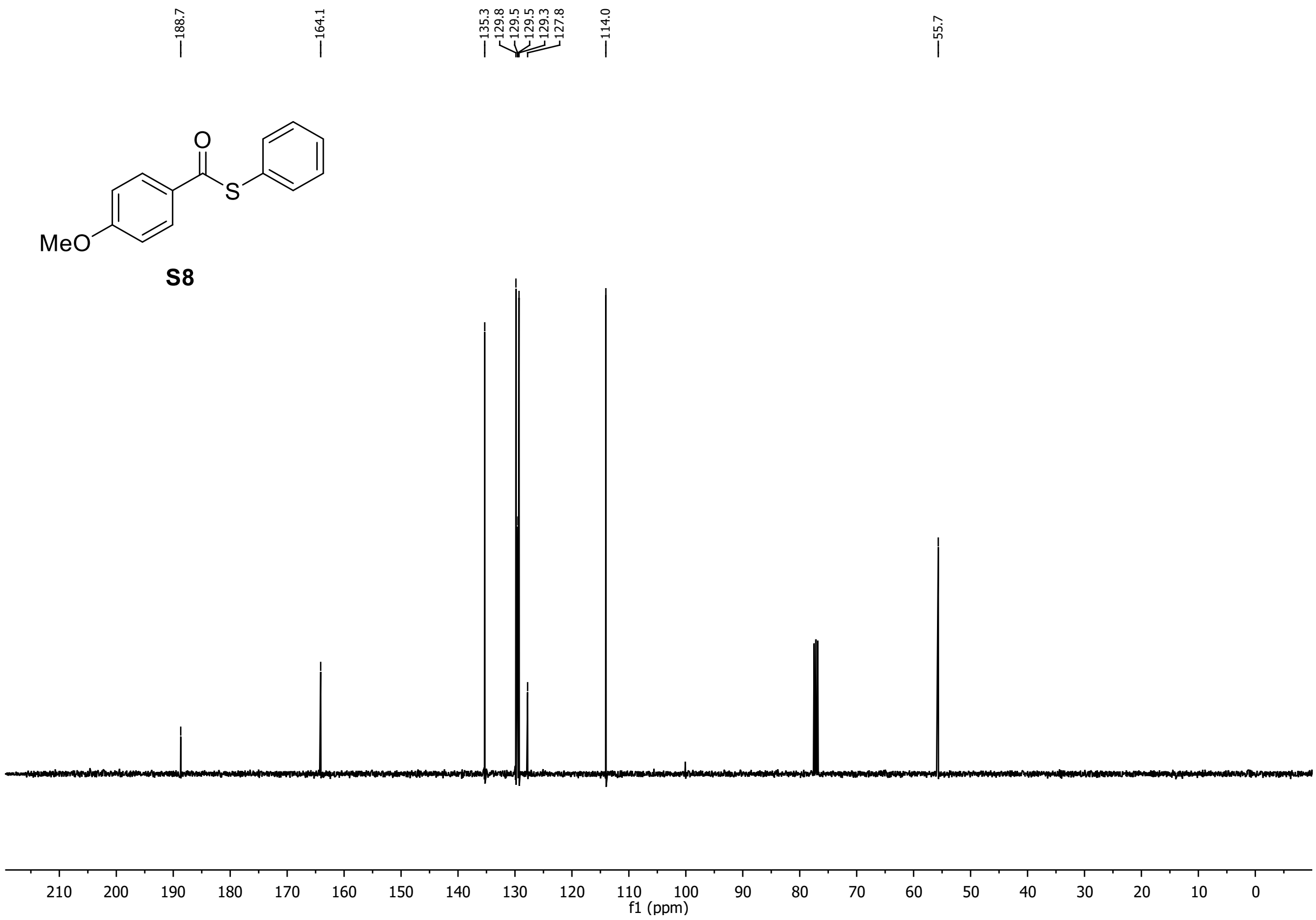

Figure S33. ^{13}C NMR spectrum (101 MHz, $CDCl_3$) of **S8**.

***S*-phenyl benzo*[d]*[1,3]dioxole-5-carbothioate (S9):**

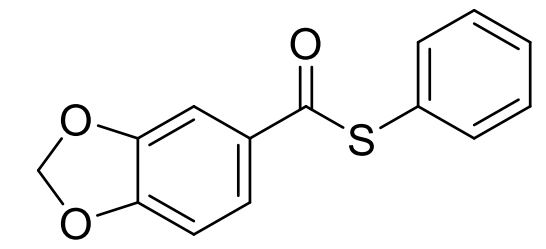

S9

$C_{14}H_{10}O_3S$ (258.29 g/mol)

Following **GP-A1**, **S9** was synthesized using 1,3-benzodioxole-5-carbonyl chloride (1.85 g, 10.0 mmol, 1 equiv.), thiopenol (1.32 g, 12.0 mmol, 1.2 equiv.), and triethylamine (1.21 g, 12 mmol, 1.2 equiv.). Purification by crystallization (DCM/n-hexane) afforded **1g** (863 mg, 3.34 mmol, 33%) as colorless solid. Conforms to reported analytical data.[10]

^{1}H NMR (400 MHz, $CDCl_3$, δ): 7.69 (dd, J = 8.2, 1.8 Hz, 1H), 7.53 – 7.48 (m, 2H), 7.48 – 7.42 (m, 4H), 6.88 (d, J = 8.2 Hz, 1H), 6.07 (s, 2H).

^{13}C{^{1}H} NMR (101 MHz, $CDCl_3$, δ): 188.5, 152.4, 148.3, 135.3, 131.2, 129.6, 129.3, 127.6, 123.8, 108.3, 107.6, 102.2.

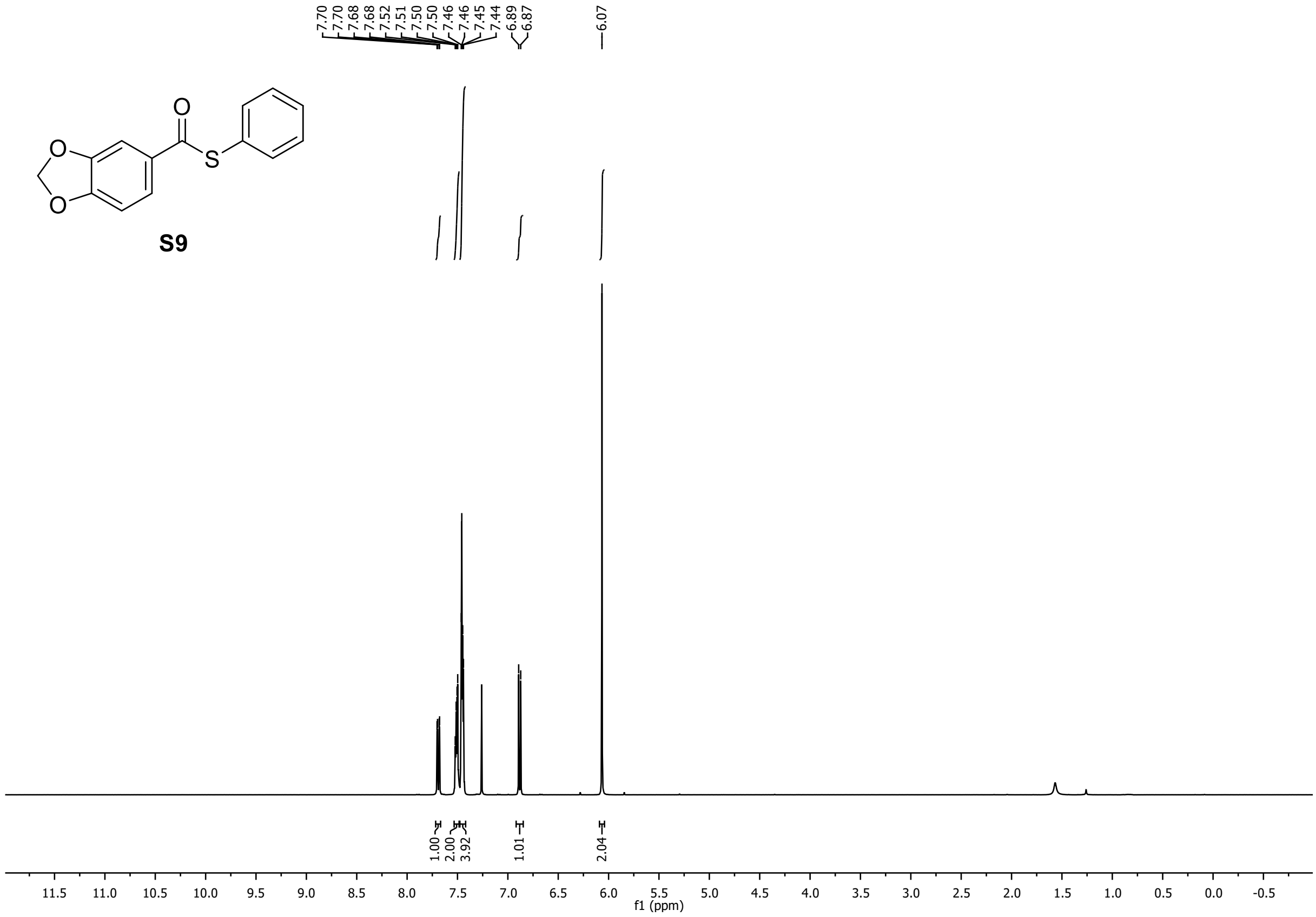

Figure S34. ^{1}H NMR spectrum (400 MHz, $CDCl_3$) of **S9**.

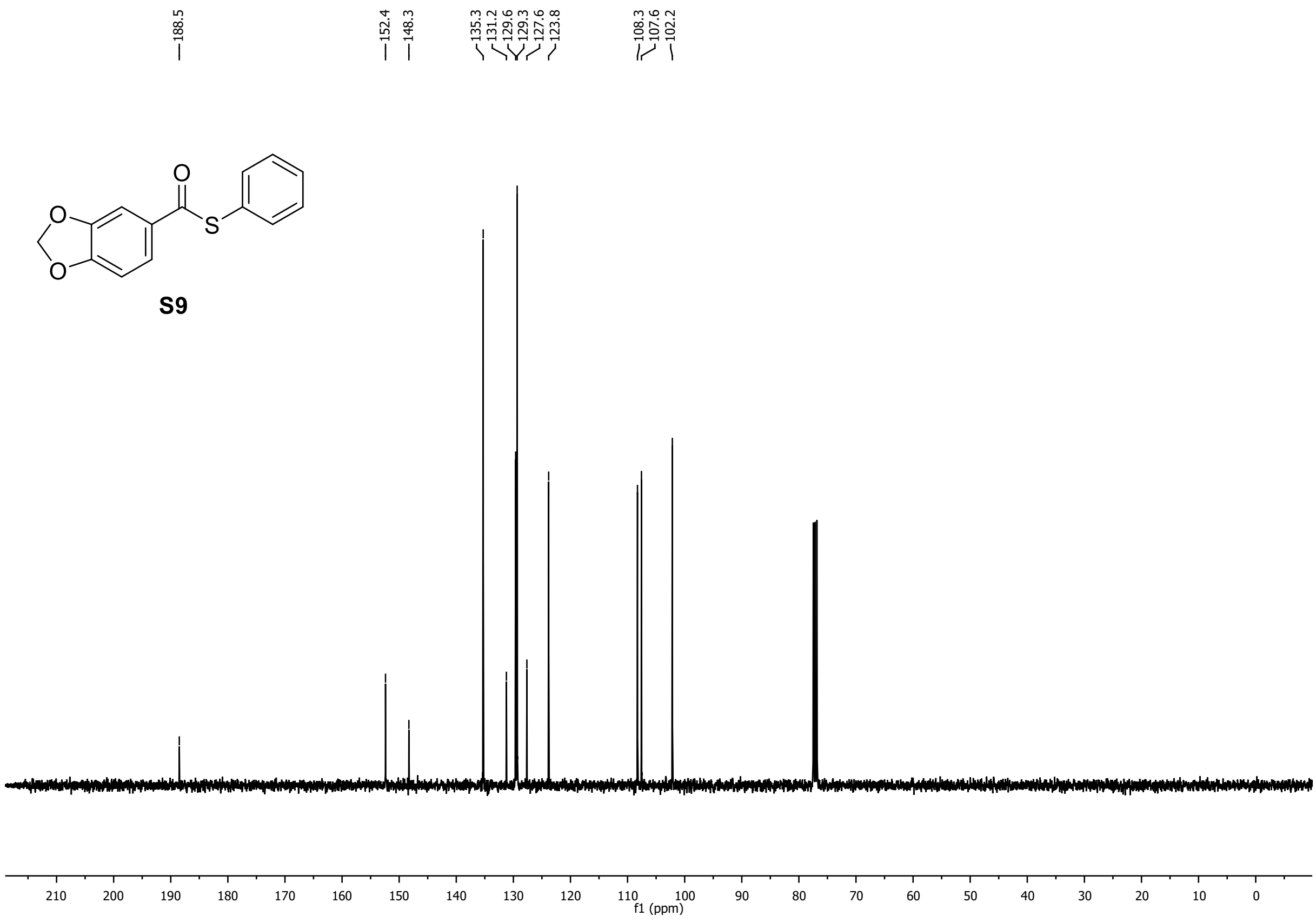

Figure S35. ^{13}C NMR spectrum (101 MHz, $CDCl_3$) of **S9**.

***S*-Phenyl 6-chloropyridine-3-carbothioate (S10):**

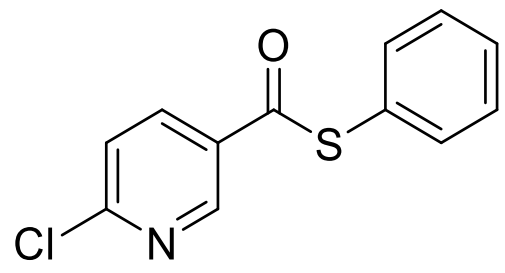

S10

$C_{12}H_8ClNOS$ (249.71 g/mol)

Following **GP-1**, **S10** was synthesized using 6-chloronicotinoyl chloride (3.52 g, 20.0 mmol, 1.0 equiv.), thiophenol (2.20 mL, 21.0 mmol, 1.05 equiv.) and triethylamine (3.35 mL, 24.0 mmol, 1.05 equiv.). Purification by crystallization (DCM/*n*-hexane) afforded **S10** (3.34 g, 13.4 mmol, 67%) as colorless solid. Conforms to reported analytical data.[11]

^{1}H NMR (400 MHz, $CDCl_3$, δ): 9.02 (d, *J* = 2.4 Hz, 1H), 8.21 (dd, *J* = 8.4, 2.4 Hz, 1H), 7.53 – 7.45 (m, 6H).

^{13}C{^{1}H} NMR (101 MHz, $CDCl_3$, δ): 187.9, 156.3, 149.0, 137.4, 135.1, 131.3, 130.2, 129.6, 126.0, 124.7.

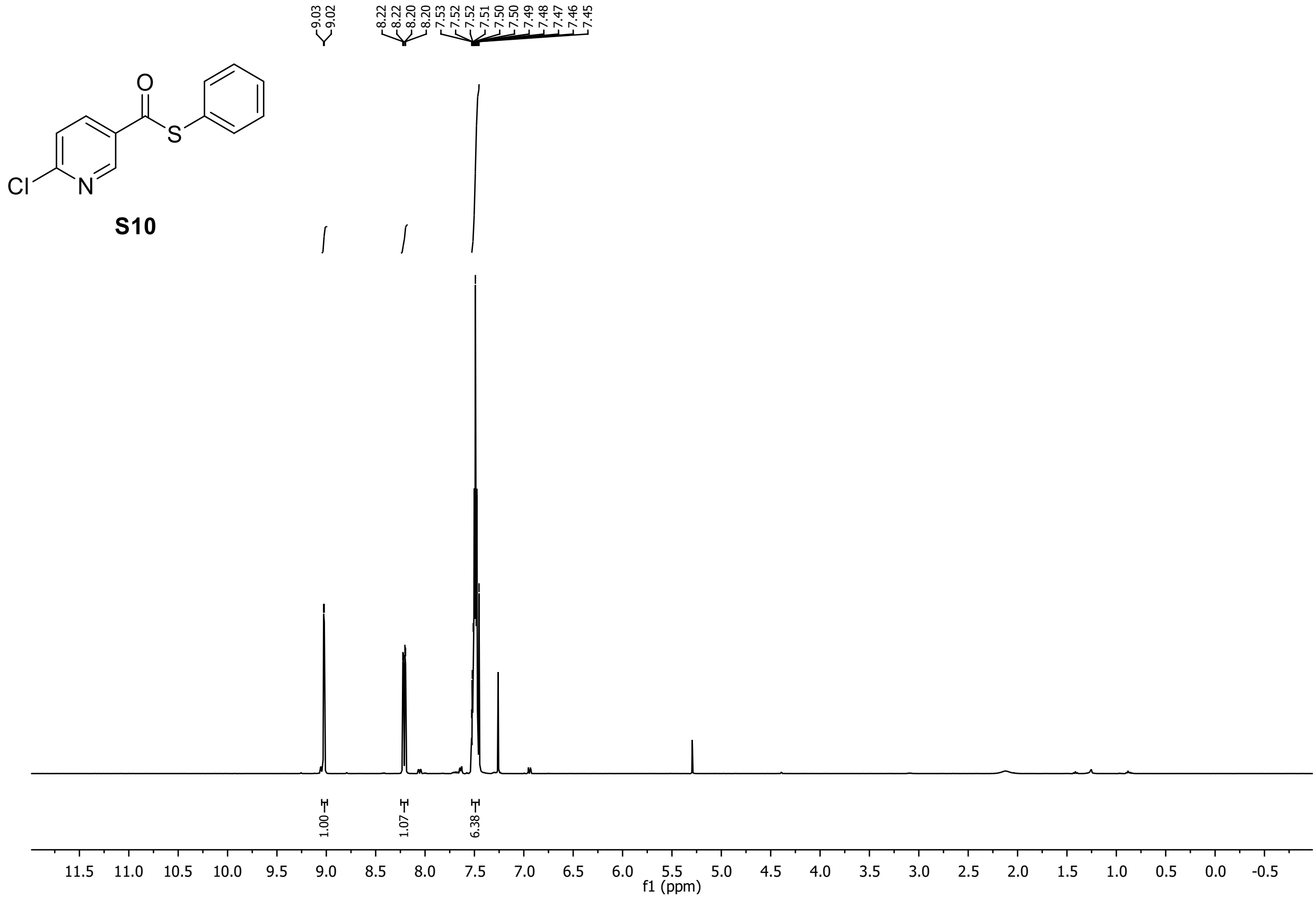

Figure S36. ^{1}H NMR spectrum (400 MHz, $CDCl_3$) of **S10**.

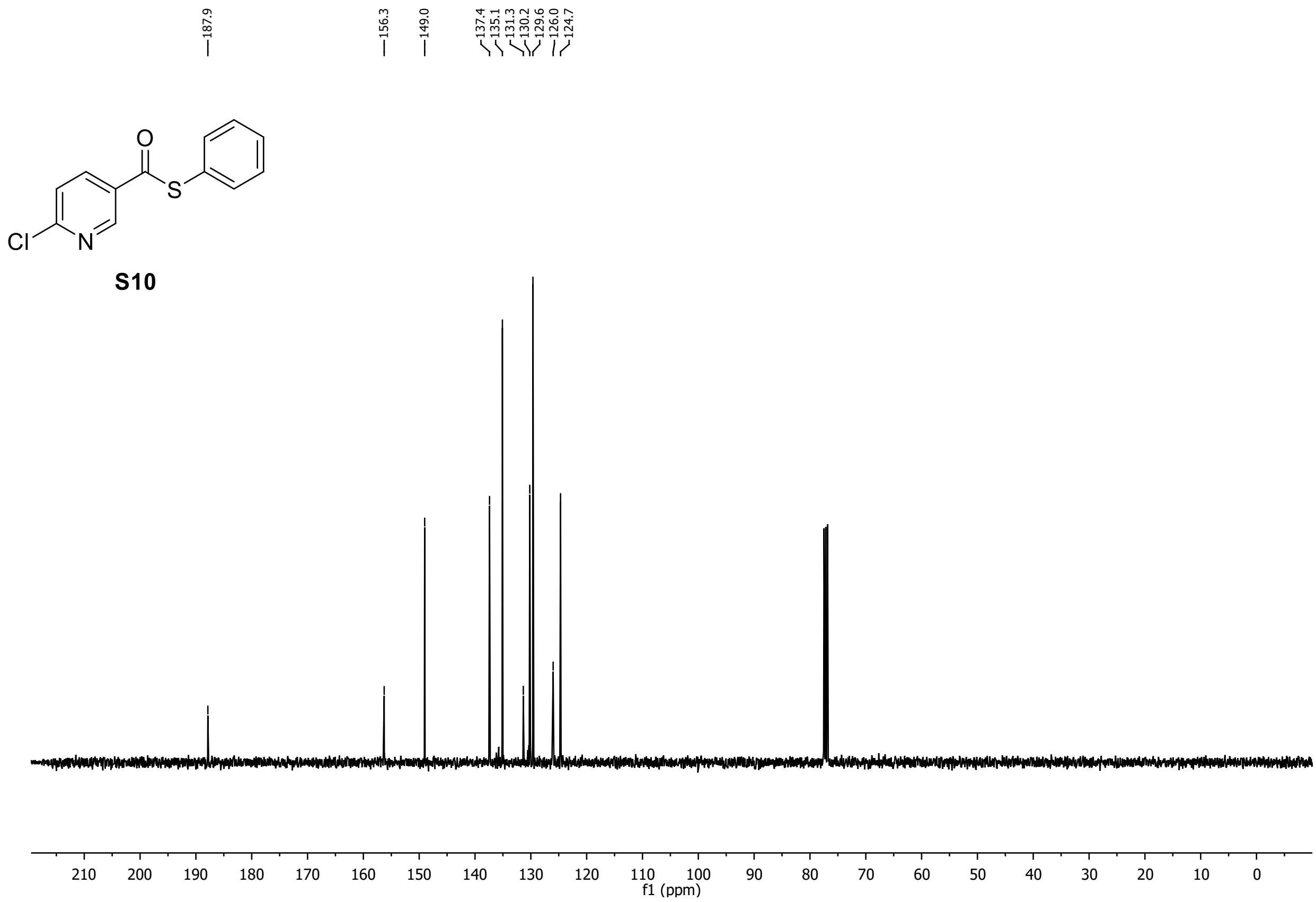

Figure S37. ^{13}C NMR spectrum (101 MHz, $CDCl_3$) of **S10**.

S-Phenyl hexanethioate (S11):

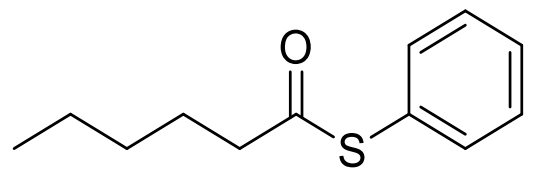

S11

$C_{12}H_{16}OS$ (208.32 g/mol)

Following **GP-A1**, **S11** was synthesized using hexanoyl chloride (1.39 g, 10.0 mmol, 1.0 equiv.), thiophenol (1.23 mL, 12.0 mmol, 1.2 equiv.) and triethylamine (1.39 mL, 10.0 mmol, 1.0 equiv.). Purification by column chromatography (*n*-hexane/EtOAc, gradient 100:0 to 95:5 over 10 CV) afforded **S11** (0.89 g, 4.27 mmol, 43%) as colorless oil. Conforms to reported analytical data.[10]

^{1}H NMR (400 MHz, $CDCl_3$, δ): 7.47 – 7.37 (m, 5H), 2.65 (t, *J* = 7.5 Hz, 2H), 1.78 – 1.66 (m, 2H), 1.42 – 1.27 (m, 4H), 0.96 – 0.86 (m, 3H).

^{13}C{^{1}H} NMR (101 MHz, $CDCl_3$, δ): 197.7, 134.6, 129.4, 129.3, 128.1, 43.8, 31.2, 25.4, 22.5, 14.0.

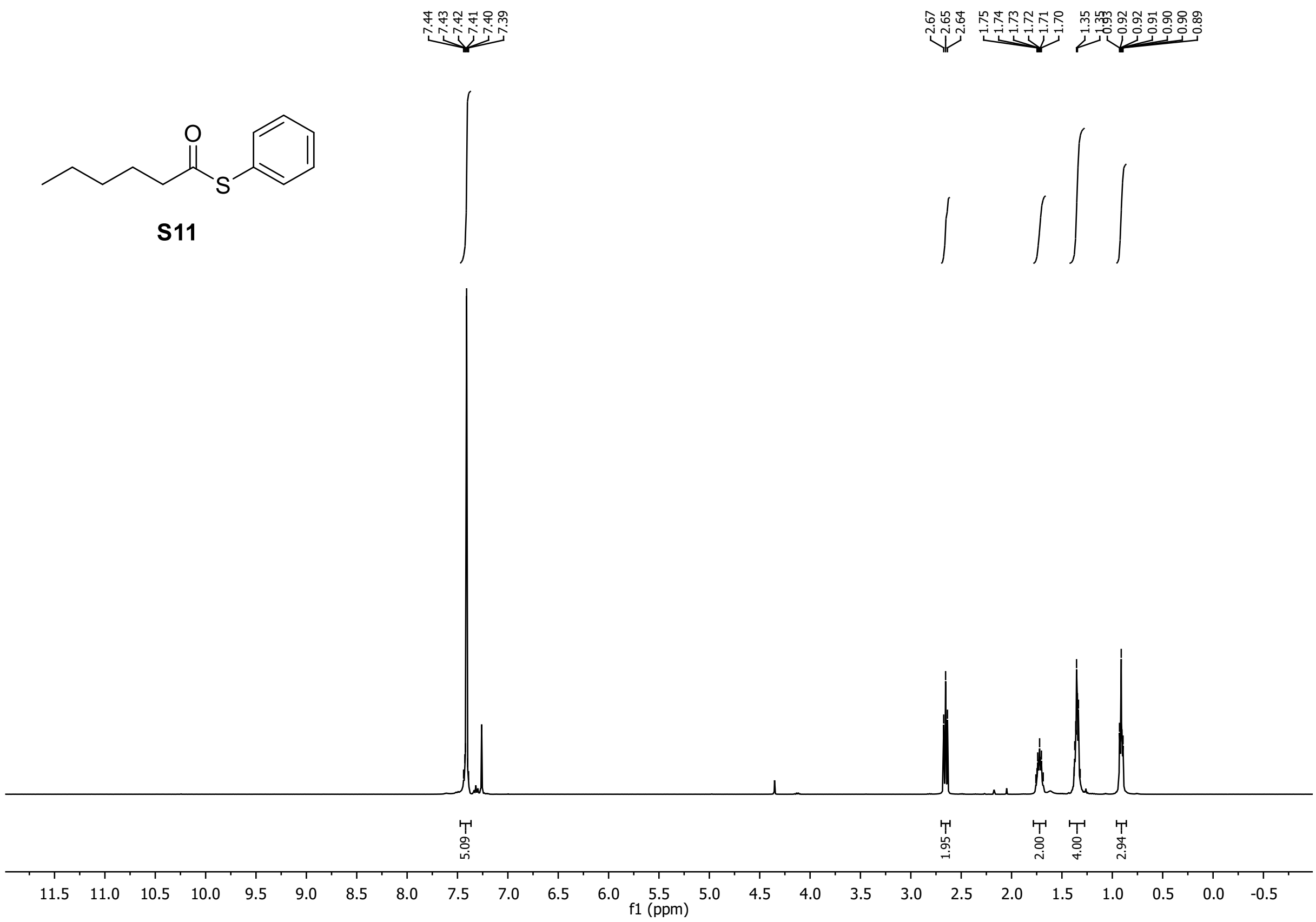

Figure S38. ^{1}H NMR spectrum (400 MHz, $CDCl_3$) of **S11**.

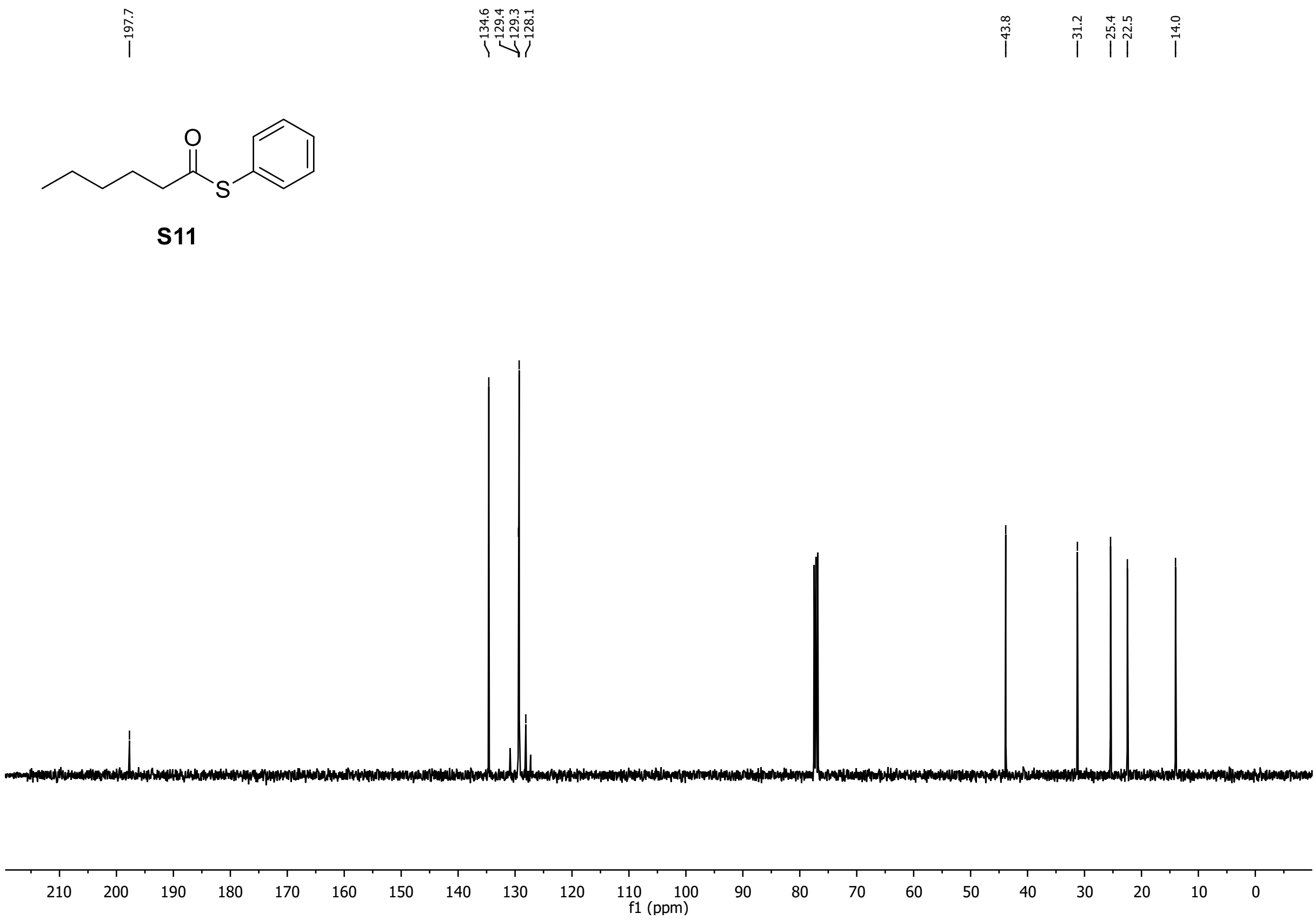

Figure S39. ^{13}C NMR spectrum (101 MHz, $CDCl_3$) of **S11**.

S-Phenyl pent-4-enethioate (S12):

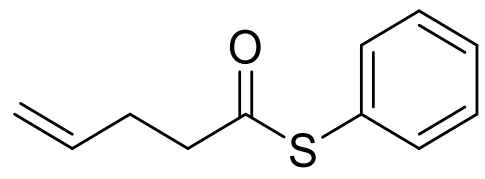

S12

$C_{11}H_{12}OS$ (192.28 g/mol)

Following **GP-A1**, **S12** was synthesized using 4-pentenoyl chloride (1.19 g, 10.0mmol, 1 equiv.), thiophenol (1.32 g, 12.0 mmol, 1.2 equiv.), and triethylamine (1.21 g, 12.0 mmol, 1.2 equiv.). Removal of the solvent afforded **S12** (1.04 g, 5.39 mmol, 54%) as yellow liquid, which was used as received. Conforms to reported analytical data.[12]

^{1}H NMR (400 MHz, $CDCl_3$, δ): δ 7.42 (m, 5H), 5.93 – 5.78 (m, 1H), 5.16 – 5.04 (m, 2H), 2.82 – 2.73 (m, 2H), 2.53 – 2.43 (m, 2H).

^{13}C{^{1}H} NMR (101 MHz, $CDCl_3$, δ): 196.8, 136.1, 134.6, 129.5, 129.3, 127.6, 116.1, 42.9, 29.4.

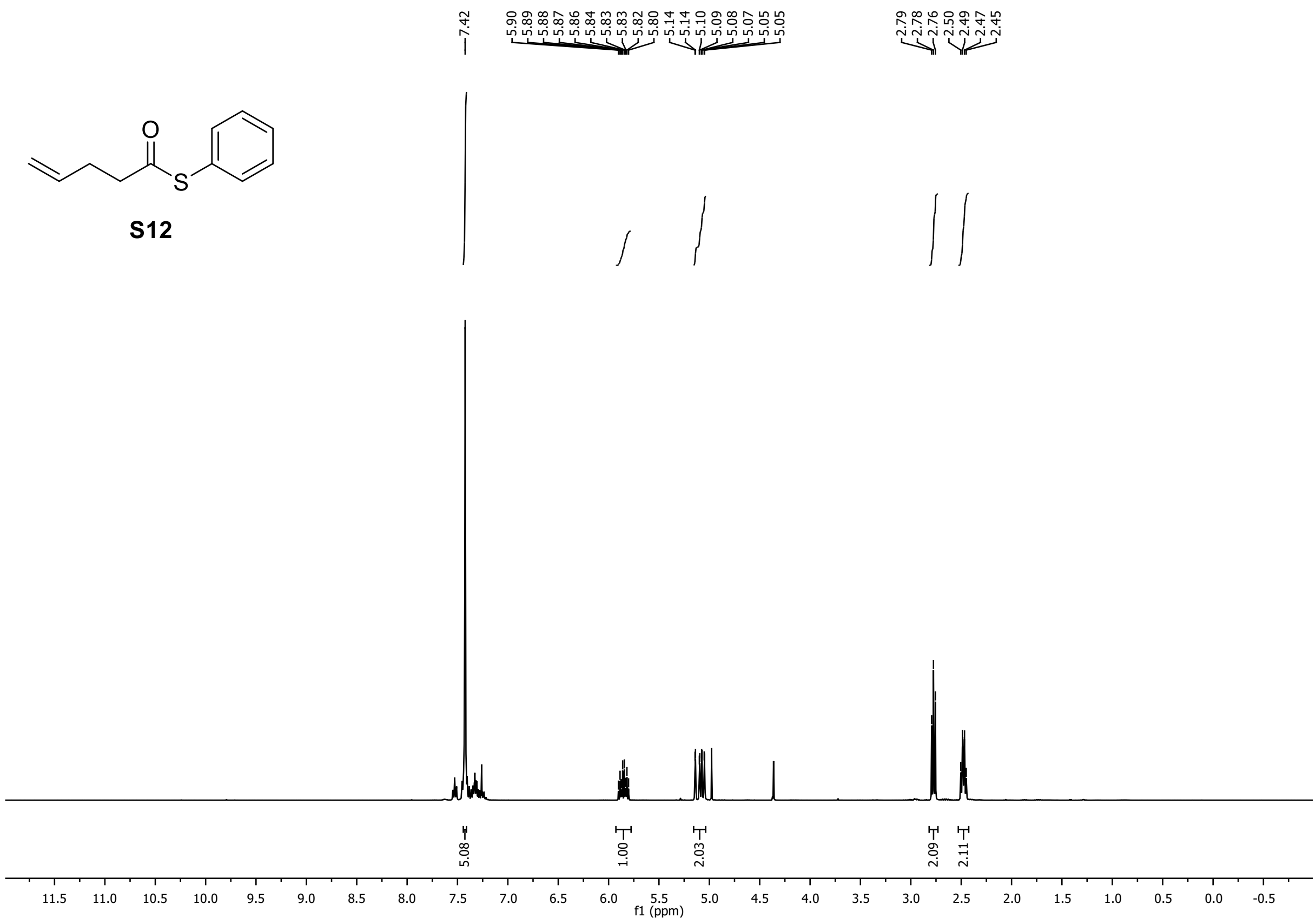

Figure S40. ^{1}H NMR spectrum (400 MHz, $CDCl_3$) of **S12**.

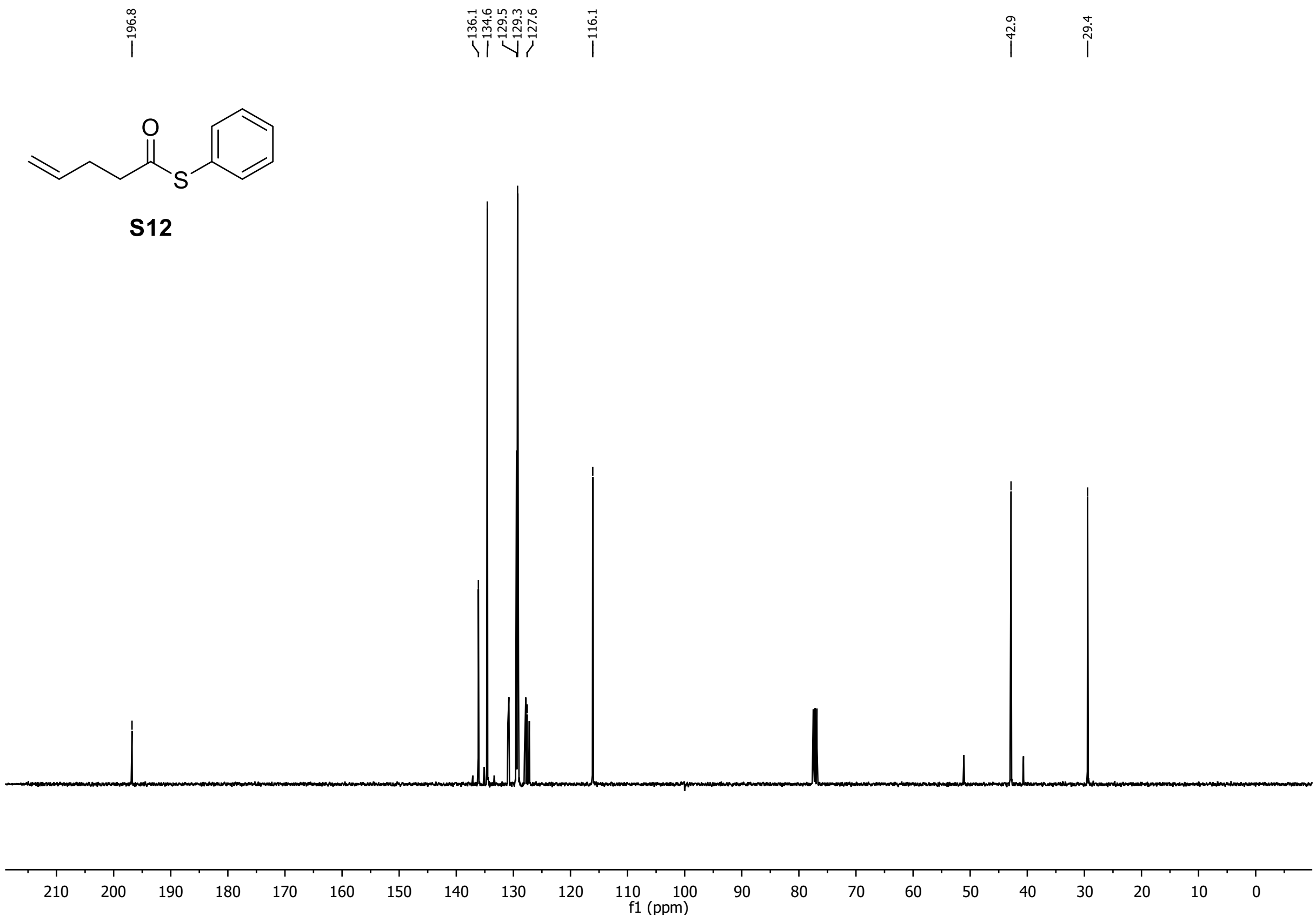

Figure S41. ^{13}C NMR spectrum (101 MHz, $CDCl_3$) of **S12**.

***S*-Phenyl cyclopropanecarbothioate (S13):**

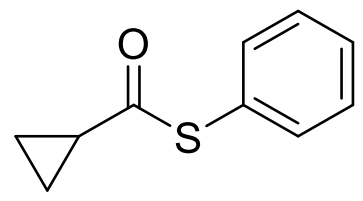

S13

$C_{10}H_{10}OS$ (178.25 g/mol)

Following **GP-A1**, **S13** was synthesized using cyclopropanecarbonyl chloride (1.81 mL, 20.0 mmol, 1.0 equiv.), thiophenol (2.04 mL, 20.0 mmol, 1.0 equiv.) and triethylamine (2.78 mL, 20.0 mmol, 1.0 equiv.). Purification by crystallization at low temperature (-20 °C, DCM/*n*-hexane) afforded **S13** (2.51 g, 14.1 mmol, 84%) as colorless oil. Conforms to reported analytical data.[11]

^{1}H NMR (400 MHz, $CDCl_3$, δ): 7.48 – 7.36 (m, 5H), 2.11 (m, 1H), 1.22 (m, 2H), 1.04 – 0.98 (m, 2H).

^{13}C{^{1}H} NMR (101 MHz, $CDCl_3$, δ): 197.6, 134.6, 129.4, 129.2, 128.0, 22.3, 11.3.

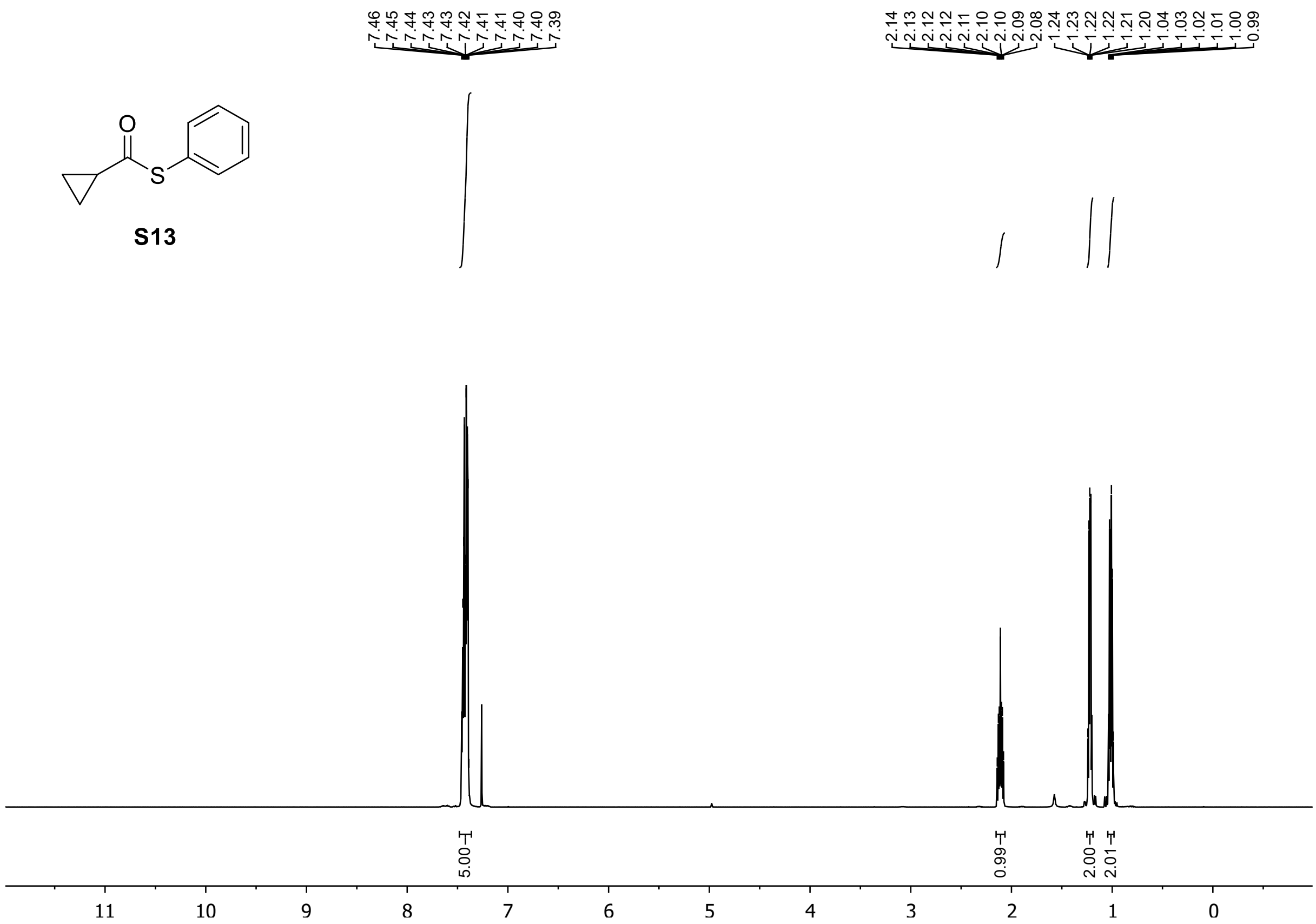

Figure S42. ^{1}H NMR spectrum (400 MHz, $CDCl_3$) of **S13**.

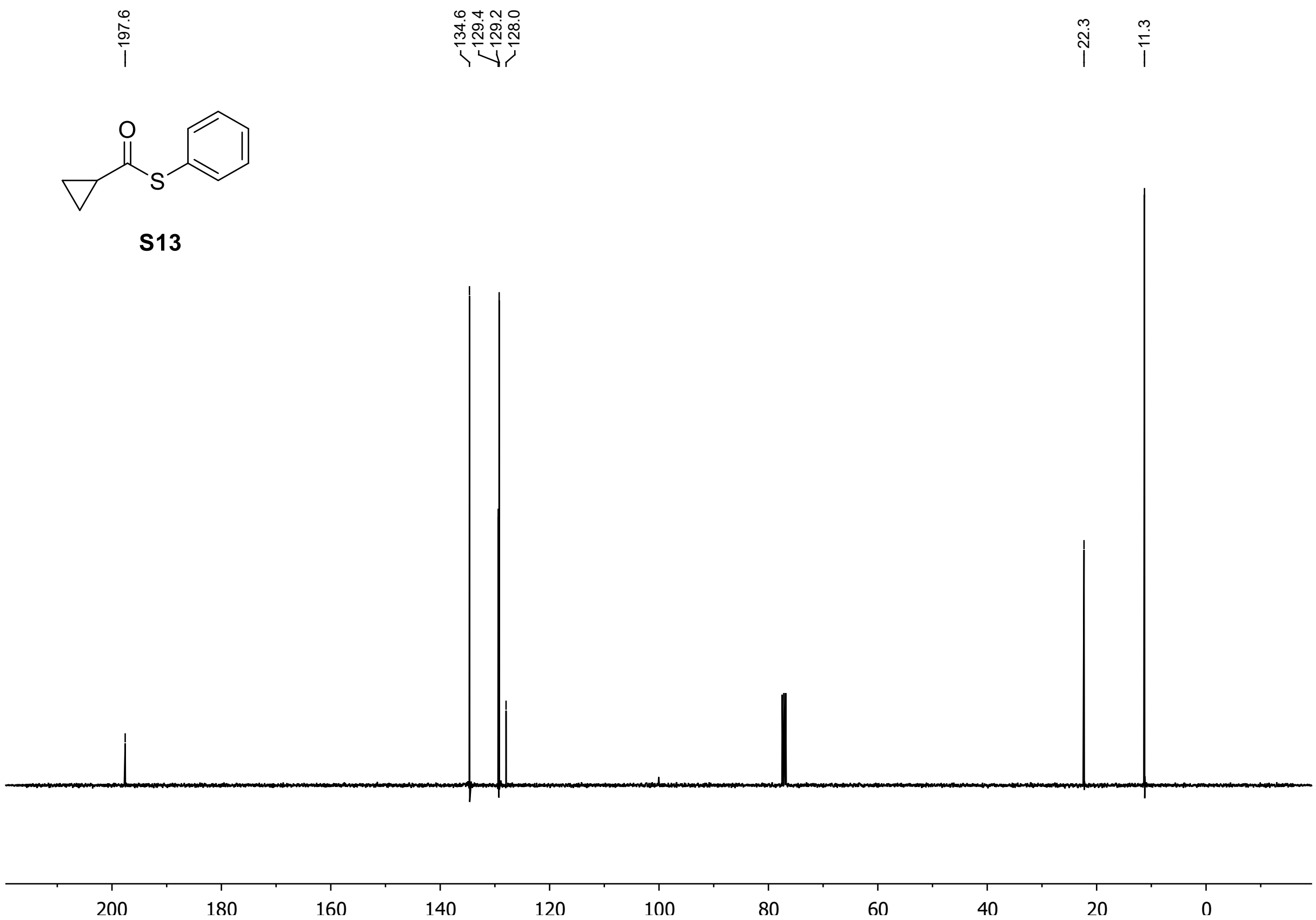

Figure S43. ^{13}C NMR spectrum (101 MHz, $CDCl_3$) of **S13**.

***S*-Phenyl cyclohexanecarbothioate (S14):**

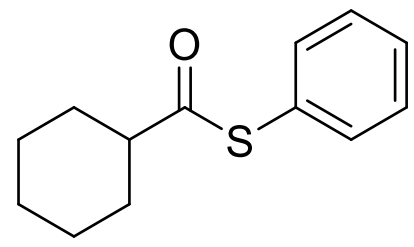

S14

$C_{13}H_{16}OS$ (220.33 g/mol)

Following **GP-A1**, **S14** was synthesized using cyclohexanecarbonyl chloride (2.75 mL, 20.0 mmol, 1.0 equiv.), thiophenol (2.45 mL, 24.0 mmol, 1.2 equiv.) and triethylamine (3.34 mL, 24.0 mmol, 1.2 equiv.). Purification by crystallization (DCM/*n*-hexane) afforded **S14** (2.64 g, 12.0 mmol, 60%) as colorless solid. Conforms to reported analytical data.[6]

^{1}H NMR (400 MHz, $CDCl_3$, δ): 7.40 (m, 5H), 2.61 (m, 1H), 2.07 – 1.95 (m, 2H), 1.88 – 1.76 (m, 2H), 1.73 – 1.62 (m, 1H), 1.53 (m, 2H), 1.40 – 1.16 (m, 3H).

^{13}C{^{1}H} NMR (101 MHz, $CDCl_3$, δ): 200.9, 134.7, 129.3, 129.2, 128.1, 52.7, 29.7, 25.7, 25.6.

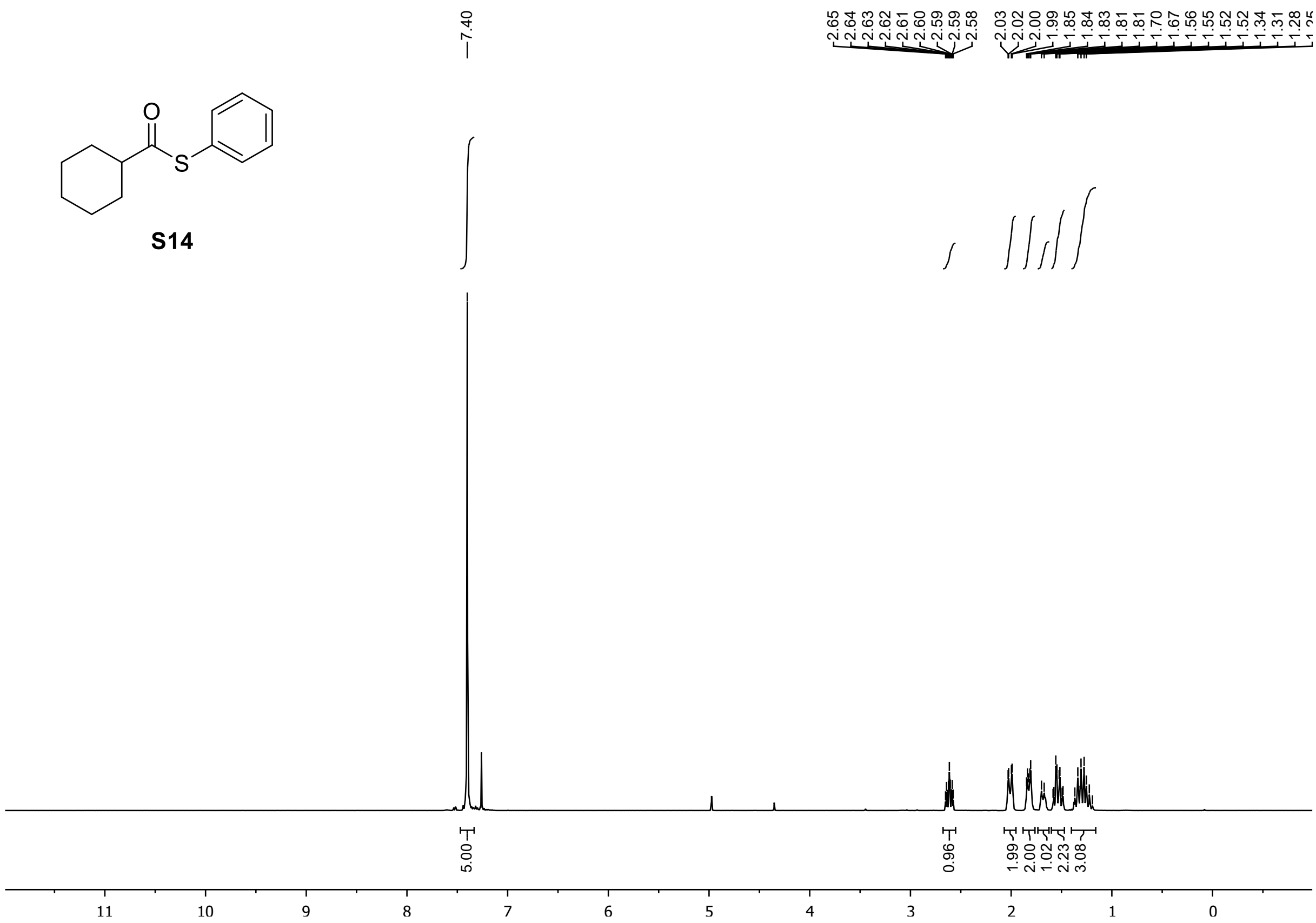

Figure S44. ^{1}H NMR spectrum (400 MHz, $CDCl_3$) of **S14**.

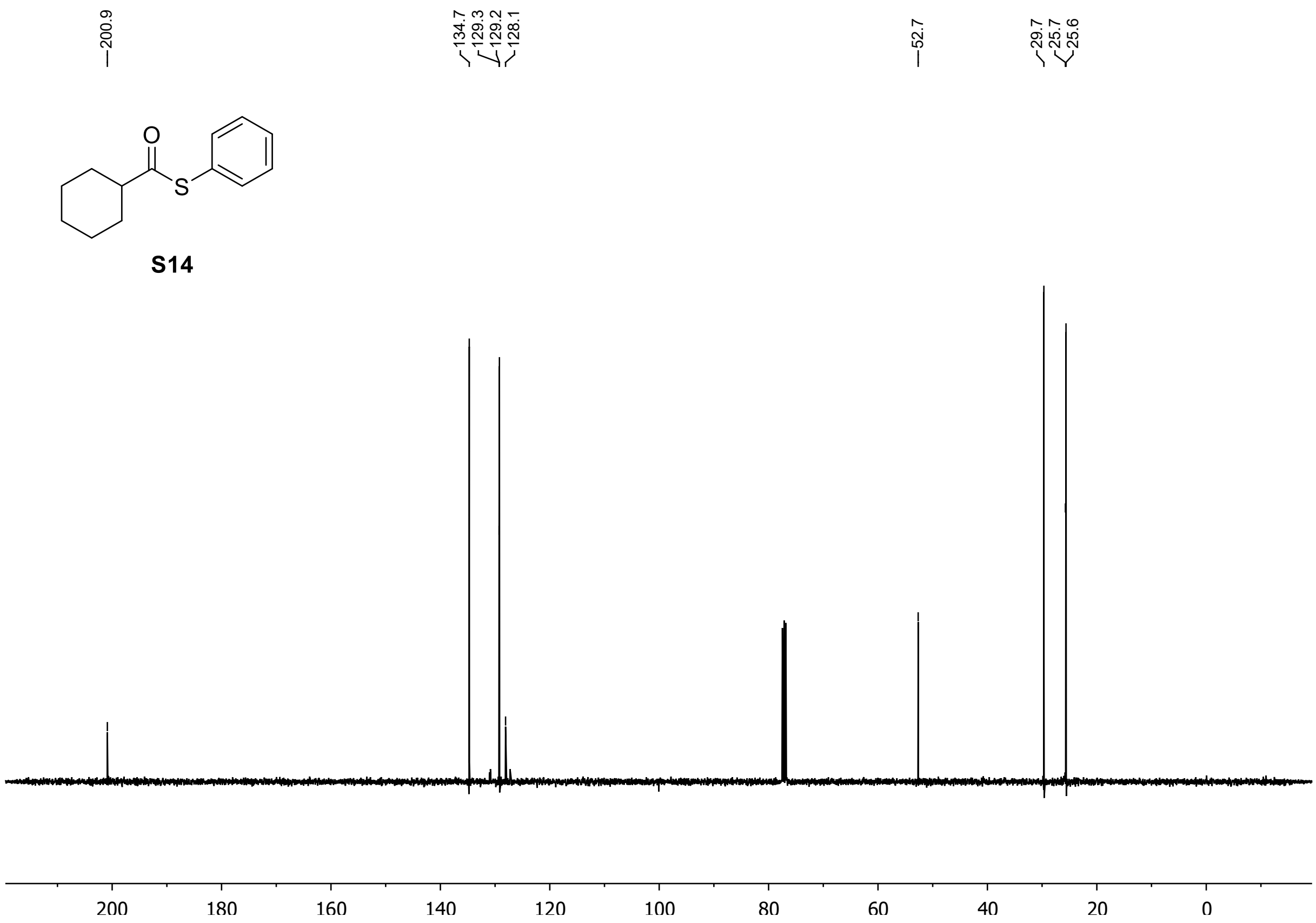

Figure S45. ^{13}C NMR spectrum (101 MHz, $CDCl_3$) of **S14**.

***S*–Phenyl tetrahydro-2H-pyran-4-carbothioate (S15):**

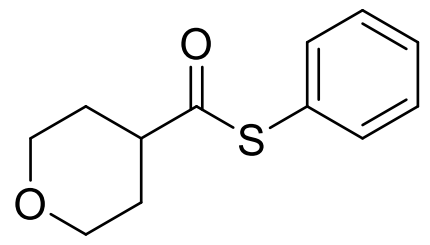

S15

$C_{12}H_{14}O_2S$ (222.3 g/mol)

Following **GP-A1**, **S15** was synthesized using tetrahydro-2*H*-pyran-4-carbonyl chloride (1.52 g, 10.0 mmol, 1 equiv.), thiophenol (1.32 g, 12.0 mmol, 1.2 equiv.), and triethylamine (1.21 g, 12.0 mmol, 1.2 equiv.). Removal of the solvent afforded **S15** (902 mg, 4.06 µmol, 41%) as colorless solid, which was used as received. Conforms to reported analytical data. [Liang, 2023]

^{1}H NMR (400 MHz, $CDCl_3$, δ): δ 7.41 (s, 5H), 4.08 – 3.98 (m, 2H), 3.52 – 3.40 (m, 2H), 2.92 – 2.79 (m, 1H), 1.97 – 1.85 (m, 4H).

^{13}C{^{1}H} NMR (101 MHz, $CDCl_3$, δ): 199.4, 134.7, 129.6, 129.3, 127.4, 67.2, 49.3, 29.3.

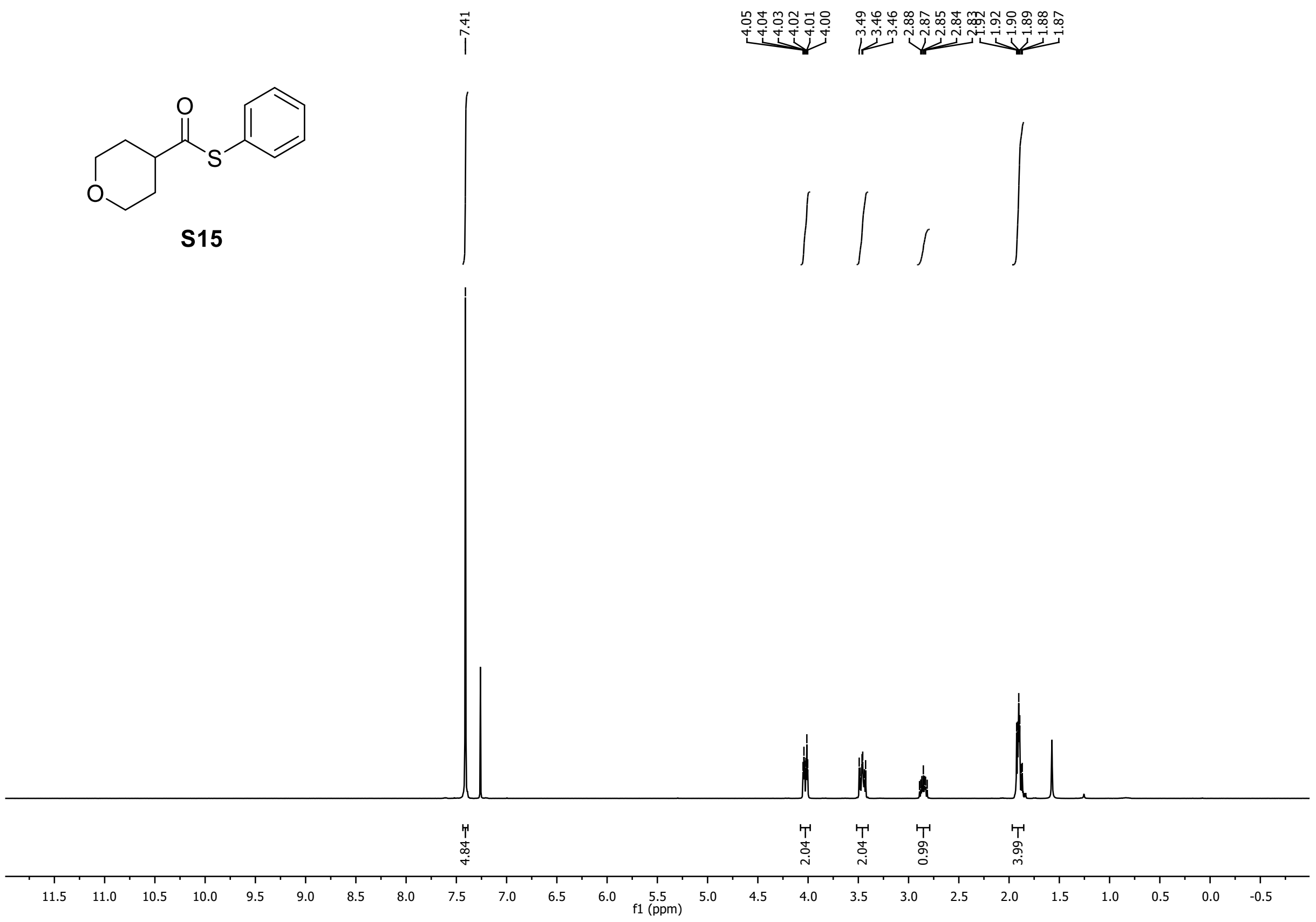

Figure S46. ^{1}H NMR spectrum (400 MHz, $CDCl_3$) of **S15**.

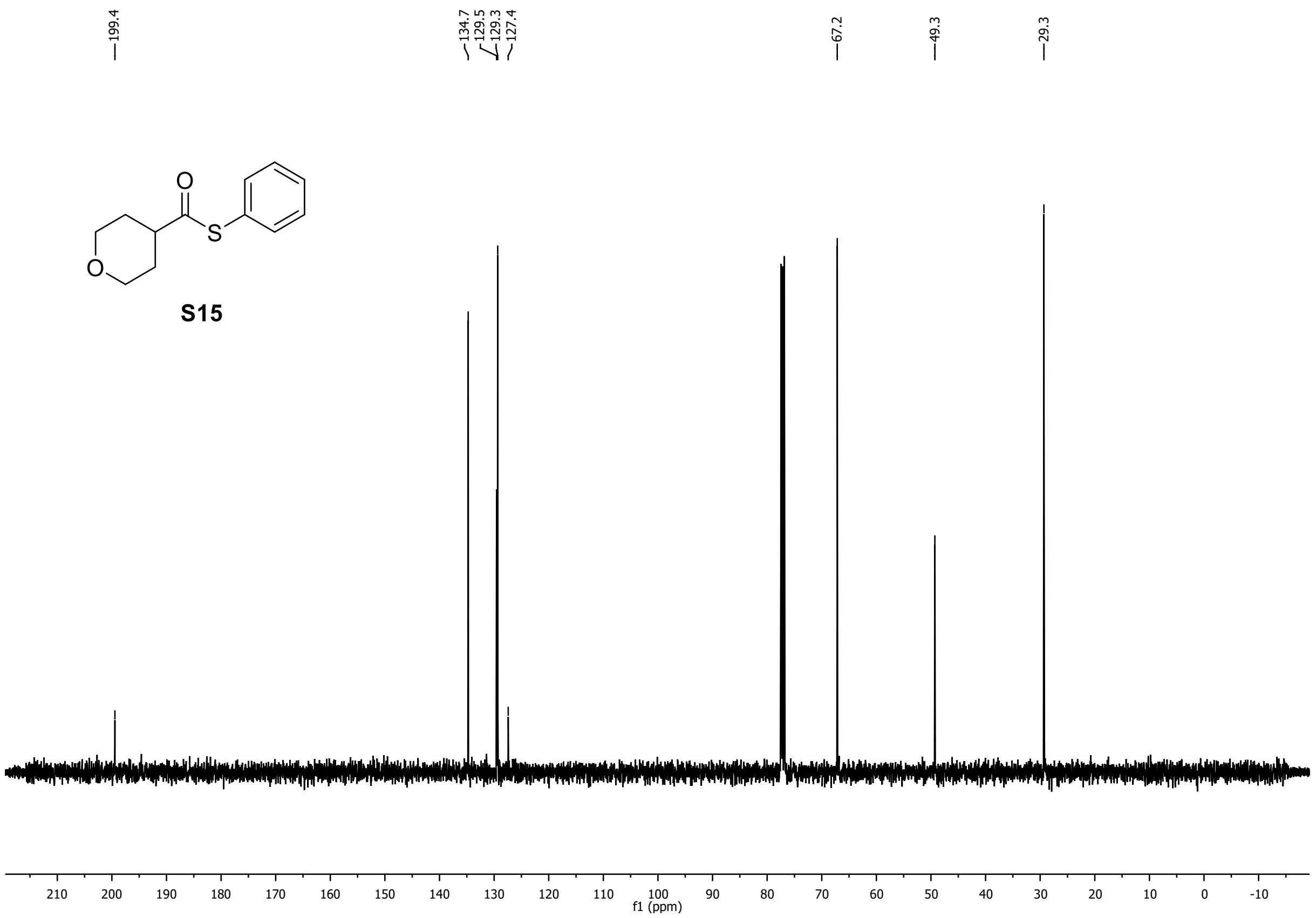

Figure S47. ^{13}C NMR spectrum (101 MHz, $CDCl_3$) of **S15**.

***S*-Phenyl 1-acetylpiperidine-4-carbothioate (S16):**

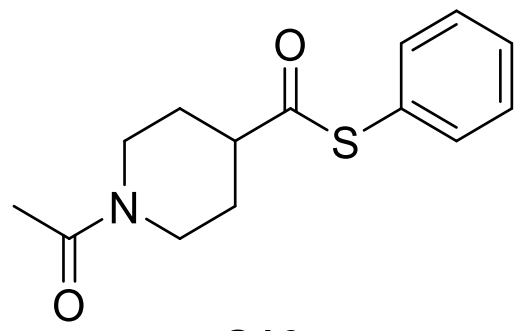

S16

$C_{14}H_{17}NO_2S$ (263.36 g/mol)

Following **GP-A1**, **S16** was synthesized using 1-acetylpiperidine-4-carbonyl chloride (3.79 g, 20.0 mmol, 1.0 equiv.), thiophenol (2.04 mL, 20.0 mmol, 1.0 equiv.) and triethylamine (2.78 mL, 20.0 mmol, 1.0 equiv.). Purification by crystallization (DCM/*n*-hexane) afforded **S16** (2.67 g, 10.2 mmol, 51%) as colorless solid. Conforms to reported analytical data.[13]

^{1}H NMR (400 MHz, $CDCl_3$, δ): 7.44 – 7.36 (m, 5H), 4.53 (m, 1H), 3.85 (m, 1H), 3.27 – 2.99 (m, 1H), 2.84 (m, 2H), 2.10 (s, 3H), 2.01 (m, 2H), 1.87 – 1.58 (m, 2H).

^{13}C{^{1}H} NMR (101 MHz, $CDCl_3$, δ): 199.2, 169.0, 134.7, 129.6, 129.4, 127.2, 49.9, 45.7, 40.9, 28.8, 21.5.

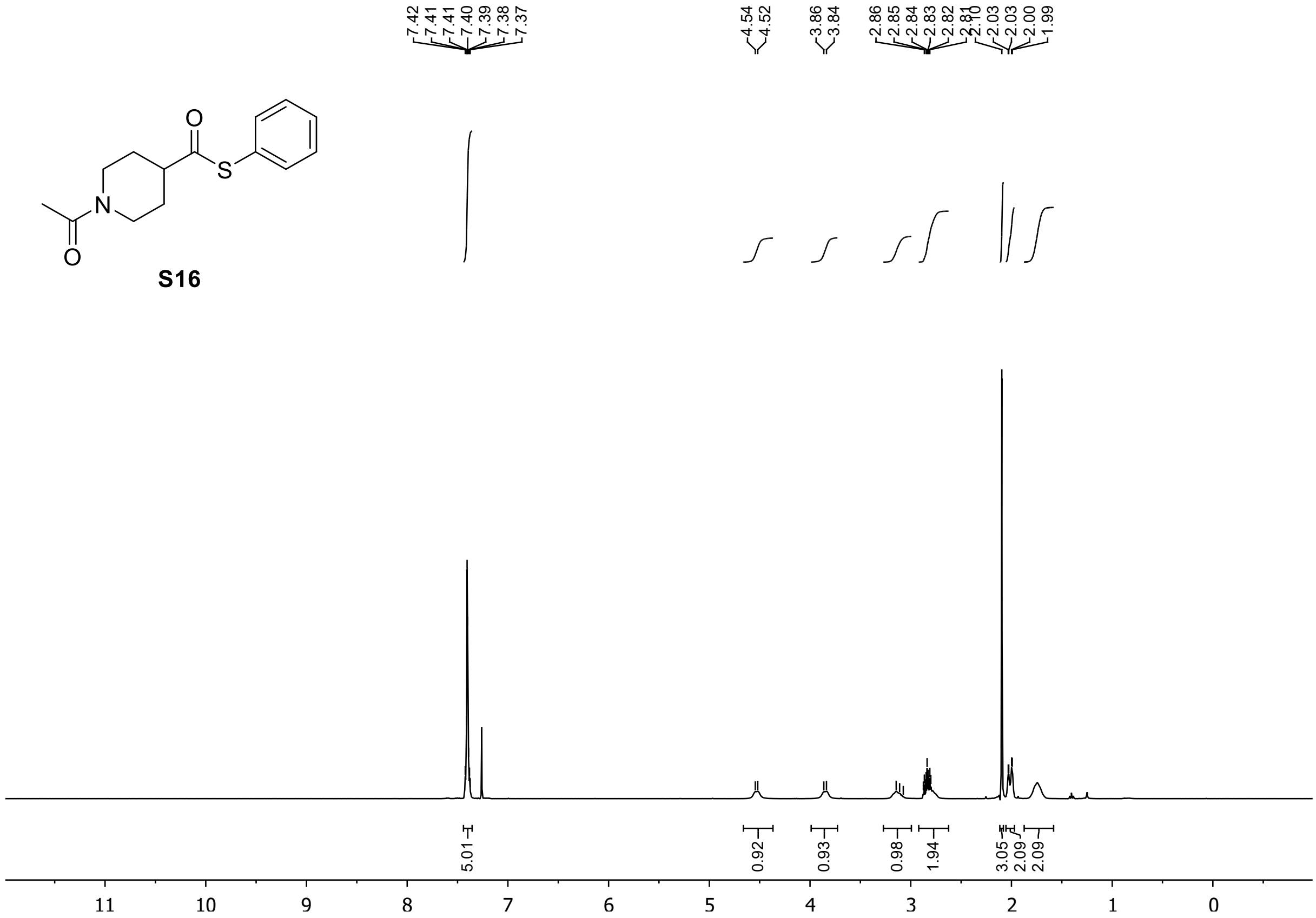

Figure S48. ^{1}H NMR spectrum (400 MHz, $CDCl_3$) of **S16**.

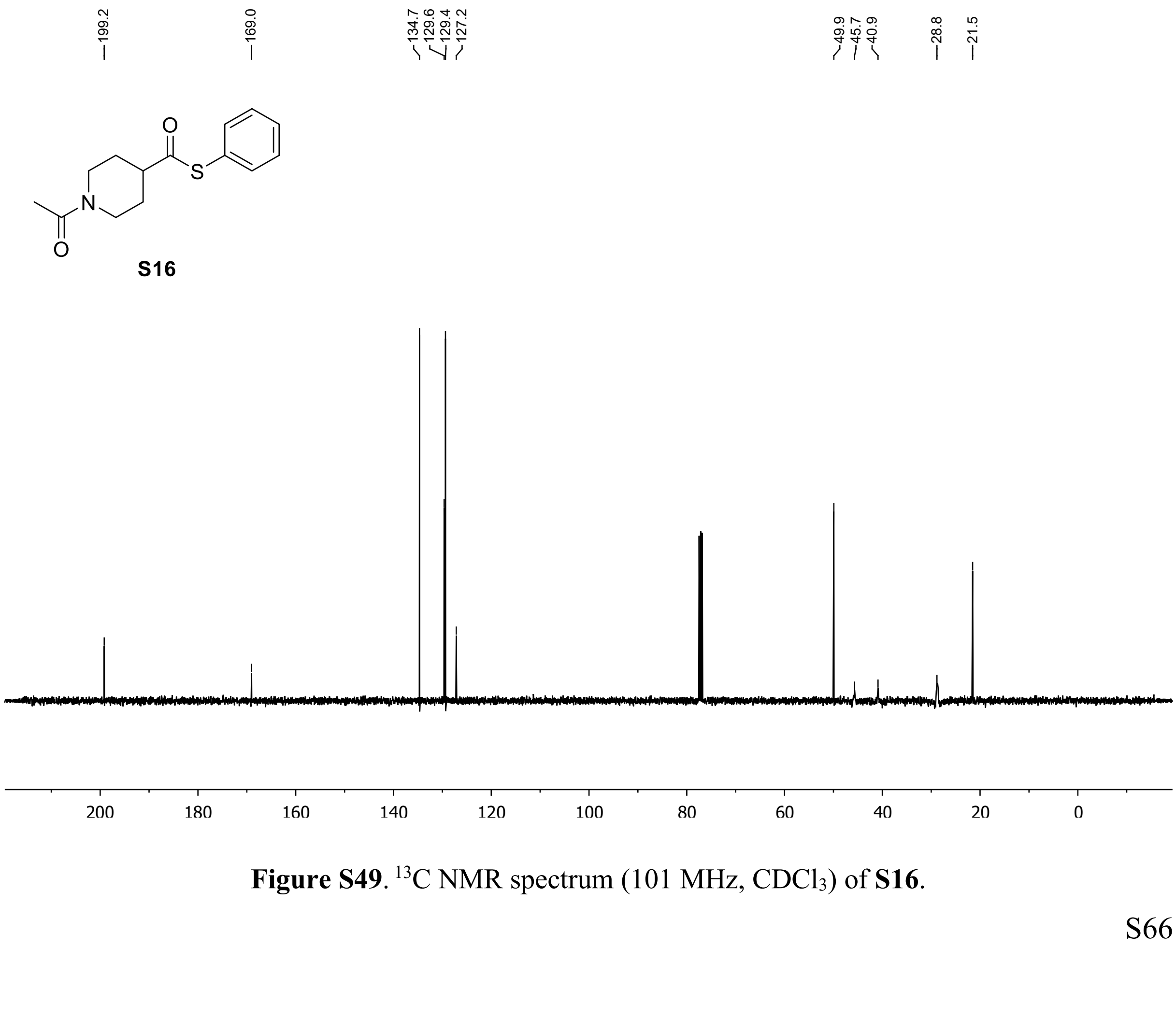

Figure S49. ^{13}C NMR spectrum (101 MHz, $CDCl_3$) of **S16**.

***S*-Phenyl adamantane-1-carbothioate (S17):**

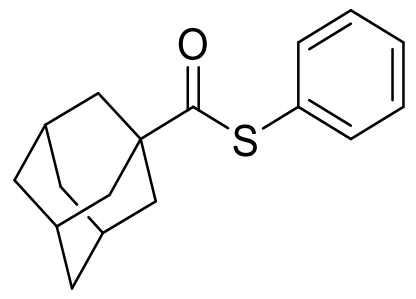

S17

$C_{17}H_{20}OS$ (272.41 g/mol)

Following **GP-A1**, **S17** was synthesized using adamantane-1-carbonyl chloride (3.97 g, 20.0 mmol, 1.0 equiv.), thiophenol (2.04 mL, 20.0 mmol, 1.0 equiv.) and triethylamine (2.78 mL, 20.0 mmol, 1.0 equiv.). Purification by crystallization at low temperature (-20 °C, DCM/*n*-hexane) afforded **S17** (3.63 g, 9.7 mmol, 49%) as colorless liquid (at ambient temperature). Conforms to reported analytical data.[11]

^{1}H NMR (400 MHz, $CDCl_3$, δ): 7.45 – 7.32 (m, 5H), 2.13 – 2.05 (m, 3H), 2.05 – 1.99 (m, 6H), 1.81 – 1.70 (m, 6H).

^{13}C{^{1}H} NMR (101 MHz, $CDCl_3$, δ): 204.3, 135.2, 129.2, 129.1, 128.2, 49.2, 39.4, 36.6, 28.4.

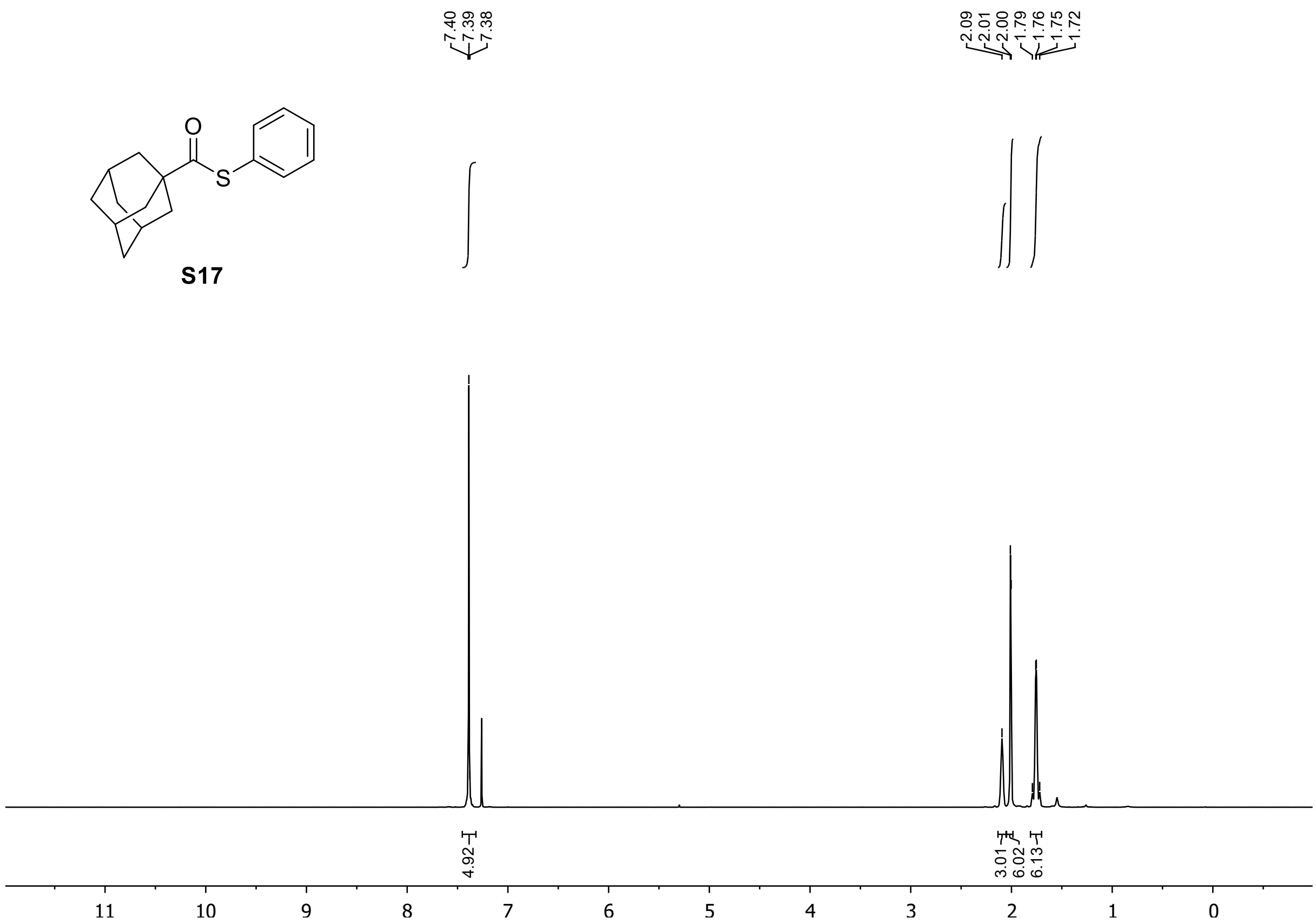

Figure S50. ^{1}H NMR spectrum (400 MHz, $CDCl_3$) of **S17**.

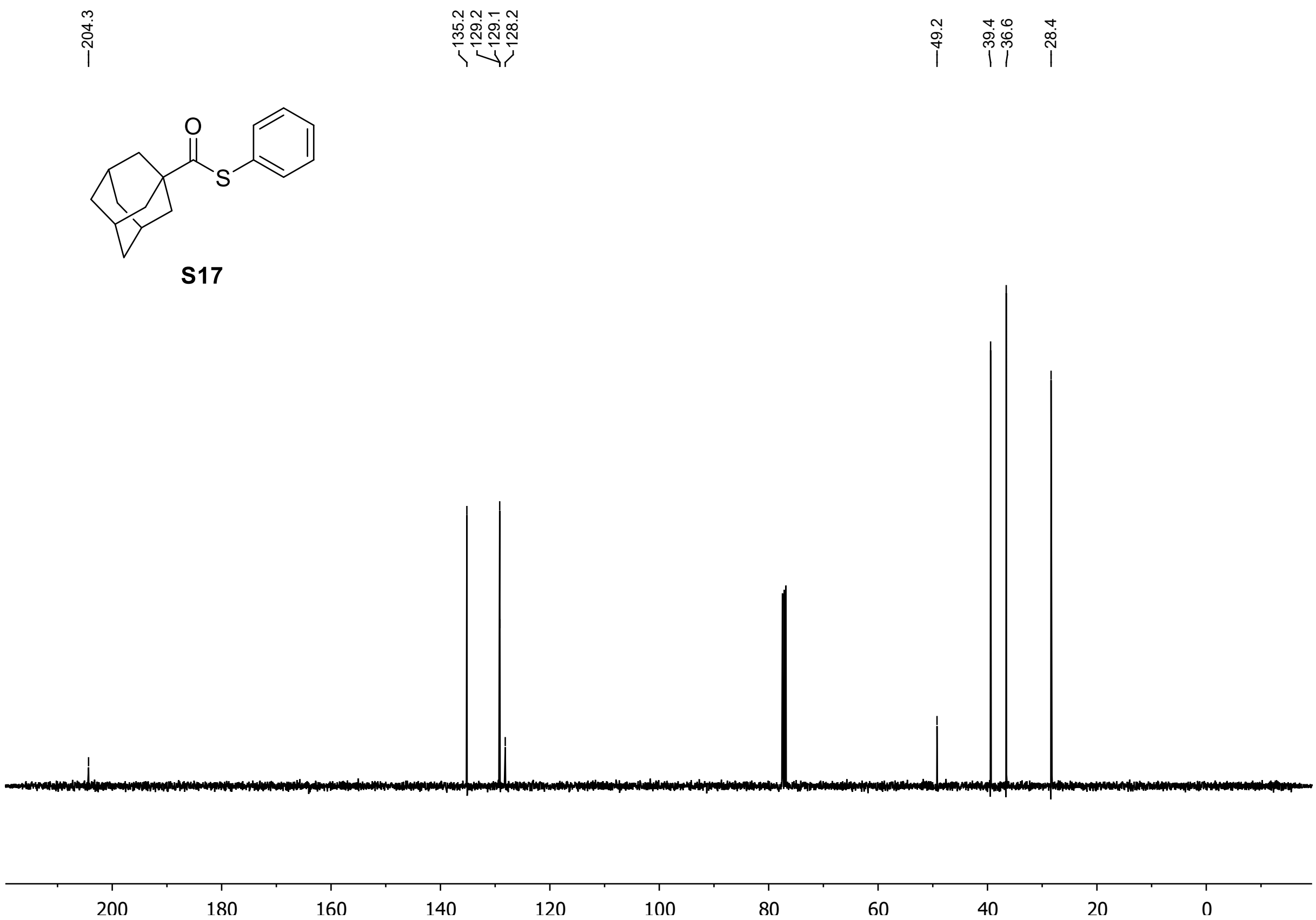

Figure S51. ^{13}C NMR spectrum (101 MHz, $CDCl_3$) of **S17**.

***S*-Phenyl 3-(cyclopropylmethoxy)-4-(difluoromethoxy)benzothioate (S18):**

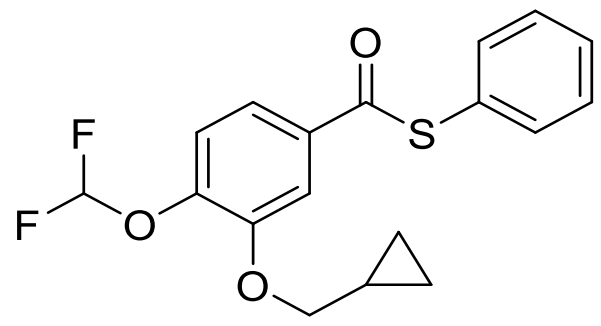

S18

$C_{18}H_{16}F_2O_3S$ (350.38 g/mol)

Following **GP-A2**, **S18** was synthesized using 3-(cyclopropylmethoxy)-4-(difluoromethoxy)benzoic acid (2.58 g, 10.0 mmol, 1.0 equiv.), thiophenol (1.23 mL, 12.0 mmol, 1.2 equiv.), EDCI (2.88 g, 15.0 mmol, 1.5 equiv.) and DMAP (122 mg, 1.0 mmol, 0.1 equiv.). Purification by flash column chromatography (*n*-hexane/EtOAc, gradient 100:0 to 80:20 over 10 CV) afforded **S18** (2.28 g, 6.78 mmol, 68%) as colorless solid. Conforms to reported analytical data.[13]

R_f: 0.47 (*n*-hexane/EtOAc 90:10).

^{1}H NMR (400 MHz, $CDCl_3$, δ): 7.69 (dd, *J* = 8.4, 2.0 Hz, 1H), 7.58 (d, *J* = 2.0 Hz, 1H), 7.55 – 7.45 (m, 5H), 7.30 – 7.27 (m, 1H), 6.75 (t, *J* = 74.8 Hz, 1H), 3.96 (d, *J* = 6.9 Hz, 2H), 1.39 – 1.29 (m, 1H), 0.74 – 0.62 (m, 2H), 0.47 – 0.33 (m, 2H).

^{13}C{^{1}H} NMR (101 MHz, $CDCl_3$, δ): 189.2, 150.6, 144.7, 135.2, 134.8, 129.8, 129.4, 127.2, 122.1, 121.1, 115.8 (t, J = 261.6 Hz), 112.9, 74.3, 10.2, 3.5.

^{19}F NMR (376 MHz, $CDCl_3$, δ): -81.9 (d, *J* = 74.8 Hz).

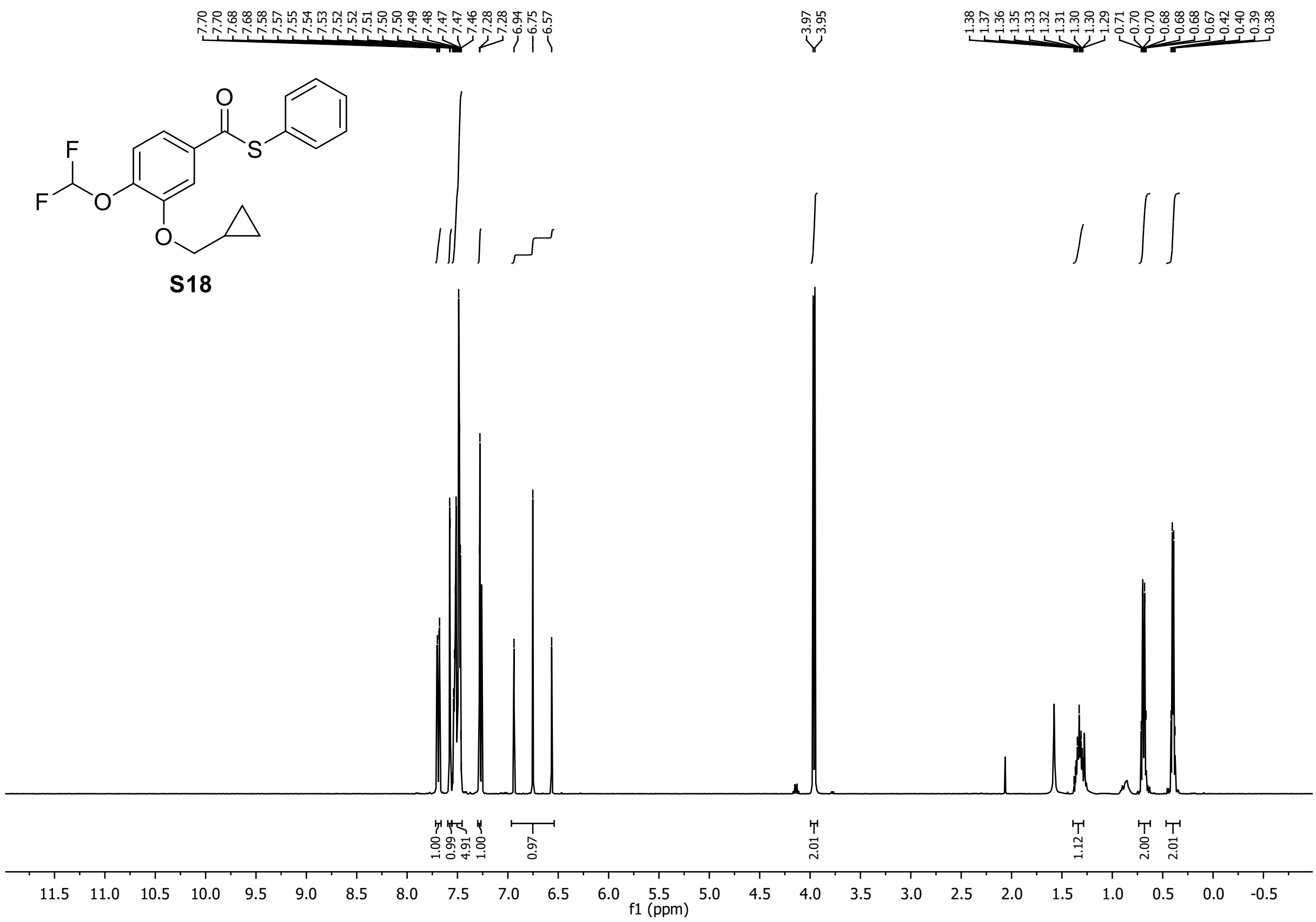

Figure S52. ^{1}H NMR spectrum (400 MHz, $CDCl_3$) of **S18**.

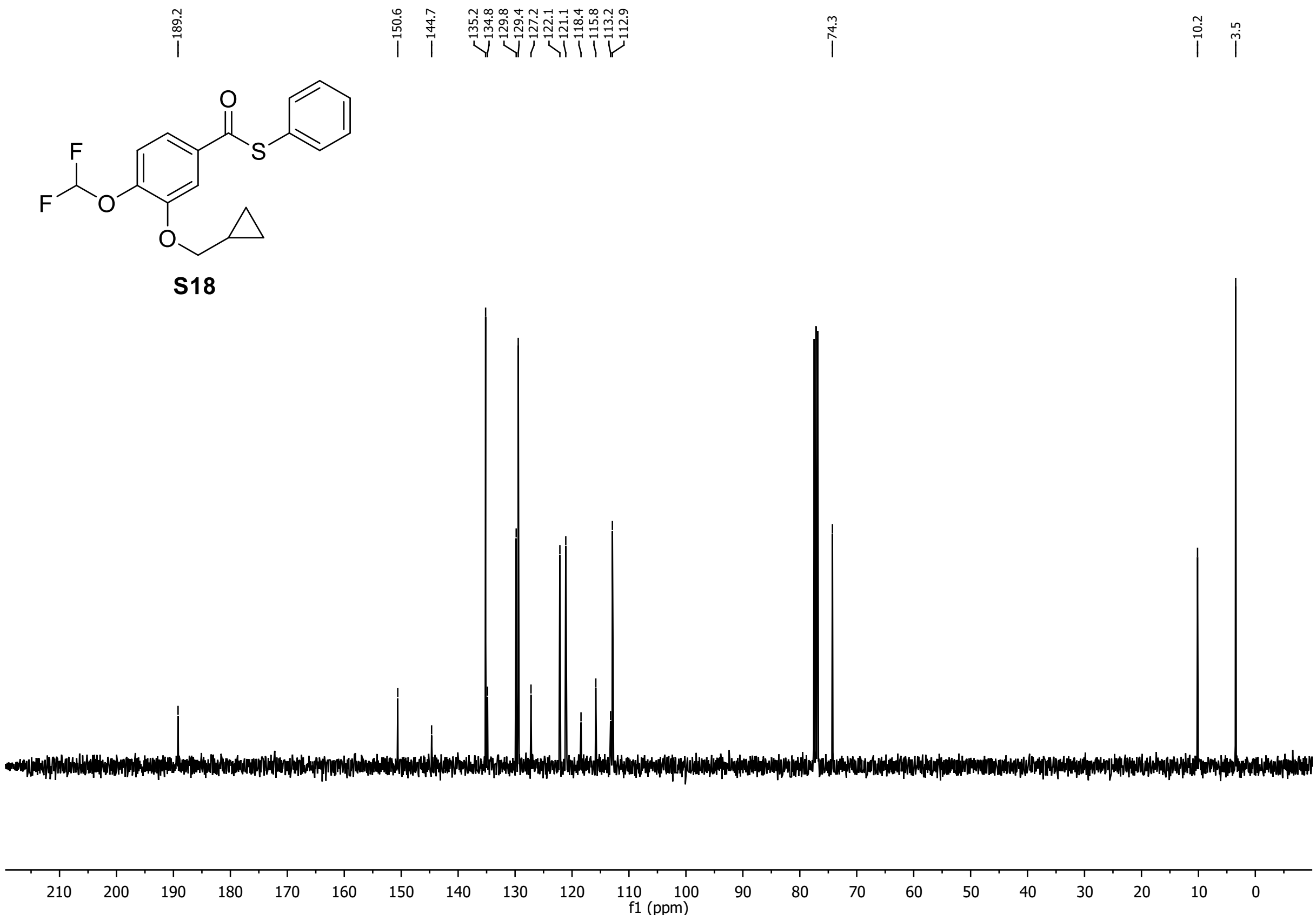

Figure S53. ^{13}C NMR spectrum (101 MHz, $CDCl_3$) of **S18**.

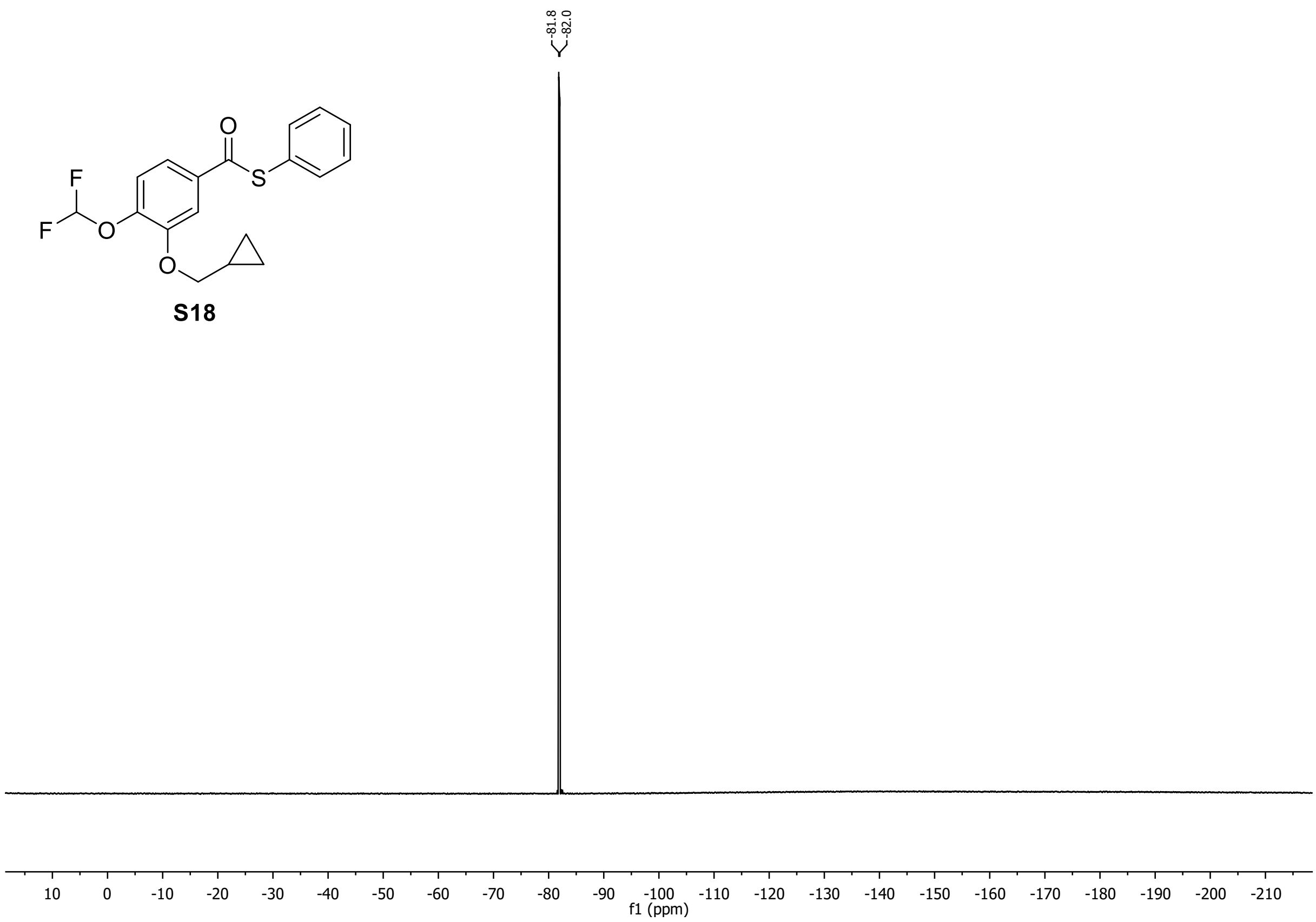

Figure S54. ^{19}F NMR spectrum (376 MHz, $CDCl_3$) of **S18**.

***S*-Phenyl 4-(*N*,*N*-dipropylsulfamoyl)benzothioate (S19):**

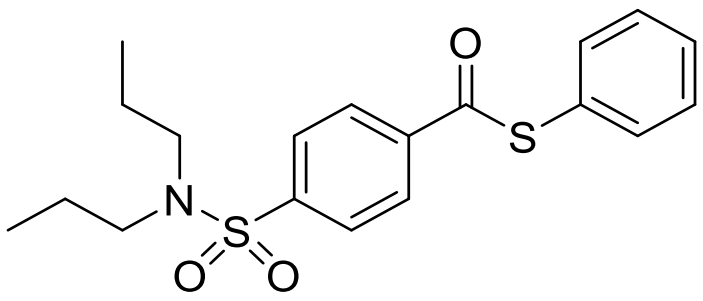

S19

$C_{19}H_{23}NO_3S_2$ (377.52 g/mol)

Following **GP-A2**, **S19** was synthesized using Probenecid (2.85 g, 10.0 mmol, 1.0 equiv.), thiophenol (1.23 mL, 12.0 mmol, 1.2 equiv.), EDCI (2.88 g, 15.0 mmol, 1.5 equiv.) and DMAP (122 mg, 1.0 mmol, 0.1 equiv.). Purification by flash column chromatography (*n*-hexane/EtOAc, gradient 100:0 to 30:70 over 10 CV) afforded **S19** (2.22 g, 5.87 mmol, 59%) as colorless solid. Conforms to reported analytical data.[9]

R$_f$: 0.61 (*n*-hexane/EtOAc 80:20).

^{1}H NMR (400 MHz, $CDCl_3$, δ): 8.17 – 8.09 (m, 2H), 7.96 – 7.88 (m, 2H), 7.56 – 7.44 (m, 5H), 3.16 – 3.07 (m, 4H), 1.64 – 1.49 (m, 4H, *residual water under compound peak*), 0.88 (t, *J* = 7.4 Hz, 6H).

^{13}C{^{1}H} NMR (101 MHz, $CDCl_3$, δ): 189.4, 144.9, 139.6, 135.1, 130.1, 129.6, 128.2, 127.5, 126.7, 50.1, 22.1, 11.3.

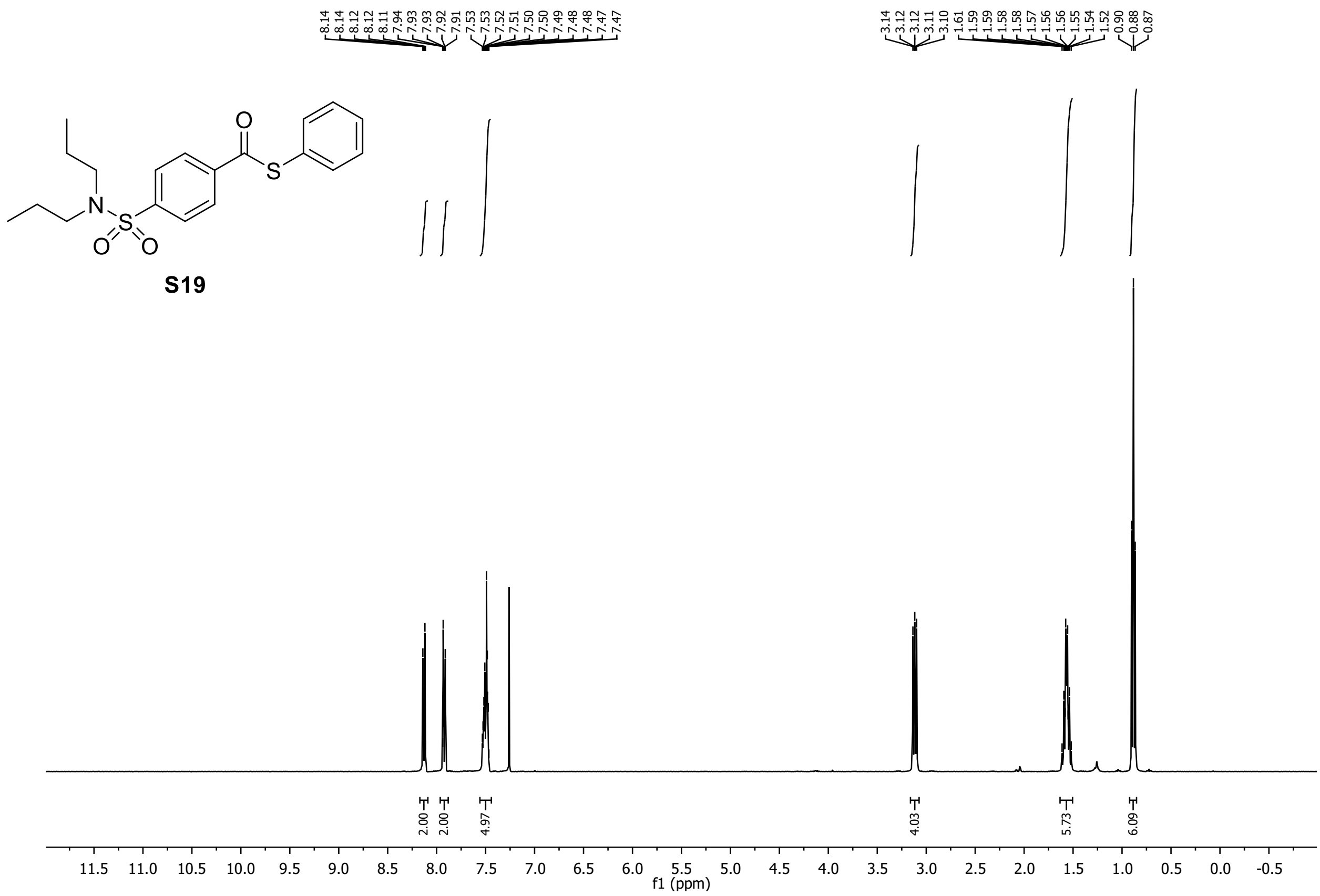

Figure S55. ^{1}H NMR spectrum (400 MHz, $CDCl_3$) of **S19**.

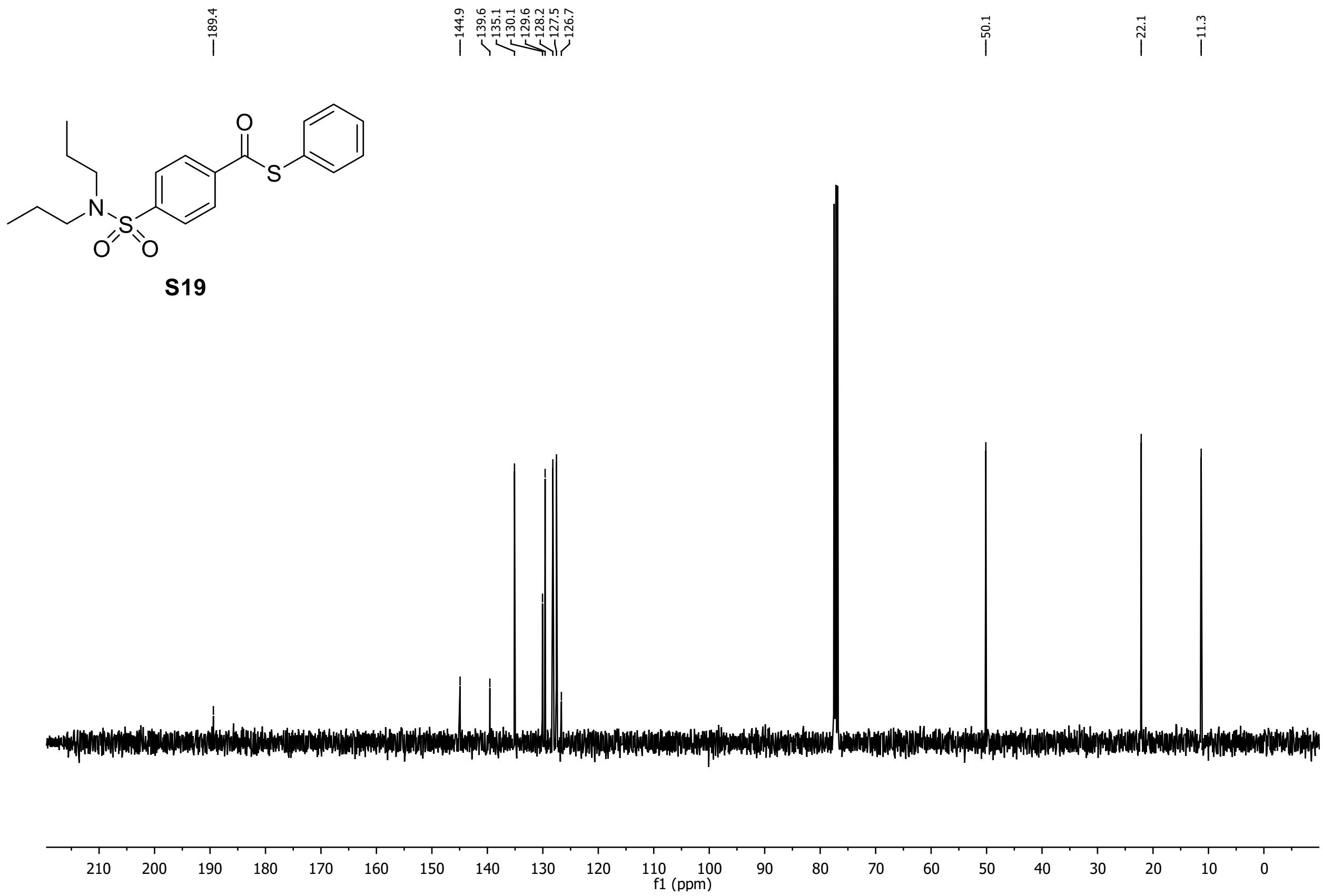

Figure S56. ^{13}C NMR spectrum (101 MHz, $CDCl_3$) of **S19**.

***S*-Phenyl oleate (S20):**

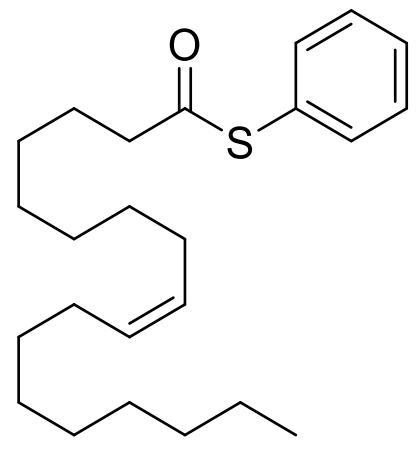

S20

$C_{24}H_{38}OS$ (374.63 g/mol)

Following **GP-1**, **S20** was synthesized using oleoyl chloride (6.02 g, 20.0 mmol, 1.0 equiv.), thiophenol (2.20 mL, 21.0 mmol, 1.05 equiv.) and triethylamine (3.35 mL, 24.0 mmol, 1.05 equiv.). Purification by column chromatography (hexane/EtOAc, gradient 100:0 to 90:10 over 10 CV) afforded **S20** (3.43 g, 9.16 mmol, 46%) as yellow oil. Conforms to reported analytical data.[14]

R$_f$: 0.85 (hexane/EtOAc 95:5).

^{1}H NMR (400 MHz, $CDCl_3$, δ): 7.45 – 7.38 (m, 5H), 5.41 – 5.33 (m, 2H), 2.66 (t, *J* = 7.5 Hz, 2H), 2.13 – 1.95 (m, 4H), 1.77 – 1.68 (m, 2H), 1.43 – 1.22 (m, 20H), 0.93 – 0.86 (m, 3H).

^{13}C{^{1}H} NMR (101 MHz, $CDCl_3$, δ): 197.6, 134.6, 130.4, 130.2, 129.8, 129.4, 129.3, 128.2, 128.1, 128.0, 43.9, 29.8, 29.7, 29.7, 29.6, 29.5, 29.4, 29.3, 29.2, 29.1, 27.4, 27.3, 25.8, 25.7, 22.8, 22.7, 14.3, 14.2.

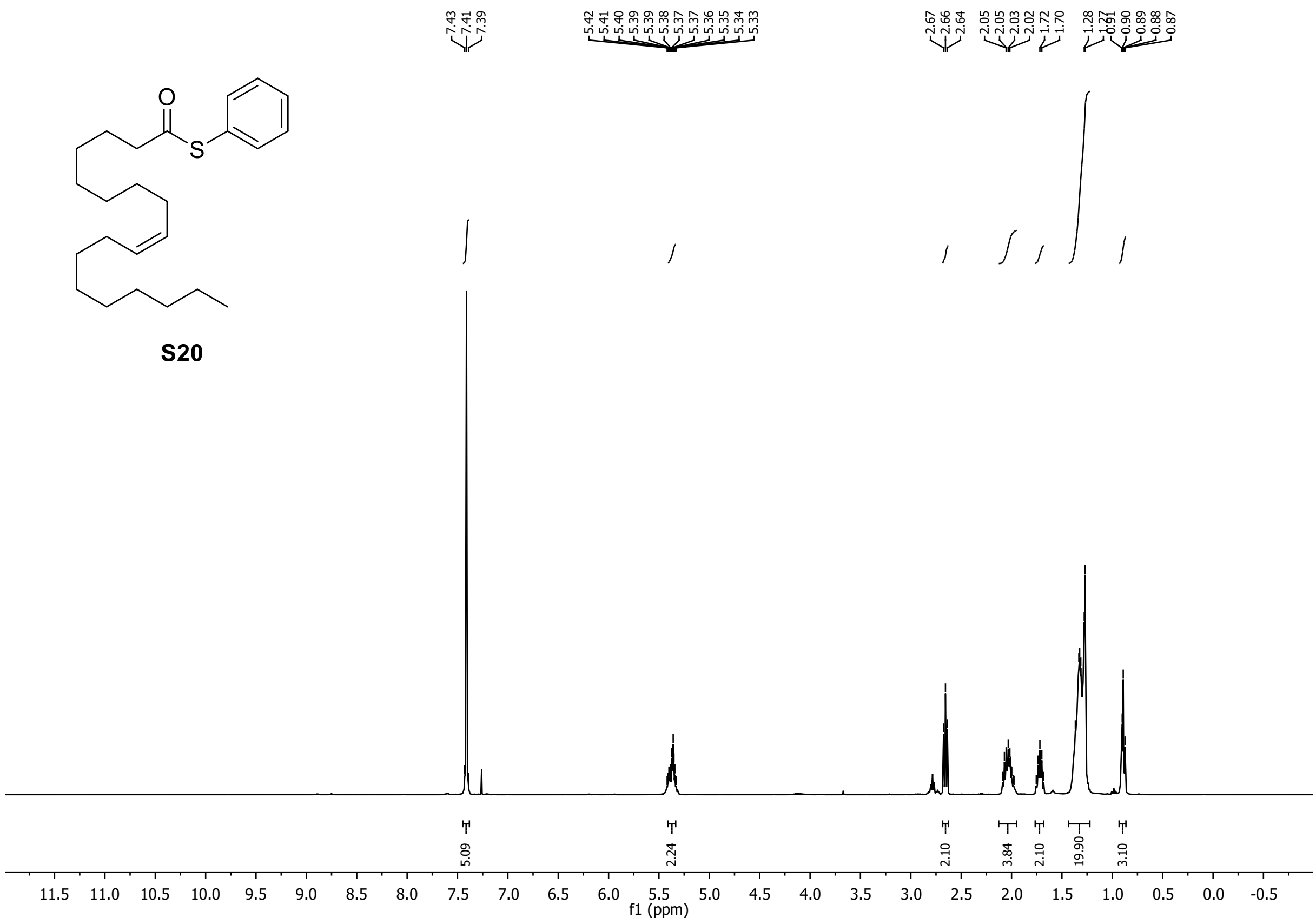

Figure S57. ^{1}H NMR spectrum (400 MHz, $CDCl_3$) of **S20**.

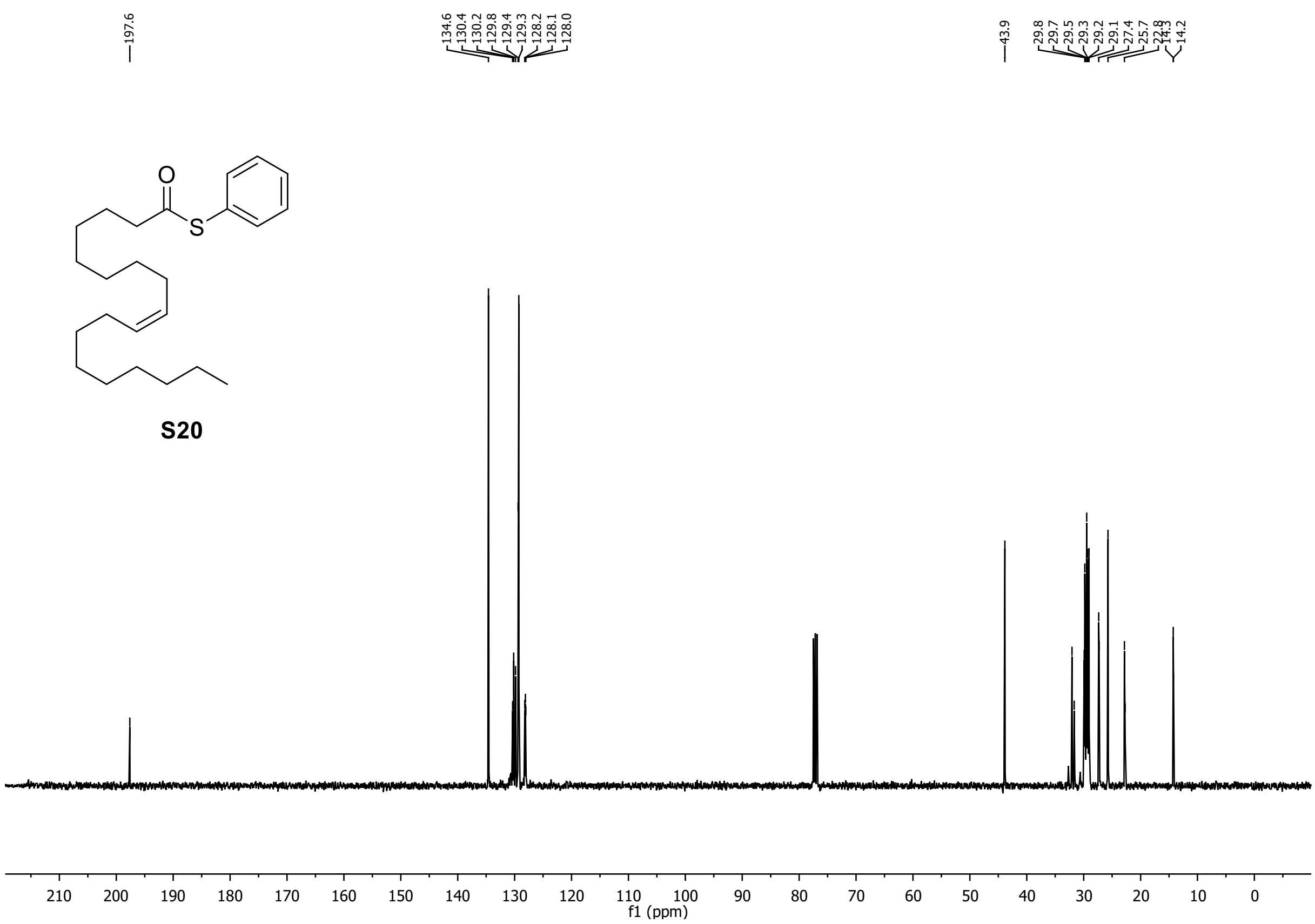

Figure S58. ^{13}C NMR spectrum (101 MHz, $CDCl_3$) of **S20**.

5.3 Synthesis of Acrylates

(1*R*,2*S*,5*R*)-2-Isopropyl-5-methylcyclohexyl acrylate (S24):

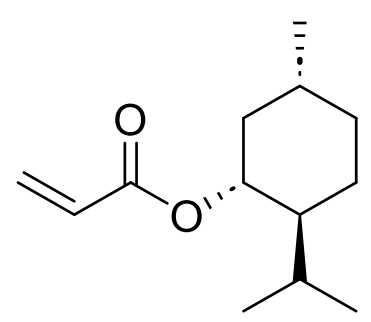

S24

$C_{13}H_{22}O_2$ (210.32 g/mol)

Acryloyl chloride (1.81 g, 20.0 mmol, 1.0 equiv.) and (–)-menthol (3.28 g, 21.0 mmol, 1.05 equiv.) and triethylamine (2.43 g, 24.0 mmol, 1.2 equiv.) were dissolved in DCM (60 mL) and stirred at rt for 18 h. Hydrochloric acid (1 M, 20 mL) was added and the organic layer was washed with sat. $NaHCO_3$ (20 mL) and brine (20 mL), dried over $MgSO_4$ and filtered. Evaporation of the solvent afforded **S24** (4.08 g, 19.4 mmol, 97%) as yellow oil, which was used without further purification. Conforms to reported analytical data.[15]

^{1}H NMR (400 MHz, $CDCl_3$, δ): 6.41 (dd, *J* = 17.3, 1.5 Hz, 1H), 6.13 (dd, *J* = 17.3, 10.4 Hz, 1H), 5.82 (dd, *J* = 10.4, 1.5 Hz, 1H), 4.79 (td, *J* = 10.9, 4.4 Hz, 1H), 2.10 – 2.02 (m, 1H), 1.95 – 1.85 (m, 1H), 1.76 – 1.68 (m, 2H), 1.60 – 1.40 (m, 2H), 1.18 – 0.98 (m, 2H), 0.97 – 0.88 (m, 7H), 0.80 (d, *J* = 7.0 Hz, 3H).

^{13}C{^{1}H} NMR (101 MHz, $CDCl_3$, δ): 165.9, 130.3, 129.2, 74.4, 47.2, 41.0, 34.4, 31.5, 26.5, 23.7, 22.1, 20.8, 16.6.

Figure S59. ^{1}H NMR spectrum (400 MHz, $CDCl_3$) of **S24**.

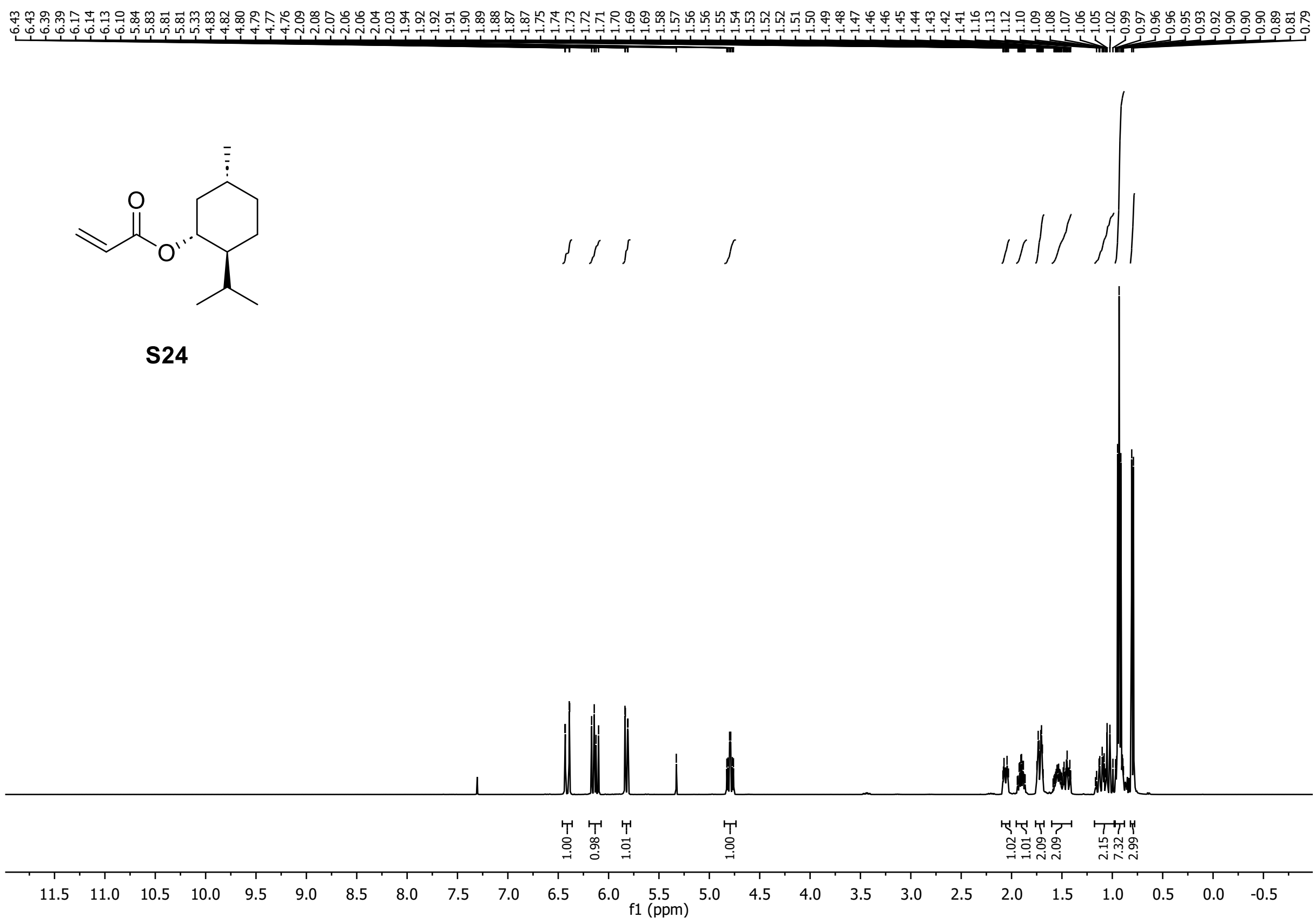

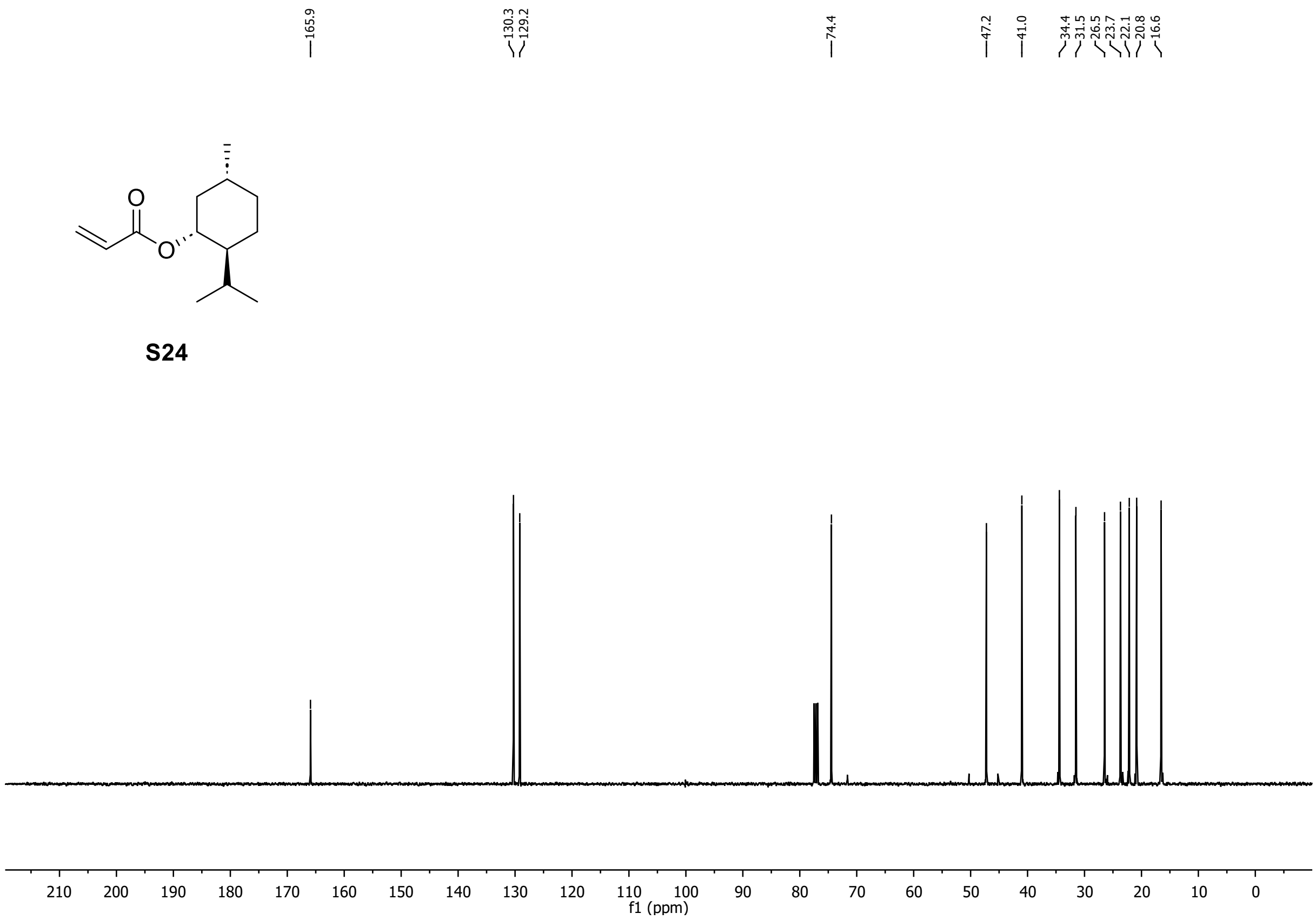

Figure S60. ^{13}C NMR spectrum (101 MHz, $CDCl_3$) of **S24**.

Methyl-d_3 acrylate-2-*d* (S30a):

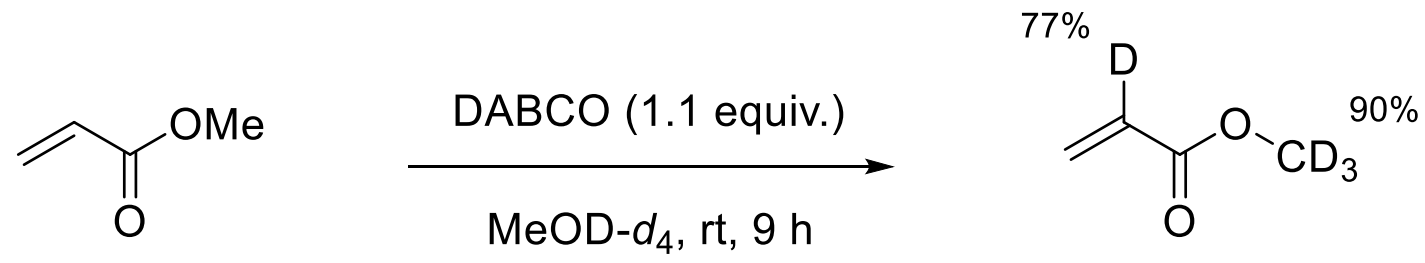

S30a

$C_4H_2D_4O_2$ (90.11 g/mol)

S30a was synthesized following a literature procedure.[16]

Methyl acrylate (6.65 g, 77.2 mmol, 1.0 equiv.) and 1,4-diazabicyclo(2.2.2)octane (9.53 g, 85.0 mmol, 1.1 equiv.) were dissolved in methanol-d_4 (25 mL) and stirred at rt for 9 h. 1,2-Dichlorobenzene (20 mL) was added and the mixture was washed with water (10 mL) and brine (10 mL), dried over $MgSO_4$ and filtered. Fractional distillation (200 mbar, 85 °C) afforded **S30a** (1.04 g, 11.5 mmol, 15%) as colorless oil.

The deuterium incorporation was determined:

^{1}H NMR: 77% D in position 2, 90% D at the CD_3 group.

^{1}H NMR (400 MHz, $CDCl_3$, δ): 6.42 – 6.33 (m, 1H), 6.09 (dd, *J* = 17.3, 10.4 Hz, 0.23H), 5.83 – 5.76 (m, 1H), 3.72 (s, 0.30H).

^{2}H NMR (46 MHz, $CDCl_3$, δ): 6.15 (dd, *J* = 2.6, 1.6 Hz), 3.72.

^{13}C{^{1}H} NMR (101 MHz, $CDCl_3$, δ): 166.7, 130.8, 130.7, 128.3 (C–H), 128.0 (t, *J* = 25.3 Hz, C–D), 51.7 (C–H), 50.9 (t, *J* = 22.6 Hz, C–D).

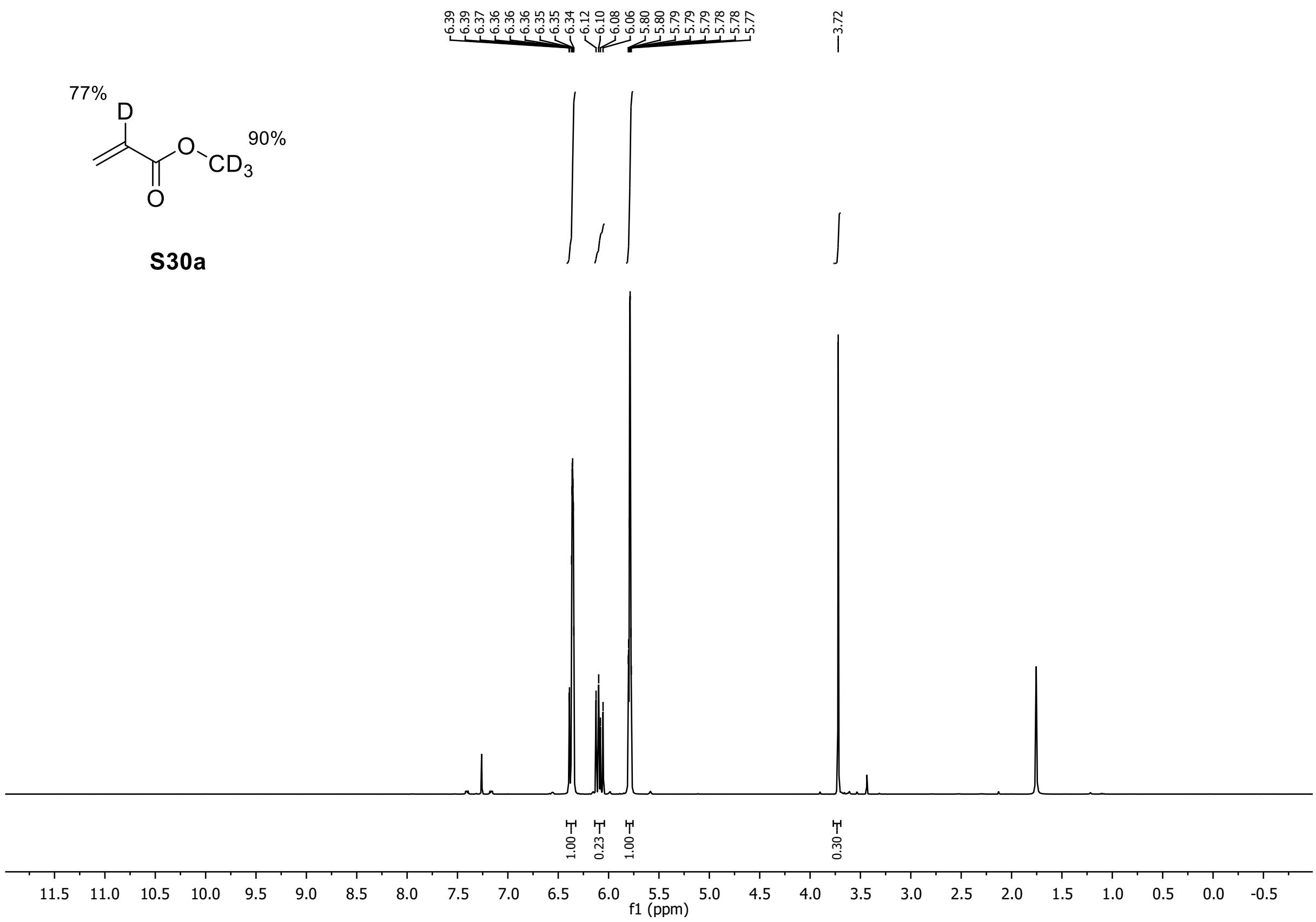

Figure S61. ^1H NMR spectrum (400 MHz, $CDCl_3$) of **S30a**.

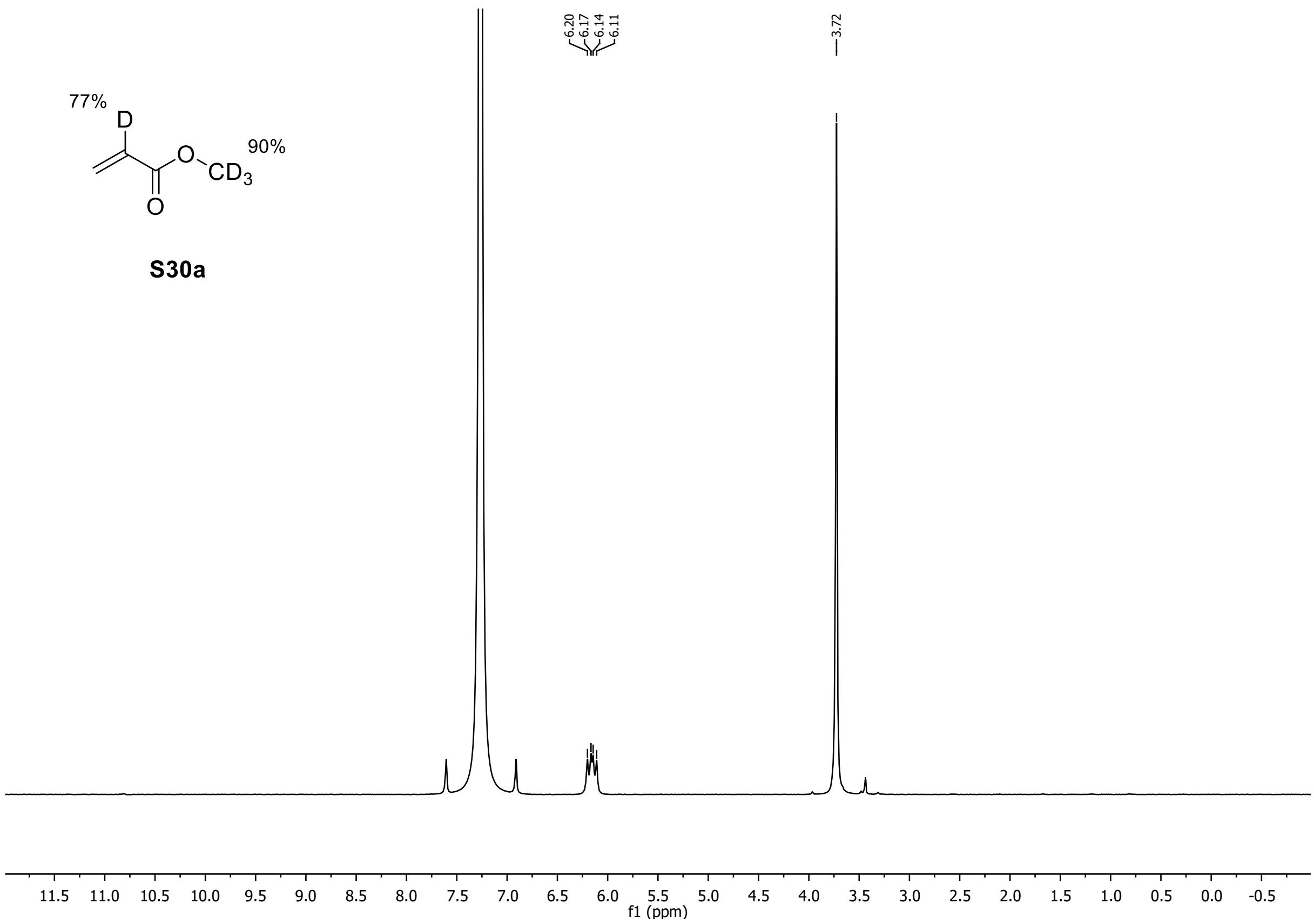

Figure S62. ^2H NMR spectrum (46 MHz, $CDCl_3$) of **S30a**.

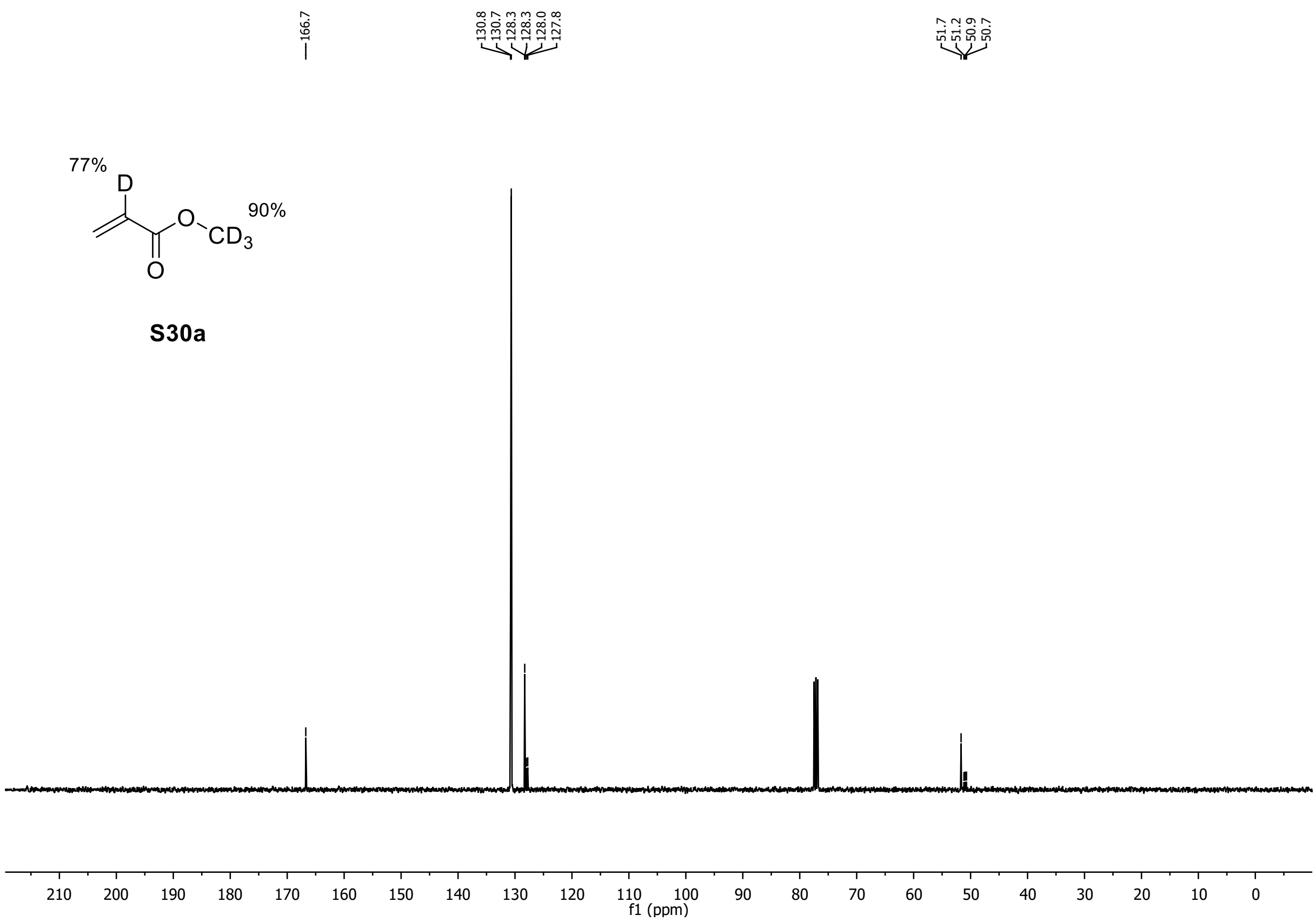

Figure S63. ^{13}C NMR spectrum (101 MHz, CDCl3) of **S30a**.

Methyl acrylate-2,3,3-d_3 (S30b):

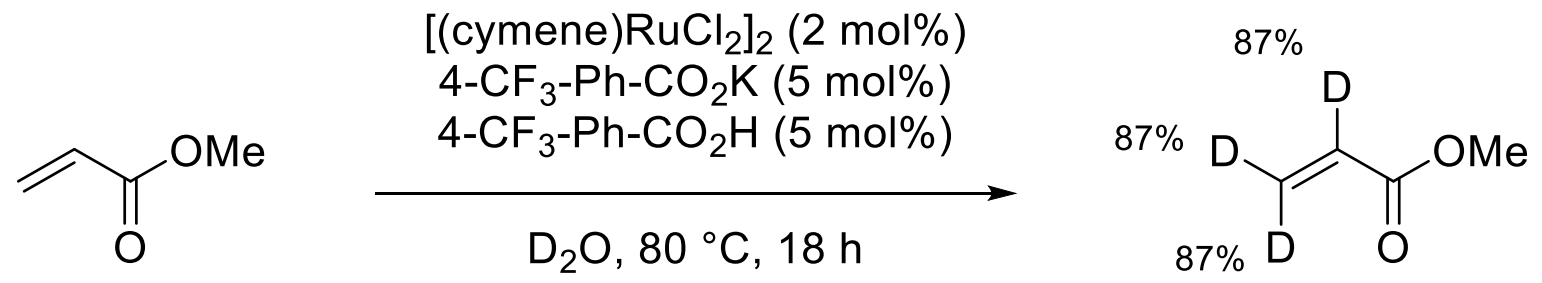

S30b

$C_3H_3D_3O_2$ (89.11 g/mol)

S30b was synthesized following a literature procedure.[17]

Methyl acrylate (4.75 g, 55.2 mmol, 1.0 equiv.), $[(cymene)RuCl_2]_2$ (338 mg, 1.10 mmol, 2 mol%), potassium 3-(trifluoromethyl)benzoate (630 mg, 2.76 mmol, 5 mol%), and 3-(trifluoromethyl)benzoic acid (525 mg, 2.76 mmol, 5 mol%) were suspended in D_2O (10 mL) and stirred at 80 °C for 18 h. The organic layer was separated and bulb-to-bulb vacuum distillation afforded **S30b** (1.43 g, 16.0 mmol, 29%) as colorless oil.

The deuterium incorporation was determined:

^{1}H NMR: 87% D in position 2 and 3.

^{1}H NMR (400 MHz, $CDCl_3$, δ): 6.42 – 6.32 (m, 0.13H), 6.15 – 6.03 (m, 0.13H), 5.83 – 5.73 (m, 0.13H), 3.73 (s, 3H).

^{2}H NMR (46 MHz, $CDCl_3$, δ): 6.37, 6.10, 5.80.

^{13}C{^{1}H} NMR (101 MHz, $CDCl_3$, δ): 166.8, 130.4 (m), 127.8 (m), 51.7.

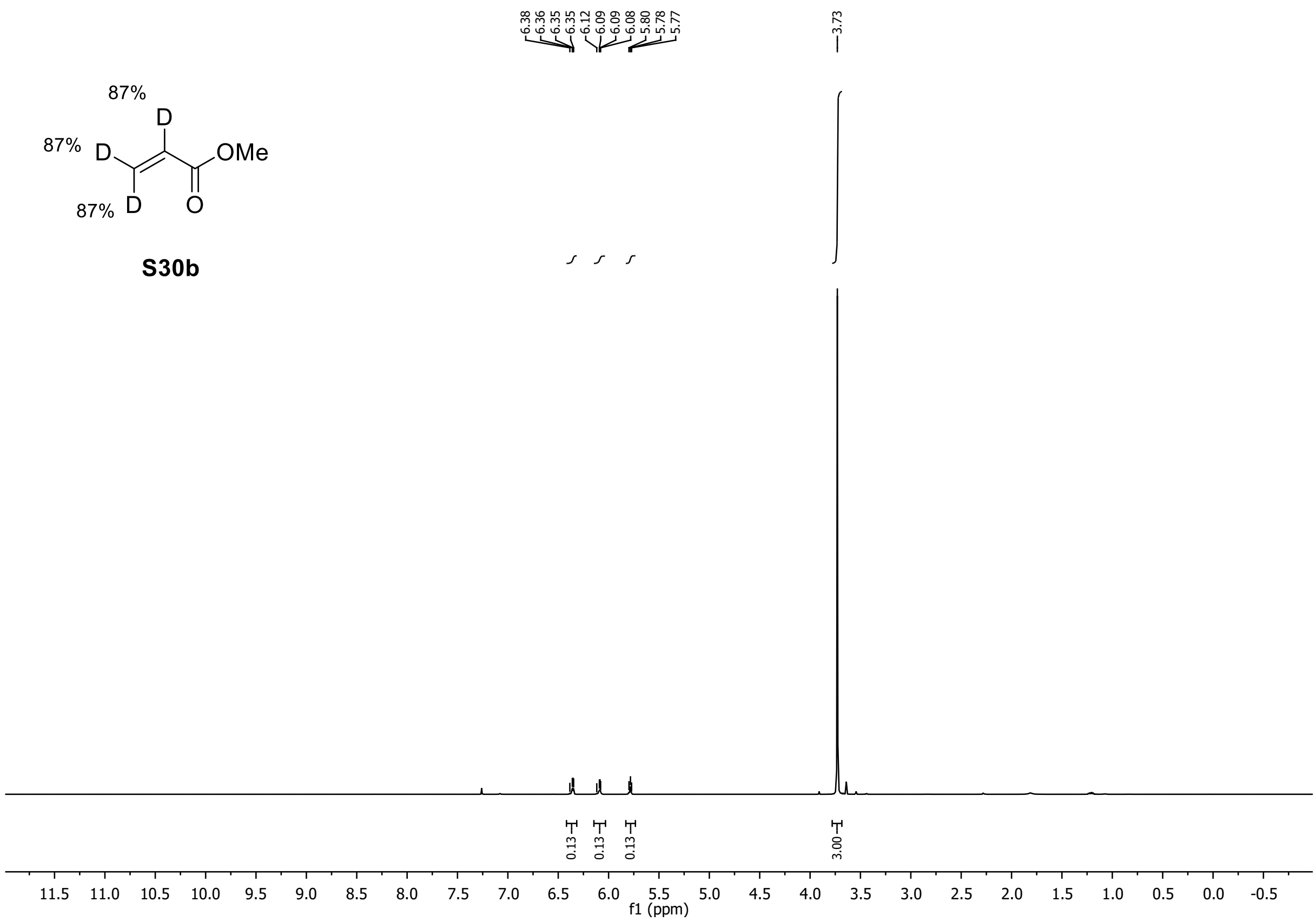

Figure S64. ^{1}H NMR spectrum (400 MHz, $CDCl_3$) of **S30b**.

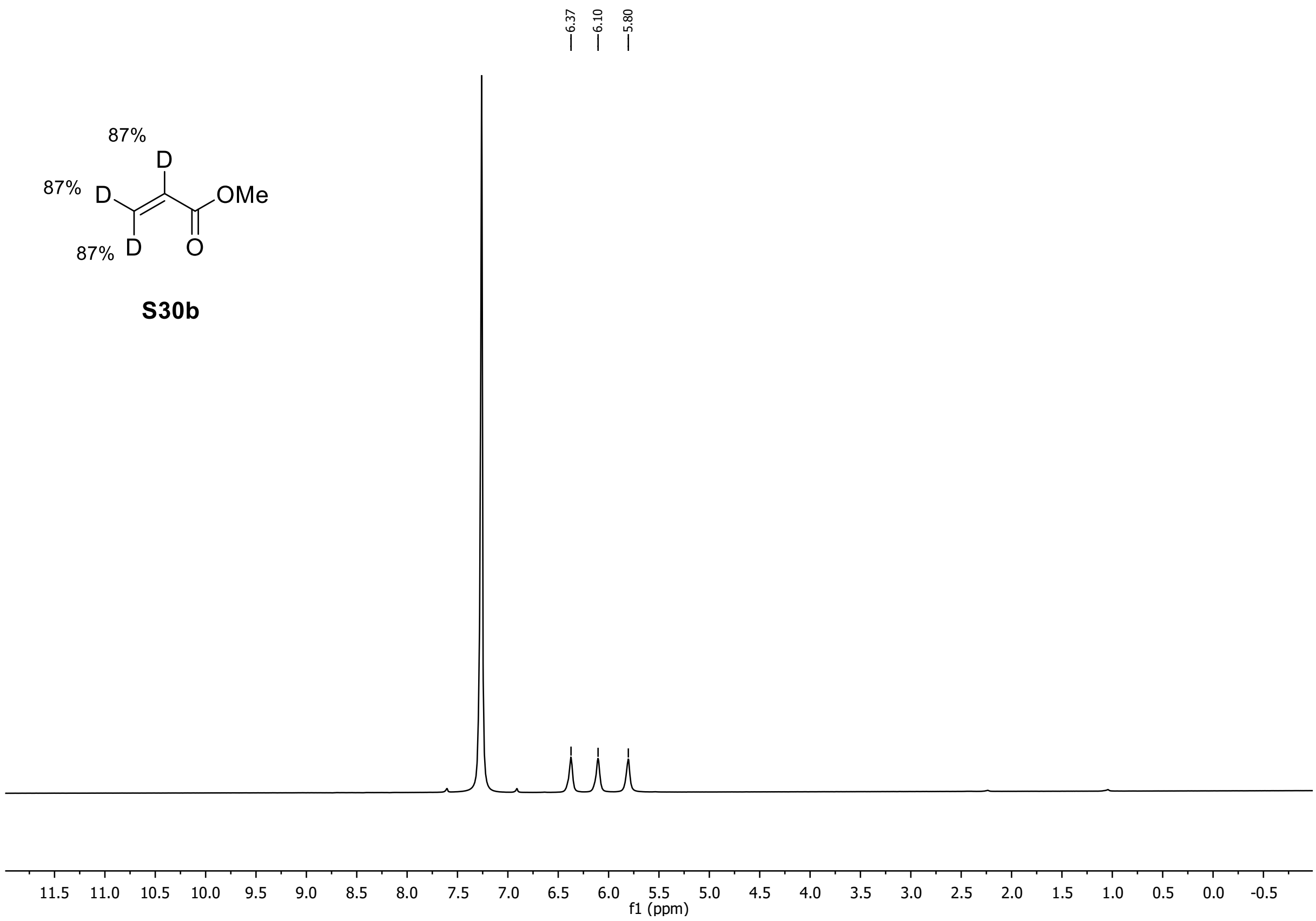

Figure S65. ^{2}H NMR spectrum (46 MHz, $CDCl_3$) of **S30b**.

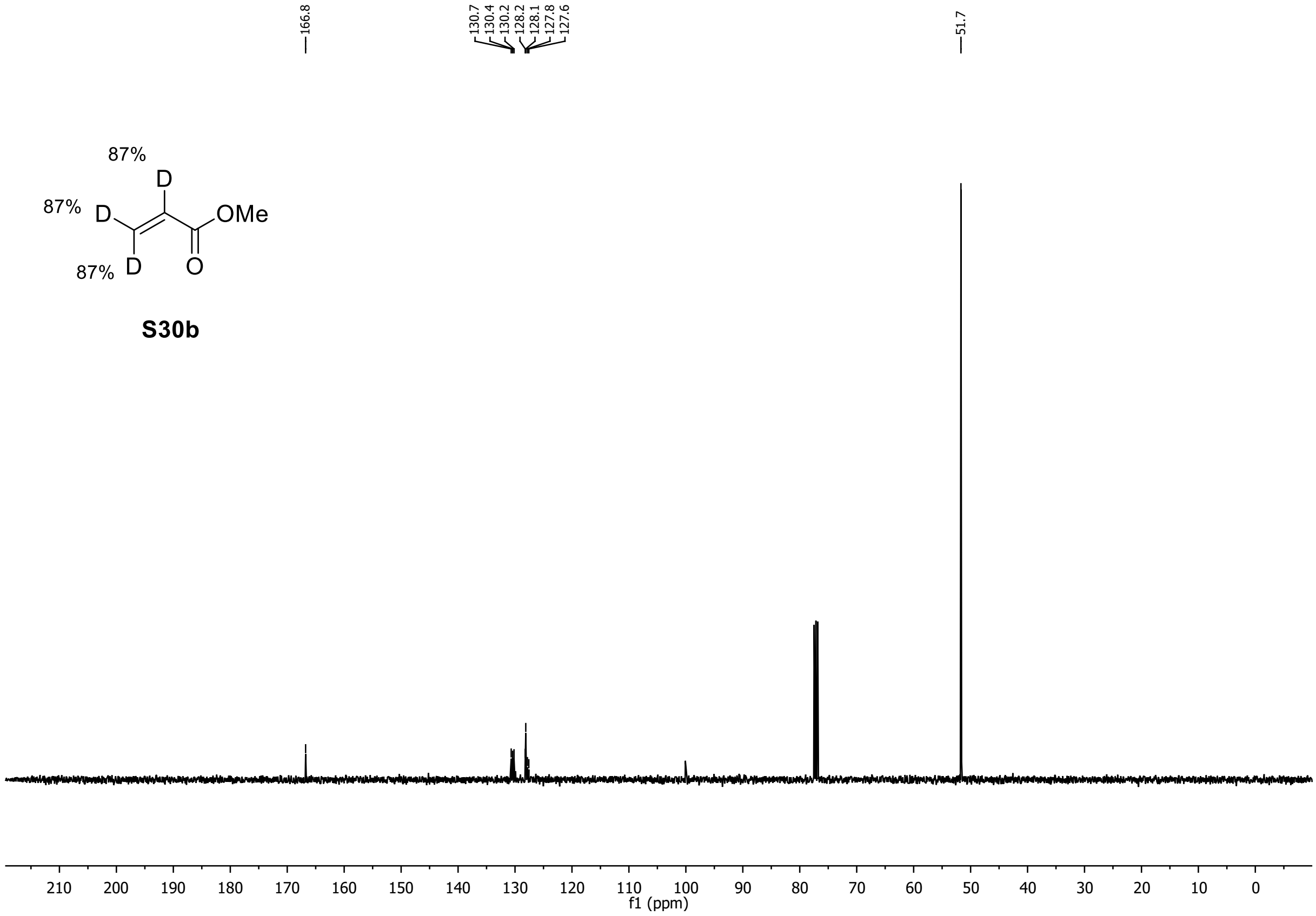

Figure S66. ^{13}C NMR spectrum (101 MHz, $CDCl_3$) of **S30b**.

(8*R*,9*S*,13*S*,14*S*)-13-Methyl-17-oxo-7,8,9,11,12,13,14,15,16,17-decahydro-6*H*-cyclopenta[*a*]phenanthren-3-yl acrylate (S29):

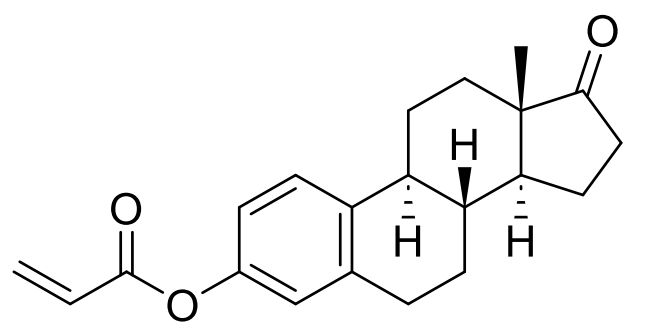

S29

$C_{21}H_{24}O_3$ (324.42 g/mol)

Acryloyl chloride (1.56 g, 17.2 mmol, 1.0 equiv.) and estrone (5.14 g, 18.1 mmol, 1.05 equiv.) and triethylamine (2.09 g, 20.7 mmol, 1.2 equiv.) were suspended in DCM (60 mL) and stirred at rt for 18 h. Hydrochloric acid (1 M, 20 mL) was added, and the organic layer was washed with sat. $NaHCO_3$ (20 mL) and brine (20 mL), dried over $MgSO_4$ and filtered. Evaporation of the solvent afforded **S29** (5.21 g, 15.4 mmol, 90%) as pale yellow solid, which was used without further purification. Conforms to reported analytical data.[18]

^{1}H NMR (400 MHz, $CDCl_3$, δ): 7.30 (d, *J* = 8.5 Hz, 1H), 6.93 – 6.84 (m, 2H), 6.59 (dd, *J* = 17.3, 1.3 Hz, 1H), 6.31 (dd, *J* = 17.3, 10.4 Hz, 1H), 6.00 (dd, *J* = 10.4, 1.3 Hz, 1H), 2.95 – 2.89 (m, 2H), 2.56 – 2.47 (m, 1H), 2.45 – 2.38 (m, 1H), 2.34 – 2.25 (m, 1H), 2.21 – 1.93 (m, 5H), 1.65 – 1.43 (m, 7H), 0.91 (s, 3H).

^{13}C{^{1}H} NMR (101 MHz, $CDCl_3$, δ): 220.9, 165.0, 148.6, 138.2, 137.6, 132.5, 128.2, 126.6, 121.7, 118.8, 50.6, 48.1, 44.3, 38.1, 36.0, 31.7, 29.5, 26.5, 25.9, 21.7, 14.0.

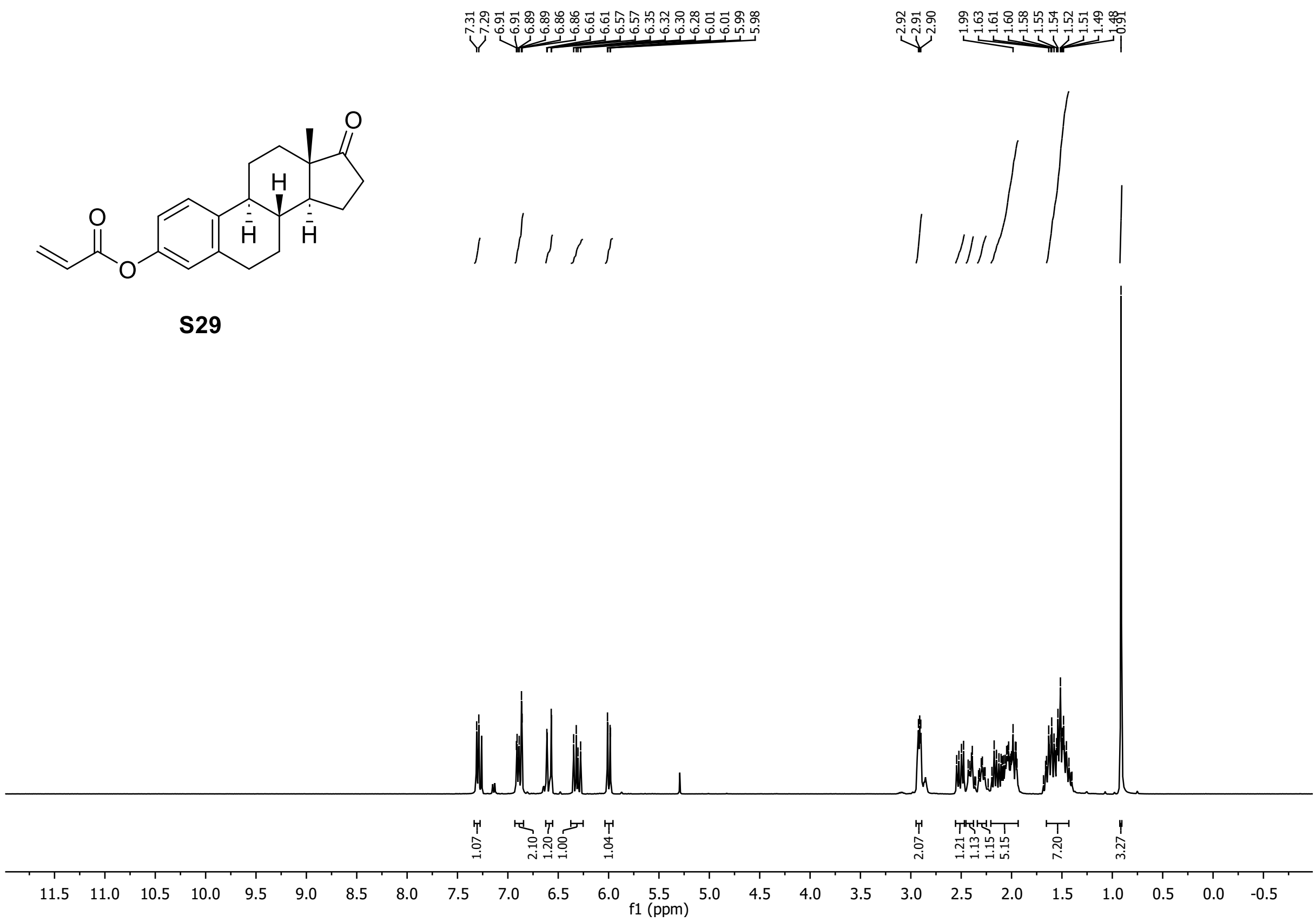

Figure S67. ^{1}H NMR spectrum (400 MHz, $CDCl_3$) of **S29**.

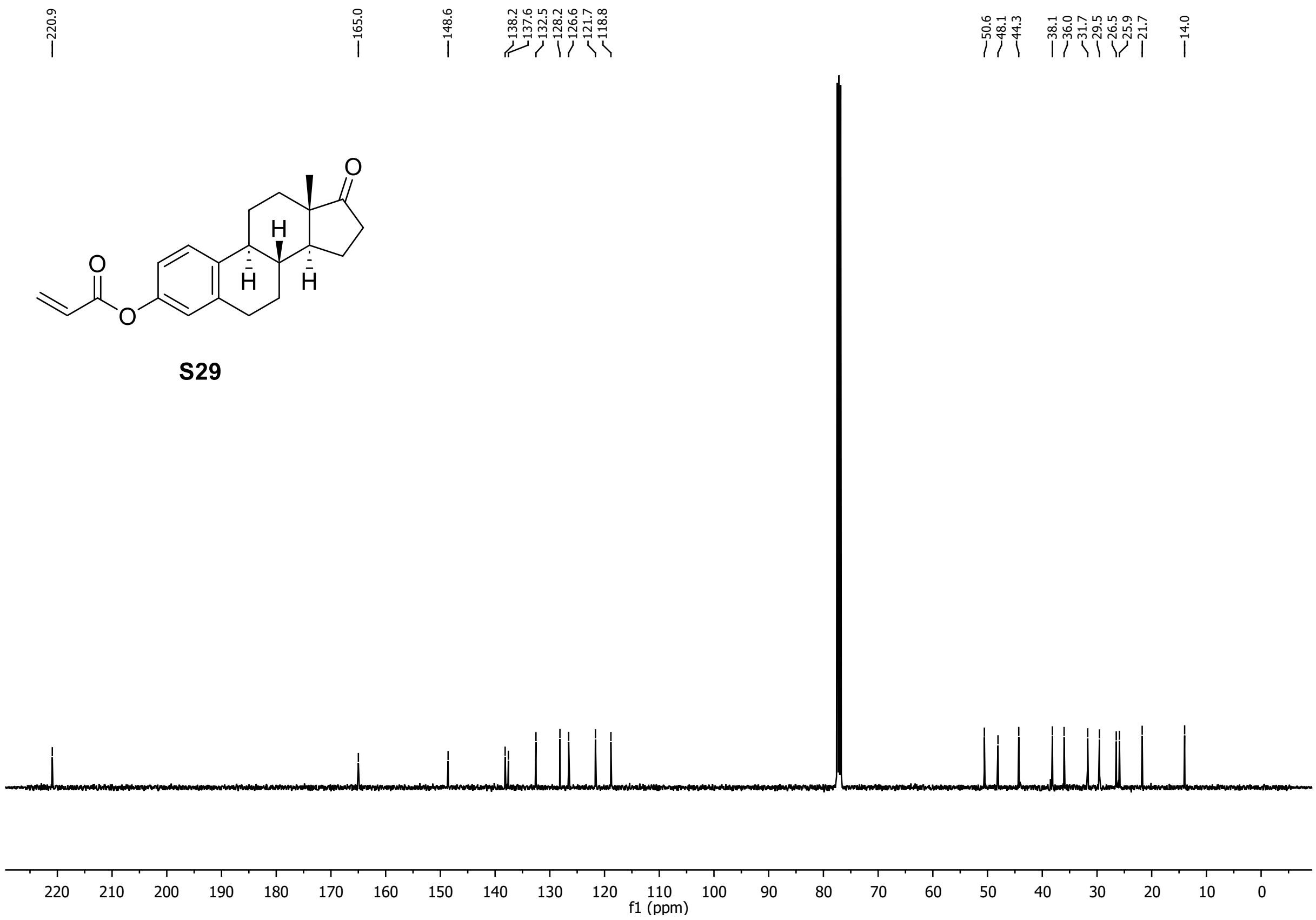

Figure S68. ^{13}C NMR spectrum (101 MHz, $CDCl_3$) of **S29**.

5.4 Synthesis of 1,4-Dicarbonyl Compounds

Ethyl 4-oxo-4-phenylbutanoate (1):

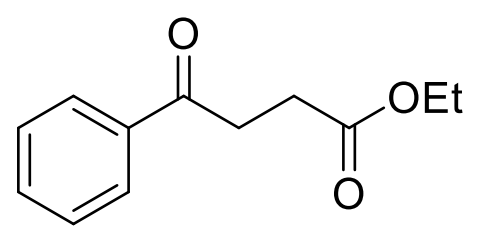

1

$C_{12}H_{14}O_3$ (206.24 g/mol)

Following **GP-B**, **1** was synthesized using **S1** (214 mg, 1.0 mmol, 1.0 equiv.) and ethyl acrylate (200 mg, 2.0 mmol, 2 equiv.). Purification by flash column chromatography (*n*-hexane/EtOAc, gradient 100:0 to 70:30 over 10 CV) afforded **1** (159 mg, 771 µmol, 77%) as colorless oil. Conforms to reported analytical data.[19]

R$_f$: 0.55 (*n*-hexane/EtOAc 80:20).

^{1}H NMR (400 MHz, $CDCl_3$, δ): 8.02 – 7.94 (m, 2H), 7.61 – 7.51 (m, 1H), 7.50 – 7.41 (m, 2H), 4.15 (q, *J* = 7.1 Hz, 2H), 3.31 (t, *J* = 6.6 Hz, 2H), 2.75 (t, *J* = 6.6 Hz, 2H), 1.26 (t, *J* = 7.1 Hz, 3H).

^{13}C{^{1}H} NMR (101 MHz, $CDCl_3$, δ): 198.2, 173.0, 136.7, 133.3, 128.7, 128.1, 60.8, 33.5, 28.4, 14.3.

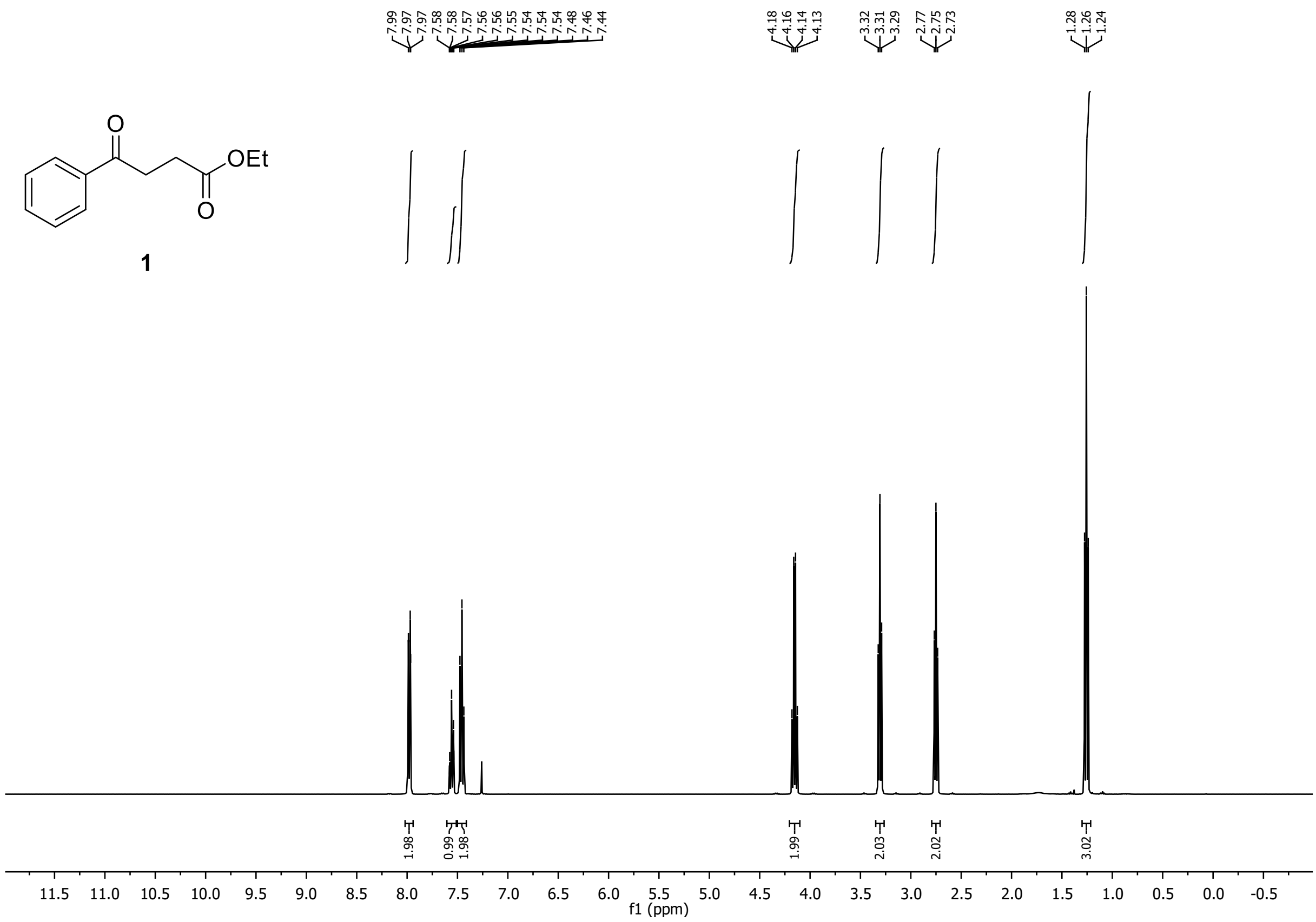

Figure S69. ^{1}H NMR spectrum (400 MHz, $CDCl_3$) of **1**.

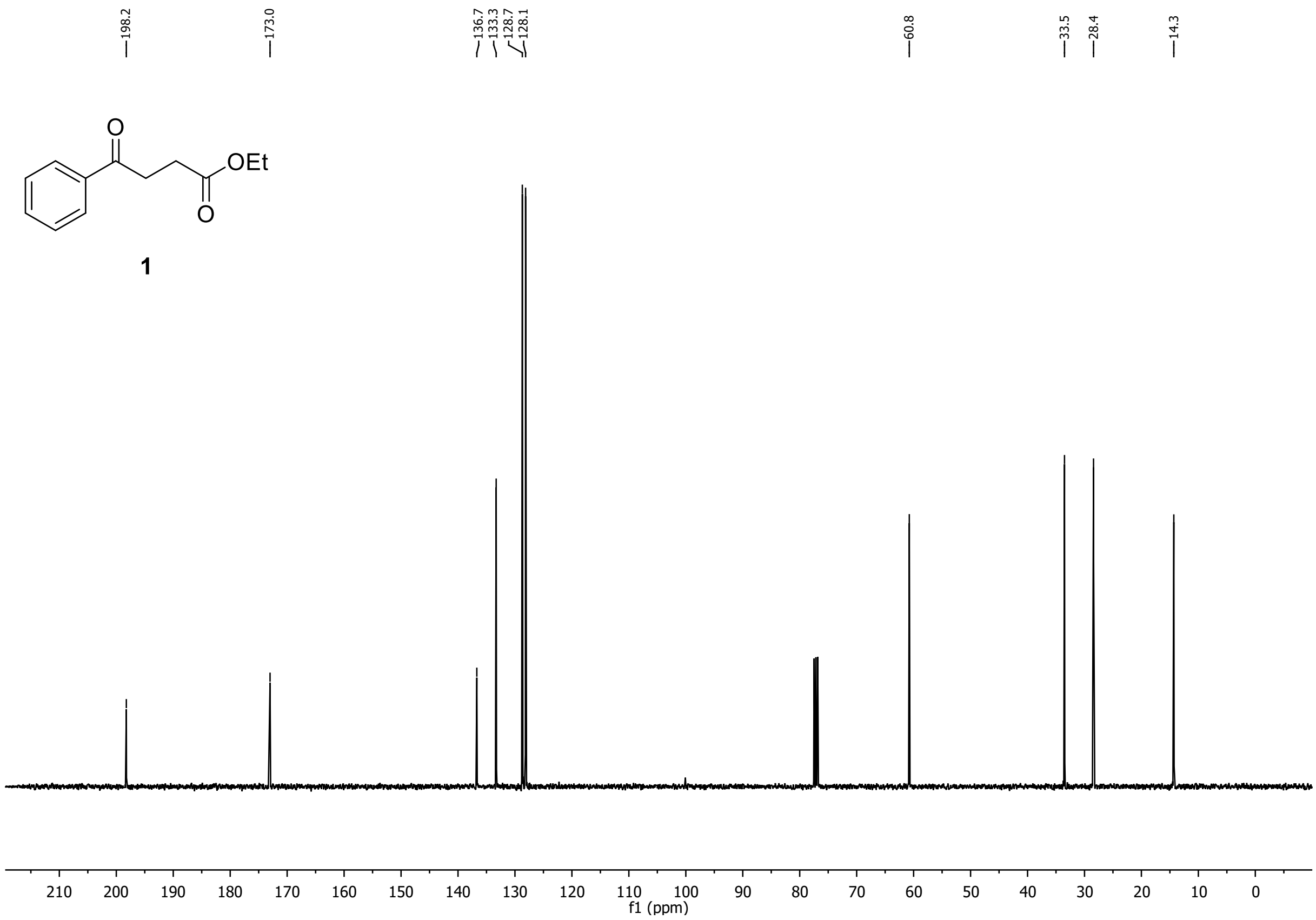

Figure S70. ^{13}C NMR spectrum (101 MHz, $CDCl_3$) of **1**.

Ethyl 4-oxo-4-(*o*-tolyl)butanoate (2):

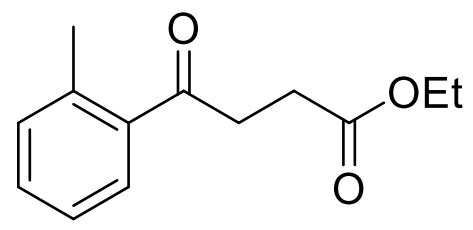

2

$C_{13}H_{16}O_3$ (220.27 g/mol)

Following **GP-B**, **2** was synthesized using **S2** (228 mg, 1.0 mmol, 1.0 equiv.) and ethyl acrylate (200 mg, 2.0 mmol, 2 equiv.). Purification by flash column chromatography (*n*-hexane/EtOAc, gradient 100:0 to 70:30 over 10 CV) afforded **2** (128 mg, 581 µmol, 58%) as colorless oil. Conforms to reported analytical data.[20]

R_f: 0.64 (*n*-hexane/EtOAc 80:20).

^{1}H NMR (400 MHz, $CDCl_3$, δ): 7.74 – 7.67 (m, 1H), 7.41 – 7.32 (m, 1H), 7.31 – 7.21 (m, 2H), 4.16 (q, J = 7.1 Hz, 2H), 3.21 (t, J = 6.5 Hz, 2H), 2.73 (t, J = 6.5 Hz, 2H), 2.49 (s, 3H), 1.26 (t, J = 7.1 Hz, 3H).

^{13}C{^{1}H} NMR (101 MHz, $CDCl_3$, δ): 202.2, 173.0, 138.3, 137.7, 132.0, 131.5, 128.6, 125.8, 60.8, 36.2, 28.6, 21.4, 14.3.

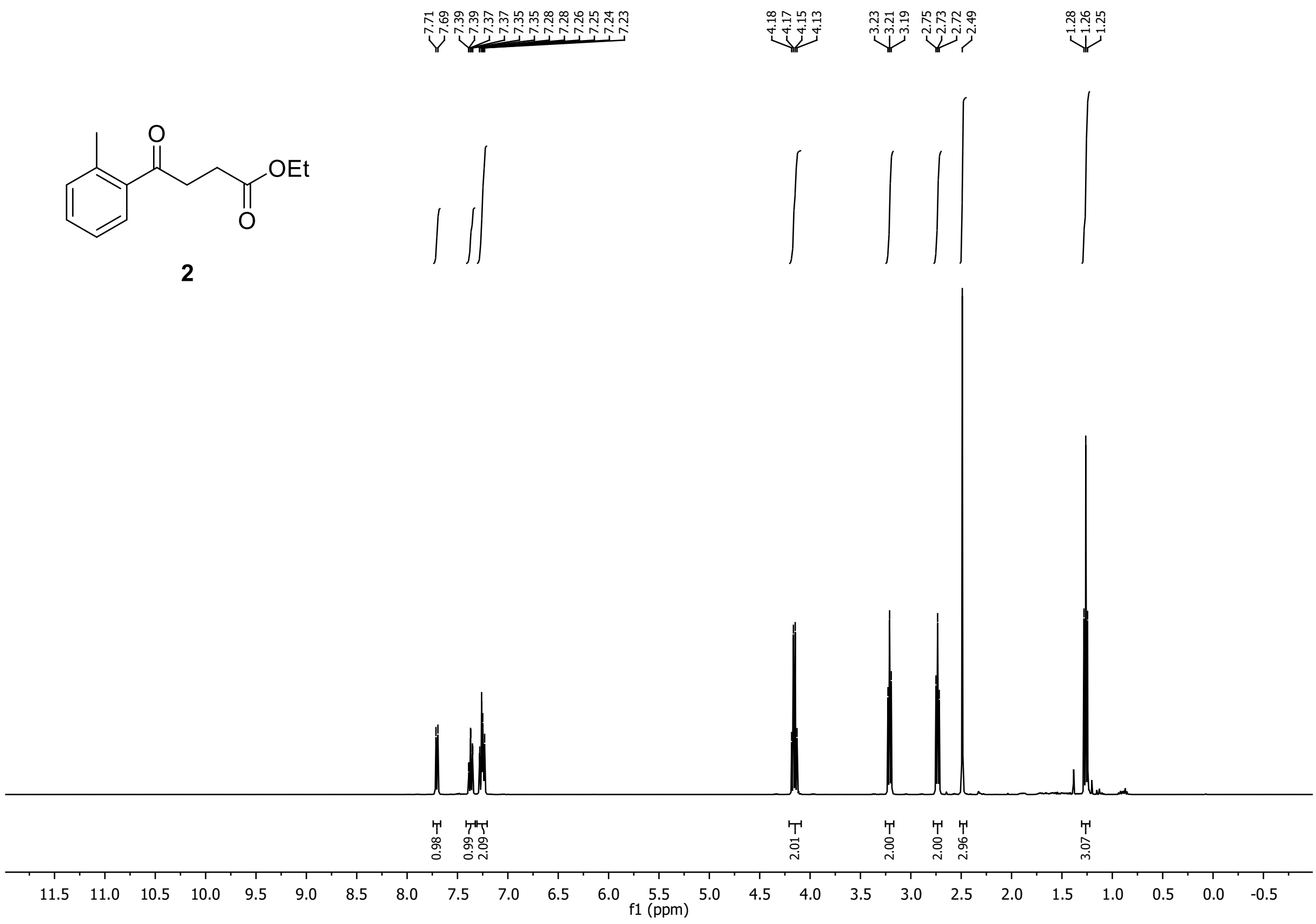

Figure S71. ^{1}H NMR spectrum (400 MHz, $CDCl_3$) of **2**.

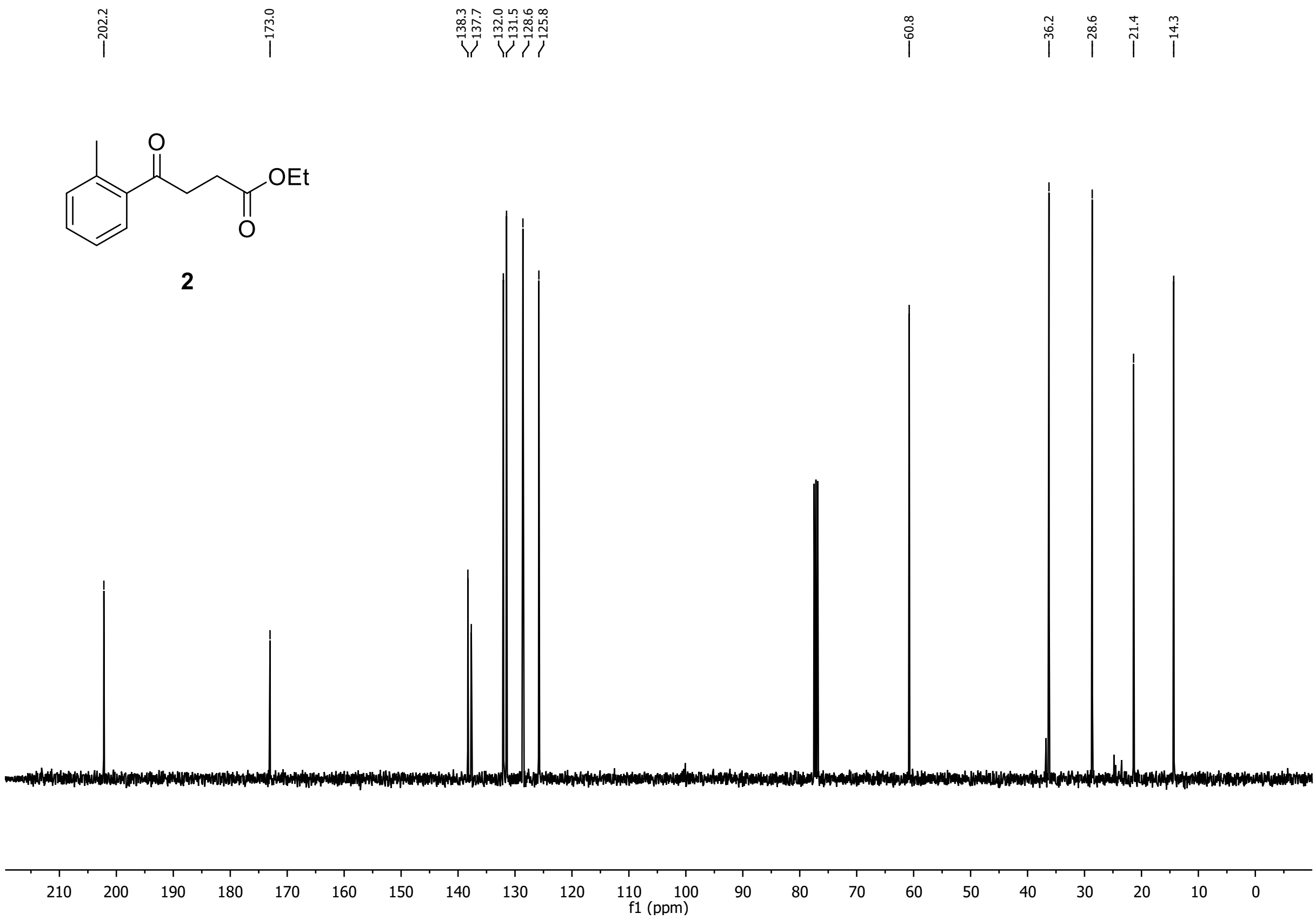

Figure S72. ^{13}C NMR spectrum (101 MHz, $CDCl_3$) of **2**.

Ethyl 4-(4-(*tert*-butyl)phenyl)-4-oxobutanoate (3):

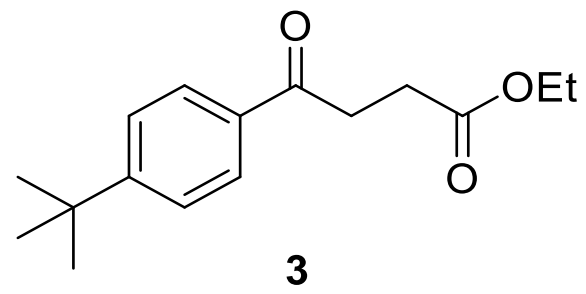

3

$C_{16}H_{22}O_3$ (262.35 g/mol)

Following **GP-B**, **3** was synthesized using **S3** (270 mg, 1.0 mmol, 1.0 equiv.) and ethyl acrylate (200 mg, 2.0 mmol, 2 equiv.). Purification by flash column chromatography (*n*-hexane/EtOAc, gradient 100:0 to 70:30 over 10 CV) afforded **3** (199 mg, 759 µmol, 76%) as colorless oil. Conforms to reported analytical data.[21]

R_f: 0.67 (*n*-hexane/EtOAc 80:20).

^{1}H NMR (400 MHz, $CDCl_3$, δ): 7.97 – 7.88 (m, 2H), 7.52 – 7.43 (m, 2H), 4.16 (q, *J* = 7.1 Hz, 2H), 3.29 (t, *J* = 6.7 Hz, 2H), 2.75 (t, *J* = 6.7 Hz, 2H), 1.34 (s, 9H), 1.26 (t, *J* = 7.1 Hz, 3H).

^{13}C{^{1}H} NMR (101 MHz, $CDCl_3$, δ): 197.9, 173.1, 157.0, 134.2, 128.1, 125.7, 60.7, 35.2, 33.4, 31.2, 28.5, 14.3.

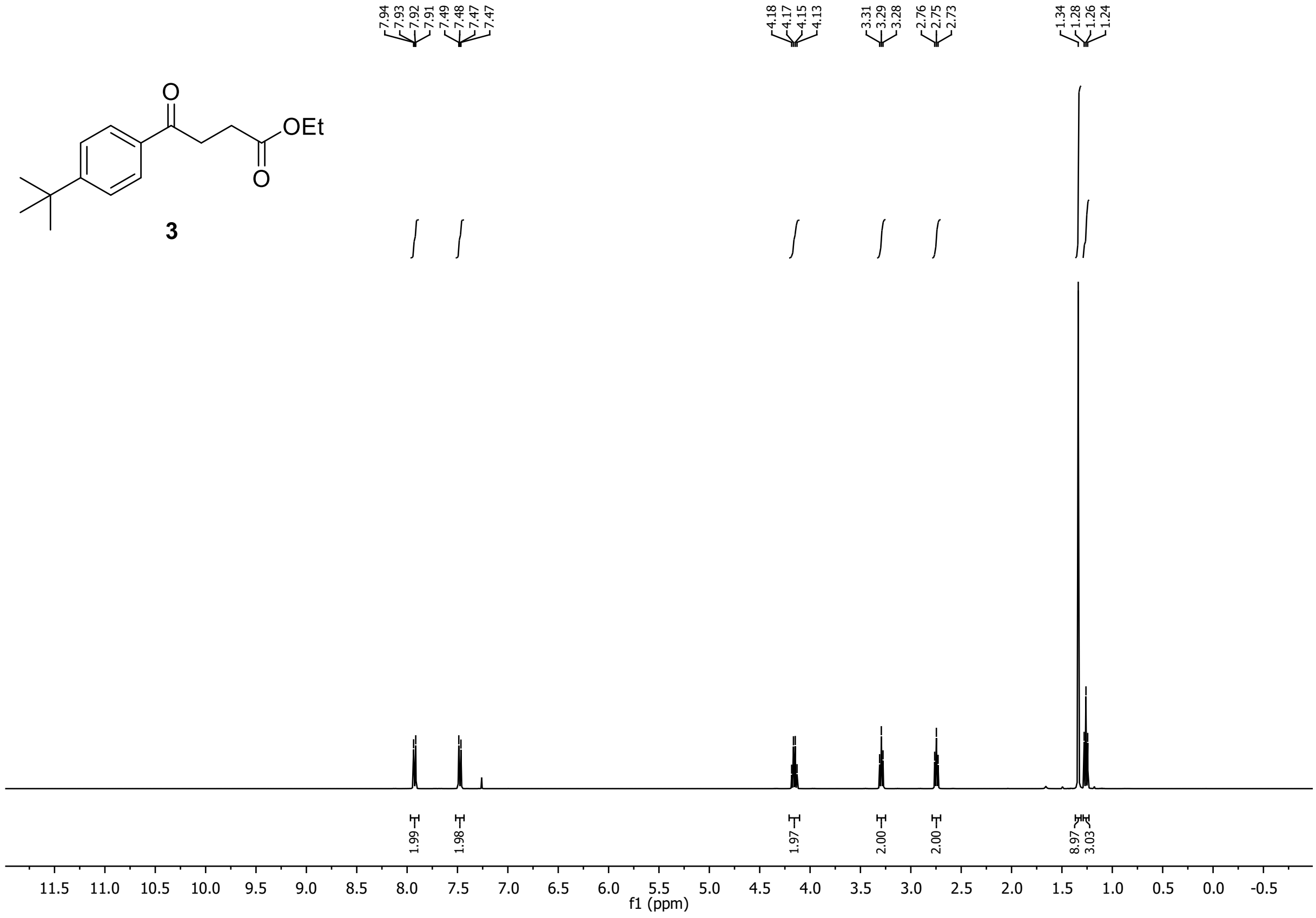

Figure S73. ^{1}H NMR spectrum (400 MHz, $CDCl_3$) of **3**.

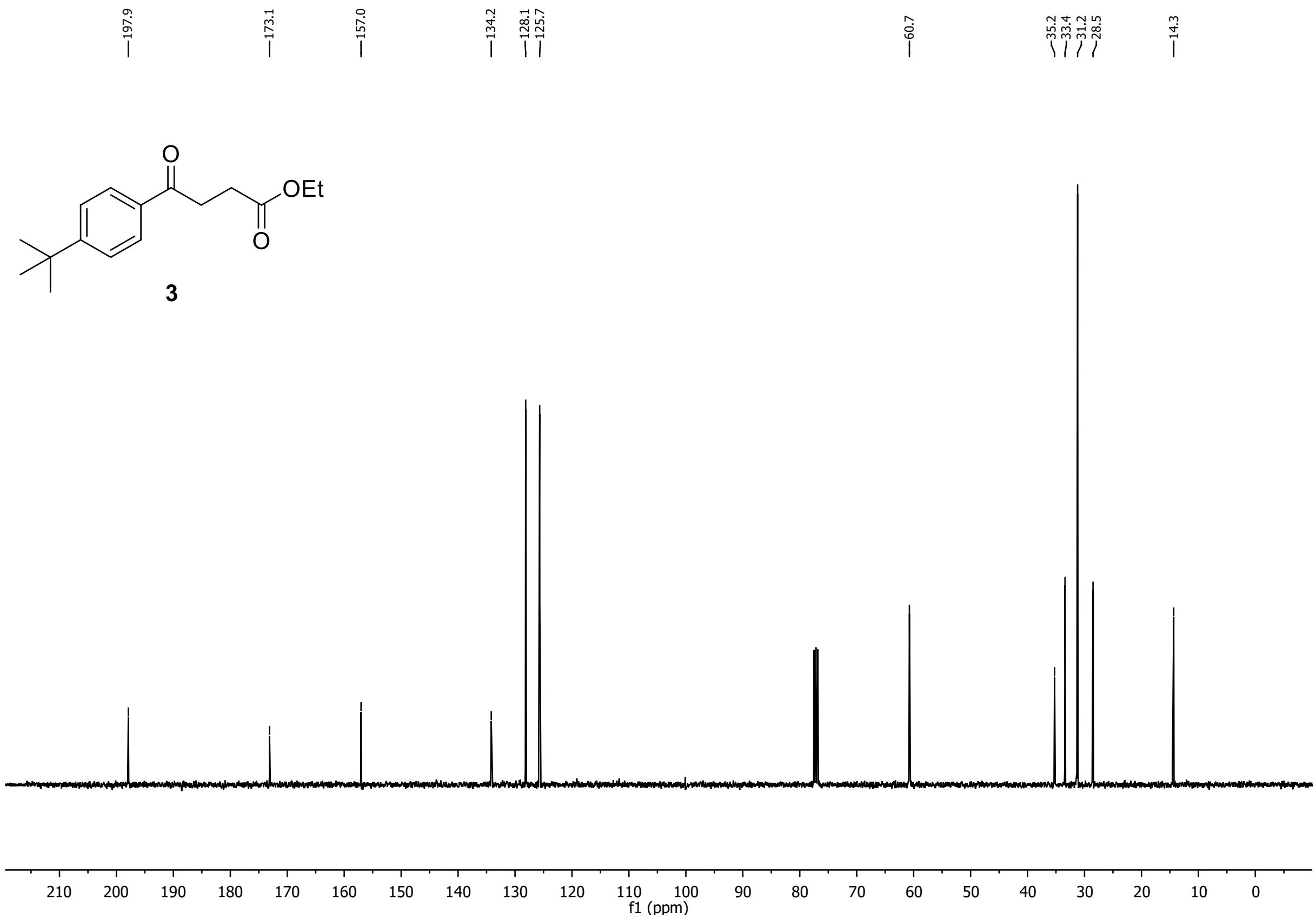

Figure S74. ^{13}C NMR spectrum (101 MHz, $CDCl_3$) of **3**.

Ethyl 4-(naphthalen-2-yl)-4-oxobutanoate (4):

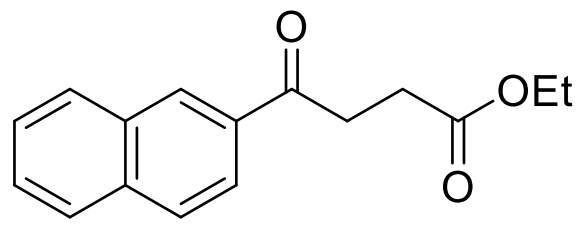

4

$C_{16}H_{16}O_3$ (256.30 g/mol)

Following **GP-B**, **4** was synthesized using **S4** (264 mg, 1.0 mmol, 1.0 equiv.) and ethyl acrylate (200 mg, 2.0 mmol, 2 equiv.). Purification by flash column chromatography (*n*-hexane/EtOAc, gradient 100:0 to 70:30 over 10 CV) afforded **4** (131 mg, 511 µmol, 51%) as colorless oil. Conforms to reported analytical data.[19]

R_f: 0.51 (*n*-hexane/EtOAc 80:20).

^{1}H NMR (400 MHz, $CDCl_3$, δ): 8.54 – 8.48 (m, 1H), 8.04 (dd, *J* = 8.6, 1.7 Hz, 1H), 8.00 – 7.84 (m, 3H), 7.64 – 7.51 (m, 2H), 4.18 (q, *J* = 7.1 Hz, 2H), 3.45 (t, *J* = 6.7 Hz, 2H), 2.82 (t, *J* = 6.7 Hz, 2H), 1.28 (t, *J* = 7.1 Hz, 3H).

^{13}C{^{1}H} NMR (101 MHz, $CDCl_3$, δ): 198.2, 173.1, 135.8, 134.0, 132.6, 129.9, 129.7, 128.6, 128.6, 127.9, 126.9, 123.9, 60.8, 33.6, 28.5, 14.3.

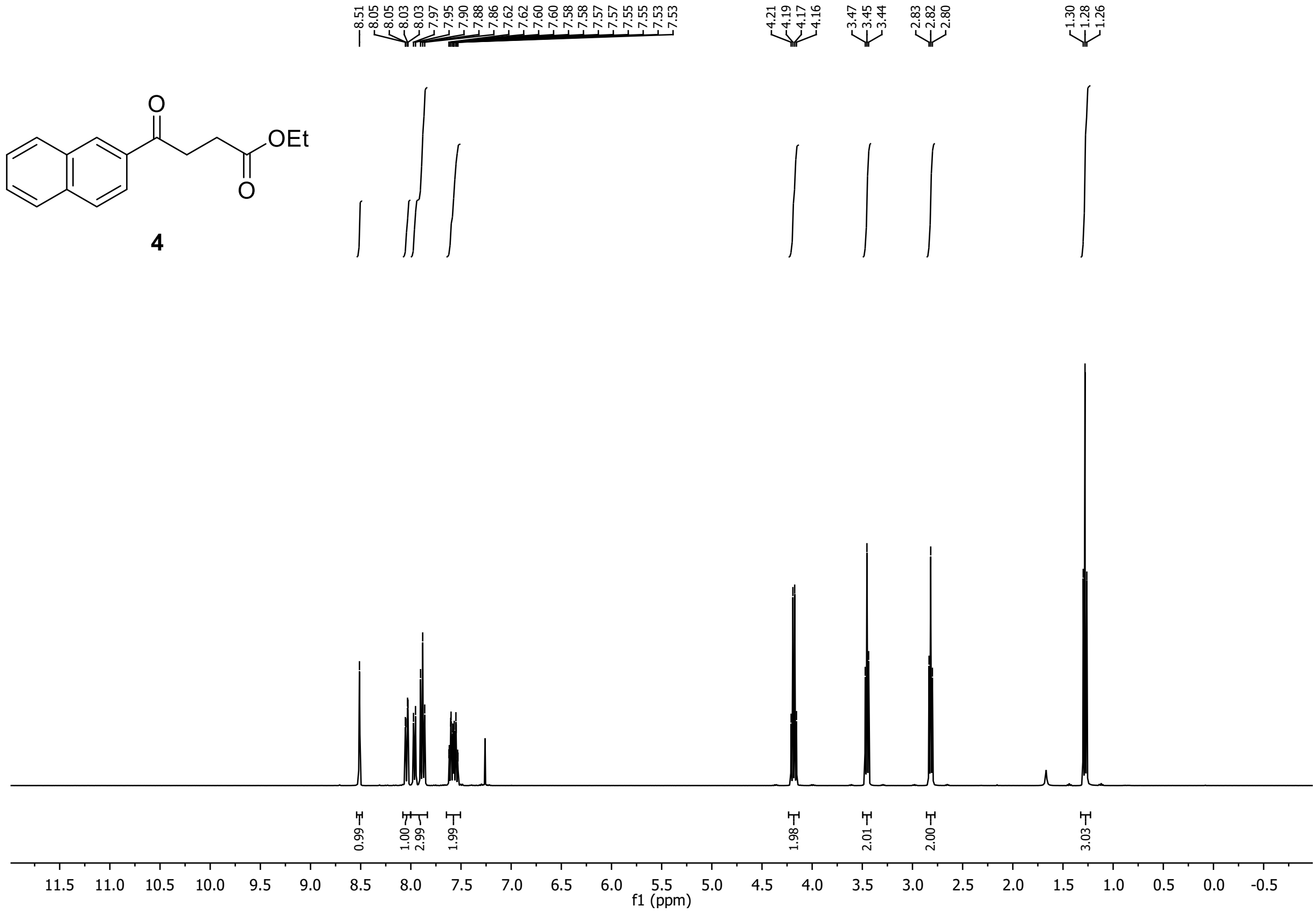

Figure S75. ^{1}H NMR spectrum (400 MHz, $CDCl_3$) of **4**.

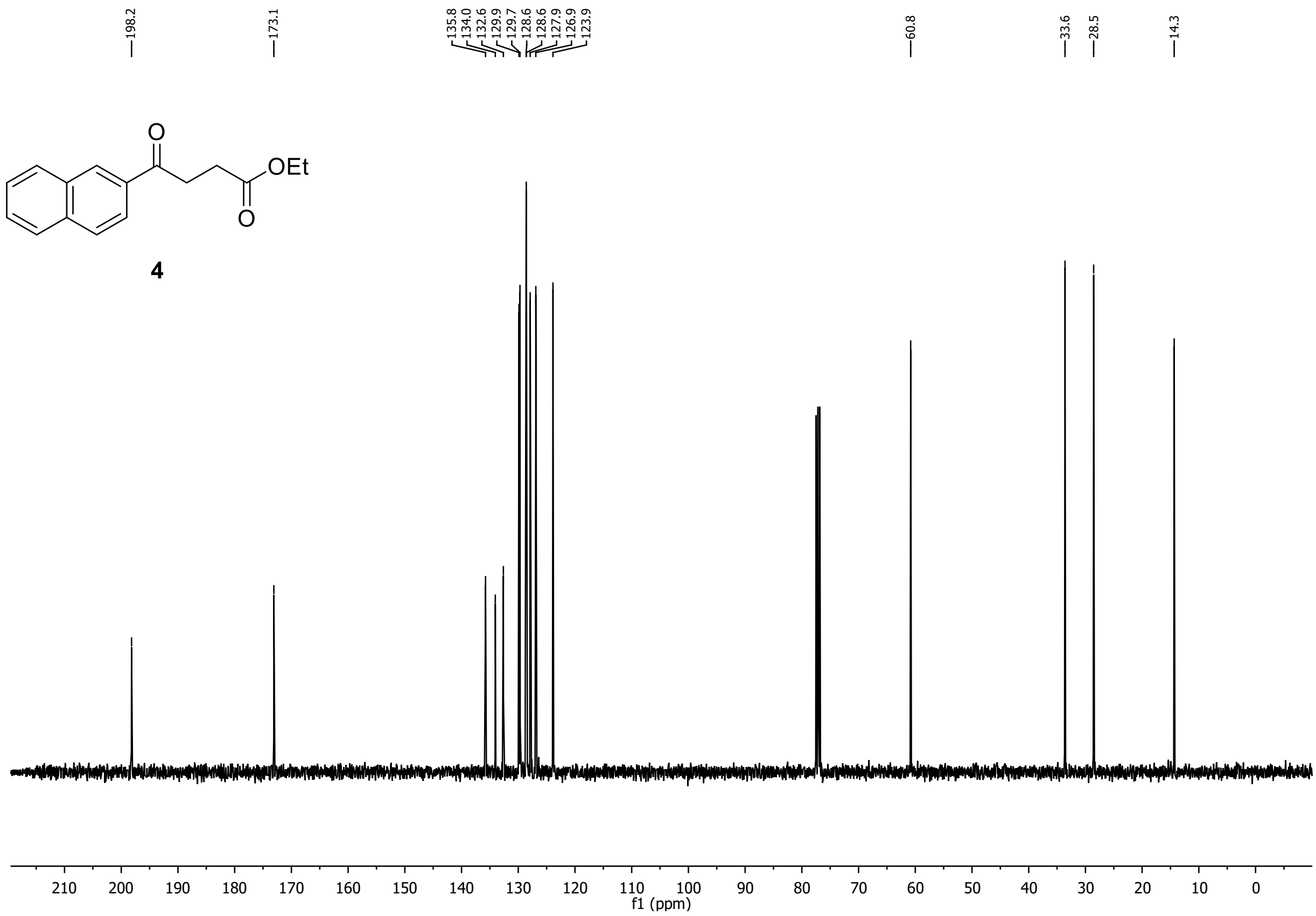

Figure S76. ^{13}C NMR spectrum (101 MHz, $CDCl_3$) of **4**.

Ethyl 4-(3-chlorophenyl)-4-oxobutanoate (5):

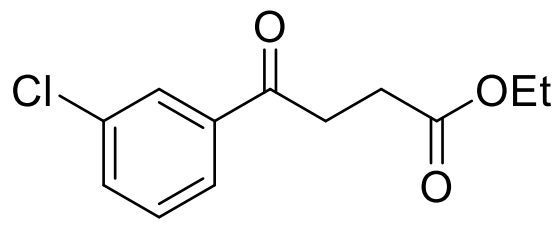

5

$C_{12}H_{13}ClO_3$ (240.68 g/mol)

Following **GP-B**, **5** was synthesized using **S5** (249 mg, 1.0 mmol, 1.0 equiv.) and ethyl acrylate (200 mg, 2.0 mmol, 2 equiv.). Purification by flash column chromatography (*n*-hexane/EtOAc, gradient 100:0 to 70:30 over 10 CV) afforded **5** (152 mg, 632 µmol, 63%) as colorless oil. Conforms to reported analytical data.[19]

R_f: 0.54 (*n*-hexane/EtOAc 80:20).

^{1}H NMR (400 MHz, $CDCl_3$, δ): 7.97 – 7.91 (m, 1H), 7.89 – 7.81 (m, 1H), 7.57 – 7.49 (m, 1H), 7.40 (dd, *J* = 7.9, 7.8 Hz, 1H), 4.15 (q, *J* = 7.1 Hz, 2H), 3.27 (t, *J* = 6.6 Hz, 2H), 2.75 (t, *J* = 6.6 Hz, 2H), 1.26 (t, *J* = 7.1 Hz, 3H).

^{13}C{^{1}H} NMR (101 MHz, $CDCl_3$, δ): 197.0, 172.8, 138.2, 135.1, 133.2, 130.1, 128.3, 126.2, 60.9, 33.6, 28.3, 14.3.

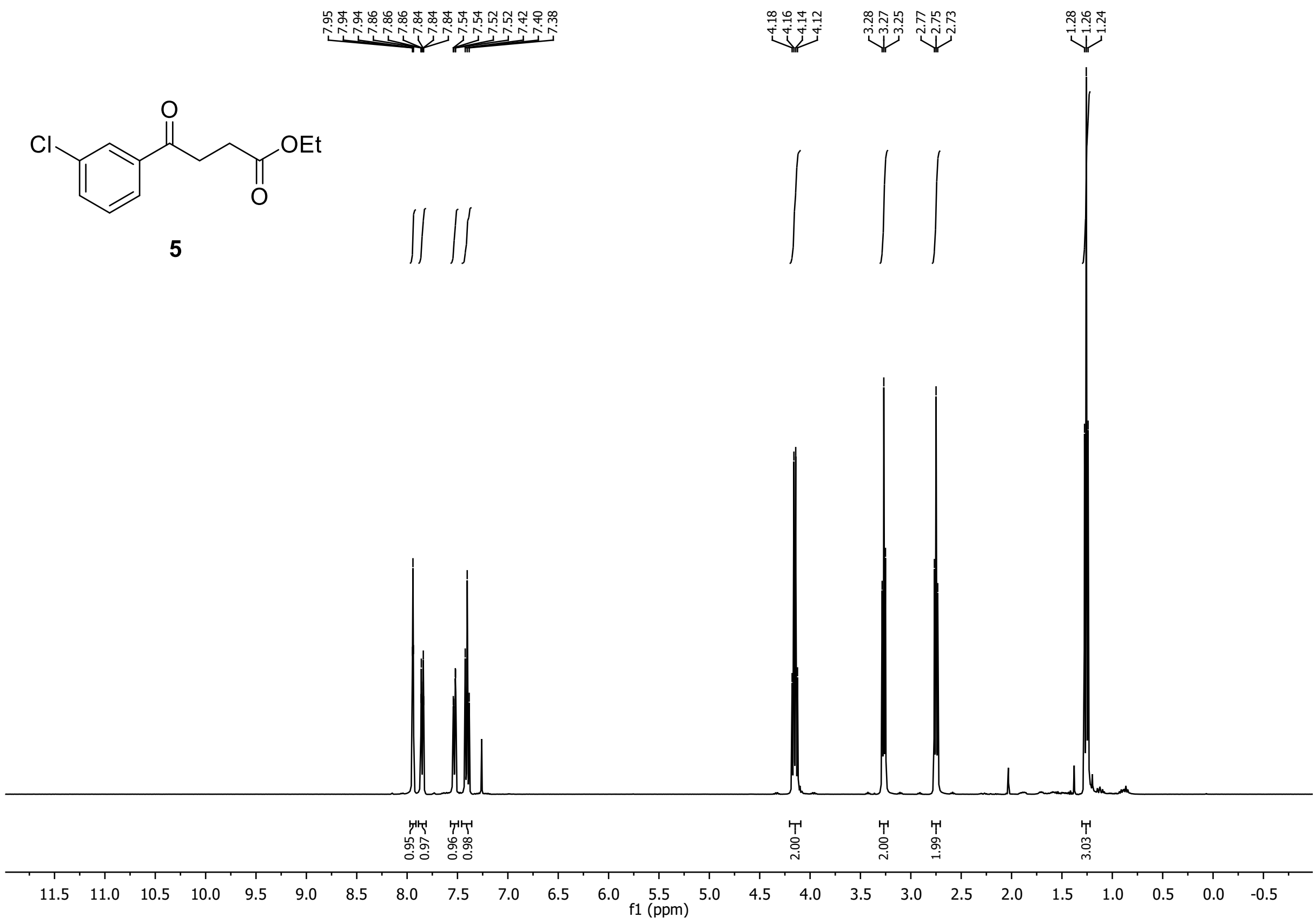

Figure S77. ^{1}H NMR spectrum (400 MHz, $CDCl_3$) of **5**.

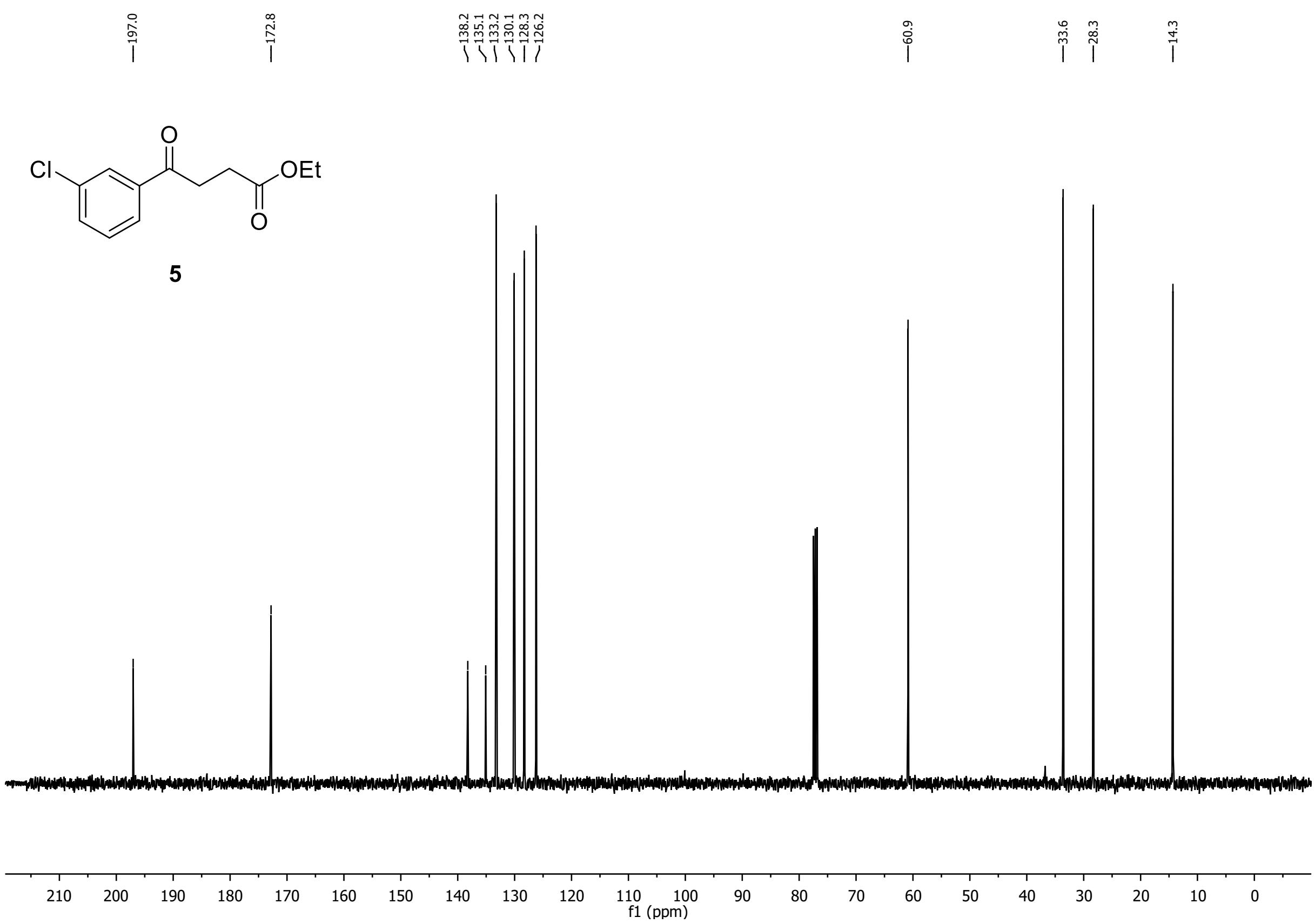

Figure S78. ^{13}C NMR spectrum (101 MHz, $CDCl_3$) of **5**.

Ethyl 4-(3-bromophenyl)-4-oxobutanoate (6):

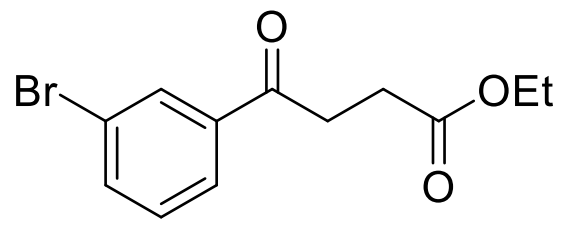

6

$C_{12}H_{13}BrO_3$ (285.14 g/mol)

Following **GP-B**, **6** was synthesized using **S6** (293 mg, 1.0 mmol, 1.0 equiv.) and ethyl acrylate (200 mg, 2.0 mmol, 2 equiv.). Purification by flash column chromatography (*n*-hexane/EtOAc, gradient 100:0 to 70:30 over 10 CV) afforded **6** (165 mg, 579 µmol, 58%) as colorless oil. Conforms to reported analytical data.[19]

R_f: 0.53 (*n*-hexane/EtOAc 80:20).

^{1}H NMR (400 MHz, $CDCl_3$, δ): 8.13 – 8.07 (m, 1H), 7.93 – 7.86 (m, 1H), 7.72 – 7.65 (m, 1H), 7.34 (dd, *J* = 7.9, 7.8 Hz, 1H), 4.15 (q, *J* = 7.1 Hz, 2H), 3.26 (t, *J* = 6.6 Hz, 2H), 2.75 (t, *J* = 6.6 Hz, 2H), 1.26 (t, *J* = 7.1 Hz, 3H).

^{13}C{^{1}H} NMR (101 MHz, $CDCl_3$, δ): 196.9, 172.8, 138.4, 136.2, 131.3, 130.3, 126.7, 123.1, 60.9, 33.6, 28.3, 14.3.

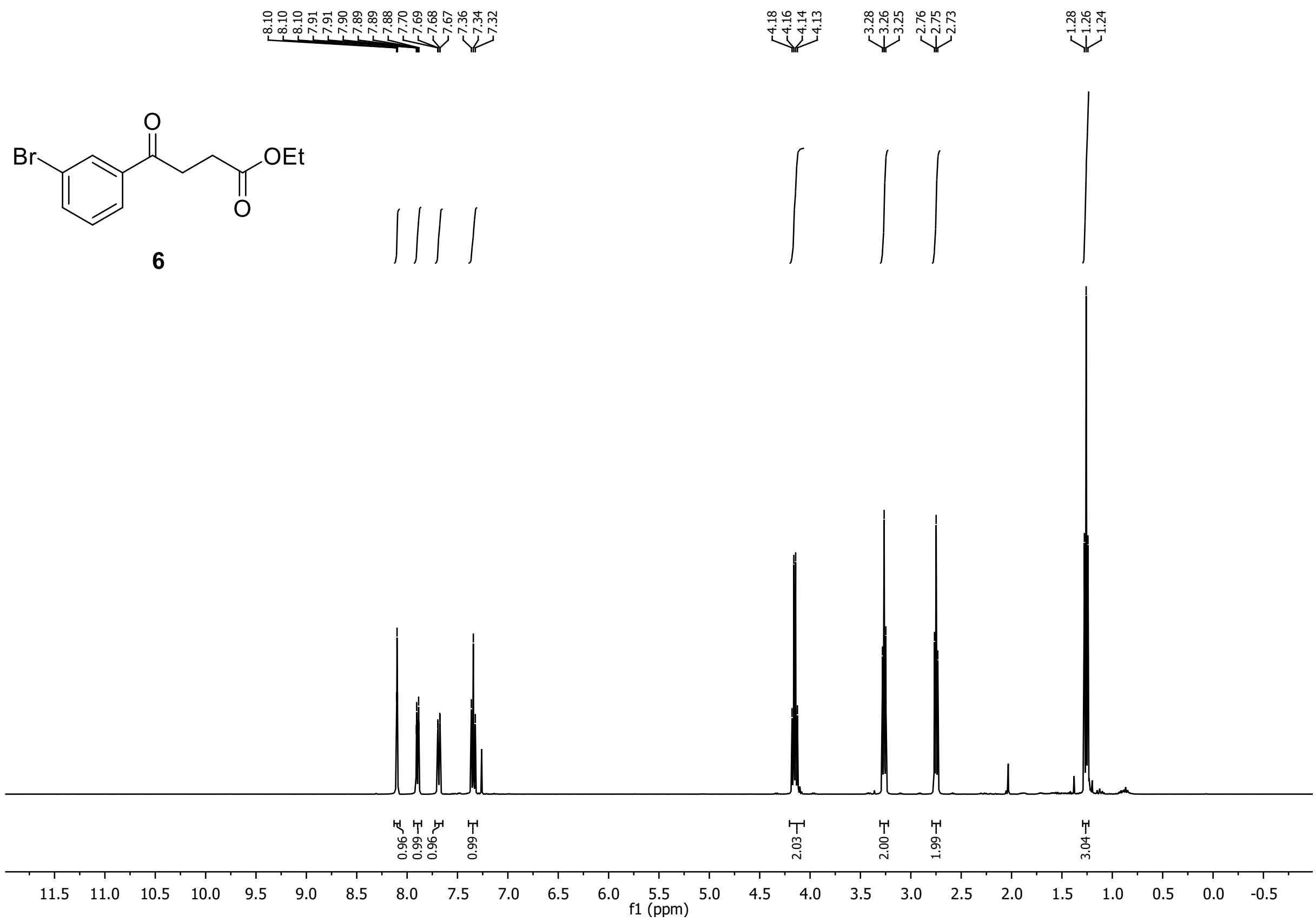

Figure S79. ^{1}H NMR spectrum (400 MHz, $CDCl_3$) of **6**.

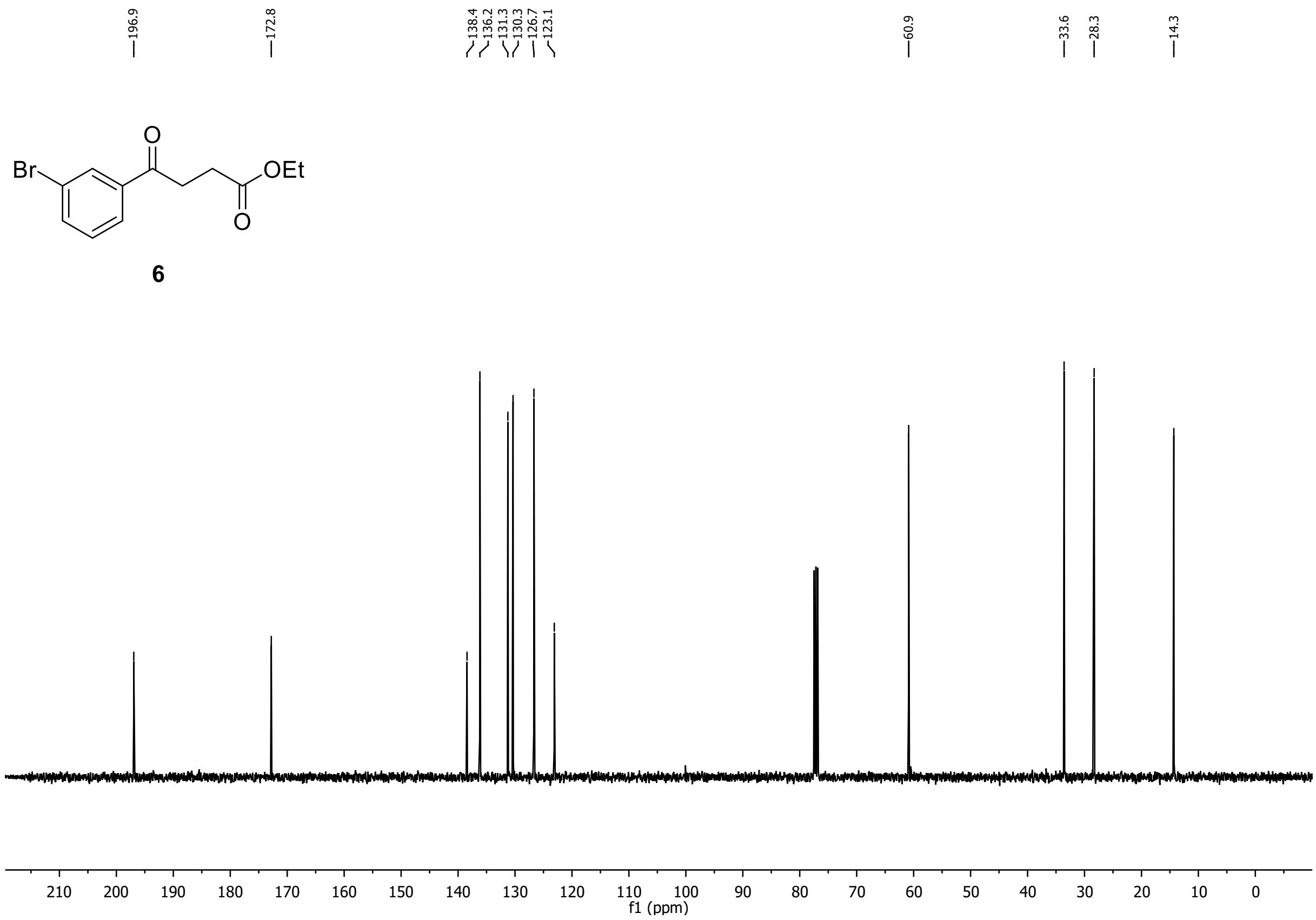

Figure S80. ^{13}C NMR spectrum (101 MHz, $CDCl_3$) of **6**.

Ethyl 4-oxo-4-(4-(trifluoromethyl)phenyl)butanoate (7):

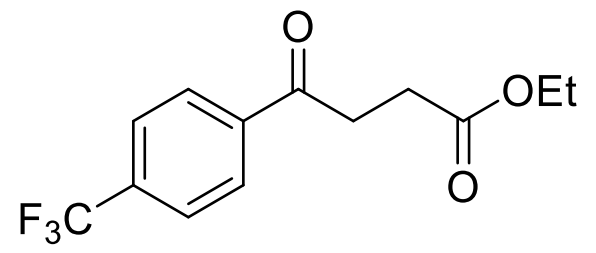

7

$C_{13}H_{13}F_3O_3$ (274.24 g/mol)

Following **GP-B**, **7** was synthesized using **S7** (282 mg, 1.0 mmol, 1.0 equiv.) and ethyl acrylate (200 mg, 2.0 mmol, 2 equiv.). Purification by flash column chromatography (*n*-hexane/EtOAc, gradient 100:0 to 70:30 over 10 CV) afforded **7** (134 mg, 489 µmol, 49%) as viscous colorless oil. Conforms to reported analytical data.[22]

R$_f$: 0.55 (*n*-hexane/EtOAc 80:20).

^{1}H NMR (400 MHz, $CDCl_3$, δ): δ 8.09 (d, J = 8.2 Hz, 2H), 7.74 (d, J = 8.2 Hz, 2H), 4.16 (q, J = 7.1 Hz, 2H), 3.32 (t, J = 6.5 Hz, 2H), 2.78 (t, J = 6.5 Hz, 2H), 1.27 (t, J = 7.1 Hz, 3H).

^{13}C{^{1}H} NMR (101 MHz, $CDCl_3$, δ): 197.4, 172.8, 139.4, 134.7 (q, J = 32.7 Hz), 128.5, 125.9 (q, J = 3.7 Hz), 123.7 (q, J = 272.6 Hz), 60.9, 33.8, 28.3, 14.3.

^{19}F NMR (376 MHz, $CDCl_3$, δ): -63.1.

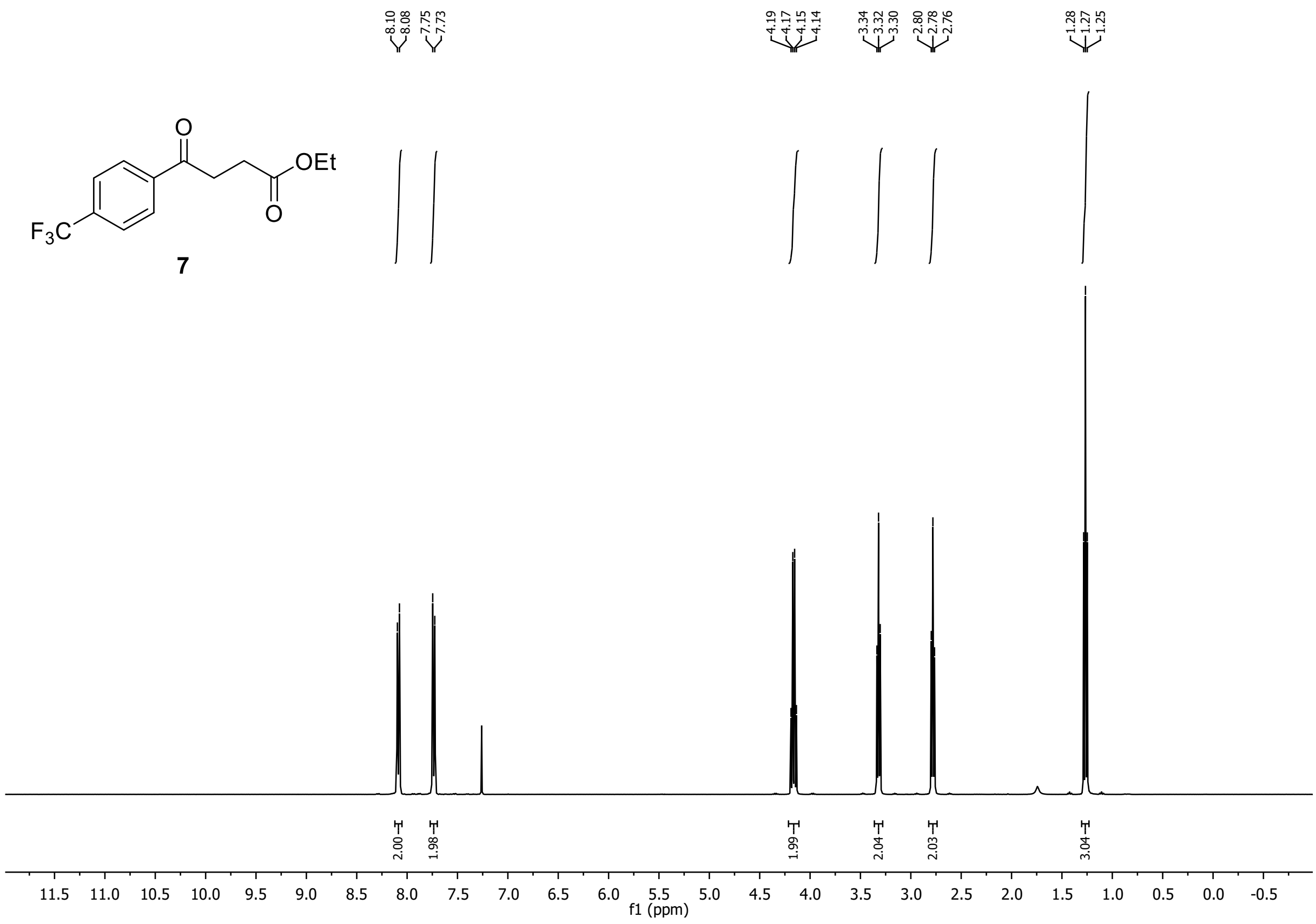

Figure S81. ^{1}H NMR spectrum (400 MHz, $CDCl_3$) of **7**.

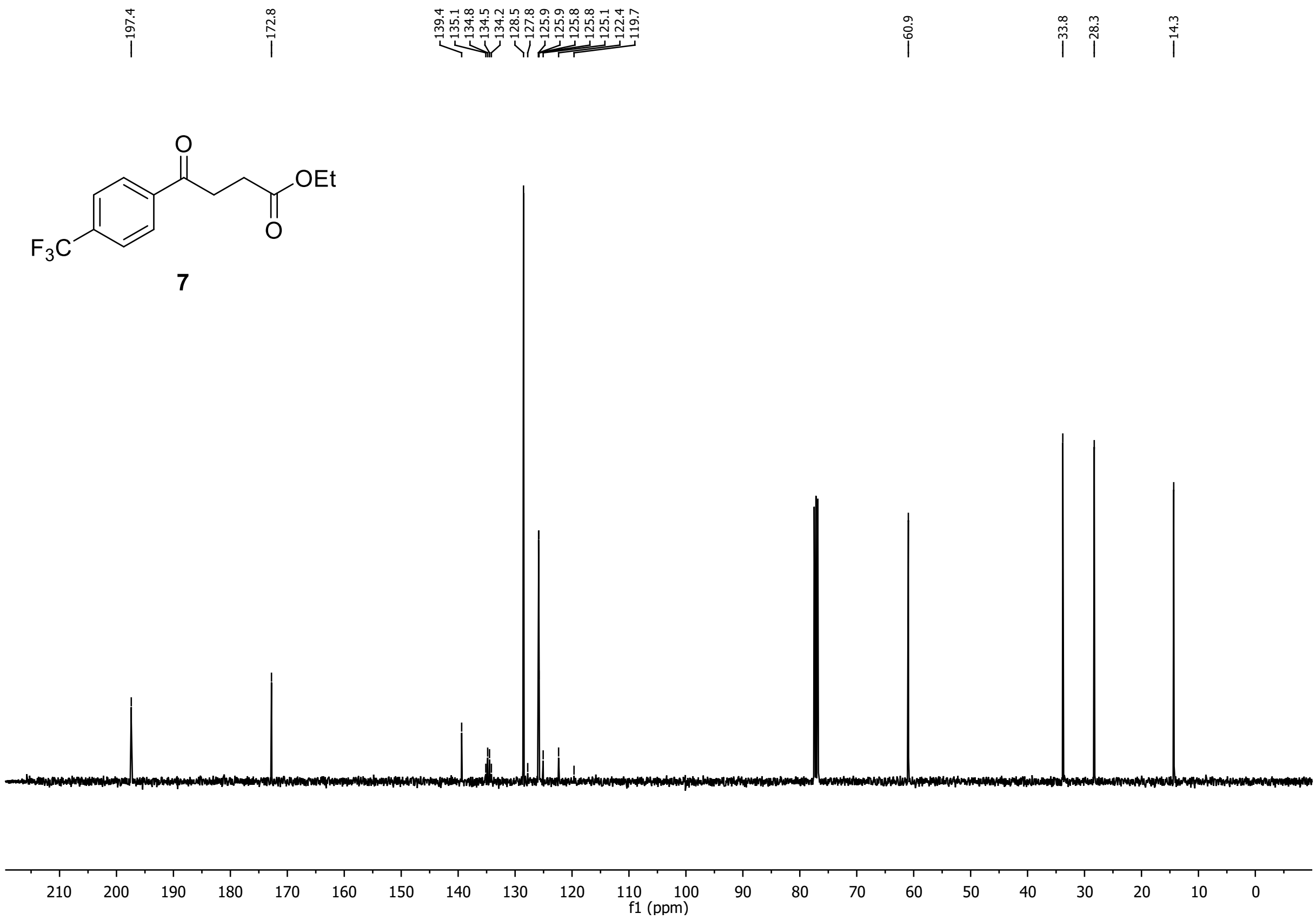

Figure S82. ^{13}C NMR spectrum (101 MHz, $CDCl_3$) of **7**.

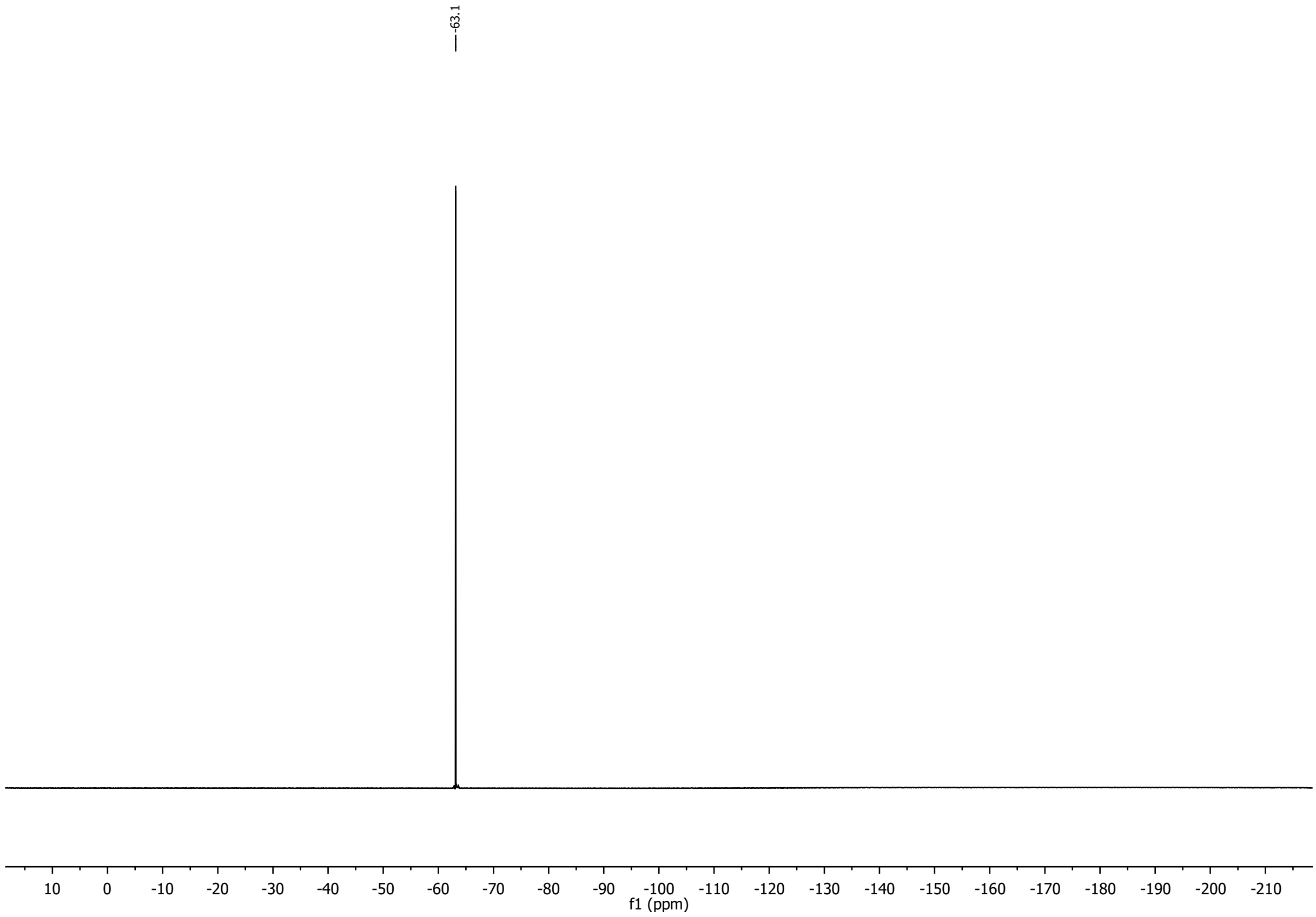

Figure S83. ^{19}F NMR spectrum (376 MHz, $CDCl_3$) of **7**.

Ethyl 4-(4-methoxyphenyl)-4-oxobutanoate (8):

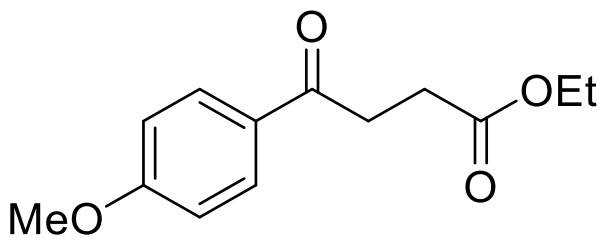

8

$C_{13}H_{16}O_4$ (236.27 g/mol)

Following **GP-B**, **8** was synthesized using **S8** (244 mg, 1.0 mmol, 1.0 equiv.) and ethyl acrylate (200 mg, 2.0 mmol, 2 equiv.). Purification by flash column chromatography (*n*-hexane/EtOAc, gradient 100:0 to 60:40 over 10 CV) afforded **8** (186 mg, 787 µmol, 79%) as colorless oil. Conforms to reported analytical data.[19]

R$_f$: 0.35 (*n*-hexane/EtOAc 80:20).

^{1}H NMR (400 MHz, $CDCl_3$, δ): 8.01 – 7.92 (m, 2H), 6.98 – 6.89 (m, 2H), 4.15 (q, *J* = 7.1 Hz, 2H), 3.86 (s, 3H), 3.26 (t, *J* = 6.7 Hz, 2H), 2.74 (t, *J* = 6.7 Hz, 2H), 1.26 (t, *J* = 7.1 Hz, 3H).

^{13}C{^{1}H} NMR (101 MHz, $CDCl_3$, δ): 196.8, 173.2, 163.7, 130.4, 129.9, 113.9, 60.7, 55.6, 33.2, 28.5, 14.3.

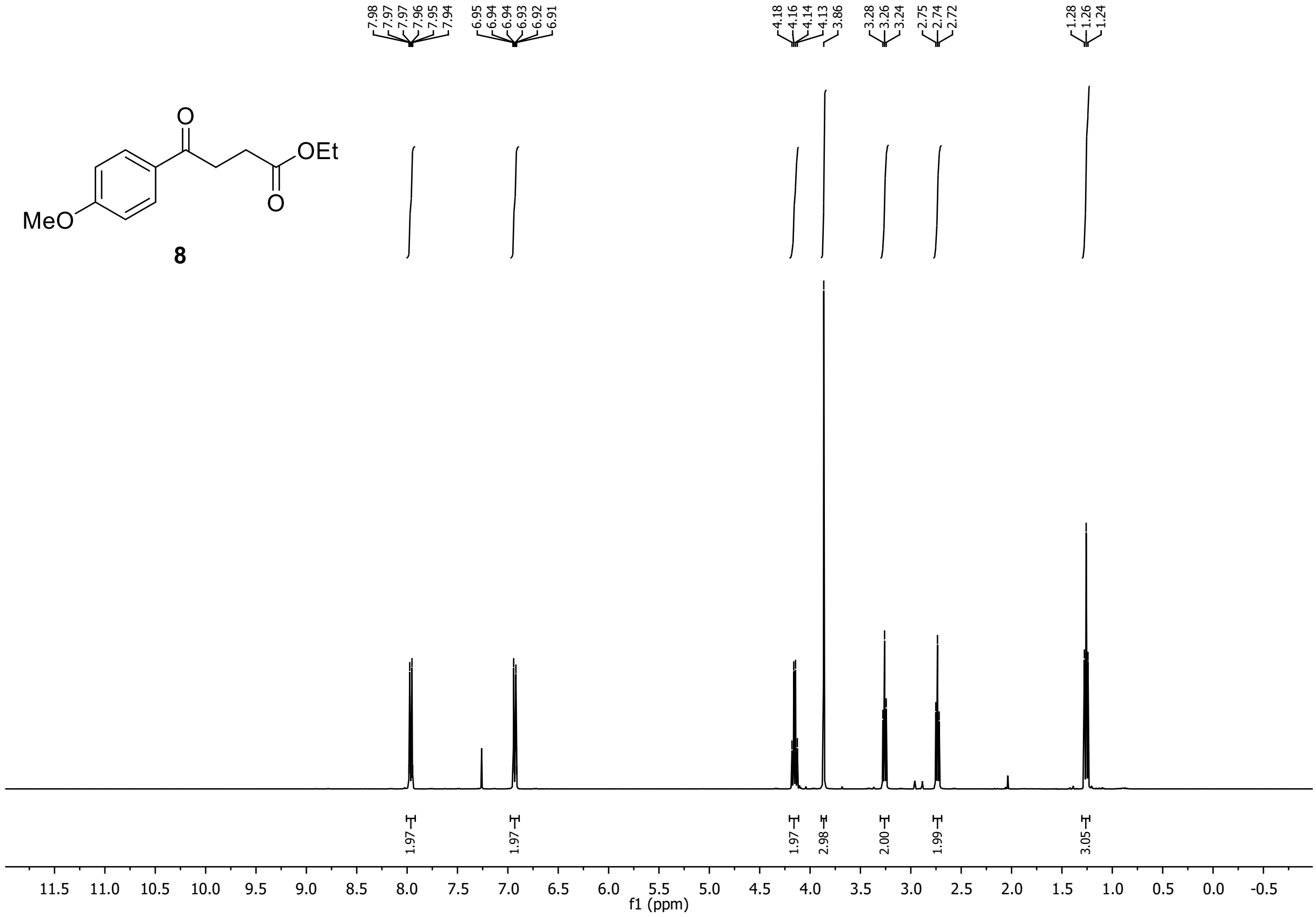

Figure S84. ^{1}H NMR spectrum (400 MHz, $CDCl_3$) of **8**.

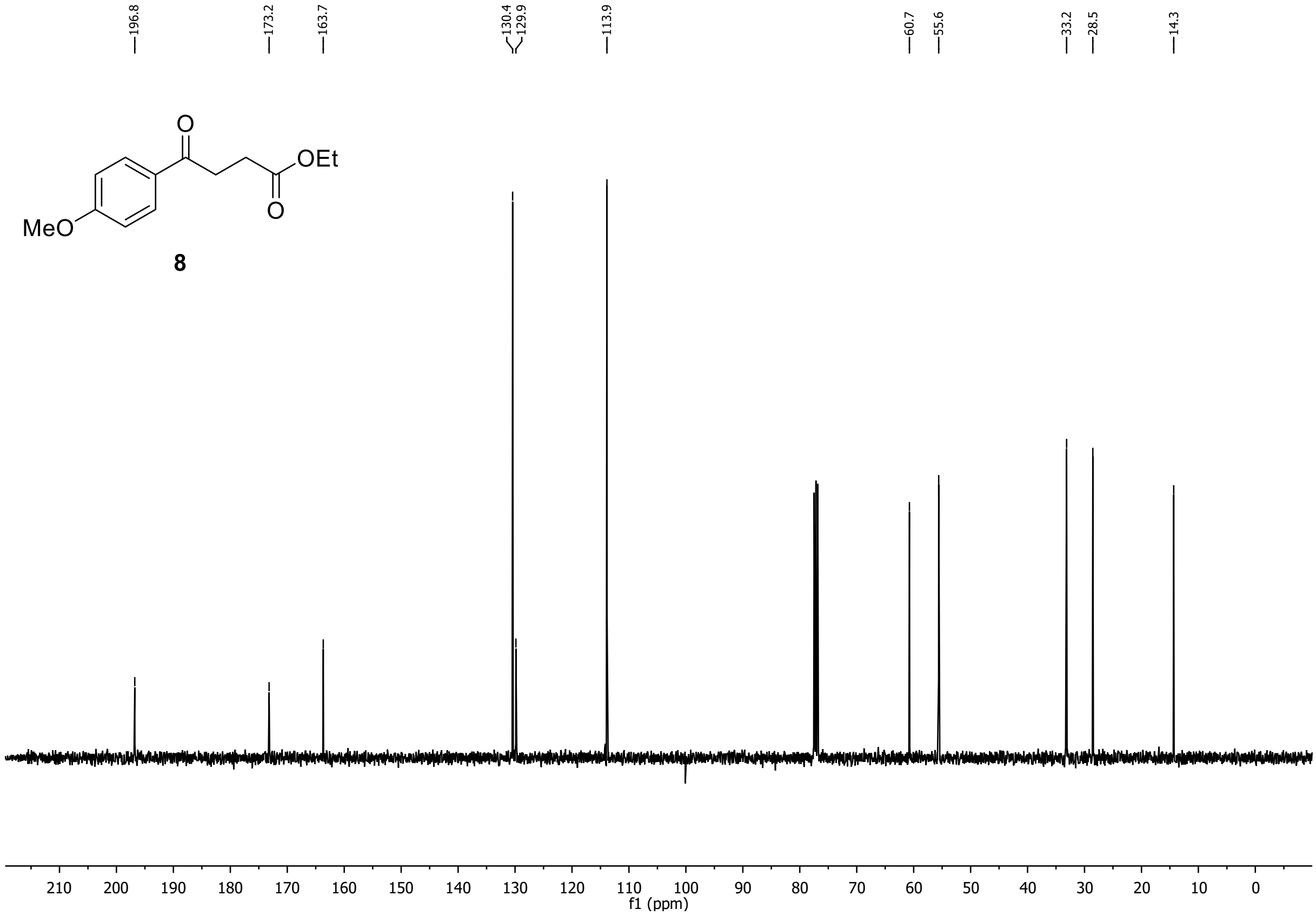

Figure S85. ^{13}C NMR spectrum (101 MHz, $CDCl_3$) of **8**.

Ethyl 4-(benzo[*d*][1,3]dioxol-5-yl)-4-oxobutanoate (9):

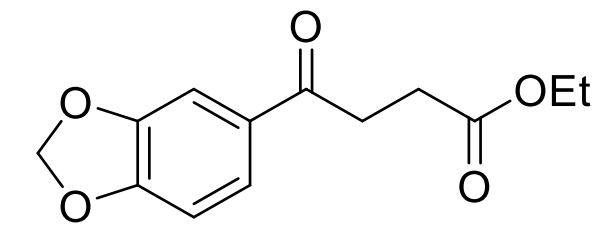

9

$C_{13}H_{14}O_5$ (250.25 g/mol)

Following **GP-B**, **9** was synthesized using **S9** (258 mg, 1.0 mmol, 1.0 equiv.) and ethyl acrylate (200 mg, 2.0 mmol, 2 equiv.). Purification by flash column chromatography (*n*-hexane/EtOAc, gradient 100:0 to 50:50 over 10 CV) afforded **9** (211 mg, 843 µmol, 84%) as colorless oil. Conforms to reported analytical data.[21]

R_f: 0.77 (*n*-hexane/EtOAc 60:40).

^{1}H NMR (400 MHz, $CDCl_3$, δ): 7.58 (dd, *J* = 8.1, 1.7 Hz, 1H), 7.44 (d, *J* = 1.7 Hz, 1H), 6.84 (d, *J* = 8.1 Hz, 1H), 6.03 (s, 2H), 4.15 (q, *J* = 7.1 Hz, 2H), 3.22 (t, *J* = 6.7 Hz, 2H), 2.72 (t, *J* = 6.7 Hz, 2H), 1.25 (t, *J* = 7.1 Hz, 3H).

^{13}C{^{1}H} NMR (101 MHz, $CDCl_3$, δ): 196.3, 173.1, 151.9, 148.3, 131.6, 124.4, 108.00, 107.96, 102.0, 60.7, 33.2, 28.5, 14.3.

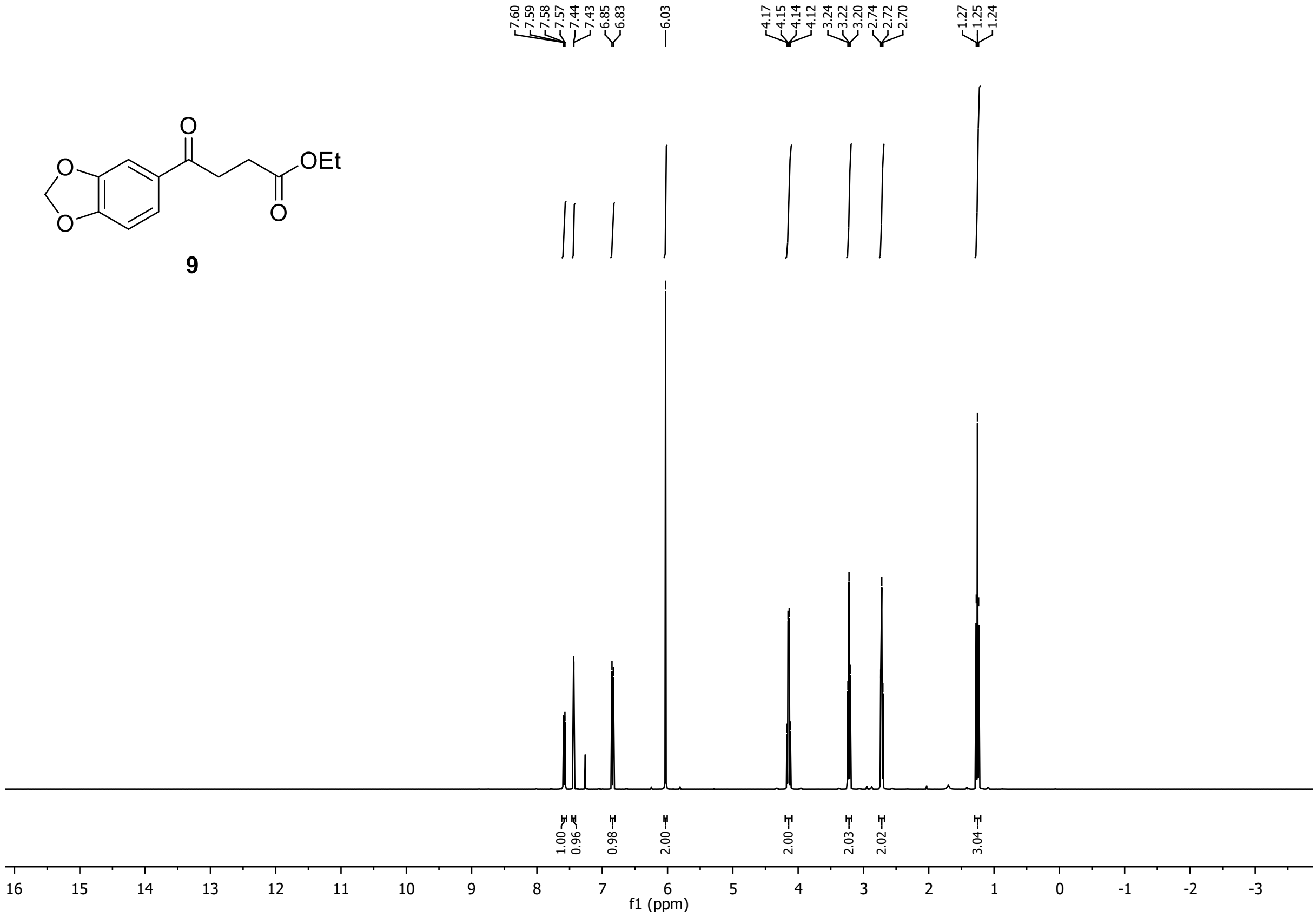

Figure S86. ^{1}H NMR spectrum (400 MHz, $CDCl_3$) of **9**.

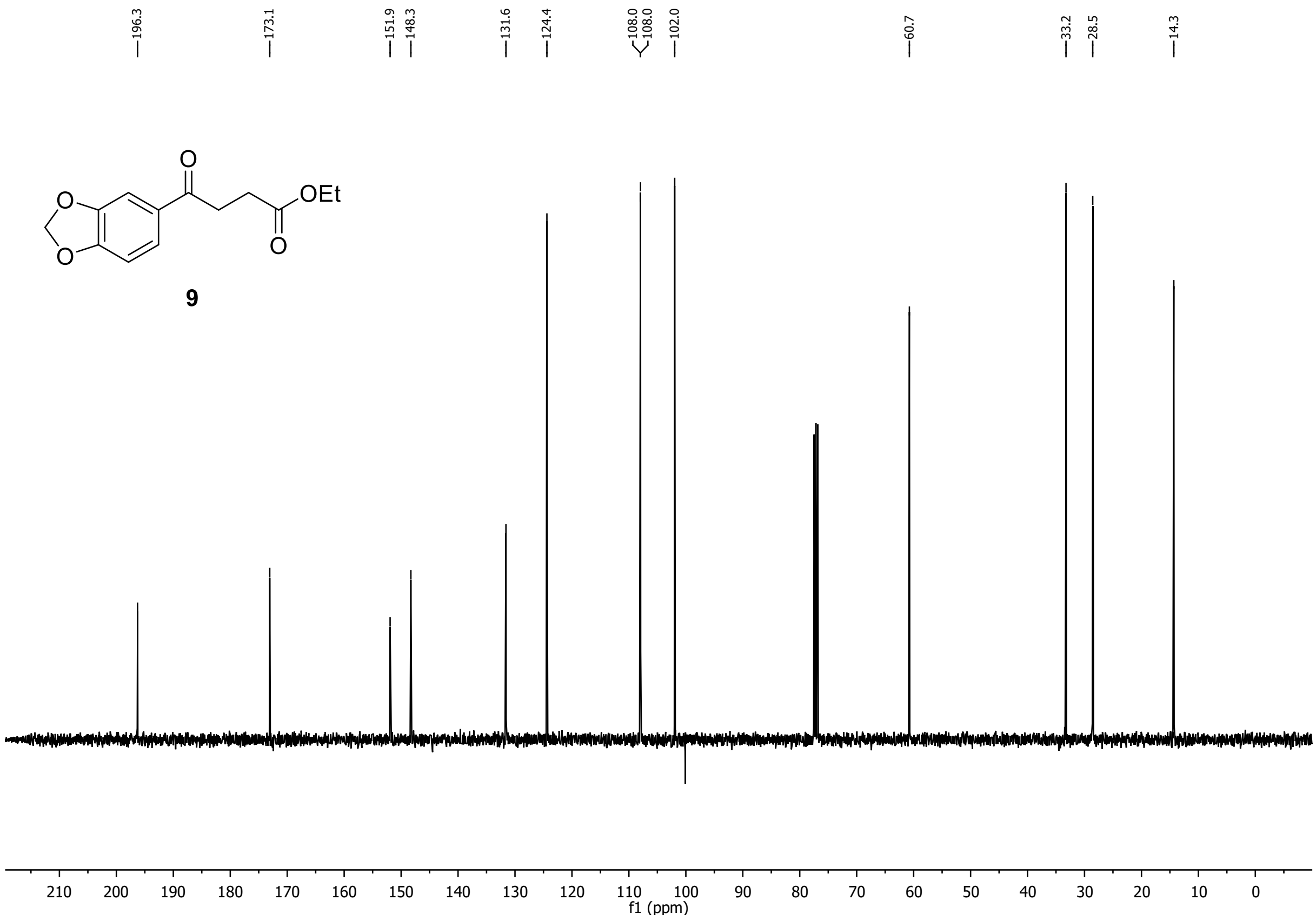

Figure S87. ^{13}C NMR spectrum (101 MHz, $CDCl_3$) of **9**.

Ethyl 4-(6-chloropyridin-3-yl)-4-oxobutanoate (10):

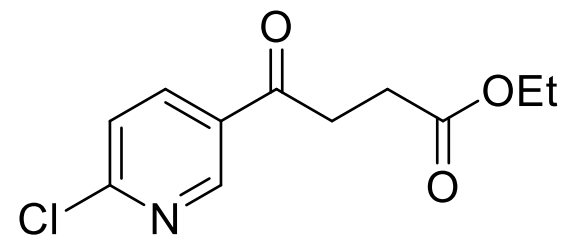

10

$C_{11}H_{12}ClNO_3$ (241.67 g/mol)

Following **GP-B**, **10** was synthesized using **S10** (250 mg, 1.0 mmol, 1.0 equiv.) and ethyl acrylate (200 mg, 2.0 mmol, 2 equiv.). Purification by flash column chromatography (*n*-hexane/EtOAc, gradient 60:40 to 0:100 over 10 CV) afforded **10** (73 mg, 302 µmol, 30%) as yellow solid. Conforms to reported analytical data.[23]

R$_f$: 0.85 (*n*-hexane/EtOAc 40:60).

^{1}H NMR (400 MHz, $CDCl_3$, δ): 8.97 (d, *J* = 2.5 Hz, 1H), 8.21 (dd, *J* = 8.3, 2.5 Hz, 1H), 7.45 (d, *J* = 8.3 Hz, 1H), 4.16 (q, *J* = 7.1 Hz, 2H), 3.27 (t, *J* = 6.5 Hz, 2H), 2.78 (t, *J* = 6.5 Hz, 2H), 1.26 (t, *J* = 7.1 Hz, 3H).

^{13}C{^{1}H} NMR (101 MHz, $CDCl_3$, δ): 196.0, 172.6, 155.9, 150.0, 130.9, 124.7, 61.0, 33.8, 28.1, 14.3.

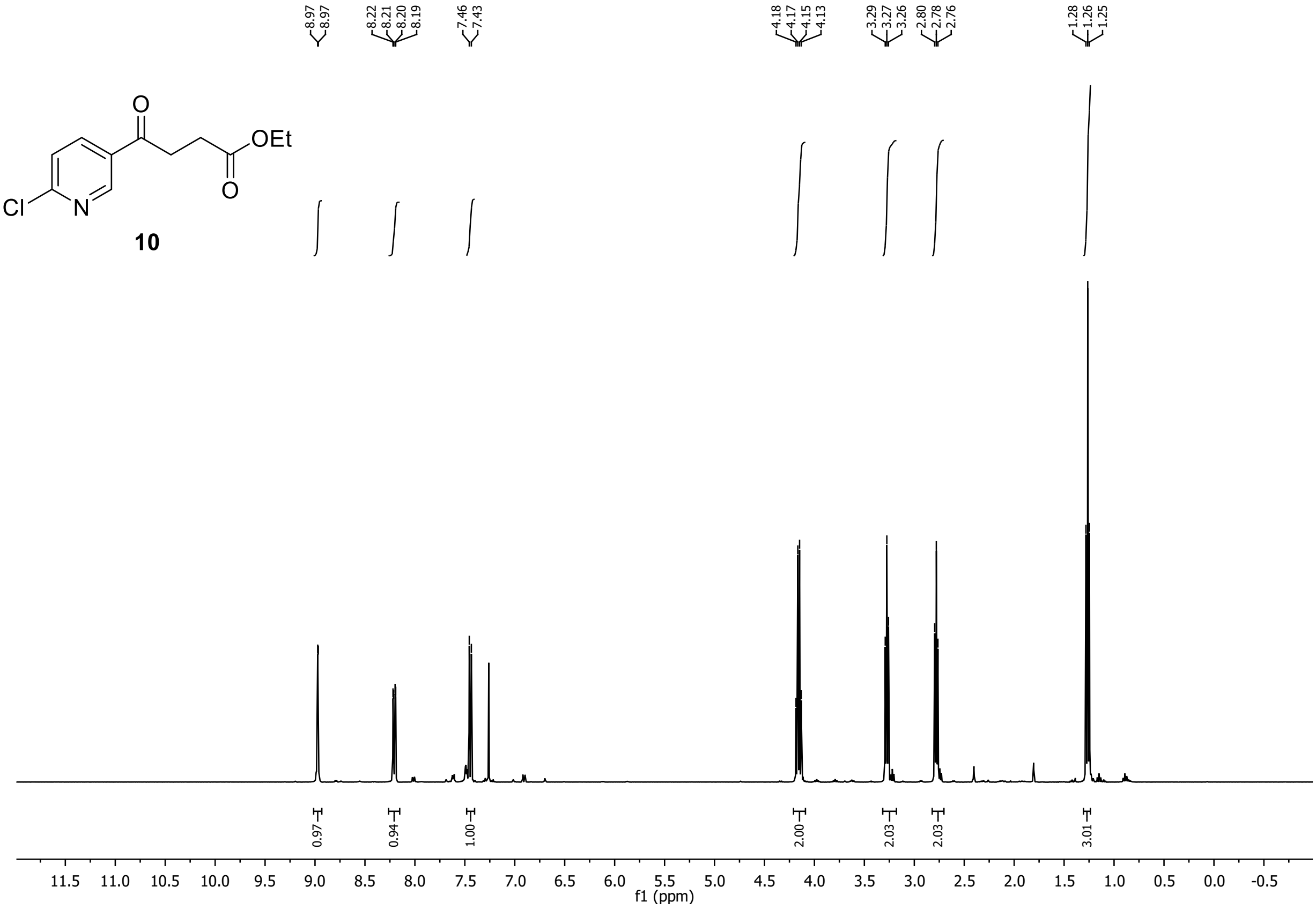

Figure S88. ^{1}H NMR spectrum (400 MHz, $CDCl_3$) of **10**.

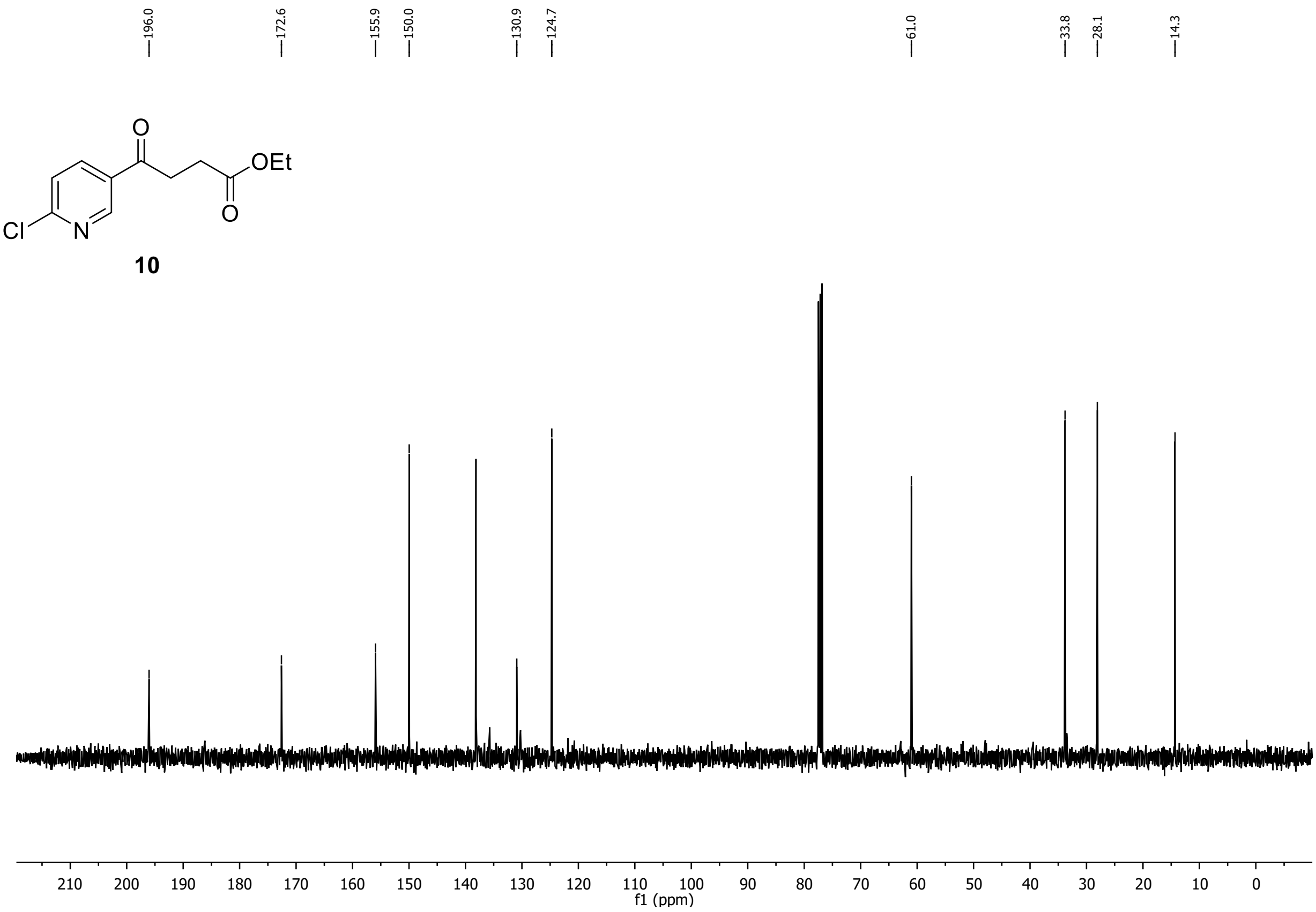

Figure S89. ^{13}C NMR spectrum (101 MHz, $CDCl_3$) of **10**.

Ethyl 4-oxononanoate (11):

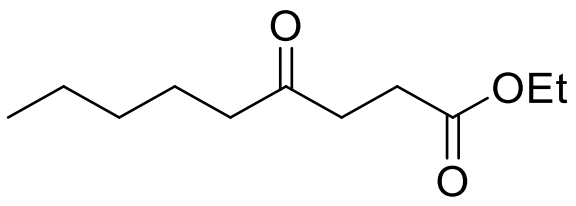

11

$C_{11}H_{20}O_3$ (200.28 g/mol)

Following **GP-B**, **11** was synthesized using **S11** (208 mg, 1.0 mmol, 1.0 equiv.) and ethyl acrylate (200 mg, 2.0 mmol, 2 equiv.). Purification by flash column chromatography (*n*-hexane/EtOAc, gradient 100:0 to 70:30 over 10 CV, detection with $KMnO_4$ stain) afforded **11** (140 mg, 699 µmol, 70%) as colorless oil. Conforms to reported analytical data.[24]

$\mathbf{R_f}$: 0.64 (*n*-hexane/EtOAc 80:20).

^{1}H NMR (400 MHz, $CDCl_3$, δ): 4.11 (q, *J* = 7.2 Hz, 2H), 2.70 (t, *J* = 6.6 Hz, 2H), 2.56 (t, *J* = 6.6 Hz, 2H), 2.43 (t, *J* = 7.5 Hz, 2H), 1.62 – 1.53 (m, 2H), 1.33 – 1.20 (m, 7H), 0.87 (t, *J* = 7.2 Hz, 3H).

^{13}C{^{1}H} NMR (101 MHz, $CDCl_3$, δ): 209.4, 173.0, 60.7, 42.9, 37.2, 31.5, 28.1, 23.6, 22.6, 14.3, 14.0.

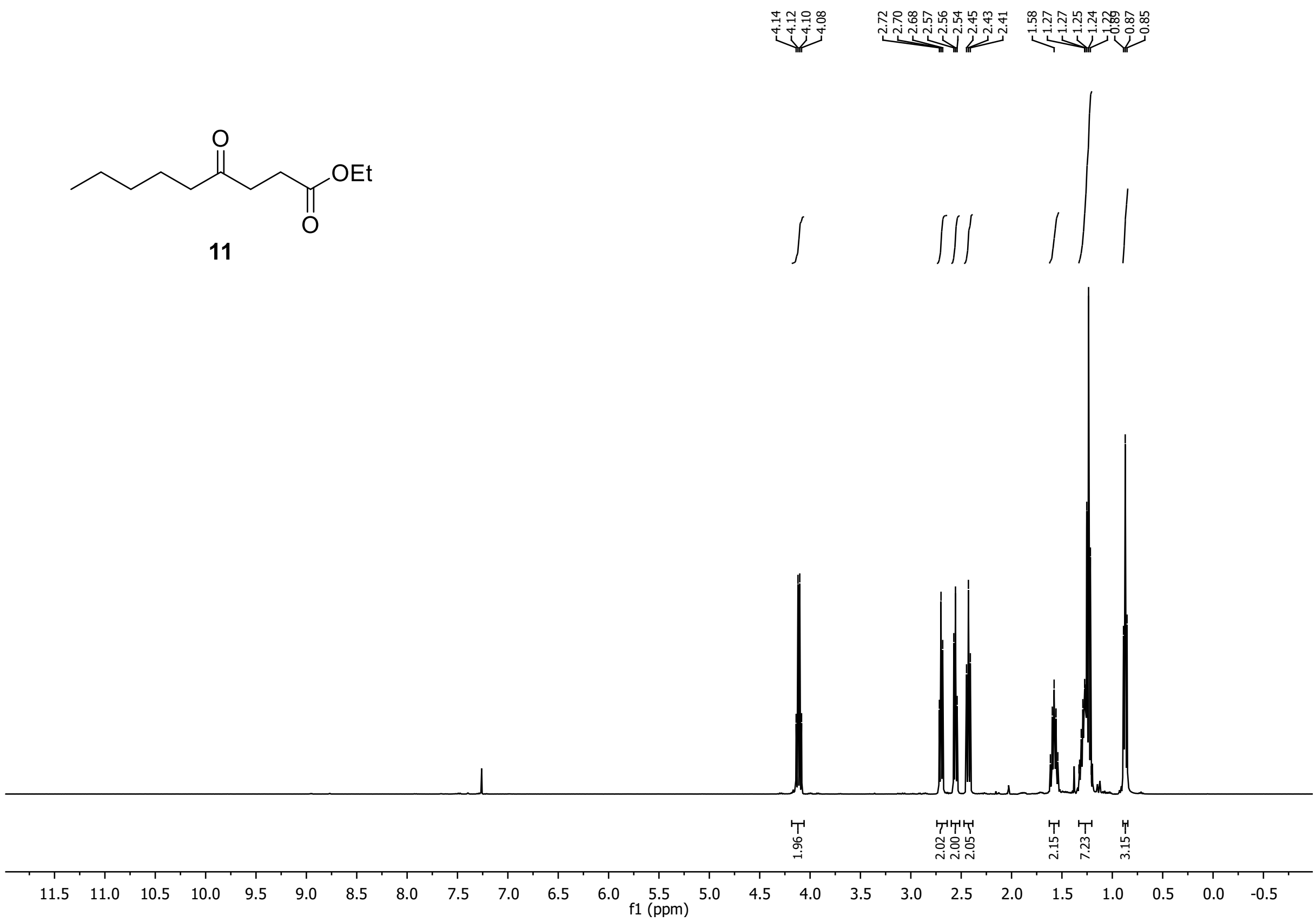

Figure S90. ^{1}H NMR spectrum (400 MHz, $CDCl_3$) of **11**.

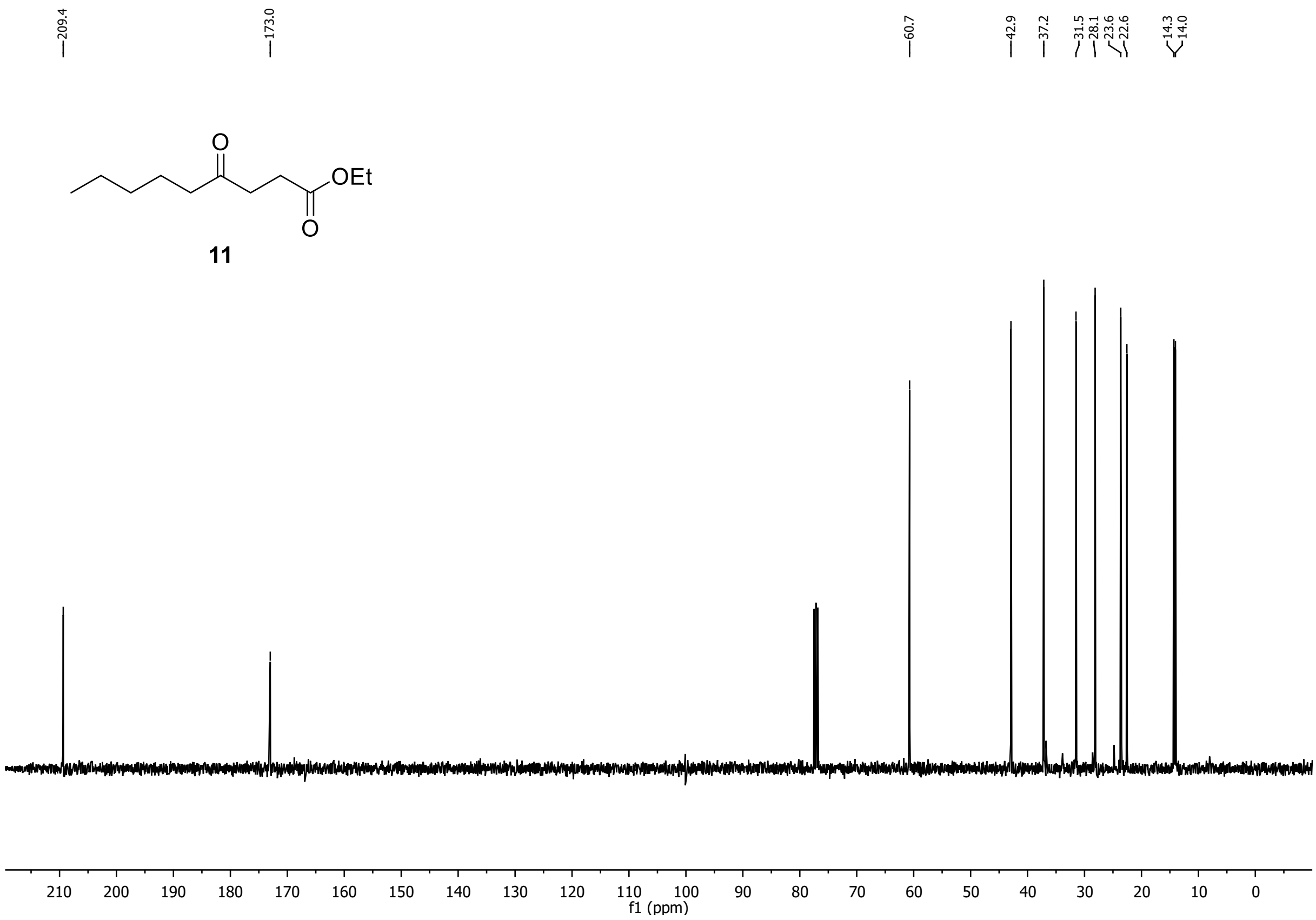

Figure S91. ^{13}C NMR spectrum (101 MHz, $CDCl_3$) of **11**.

Ethyl 4-oxooct-7-enoate (12):

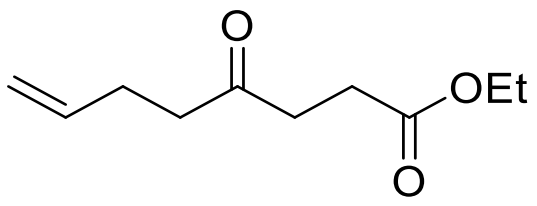

12

$C_{10}H_{16}O_3$ (184.24 g/mol)

Following **GP-B**, **12** was synthesized using **S12** (192 mg, 1.0 mmol, 1.0 equiv.) and ethyl acrylate (200 mg, 2.0 mmol, 2 equiv.). Purification by flash column chromatography (*n*-hexane/EtOAc, gradient 100:0 to 70:30 over 10 CV, detection with $KMnO_4$ stain) afforded **12** (106 mg, 575 µmol, 58%) as colorless oil. Conforms to reported analytical data.[20]

R$_f$: 0.55 (*n*-hexane/EtOAc 80:20).

^{1}H NMR (400 MHz, $CDCl_3$, δ): 5.85 – 5.72 (m, 1H), 5.05 – 4.94 (m, 2H), 4.11 (q, *J* = 7.1 Hz, 2H), 2.71 (t, *J* = 6.5 Hz, 2H), 2.60 – 2.50 (m, 4H), 2.37 – 2.28 (m, 2H), 1.23 (t, *J* = 7.1 Hz, 3H).

^{13}C{^{1}H} NMR (101 MHz, $CDCl_3$, δ): 208.3, 172.9, 137.1, 115.4, 60.7, 41.9, 37.2, 28.1, 27.8, 14.3.

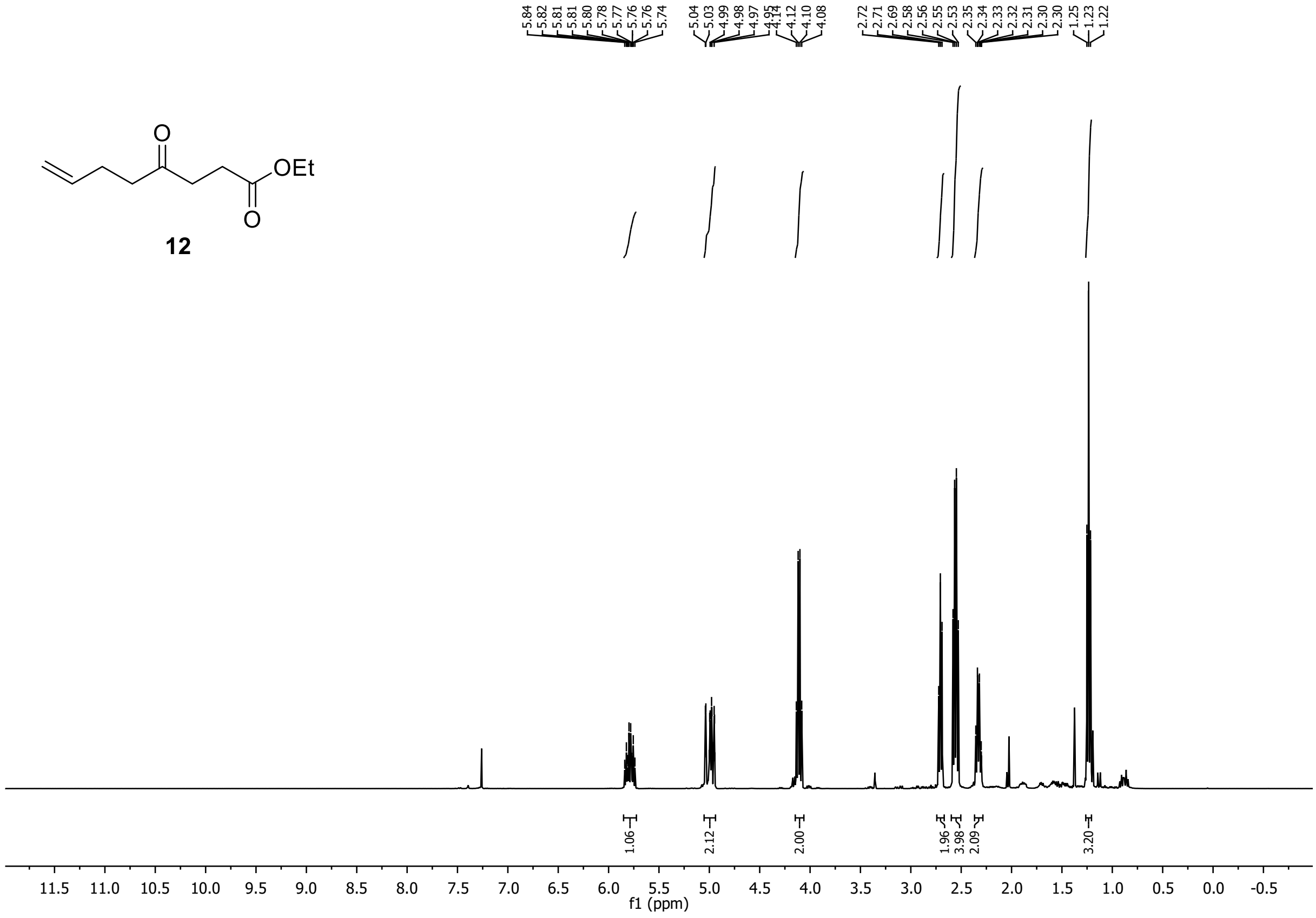

Figure S92. ^{1}H NMR spectrum (400 MHz, $CDCl_3$) of **12**.

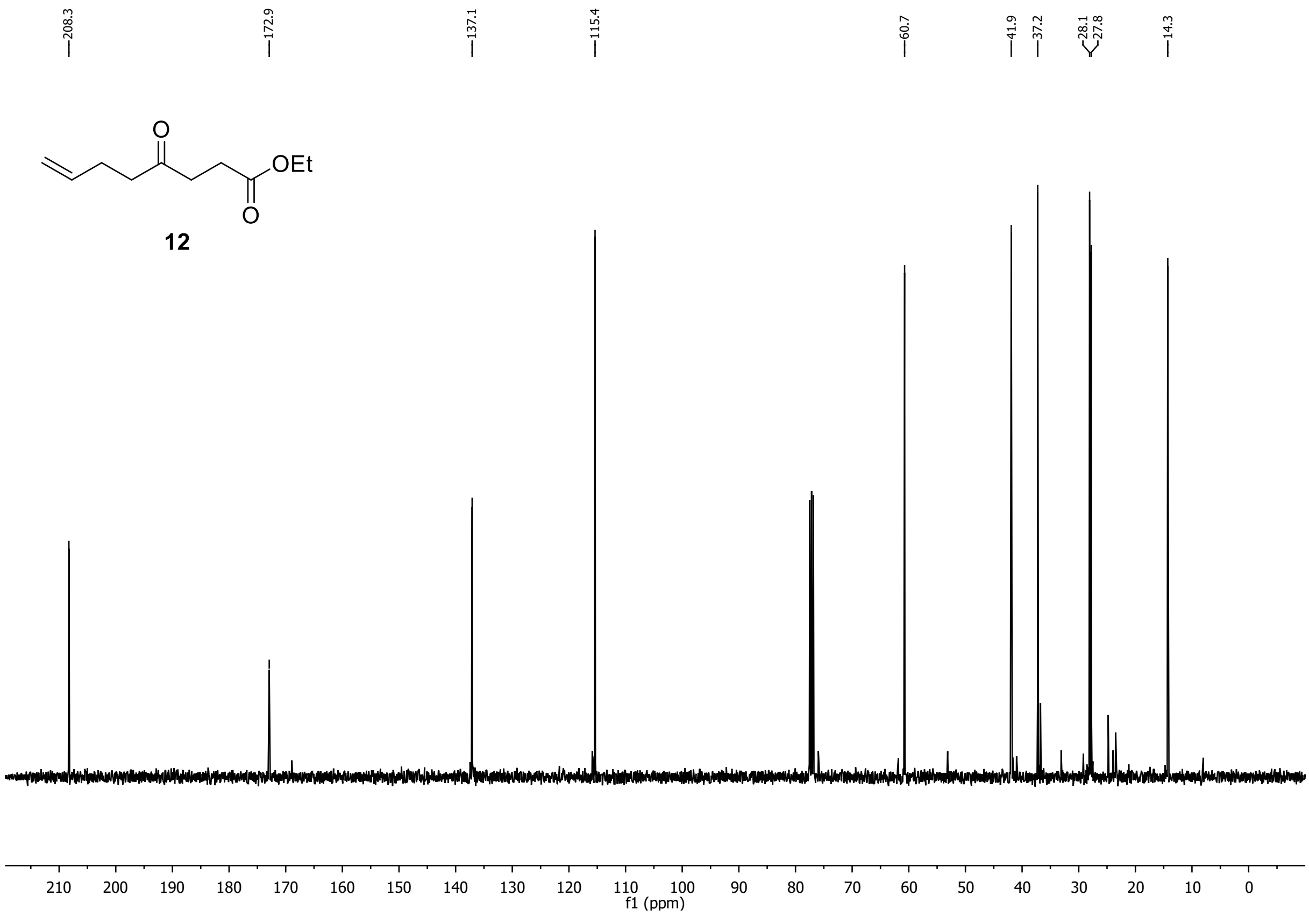

Figure S93. ^{13}C NMR spectrum (101 MHz, $CDCl_3$) of **12**.

Ethyl 4-cyclopropyl-4-oxobutanoate (13):

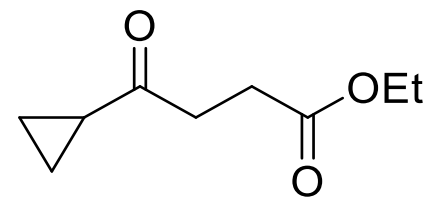

13

$C_9H_{14}O_3$ (170.21 g/mol)

Following **GP-B**, **13** was synthesized using **S13** (178 mg, 1.0 mmol, 1.0 equiv.) and ethyl acrylate (200 mg, 2.0 mmol, 2 equiv.). Purification by flash column chromatography (*n*-hexane/EtOAc, gradient 100:0 to 70:30 over 10 CV, detection with $KMnO_4$ stain) afforded **13** (82 mg, 482 µmol, 48%) as colorless oil.

R_f: 0.52 (*n*-hexane/EtOAc 80:20).

^{1}H NMR (400 MHz, $CDCl_3$, δ): 4.10 (q, *J* = 7.1 Hz, 2H), 2.87 (t, *J* = 6.7 Hz, 2H), 2.55 (t, *J* = 6.7 Hz, 2H), 1.99 – 1.88 (m, 1H), 1.23 (t, *J* = 7.1 Hz, 3H), 1.05 – 0.98 (m, 2H), 0.90 – 0.83 (m, 2H).

^{13}C{^{1}H} NMR (101 MHz, $CDCl_3$, δ): 208.8, 172.9, 60.6, 37.7, 28.2, 20.6, 14.3, 10.8.

HR-MS (ESI): m/z calc for $[M+H]^+$ 171.10157, found 171.10163 (err. 0.34 ppm).

IR (ATR, $\tilde{\nu}$ [cm^{-1}]): 2983 (w), 2935 (w), 2910 (w), 1731 (s), 1698 (s), 1448 (w), 1379 (s), 1299 (w), 1254 (m), 1202 (s), 1165 (s), 1090 (s), 1015 (s), 978 (w), 952 (w), 900 (w), 863 (w), 815 (w), 792 (w), 762 (w).

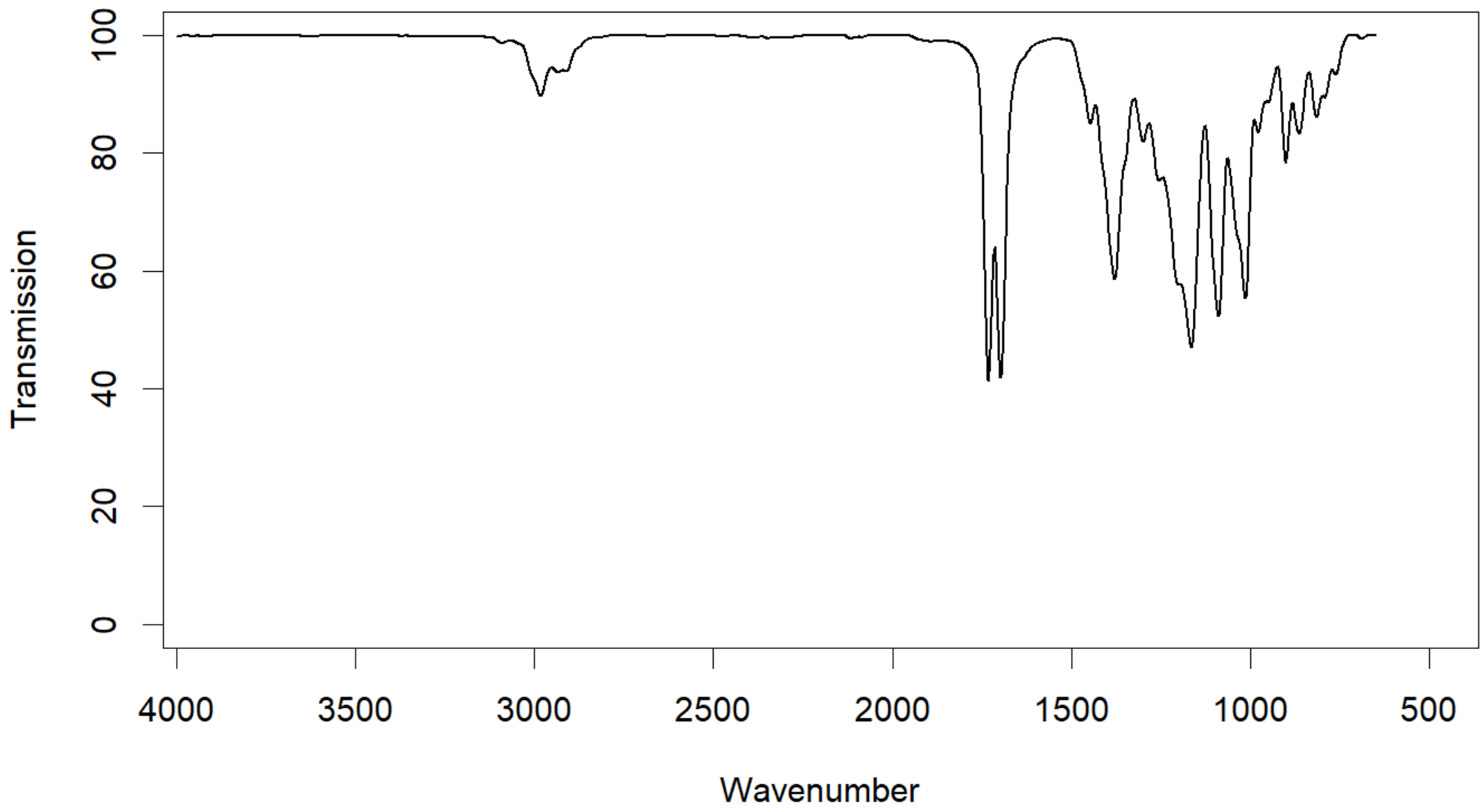

Figure S94. IR spectrum (ATR, neat) of **13**.

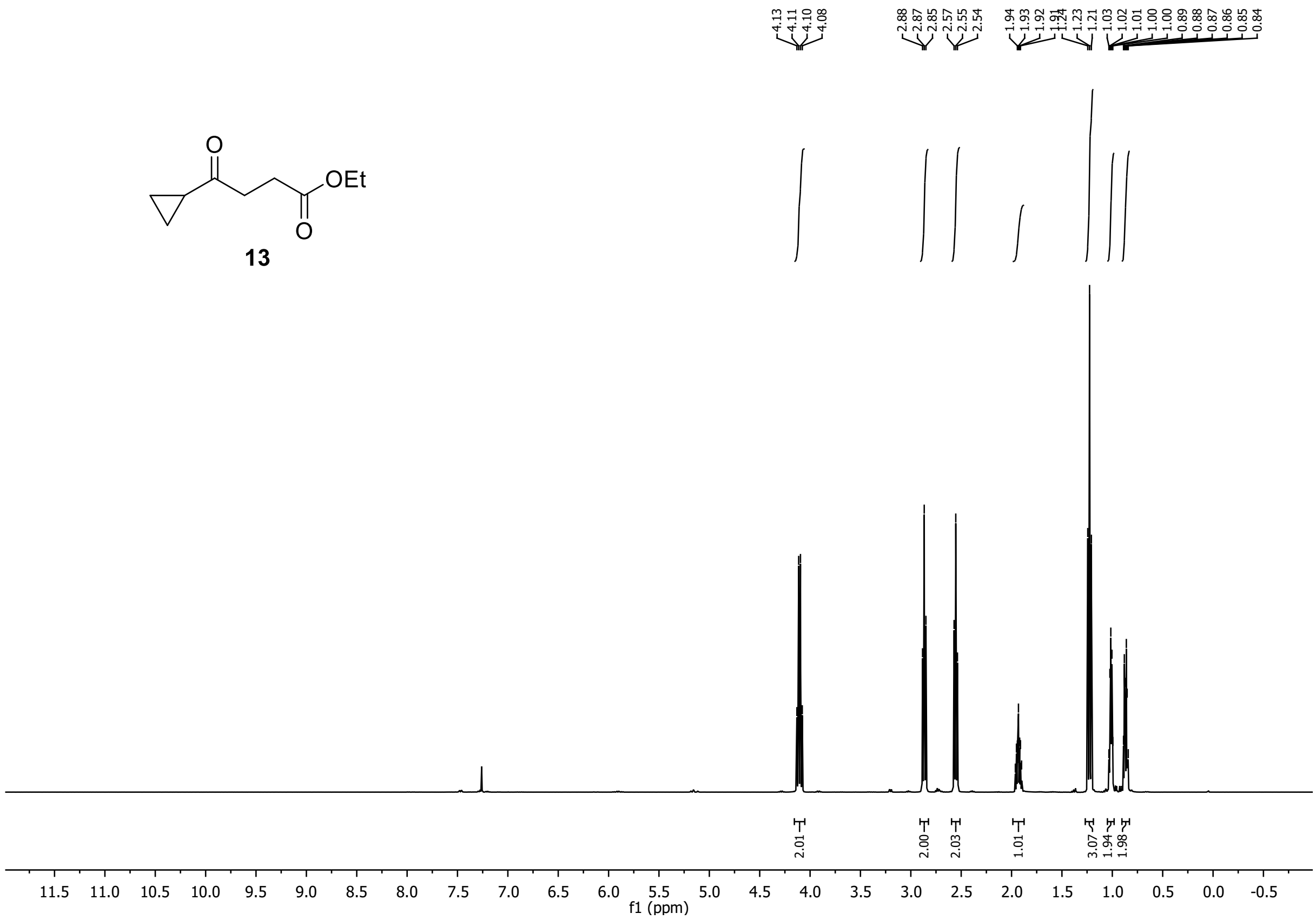

Figure S95. ^{1}H NMR spectrum (400 MHz, $CDCl_3$) of **13**.

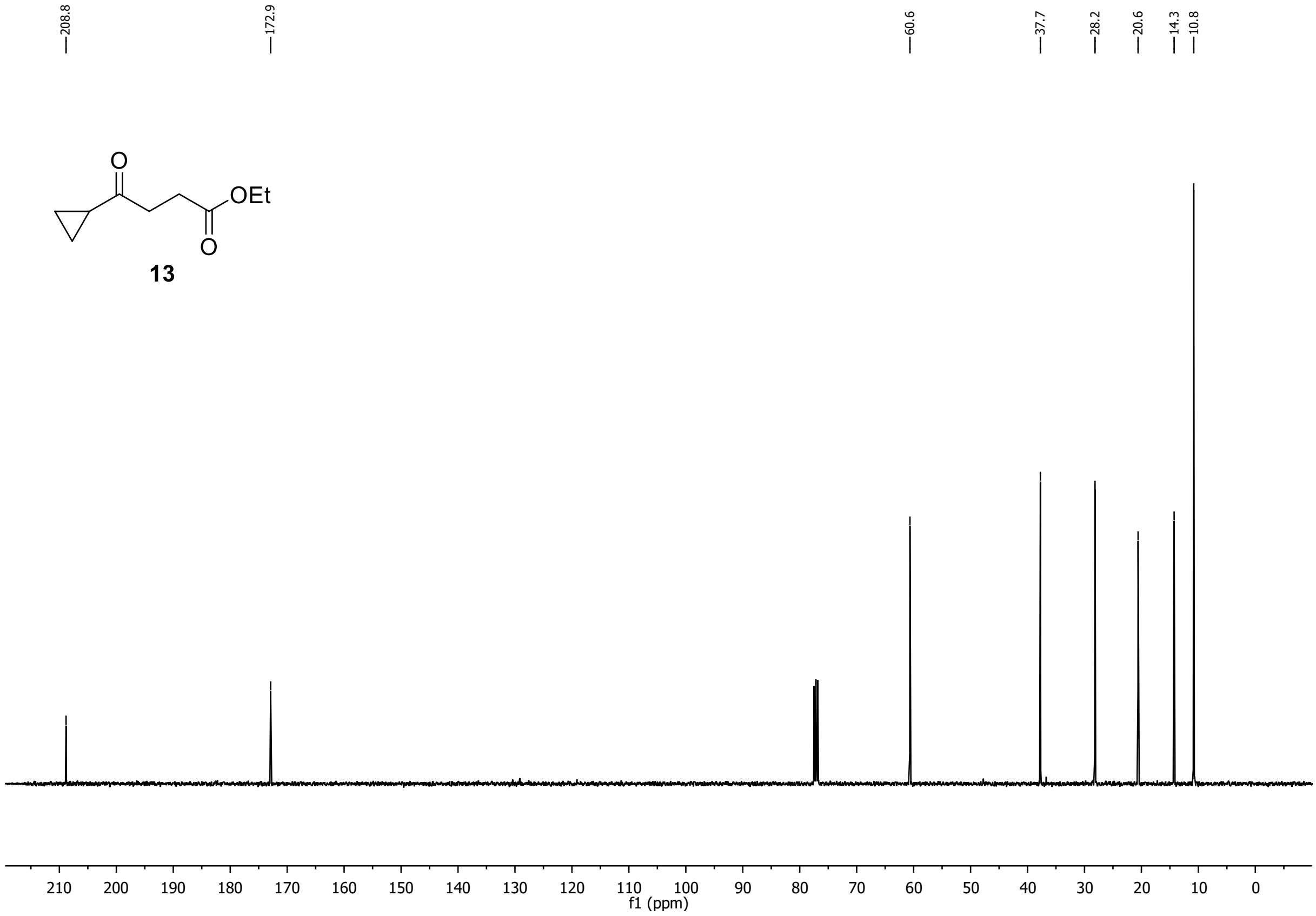

Figure S96. ${}^{13}C$ NMR spectrum (101 MHz, $CDCl_3$) of **13**.

Ethyl 4-cyclohexyl-4-oxobutanoate (14):

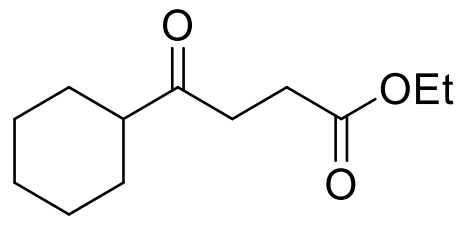

14

$C_{12}H_{20}O_3$ (212.29 g/mol)

Following **GP-B**, **14** was synthesized using **S14** (220 mg, 1.0 mmol, 1.0 equiv.) and ethyl acrylate (200 mg, 2.0 mmol, 2 equiv.). The reaction time was prolonged to 2 h. Purification by flash column chromatography (*n*-hexane/EtOAc, gradient 100:0 to 70:30 over 10 CV, detection with $KMnO_4$ stain) afforded **14** (165 mg, 778 µmol, 78%) as colorless oil. Conforms to reported analytical data.[25]

R$_f$: 0.64 (*n*-hexane/EtOAc 80:20).

^{1}H NMR (400 MHz, $CDCl_3$, δ): 4.11 (q, *J* = 7.2 Hz, 2H), 2.73 (t, *J* = 6.6 Hz, 2H), 2.55 (t, *J* = 6.6 Hz, 2H), 2.43 – 2.30 (m, 1H), 1.90 – 1.73 (m, 4H), 1.69 – 1.62 (m, 1H), 1.40 – 1.15 (m, 8H).

^{13}C{^{1}H} NMR (101 MHz, $CDCl_3$, δ): 212.2, 173.1, 60.7, 50.9, 35.2, 28.6, 28.1, 26.0, 25.8, 14.3.

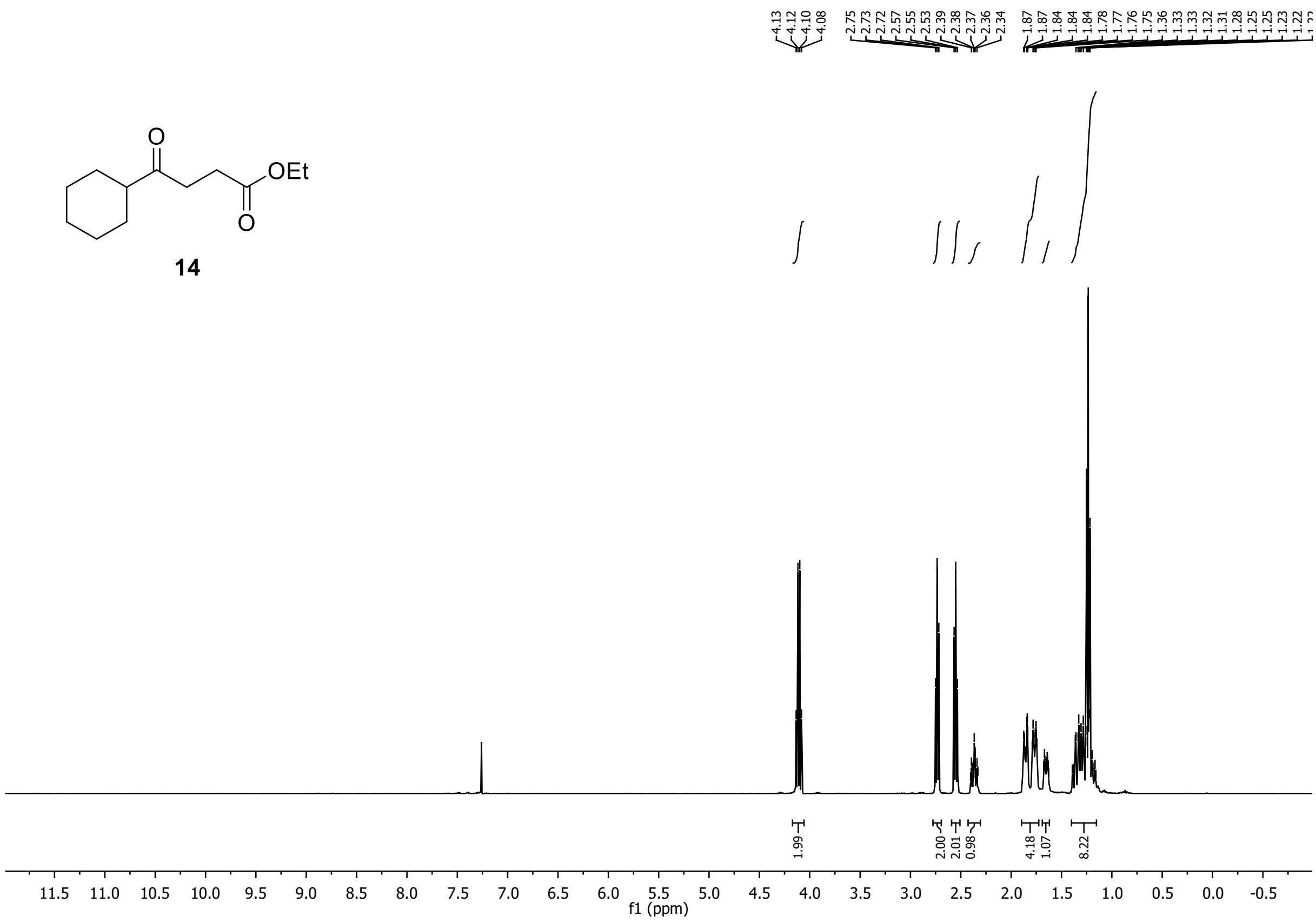

Figure S97. ^{1}H NMR spectrum (400 MHz, $CDCl_3$) of **14**.

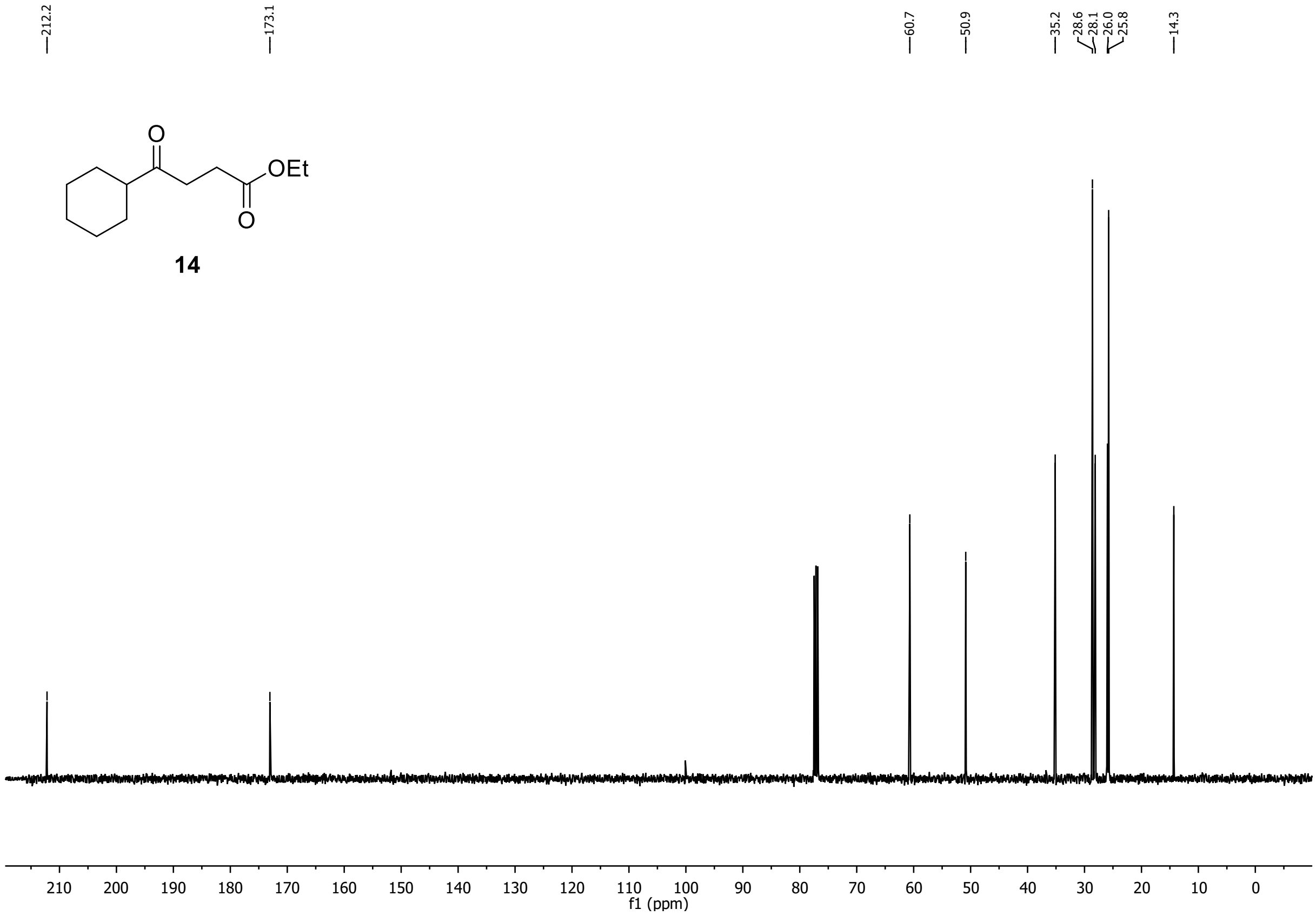

Figure S98. ^{13}C NMR spectrum (101 MHz, $CDCl_3$) of **14**.

Ethyl 4-oxo-4-(tetrahydro-2*H*-pyran-4-yl)butanoate (15):

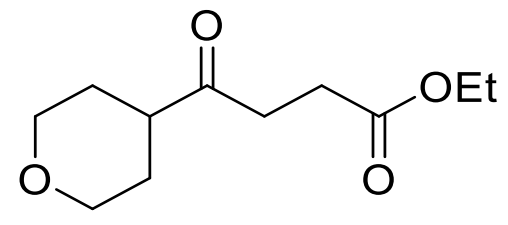

15

$C_{11}H_{18}O_4$ (214.26 g/mol)

Following **GP-B**, **15** was synthesized using **S15** (222 mg, 1.0 mmol, 1.0 equiv.) and ethyl acrylate (200 mg, 2.0 mmol, 2 equiv.). The reaction time was prolonged to 2 h. Purification by flash column chromatography (*n*-hexane/EtOAc, gradient 100:0 to 60:40 over 10 CV, detection with $KMnO_4$ stain) afforded **15** (124 mg, 579 µmol, 58%) as brown oil.

R_f: 0.47 (*n*-hexane/EtOAc 60:40).

^{1}H NMR (400 MHz, $CDCl_3$, δ): 4.11 (q, J = 7.1 Hz, 2H), 4.03 – 3.94 (m, 2H), 3.42 (td, J = 11.4, 2.6 Hz, 2H), 2.74 (t, J = 6.4 Hz, 2H), 2.64 – 2.53 (m, 3H), 1.82 – 1.67 (m, 4H), 1.23 (t, J = 7.1 Hz, 3H).

^{13}C{^{1}H} NMR (101 MHz, $CDCl_3$, δ): 210.1, 172.9, 67.3, 60.8, 47.6, 34.8, 28.2, 28.0, 14.3.

HR-MS (ESI): m/z calc for $[M+Na]^+$ 237.10973, found 237.10984 (err. 0.48 ppm).

IR (ATR, $\tilde{\nu}$ [cm^{-1}]): 2950 (w), 2842 (w), 1728 (s), 1709 (s), 1444 (w), 1374 (m), 1344 (w), 1299 (w), 1262 (m), 1232 (m), 1202 (s), 1169 (s), 1112 (s), 1090 (s), 1016 (s), 945 (w), 889 (w), 852 (w).

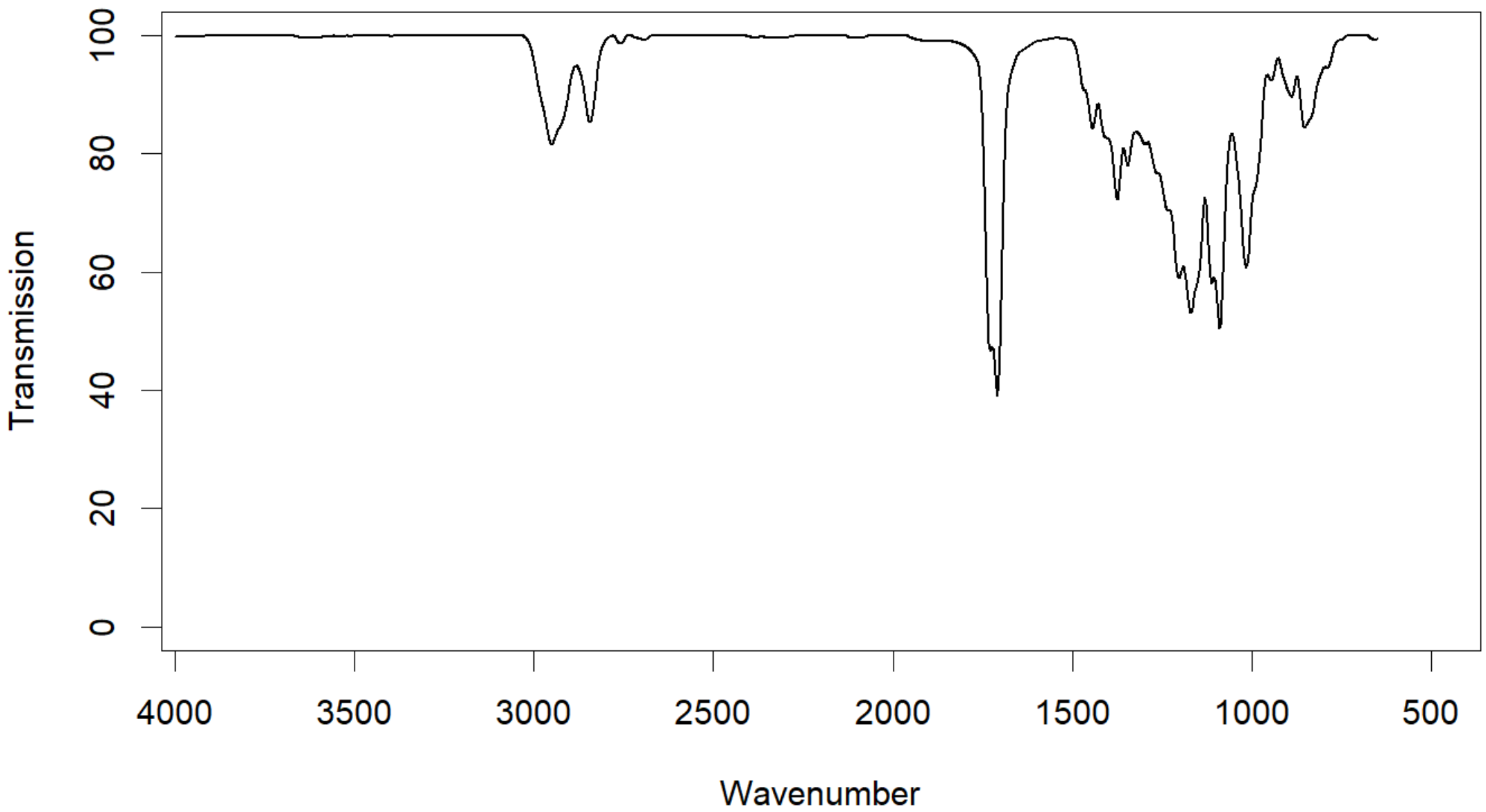

Figure S99. IR spectrum (ATR, neat) of **15**.

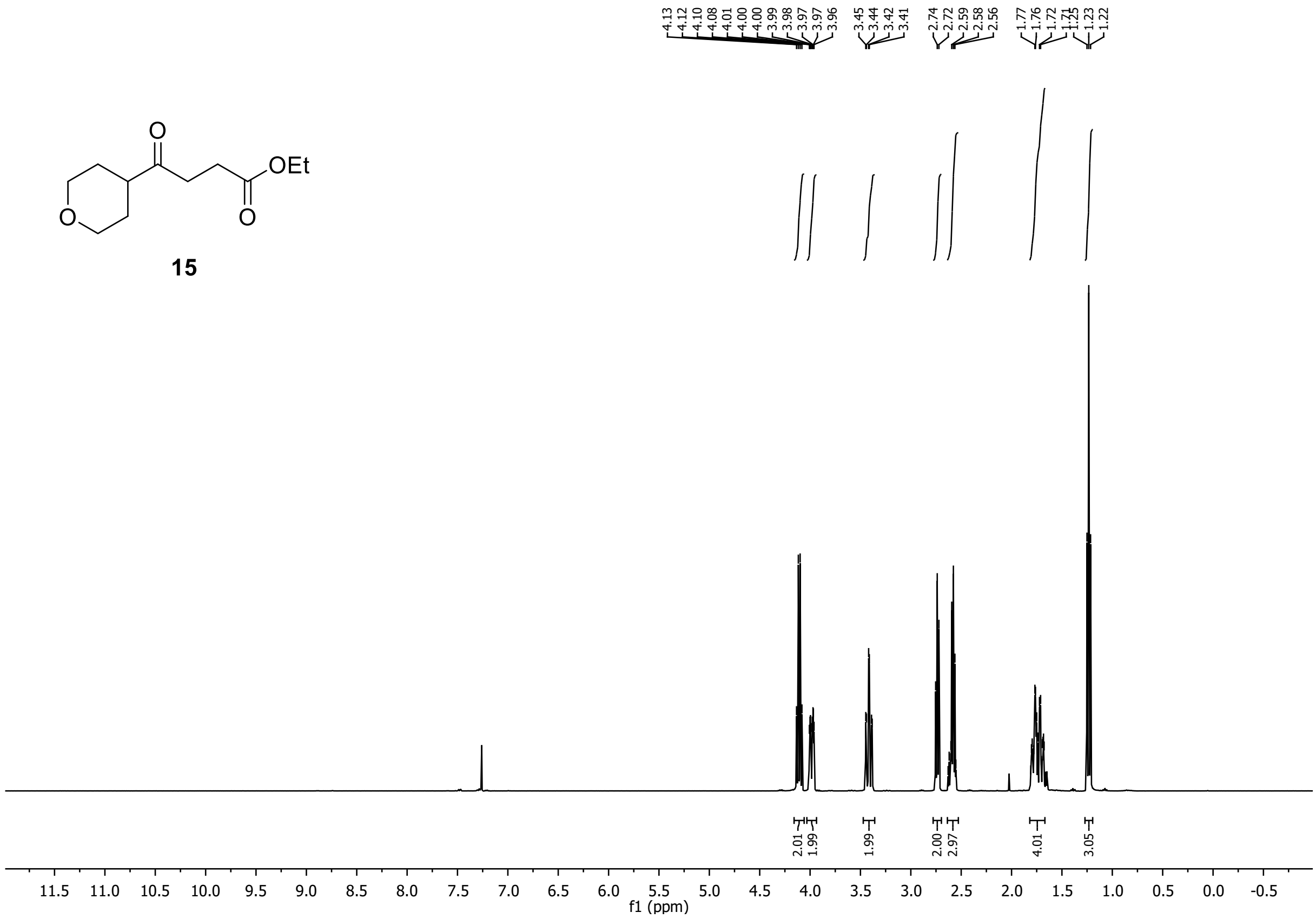

Figure S100. ^{1}H NMR spectrum (400 MHz, $CDCl_3$) of **15**.

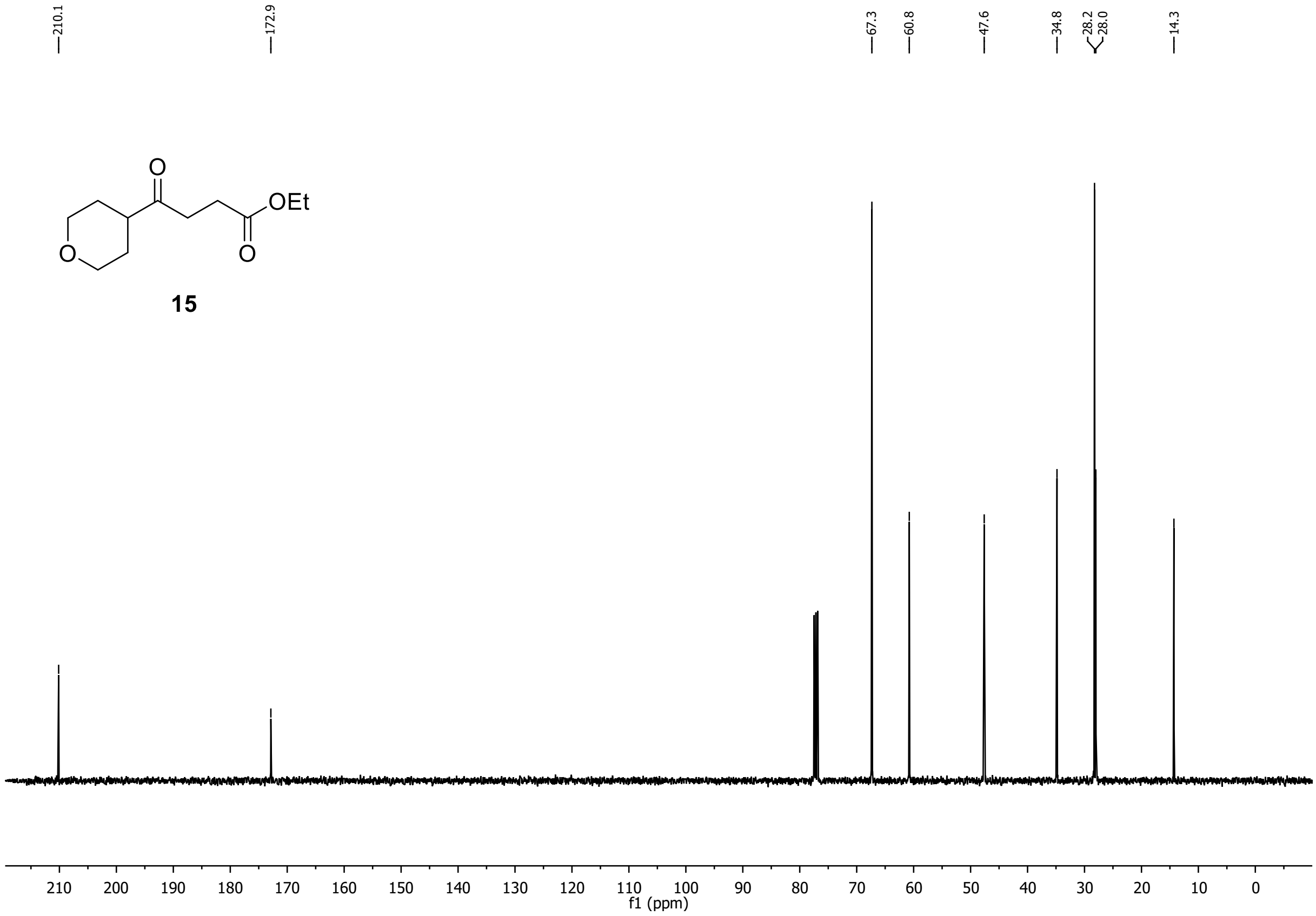

Figure S101. ^{13}C NMR spectrum (101 MHz, $CDCl_3$) of **15**.

Ethyl 4-(1-acetylpiperidin-4-yl)-4-oxobutanoate (16):

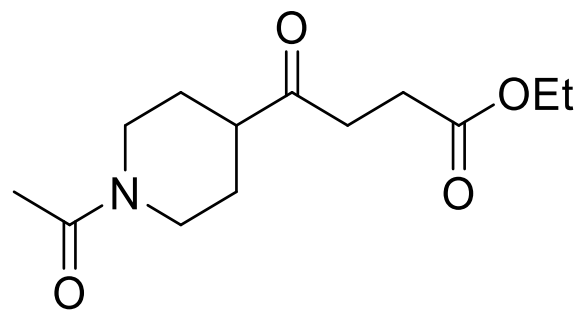

16

$C_{13}H_{21}NO_4$ (255.31 g/mol)

Following **GP-B**, **16** was synthesized using **S16** (222 mg, 1.0 mmol, 1.0 equiv.) and ethyl acrylate (200 mg, 2.0 mmol, 2 equiv.). The reaction time was prolonged to 2 h. Purification by flash column chromatography (EtOAc/MeOH, gradient 100:0 to 0:100 over 10 CV, detection with $KMnO_4$ stain) afforded **16** (155 mg, 607 µmol, 61%) as brown oil.

R$_f$: 0.06 (*n*-hexane/EtOAc 40:60).

^{1}H NMR (400 MHz, $CDCl_3$, δ): 4.56 – 4.46 (m, 1H), 4.11 (q, *J* = 7.1 Hz, 2H), 3.89 – 3.77 (m, 1H), 3.10 (m, 1H), 2.79 – 2.53 (m, 6H), 2.08 (s, 3H), 1.94 – 1.86 (m, 2H), 1.68 – 1.47 (m, 2H), 1.24 (t, *J* = 7.1 Hz, 3H).

^{13}C{^{1}H} NMR (101 MHz, $CDCl_3$, δ): 210.0, 172.8, 169.1, 60.8, 48.3, 45.9, 41.1, 35.2, 28.0, 27.9, 27.5, 21.6, 14.3.

HR-MS (ESI): m/z calc for $[M+H]^+$ 256.15433, found 256.15467 (err. 1.31 ppm).

IR (ATR, $\tilde{\nu}$ [cm^{-1}]): 2977 (w), 2931 (w), 2860 (w), 1709 (s), 1632 (s), 1432 (s), 1370 (m), 1310 (w), 1269 (m), 1229 (m), 1195 (s), 1165 (s), 1127 (m), 1094 (m), 1031 (m), 978 (s), 855 (w), 748 (w).

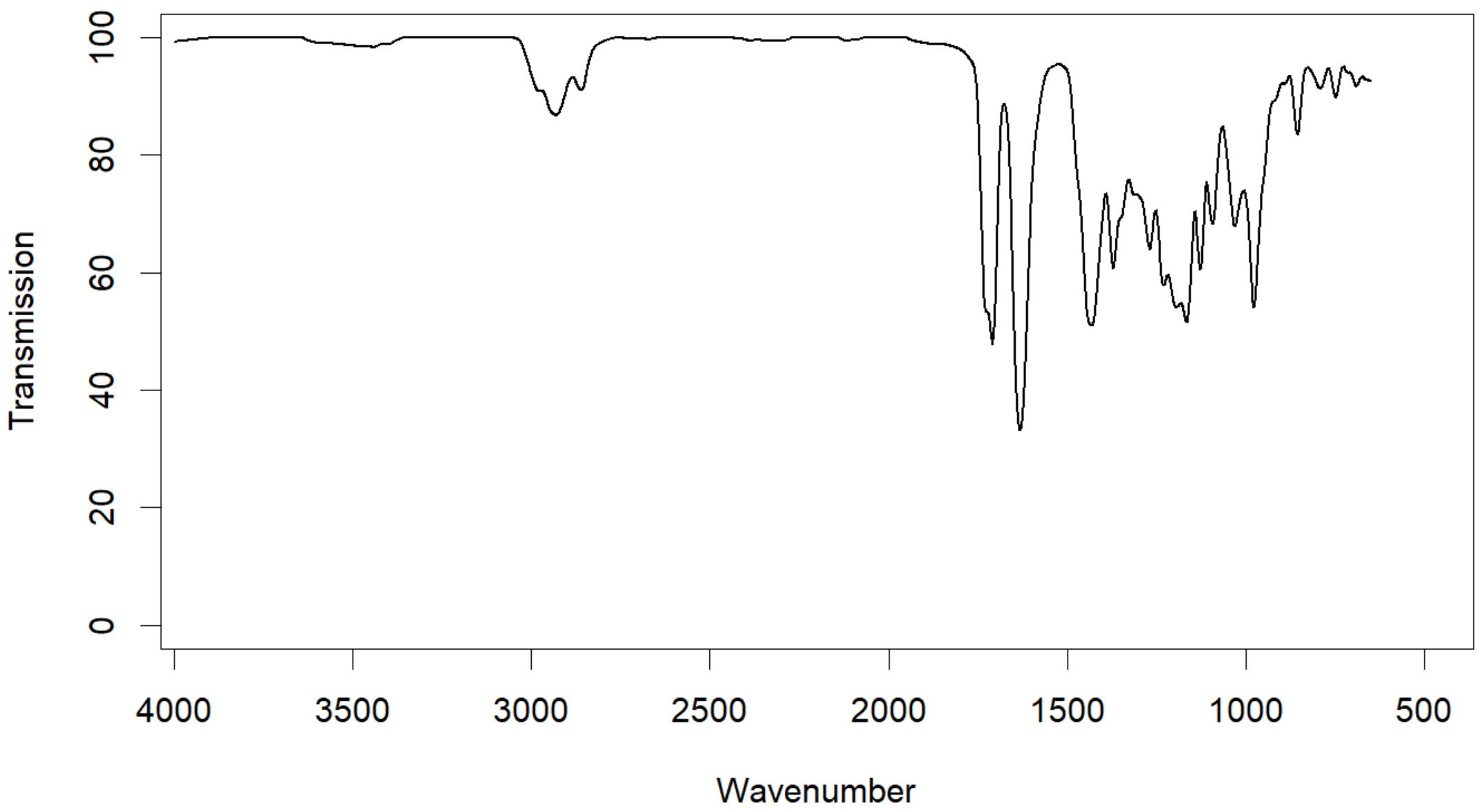

Figure S102. IR spectrum (ATR, neat) of **16**.

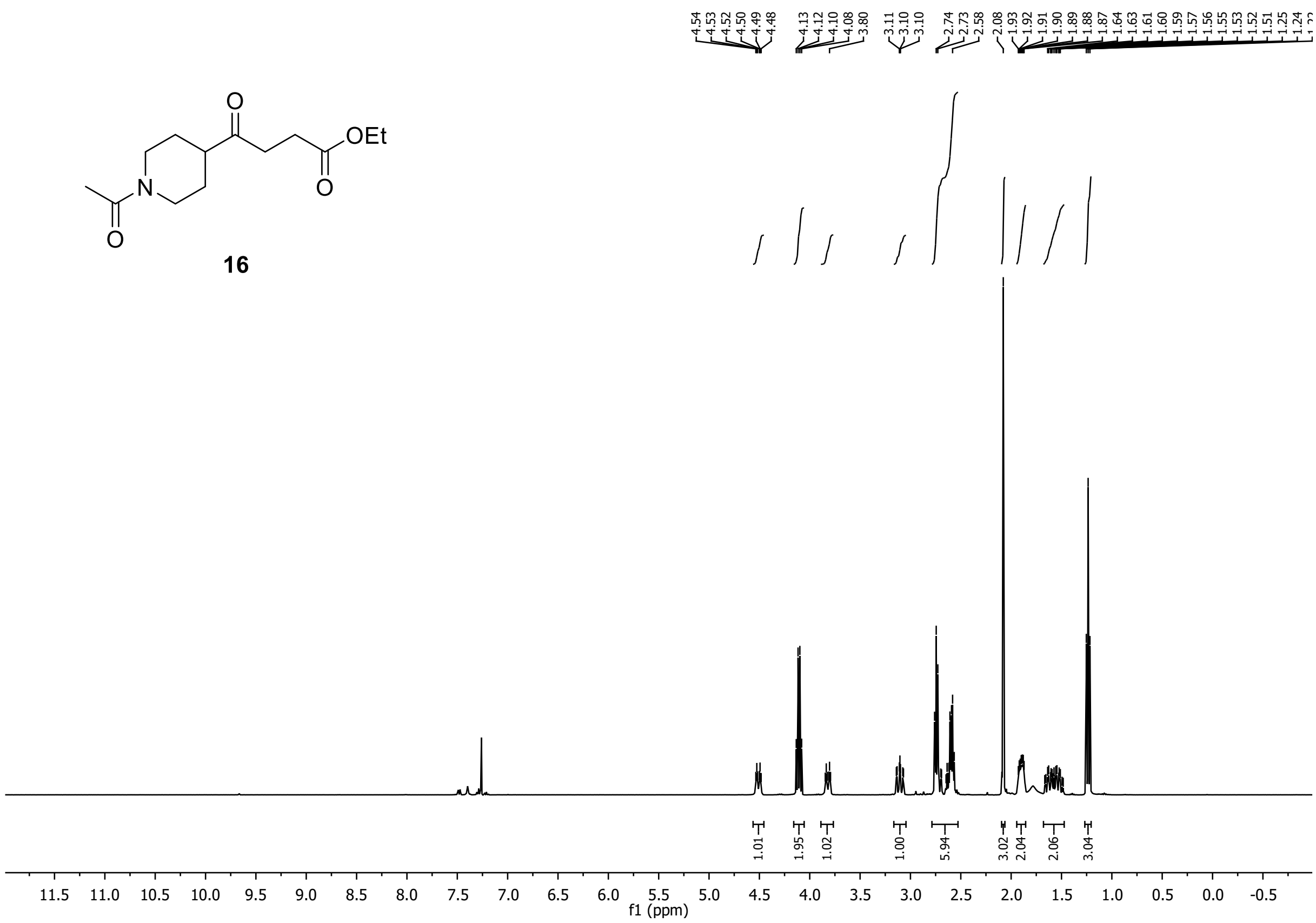

Figure S103. ^{1}H NMR spectrum (400 MHz, $CDCl_3$) of **16**.

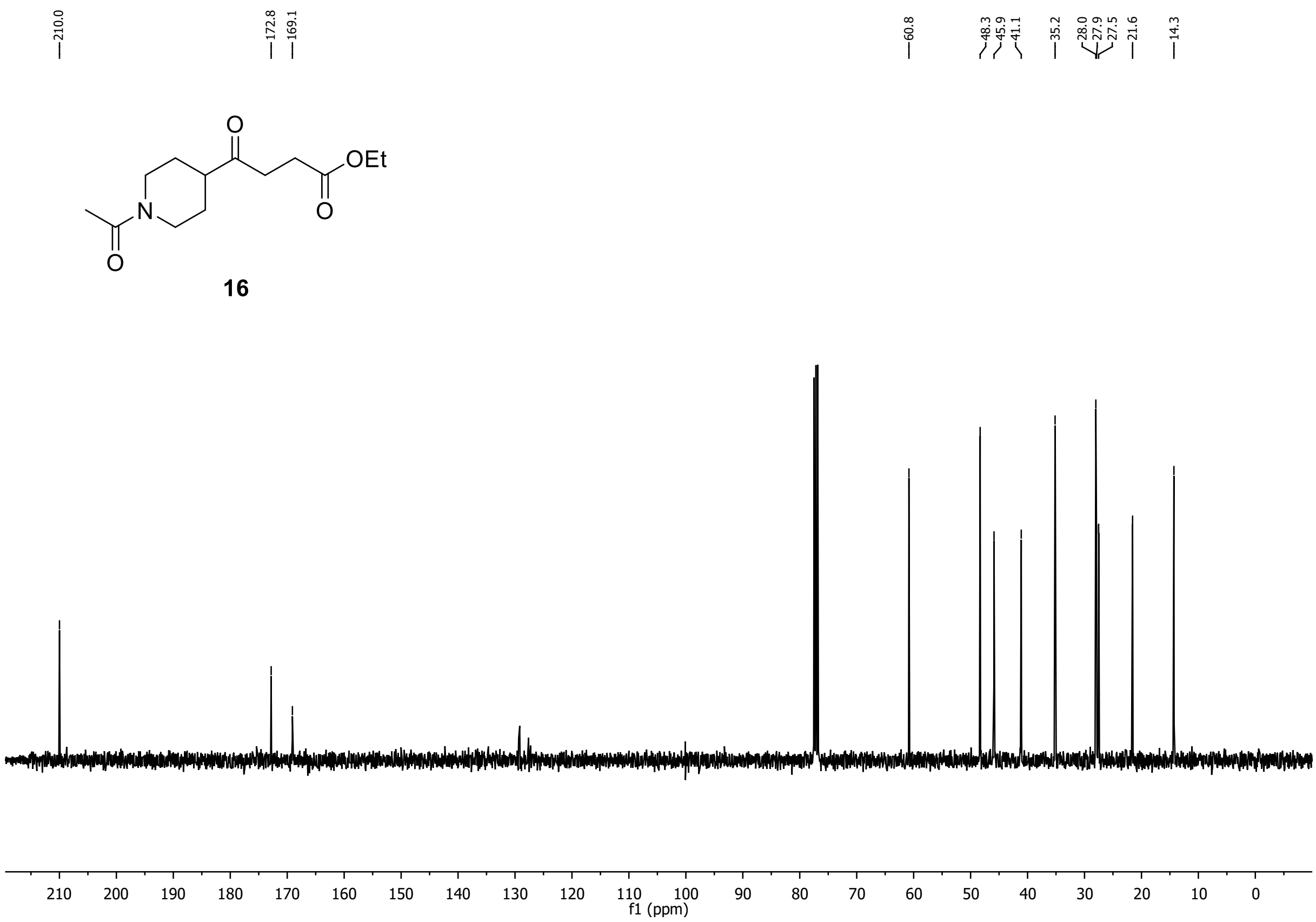

Figure S104. ^{13}C NMR spectrum (101 MHz, $CDCl_3$) of **16**.

Ethyl 4-adamantan-1-yl-4-oxobutanoate (17):

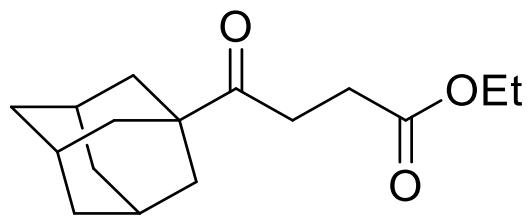

17

$C_{16}H_{24}O_3$ (264.37 g/mol)

Following **GP-B**, **17** was synthesized using **S17** (270 mg, 1.0 mmol, 1.0 equiv.) and ethyl acrylate (200 mg, 2.0 mmol, 2 equiv.). The reaction time was prolonged to 2 h. Purification by flash column chromatography (*n*-hexane/EtOAc, gradient 100:0 to 70:30 over 10 CV, detection with $KMnO_4$ stain) afforded **17** (53 mg, 200 µmol, 20%) as colorless oil. Conforms to reported analytical data.[26]

R_f: 0.69 (*n*-hexane/EtOAc 80:20).

^{1}H NMR (400 MHz, $CDCl_3$, δ): 4.11 (q, J = 7.1 Hz, 2H), 2.76 (t, J = 6.6 Hz, 2H), 2.54 (t, J = 6.6 Hz, 2H), 2.08 – 1.99 (m, 3H), 1.88 – 1.80 (m, 6H), 1.77 – 1.65 (m, 6H), 1.24 (t, J = 7.1 Hz, 3H).

^{13}C{^{1}H} NMR (101 MHz, $CDCl_3$, δ): 213.9, 173.2, 60.6, 46.3, 38.4, 36.7, 31.2, 28.1, 28.1, 14.3.

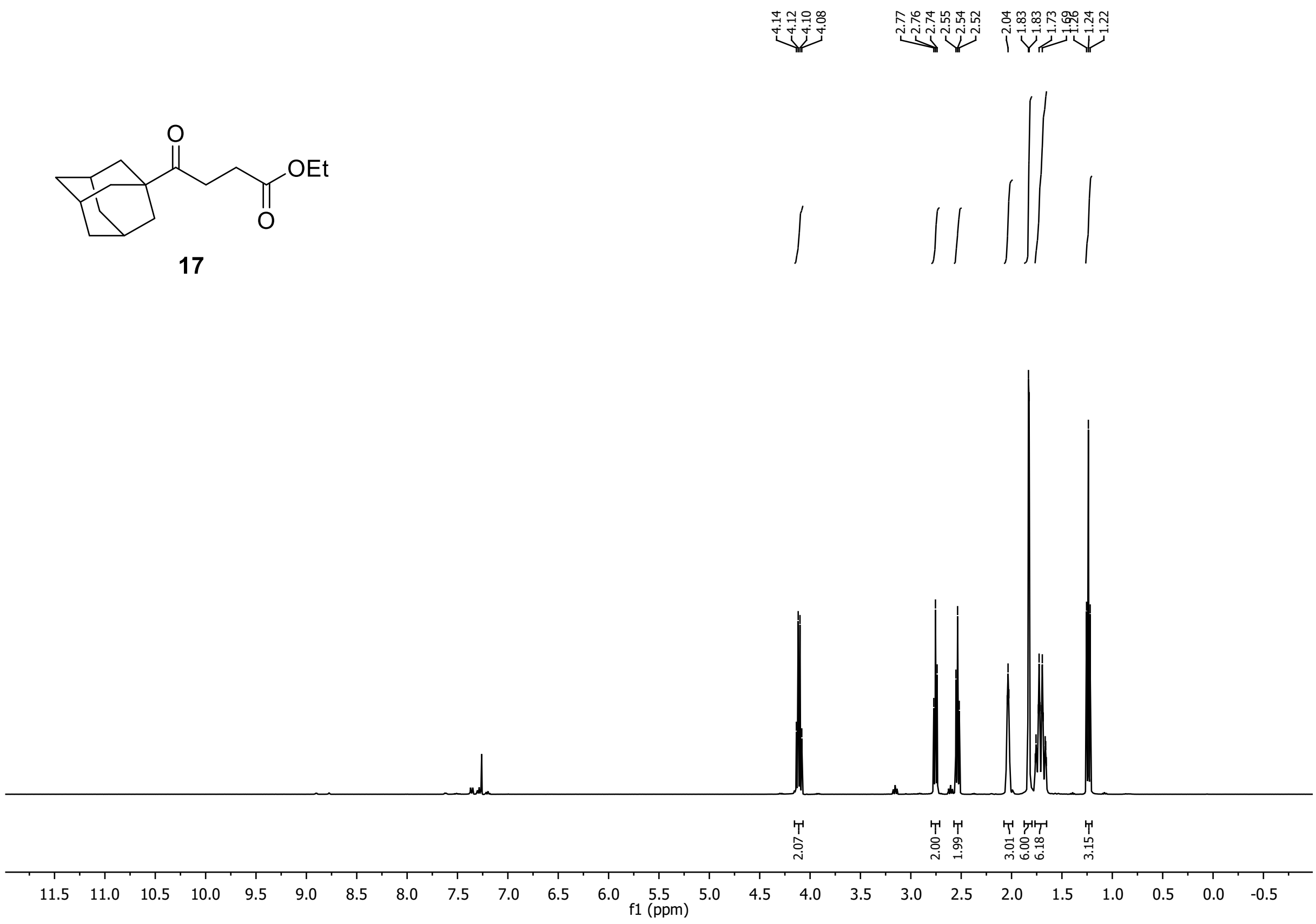

Figure S105. ^{1}H NMR spectrum (400 MHz, $CDCl_3$) of **17**.

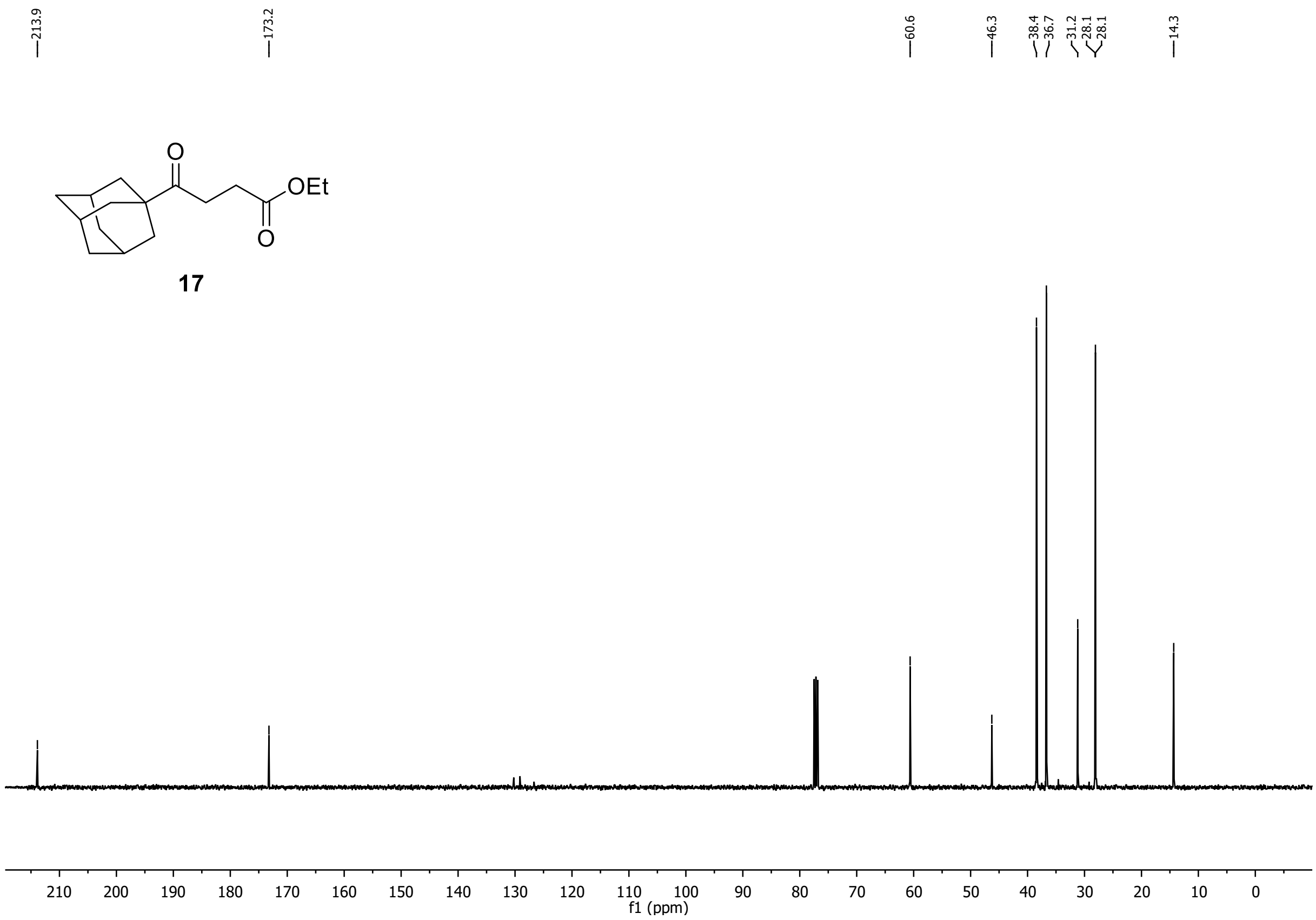

Figure S106. ^{13}C NMR spectrum (101 MHz, $CDCl_3$) of **17**.

Ethyl 4-(3-(cyclopropylmethoxy)-4-(difluoromethoxy)phenyl)-4-oxobutanoate (18):

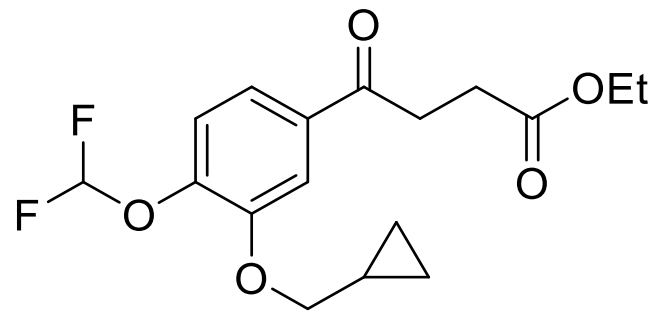

18

$C_{17}H_{20}F_2O_4$ (342.34 g/mol)

Following **GP-B**, **18** was synthesized using **S18** (350 mg, 1.0 mmol, 1.0 equiv.) and ethyl acrylate (200 mg, 2.0 mmol, 2 equiv.). Purification by flash column chromatography (*n*-hexane/EtOAc, gradient 100:0 to 45:55 over 10 CV) afforded **18** (246 mg, 719 µmol, 72%) as colorless oil.

R_f: 0.83 (*n*-hexane/EtOAc 60:40).

^{1}H NMR (400 MHz, $CDCl_3$, δ): 7.60 – 7.53 (m, 2H), 7.22 (d, *J* = 8.7 Hz, 1H), 6.72 (t, *J* = 75.0 Hz, 1H), 4.16 (q, *J* = 7.1 Hz, 2H), 3.92 (d, *J* = 6.9 Hz, 2H), 3.27 (t, *J* = 6.6 Hz, 2H), 2.74 (t, *J* = 6.6 Hz, 2H), 1.36 – 1.23 (m, 4H), 0.70 – 0.59 (m, 2H), 0.43 – 0.29 (m, 2H).

^{13}C{^{1}H} NMR (101 MHz, $CDCl_3$, δ): 197.0, 172.9, 150.6, 144.4, 134.9, 121.9, 121.8, 115.9 (t, *J* = 260.7 Hz), 113.4, 74.2, 60.9, 33.4, 28.4, 14.3, 10.2, 3.4.

^{19}F NMR (376 MHz, $CDCl_3$, δ): -82.0 (d, *J* = 75.0 Hz).

HR-MS (ESI): m/z calc for $[M+K]^+$ 381.09104, found 381.09128 (err. 0.64 ppm).

IR (ATR, $\tilde{\nu}$ [cm^{-1}]): 2984 (w), 2926 (w), 2880 (w), 1728 (s), 1683 (m), 1594 (w), 1507 (w), 1467 (w), 1403 (m), 1374 (w), 1351 (w), 1314 (w), 1266 (s), 1206 (m), 1106 (vs), 1049 (s), 1023 (s), 997 (s), 919 (w), 878 (w), 822 (m), 796 (m), 733 (w).

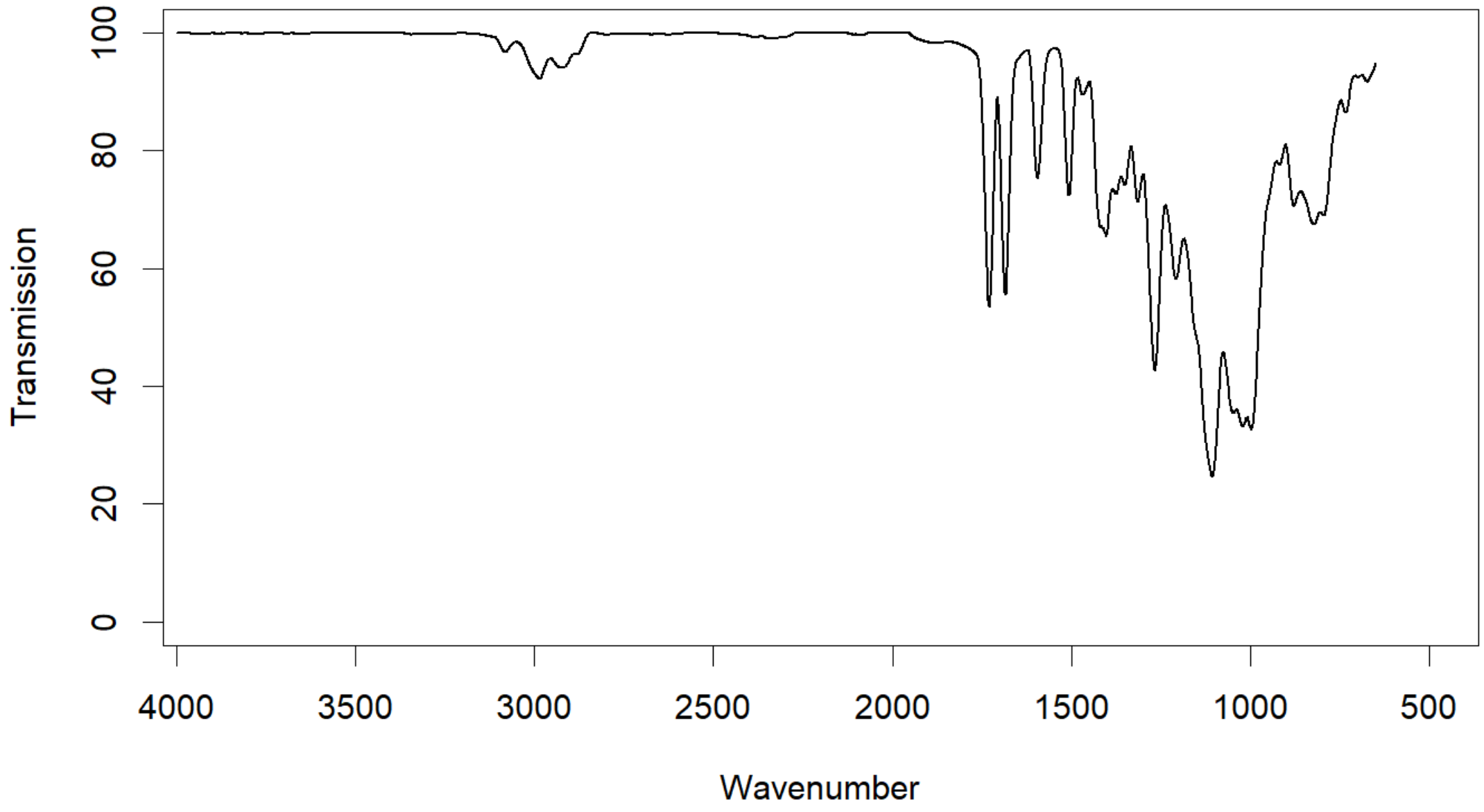

Figure S107. IR spectrum (ATR, neat) of **18**.

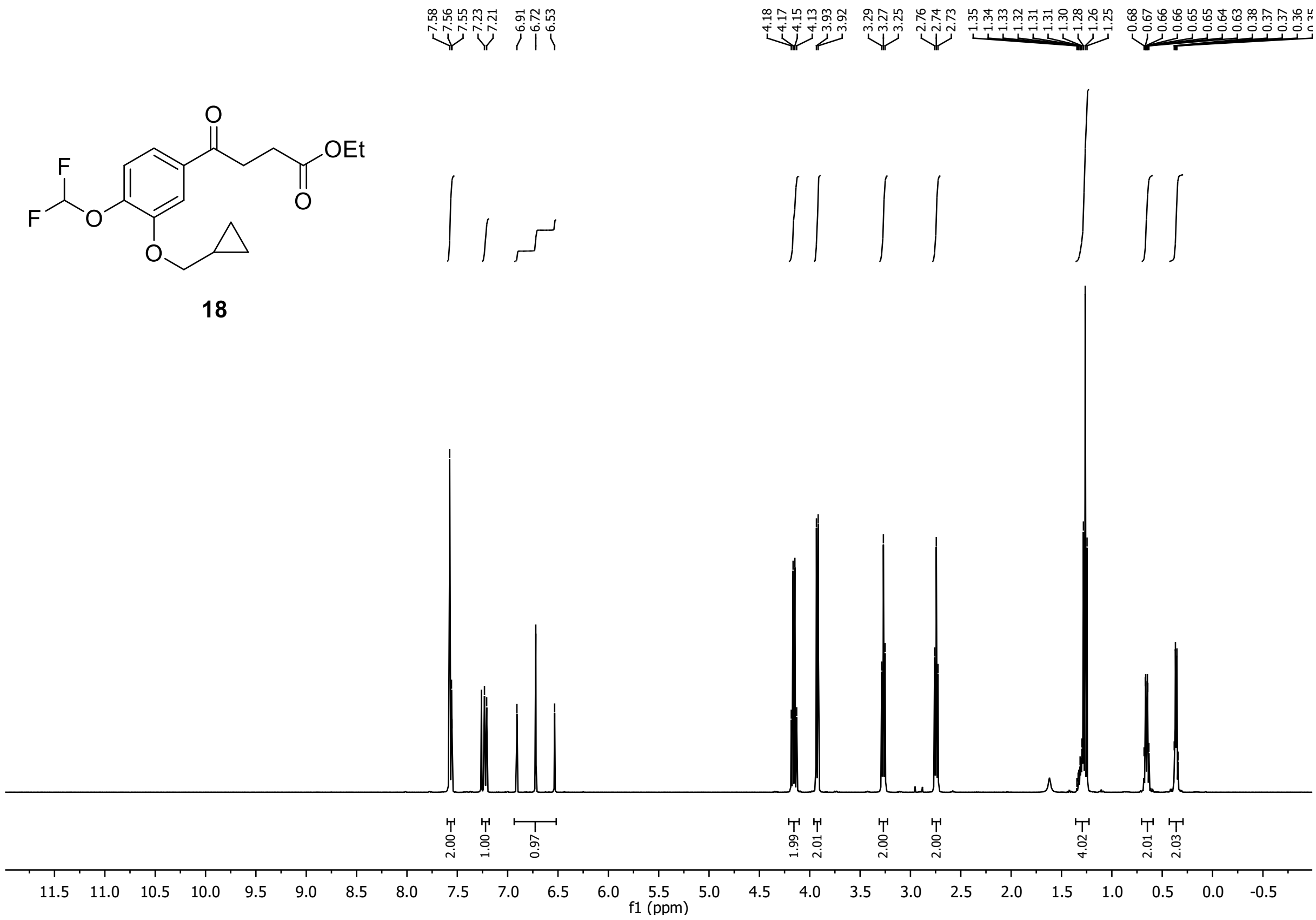

Figure S108. ^{1}H NMR spectrum (400 MHz, $CDCl_3$) of **18**.

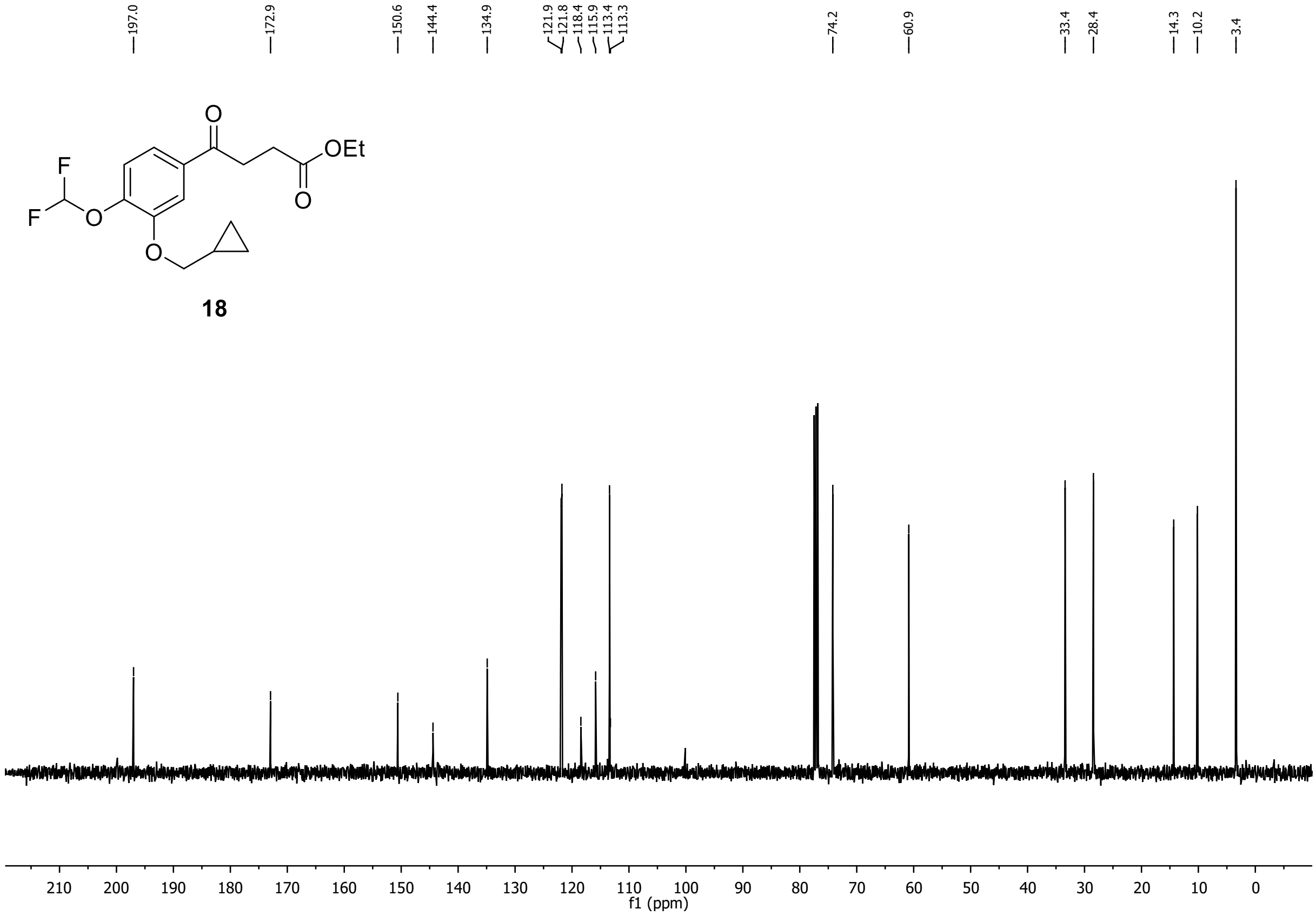

Figure S109. ^{13}C NMR spectrum (101 MHz, $CDCl_3$) of **18**.

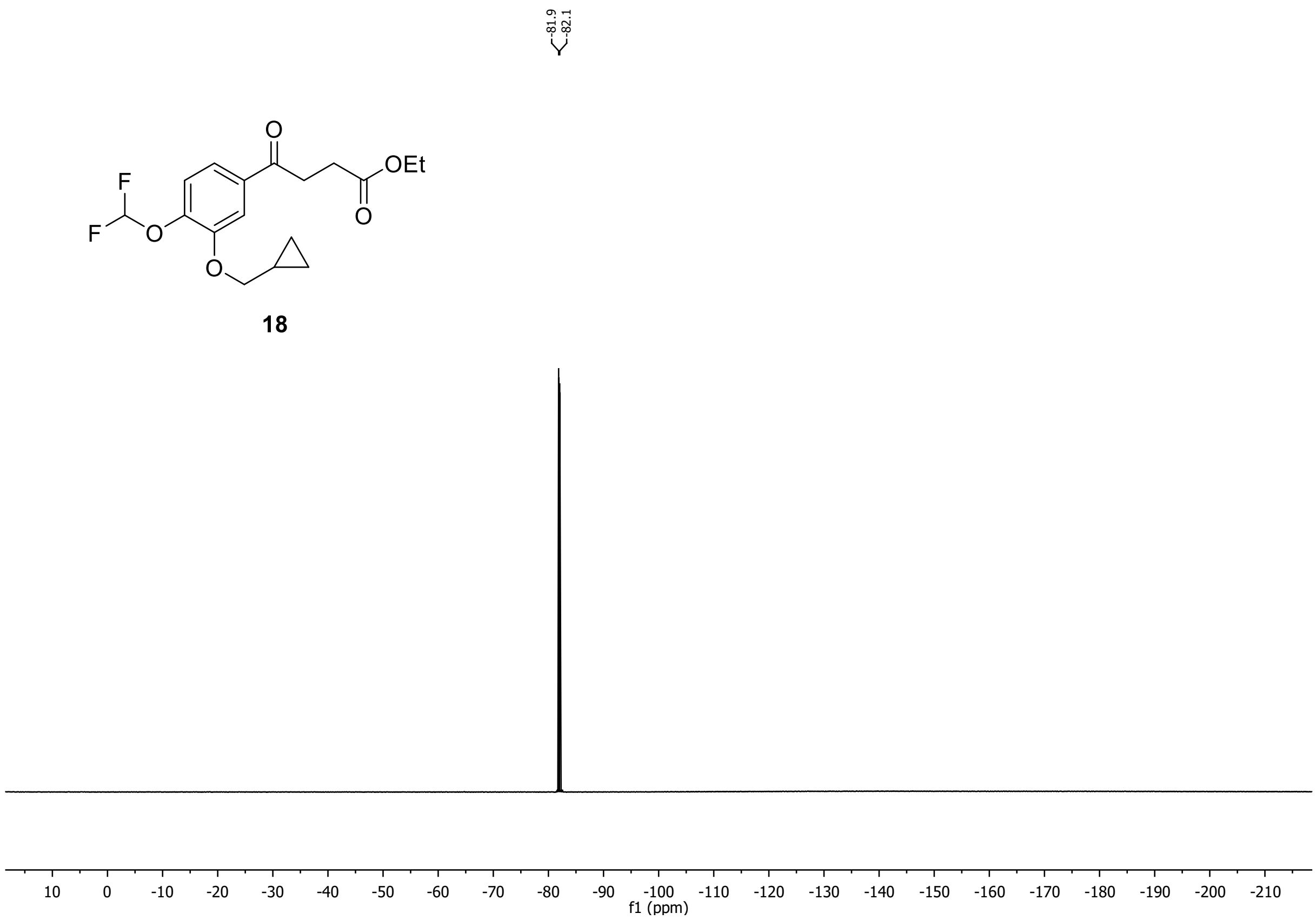

Figure S110. ^{19}F NMR spectrum (376 MHz, $CDCl_3$) of **18**.

Ethyl 4-(4-(*N*,*N*-dipropylsulfamoyl)phenyl)-4-oxobutanoate (19):

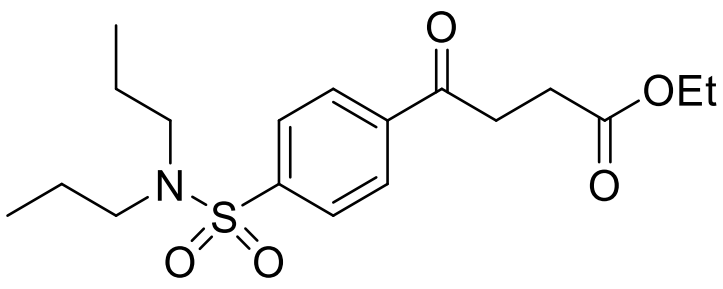

19

$C_{18}H_{27}NO_5S$ (369.48 g/mol)

Following **GP-B**, **19** was synthesized using **S19** (378 mg, 1.0 mmol, 1.0 equiv.) and ethyl acrylate (200 mg, 2.0 mmol, 2 equiv.). Purification by flash column chromatography (*n*-hexane/EtOAc, gradient 100:0 to 0:100 over 10 CV) afforded **19** (117 mg, 317 µmol, 32%) as yellow oil.

R_f: 0.74 (*n*-hexane/EtOAc 60:40).

^{1}H NMR (400 MHz, $CDCl_3$, δ): 8.05 (d, *J* = 8.4 Hz, 2H), 7.86 (d, *J* = 8.4 Hz, 2H), 4.12 (q, *J* = 7.1 Hz, 2H), 3.29 (t, *J* = 6.5 Hz, 2H), 3.10 – 3.03 (m, 4H), 2.74 (t, *J* = 6.5 Hz, 2H), 1.58 – 1.44 (m, 4H, *residual water under compound peak*), 1.23 (t, *J* = 7.1 Hz, 3H), 0.83 (t, *J* = 7.4 Hz, 6H).

^{13}C{^{1}H} NMR (101 MHz, $CDCl_3$, δ): 197.2, 172.7, 144.3, 139.3, 128.7, 127.3, 60.8, 50.0, 33.7, 28.2, 22.0, 14.2, 11.2.

HR-MS (ESI): m/z calc for $[M+H]^+$ 370.16827, found 370.16898 (err. 1.92 ppm).

IR (ATR, $\tilde{\nu}$ [cm^{-1}]): 2968 (w), 2932 (w), 2875 (w), 1731 (s), 1690 (s), 1460 (w), 1396 (w), 1373 (w), 1337 (s), 1299 (m), 1250 (w), 1213 (m), 1154 (vs), 1090 (m), 1029 (m), 986 (s), 893 (w), 867 (w), 833 (w), 787 (m), 733 (s).

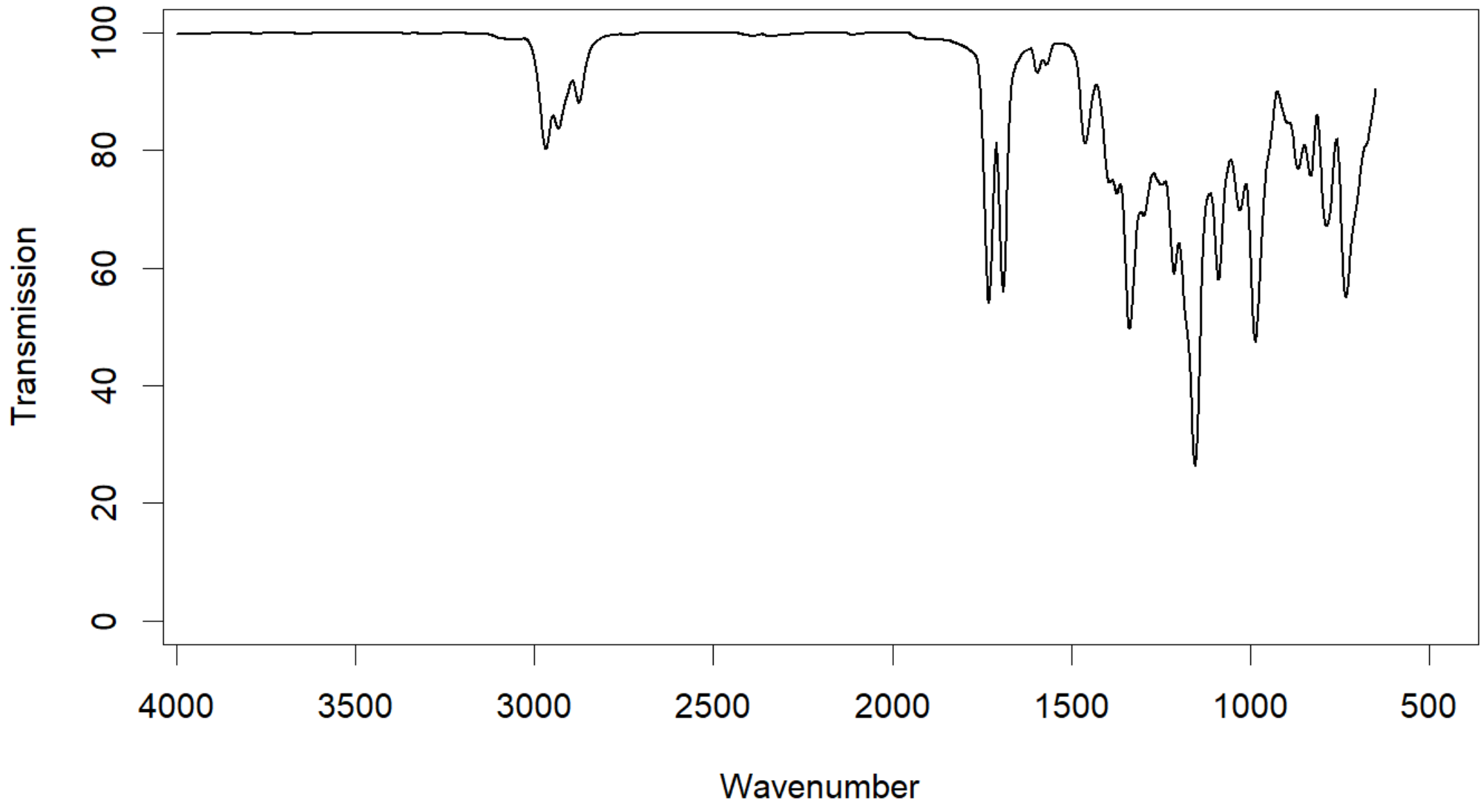

Figure S111. IR spectrum (ATR, neat) of **19**.

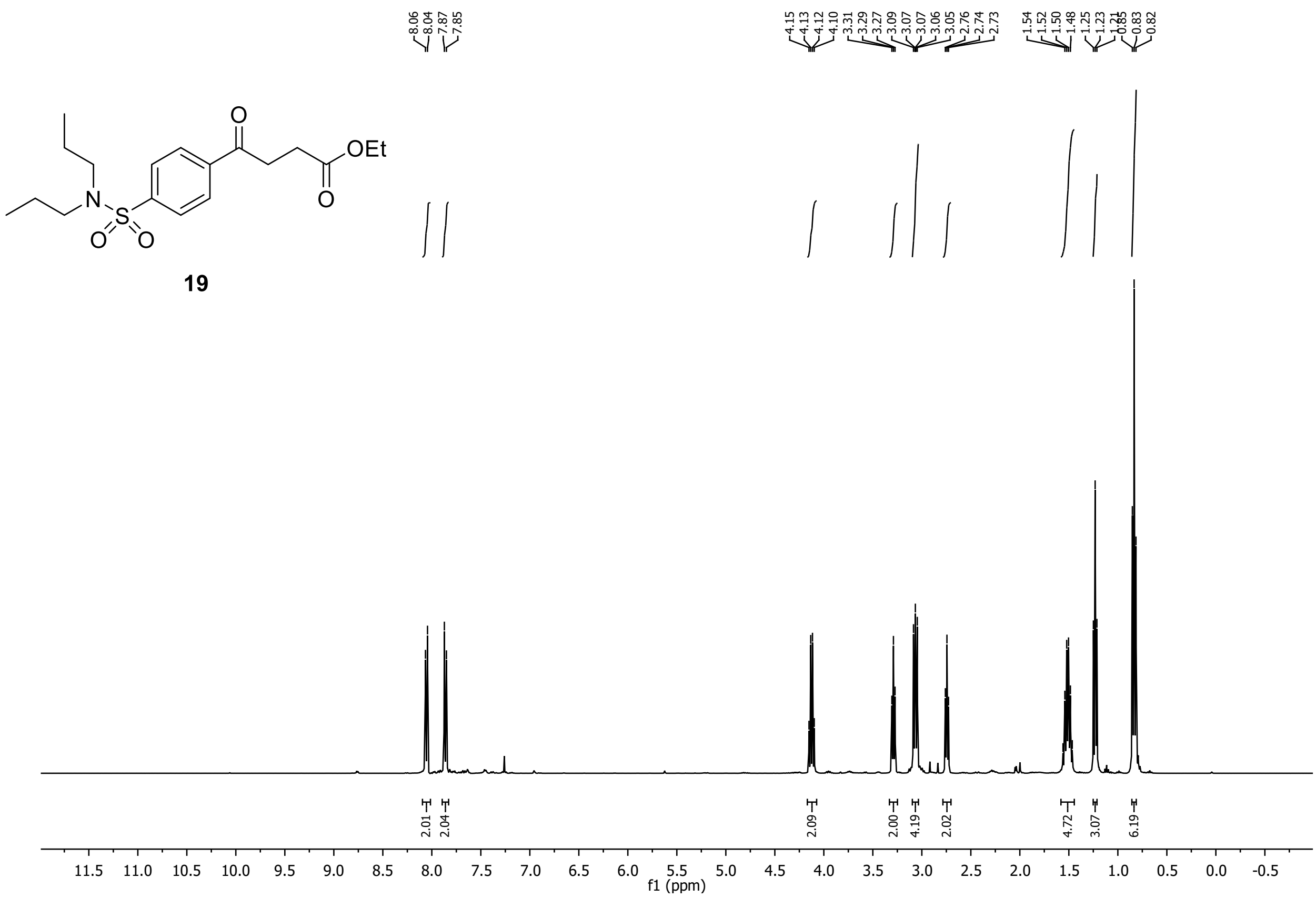

Figure S112. ^{1}H NMR spectrum (400 MHz, $CDCl_3$) of **19**.

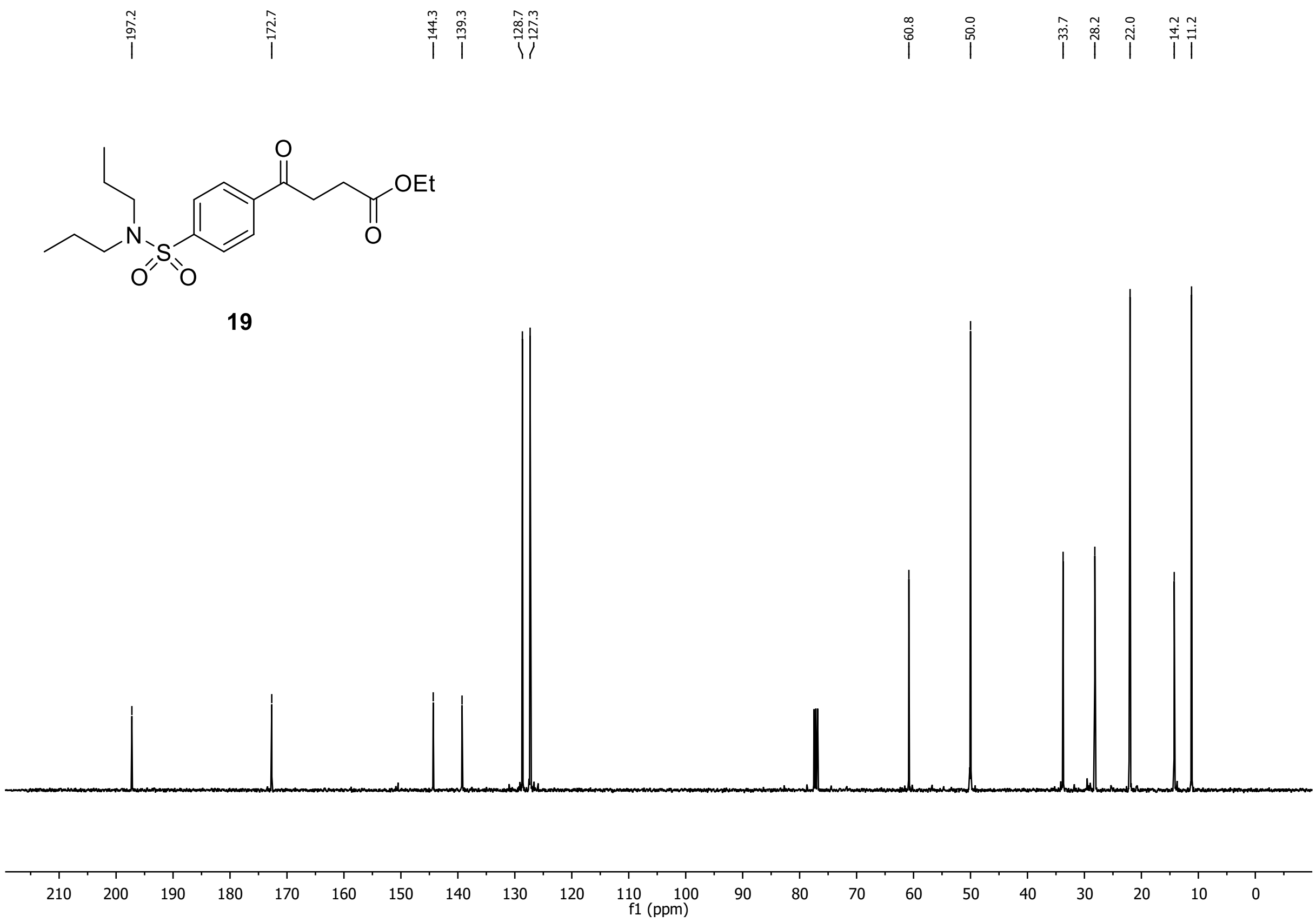

Figure S113. ^{13}C NMR spectrum (101 MHz, $CDCl_3$) of **19**.

Ethyl (*Z*)-4-oxohenicos-12-enoate (20):

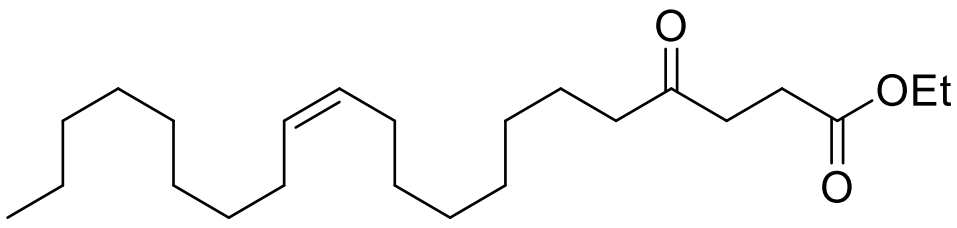

20

$C_{23}H_{42}O_3$ (366.59 g/mol)

Following **GP-3**, **20** was synthesized using **S20** (375 mg, 1.0 mmol, 1.0 equiv.) and ethyl acrylate (200 mg, 2.0 mmol, 2 equiv.). The reaction time was prolonged to 2 h. Purification by flash column chromatography (*n*-hexane/EtOAc, gradient 100:0 to 70:30 over 10 CV, detection of spots with $KMnO_4$) afforded **20** (178 mg, 486 µmol, 49%) as colorless oil.

R$_f$: 0.67 (*n*-hexane/EtOAc 90:10).

^{1}H NMR (400 MHz, $CDCl_3$, δ): 5.40 – 5.30 (m, 2H), 4.12 (q, *J* = 7.1 Hz, 2H), 2.71 (t, *J* = 6.6 Hz, 2H), 2.57 (t, *J* = 6.6 Hz, 2H), 2.44 (t, *J* = 7.4 Hz, 2H), 2.07 – 1.97 (m, 3H), 1.78 – 1.49 (m, 3H), 1.47 – 1.06 (m, 23H), 0.91 – 0.84 (m, 3H).

^{13}C{^{1}H} NMR (101 MHz, $CDCl_3$, δ): 209.3, 173.0, 130.4, 130.2, 130.1, 129.9, 128.2, 128.1, 60.7, 43.0, 37.2, 32.1, 31.7, 29.9, 29.85, 29.81, 29.76, 29.7, 29.6, 29.5, 29.33 29.26, 28.2, 27.4, 25.8, 24.0, 22.8, 22.7, 14.33, 14.26, 14.2.

HR-MS (APCI): calc. for $[M+H]^+$ 367.32067, found 367.32003 (err. 1.76 ppm).

IR (ATR, $\tilde{\nu}$ [cm^{-1}]): 2924 (s), 2853 (m), 1716 (s), 1459 (w), 1407 (w), 1370 (m), 1303 (w), 1180 (m), 1094 (m), 1028 (m), 975 (w), 856 (w), 785 (w), 725 (w), 692 (w).

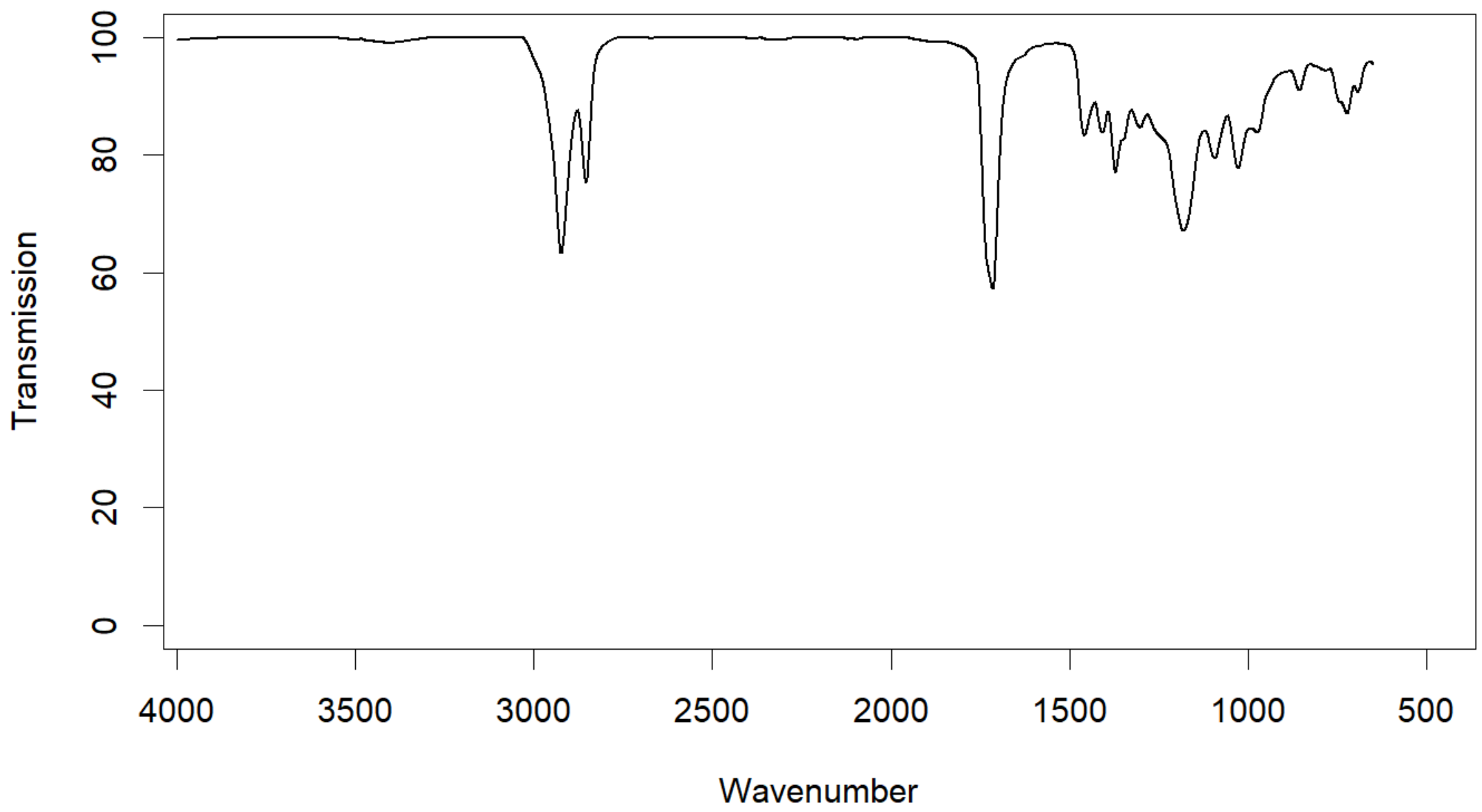

Figure S114. IR spectrum (ATR, neat) of **20**.

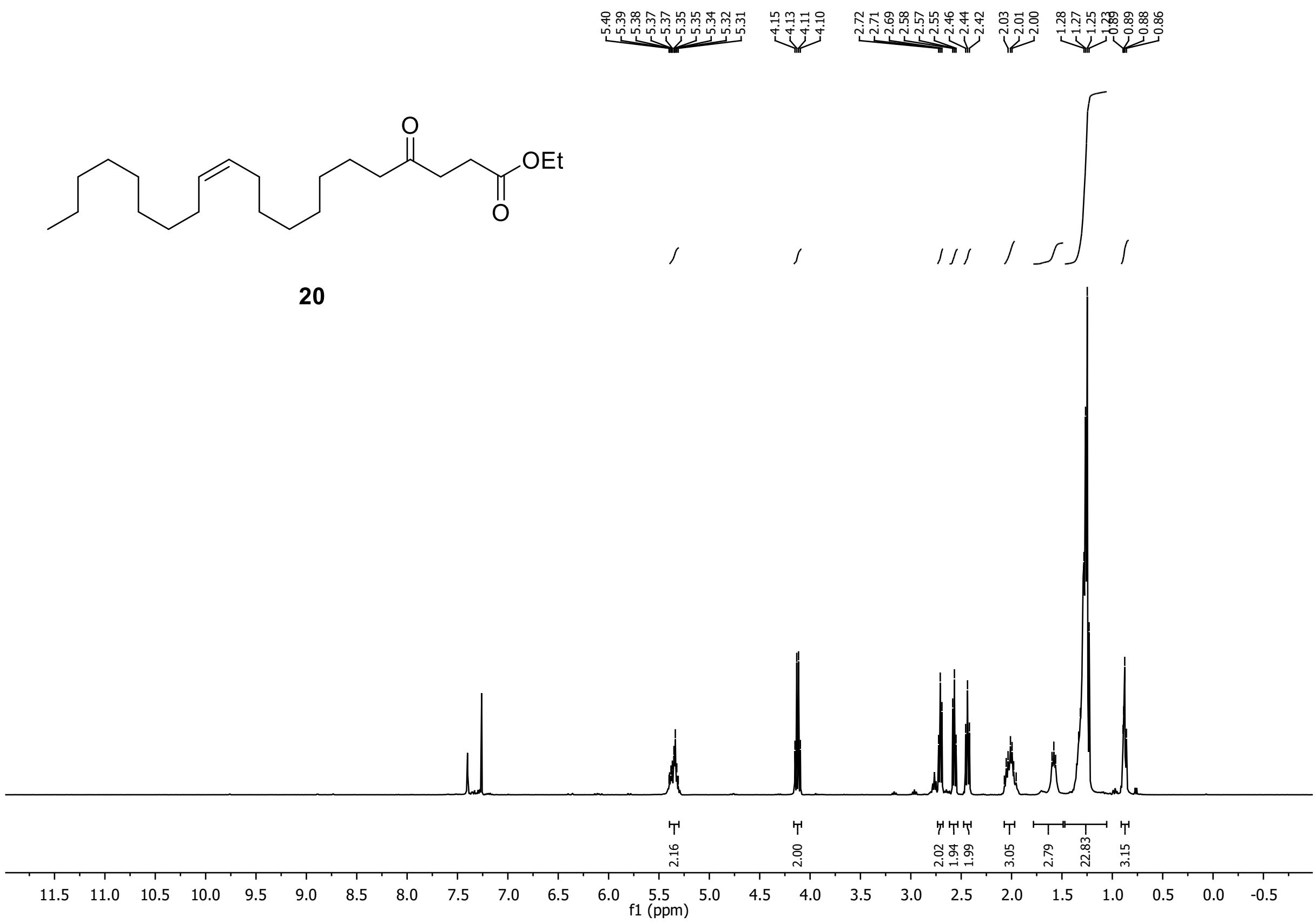

Figure S115. ^{1}H NMR (400 MHz, $CDCl_3$) spectrum of **20**.

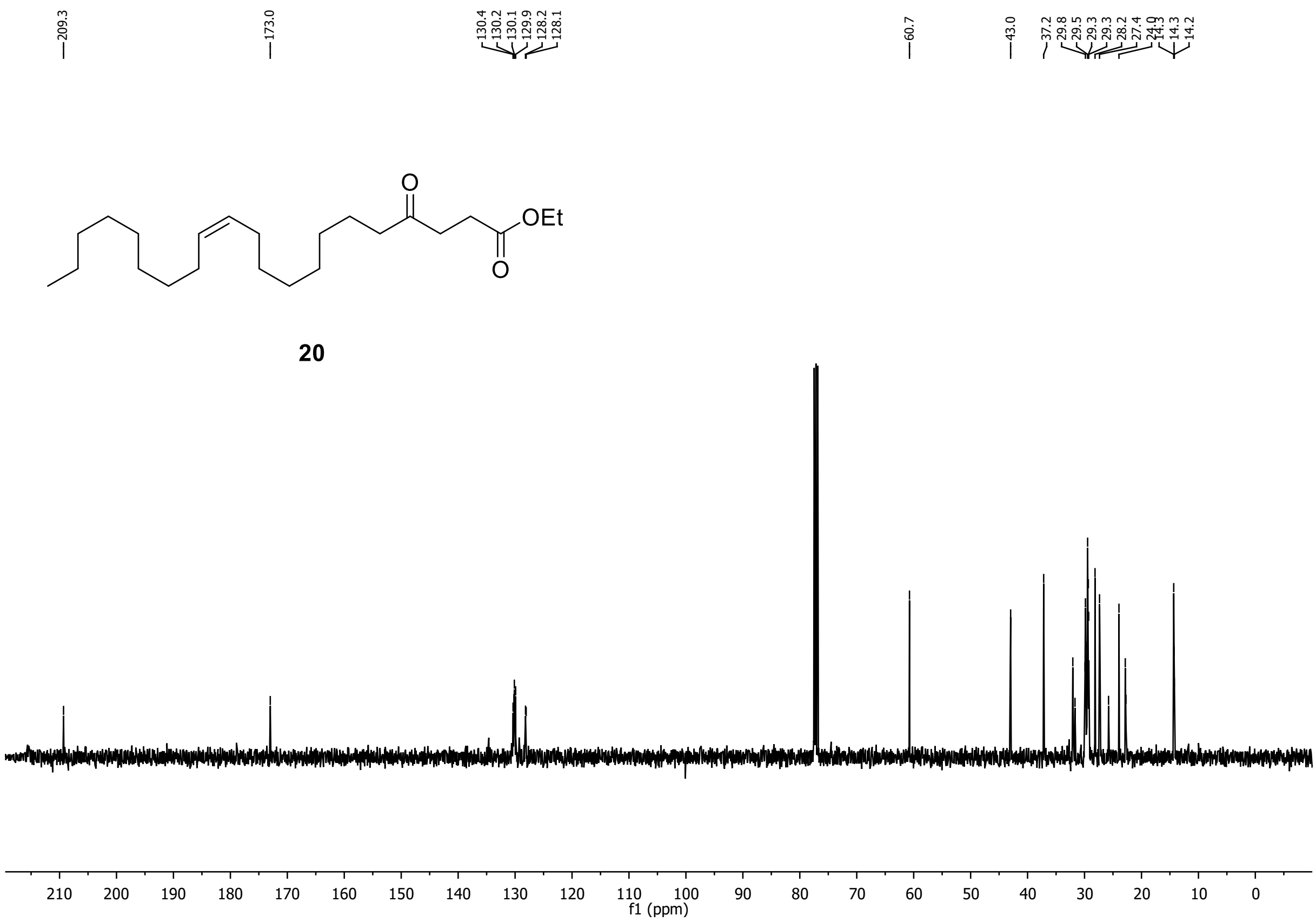

20

Figure S116. ^{13}C NMR (101 MHz, $CDCl_3$) spectrum of **20**.

***tert*-Butyl 4-oxo-4-phenylbutanoate (21):**

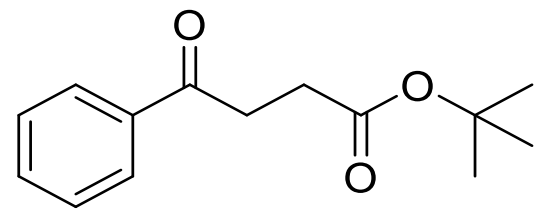

21

$C_{14}H_{18}O_3$ (234.30 g/mol)

Following **GP-B**, **21** was synthesized using **S1** (214 mg, 1.0 mmol, 1.0 equiv.) and *tert*-butyl acrylate (256 mg, 2.0 mmol, 2 equiv.). Purification by flash column chromatography (*n*-hexane/EtOAc, gradient 100:0 to 70:30 over 10 CV) afforded **21** (153 mg, 653 µmol, 65%) as colorless oil. Conforms to reported analytical data.[22]

R_f: 0.65 (*n*-hexane/EtOAc 80:20).

^{1}H NMR (400 MHz, $CDCl_3$, δ): 8.02 – 7.94 (m, 2H), 7.60 – 7.51 (m, 1H), 7.50 – 7.41 (m, 2H), 3.25 (t, J = 6.7 Hz, 2H), 2.68 (t, J = 6.7 Hz, 2H), 1.45 (s, 9H).

^{13}C{^{1}H} NMR (101 MHz, $CDCl_3$, δ): 198.5, 172.3, 136.9, 133.2, 128.7, 128.1, 80.7, 33.6, 29.6, 28.2.

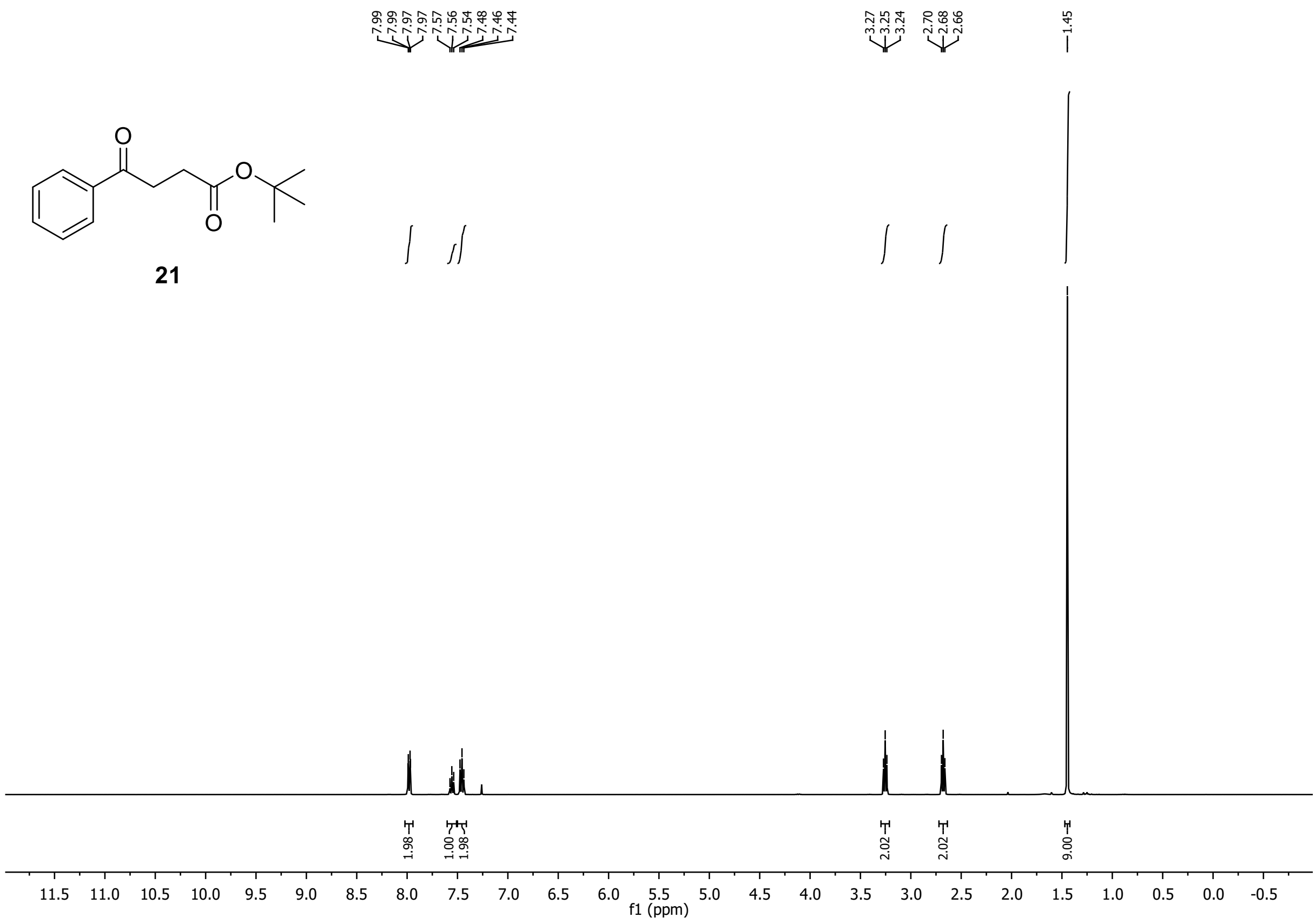

Figure S117. ^{1}H NMR spectrum (400 MHz, $CDCl_3$) of **21**.

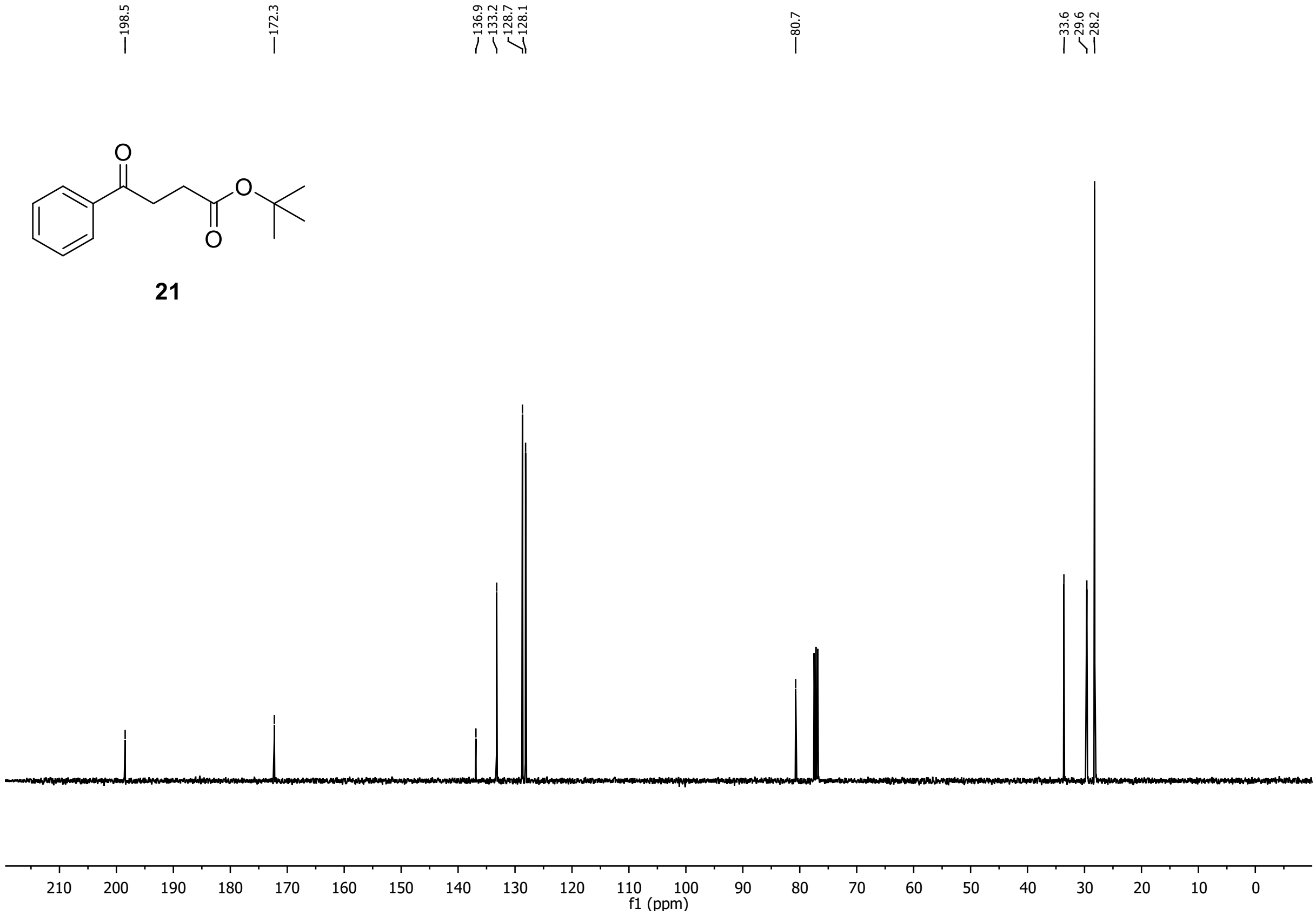

Figure S118. ^{13}C NMR spectrum (101 MHz, $CDCl_3$) of **21**.

Phenyl 4-oxo-4-phenylbutanoate (22):

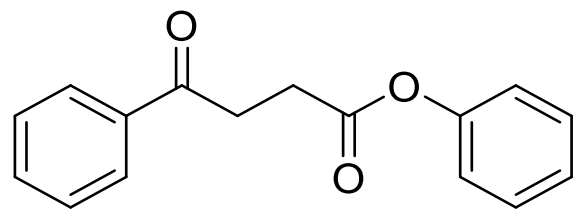

22

$C_{16}H_{14}O_3$ (254.29 g/mol)

Following **GP-B**, **22** was synthesized using **S1** (214 mg, 1.0 mmol, 1.0 equiv.) and phenyl acrylate (296 mg, 2.0 mmol, 2 equiv.). Purification by flash column chromatography (*n*-hexane/EtOAc, gradient 100:0 to 70:30 over 10 CV) afforded **22** (178 mg, 700 µmol, 70%) as yellow oil. Conforms to reported analytical data.[21]

R$_f$: 0.48 (*n*-hexane/EtOAc 80:20).

^{1}H NMR (400 MHz, $CDCl_3$, δ): 8.06 – 7.97 (m, 2H), 7.63 – 7.54 (m, 1H), 7.53 – 7.44 (m, 2H), 7.43 – 7.33 (m, 2H), 7.24 – 7.19 (m, 1H), 7.16 – 7.08 (m, 2H), 3.44 (t, *J* = 6.5 Hz, 2H), 3.02 (t, *J* = 6.5 Hz, 2H).

^{13}C{^{1}H} NMR (101 MHz, $CDCl_3$, δ): 198.0, 171.7, 150.9, 136.6, 133.5, 129.5, 128.8, 128.2, 125.9, 121.7, 33.6, 28.6.

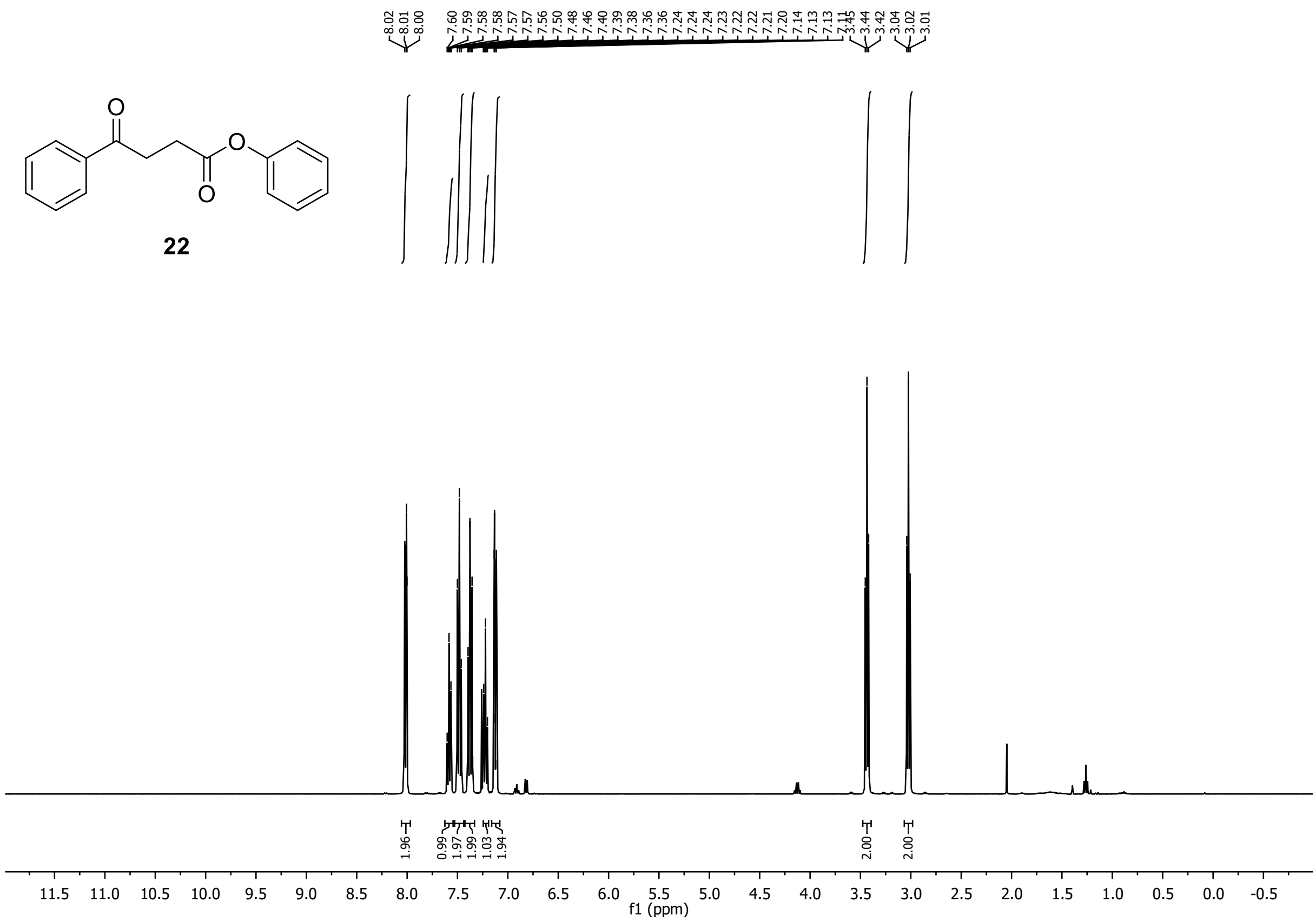

Figure S119. ^{1}H NMR spectrum (400 MHz, $CDCl_3$) of **22**.

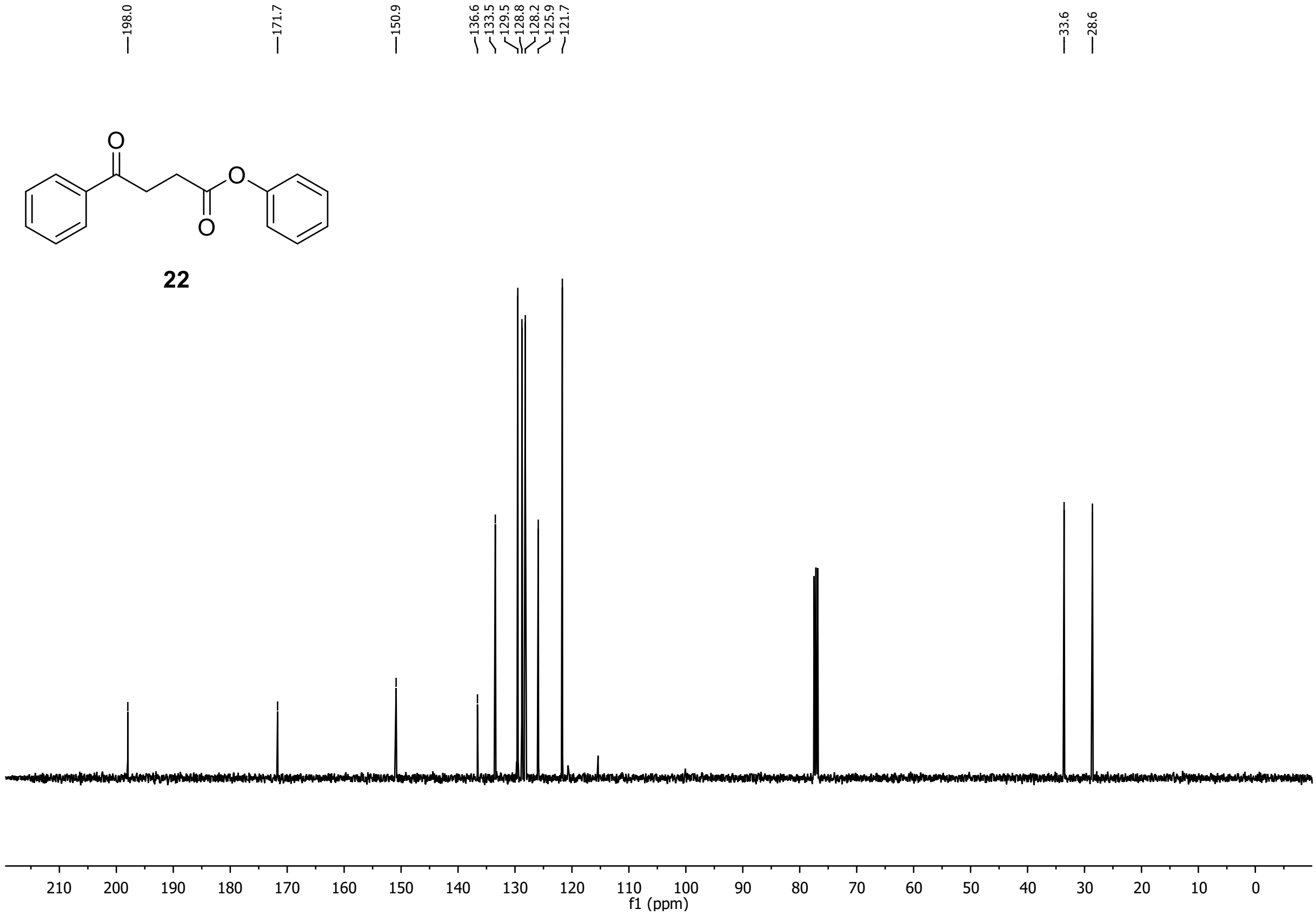

Figure S120. ^{13}C NMR spectrum (101 MHz, $CDCl_3$) of **22**.

Benzyl 4-oxo-4-phenylbutanoate (23):

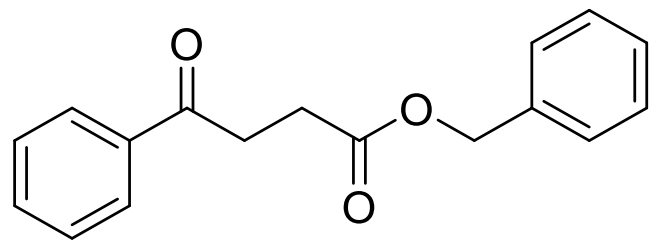

23

$C_{17}H_{16}O_3$ (268.31 g/mol)

Following **GP-B**, **23** was synthesized using **S1** (214 mg, 1.0 mmol, 1.0 equiv.) and benzyl acrylate (324 mg, 2.0 mmol, 2 equiv.). Purification by flash column chromatography (*n*-hexane/EtOAc, gradient 100:0 to 70:30 over 10 CV) afforded **23** (215 mg, 798 µmol, 80%) as colorless solid. Conforms to reported analytical data.[19]

R_f: 0.56 (*n*-hexane/EtOAc 80:20).

^{1}H NMR (400 MHz, $CDCl_3$, δ): 8.03 – 7.95 (m, 2H), 7.62 – 7.54 (m, 1H), 7.51 – 7.43 (m, 2H), 7.40 – 7.29 (m, 5H), 5.16 (s, 2H), 3.34 (t, J = 6.6 Hz, 2H), 2.84 (t, J = 6.6 Hz, 2H).

^{13}C{^{1}H} NMR (101 MHz, $CDCl_3$, δ): 198.1, 172.9, 136.7, 136.0, 133.3, 128.7, 128.7, 128.7, 128.3, 128.2, 66.6, 33.5, 28.4.

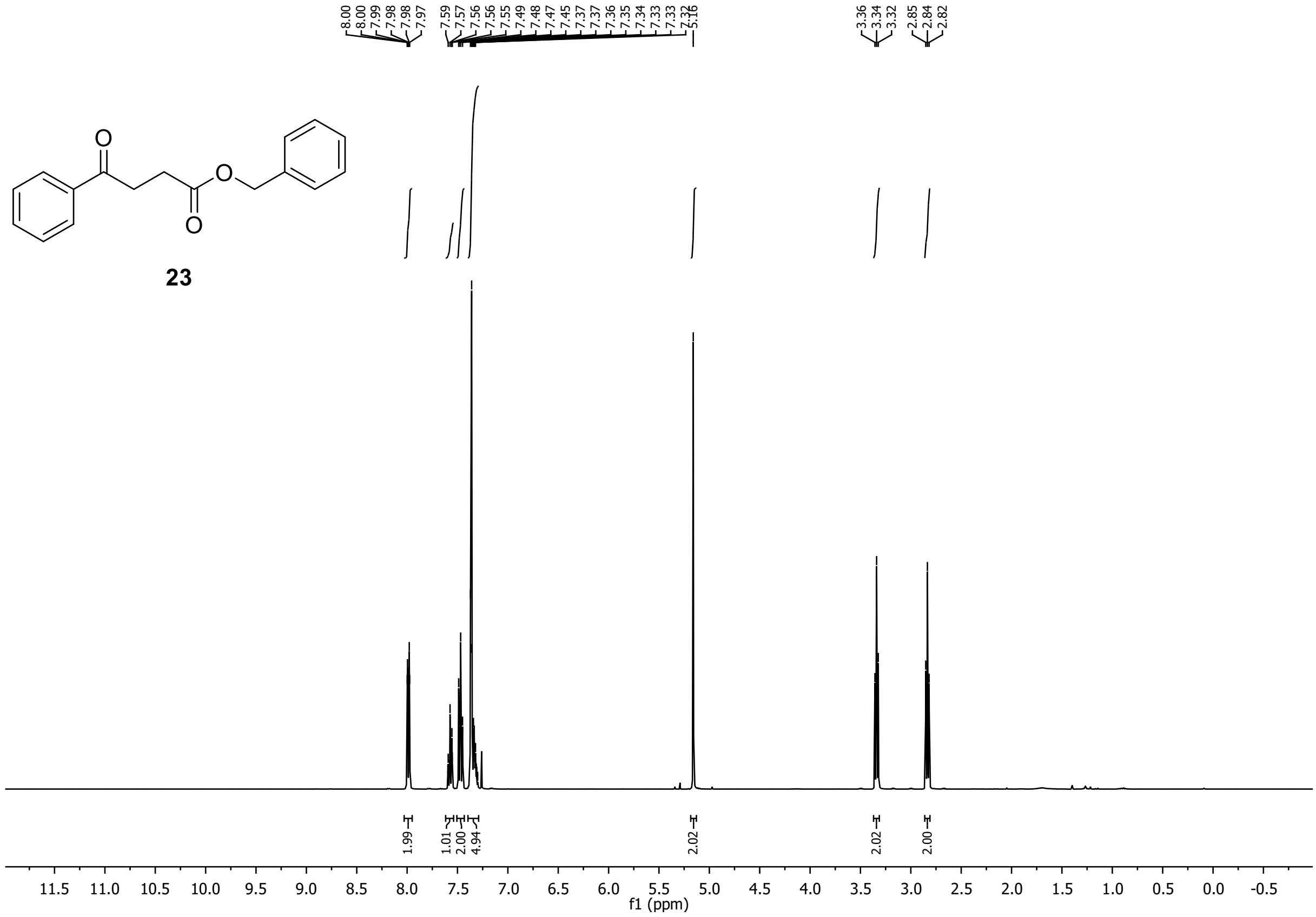

Figure S121. ^{1}H NMR spectrum (400 MHz, $CDCl_3$) of **23**.

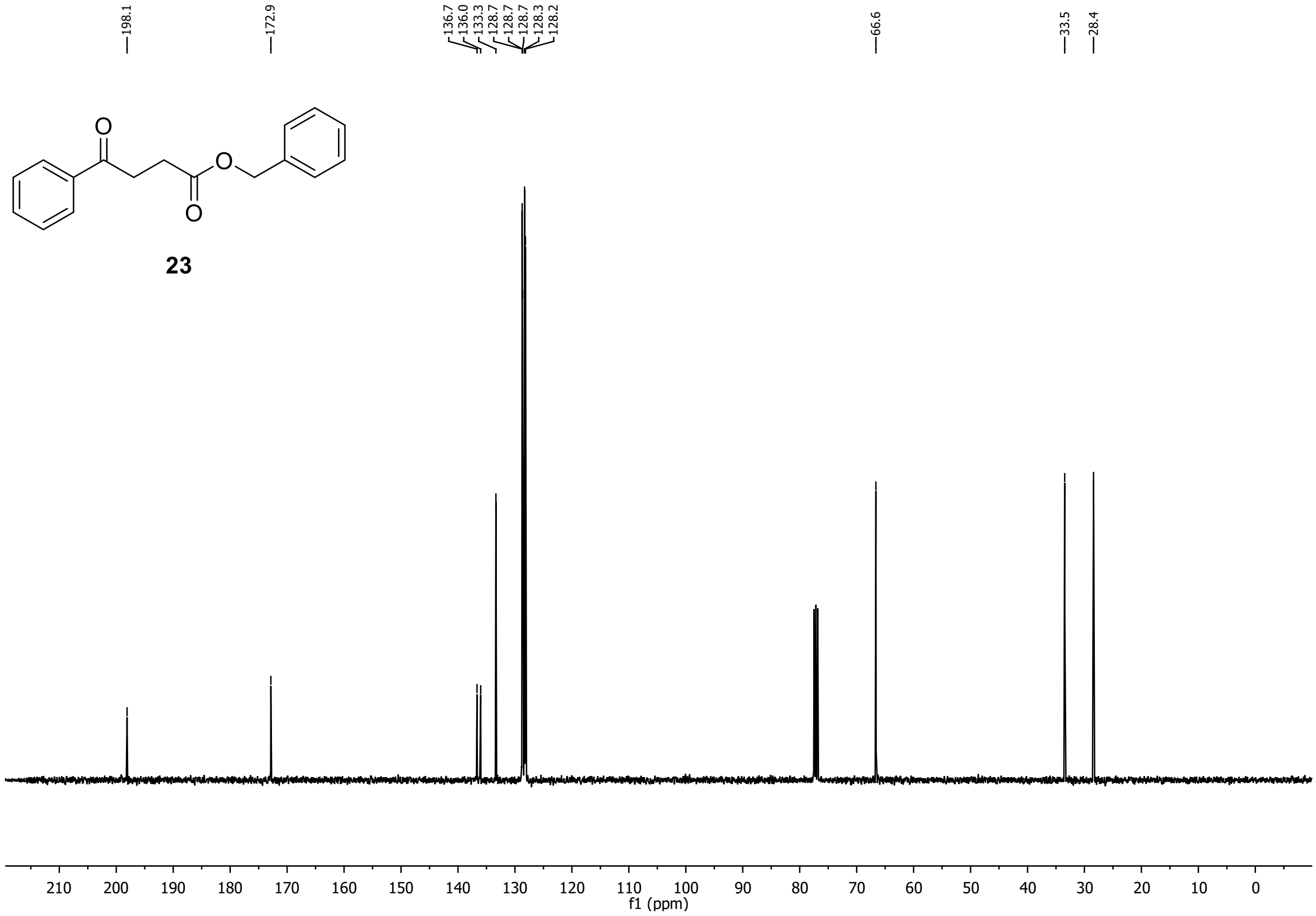

Figure S122. ^{13}C NMR spectrum (101 MHz, $CDCl_3$) of **23**.

(1*R*,2*S*,5*R*)-2-Isopropyl-5-methylcyclohexyl 4-oxo-4-phenylbutanoate (24):

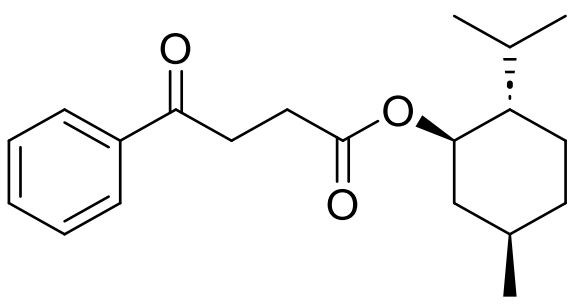

24

$C_{20}H_{28}O_3$ (316.44 g/mol)

Following **GP-B**, **24** was synthesized using **S1** (214 mg, 1.0 mmol, 1.0 equiv.) and **S24** (421 mg, 2.0 mmol, 2 equiv.). Purification by flash column chromatography (*n*-hexane/EtOAc, gradient 100:0 to 70:30 over 10 CV) afforded **24** (182 mg, 575 µmol, 58%) as colorless solid. Conforms to reported analytical data.[27]

R_f: 0.83 (*n*-hexane/EtOAc 70:30).

^{1}H NMR (400 MHz, $CDCl_3$, δ): 8.03 – 7.95 (m, 2H), 7.61 – 7.52 (m, 1H), 7.51 – 7.42 (m, 2H), 4.70 (td, *J* = 10.9, 4.4 Hz, 1H), 3.40 – 3.21 (m, 2H), 2.75 (t, *J* = 6.7 Hz, 2H), 2.05 – 1.97 (m, 1H), 1.93 – 1.81 (m, 1H), 1.72 – 1.62 (m, 2H), 1.53 – 1.41 (m, 1H), 1.41 – 1.31 (m, 1H), 1.12 – 0.93 (m, 2H), 0.92 – 0.83 (m, 7H), 0.76 (d, *J* = 6.9 Hz, 3H).

^{13}C{^{1}H} NMR (101 MHz, $CDCl_3$, δ): 198.3, 172.6, 136.8, 133.3, 128.7, 128.2, 74.7, 47.2, 41.0, 34.4, 33.6, 31.5, 28.8, 26.4, 23.6, 22.2, 20.9, 16.5.

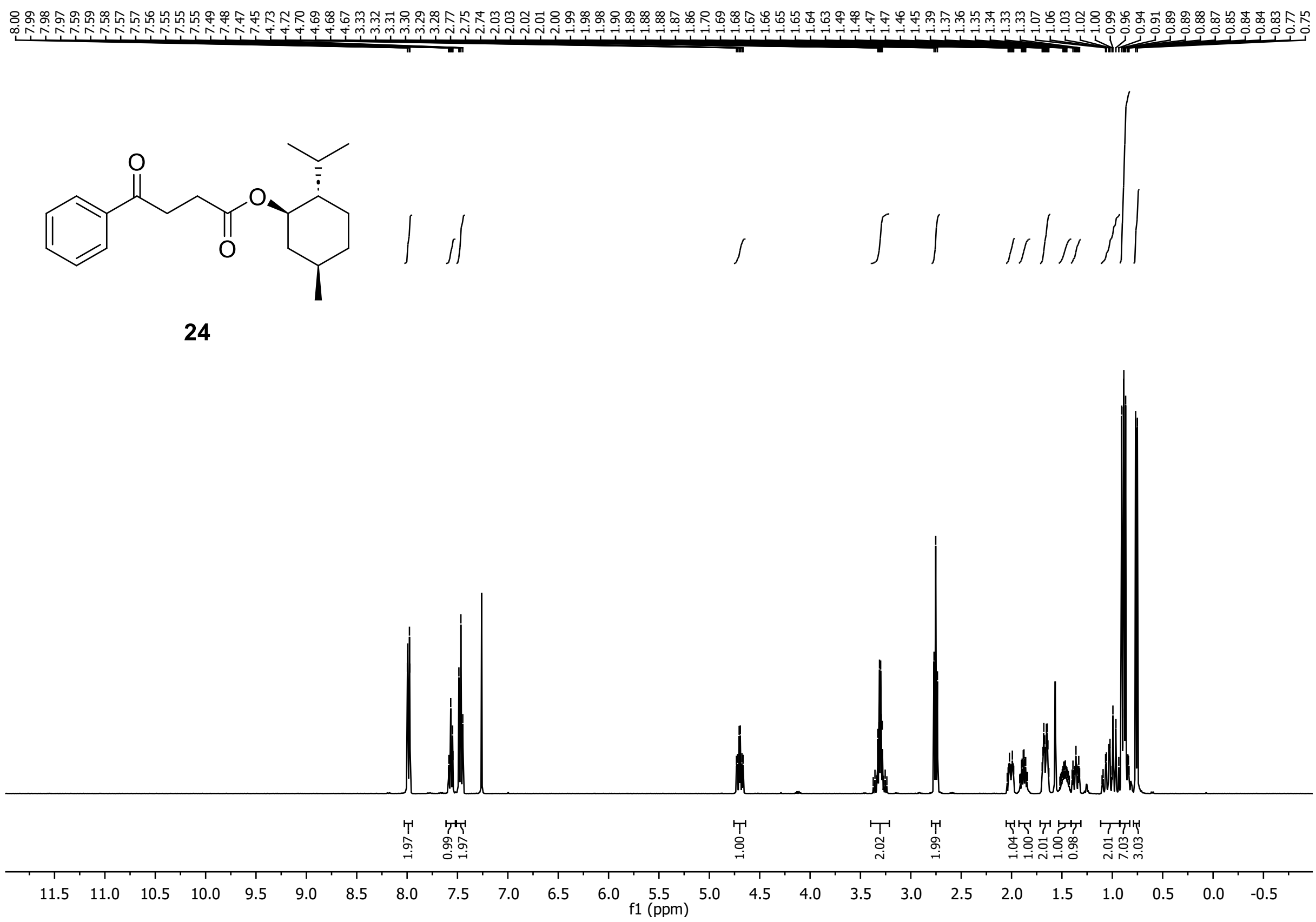

Figure S123. ^{1}H NMR spectrum (400 MHz, $CDCl_3$) of **24**.

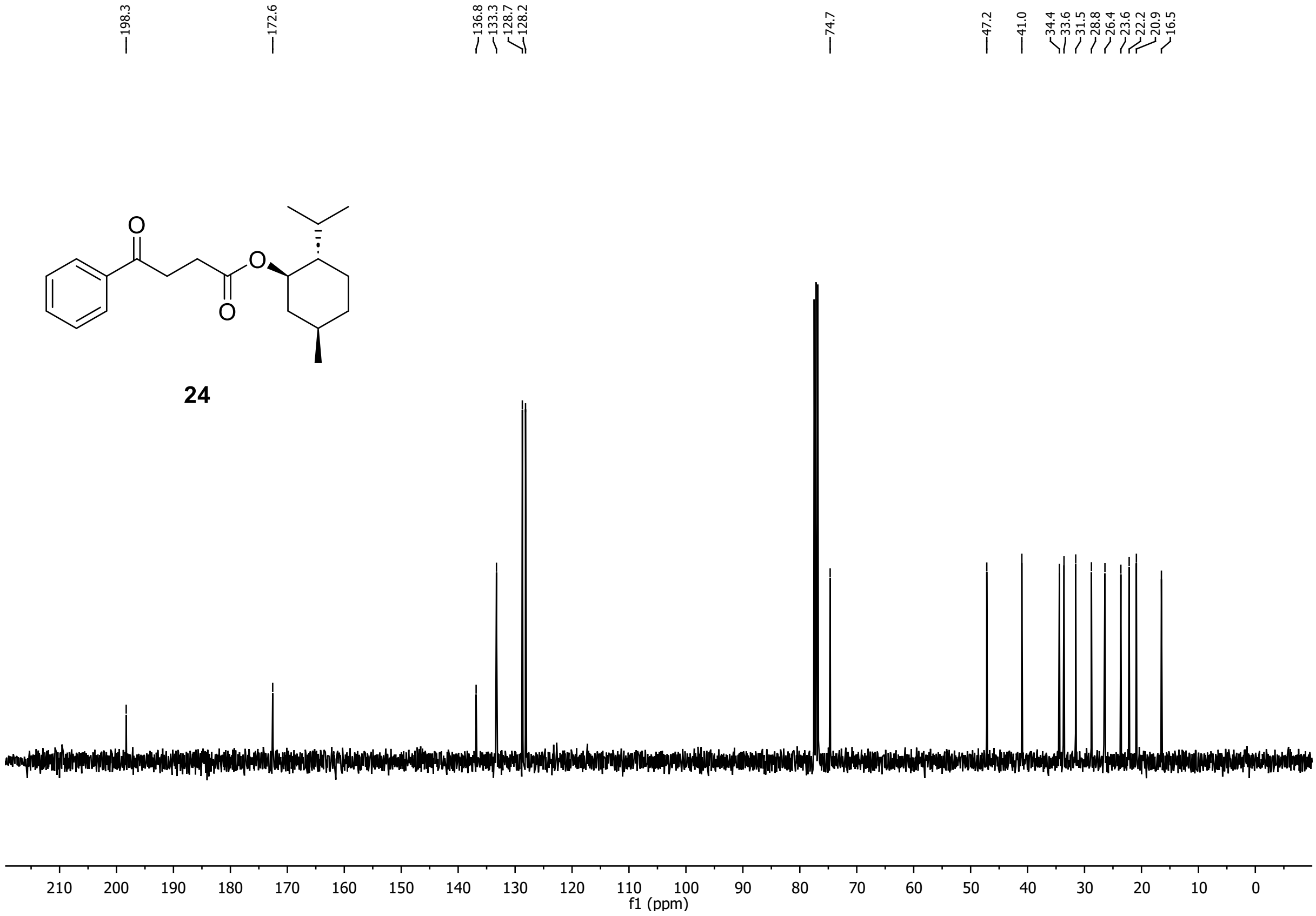

Figure S124. ^{13}C NMR spectrum (101 MHz, $CDCl_3$) of **24**.

3-(2-oxo-2-phenylethyl)dihydrofuran-2(3*H*)-one (25):

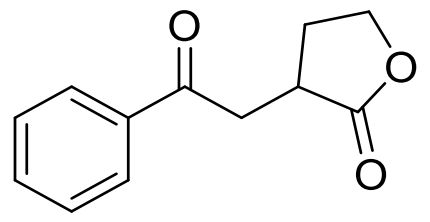

25

$C_{12}H_{12}O_3$ (204.23 g/mol)

Following **GP-B**, **25** was synthesized using **S1** (214 mg, 1.0 mmol, 1.0 equiv.) and 3-methylenedihydrofuran-2(3*H*)-one (196 mg, 2.0 mmol, 2 equiv.). Purification by flash column chromatography (*n*-hexane/EtOAc, gradient 100:0 to 70:30 over 10 CV) afforded **25** (112 mg, 543 µmol, 54%) as colorless oil. Conforms to reported analytical data.[28]

R$_f$: 0.32 (*n*-hexane/EtOAc 80:20).

^{1}H NMR (400 MHz, $CDCl_3$, δ): 8.01 – 7.93 (m, 2H), 7.63 – 7.56 (m, 1H), 7.52 – 7.44 (m, 2H), 4.44 (m, 1H), 4.33 – 4.23 (m, 1H), 3.72 – 3.61 (m, 1H), 3.23 – 3.10 (m, 2H), 2.71 – 2.59 (m, 1H), 2.07 – 1.90 (m, 1H).

^{13}C{^{1}H} NMR (101 MHz, $CDCl_3$, δ): 197.1, 179.3, 136.3, 133.7, 128.9, 128.2, 67.0, 39.5, 35.4, 29.3.

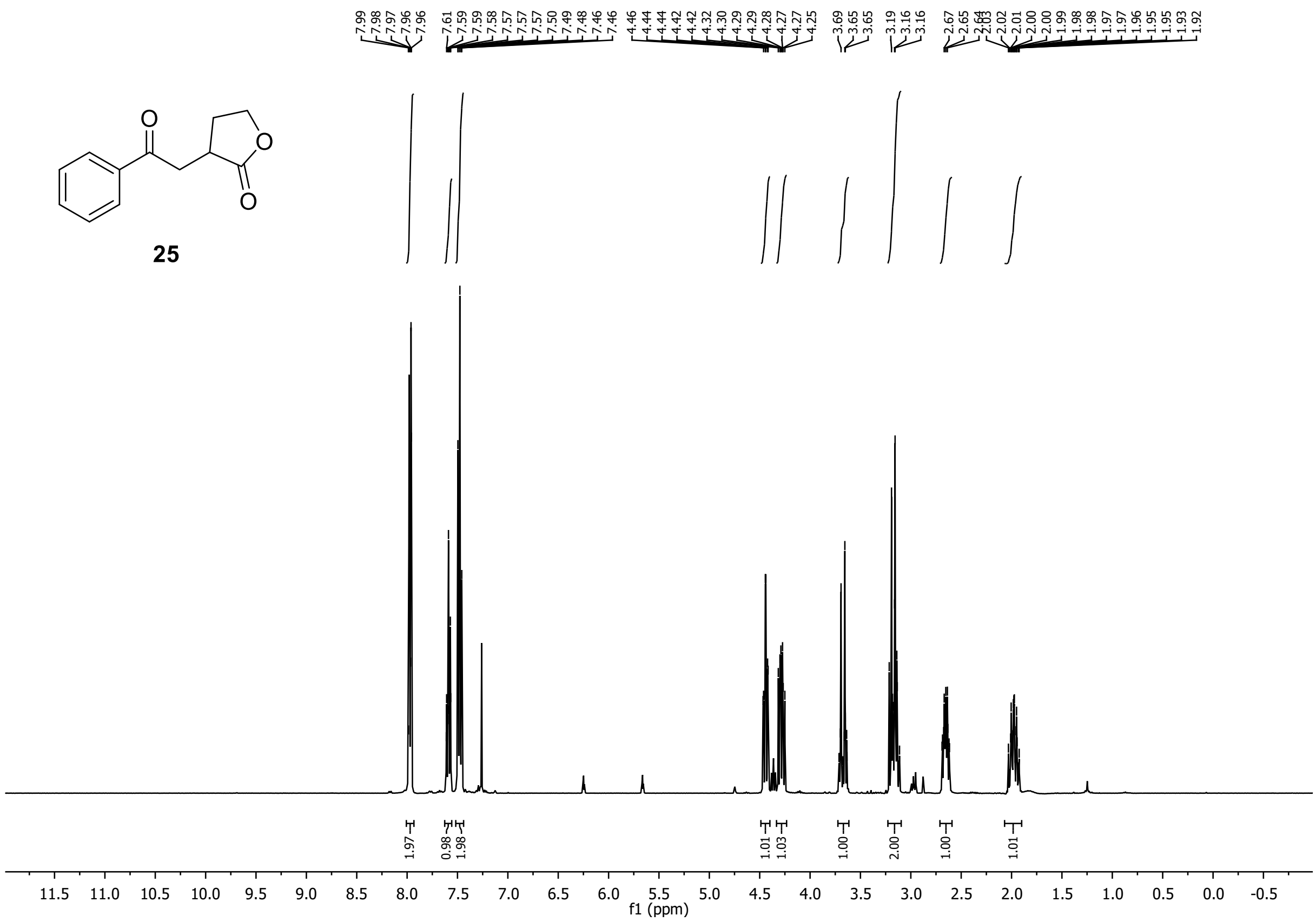

Figure S125. ^{1}H NMR spectrum (400 MHz, $CDCl_3$) of **25**.

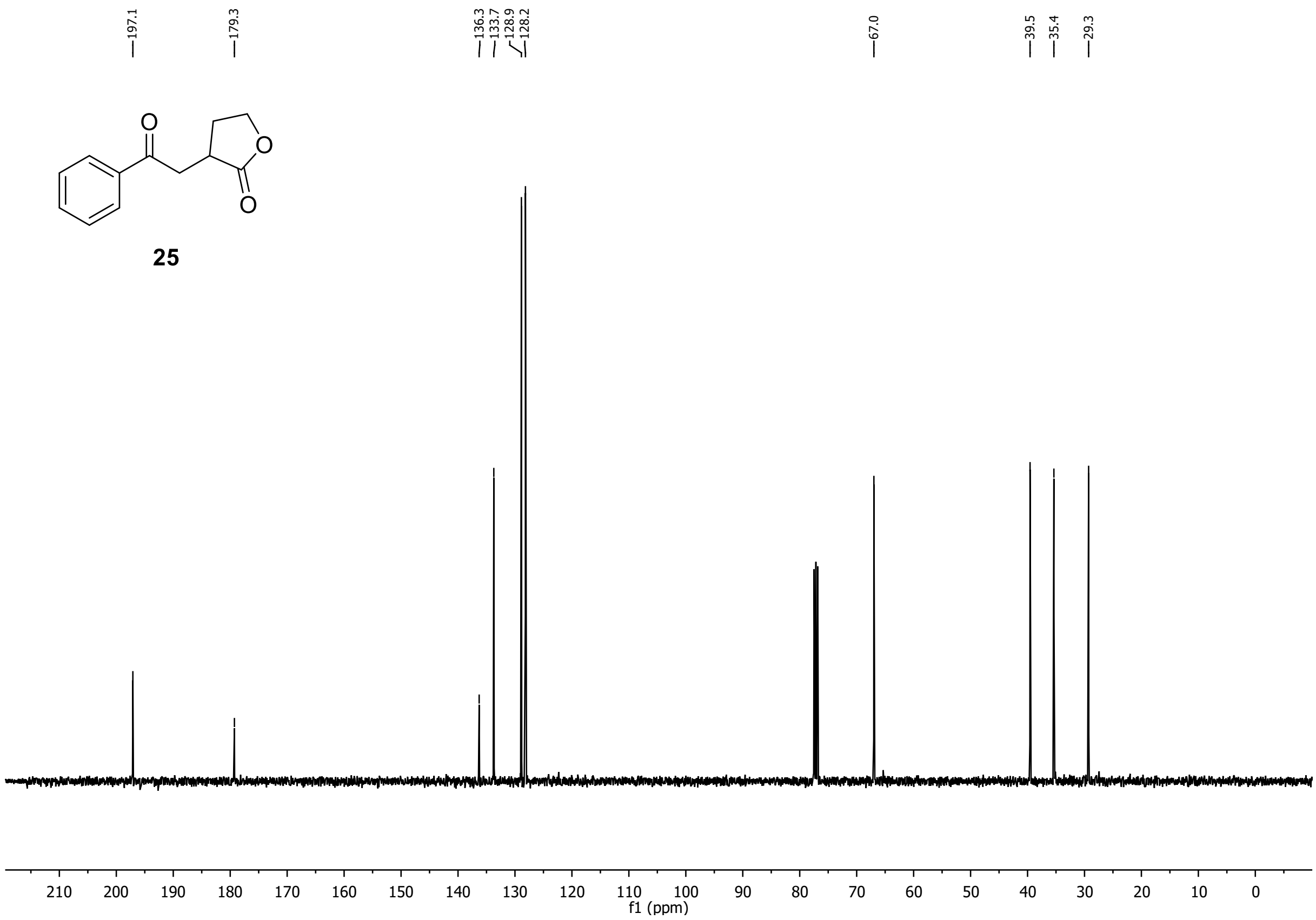

Figure S126. ^{13}C NMR spectrum (101 MHz, $CDCl_3$) of **25**.

1-Phenylpentane-1,4-dione (26):

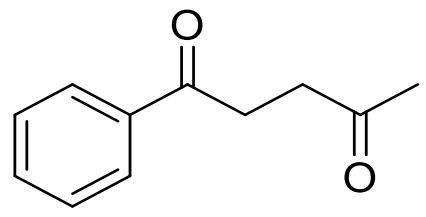

26

$C_{11}H_{12}O_2$ (176.22 g/mol)

Following **GP-B**, **26** was synthesized using **S1** (214 mg, 1.0 mmol, 1.0 equiv.) and methyl vinyl ketone (140 mg, 2.0 mmol, 2 equiv.). Purification by flash column chromatography (*n*-hexane/EtOAc, gradient 100:0 to 50:50 over 10 CV) afforded **26** (92 mg, 629 µmol, 63%) as yellow oil. Conforms to reported analytical data.[29]

R_f: 0.67 (*n*-hexane/EtOAc 60:40).

^{1}H NMR (400 MHz, $CDCl_3$, δ): 8.02 – 7.94 (m, 2H), 7.61 – 7.51 (m, 1H), 7.50 – 7.42 (m, 2H), 3.28 (t, *J* = 6.3 Hz, 2H), 2.89 (t, *J* = 6.3 Hz, 2H), 2.26 (s, 3H).

^{13}C{^{1}H} NMR (101 MHz, $CDCl_3$, δ): 207.4, 198.6, 136.8, 133.3, 128.7, 128.2, 37.2, 32.6, 30.2.

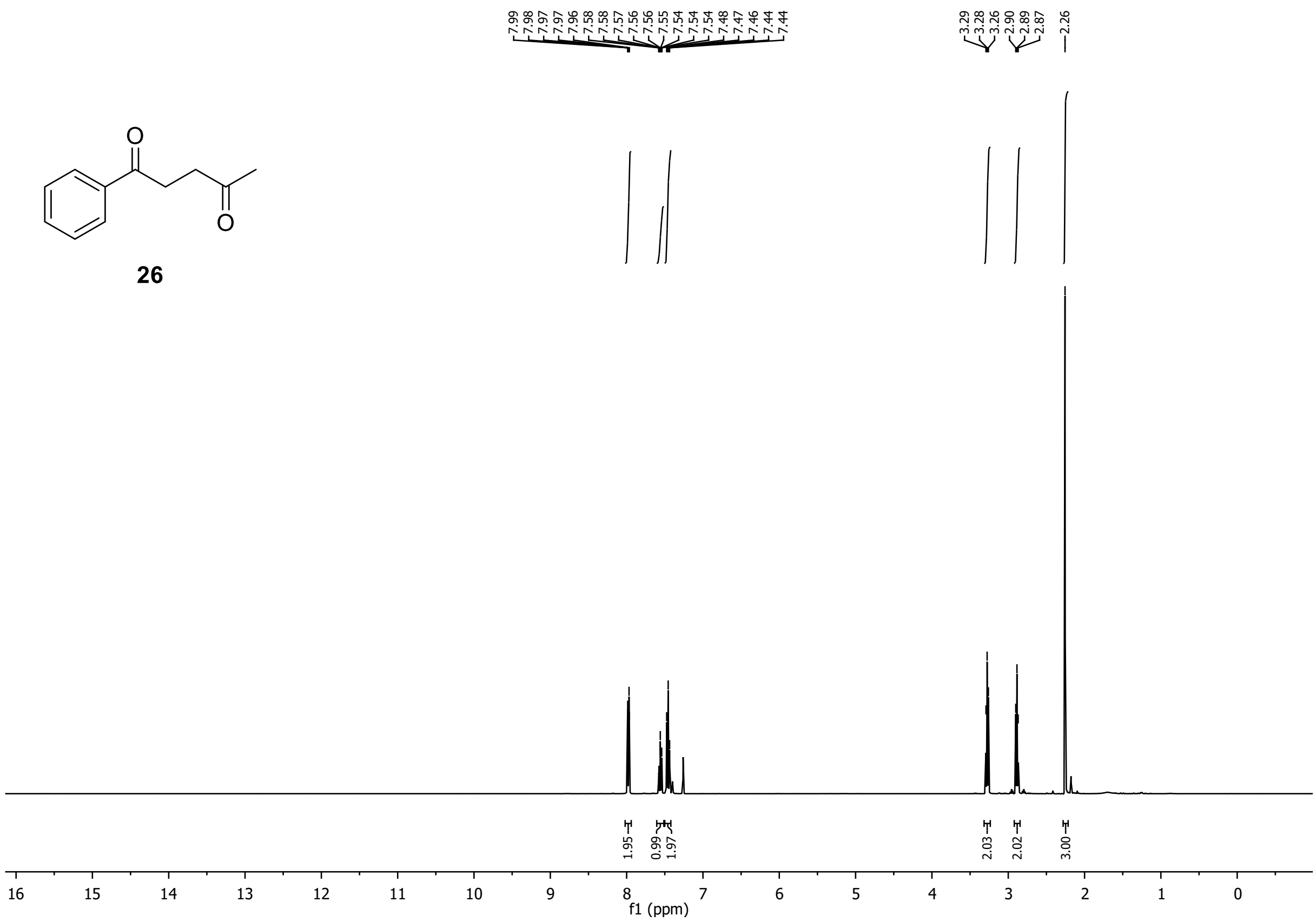

Figure S127. ^{1}H NMR spectrum (400 MHz, $CDCl_3$) of **26**.

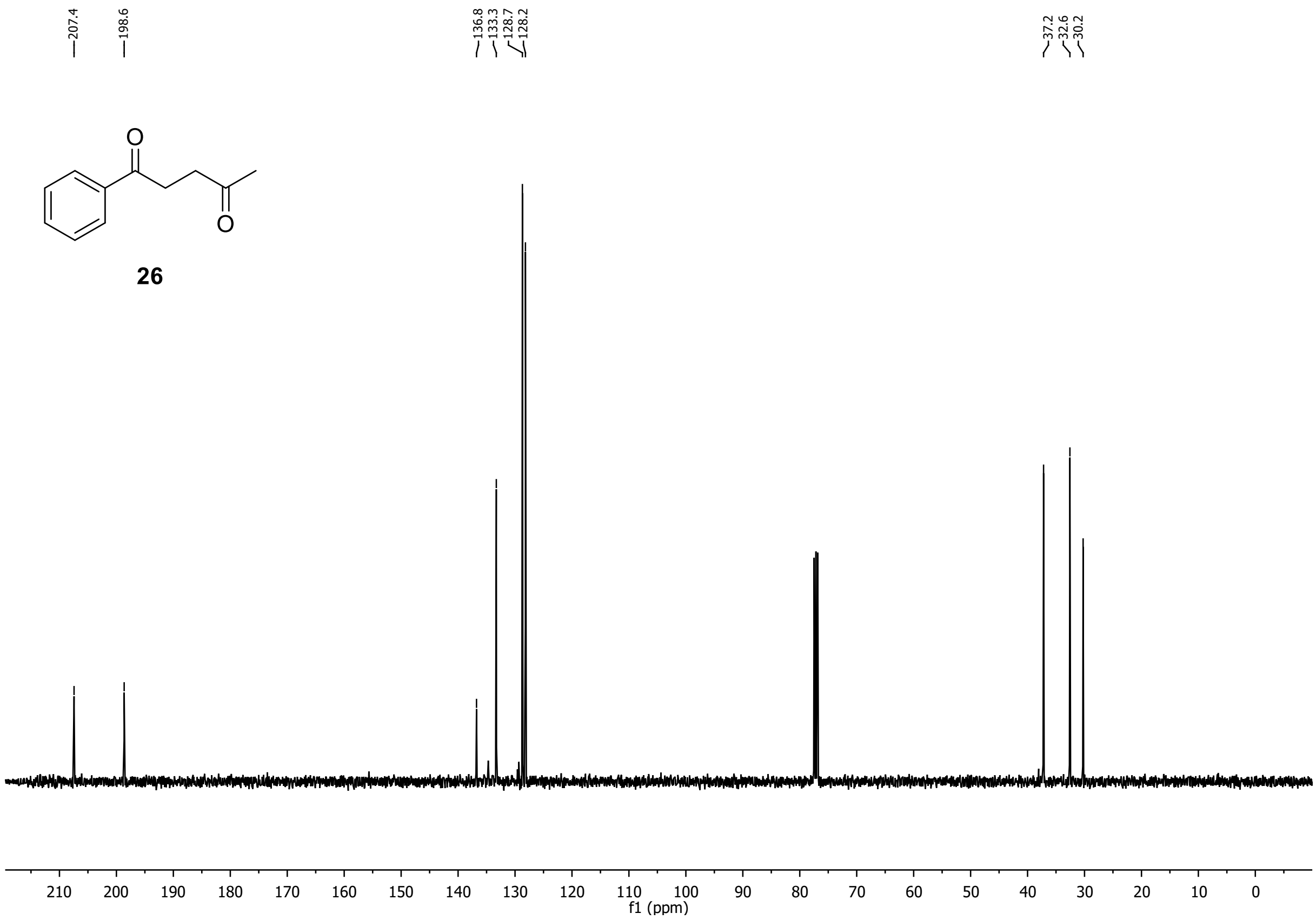

Figure S128. ^{13}C NMR spectrum (101 MHz, $CDCl_3$) of **26**.

***N*,*N*-dimethyl-4-oxo-4-phenylbutanamide (27):**

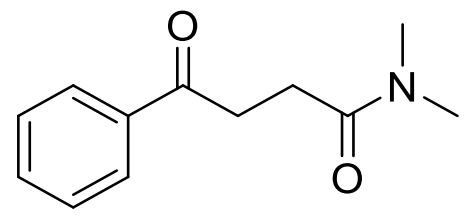

27

$C_{12}H_{15}NO_2$ (205.26 g/mol)

Following **GP-B**, **27** was synthesized using **S1** (214 mg, 1.0 mmol, 1.0 equiv.) and *N*,*N*-dimethyl acrylamide (198 mg, 2.0 mmol, 2 equiv.). Purification by flash column chromatography (*n*-hexane/EtOAc, gradient 60:40 to 0:100 over 10 CV) afforded **27** (85 mg, 414 µmol, 41%) as yellow oil. Conforms to reported analytical data.[30]

R_f: 0.22 (*n*-hexane/EtOAc 40:60).

^{1}H NMR (400 MHz, $CDCl_3$, δ): 8.05 – 7.98 (m, 2H), 7.59 – 7.52 (m, 1H), 7.49 – 7.41 (m, 2H), 3.36 (t, J = 6.6 Hz, 2H), 3.04 (bs, 6H), 2.79 (t, J = 6.6 Hz, 2H).

^{13}C{^{1}H} NMR (101 MHz, $CDCl_3$, δ): 199.4, 171.9, 136.9, 133.1, 128.6, 128.2, 37.2 (bs), 35.7 (bs), 33.8, 27.3.

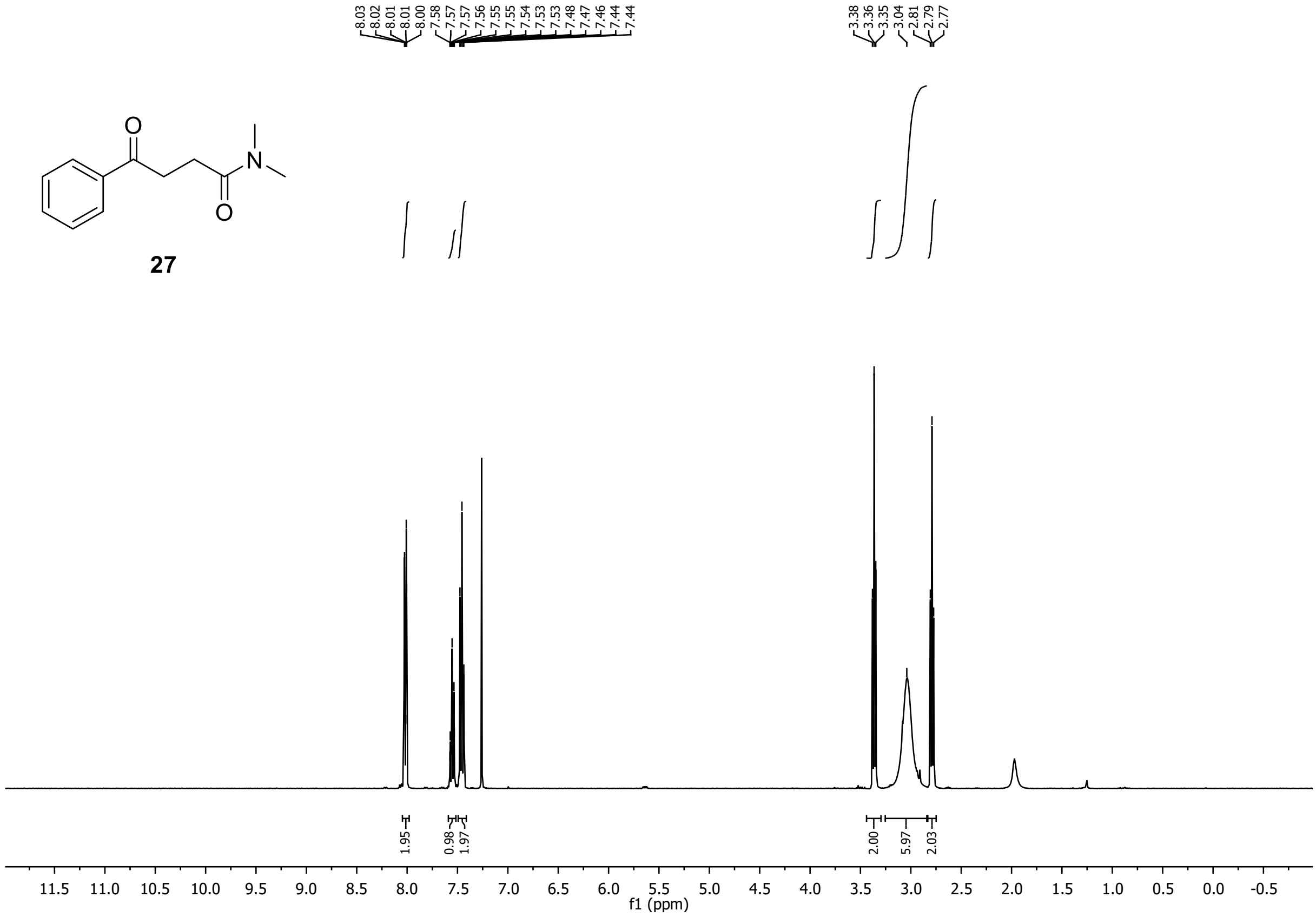

Figure S129. ^{1}H NMR spectrum (400 MHz, $CDCl_3$) of **27**.

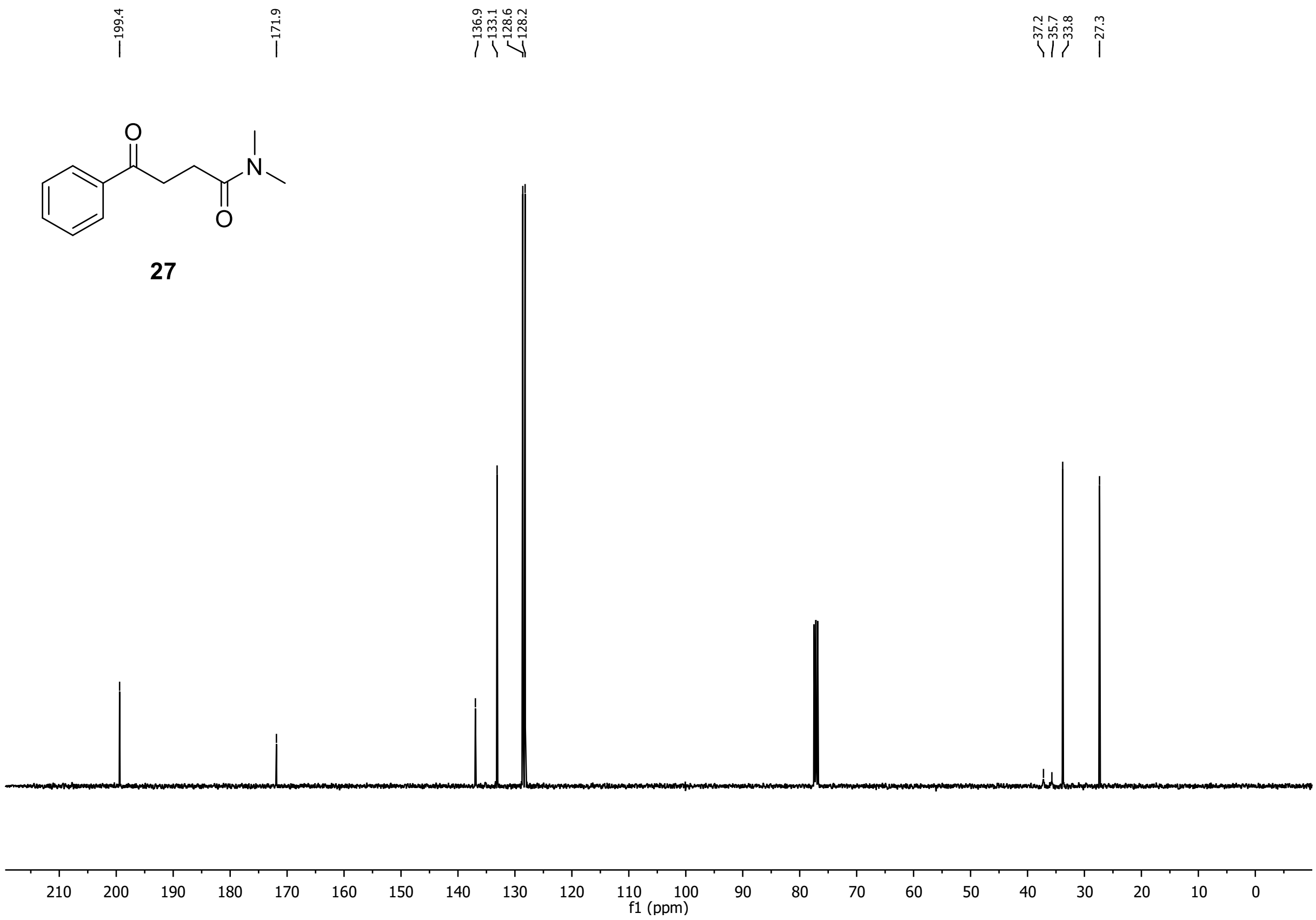

Figure S130. ^{13}C NMR spectrum (101 MHz, $CDCl_3$) of **27**.

4-Oxo-4-phenylbutanenitrile (28):

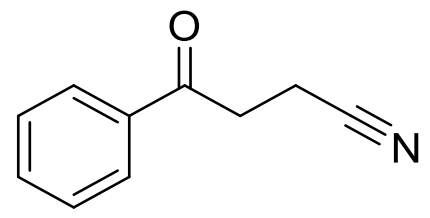

28

$C_{10}H_9NO$ (159.19 g/mol)

Following **GP-B**, **28** was synthesized using **S1** (214 mg, 1.0 mmol, 1.0 equiv.) and acrylonitrile (106 mg, 2.0 mmol, 2 equiv.). Purification by flash column chromatography (*n*-hexane/EtOAc, gradient 100:0 to 50:50 over 10 CV) afforded **28** (125 mg, 785 µmol, 79%) as colorless solid. Conforms to reported analytical data.[31]

$\mathbf{R_f}$: 0.69 (*n*-hexane/EtOAc 60:40).

^{1}H NMR (400 MHz, $CDCl_3$, δ): 8.00 – 7.92 (m, 2H), 7.66 – 7.57 (m, 1H), 7.54 – 7.45 (m, 2H), 3.38 (t, *J* = 7.2 Hz, 2H), 2.78 (t, *J* = 7.2 Hz, 2H).

^{13}C{^{1}H} NMR (101 MHz, $CDCl_3$, δ): 195.4, 135.7, 134.0, 129.0, 128.1, 119.3, 34.4, 11.9.

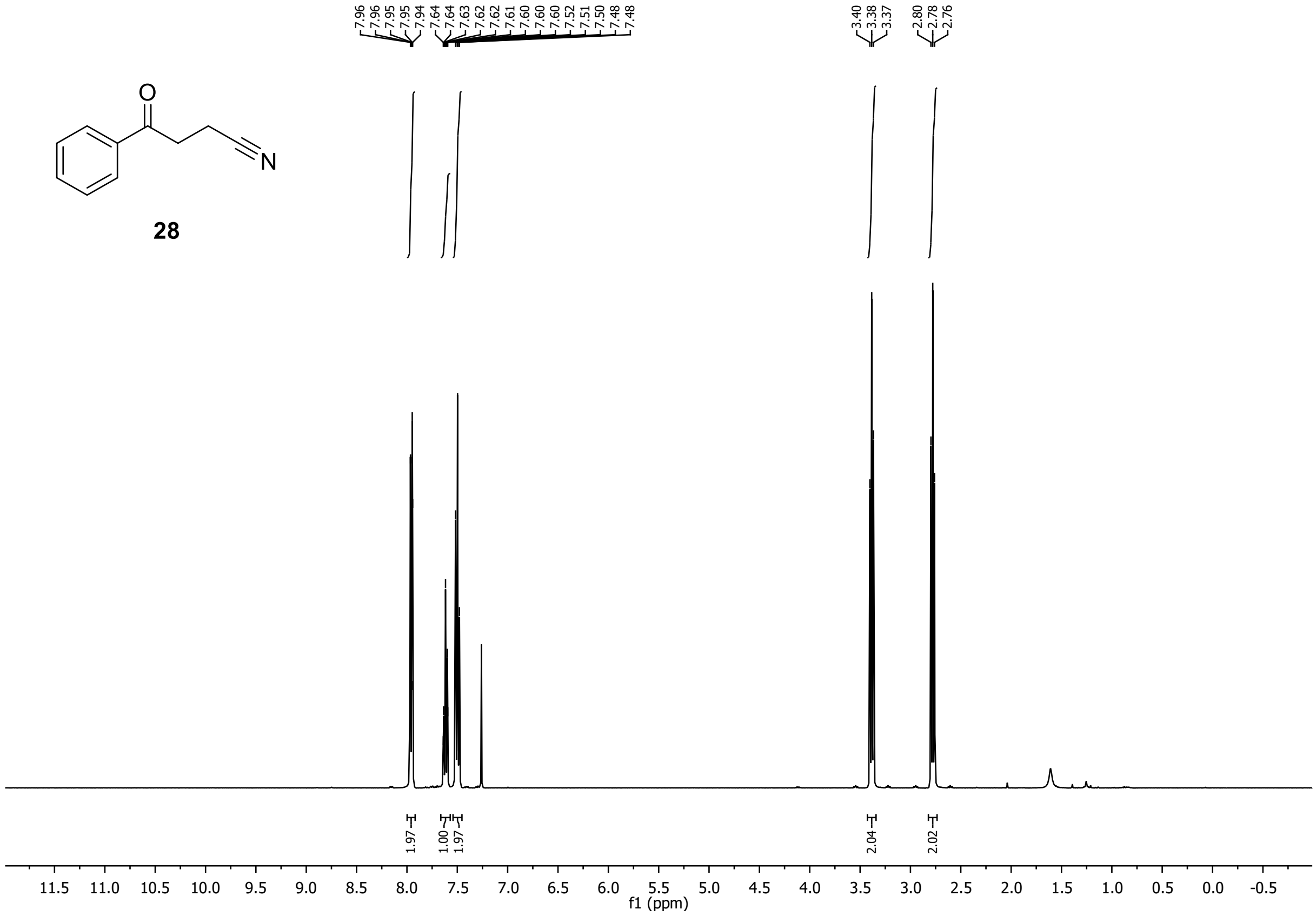

Figure S131. ^{1}H NMR spectrum (400 MHz, $CDCl_3$) of **28**.

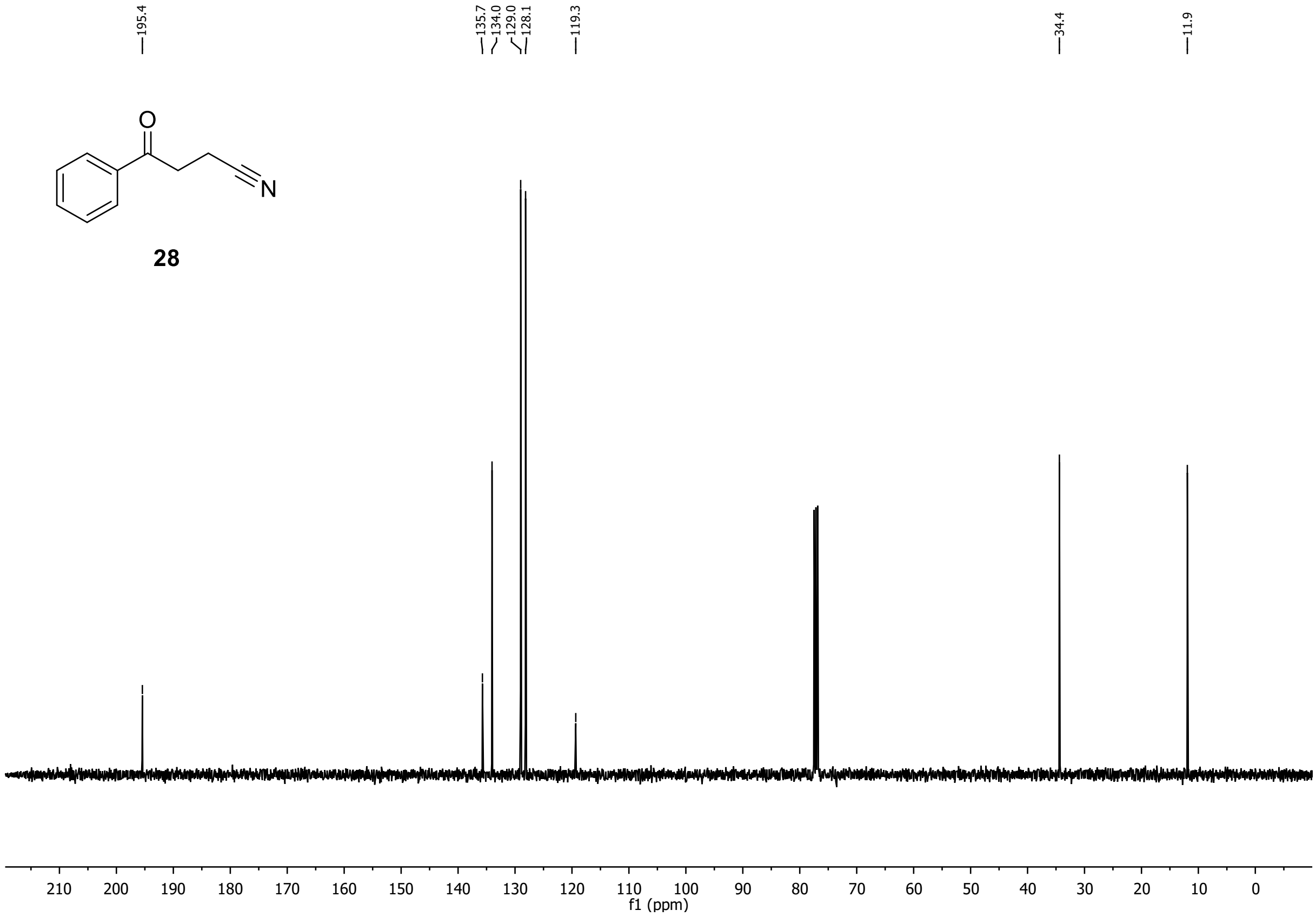

Figure S132. ^{13}C NMR spectrum (101 MHz, $CDCl_3$) of **28**.

5.5 Synthesis of Deuterated Compounds

Ethyl 4-oxo-4-phenylbutanoate-2-*d* (1-D):

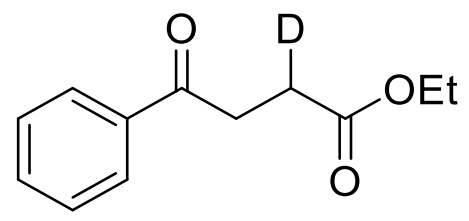

1-D

$C_{12}H_{13}DO_3$ (207.25 g/mol)

Following **GP-C**, **1-D** was synthesized using **S1** (214 mg, 1.0 mmol, 1.0 equiv.) and ethyl acrylate (200 mg, 2.0 mmol, 2 equiv.). Purification by flash column chromatography (*n*-hexane/EtOAc, gradient 100:0 to 70:30 over 10 CV) afforded **1-D** (165 mg, 796 µmol, 80%) as colorless oil. Conforms to reported analytical data.[21]

The deuterium incorporation was determined:

^{1}H NMR: 98% D

A scale-up experiment using **S1** (4.29 g, 20.0 mmol, 1.0 equiv.) and ethyl acrylate (4.0 g, 40.0 mmol, 2.0 equiv.) was performed in a stainless-steel jar (50 mL) using stainless steel milling balls (5 x 3 g). Otherwise, **GP-C** was followed using a solvent mixture of DMF/D_2O (3:1, 4 mL). Purification by column chromatography afforded **1-D** (2.92 g, 14.1 mmol, 71% yield, 99% D) as colorless oil.

R$_f$: 0.55 (*n*-hexane/EtOAc 80:20).

^{1}H NMR (300 MHz, $CDCl_3$, δ): 8.03 – 7.92 (m, 2H), 7.60 – 7.51 (m, 1H), 7.49 – 7.41 (m, 2H), 4.15 (q, *J* = 7.1 Hz, 2H), 3.30 (d, *J* = 6.6 Hz, 2H), 2.80 – 2.66 (m, 1H), 1.25 (t, *J* = 7.1 Hz, 3H).

^{2}H NMR (46 MHz, $CDCl_3$, δ): 2.73.

^{13}C{^{1}H} NMR (75 MHz, $CDCl_3$, δ): 198.2, 173.0, 136.7, 133.3, 128.7, 128.1, 60.7, 33.4, 28.1 (t, *J* = 19.9 Hz), 14.3.

HR-MS (ESI): calc. for $[M+H]^+$ 208.10840, found 208.10793 (err. 2.3 ppm).

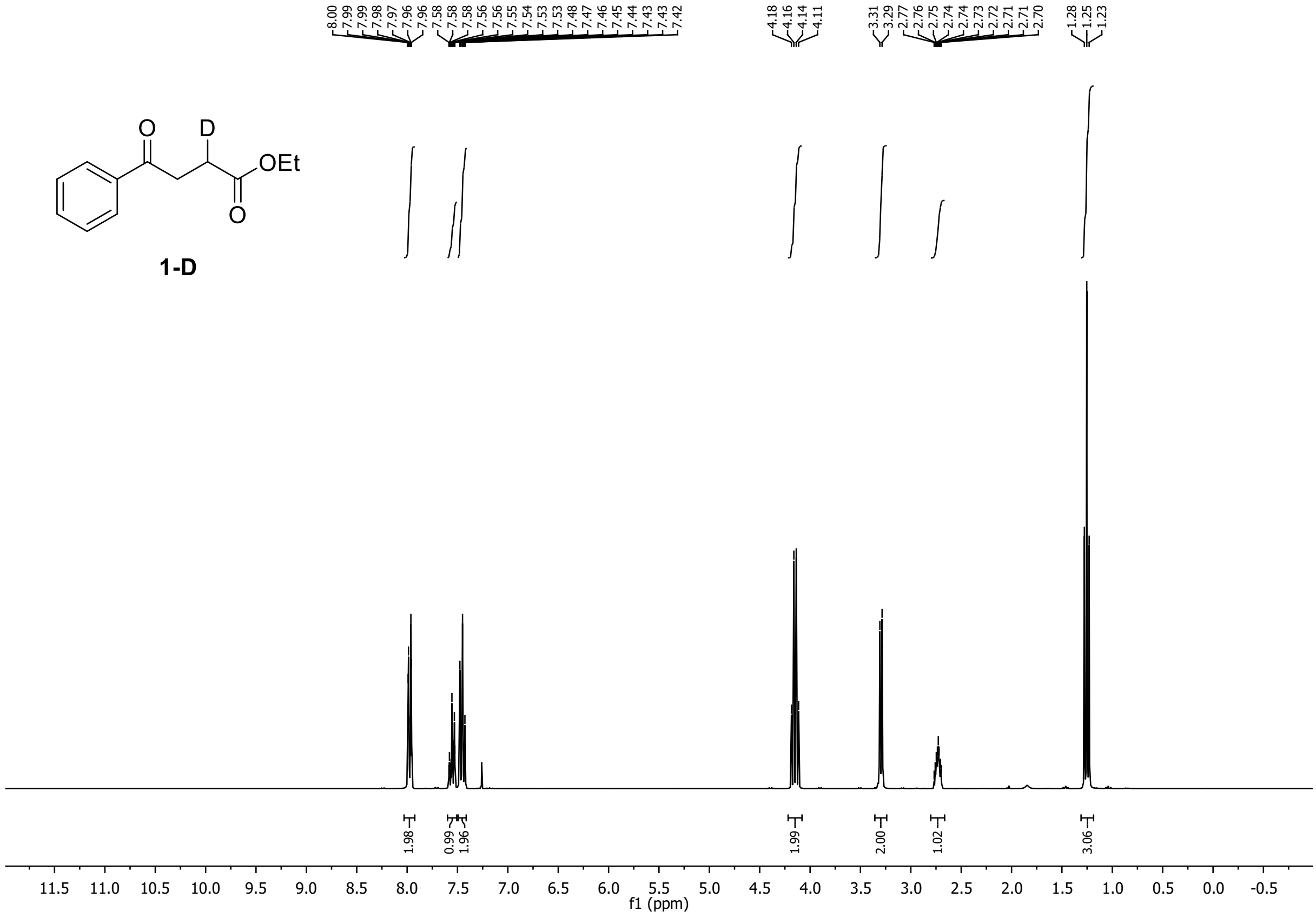

Figure S133. ^{1}H NMR spectrum (300 MHz, $CDCl_3$) of **1-D**.

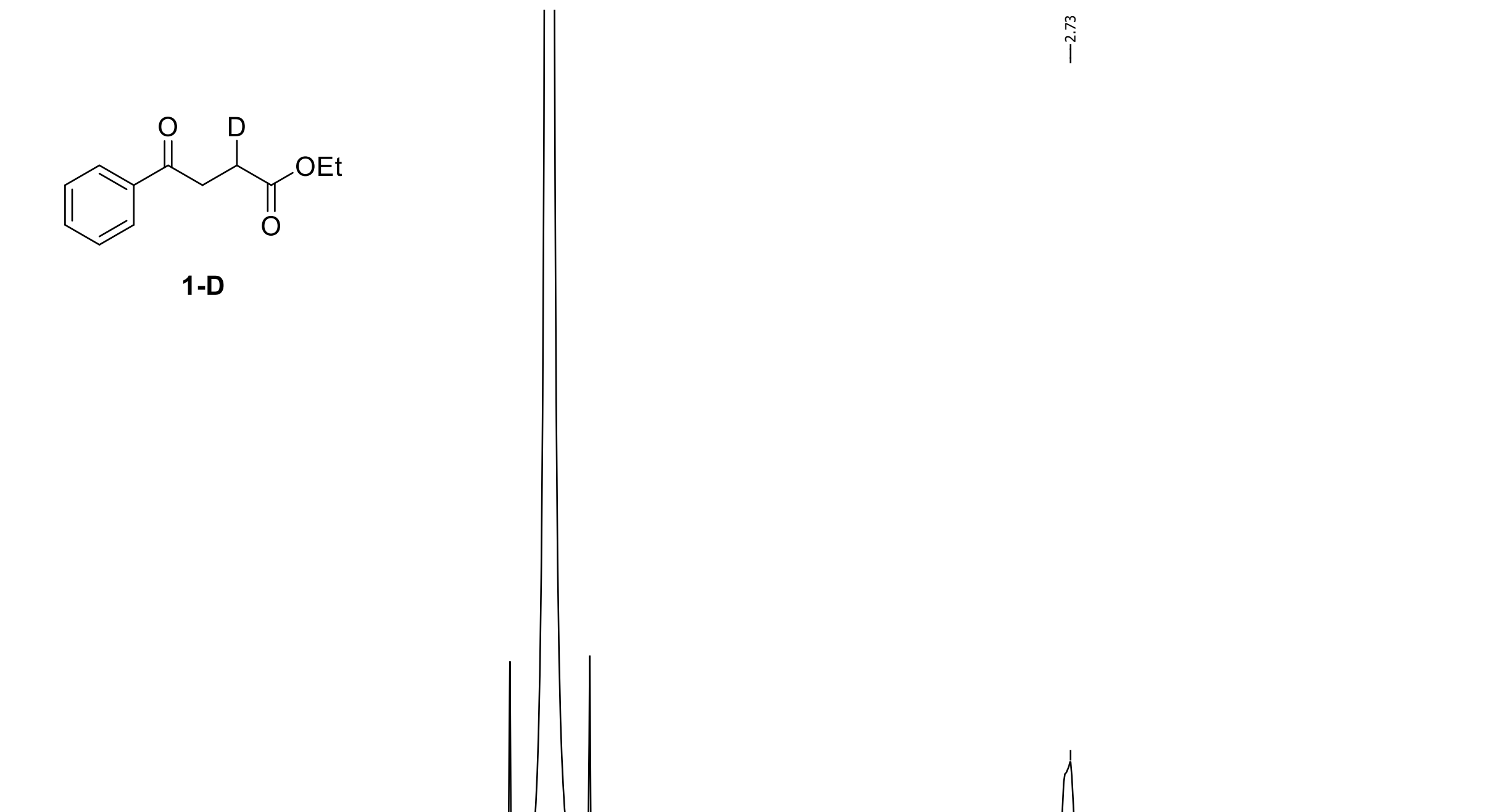

Figure S134. ^{2}H NMR spectrum (46 MHz, $CDCl_3$) of **1-D**.

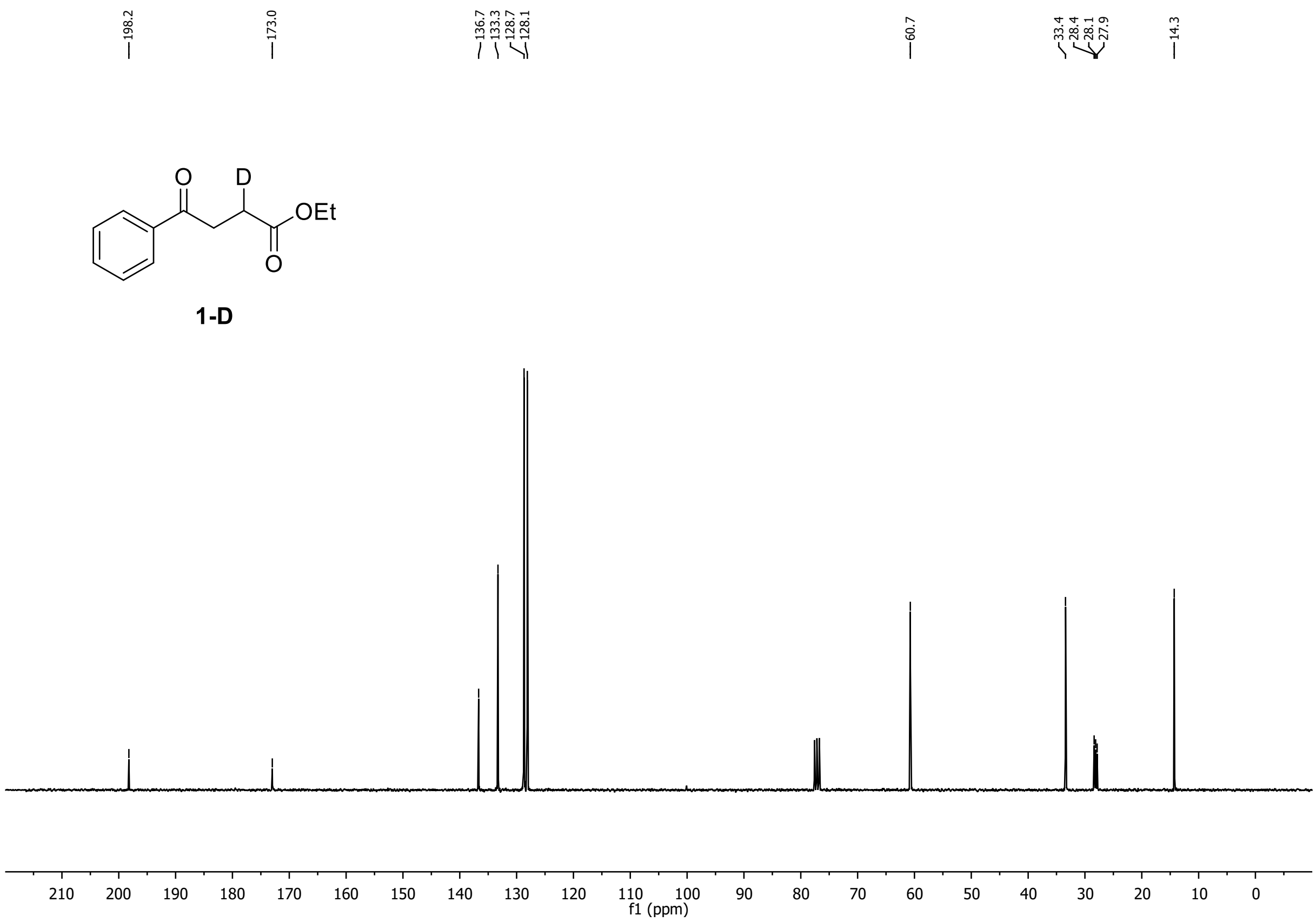

Figure S135. ^{13}C NMR spectrum (75 MHz, $CDCl_3$) of **1-D**.

Ethyl 4-(3-bromophenyl)-4-oxobutanoate-2-*d* (6-D):

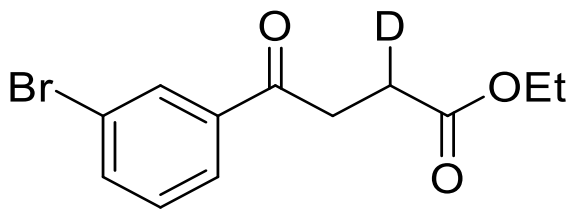

6-D

$C_{12}H_{12}DBrO_3$ (286.14 g/mol)

Following **GP-C**, **6-D** was synthesized using **S6** (293 mg, 1.0 mmol, 1.0 equiv.) and ethyl acrylate (200 mg, 2.0 mmol, 2 equiv.). Purification by flash column chromatography (*n*-hexane/EtOAc, gradient 100:0 to 70:30 over 10 CV) afforded **6-D** (111 mg, 388 µmol, 39%) as colorless oil.

The deuterium incorporation was determined:

^{1}H NMR: 99% D

R$_f$: 0.53 (*n*-hexane/EtOAc 80:20).

^{1}H NMR (300 MHz, $CDCl_3$, δ): 8.10 (dd, *J* = 1.8, 1.8 Hz, 1H), 7.95 – 7.85 (m, 1H), 7.74 – 7.63 (m, 1H), 7.34 (dd, *J* = 7.9, 7.9 Hz, 1H), 4.15 (q, *J* = 7.1 Hz, 2H), 3.26 (d, *J* = 6.5 Hz, 2H), 2.79 – 2.67 (m, 1H), 1.26 (t, *J* = 7.1 Hz, 3H).

^{2}H NMR (46 MHz, $CDCl_3$, δ): 2.74.

^{13}C{^{1}H} NMR (75 MHz, $CDCl_3$, δ): 196.9, 172.8, 138.4, 136.2, 131.3, 130.3, 126.7, 123.1, 60.9, 33.5, 28.0 (t, *J* = 20.0 Hz), 14.3.

HR-MS (ESI): calc. for $[M+H]^+$ 286.01891, found 286.01804 (err. 3.0 ppm).

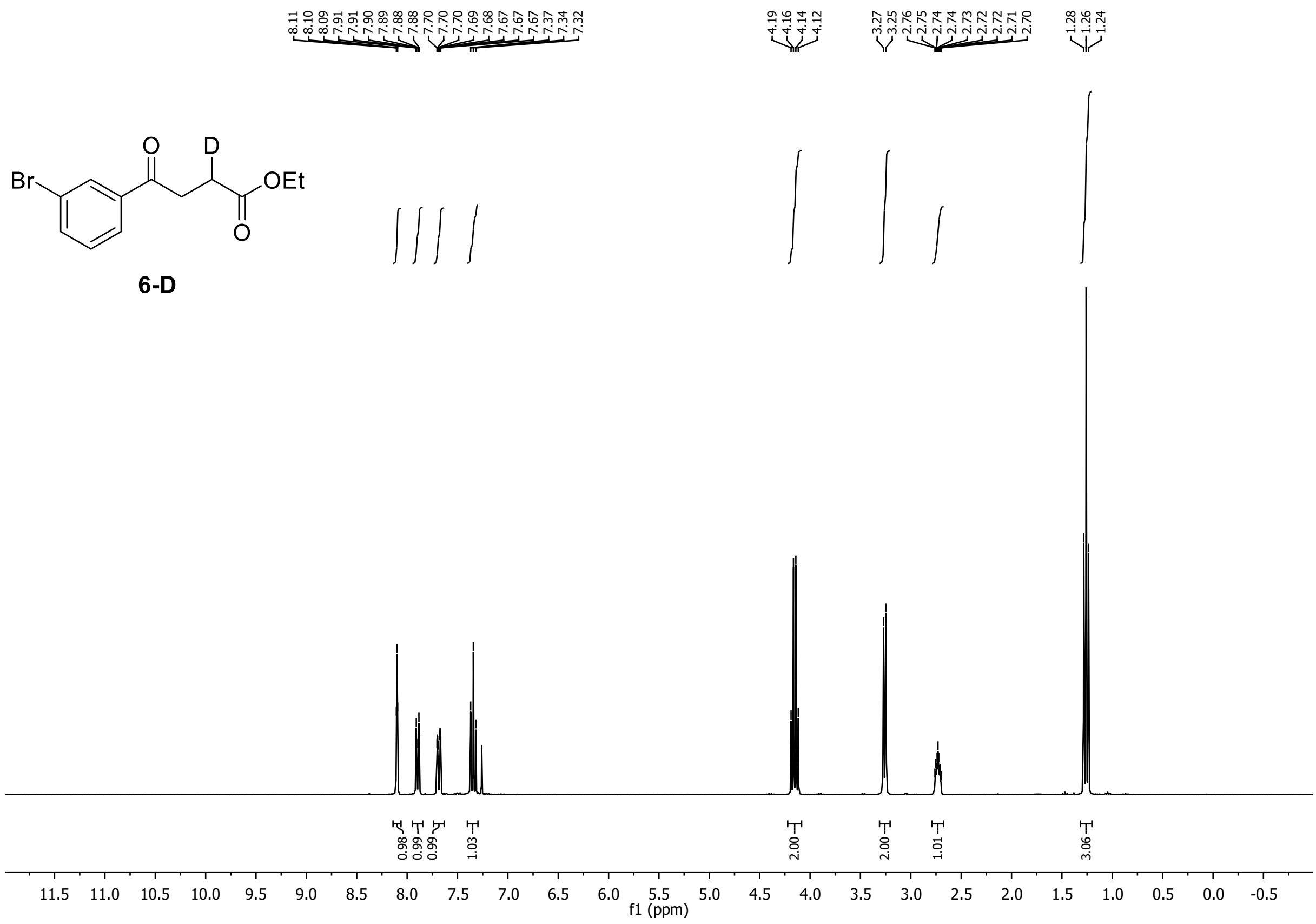

Figure S136. ^{1}H NMR spectrum (300 MHz, $CDCl_3$) of **6-D**.

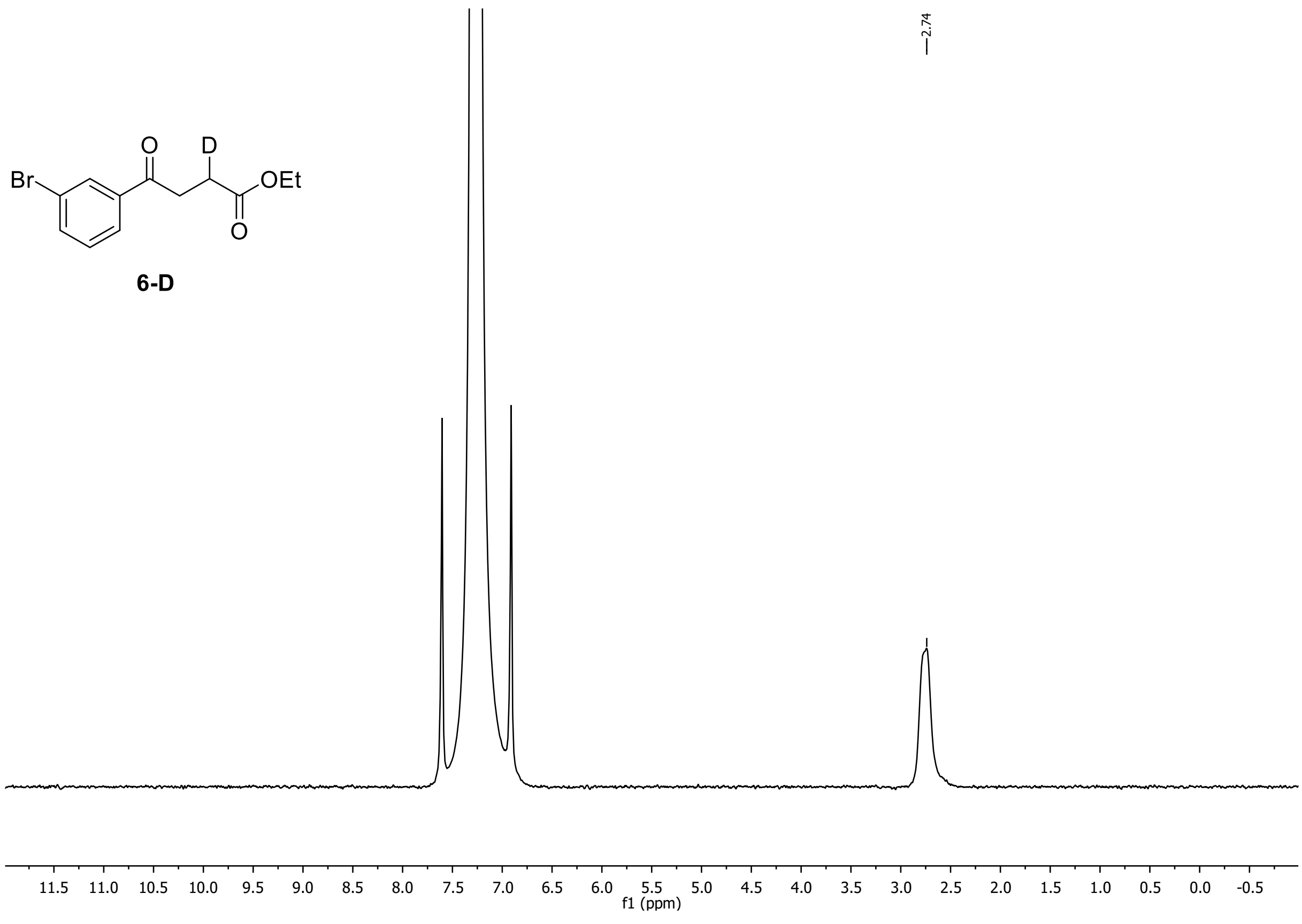

Figure S137. ^{2}H NMR spectrum (46 MHz, $CDCl_3$) of **6-D**.

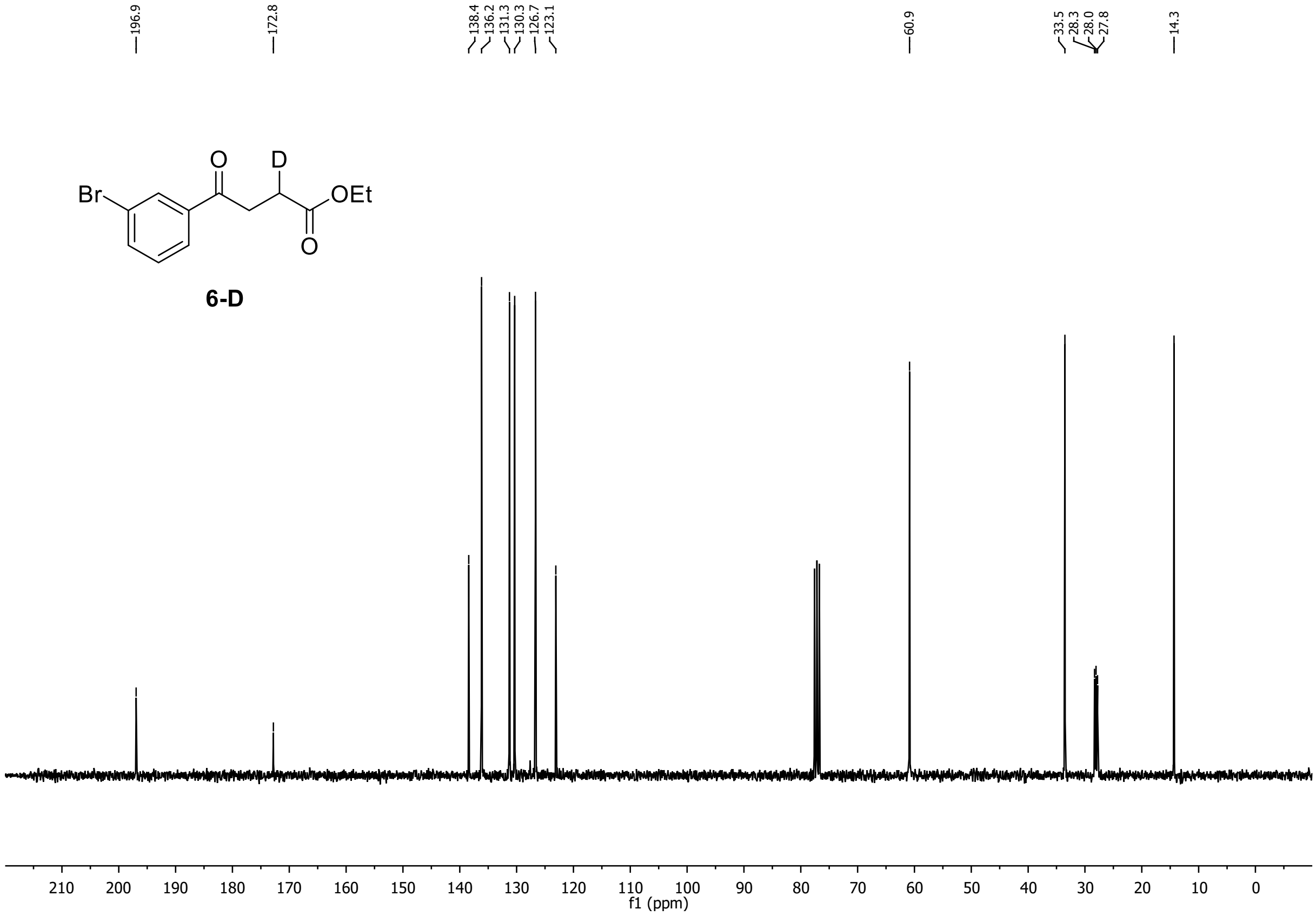

Figure S138. ^{13}C NMR spectrum (75 MHz, $CDCl_3$) of **6-D**.

Ethyl 4-(benzo[*d*][1,3]dioxol-5-yl)-4-oxobutanoate-2-*d* (9-D):

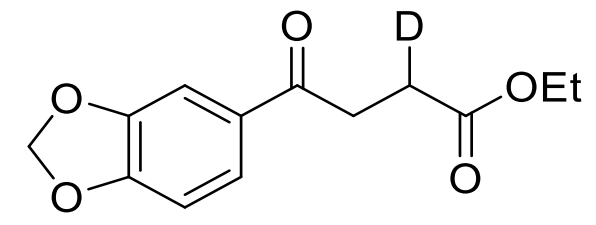

9-D

$C_{13}H_{13}DO_5$ (251.26 g/mol)

Following **GP-C**, **9-D** was synthesized using **S9** (258 mg, 1.0 mmol, 1.0 equiv.) and ethyl acrylate (200 mg, 2.0 mmol, 2 equiv.). Purification by flash column chromatography (*n*-hexane/EtOAc, gradient 100:0 to 30:70 over 10 CV) afforded **9-D** (210 mg, 836 µmol, 84%) as colorless oil.

The deuterium incorporation was determined:

^{1}H NMR: 99% D

R$_f$: 0.77 (*n*-hexane/EtOAc 60:40).

^{1}H NMR (300 MHz, $CDCl_3$, δ): 7.58 (dd, *J* = 8.1, 1.7 Hz, 1H), 7.44 (d, *J* = 1.7 Hz, 1H), 6.84 (d, *J* = 8.1 Hz, 1H), 6.03 (s, 2H), 4.15 (q, *J* = 7.1 Hz, 2H), 3.22 (d, *J* = 6.6 Hz, 2H), 2.76 – 2.65 (m, 1H), 1.25 (t, *J* = 7.1 Hz, 3H).

^{2}H NMR (46 MHz, $CDCl_3$, δ): 2.71.

^{13}C{^{1}H} NMR (75 MHz, $CDCl_3$, δ): 196.3, 173.1, 151.9, 148.3, 131.6, 124.4, 108.00, 107.96, 102.0, 60.7, 33.2, 28.3 (t, J = 19.6 Hz), 14.3.

HR-MS (ESI): calc. for $[M+H]^+$ 252.09823, found 252.09750 (err. 2.9 ppm).

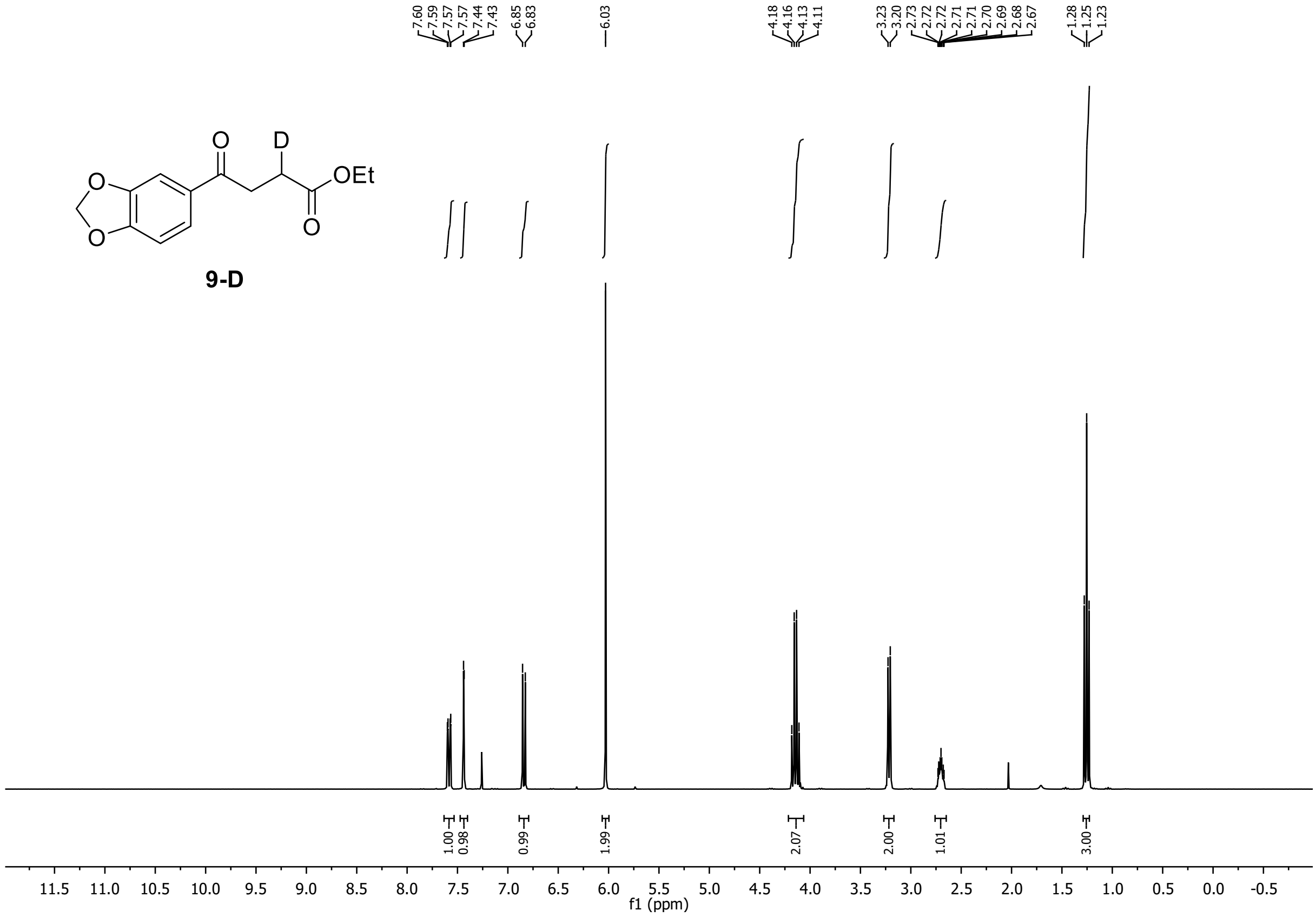

Figure S139. ^{1}H NMR spectrum (300 MHz, $CDCl_3$) of **9-D**.

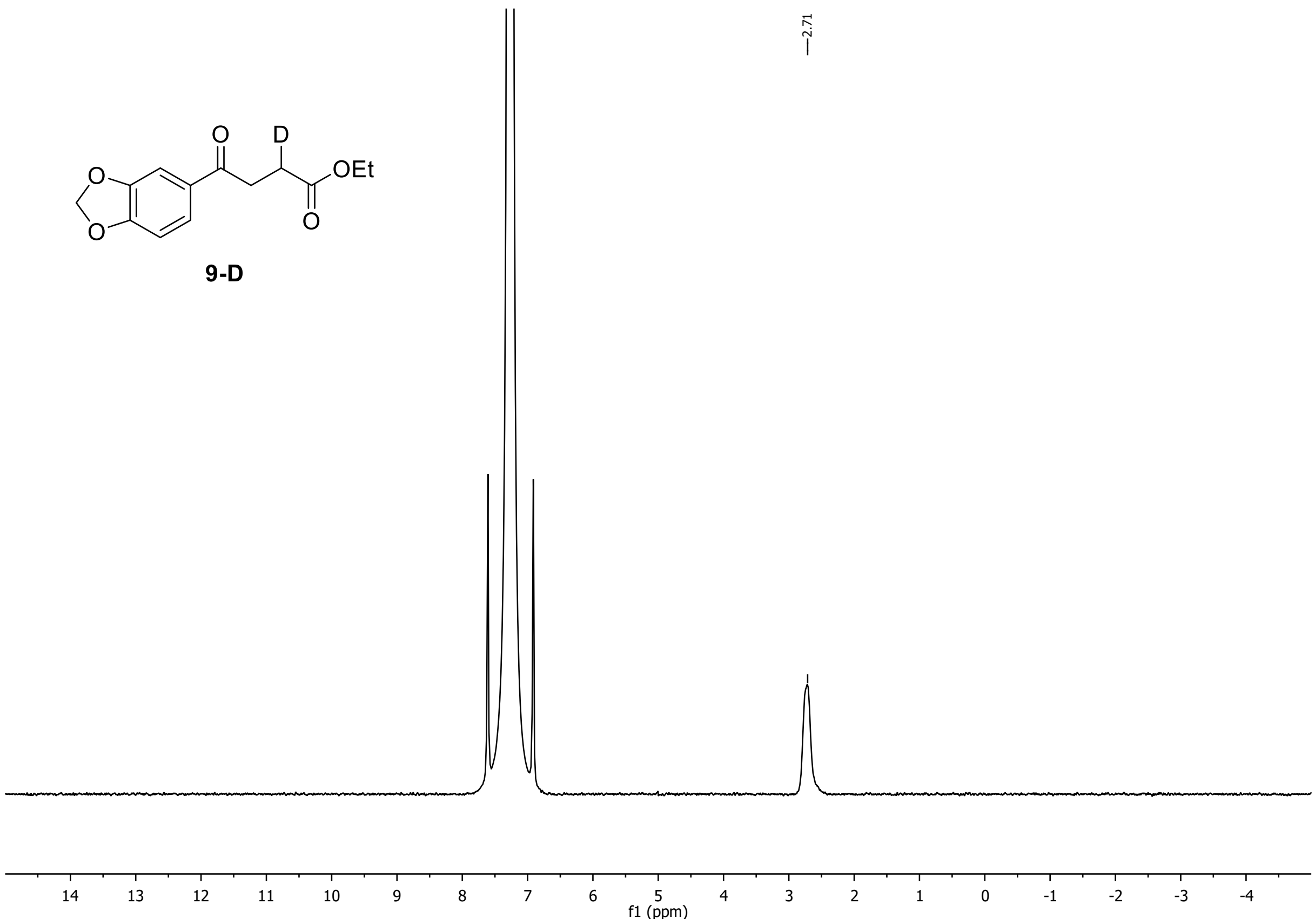

Figure S140. ^{2}H NMR spectrum (46 MHz, $CDCl_3$) of **9-D**.

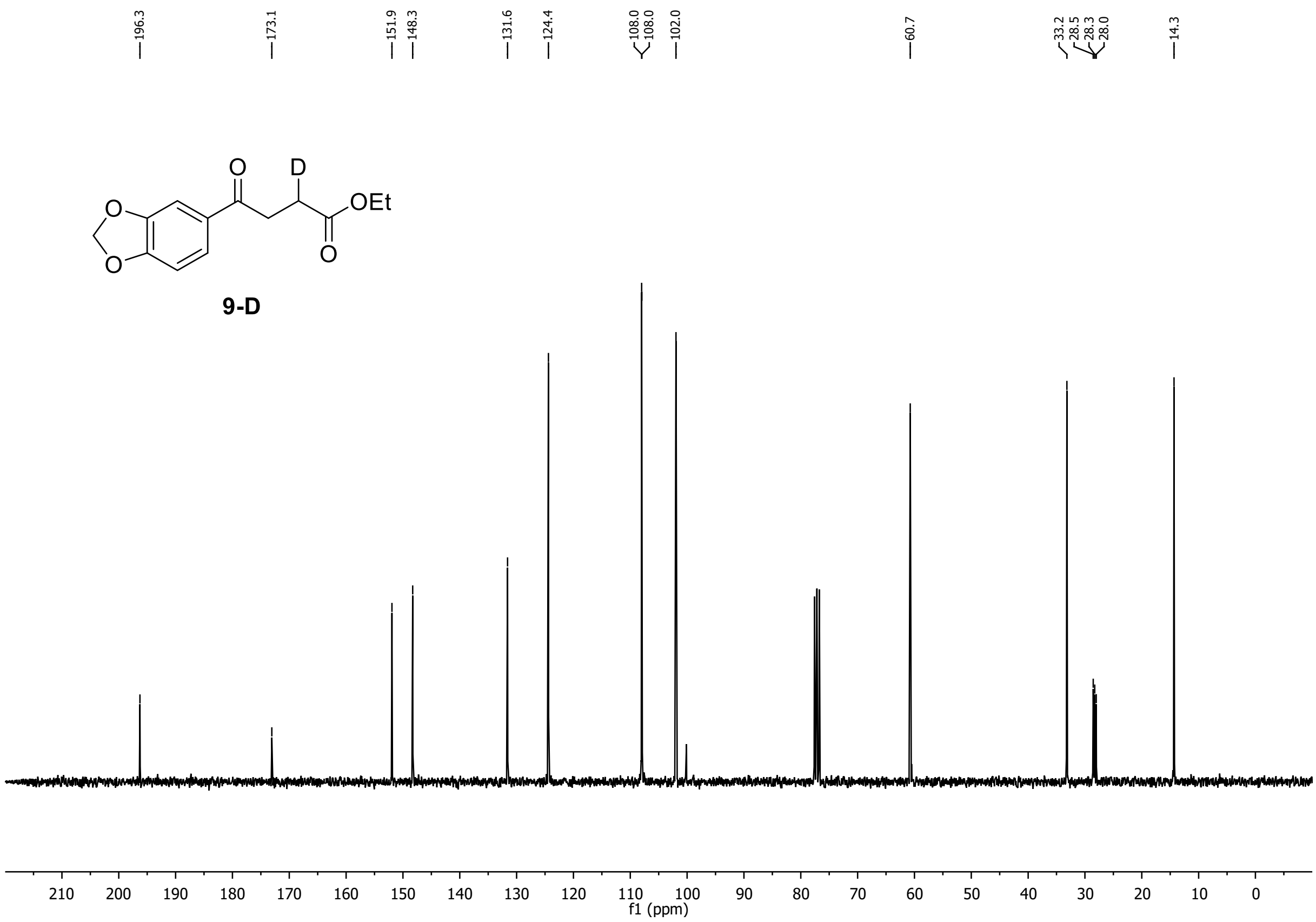

Figure S141. ^{13}C NMR spectrum (75 MHz, $CDCl_3$) of **9-D**.

4-Oxo-4-phenylbutanenitrile-2-*d* (28-D):

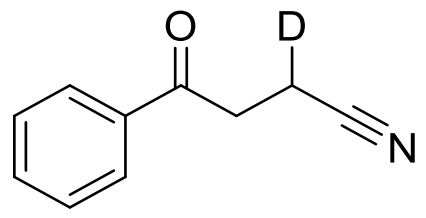

28-D

$C_{10}H_8DNO$ (160.19 g/mol)

Following **GP-4**, **28-D** was synthesized using **S1** (214 mg, 1.0 mmol, 1.0 equiv.) and acrylonitrile (106 mg, 2.0 mmol, 2 equiv.). Purification by flash column chromatography (*n*-hexane/EtOAc, gradient 100:0 to 50:50 over 10 CV) afforded **28-D** (128 mg, 799 µmol, 80%) as colorless solid.

The deuterium incorporation was determined: > 99% D (^{1}H NMR).

R$_f$: 0.69 (*n*-hexane/EtOAc 60:40).

^{1}H NMR (300 MHz, $CDCl_3$, δ): 8.02 – 7.91 (m, 2H), 7.67 – 7.57 (m, 1H), 7.56 – 7.45 (m, 2H), 3.43 – 3.34 (m, 2H), 2.84 – 2.72 (m, 1H).

^{2}H NMR (46 MHz, $CDCl_3$, δ): 2.73.

^{13}C{^{1}H} NMR (75 MHz, $CDCl_3$, δ): 195.4, 137.1, 135.7, 134.1, 129.0, 128.2, 34.3, 11.8 (t, J = 20.8 Hz).

HR-MS (APCI): m/z calc for $[M+H]^+$ 161.08197, found 161.08165 (err. 1.99 ppm).

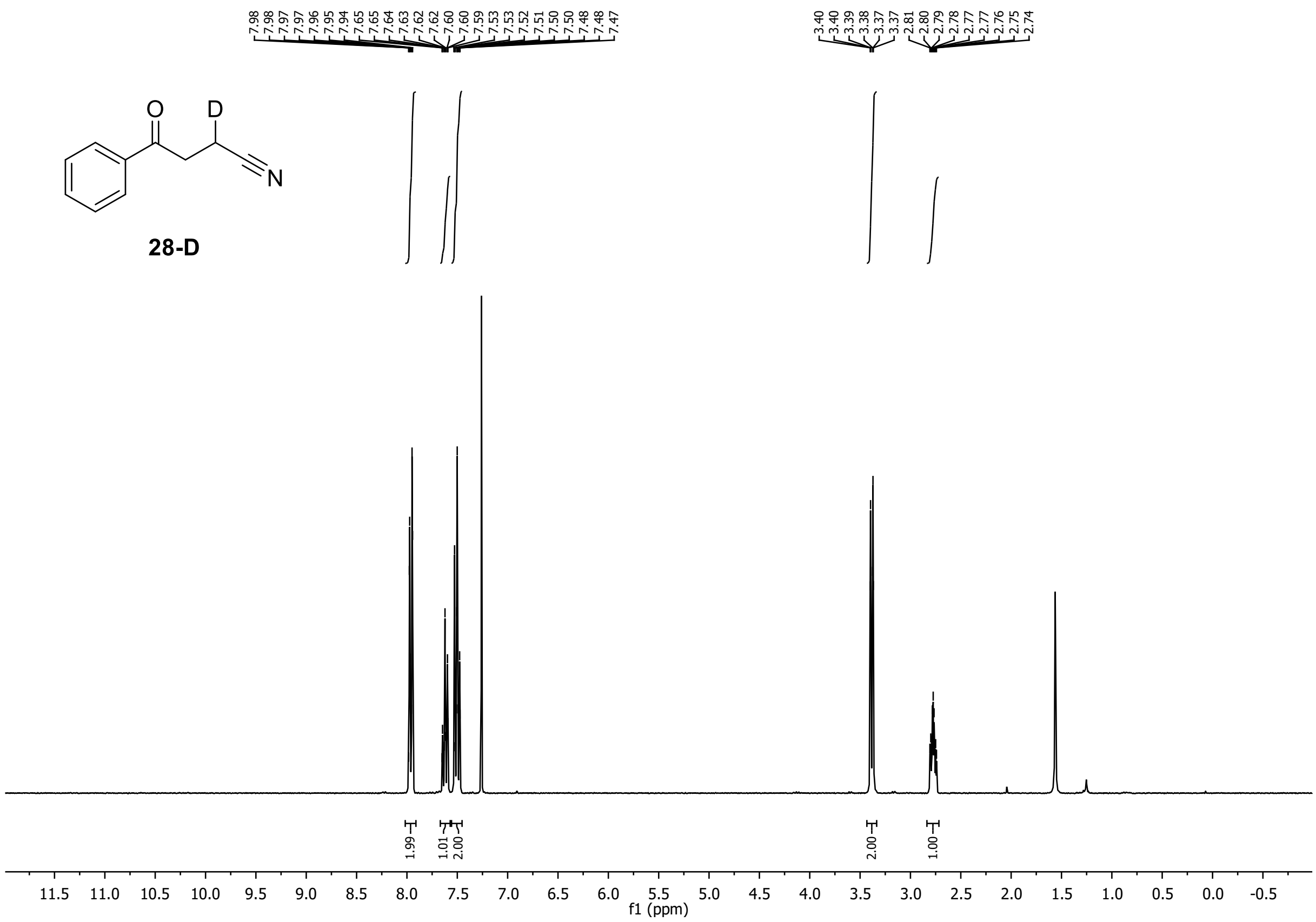

Figure S142. ^{1}H NMR (300 MHz, $CDCl_3$) spectrum of **28-D**.

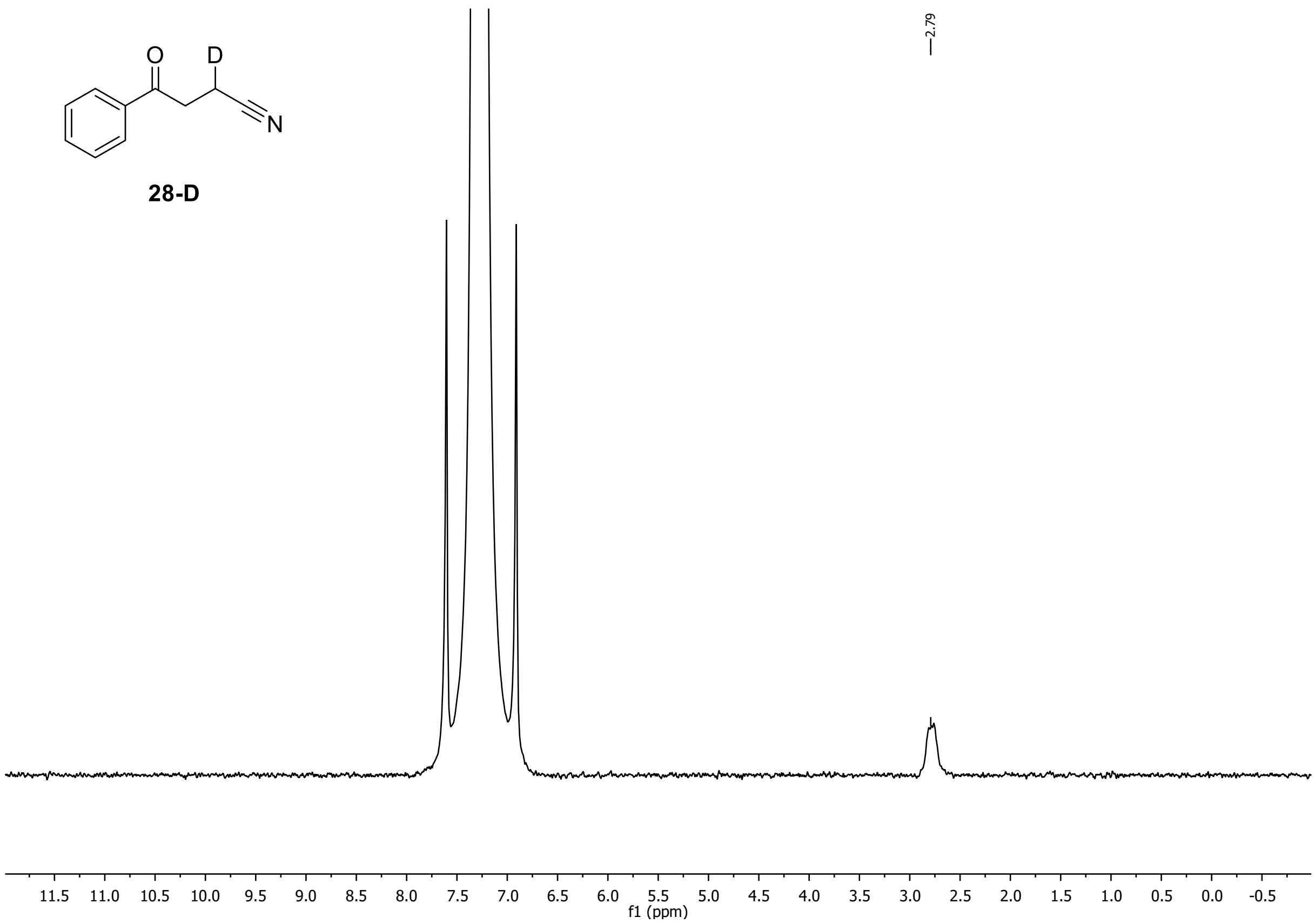

Figure S143. ^{2}H NMR (46 MHz, $CDCl_3$) spectrum of **28-D**.

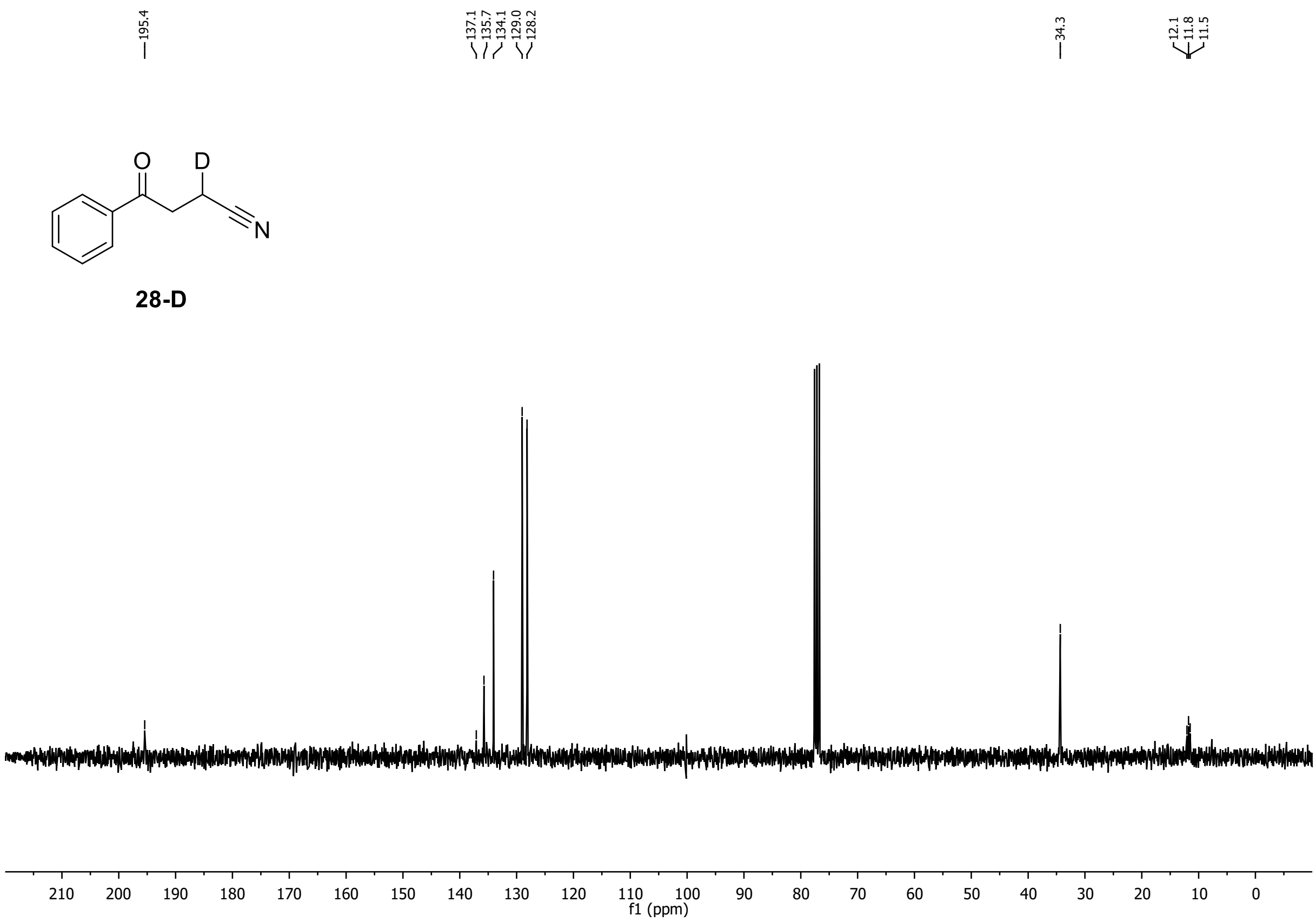

Figure S144. ^{13}C NMR (75 MHz, $CDCl_3$) spectrum of **28-D**.

(8*R*,9*S*,13*S*,14*S*)-13-Methyl-17-oxo-7,8,9,11,12,13,14,15,16,17-decahydro-6*H*-cyclopenta[*a*]phenanthren-3-yl 4-oxo-4-phenylbutanoate-2-*d* (29-D):

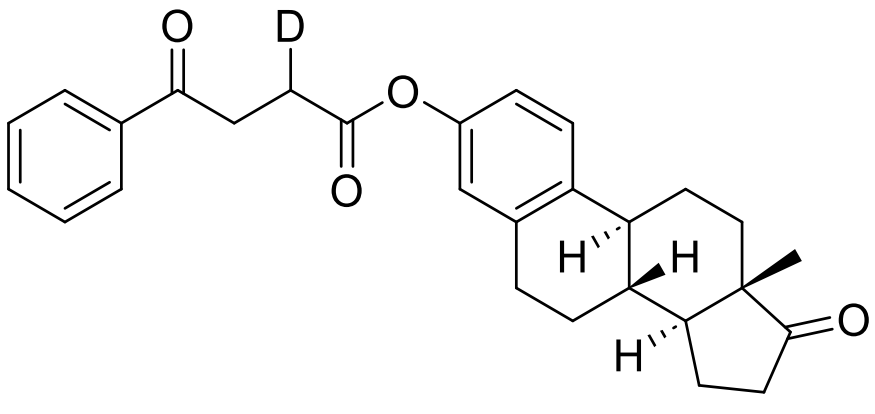

29-D

$C_{28}H_{29}DO_4$ (431.55 g/mol)

Following **GP-4**, **29-D** was synthesized using **S1** (214 mg, 1.0 mmol, 1.0 equiv.) and **S29** (649 mg, 2.0 mmol, 2 equiv.). Purification by flash column chromatography (*n*-hexane/EtOAc, gradient 70:30 to 0:100 over 10 CV) afforded **29-D** (262 mg, 607 µmol, 61%) as colorless solid.

The deuterium incorporation was determined: 92% D (^{1}H NMR).

R$_f$: 0.32 (*n*-hexane/EtOAc 70:30).

m.p.: 165.4 °C.

^{1}H NMR (300 MHz, $CDCl_3$, δ): δ 8.06 – 7.96 (m, 2H), 7.63 – 7.53 (m, 1H), 7.54 – 7.43 (m, 2H), 7.30 – 7.26 (m, 1H), 6.93 – 6.81 (m, 2H), 3.42 (d, *J* = 6.5 Hz, 2H), 3.04 – 2.95 (m, 1H), 2.94 – 2.87 (m, 2H), 2.56 – 2.46 (m, 1H), 2.44 – 2.37 (m, 1H), 2.34 – 2.24 (m, 1H), 2.21 – 2.10 (m, 1H), 2.04 – 1.94 (m, 2H), 1.64 – 1.42 (m, 7H), 0.91 (s, 3H).

^{2}H NMR (46 MHz, $CDCl_3$, δ): 2.99.

^{13}C{^{1}H} NMR (101 MHz, $CDCl_3$, δ): 221.0, 198.0, 172.0, 148.8, 138.1, 137.5, 136.6, 133.5, 128.8, 128.2, 126.5, 121.7, 118.9, 50.6, 48.1, 44.3, 38.1, 36.0, 33.5, 31.7, 29.5, 26.5, 25.9, 21.7, 14.0.

HR-MS (APCI): m/z calc for $[M+H]^+$ 432.22796, found 432.22711 (err. 1.97 ppm).

IR (ATR, $\tilde{\nu}$ [cm^{-1}]): 2928 (w), 2864 (w), 2819 (w), 1728 (s), 1683 (m), 1588 (w), 1493 (w), 1448 (w), 1414 (w), 1370 (w), 1280 (m), 1219 (s), 1164 (s), 1109 (w), 1086 (w), 1053 (w), 1008 (m), 960 (w), 906 (w), 844 (w), 818 (w), 777 (w), 729 (m), 692 (m).

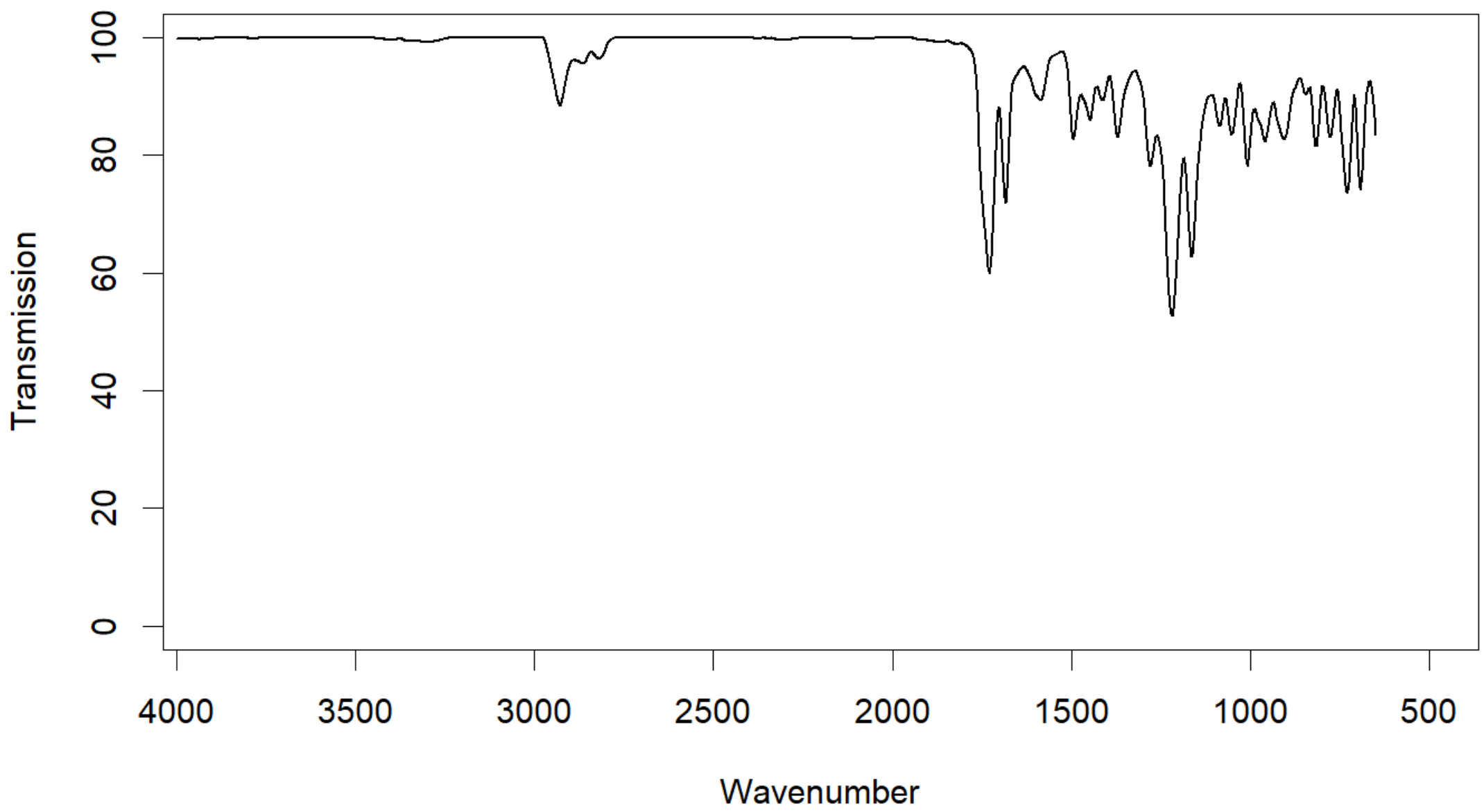

Figure S145. IR spectrum (ATR, neat) of **29-D**.

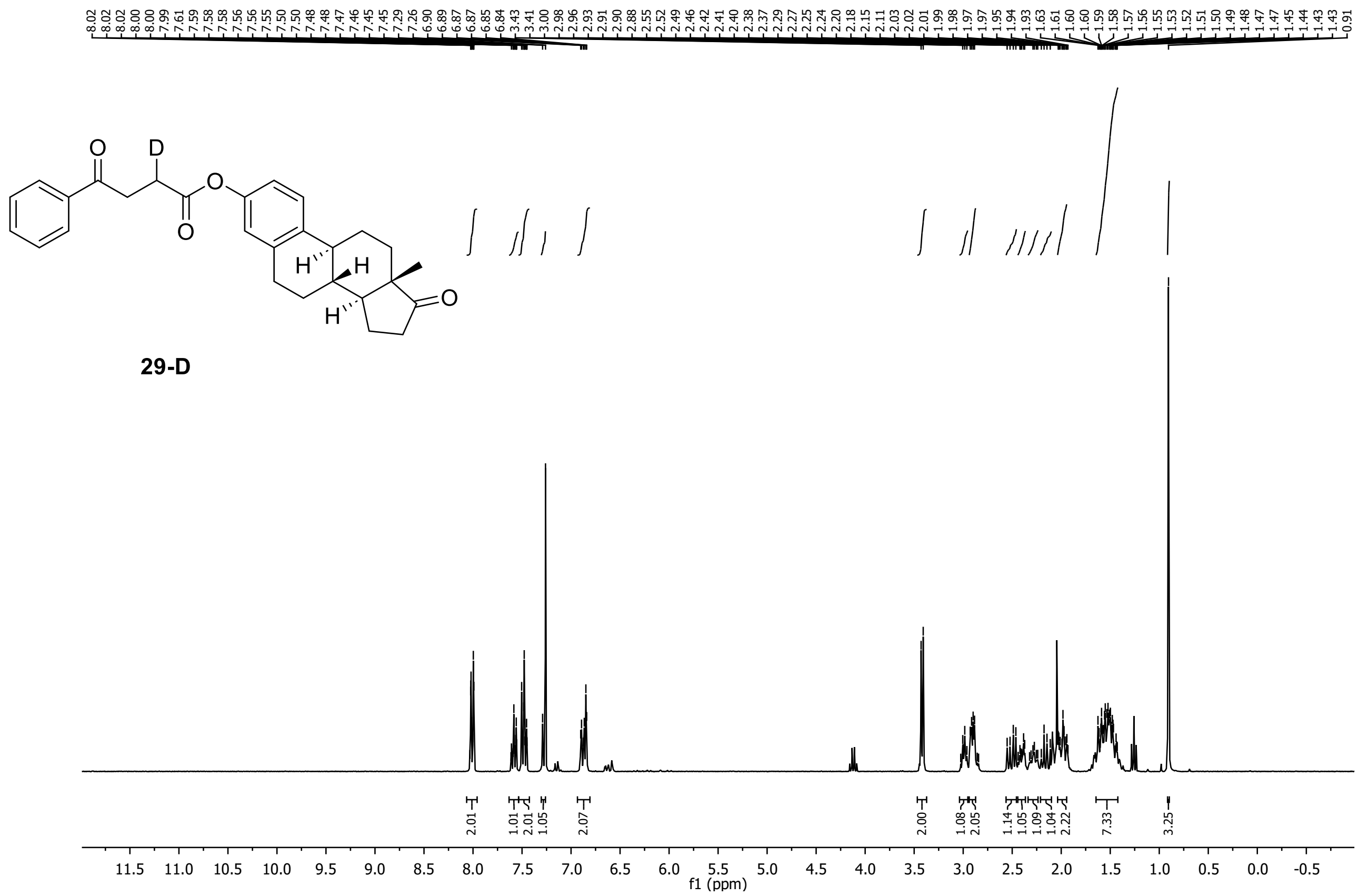

Figure S146. ^{1}H NMR (300 MHz, $CDCl_3$) spectrum of **29-D**.

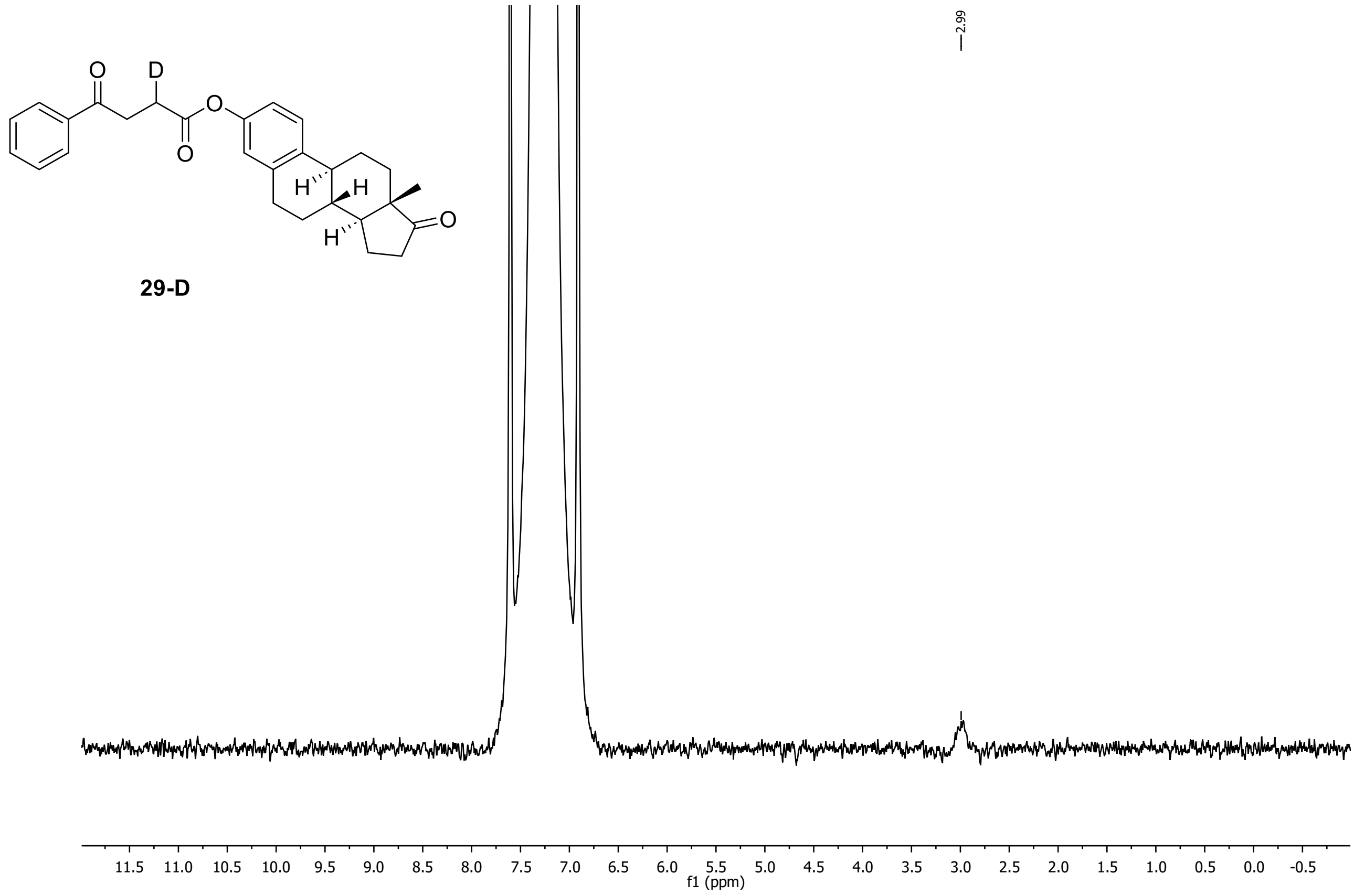

Figure S147. ^{2}H NMR (46 MHz, $CDCl_3$) spectrum of **29-D**.

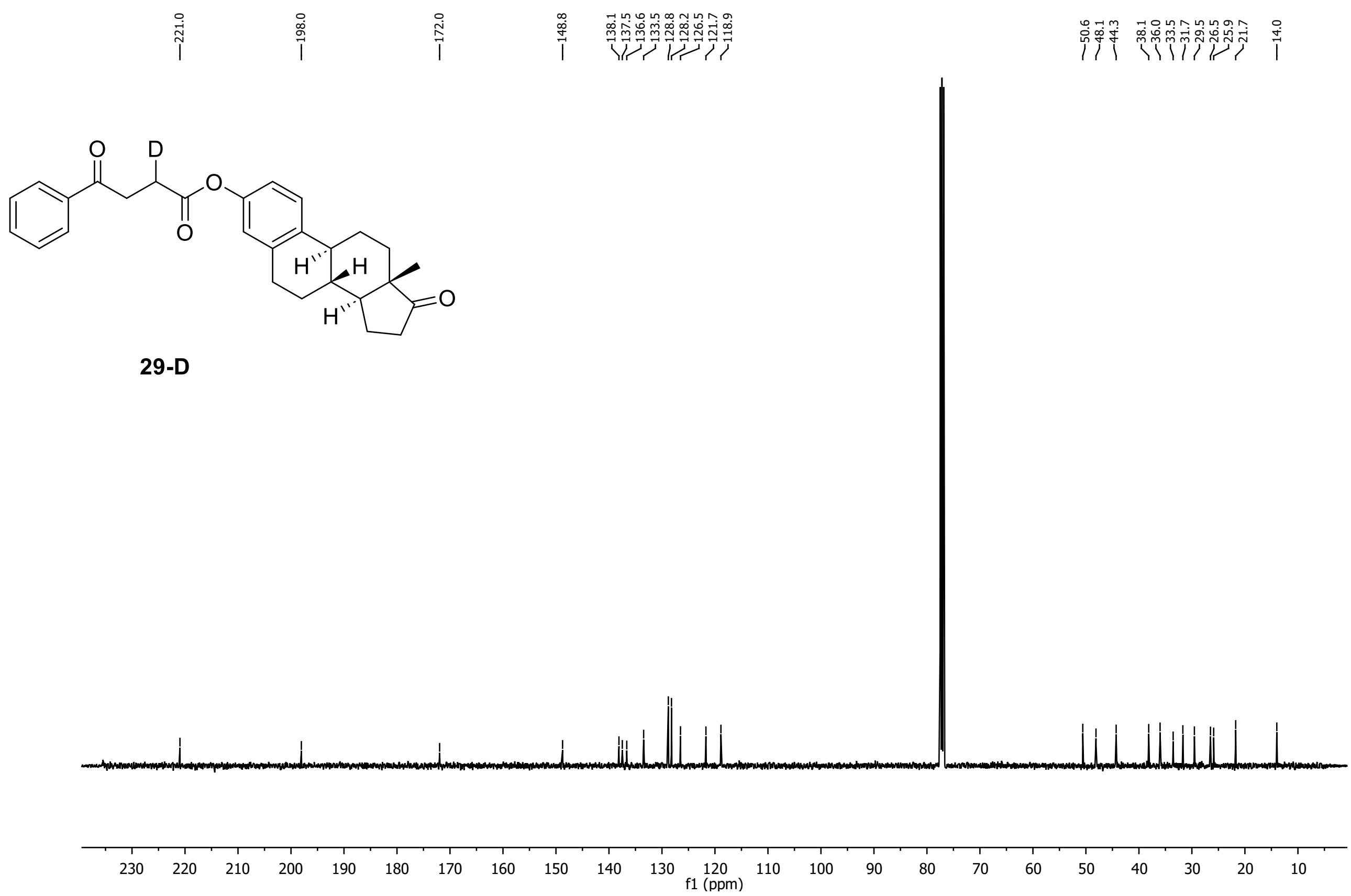

Figure S148. ^{13}C NMR (101 MHz, $CDCl_3$) spectrum of **29-D**.

Methyl-d_3 4-oxo-4-phenylbutanoate-2,2-d_2 (30-D_5):

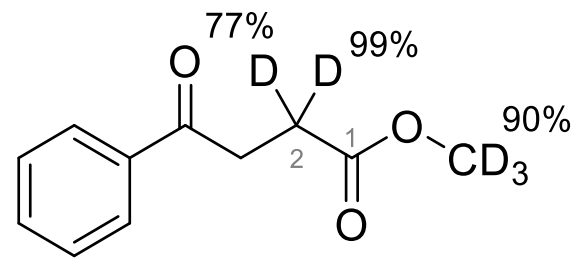

30-D_5

$C_{11}H_7D_5O_3$ (197.24 g/mol)

Following **GP-C**, **30-D_5** was synthesized using **S1** (293 mg, 1.0 mmol, 1.0 equiv.) and methyl acrylate-d_4 (**S30a**) (180 mg, 2.0 mmol, 2 equiv., 77% D at 2-position). Purification by flash column chromatography (*n*-hexane/EtOAc, gradient 100:0 to 70:30 over 10 CV) afforded **30-D_5** (160 mg, 811 µmol, 81%) as colorless oil.

The deuterium incorporation was determined:

^{1}H NMR: 84% D in position 2.

Assuming that the D-incorporation of the starting material did not change during the reaction, 99% D was incorporated during the deuteroacylation reaction.

R_f: 0.47 (*n*-hexane/EtOAc 80:20).

^{1}H NMR (300 MHz, $CDCl_3$, δ): 8.02 – 7.93 (m, 2H), 7.61 – 7.51 (m, 1H), 7.50 – 7.41 (m, 2H), 3.70 (s, 0.29H), 3.36 – 3.25 (m, 2H), 2.80 – 2.69 (m, 0.33H).

^{2}H NMR (46 MHz, $CDCl_3$, δ): 3.68, 2.75.

$^{13}C\{^1H\}$ NMR (75 MHz, $CDCl_3$, δ): 198.1, 173.5, 136.6, 133.3, 128.7, 128.1, 51.9, 33.43, 33.36, 27.9 (t, J = 19.9 Hz).

HR-MS (ESI): calc. for $[M+H]^+$ 198.11785, found 198.11714 (err. 3.6 ppm).

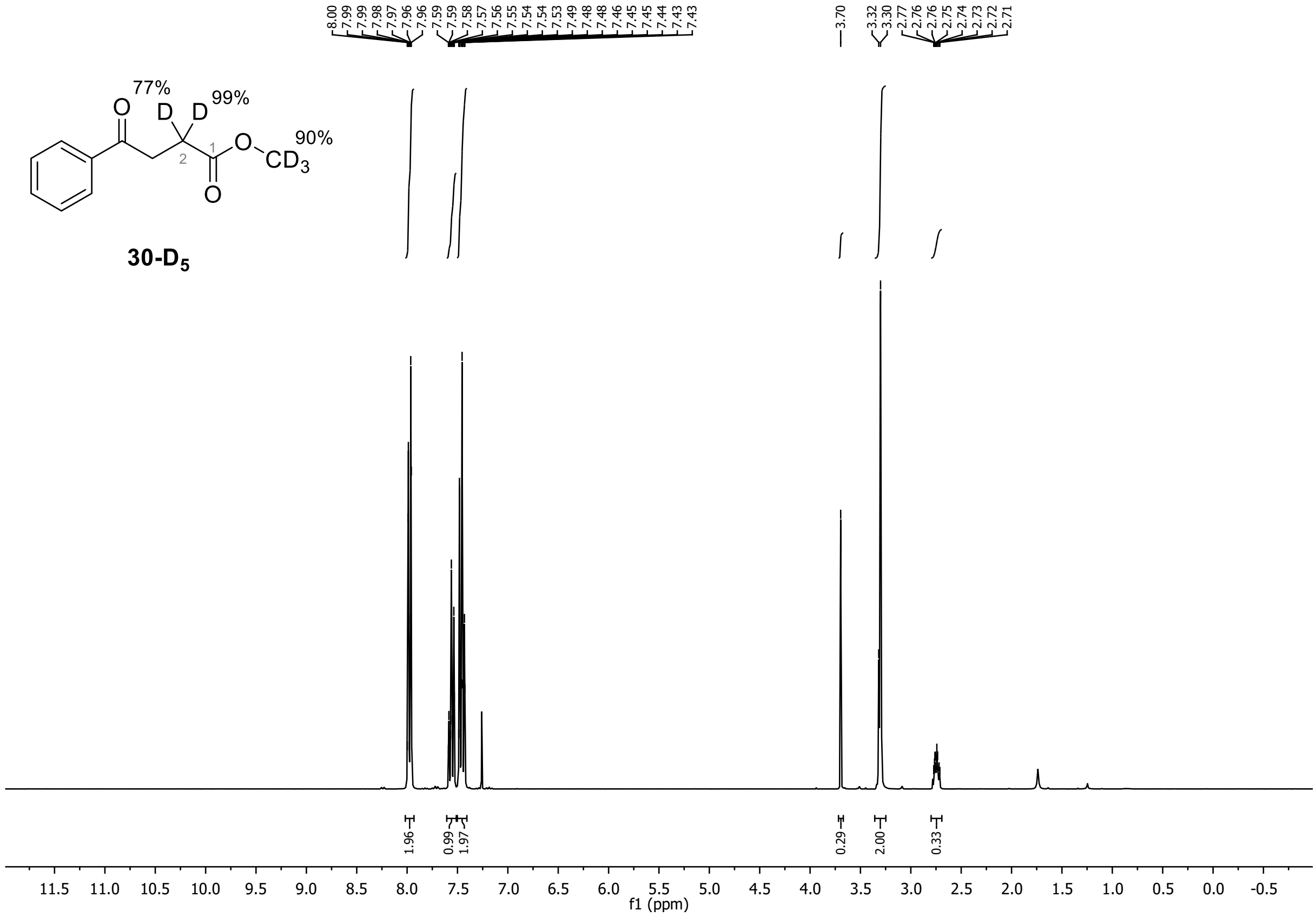

Figure S149. ^{1}H NMR spectrum (300 MHz, $CDCl_3$) of **30-D$_5$**.

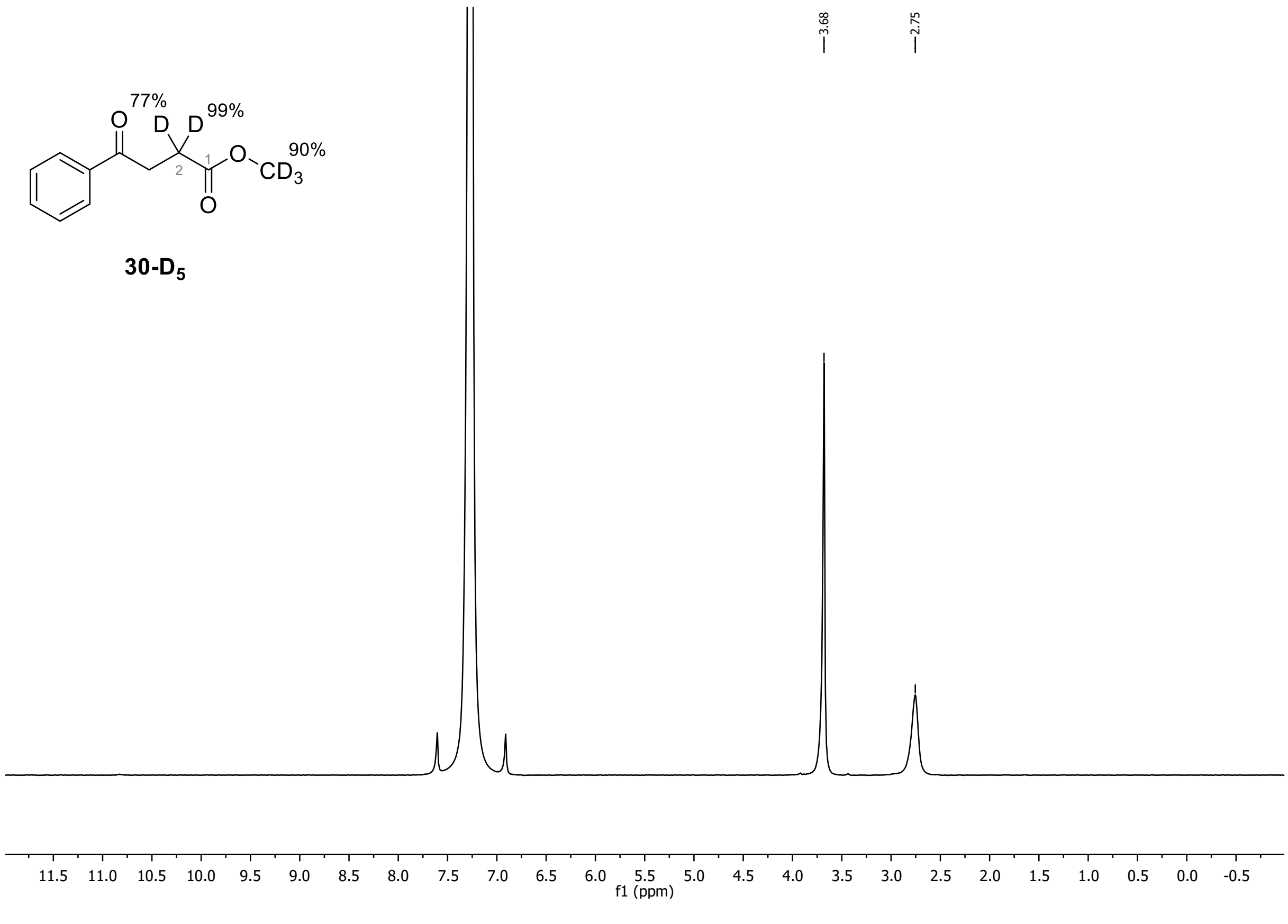

Figure S150. ^{2}H NMR spectrum (46 MHz, $CDCl_3$) of **30-D$_5$**.

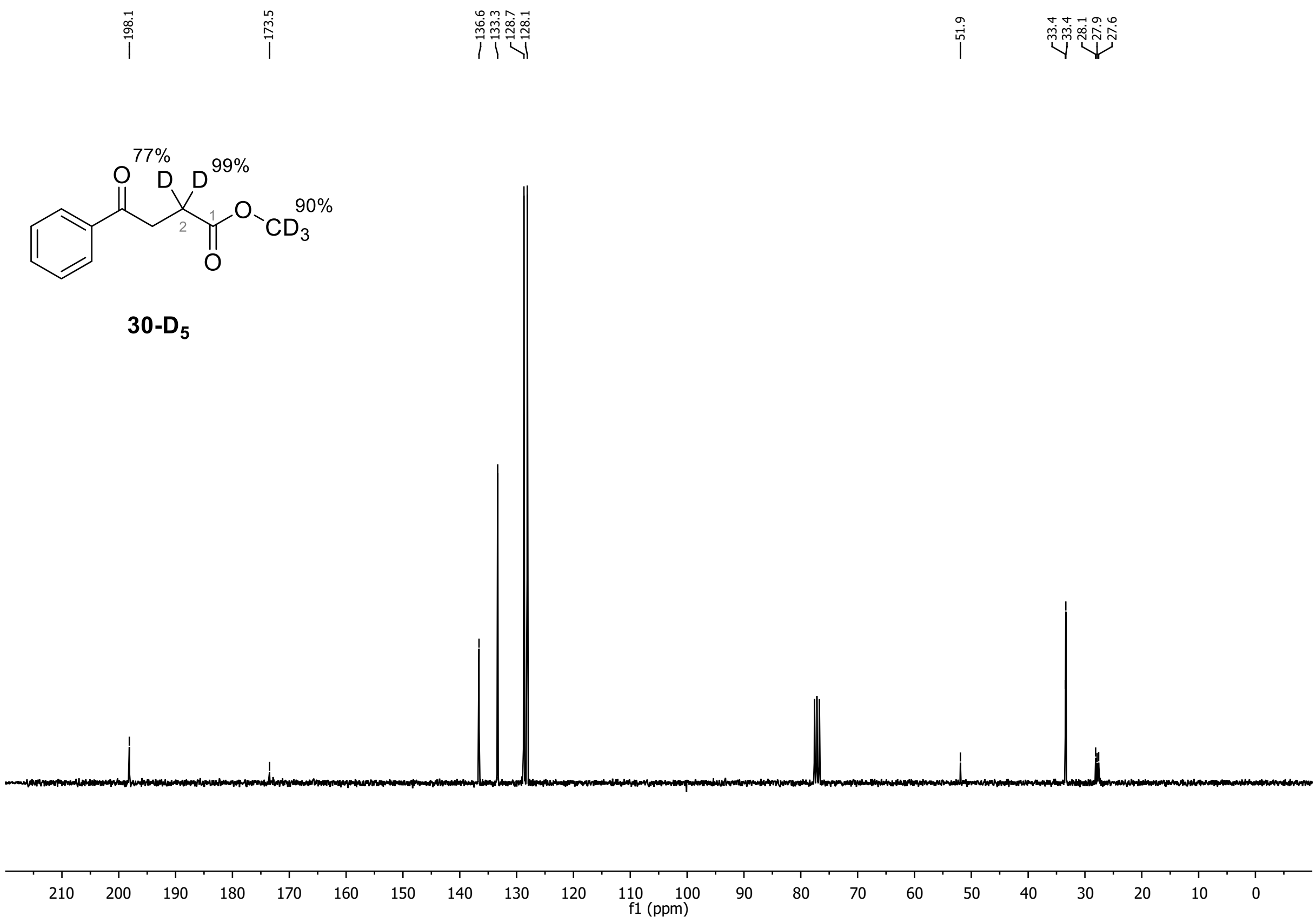

Figure S151. ^{13}C NMR spectrum (75 MHz, $CDCl_3$) of **30-D$_5$**.

Methyl 4-oxo-4-phenylbutanoate-2,2,3,3-*d*4 (30-D4):

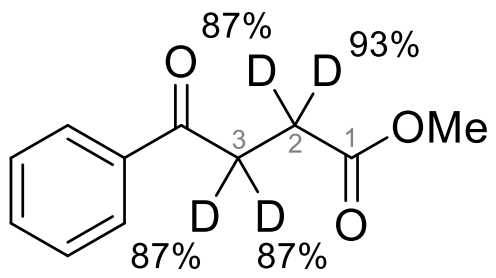

30-D4

$C_{11}H_8D_4O_3$ (196.24 g/mol)

Following **GP-C**, **30-D4** was synthesized using **S1** (293 mg, 1.0 mmol, 1.0 equiv.) and methyl acrylate-d_3 (**S30b**) (178 mg, 2.0 mmol, 2 equiv., 87% D). Purification by flash column chromatography (*n*-hexane/EtOAc, gradient 100:0 to 70:30 over 10 CV) afforded **30-D4** (169 mg, 861 µmol, 86%) as colorless oil.

The deuterium incorporation was determined:

^{1}H NMR: 87% D in position 3; 90% D in position 2.

Given that the D-incorporation of the starting material did not change during the reaction, 93% D was incorporated during the deuteroacylation reaction.

R$_f$: 0.47 (*n*-hexane/EtOAc 80:20).

^{1}H NMR (300 MHz, $CDCl_3$, δ): 8.03 – 7.93 (m, 2H), 7.61 – 7.52 (m, 1H), 7.51 – 7.41 (m, 2H), 3.70 (s, 3H), 3.28 (m, 0.26H), 2.74 (m, 0.20H).

^{2}H NMR (46 MHz, $CDCl_3$, δ): 3.30, 2.74.

^{13}C{^{1}H} NMR (75 MHz, $CDCl_3$, δ): 173.5, 136.6, 133.4, 128.7, 128.2, 51.9, 33.1 (t, J = 19.5 Hz), 27.7 (t, J = 19.8 Hz).

Note: The ketone carbon was not detected due to low intensity, most likely caused by the absence of protons for polarization transfer (INEPT).

HR-MS (ESI): calc. for $[M+H]^+$ 197.11158, found 197.11078 (err. 4.1 ppm).

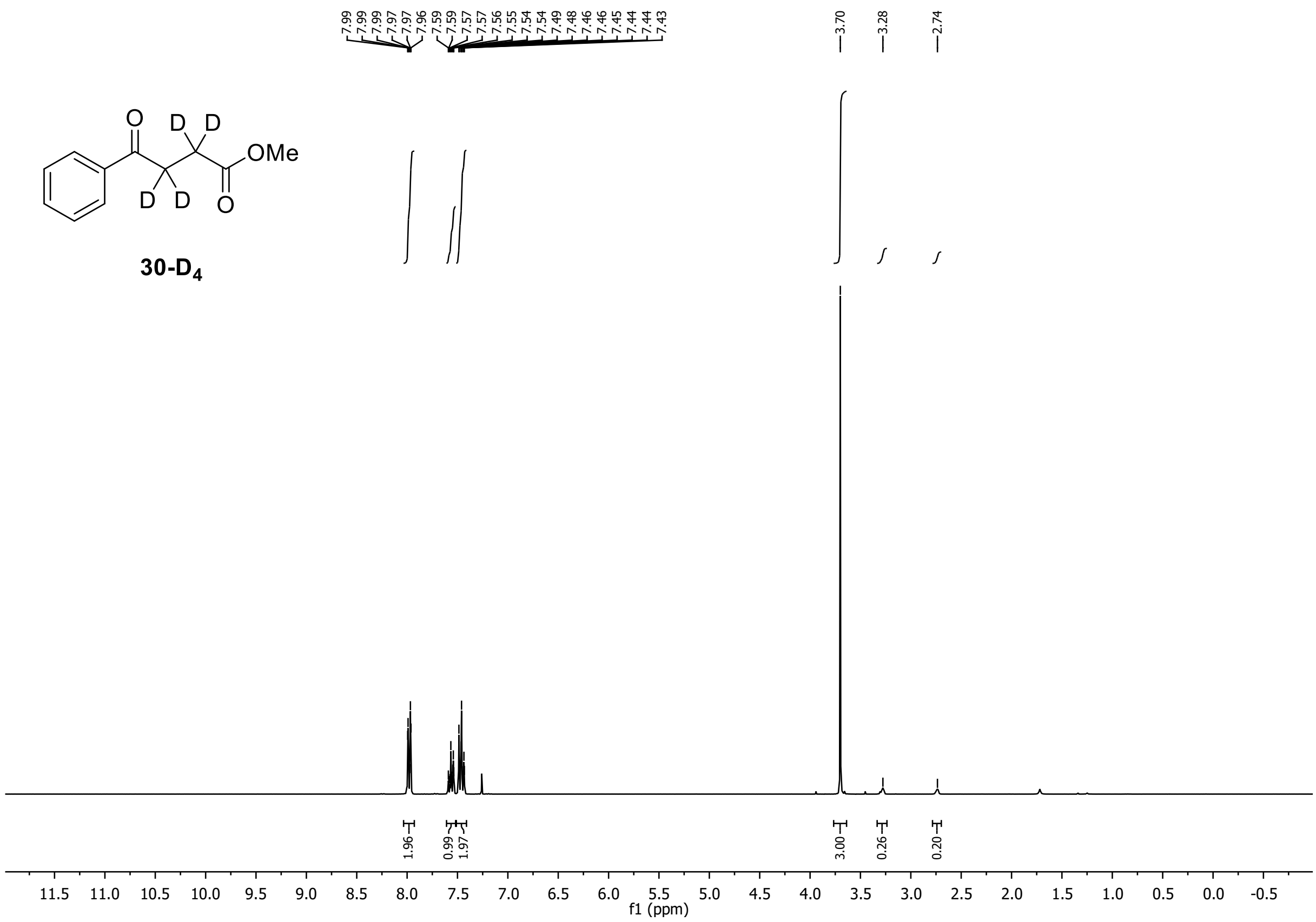

Figure S152. ^{1}H NMR spectrum (300 MHz, $CDCl_3$) of **30-D$_4$**.

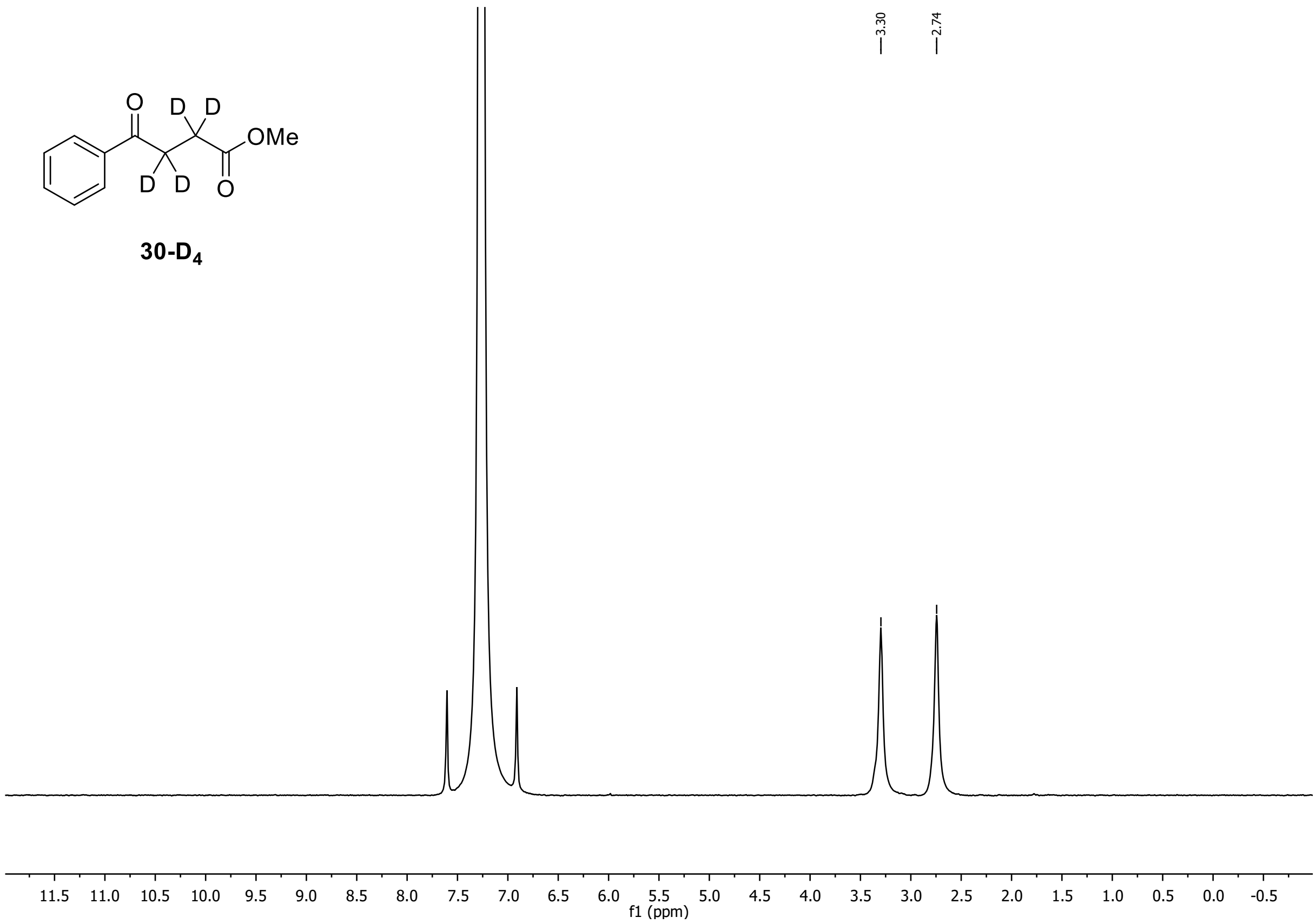

Figure S153. ^{2}H NMR spectrum (46 MHz, $CDCl_3$) of **30-D$_4$**.

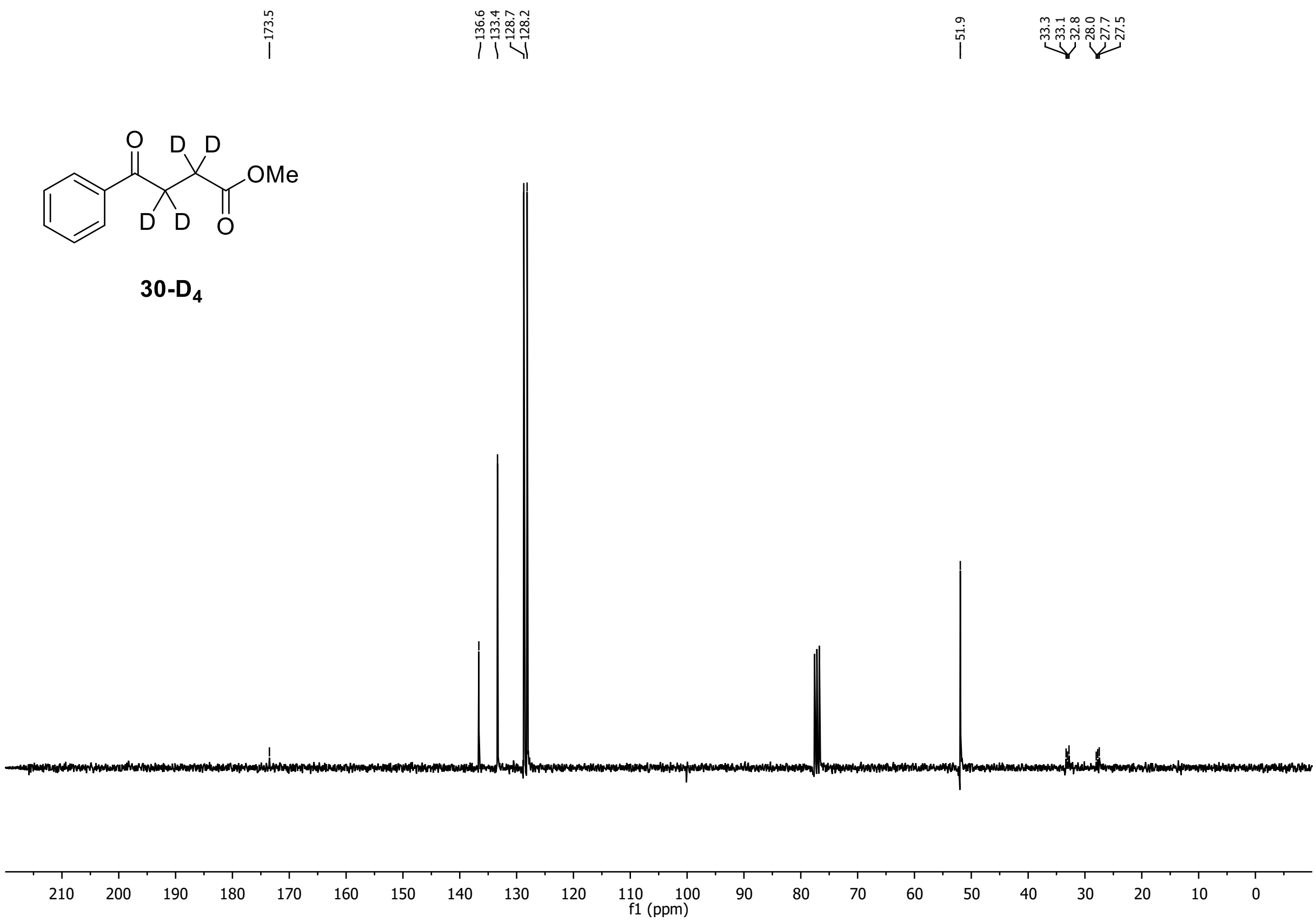

Figure S154. ^{13}C NMR spectrum (75 MHz, $CDCl_3$) of **30-D4**.

1-Phenylbutane-3-d-1,4-diol (31-D):

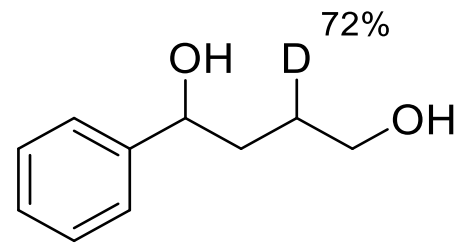

31-D

$C_{10}H_{13}DO_2$ (167.23 g/mol)

A modified literature procedure was followed.[32]

1-D (207 mg, 1.0 mmol, 1.0 equiv.) was dissolved in THF (20 mL), cooled to 0 °C and $LiAlH_4$ (2.0 M in THF, 2.0 mL, 4.0 mmol, 4.0 equiv.) was added dropwise. Water (5 mL) and EtOAc (10 mL) were added. The aqueous layer was extracted with EtOAc (2 × 10 mL), the combined organic layers were washed with brine (10 mL), dried over $MgSO_4$, filtered and concentrated *in vacuo*. **31-D** (168 mg, 1.0 mmol, quant.) was received as a colorless oil.

The deuterium incorporation was determined: 72% D (^{1}H NMR).

^{1}H NMR (300 MHz, $CDCl_3$, δ): 7.39 – 7.18 (m, 5H), 4.66 (t, *J* = 6.3 Hz, 1H), 3.64 – 3.56 (m, 2H), 3.36 (br s, 2H), 1.86 – 1.75 (m, 2H), 1.66 – 1.55 (m, 1.28H).

^{2}H NMR (46 MHz, $CDCl_3$, δ): 1.64.

^{13}C{^{1}H} NMR (75 MHz, $CDCl_3$, δ): 144.8, 128.5, 127.5, 125.9, 74.3, 62.7, 36.3, 29.9 (C–H), 28.8 (t, *J* = 19.1 Hz, C–D).

HR-MS (ESI): m/z calc for $[M+Na]^+$ 190.09488, found 190.09493 (err. 0.29 ppm).

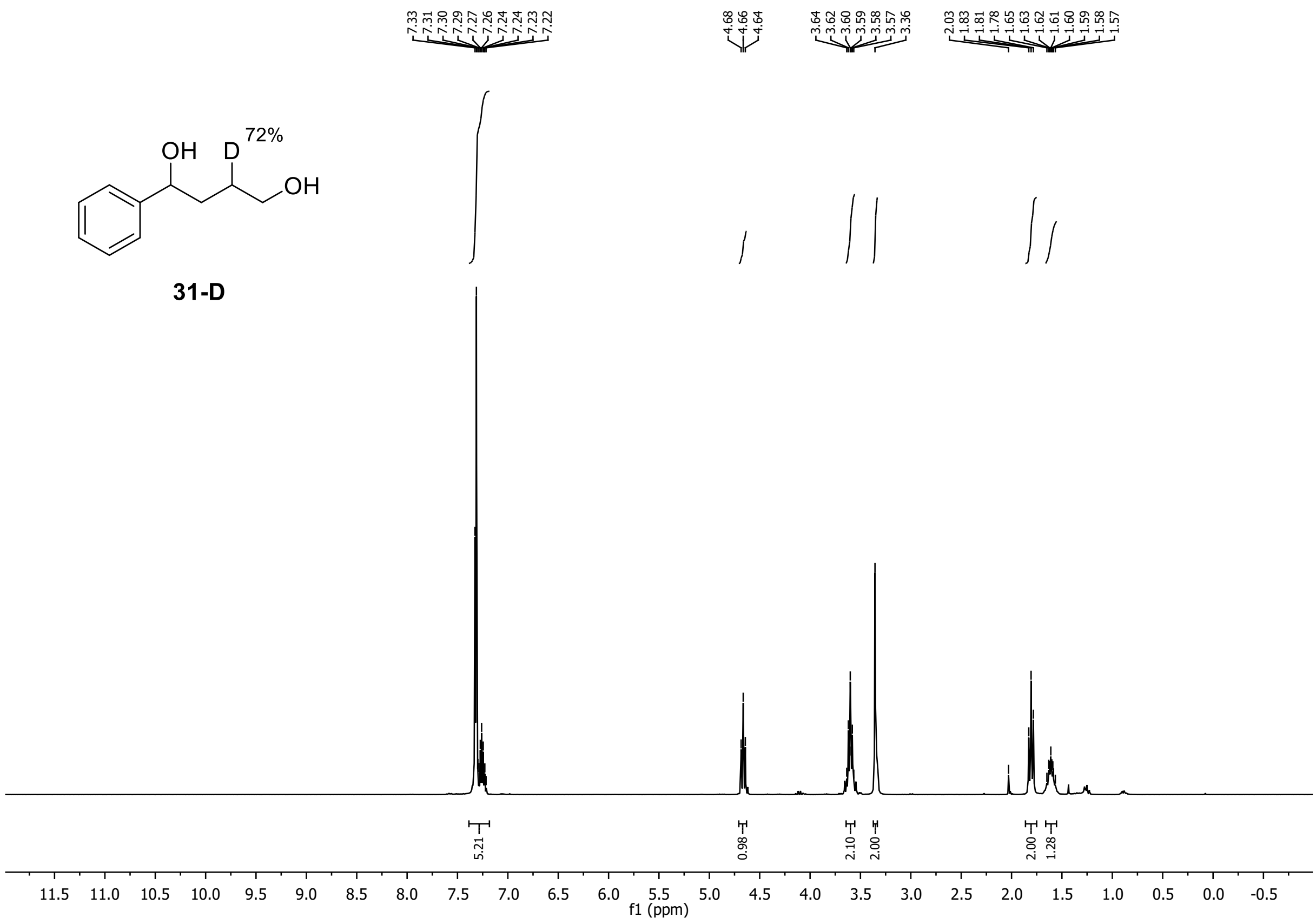

Figure S155. ^{1}H NMR spectrum (300 MHz, $CDCl_3$) of **31-D**.

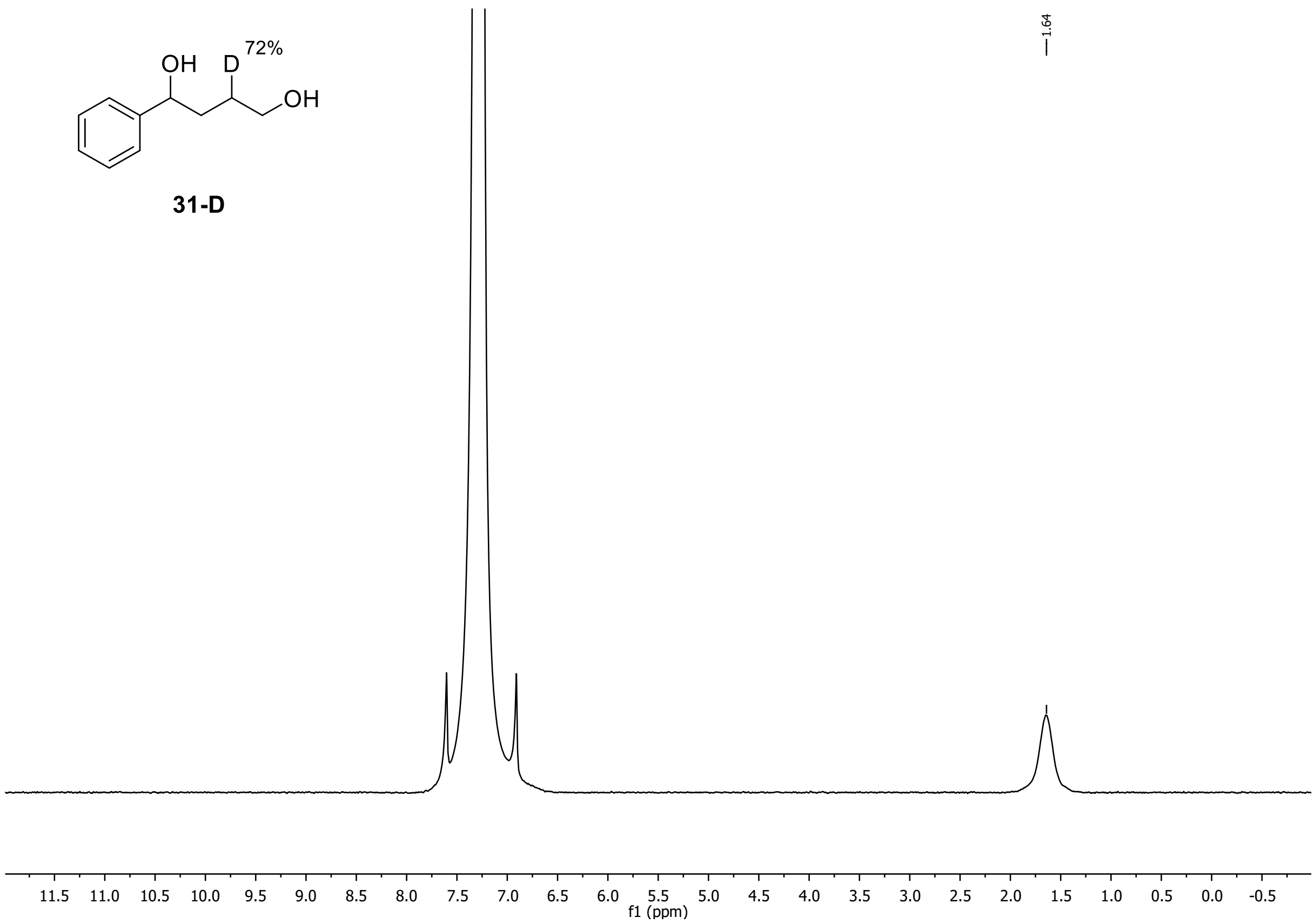

Figure S156. ^{2}H NMR spectrum (46 MHz, $CDCl_3$) of **31-D**.

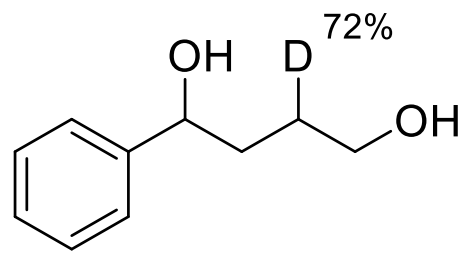

31-D

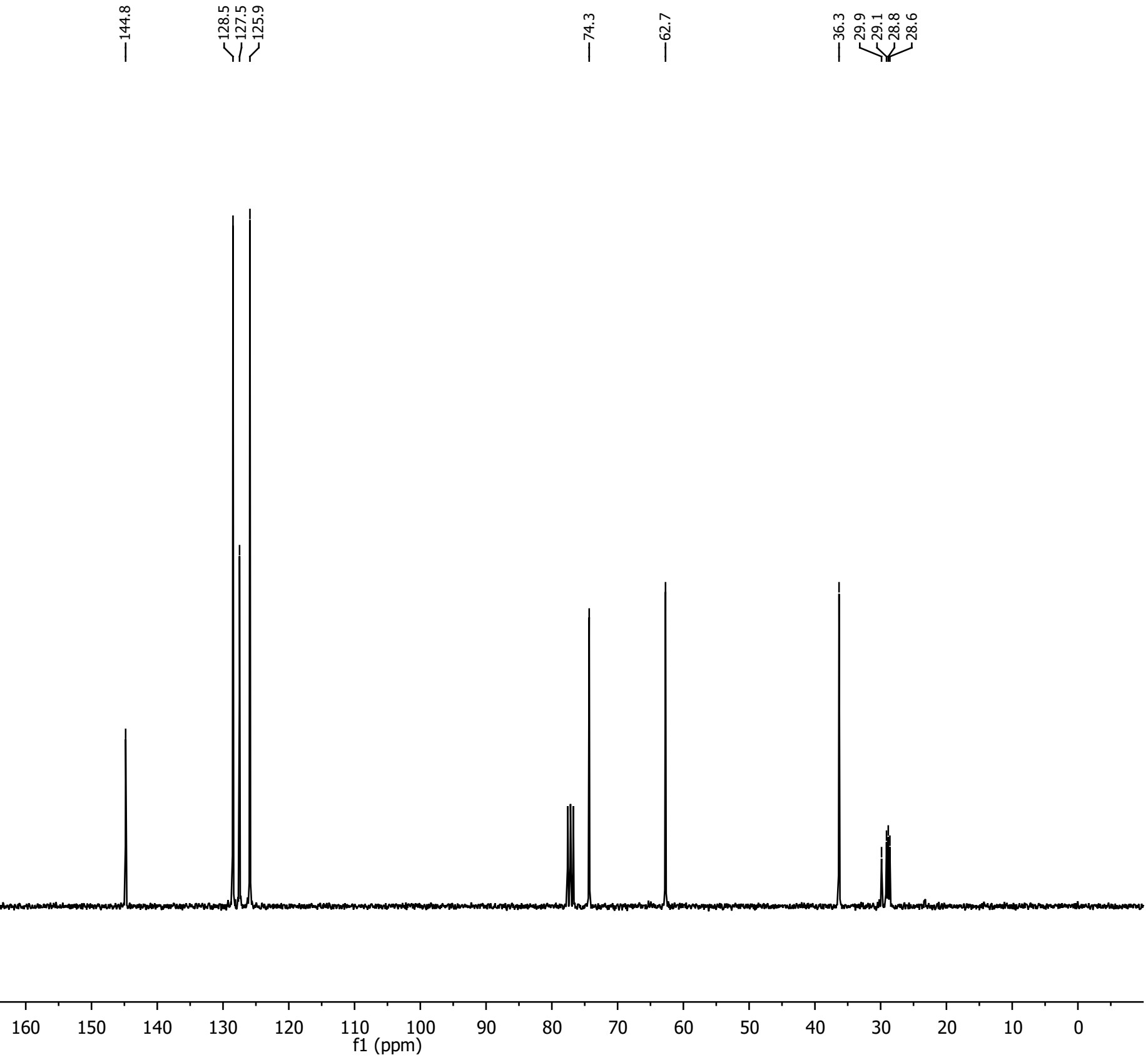

Figure S157. ^{13}C NMR spectrum (75 MHz, $CDCl_3$) of **31-D**.

2-(4-Fluorophenyl)-6-phenyl-4,5-dihydropyridazin-3(2*H*)-one-4-*d* (32-D):

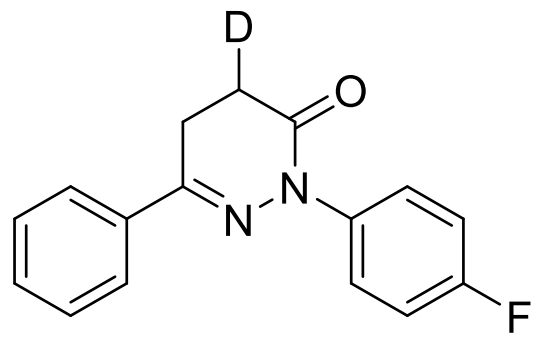

32-D

$C_{16}H_{12}DFN_2O$ (269.30 g/mol)

A modified literature procedure was followed.[33]

1-D (207 mg, 1.0 mmol, 1.0 equiv.) and 4-fluorophenylhydrazine hydrochloride (244 mg, 1.5 mmol, 1.5 equiv.) were dissolved in EtOH (5 mL) and stirred at 80 °C for 18 h. Water (5 mL) and EtOAc (10 mL) were added. The aqueous layer was extracted with EtOAc (2 × 10 mL), the combined organic layers were dried over $MgSO_4$, filtered and concentrated *in vacuo*. Purification by flash column chromatography (*n*-hexane/EtOAc, gradient 100:0 to 0:100 over 10 CV) afforded **32-D** (164 mg, 609 µmol, 61%) as pale brown solid.

The deuterium incorporation was determined: 78% D (^{1}H NMR).

R_f: 0.72 (*n*-hexane/EtOAc 50:50).

m.p.: 81.5 °C.

^{1}H NMR (300 MHz, $CDCl_3$, δ): 7.85 – 7.75 (m, 2H), 7.62 – 7.52 (m, 2H), 7.47 – 7.39 (m, 3H), 7.19 – 7.03 (m, 3H), 3.16 – 3.05 (m, 2H), 2.82 – 2.72 (m, 1H).

^{2}H NMR (46 MHz, $CDCl_3$, δ): 2.78.

^{13}C{^{1}H} NMR (75 MHz, $CDCl_3$, δ): 165.5, 160.9 (d, J = 246.0 Hz), 151.9, 137.4 (d, J = 3.0 Hz), 135.4, 130.2, 128.8, 126.7 (d, J = 8.5 Hz), 126.2, 115.4 (d, J = 22.8 Hz), 27.7 (m), 22.9.

^{19}F NMR (282 MHz, $CDCl_3$, δ): -115.6.

HR-MS (ESI): m/z calc for $[M+H]^+$ 270.11474, found 270.11439 (err. 1.3 ppm).

IR (ATR, $\tilde{\nu}$ [cm^{-1}]): 3066 (w), 2969 (w), 1672 (s), 1597 (w), 1567 (w), 1504 (m), 1440 (w), 1385 (w), 1329 (s), 1287 (m), 1258 (w), 1220 (m), 1188 (m), 1157 (m), 1124 (m), 1084 (m), 1042 (w), 1004 (m), 975 (w), 949 (w), 926 (w), 841 (m), 807 (m), 766 (m), 747 (m), 721 (m), 695 (s).

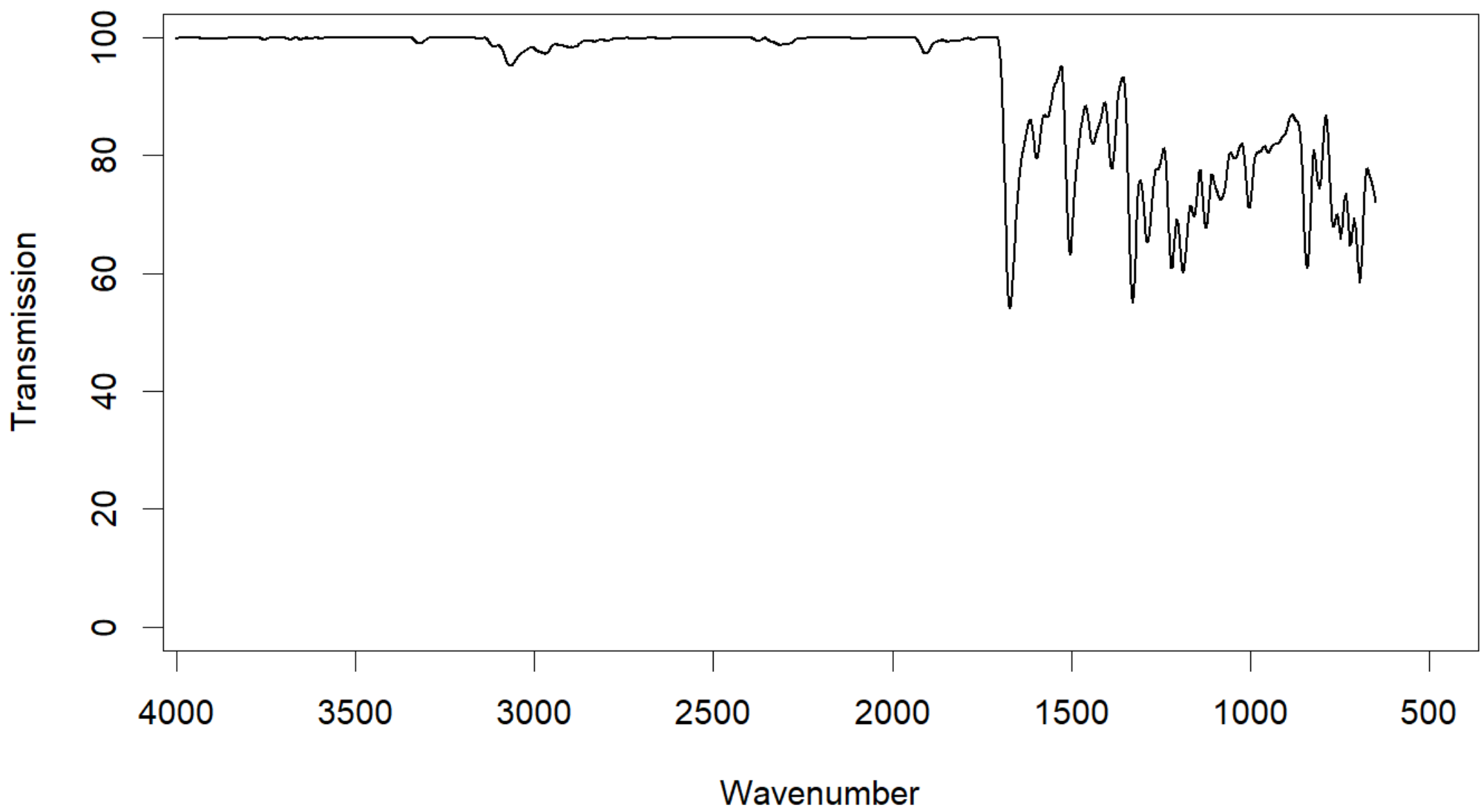

Figure S158. IR spectrum (ATR, neat) of **32-D**.

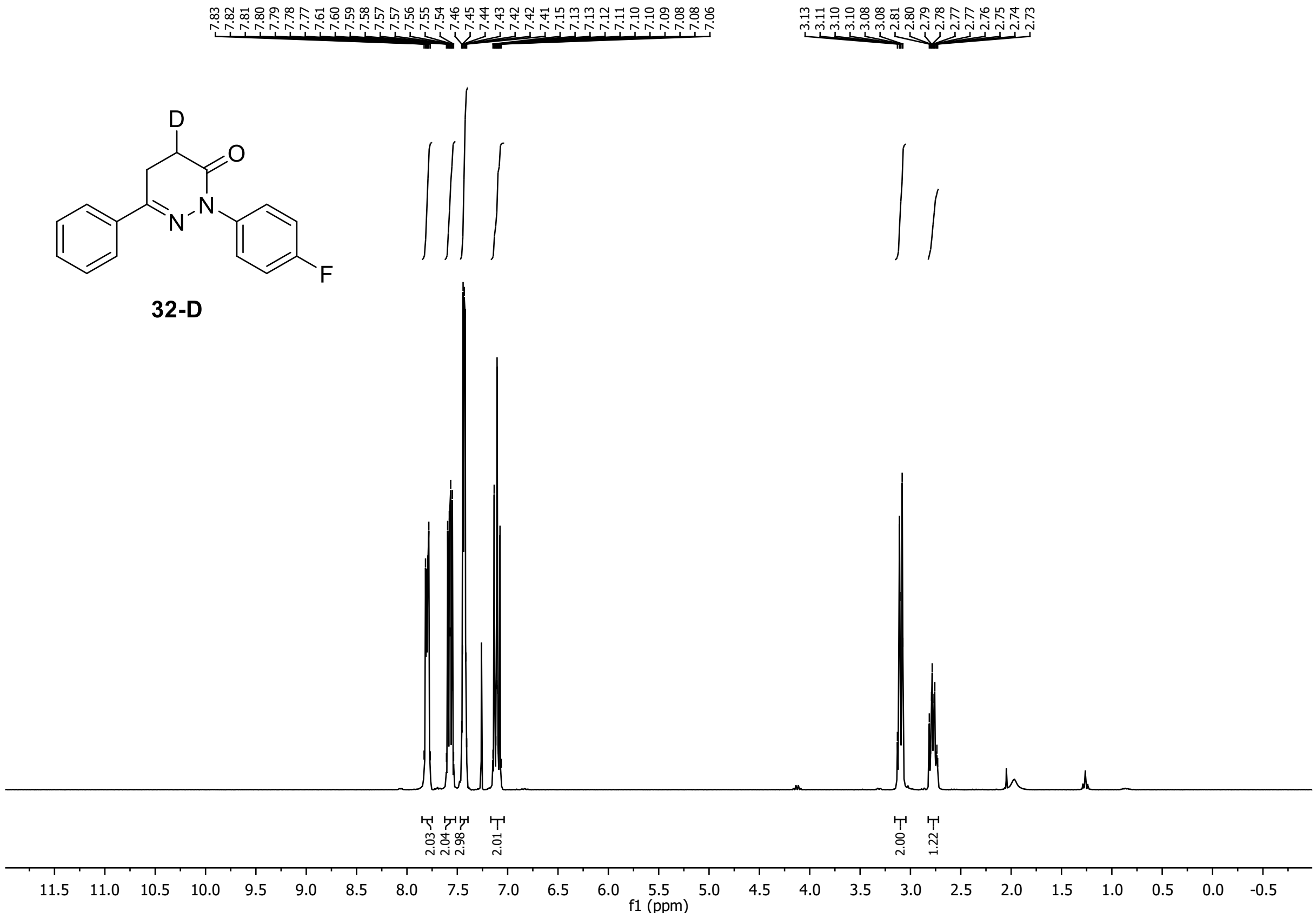

Figure S159. ^{1}H NMR (300 MHz, $CDCl_3$) spectrum of **32-D**.

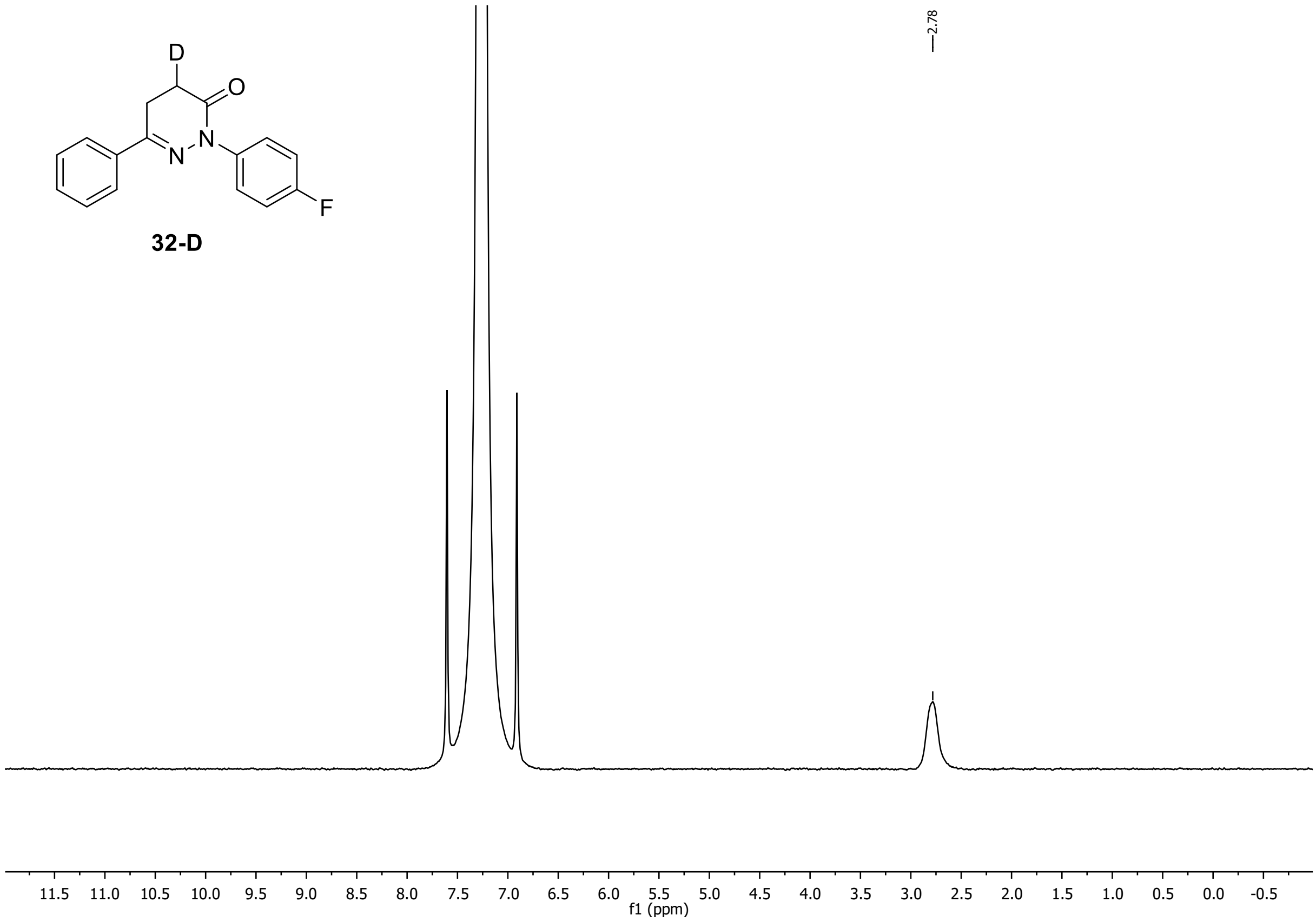

Figure S160. ^{2}H NMR (46 MHz, $CDCl_3$) spectrum of **32-D**.

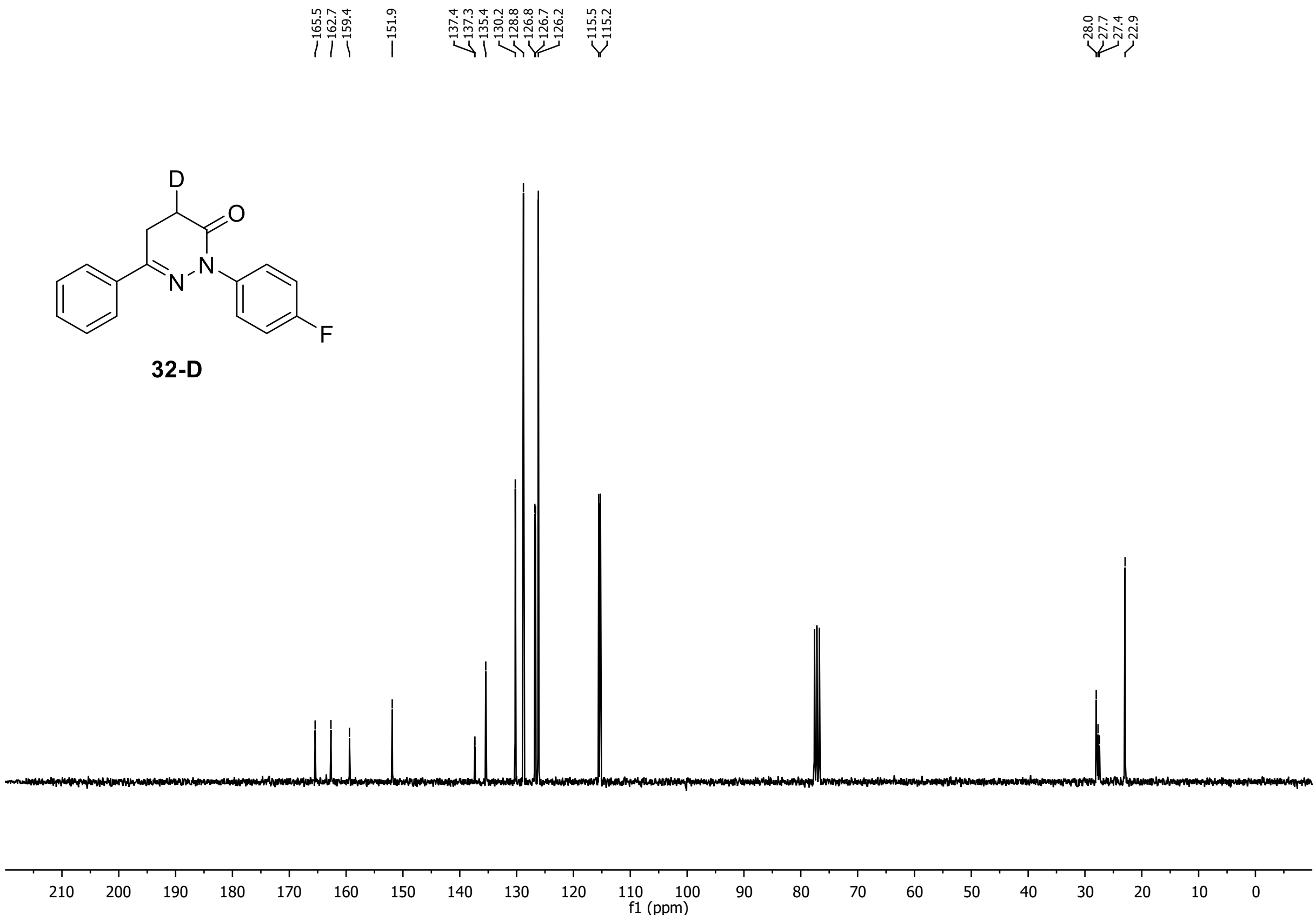

Figure S161. ^{13}C NMR (75 MHz, $CDCl_3$) spectrum of **32-D**.

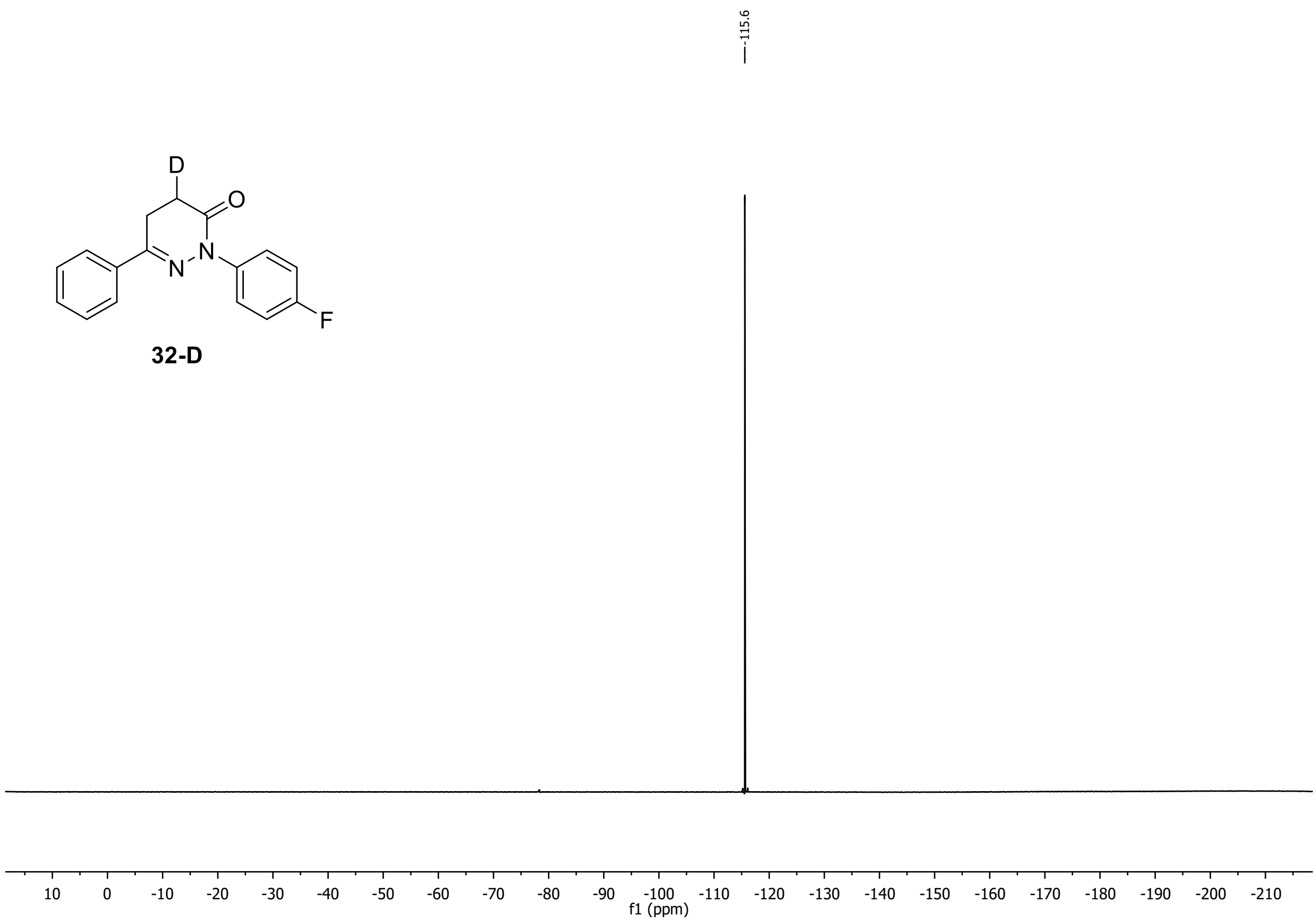

Figure S162. ^{19}F NMR (282 MHz, $CDCl_3$) spectrum of **32-D**.

6. Computational Details

All reported computations were performed with the gaussian16 program.[34] Geometry optimizations were optimized using the PBE0 exchange-correction functional[35] and dispersion correction with Becke-Johnson damping (PBE0-D3(BJ)).[36] Calculations involving the open-shell Ni(I)/Ni(III) pathway were performed using the unrestricted PBE0 functional (UPBE0). The def2-TZVP basis set was utilized for the nickel atoms, while all other atoms were treated with the def2-SVP basis set.[37, 38] The resulting structures were confirmed to be stationary points at local minima (no imaginary frequency) or transitions states (one imaginary frequency) by subsequent vibrational frequency analysis at the same level of theory. Connections of the transition states between the reactants and products were confirmed by intrinsic reaction coordinate (IRC) calculations.[39, 40]

Single-point energy calculations were performed at the same level of theory with implicit solvent effects (*N*,*N*-dimethylformamide) accounted for using the Solvent Model Density (SMD) method.[41] The given Gibbs free energies are the sum of electronic energy in solution and thermal correction in gas phase at 298 K and 1 atm in kcal/mol.

Hirshfeld-CM5 atomic charges (in e) were computed at the same level of theory on optimized geometries using Gaussian 16.[42, 43] Reported charges correspond to the electronic ground state. The Hirshfeld charges are used qualitatively to illustrate charge redistribution along the reaction coordinate.

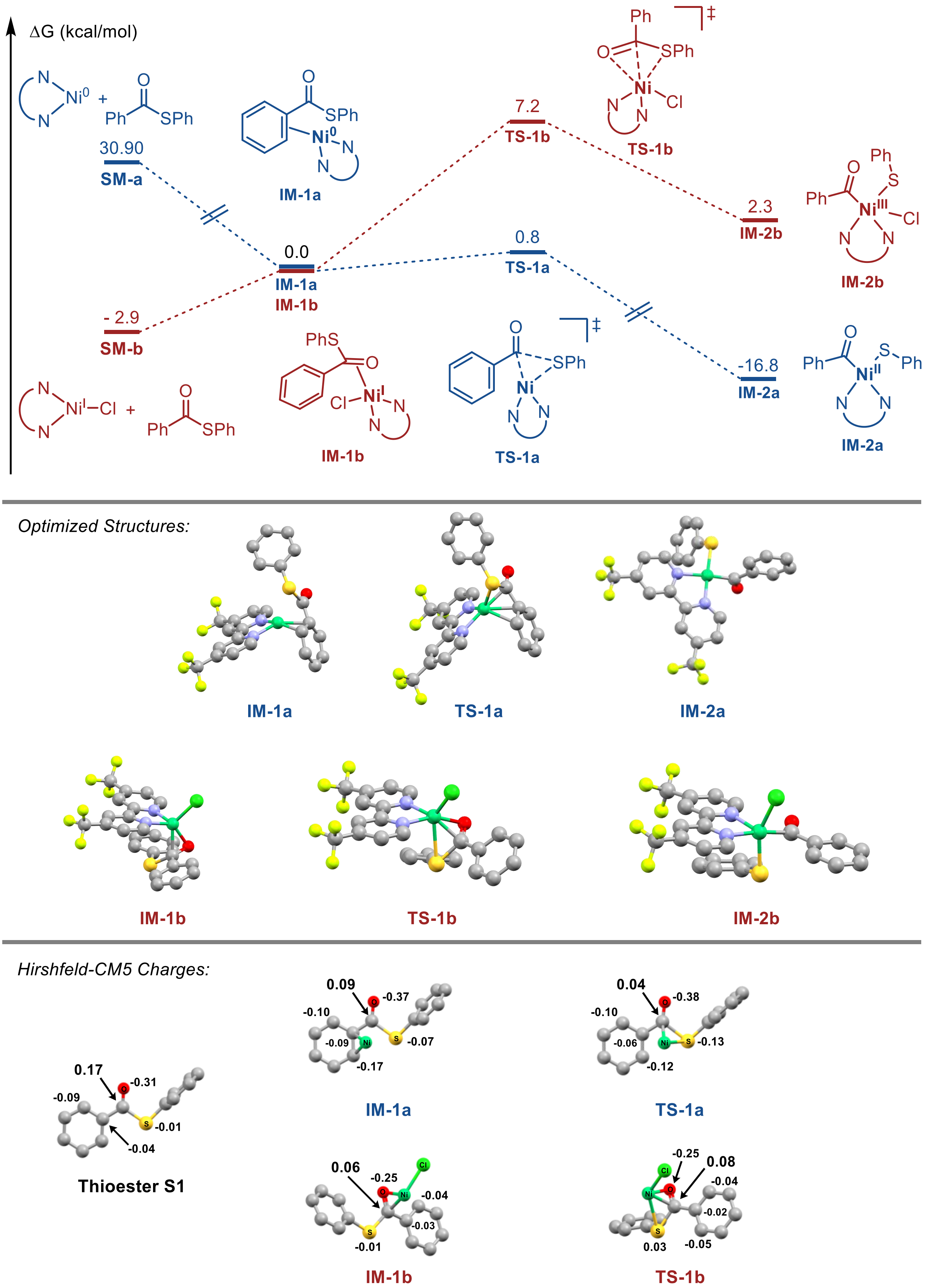

Figure S163. Computational analysis of the oxidative addition of *S*-phenyl benzothioate to (**L5**)Ni(0) or (**L5**)Ni(I)–Cl at the PBE0-D3(BJ)(SMD:DMF) /def2-TZVP(Ni)+def2-SVP(others) level of theory.

(L5)Ni^0

0 1

N	1.23370500	1.67280100	-0.41186000
C	2.58555800	1.80862900	-0.42100700
C	3.45321400	0.75742300	-0.25045100
C	2.91610800	-0.53967300	-0.03900700
C	1.54561000	-0.69036000	0.04939900
C	0.71121400	0.42759900	-0.10146500
C	-0.71120400	0.42759600	0.10155100
C	-1.54557600	-0.69039000	-0.04924400
C	-2.91607600	-0.53972300	0.03909200
C	-3.45321400	0.75739100	0.25038100
C	-2.58557900	1.80861700	0.42090700
N	-1.23372300	1.67279900	0.41183500
C	-3.84075200	-1.70178800	-0.10998400
C	3.84078000	-1.70175000	0.10998400
F	-4.70788400	-1.52954600	-1.12272300
F	-4.59135000	-1.88797400	0.98959500
F	-3.19459400	-2.84892600	-0.33996200
F	4.59075500	-1.88842500	-0.98994700
F	3.19466200	-2.84876100	0.34073500
F	4.70848800	-1.52918900	1.12216600
Ni	-0.00003800	3.01773500	0.00001900
H	2.96338900	2.81216900	-0.63647100
H	4.53013600	0.92192100	-0.32359700
H	1.10773900	-1.66495700	0.27160100
H	-1.10767300	-1.66499400	-0.27134700
H	-4.53014300	0.92188300	0.32346600
H	-2.96342500	2.81217000	0.63627900

PBE0-D3 (E_h): **-2675.487748**

Therm. corr. G-E (E_h): **0.119467**

(L5)Ni^{I}–Cl

0 2

C	3.46630600	0.16207600	-0.04703300
C	2.86706300	-1.09652300	-0.03378700
C	0.72741000	-0.02701500	-0.02831100
C	2.64272300	1.27976200	-0.04494100
C	-0.74669400	0.00895300	-0.02106600
C	-2.93365100	-0.96145000	-0.02511700
C	-3.47383600	0.32379800	-0.00035200
C	-2.59973800	1.40217600	0.01134300
H	4.55075200	0.28101600	-0.05965500
H	3.06212600	2.28869300	-0.05315400
H	-4.55167500	0.49342800	0.00655900
H	-2.97342100	2.42866200	0.02803200
N	-1.27228200	1.24946900	0.00138800
N	1.31058000	1.18714000	-0.03358700
Ni	0.05065400	2.70850000	-0.00050800
Cl	0.21502700	4.87904100	0.02995400
C	-1.55397200	-1.12706500	-0.03743300
H	-1.11699600	-2.12479400	-0.06448200
C	1.48093500	-1.20004000	-0.02691200
H	1.00003700	-2.17822300	-0.02076300
C	-3.84459400	-2.16001300	-0.00116300
C	3.72174100	-2.33516800	0.01406900
F	-3.22422700	-3.26719500	-0.41051200
F	-4.91031000	-1.98032600	-0.78472100
F	-4.30112700	-2.39413500	1.23445900
F	3.06153500	-3.41298700	-0.41133900
F	4.13328900	-2.58909500	1.26149900
F	4.81474700	-2.20555900	-0.74071200

PBE0-D3 (E_h): --3135.570933

Therm. corr. G-E (E_h): 0.118911

S-Phenyl benzothioate

0 1

C	4.02615300	-1.19185300	-0.00058700
C	4.82336500	-0.04633000	-0.00009800
C	4.23162900	1.21851700	0.00043700
C	2.84591600	1.33777800	0.00048500
C	2.03872300	0.19064800	0.00000800
C	2.63857400	-1.07637500	-0.00053400
C	0.56285100	0.39334000	0.00008900
S	-0.41046800	-1.13009700	0.00037900
O	0.03999800	1.47826700	0.00005400
C	-2.05978300	-0.46951600	0.00011100
C	-2.71402000	-0.23411300	1.21375500
C	-4.02235800	0.24772600	1.20869900
C	-4.67585100	0.48957900	-0.00032700
C	-4.02171500	0.24837200	-1.20913700
C	-2.71338700	-0.23348800	-1.21375700
H	4.48705900	-2.18249300	-0.00102400
H	5.91250600	-0.14016900	-0.00013900
H	4.85523000	2.11604400	0.00081500
H	2.36892800	2.32011300	0.00090000
H	2.02943300	-1.98392500	-0.00096300
H	-2.19930100	-0.42742100	2.15811000
H	-4.53379200	0.43363500	2.15668400
H	-5.70229300	0.86546100	-0.00050000
H	-4.53264800	0.43478600	-2.15729200
H	-2.19817600	-0.42633200	-2.15793900

PBE0-D3 (E_h): -973.50321

Therm. corr. G-E (E_h): 0.150701

IM-1a

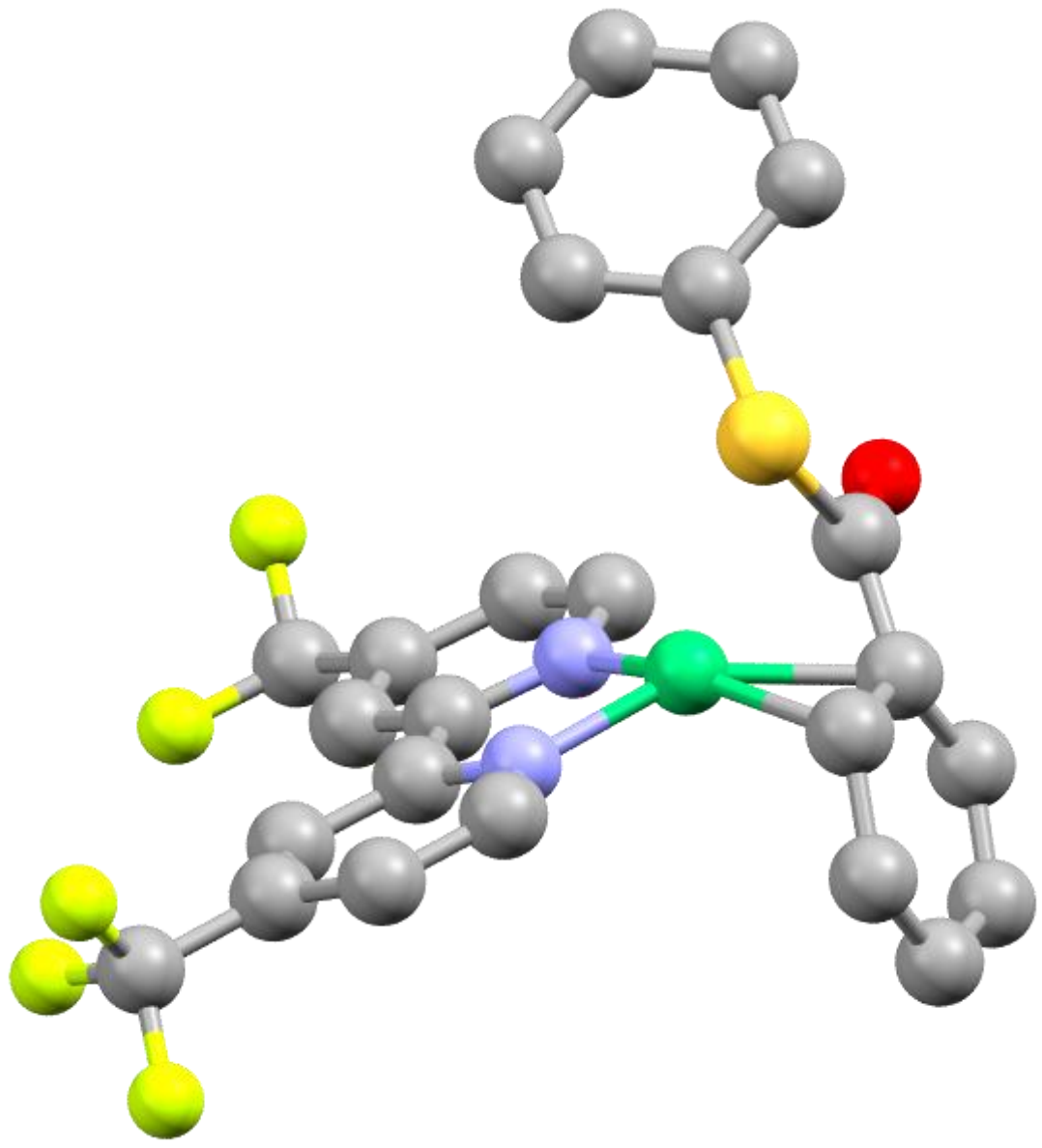

0 1

N	1.17804600	-1.29445200	-0.22892500
C	1.87607100	-2.33067400	-0.71031700
C	3.20604200	-2.22495100	-1.08805400
C	3.83566800	-0.98606900	-0.95535100
C	3.11814600	0.09544600	-0.46383300
C	1.78038000	-0.09224400	-0.11010900
C	0.91174300	0.97816700	0.40684800
C	1.33662200	2.29139700	0.61407500
C	0.42262800	3.21808200	1.09702800
C	-0.89017400	2.81963500	1.35315100
C	-1.23555300	1.49606600	1.11891900
N	-0.35605200	0.59555300	0.66653800
C	0.82582000	4.64741100	1.34629000
C	5.28247600	-0.84613100	-1.34946700
F	0.66286700	4.97968200	2.63119500
F	0.07950900	5.49245300	0.62778000
F	2.10167600	4.87492500	1.03327100
F	5.74114200	0.39082100	-1.16054100
F	5.46288000	-1.14927900	-2.63900300
F	6.05764800	-1.67475100	-0.64236200
Ni	-0.67492900	-1.30854200	0.35121900

H	1.34696400	-3.28204600	-0.79261400
H	3.73366300	-3.09870500	-1.47536900
H	3.59128000	1.07138000	-0.35838800
H	2.36455000	2.58554500	0.40411300
H	-1.63846400	3.52063800	1.72842800
H	-2.25375300	1.12590700	1.28617200
C	-1.33734200	-2.93690300	3.63869000
C	-0.35815600	-3.80357300	3.07147700
C	-0.27479100	-3.95854400	1.71007100
C	-1.13031300	-3.23102300	0.81833900
C	-2.08892800	-2.32236200	1.40507900
C	-2.17289400	-2.20830900	2.83270200
C	-2.79973100	-1.37977400	0.57162300
S	-2.54683700	-1.72019400	-1.27328600
O	-3.56910100	-0.49133700	0.92934500
C	-3.32728400	-0.27673100	-1.94777900
C	-4.72358900	-0.19045600	-1.99467300
C	-5.33004800	0.93201400	-2.55647300
C	-4.55015300	1.96537800	-3.07890800
C	-3.15842000	1.87622700	-3.03648600
C	-2.54421200	0.75979600	-2.46870700
H	-1.42058600	-2.85619300	4.72620300
H	0.30551500	-4.37116100	3.72956800
H	0.43438800	-4.67572200	1.28605700
H	-1.30853800	-3.68334200	-0.16510000
H	-2.92398300	-1.53468400	3.25287300
H	-5.33233400	-1.00404400	-1.59383100
H	-6.42087800	0.99725400	-2.58991400
H	-5.02931700	2.84203500	-3.52267400
H	-2.54335700	2.68239200	-3.44512000
H	-1.45441900	0.69010500	-2.42789200

PBE0-D3 (E_h): **-3649.0679**

Therm. corr. G-E (E_h): **0.297893**

IM-1b

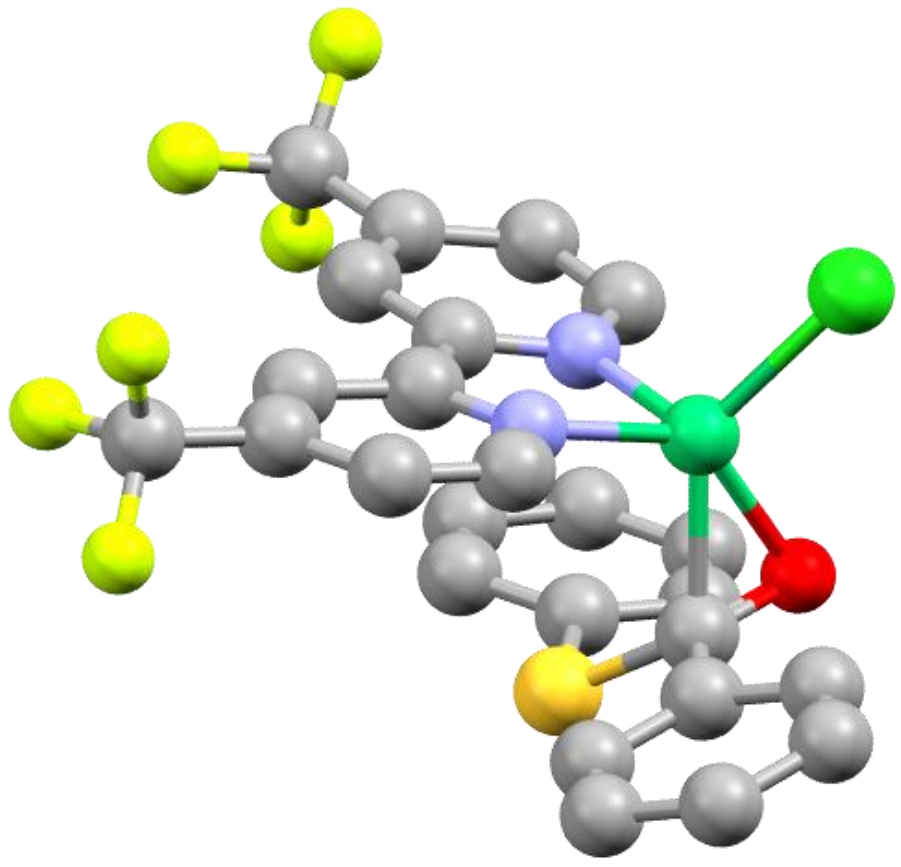

0 2

C	3.36907300	0.26190100	1.09614000
C	3.17187100	1.54134400	0.58003700
C	0.81824700	1.11079800	0.52665800
C	2.25478700	-0.54850700	1.27393300
C	-0.60067900	1.48141300	0.33251900
C	-2.37653600	2.98055100	-0.24113700
C	-3.29237900	1.99607800	0.12743600
C	-2.79760400	0.78077100	0.58218500
H	4.36356000	-0.11148100	1.34589100
H	2.35435900	-1.56584600	1.65901500
H	-4.36963400	2.16076600	0.06463600
H	-3.47155500	-0.02364500	0.88703300
N	-1.49021500	0.53332900	0.67388700
N	1.02221500	-0.13526100	0.98316400
Ni	-0.67221500	-1.20212400	1.27978800
C	4.36676400	2.41825500	0.30484000
C	-2.88373500	4.31072300	-0.73664400
C	-4.36963300	-3.37789400	0.22601800
C	-5.03596100	-2.70212300	-0.79711500
C	-4.30652200	-1.93661600	-1.71002500
C	-2.92193000	-1.84511000	-1.60336400
C	-2.24208700	-2.52912800	-0.58054600
C	-2.98351800	-3.29515100	0.33583200
C	-0.76522800	-2.45333600	-0.39582300

O	-0.19743400	-3.03872700	0.56917600
S	0.10588100	-1.99314800	-1.92292000
H	-4.93327900	-3.98270300	0.94133800
H	-6.12330000	-2.77149900	-0.88494500
H	-4.82177400	-1.40210100	-2.51226500
H	-2.37159200	-1.22879500	-2.31891100
H	-2.45425300	-3.83177400	1.12606200
Cl	-1.11539500	-1.42025200	3.50022800
C	1.82320100	-2.25706700	-1.58722600
C	2.31079900	-3.42983000	-0.99478500
C	2.71868600	-1.28249800	-2.04725900
C	3.68529600	-3.60061200	-0.83774900
H	1.61837100	-4.19942400	-0.65269200
C	4.09224300	-1.47393200	-1.90545600
H	2.33705900	-0.36817900	-2.50923700
C	4.57900600	-2.62856600	-1.29215900
H	4.06175400	-4.51224400	-0.36585400
H	4.78275100	-0.70462800	-2.26073700
H	5.65559200	-2.77451600	-1.17146600
F	-3.69598800	4.15468900	-1.78599400
F	-3.58373000	4.94128000	0.21083200
F	-1.89414000	5.12062700	-1.11060500
F	4.01897300	3.67051100	0.01182500
F	5.07241000	1.94666100	-0.73041300
F	5.19136400	2.46147400	1.35351600
C	-1.01444100	2.72949200	-0.13685400
H	-0.28836000	3.49332400	-0.41379600
C	1.88507700	1.98176000	0.29552000
H	1.71663600	2.98671600	-0.09038200

PBE0-D3 (E_h): **--4109.117055**

Therm. corr. G-E (E_h): **0.297433**

TS-1a:

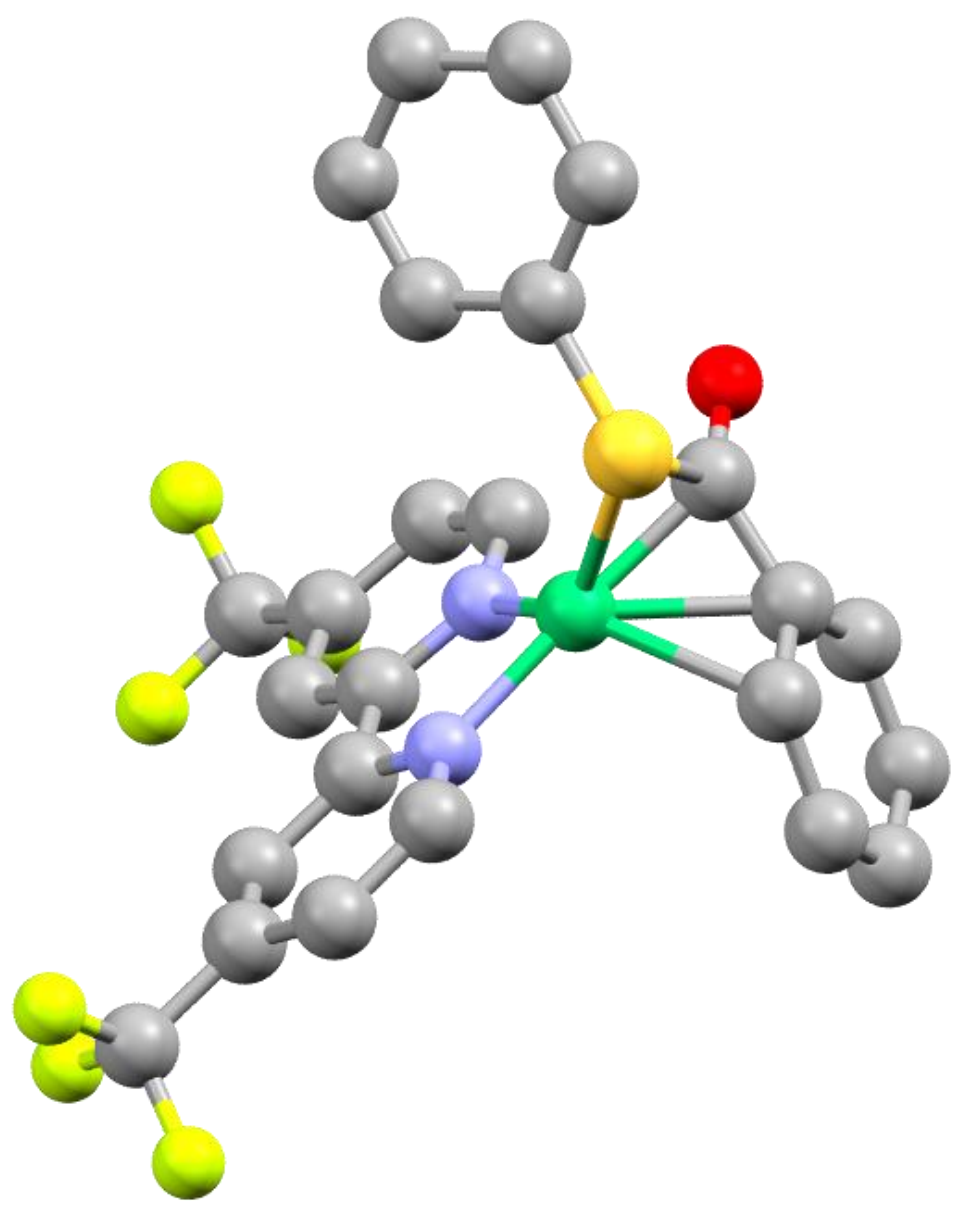

0 1

N	0.79686100	-1.39382800	-0.16783600
C	1.19933300	-2.62496300	-0.50253200
C	2.50021100	-2.90630800	-0.89158700
C	3.41827500	-1.85515900	-0.93269000
C	3.00936700	-0.57451700	-0.58878300
C	1.67991200	-0.37489800	-0.20779900
C	1.12143200	0.93318000	0.17400500
C	1.85753500	2.11958700	0.19441900
C	1.21758000	3.29536800	0.56192000
C	-0.13959700	3.26540200	0.89072200
C	-0.80322100	2.04909300	0.84364700
N	-0.18889600	0.90929500	0.50193300
C	1.95933900	4.60422300	0.60418800
C	4.83540600	-2.12697500	-1.36397900
F	1.88982900	5.16103400	1.81813400
F	1.43819600	5.48490800	-0.25709200
F	3.25027300	4.46559700	0.30106800
F	5.61862000	-1.05676700	-1.22876400
F	4.88643700	-2.50098000	-2.64694100
F	5.37838100	-3.11609700	-0.64779200
Ni	-1.00345000	-0.84650100	0.41046200

H	0.44724100	-3.41616400	-0.45508200
H	2.78362100	-3.92738500	-1.15349100
H	3.71568800	0.25498500	-0.61309700
H	2.91355300	2.12650900	-0.07427000
H	-0.67988700	4.17051700	1.17567000
H	-1.87206500	1.96215900	1.06913500
C	-1.44433500	-1.21267000	4.36433000
C	-0.76509000	-2.42957200	4.16119500
C	-0.88436200	-3.09380200	2.95086200
C	-1.66697400	-2.55620300	1.90972200
C	-2.30612900	-1.30196200	2.09070800
C	-2.19143900	-0.65038800	3.34700600
C	-2.81212700	-0.51894700	0.94741300
S	-2.84455900	-1.67834000	-0.69471400
O	-3.49913600	0.49826700	0.98313800
C	-3.47058900	-0.47985900	-1.84294700
C	-4.81548400	-0.55103700	-2.22769700
C	-5.32366400	0.35264600	-3.16062300
C	-4.49781300	1.33348900	-3.71136200
C	-3.15790300	1.40639400	-3.32618700
C	-2.64258900	0.50313800	-2.39780400
H	-1.37450600	-0.70689400	5.33141300
H	-0.16598500	-2.86087800	4.96746000
H	-0.39820400	-4.06239900	2.80605500
H	-1.90146100	-3.19824500	1.05688100
H	-2.70290400	0.30557600	3.48397400
H	-5.46469700	-1.31605600	-1.79368900
H	-6.37465300	0.29036500	-3.45549400
H	-4.89825600	2.04154100	-4.44163000
H	-2.50520000	2.17214000	-3.75425700
H	-1.59347800	0.55446600	-2.09529100

PBE0-D3 (E_h): **-3649.065889**

Therm. corr. G-E (E_h): **0.297049**

TS-1b:

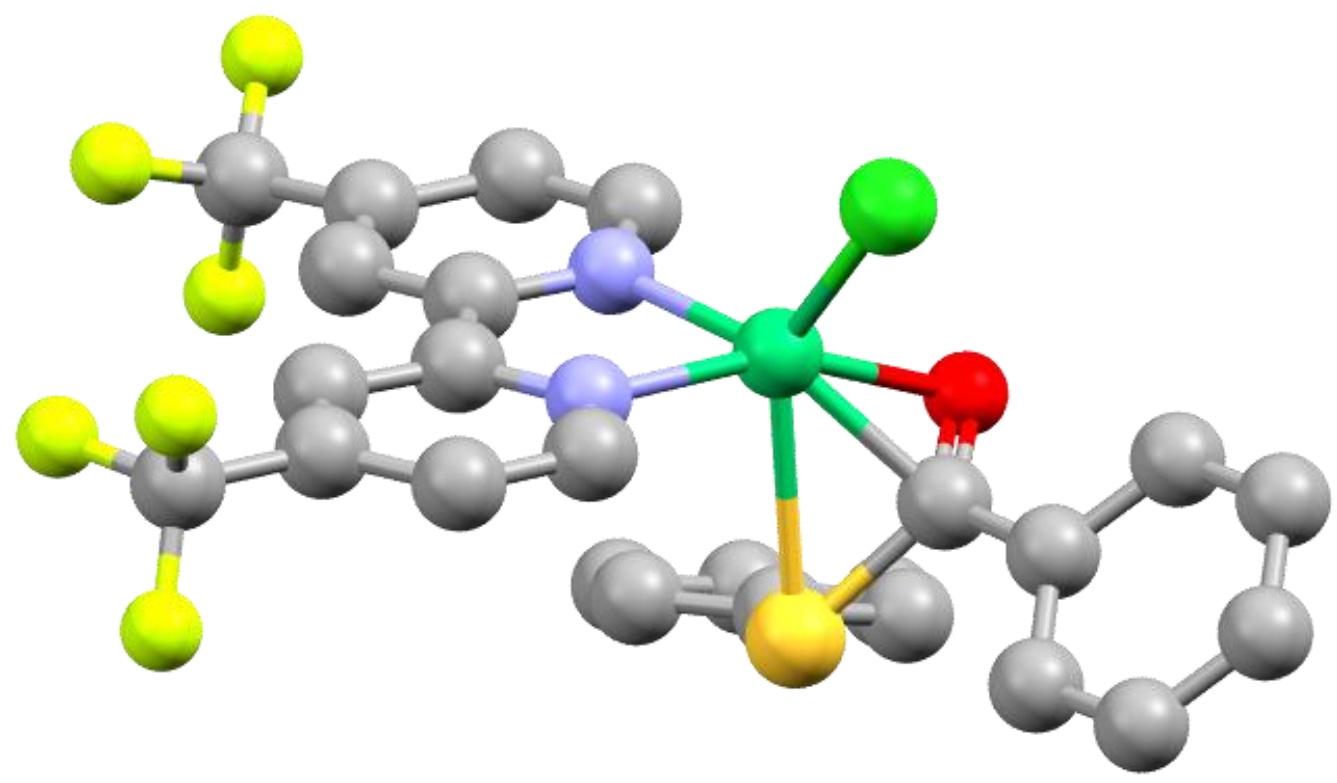

0 2

C	-2.47221600	-2.46750800	-1.38999700
C	-3.43282300	-1.60191200	-0.87460700
C	-1.73329600	0.06533700	-0.61695400
C	-1.16203100	-2.00899000	-1.47581400
C	-1.23017400	1.40840800	-0.23691700
C	-1.48631600	3.67411900	0.49132600
C	-0.10722600	3.84049100	0.37750800
C	0.64711600	2.75399400	-0.04801200
H	-2.72234500	-3.47947000	-1.71198400
H	-0.36755700	-2.65503500	-1.85737800
H	0.37839400	4.79068100	0.60806800
H	1.72918400	2.82921500	-0.17889800
N	0.09675500	1.57564400	-0.33238500
N	-0.80867900	-0.78333700	-1.09477900
Ni	1.15374200	-0.06587900	-0.95275600
C	-4.84959000	-2.08200200	-0.68483500
C	-2.33212000	4.83116700	0.95991200
C	6.17137600	0.12637000	-1.06186100
C	6.59427800	0.93528500	-0.00400200
C	5.74880300	1.14886300	1.08556200
C	4.48967400	0.55414900	1.12281600
C	4.05657900	-0.25288900	0.06027800
C	4.91064800	-0.45991200	-1.03527400

C	2.72630100	-0.90382100	0.03225900
O	2.37915200	-1.74824800	-0.82476200
S	1.71663100	-0.79379300	1.58150300
H	6.83140700	-0.04601600	-1.91621800
H	7.58530100	1.39615900	-0.02755000
H	6.07480500	1.77592800	1.91936200
H	3.84695700	0.71281600	1.99238500
H	4.56310600	-1.08072100	-1.86207700
Cl	2.19625600	1.01092600	-2.68825800
C	0.96107200	-2.39692500	1.66852300
C	1.71359800	-3.57223300	1.55260100
C	-0.41084500	-2.46660000	1.93687900
C	1.08512400	-4.80799000	1.68929000
H	2.78696500	-3.52156500	1.35759400
C	-1.02740000	-3.70885400	2.08855300
H	-0.99752700	-1.54865800	2.02506600
C	-0.28325100	-4.88116800	1.95969400
H	1.67445900	-5.72374200	1.59270100
H	-2.10024400	-3.75489000	2.29404000
H	-0.76842200	-5.85432700	2.07048100
F	-2.02744400	5.16864000	2.21670500
F	-2.13075600	5.91327800	0.20339500
F	-3.63456100	4.55179900	0.92507100
F	-5.71951300	-1.07280800	-0.64261900
F	-4.97073900	-2.75947700	0.46297100
F	-5.22557400	-2.90126700	-1.66768700
C	-2.06361200	2.45018700	0.17708300
H	-3.14275800	2.31645600	0.24540800
C	-3.06973200	-0.31587100	-0.48779800
H	-3.81503800	0.37091200	-0.08705800

PBE0-D3 (E_h): -4109.105019

Therm. corr. G-E (E_h): 0.296931

IM-2a

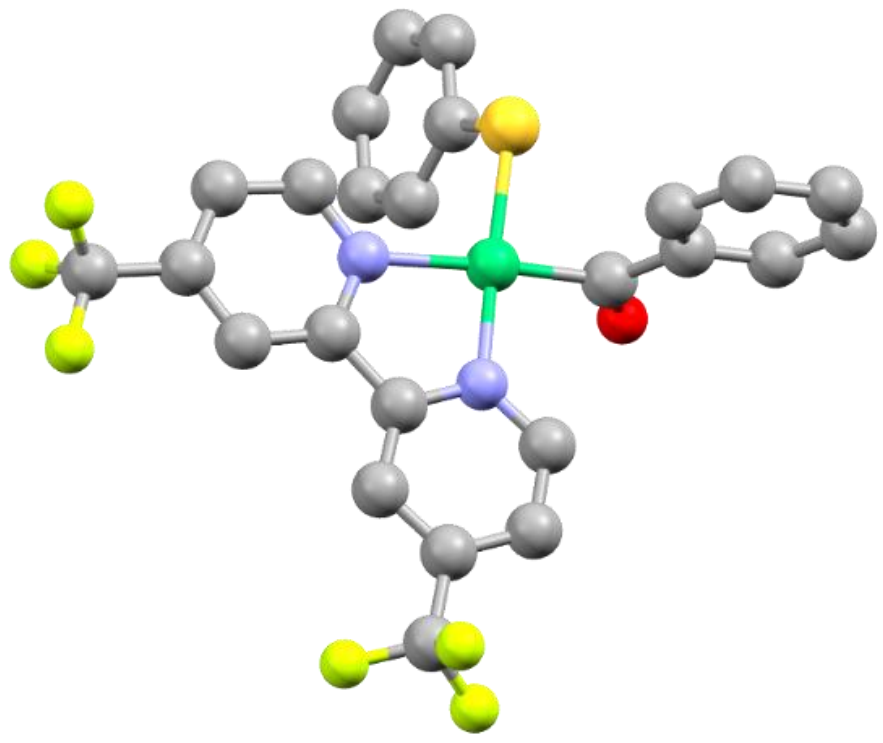

0 1

N	0.46280500	-1.11939400	0.50485300
C	0.42917100	-2.38393400	0.92520700
C	1.58323500	-3.12352700	1.15222600
C	2.81285500	-2.50639500	0.93435500
C	2.85446400	-1.18552500	0.50545600
C	1.64672700	-0.51776900	0.29902300
C	1.54624600	0.88535800	-0.14176000
C	2.65639500	1.68584200	-0.40426000
C	2.45570100	3.00255900	-0.79885400
C	1.15732300	3.48612700	-0.92359500
C	0.10377300	2.62379200	-0.64950200
N	0.28505800	1.35435200	-0.26877900
C	3.63005900	3.91249900	-1.05779700
C	4.08147100	-3.28976700	1.15954000
F	3.86662300	4.70069500	-0.00462300
F	3.40666000	4.70758800	-2.10508500
F	4.74665500	3.22604400	-1.30015800
F	5.17253700	-2.54100000	1.00604700
F	4.17112700	-4.31114600	0.30263300
F	4.11472600	-3.80532100	2.39117000
Ni	-1.10893300	0.05370200	0.09821100
H	-0.56602600	-2.80688200	1.08912900

H	1.51167400	-4.15833900	1.49209800
H	3.81020800	-0.68986800	0.33830000
H	3.66521400	1.28683700	-0.30444400
H	0.94981200	4.51192700	-1.23188300
H	-0.92375400	2.97388700	-0.74700000
C	-5.03455100	3.53829300	1.11100300
C	-4.65699300	3.51310500	2.45672700
C	-3.58637800	2.71726600	2.86839600
C	-2.89711100	1.94398500	1.93519400
C	-3.27134200	1.96080400	0.58665900
C	-4.34251800	2.76836000	0.18041800
C	-2.51964800	1.14688700	-0.43419200
S	-2.74178100	-1.36378800	0.61847000
O	-2.78966200	1.24598900	-1.61697000
C	-2.77350400	-2.54887900	-0.68878100
C	-3.67166100	-3.63105300	-0.60232500
C	-3.73430400	-4.58990000	-1.60996400
C	-2.90589200	-4.49926500	-2.73189100
C	-2.01456000	-3.43030300	-2.82913400
C	-1.94589100	-2.46594400	-1.82353100
H	-5.87280700	4.16333100	0.79073000
H	-5.19998400	4.11856400	3.18770900
H	-3.28889100	2.70045300	3.92022600
H	-2.05407500	1.31831100	2.24781700
H	-4.61806700	2.77541400	-0.87745900
H	-4.32736300	-3.71555000	0.26913900
H	-4.44112100	-5.41963000	-1.51651000
H	-2.95766900	-5.25279900	-3.52193500
H	-1.36047900	-3.34019200	-3.70146100
H	-1.24915700	-1.62857400	-1.91820000

PBE0-D3 (E_h): -3649.092797

Therm. corr. G-E (E_h): 0.295979

IM-2b

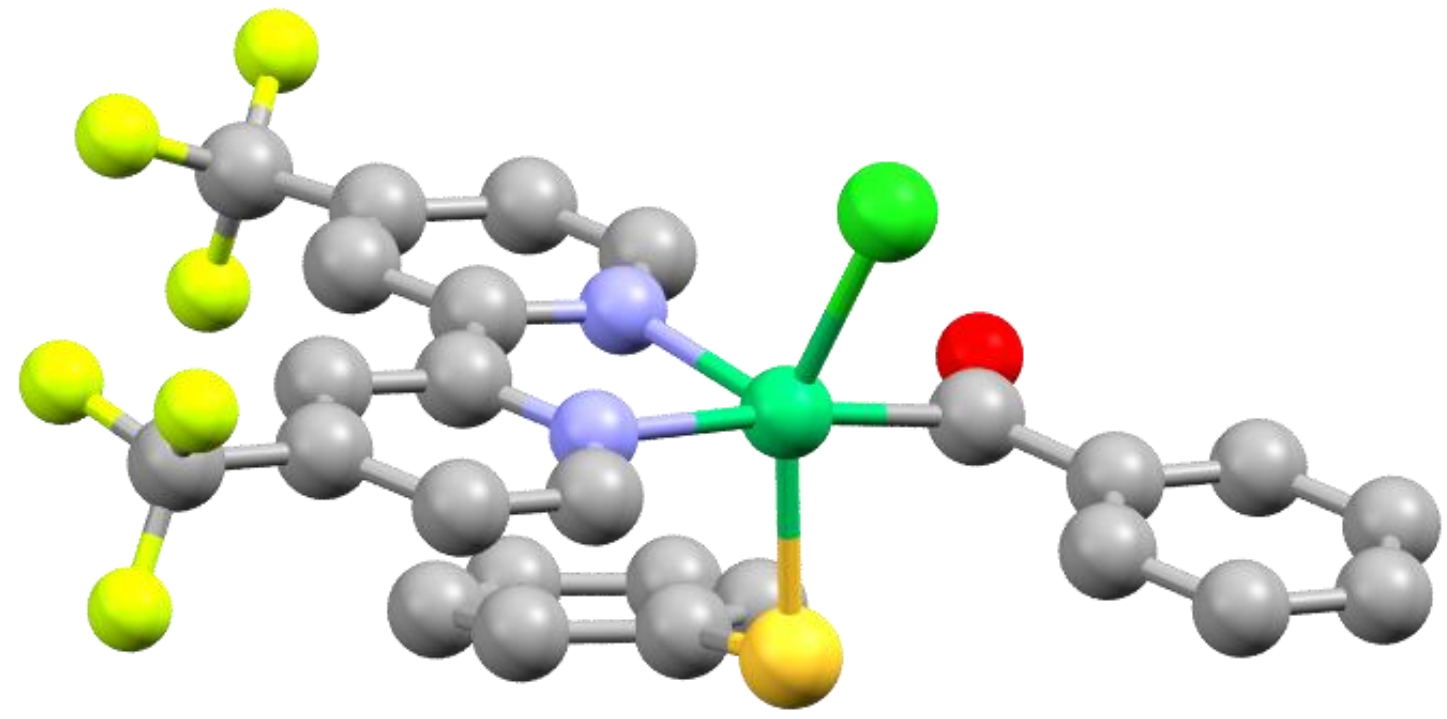

0 2

N	-0.20601400	-0.77906800	-0.80965800
C	-0.09491500	-2.06960600	-1.11573200
C	-1.20049500	-2.90530000	-1.22187200
C	-2.46009100	-2.35654700	-1.00552200
C	-2.58176900	-1.00261300	-0.72004300
C	-1.42070800	-0.23665000	-0.62318300
C	-1.43074200	1.19654100	-0.26491700
N	-0.22281800	1.74522600	-0.09038400
C	-0.10459700	3.02704600	0.24315900
C	-1.21105300	3.84590500	0.42816200
C	-2.47466300	3.28525100	0.24978300
C	-2.59666200	1.94761500	-0.10283400
H	0.91070200	3.41300800	0.36513800
H	-1.08065700	4.89433000	0.70112800
H	-3.57973100	1.50344100	-0.25403200
H	-3.56237000	-0.55922500	-0.55087300
H	-1.06608400	-3.96247700	-1.45427800
H	0.92139400	-2.44215500	-1.25406900
C	-3.69845400	4.13908300	0.46230200
C	-3.67520300	-3.24643700	-0.99823000
F	-3.85584700	4.43541400	1.75613900
F	-4.81234600	3.53320500	0.05307500
F	-3.60033700	5.29540800	-0.19743700

F	-3.60118400	-4.19211600	-1.93526900
F	-3.79481500	-3.86812800	0.18182600
F	-4.80053700	-2.56094400	-1.20018900
Ni	1.36342100	0.48531400	-0.39615000
Cl	1.90894000	1.30001500	-2.41602000
O	2.89064300	-1.76271600	-0.84747400
S	1.79097800	-0.09670600	1.79396600
C	2.96962100	-0.61780000	-0.49750400
C	4.26465300	0.09413800	-0.28939000
C	4.32758800	1.43145400	0.11831100
C	5.56073300	2.04953900	0.29515800
C	6.73998700	1.33745800	0.06257600
C	6.68248200	0.00516300	-0.34728200
C	5.44875100	-0.61589900	-0.52382900
H	5.38675300	-1.65835900	-0.84359500
H	7.60399900	-0.55329200	-0.53037200
H	7.70798300	1.82569600	0.20185900
H	5.60426800	3.09331600	0.61513200
H	3.41299500	2.00366400	0.29842700
C	0.81552000	-1.56223300	1.97040600
C	-0.55874600	-1.47970400	2.24713400
C	-1.32652600	-2.63565000	2.36476100
C	-0.73411000	-3.89235500	2.22079600
C	0.63490700	-3.98299400	1.96919500
C	1.40732800	-2.82786300	1.84578900
H	2.47671900	-2.89993200	1.63791400
H	1.10866900	-4.96252500	1.86113800
H	-1.34080600	-4.79767800	2.30339800
H	-2.39872600	-2.55500900	2.56226100
H	-1.02327700	-0.49673400	2.35553200

PBE0-D3 (E_h): -4109.112794

Therm. corr. G-E (E_h): 0.296798

7. Unsuccessful Substrates

Other acyl sources:

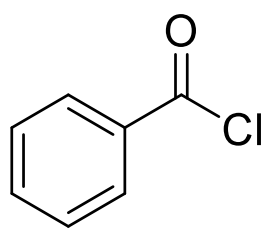

n. d.

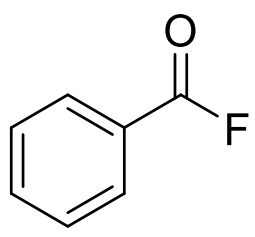

5%

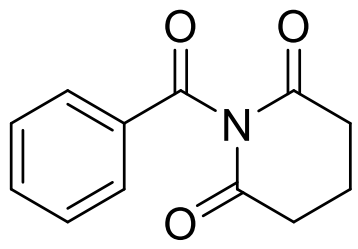

4%

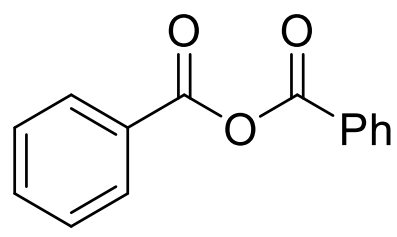

11%

Thioesters

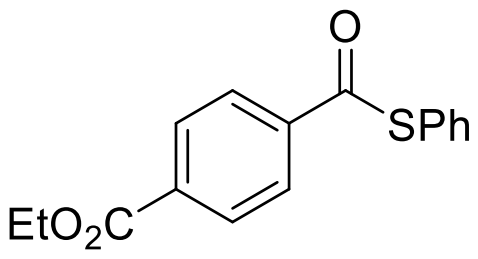

low conversion

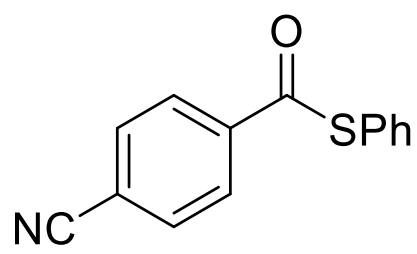

low conversion

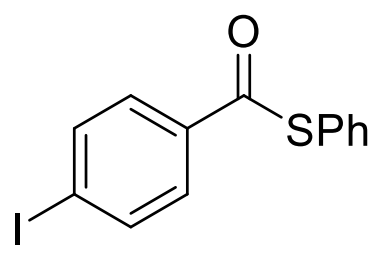

high conversion, but ~1:1:1 mixture of product and protodehalogenated SM and product

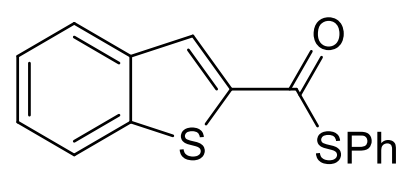

16% (isolated)

Acrylates

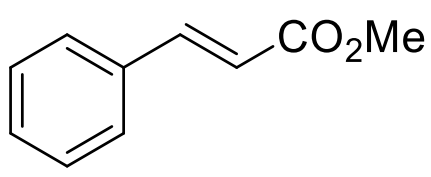

low conversion

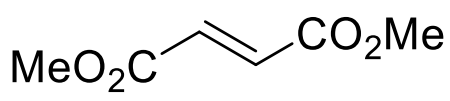

low conversion

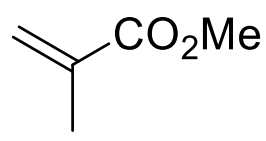

low conversion

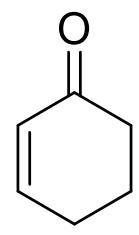

ca. 20% yield (GC)

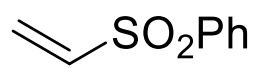

low conversion

Styrenes

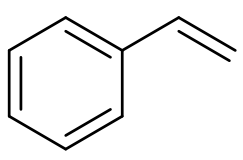

n. d.

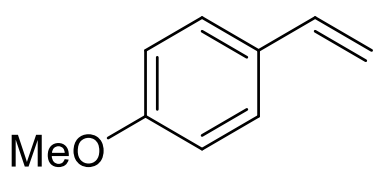

n. d.

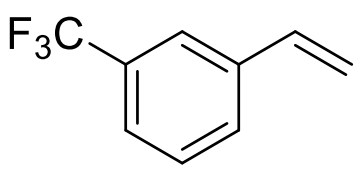

n. d.

8. References

1. G. R. Fulmer, A. J. M. Miller, N. H. Sherden, H. E. Gottlieb, A. Nudelman, B. M. Stoltz, J. E. Bercaw and K. I. Goldberg, NMR Chemical Shifts of Trace Impurities: Common Laboratory Solvents, Organics, and Gases in Deuterated Solvents Relevant to the Organometallic Chemist, *Organometallics*, 2010, **29**, 2176-2179.
2. H. Cao, X. Liu, F. Bie, Y. Shi, Y. Han, P. Yan, M. Szostak and C. Liu, Rh(I)-Catalyzed Intramolecular Decarbonylation of Thioesters, *J. Org. Chem.*, 2021, **86**, 10829-10837.
3. B. Neises and W. Steglich, Simple Method for the Esterification of Carboxylic Acids, *Angew. Chem., Int. Ed.*, 1978, **17**, 522-524.
4. C. Hansch, A. Leo and R. W. Taft, A survey of Hammett substituent constants and resonance and field parameters, *Chem. Rev.*, 1991, **91**, 165-195.
5. L. Gao, Y.-X. Xin, P. Lv, X. Xie, Y.-Y. Jiang and Y. Liu, Nickel/Photoredox Catalyzed Aryl-Alkyl Cross-Coupling with Alkyl Boronic Esters as Radical Precursors, *Chem. Eur. J.*, 2025, **31**, e202404253.
6. F. Bie, X. Liu, H. Cao, Y. Shi, T. Zhou, M. Szostak and C. Liu, Pd-Catalyzed Double-Decarbonylative Aryl Sulfide Synthesis through Aryl Exchange between Amides and Thioesters, *Org. Lett.*, 2021, **23**, 8098-8103.
7. W.-P. Mai, Y. Zhao, M.-X. Lv, W.-R. Zhang, Y.-M. Xiao, J.-W. Yuan and L.-R. Yang, Nickel-Catalyzed Synthesis of Thioesters from Amides and Disulfides, *Eur. J. Org. Chem.*, 2024, **27**, e202400026.
8. J. Su, A. Chen, G. Zhang, Z. Jiang and J. Zhao, Photocatalytic Phosphine-Mediated Thioesterification of Carboxylic Acids with Disulfides, *Org. Lett.*, 2023, **25**, 8033-8037.
9. S. Yang, X. Yu and M. Szostak, Divergent Acyl and Decarbonylative Liebeskind–Srogl Cross-Coupling of Thioesters by Cu-Cofactor and Pd–NHC (NHC = N-Heterocyclic Carbene) Catalysis, *ACS Catal.*, 2023, **13**, 1848-1855.
10. L.-W. Pan, N. Taniguchi, M. Hyodo and I. Ryu, CO-Boosted Protocol for the Photocatalytic C–H Thiolation of Aldehydes Using Phenylthiobenzenesulfonate, *Eur. J. Org. Chem.*, 2024, **27**, e202400138.
11. J. Xu, F. Lu, L. Sun, M. Huang, J. Jiang, K. Wang, D. Ouyang, L. Lu and A. Lei, Electrochemical reductive cross-coupling of acyl chlorides and sulfinic acids towards the synthesis of thioesters, *Green Chem.*, 2022, **24**, 7350-7354.
12. C. Wang, S. Mavila, B. T. Worrell, W. Xi, T. M. Goldman and C. N. Bowman, Productive Exchange of Thiols and Thioesters to Form Dynamic Polythioester-Based Polymers, *ACS Macro Lett.*, 2018, **7**, 1312-1316.
13. I. H. Lindenmaier, R. C. Richter, L. Renz, M. T. Vochezer, M. Schier, P. Faßnacht, M. Ströbele and I. Fleischer, Nickel-catalyzed cross-electrophile-coupling of thioesters with alkyl bromides to form ketones under mechanochemical conditions, *Chem. Commun.*, 2026, **62**, 5284-5288.
14. S. S. a. A. R. Saloni Gangal, A Mild and Efficient Synthesis of Thiol Esters from Long-Alkenyl Chain Carboxylic Acids and their Antimicrobial Evaluation, *Rasayan J. Chem.*, 2009, **2**, 682-687.
15. E. Cassera and M. Fagnoni, Aldehydes as CO Releasing Molecules: In Situ and Ex Situ Giese Reactions and Palladium-Catalyzed Aminocarbonylations, *Org. Lett.*, 2025, **27**, 11999-12005.

16. K. E. Price, S. J. Broadwater, B. J. Walker and D. T. McQuade, A New Interpretation of the Baylis−Hillman Mechanism, *J. Org. Chem.*, 2005, **70**, 3980-3987.

17. A. Bechtoldt and L. Ackermann, Ruthenium(II)biscarboxylate-Catalyzed Hydrogen-Isotope Exchange by Alkene C−H Activation, *ChemCatChem*, 2019, **11**, 435-438.

18. Y.-M. Cai, K.-N. Yuan, P.-Y. Huang, T. Xie and M. Shang, Quaternary Carbon Centers via Electrochemical Direct Dehydroxylative Alkylation, *Angew. Chem. Int. Ed.*, 2026, **65**, e20816.

19. Y. Dong, R. Li, J. Zhou and Z. Sun, Synthesis of Unsymmetrical 1,4-Dicarbonyl Compounds by Photocatalytic Oxidative Radical Additions, *Org. Lett.*, 2021, **23**, 6387-6390.

20. J. Jiang, J. Liu, L. Yang, Y. Shao, J. Cheng, X. Bao and X. Wan, Cu-based carbene involved in a radical process: a new crossover reaction to construct γ-peroxy esters and 1,4-dicarbonyl compounds, *Chem. Commun.*, 2015, **51**, 14728-14731.

21. F. Gao, Z.-Y. Liao, Y.-H. Ye, Q.-H. Yu, C. Yang, Q.-Y. Luo, F. Du, B. Pan, W.-W. Zhong and W. Liang, Photomediated Hydro(deutero)acylation of Olefins by Decarboxylative Addition of α-Oxocarboxylic Acids, *J. Org. Chem.*, 2024, **89**, 2741-2747.

22. K.-K. Niu, R.-Z. Zhang, X.-Z. Yang, C.-Q. Ma, H. Liu, S. Yu and L.-B. Xing, Nitrogen-doped Carbon Dots as Efficient Photocatalysts for High Selectivity of Dehalogenative Oxyalkylation of Styrene, *ChemSusChem*, 2024, **17**, e202301686.

23. T. Iwai, T. Nakai, M. Mihara, T. Ito, T. Mizuno and T. Ohno, Pd-Catalyzed Cross-Coupling Reactions of Pyridine Carboxylic Acid Chlorides with Alkylzinc Reagents, *Synlett*, 2009, **2009**, 1091-1094.

24. E.-A. Jo and C.-H. Jun, One-Pot Synthesis of Oxo Acid Derivatives by RhI-Catalyzed Chelation-Assisted Hydroacylation, *Eur. J. Org. Chem.*, 2006, **2006**, 2504-2507.

25. M. D. Vu, M. Das and X.-W. Liu, Direct Aldehyde Csp2−H Functionalization through Visible-Light-Mediated Photoredox Catalysis, *Chem. Eur. J.*, 2017, **23**, 15899-15902.

26. T. Kawamoto, S. Uehara, H. Hirao, T. Fukuyama, H. Matsubara and I. Ryu, Borohydride-Mediated Radical Addition Reactions of Organic Iodides to Electron-Deficient Alkenes, *J. Org. Chem.*, 2014, **79**, 3999-4007.

27. D. Xia, W. Wang, X. Xu, Y. Zhu, Q. Li, J. Wang, Y. Zhang and W.-D. Zhang, Access to Diverse Carbonyl Compounds by Catalyst-Free and Photoinduced Aerobic Cleavage of C=N Bonds, *Chem. Eur. J.*, 2024, **30**, e202402607.

28. Y. Zhou, Y.-Q. He, X. Nie, L. Lu, X.-R. Song, Z.-Z. Zhou, W.-F. Tian and Q. Xiao, Ir/Co dual catalyzed hydroacylation of electron-deficient alkenes overcoming redox potential limitations, *Org. Chem. Front.*, 2024, **11**, 4269-4274.

29. J. Brauer, E. Quraishi, L. M. Kammer and T. Opatz, Nickel-Mediated Photoreductive Cross Coupling of Carboxylic Acid Derivatives for Ketone Synthesis, *Chem. Eur. J.*, 2021, **27**, 18168-18174.

30. C. G. Page, S. J. Cooper, J. S. DeHovitz, D. G. Oblinsky, K. F. Biegasiewicz, A. H. Antropow, K. W. Armbrust, J. M. Ellis, L. G. Hamann, E. J. Horn, K. M. Oberg, G. D. Scholes and T. K. Hyster, Quaternary Charge-Transfer Complex Enables Photoenzymatic Intermolecular Hydroalkylation of Olefins, *J. Am. Chem. Soc.*, 2021, **143**, 97-102.

31. D. Spinnato, B. Schweitzer-Chaput, G. Goti, M. Ošeka and P. Melchiorre, A Photochemical Organocatalytic Strategy for the α-Alkylation of Ketones by using Radicals, *Angew. Chem. Int. Ed.*, 2020, **59**, 9485-9490.

32. A. G. Edmonds, D. A. Walsh, K. Ermanis and J. D. Cuthbertson, Metal-Free Direct Electrochemical Deoxygenation of Benzylic Alcohols, *ACS Sustain. Chem. Eng.*, 2025, **13**, 21101-21110.

33. Z.-X. He, S.-x. Tan, Z. Dan, G.-j. Dong, Y.-l. Liu, L.-Y. Ma and W. Zhao, Design, synthesis, and biological evaluation of novel pyridazinone-based EED inhibitors for prostate cancer, *Eur. J. Med. Chem.*, 2025, **296**, 117837.

34. M. J. Frisch, G. W. Trucks, H. B. Schlegel, G. E. Scuseria, M. A. Robb, J. R. Cheeseman, G. Scalmani, V. Barone, G. A. Petersson, H. Nakatsuji, X. Li, M. Caricato, A. V. Marenich, J. Bloino, B. G. Janesko, R. Gomperts, B. Mennucci, H. P. Hratchian, J. V. Ortiz, A. F. Izmaylov, J. L. Sonnenberg, D. Williams-Young, F. Ding, F. Lipparini, F. Egidi, J. Goings, B. Peng, A. Petrone, T. Henderson, D. Ranasinghe, V. G. Zakrzewski, J. Gao, N. Rega, G. Zheng, W. Liang, M. Hada, M. Ehara, K. Toyota, R. Fukuda, J. Hasegawa, M. Ishida, T. Nakajima, Y. Honda, O. Kitao, H. Nakai, T. Vreven, K. Throssell, J. A. Montgomery, J. E. Peralta, F. Ogliaro, M. J. Bearpark, J. J. Heyd, E. N. Brothers, K. N. Kudin, V. N. Staroverov, T. A. Keith, R. Kobayashi, J. Normand, K. Raghavachari, A. P. Rendell, J. C. Burant, S. S. Iyengar, J. Tomasi, M. Cossi, J. M. Millam, M. Klene, C. Adamo, R. Cammi, J. W. Ochterski, R. L. Martin, K. Morokuma, O. Farkas, J. B. Foresman and D. J. Fox, *Gaussian 16, Revision C.01*, Gaussian Inc., Wallingford CT, 2016.

35. C. Adamo and V. Barone, Toward reliable density functional methods without adjustable parameters: The PBE0 model, *J. Chem. Phys.*, 1999, **110**, 6158-6170.

36. S. Grimme, S. Ehrlich and L. Goerigk, Effect of the damping function in dispersion corrected density functional theory, *J. Comput. Chem.*, 2011, **32**, 1456-1465.

37. F. Weigend and R. Ahlrichs, Balanced basis sets of split valence, triple zeta valence and quadruple zeta valence quality for H to Rn: Design and assessment of accuracy, *Phys. Chem. Chem. Phys.*, 2005, **7**, 3297-3305.

38. F. Weigend, Accurate Coulomb-fitting basis sets for H to Rn, *Phys. Chem. Chem. Phys.*, 2006, **8**, 1057-1065.

39. K. Fukui, Formulation of the reaction coordinate, *J. Phys. Chem.*, 1970, **74**, 4161-4163.

40. K. Fukui, The path of chemical reactions - the IRC approach, *Acc. Chem. Res.*, 1981, **14**, 363-368.

41. A. V. Marenich, C. J. Cramer and D. G. Truhlar, Universal Solvation Model Based on Solute Electron Density and on a Continuum Model of the Solvent Defined by the Bulk Dielectric Constant and Atomic Surface Tensions, *J. Phys. Chem. B*, 2009, **113**, 6378-6396.

42. F. De Proft, C. Van Alsenoy, A. Peeters, W. Langenaeker and P. Geerlings, Atomic charges, dipole moments, and Fukui functions using the Hirshfeld partitioning of the electron density, *J. Comput. Chem.*, 2002, **23**, 1198-1209.

43. A. V. Marenich, S. V. Jerome, C. J. Cramer and D. G. Truhlar, Charge Model 5: An Extension of Hirshfeld Population Analysis for the Accurate Description of Molecular Interactions in Gaseous and Condensed Phases, *J. Chem. Theory Comput.*, 2012, **8**, 527-541.
